# A hidden human proteome encoded by ‘non-coding’ genes

**DOI:** 10.1093/nar/gkz646

**Published:** 2019-07-24

**Authors:** Shaohua Lu, Jing Zhang, Xinlei Lian, Li Sun, Kun Meng, Yang Chen, Zhenghua Sun, Xingfeng Yin, Yaxing Li, Jing Zhao, Tong Wang, Gong Zhang, Qing-Yu He

**Affiliations:** 1 Key Laboratory of Functional Protein Research of Guangdong Higher Education Institutes, Institute of Life and Health Engineering, College of Life Science and Technology, Jinan University, Guangzhou 510632, China; 2 Laboratory of Veterinary Pharmacology, College of Veterinary Medicine, South China Agricultural University, Guangzhou 510642, China

## Abstract

It has been a long debate whether the 98% ‘non-coding’ fraction of human genome can encode functional proteins besides short peptides. With full-length translating mRNA sequencing and ribosome profiling, we found that up to 3330 long non-coding RNAs (lncRNAs) were bound to ribosomes with active translation elongation. With shotgun proteomics, 308 lncRNA-encoded new proteins were detected. A total of 207 unique peptides of these new proteins were verified by multiple reaction monitoring (MRM) and/or parallel reaction monitoring (PRM); and 10 new proteins were verified by immunoblotting. We found that these new proteins deviated from the canonical proteins with various physical and chemical properties, and emerged mostly in primates during evolution. We further deduced the protein functions by the assays of translation efficiency, RNA folding and intracellular localizations. As the new protein UBAP1-AST6 is localized in the nucleoli and is preferentially expressed by lung cancer cell lines, we biologically verified that it has a function associated with cell proliferation. In sum, we experimentally evidenced a hidden human functional proteome encoded by purported lncRNAs, suggesting a resource for annotating new human proteins.

## INTRODUCTION

A fundamental question in biology is how many proteins a human genome can encode. To date, 19 467 genes are annotated as protein-coding genes, among which 17 470 proteins have been evidenced at protein level (1). A considerable fraction of the rest 98% of the human genome can be transcribed into ‘non-coding RNAs’ (ncRNA). Other than the miRNA, tRNA, snoRNA and rRNA, there are ∼2600 annotated long non-coding RNA (lncRNA) genes that can be transcribed into RNAs longer than 200nt (2). Until 2013, it had been widely believed that these RNAs do not encode proteins (3), while regulating translation (4,5).

It has been widely debated regarding whether the lncRNAs can encode proteins. Banfai *et al.* have proposed that ribosomes are able to differentiate coding genes from non-coding ones as most predicted open reading frames (ORFs) have upstream stop codons that lead to early translation termination and very short peptide production (6). With the evidence of full-length translating mRNA analyses, we have reported that over 1300 such lncRNAs are bound with ribosomes (7). We have accordingly proposed that the translating lncRNAs may produce new proteins (7), which, if substantiated, may represent a hidden human proteome. On the contrary, Gutman *et al.* proposed with computational approaches that the lncRNAs lack ribosome release behavior at the stop codon, which distinguishes them from coding RNAs, and thus concluded that the lncRNAs do not encode proteins (3). Later on, the same group proposed pervasive translation outside the conserved coding regions in yeast, but they provided no protein evidence direct for such a conclusion (8). With bioinformatics rationale, Ji *et al.* have recently proposed that numerous lncRNAs, 5′UTRs and pseudogenes can be translated into peptides and potentially even functional proteins (9). Jackson et al recently found in mice that short and non-ATG-initiated open reading frames (ORFs) in non-protein coding genes could express proteins (10).

Indeed, mis-annotations of individual ncRNAs have been reported with their protein products since a decade ago, such as *CLUU1* (11) and *ESRG* (12), although some ncRNAs only encode short peptides (13–17). The discovery of these individual mis-annotations implicatively favors our hypothesis that a hidden human proteome encoded by purported ‘non-coding’ RNAs may exist. Recently, Heesch *et al.* combined ribosome profiling with MS analysis and detected 128 peptides from 86 human microproteins/micropeptides encoded by non-coding genes (18). These findings implicate that translating mRNA analysis has potentials to direct new protein discovery.

Based on above rationales, we believe that the lncRNA-encoded proteins are not merely individual mis-annotations, the existence of these new proteins should be at proteome level. To address this question, we have to: (a) find protein evidence of the lncRNA-coded proteins in a genome-wide scale; (b) assess whether these ‘new proteins’ are potentially functional. As such, in this study, we employed stringent criteria and multiple technologies to experimentally evidence the systematic existence of lncRNA-encoded new proteins.

## MATERIALS AND METHODS

### Cell culture, mRNA-seq and full-length translating mRNA sequencing

Human HBE, A549 and H1299 cells were purchased from American Type Culture Collections (ATCC, Rockville, MD, USA), the human hepatoma Hep3B, MHCC97H, MHCCLM3 and MHCCLM6 cells were acquired from Professor Yinkun Liu, Fudan University (19). The cells were cultured as we previously described (7,19); all cells were detected mycoplasma negative during maintenance and upon experiments. mRNA-seq and the full-length translating mRNA-seq of MHCCLM6 cells were performed according to the protocol as we previously described (19). The raw data was deposited in Gene Expression Omnibus database, accession number GSE79539.

### Ribosome profiling

The ribosome footprints (RFP) extraction was performed as we previously reported (20). In brief, RFP samples were subjected to rRNA depletion with a Ribo-Zero™ Magnetic Kit (Human/Mouse/Rat) (Epicenter, Madison, WI, USA), by following the manufacturer's instructions. Dynabeads^®^ MyOne™ Streptavidin C1 (Thermo Fisher Scientific, Shanghai, China) and Biotinylated probes (20 μm and 0.5 μl per sample) were used to further remove the rRNA and miR-21 fragments. The reaction for each sample was taken place in a 20 μl volume, consisting of probes (20 μm, 0.5 μl), 2 μl 20× SSC (0.3 M Na_3_C_6_H_5_O_7_·2H_2_O and 3 M NaCl) and H_2_O. Samples were denatured for 90 s at 95°C, followed by programmed cooling down at a rate of 0.1°C/s to 37°C and a continuous incubation at 37°C for 15 min. At the same time, 50 μl Dynabeads were washed by 1× Bind/Wash buffer (2× Bind/Wash buffer, 2 M NaCl, 1 mM EDTA, 5 mM Tris (pH7.5), 0.2% (v/v) Triton X-100) twice, resuspended in 20 μl 2× Bind/Wash buffer, and mixed with the rRNA-depleted RFP samples. After an incubation at 37°C for 30 min, RFPs were magnetically isolated and purified by ethanol precipitation. The probe sequences of were listed in Supplementary Table S8.

Sequencing libraries were constructed by following the guide for the NEBNext Multiplex Small RNA Library Prep Set for Illumina (NEB, Beijing, China). Briefly, the multiplex 3′ SR adapters were ligated to RFPs and hybridized with reverse transcription primers. The reverse transcription was performed after the ligation of the multiplex 5′ SR adapter. Fifteen cycles of PCR were performed to enrich those DNA fragments that have adapters on both ends and the library fragments were size selected by the 6% PAGE-gel extraction. Purified libraries were sequenced by an Illumina Hiseq-2500 sequencer for 50 cycles. High quality reads that passed the Illumina quality filters were kept for the sequence analysis. The raw data was deposited in Gene Expression Omnibus database, accession number GSE79539.

### Reference sequences

The NCBI RefSeq-RNA database (downloaded on 21 May 2014) was used for human transcriptome reference sequences. The genomic reference sequences are listed in Supplementary Table S9.

### Sequence analysis

The mRNA and full-length translating mRNA sequencing (RNC-seq) datasets (GEO accession numbers: GSE42006, GSE46613, GSE48603 and GSE49994) were taken from our previous publications (7,19–21). For mRNA-seq and full-length translating mRNA-seq data, reads were mapped to human transcriptome reference sequences using FANSe2 with the parameters –L80 –E5 –I0 –S14 –B1 –U0 (22). For RFP datasets, adapter sequences were removed from all reads. Reads were further truncated at their first nucleotides, whose Phred quality scores were less than 10. RFP reads were mapped to the human transcriptome reference sequences using FANSe2 with the parameters –L55 –E4 –I1 –U1 –S10 (22). Genes with read counts > = 10 were considered as true expression (7,23).

### Bioinformatics

The ORF prediction of the translating lncRNAs was performed using MATLAB 2013a. The folding energy of the mRNA near the start codon using a ±19nt sliding window was determined according to (24) using the ‘rnafold’ function in MATLAB 2013a. The amino acids were classified as nonpolar amino acid including alanine, valine, leucine, isoleucine, proline, phenylalanine, tryptophan, methionine; uncharged polar amino acid including serine, threonine, tyrosine, glutamine, asparagine, cysteine, glycine; negative charged amino acid including aspartic acid, glutamic acid; and positive charged amino acid including arginine, lysine, histidine. NCBI BLAST+ 2.2.31 software package (win64 version) was used for the homology search of the new proteins.

### Shotgun proteomics datasets

The proteomics datasets of HCC cell lines and Hela cells were downloaded from the ProteomeXchange Consortium (http://proteomecentral.proteomexchange.org) with the following respective identifiers, PXD000529, PXD000533, PXD000535 (19) and PXD001305 (25). The human lung cell proteomics data are available from the iProX database (http://www.iprox.org, accession number: IPX00076200); and the MS raw data for low molecular weight proteins are publicly available in iProX (accession number: IPX00076300).

### In-gel digestion of low molecular weight proteins

Protein samples were separated in Novex^®^ 10–20% Tricine gels (Life Technologies, Carlsbad, CA, USA). Visualized by silver staining (Supplementary Figure S7), 5–25 kDa bands were cut into gel pieces and digested as we previously described (7,26). Peptide samples were reconstituted in Solvent A (0.1% formic acid and 2% ACN) and analyzed in a Triple TOF 5600 MS (AB SCIEX, Framingham, CA, USA) as we previously described (26).

### Database construction

To create cell-specific reference protein databases, we used the full-length translating mRNA-seq data to include protein products from all translating genes, including protein evidence level 1 (PE1) proteins (defined in neXtProt database) and predicted ORFs (protein product ≥ 50nt) of the translating lncRNAs. Small protein databases were generated for all proteins less than 25 kDa. The number of entries of the cell-specific reference databases are listed in Supplementary Table S12.

### Database searches

Mascot (version 2.5.1), MaxQuant (version 1.5.2.8) and X!Tandem (version 1.7.18) (27) were used for shotgun MS data searches either stand-alone or in combinations described as follows. All searches were against cell-specific protein databases. The common search parameters included: enzyme, trypsin; fixed modification, carbamidomethyl (C); variable modifications, oxidation (M), Gln → pyro-Glu (N-terminus), and acetyl (N-terminus); two missed cleavage sites were allowed. Specific to MS analyzers, mass tolerance parameters were set as follows (7,26): Triple TOF 5600 data (15 ppm for MS, 0.05 Da for MS/MS), Orbitrap Q Exactive data (10 ppm for MS and 0.02 Da for MS/MS), and LTQ-Orbitrap data (20 ppm for MS and 0.5 Da for MS/MS).

We adopted the criteria for confident identification with false discovery rate (FDR) <0.01 at protein level for Mascot searches, and FDR <0.01 at PSM, peptide and protein levels in MaxQuant and X!Tandem searches. To control FDR, the .dat file resulted from Mascot searches were re-analyzed by Scaffold Q + S (version 4.4.6) to acquire protein lists with the protein level FDR <1%. For those resulted from the X!tandem search, PeptideShaker (version 1.1.4) was used for the FDR control (27). Data from label-free MS analyses were searched with all three search engines, while those the SILAC-based MS data were searched with MaxQuant solely.

To apply stringent quality control, we performed sequence similarity inspection considering the amino acids with the same molecular weights caused by certain post-translational modifications as we described previously (26). In addition, only unique peptides with at least 9 residues were used for protein identifications (26). Proteins that do not belong to PE1–5 proteins (the ‘protein products of known coding genes’ according to the Swiss-Prot database) (28) were defined as ‘new proteins’.

### Protein abundance analysis

Label-free MS data were quantified with the iBAQ (intensity-based absolute quantification) algorithm (29) as provided in MaxQuant. The displayed protein abundance values were log_10_ transformed.

### Smith-Waterman analysis

Smith-Waterman analysis was conducted to rule out the possible single amino acid variation derived misinterpretation of new protein discovery, following the procedure as we previously reported with minor modifications (26). In brief, we generated a background reference protein sequence collection, by combining the RefSeq protein sequences and our predicted ORF sequences of NR genes (virtually translated into amino acid sequences), as well as the UniProt-SwissProt protein sequences. Smith-Waterman alignment was then performed on all candidate unique peptides against such a collection, with isoleucine and leucine considered as identical amino acids. Only those peptides with at least two mismatches/indels were considered true unique peptides for the new proteins to rule out very similar identifications and potential products of non-distinguishable pseudogenes.

### Non-synthetic peptide - based multiple reaction monitoring (MRM) analysis

Peptide sequences were imported into Skyline v3.5 (MacCoss Lab, University of Washington) to generate the MRM transitions, considering both 2+ and 3+ precursor charges with only the y-type fragment ions. As a result, all transitions with default optimized of Declustering Potential (DP) and Collision Energy (CE) settings were exported.

The tryptic peptides mixtures from sample A (97H) and B (LM3) were analyzed either in bulk or in 12 fractions by high pH (pH 10.0 with ammonium formate buffer) RP-LC.

The MRM data acquisition was performed on a SCIEX QTRAP 6500+ system interfaced with an Eksigent 425 Nano-HPLC system via a NanoSpray Source III. Peptides reconstituted with solvent A (2% ACN, 0.1% FA) were first loaded in a trap column, followed by the separation through a nano column (75 μm × 15 cm, C18, 3 μm, 120 Å). The flow rate was set to 300 nl/min over a 90-min multi-segment gradient on solvent B (98% ACN, 0.1% FA): 0 min 5%, 0.1 min 12%, 62 min 30%, 68 min 40%, 72 min 90%, 82 min 90%, 82.1 min 5% and 90 min 5%. The MS analysis was carried out in the positive ionization mode, using the following settings: ion spray voltage, 2400 V; curtain gas flow (nitrogen), 20 psi; Gas1, 6 psi; Gas2, 0 psi; IHT, 150°C; EP, 10 V; CXP, 13 V and CAD, medium-high.

The MRM experiments were sequentially conducted with the following design. The first general screening was focused on the transitions in the unfractionated peptide mixtures. The positive-response transitions were collected and combined to run the MRM confirmation experiments at the second round, using the peptides mixtures before fractionated as sample input. We further performed the third-round MRM to specifically analyze all the negative-response transitions using the fractionations of each sample.

All data files were imported into Skyline and manually screened out the positive MRM XIC spectra for each corresponding peptide. The MS raw data are publicly available in iProX (accession number: IPX00076300).

### Parallel reaction monitoring (PRM)

Heavy-labeled standard peptides (heavy-peptides) were synthesized by the JPT Peptide Technologies (Berlin, Germany) in the SpikeTides_L mode, with the following technical requirements: (i) ≤20 aa in length and (ii) Arg/Lys residue at C-terminal. Peptides were isotopically labeled at C-term using Arg U-^13^C_6_; U-^15^N_4_ or Lys U-^13^C_6_; U-^15^N_2_. Prior to PRM or heavy-MRM analyses, heavy-peptides were mixed and subjected to the data-dependent acquisition (DDA) MS analysis with a Thermo Orbitrap Fusion Lumos mass spectrometer (Thermo, Shanghai).

PRM was then performed to validate the presence of target peptides in lung cancer and HCC samples as described (30). Briefly, synthetic heavy-peptides were first used as spiked-in standards and mixed with different tryptic digested cell lysates. The peptide mixture was next separated with a C18 column (75 μm internal diameter, 20 cm length, 1.9 μm particle size) prior to the injection into the mass spectrometer. Peptide precursors were isolated through a quadrupole at the window of 1.2Th. Fragment ions were generated in the HCD mode and detected in an Orbitrap at a resolution of 15K. PRM data were analyzed using the Skyline software, and endogenous peptide was considered to be verified when the following criteria met: (a) the transition generated from endogenous peptide (light) and synthesized standard (heavy) peptide shared the same elution profile on the liquid chromatograph; (b) at least three transitions from the same precursor were detected with S/N > 3 (31); (c) the deviation of light/heavy peptide calculated from each transition pair of the same precursor was less than 20%. The MS raw data for PRM are publicly available in iProX (accession number: IPX00076300)

### Heavy isotope-labeled peptide referenced MRM

For each target peptide awaiting for verification, we optimized the transitions, collision energy and retention time. In detail, each heavy-peptide was imported into the Skyline software to generate the transition list for 2+, 3+ and 4+ precursors, considering only the y-ions. By using the Skyline software, the collision energy was optimized by an integrated three-step method with the interval of ± 2 V starting from the predicted collision energy (CE). For each heavy-peptide, 200 fmol peptide was reconstituted with solvent A (0.1% FA), and sequentially loaded into the trap column (100 μm × 2 cm, nanoViper C18, 5 μm, 100 Å), and the nano column (50 μm × 15 cm, nanoViper C18, 2 μm, 100 Å) with an UltiMate 3000 RSLCnano System (Thermo Scientific) HPLC system. Peptides were then eluted with the gradient buffer (6%-30% solvent B (80% ACN, 0.1% FA)) for 30 min under a flow rate of 200 nl/min. The MRM acquisition was performed with the TSQ Quantiva system (Thermo Scientific) coupled with a Nanospray NG ion soure in positive ion mode. MS conditions were set as follows: ion spray voltage 2300 V; ion transfer tube temperature 320°C; CID gas 1.5 mTorr; cycle time 3 s; chrome filter of 3 s. The raw data were analyzed using the Skyline software. The 3–6 most intense transitions of each heavy-peptide and its corresponding 3–6 transitions of light peptide were exported as a single transition list, using the CE value that gave the highest intensity and the retention time. Post such optimization for each peptide, heavy-peptides and unlabeled peptides were mixed and analyzed with the same MRM method. To avoid retention time overlap, heavy-peptides were allocated into 18 groups based on their cycle times. The MS raw data for Heavy-MRM are publicly available in iProX (accession number: IPX00076300)

### Tissue samples

Human tissue samples were acquired from the First Affiliated Hospital of Jinan University. The scientific and ethics review committees of Jinan University approved this study, and written informed consents were obtained from all of the study participants.

### Plasmid constructs

To generate eGFP, mCherry and Flag fusion protein constructs with the *UBAP1-AST6* (UBAP1-AST6-eGFP, UBAP1-AST6-mCherry and KO-rescue plasmid), the *UBAP1-AST6* sequences were amplified using RT-PCR and cloned into a pEGFP-N1 (Clontech) vector, mCherry-N1 (Clontech) vector and pcDNA3.1(+) vector (Life Technologies), respectively. The following primers were used to generate the UBAP1-AST6-eGFP and UBAP1-AST6-mCherry plasmids: forward (5′-ATGGCTCACGGCAACCTTTG-3′) and reverse (5′-TTACTTTAGCTTCTGCTTCCGC-3′). For constructing the KO-rescue plasmid, the primers were: forward (5′-ATGGCTCACGGCAACCTTTG-3′), and the reverse (5′-TTACTTATCGTCGTCATCCTTGTAATCCTTTAGCTTCTGCTTCCGC-3′), which consisted of a Flag tag sequence. For the KO-rescue-ATG-mut plasmid construction, the start codon of the *UBAP1-AST6* gene was mutated to GCG using a Mut Express II Fast Mutagenesis Kit (Vazyme, Nanjing, Jiangsu, China).

### Lentivirus transductions

Lentiviral packaging used the PLVX-IRES-GFP, PMD2.G, PSPAX2 system (Clontech). First, *UBAP1-AST6* gene was cloned into PLVX-IRES-GFP plasmid, then PLVX-IRES-UBAP1-AST6-GFP, PMD2.G, PSPAX2 co-transfection into 293T cells following the manufacturer's instructions. After 48 h, culture supernatant was used to infect A549 cells, followed by purinomycin drug screening for the infected cells.

### CRISPR/Cas9 Knockout

UBAP1-AST6 knockout cell lines were generated by using the CRISPR/Cas9 system. The gRNAs targeting *UBAP1-AST6* gene were designed by the online tool developed by Prof. Zhang (http://crispr.mit.edu/). The DNA sequences expressing these gRNAs were cloned into pGK1.1 linear vector (Genloci Biotechnologies Inc. Nanjing, Jiangsu, China). The DNA sequences were: UBAP1-AST6-F: 5′-caccGCTTGTTTTTCAGGTTCTAAA; UBAP1-AST6-R: 5′-aaacTTTAGAACCTGAAAAACAAGC. A549 cells were transfected with pGK1.1-*UBAP1-AST6* by lipo.2000, expanded and examined for mutations at nuclease target sites by PCR amplification of genomic sequences, followed by DNA sequencing and immunoblotting. The complete deletion the *UBAP1-AST6* gene was confirmed by TA cloning of the PCR products.

### Cellular protein extraction

Cellular total proteins were extracted by using SDS Lysis Buffer (Beyotime, Jiangsu, China). To acquire subcellular protein fraction, cells were sequentially lysed by buffer A (10 mM Tris–HCl, 10 mM KCl, 5 mM MgCl_2_ and 0.6% Triton-100 (pH 7.6)) and buffer B ((10 mM Tris–HCl, 10 mM KCl, 5 mM MgCl_2_ and 0.35 M Sucrose (pH 7.6)). Lysates were then centrifuged at 600 × *g* for 10 min. Cytosolic proteins were largely remained in the supernatant, while the pellet was enriched with nucleic proteins.

### Western blotting

Protein extracts were separated by 10–20% Tricine Gels (Life Technologies) and subjected to immunoblotting analyses as we previously described (26). The primary antibodies against the new proteins were customized and raised by Tianjin Sungene Biotech Co., Ltd (Tianjin, China) using the specified short peptide sequence. As an exception, UBAP1-AST6 mAb was prepared by using the purified protein. Other primary Abs included rabbit anti-Lamin B1 polyAb (Protein Tech, Wuhan, Hubei, China), mouse anti-GAPDH mAb (Tianjin Sungene Biotech Co., Ltd). All secondary Abs were purchased from Tianjin Sungene Biotech Co., Ltd.

### Cell transfection and immunofluorescence analysis

Cells were cultured on coverslips with 70–80% confluence and were transfected by using Lipofectamin 2000 (Life Technologies). At 36 h post-transfection, cells were fixed with 4% paraformaldehyde in PBS (LEAGENE, Beijing, China) for 15 min, permeabilized with 0.1% Triton X-100 (GBCBIO Technologies Inc) for 5 min and blocked with 6% BSA (ZSGB-BIO, Beijing, China) in PBS, and incubated with Rhodamine Phalloidin (Cytoskeleton, Denver, CO, USA). To visualize different target molecules, slides were treated with mouse anti-β-actin mAb (Protein Tech), MitoTracker® Mitochondrion-Selective Probes (Life Technologies), Golgi-Tracker Red (Beyotime), ER-Tracker Red (Beyotime) or FITC Goat anti-mouse IgG (H+L) (Tianjin Sungene Biotech Co., Ltd). After being mounted with Prolong Gold Antifade Reagent with DAPI (Life Technologies), slides were observed with a Zeiss LSM710 confocal microscope (Zeiss, Oberkochen, Germany).

### qRT-PCR

Total RNA was extracted from cells using the Trizol total RNA isolation reagent (Life Technologies). RNA levels of *UBAP1-AST6* and *GAPDH* were detected using qRT-PCR. The *UBAP1-AST6* primers were: forward, 5′-GCTCAAGCCATCCTCCCAC’-3, reverse, 5′-GGACATCATCAAGGTAACTGAAAG’-3. The *GAPDH* primers were: forward, 5′-GAAGGTGAAGGTCGGAGTC’-3, reverse, 5′-GAAGATGGTGATGGGATTTC.

### Proliferation assay

We used the WST-1 assay (Beyotime) to examine the cell proliferation ability as we previously described (32). In brief, 2000 cells were seeded in 96-well plates with final volumes of 100 μl per well. Cells were treated with 10 μl WST-1 solution and continuously incubated for 2 h at 37°C. The absorbance was detected by a microplate reader (BioTek, Vermont, Winooski, VT, USA) using the dual wavelength method by OD_450_–OD_630_ (nm).

### Colony formation assay

Colony formation assay was performed as we previously described (33). Briefly, five hundred cells were plated in 6-well plates and cultured in DMEM medium supplemented with 10% FBS for 2 weeks. These cells were fixed with methanol and stained with 0.1% crystal violet. The number of colonies was then counted.

### Statistical analysis

Two-tailed Student's *t*-test or two-way ANOVA tests were performed using GraphPad PRISM software (GraphPad Software Inc., San Diego, CA, USA), for comparisons between two groups. For multiple comparisons, ANOVA with *post hoc* tests were used. Data were presented as mean ± sem. Statistical difference was accepted when *P* < 0.01.

## RESULTS

### Translating lncRNAs in human cell lines

**Figure 1.**
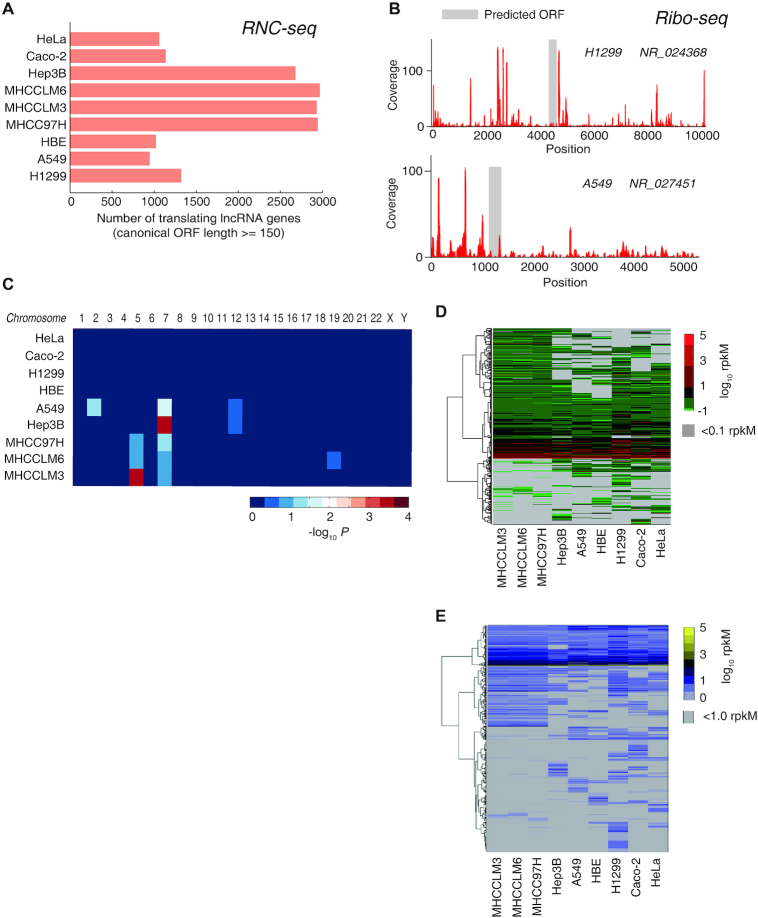
The translating lncRNAs that can encode proteins (canonical ORF length ≥150 nt). (**A**) The number of translating lncRNAs detected by the full-length translating mRNA sequencing (RNC-seq) in nine cell lines. (**B**) Ribosome footprints (RFP) of two translating lncRNAs as examples. Red bars denote the RFP coverage along the RNA, and the grey region marks the predicted canonical ORF. (**C**) The chromosome enrichment of the translating lncRNAs. *P*-values were calculated using Fisher Exact test. (D, E) Expression patterns of the translating lncRNAs in nine cell lines. The threshold of 0.1 (**D**) and 1.0 (**E**) RPKM are respectively used for positive detections.

We have previously sequenced the full-length translating mRNA in human lung HBE, A549 and H1299 cells (7), human colorectal cancer Caco-2 cells (21), human liver cancer MHCCLM3, MHCC97H and Hep3B cells (19) and human cervical cancer HeLa cells (20). Here, we further performed mRNA sequencing and the full-length translating mRNA-seq (RNC-seq) of MHCCLM6 cells. All together, we found that 1028∼3330 lncRNAs were bound to ribosomes in the nine tested human cell lines. Among them, 2969 translating lncRNAs possess at least one canonical open reading frame (ORF) that started with AUG and can encode proteins with at least 50 aa in length (Figure 1A and Supplementary Table S1). This implicates a remarkable hidden human proteome that has never been discovered before.

Our ribosome profiling results confirmed that these translating lncRNAs were actively translated by ribosomes, as the ribosome footprints spanned across the transcripts, especially across the predicted CDS regions (examples are shown in Figure 1B). The translating lncRNAs distributed across the entire human genome in all chromosomes (Supplementary Figure S1) with almost no chromosome enrichment detected (Figure 1C), indicating that these potential new coding genes were generally universal in the entire human genome. Meanwhile, approximately 1/3 of these translating lncRNAs were detected (≥0.1 RPKM and ≥10 reads) in almost all the 9 tested cell lines, and the rest showed remarkable cell-specific expression (Figure 1D), which was also observed when we used RPKM = 1.0 as a threshold (Figure 1E). These results suggested that the translation of these lncRNAs was universally regulated.

### Protein evidence for the lncRNA-encoded proteome

**Figure 2.**
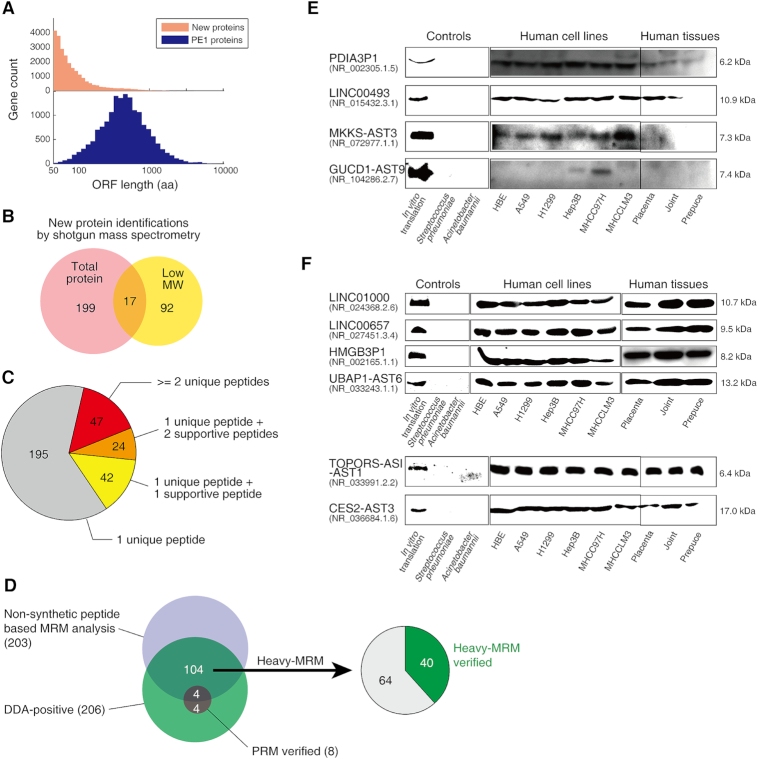
Protein evidence of the translating lncRNAs. (**A**) Length distribution of the new proteins and the PE1 proteins. (**B**) Identifications of new proteins by shotgun mass spectrometry analysis on total and low molecular weight proteins. (**C**) Bioinformatics based peptide evidence of the new proteins. (**D**) Data independent analysis verification by non-synthetic peptide based MRM, PRM and heavy synthetic peptide based MRM (heavy-MRM). (**E**) Immunoblotting verification of dour MS-detected new proteins in human cell lines and human tissues. (**F**) Western blot analysis of six MS-undetected new proteins.

Next, we aimed to find MS evidence for the potential new proteins encoded by these translating lncRNAs. We noted that the lengths of the new proteins were much shorter than the known proteins, i.e. the protein evidence level 1 (PE1) proteins proposed by the Human Proteome Project (HPP) (Figure 2A). Therefore, we performed MS for low-molecular weight proteins (<25 kDa) in supplement to the shotgun proteomics analysis on total proteins. We deemed confident identifications with the following criteria: (i) protein level FDR <1%, (ii) peptide length at least 9 aa, (iii) at least one unique peptide with no more than two possible aa mismatches allowed per Smith–Waterman analysis as we described previously (26). As such, we identified 372 unique peptides corresponding to 308 new proteins encoded by lncRNAs with MS analyses from total (216 proteins) and low molecular weight (109 proteins) protein fractions, respectively (Figure 2B), with only 17 overlapped. All the protein identification information of the 308 new proteins, including protein names, unique peptide sequences, peptide length, search engines can be found in Supplementary Table S2. The raw data files, reference databases, search result files are available through iProX database (accession number: IPX00076300). The file relationship and biological description can be found in the Supplementary Tables S3–S5. All MS-detected new proteins are listed in Supplementary Table S13 (in FASTA format).

We found that more than 36% MS-detected new proteins were evidenced by at least two unique peptides (47 proteins) or by one unique peptide plus at least one supportive peptide (66 proteins). Here, a supportive peptide is a non-unique peptide that is assigned by the database search engine to a certain protein identification. In addition, 261 of 308 MS-identified new proteins were evidenced by only one unique peptide (Figure 2C), which could be partially explained by their generally low expression levels (<10 RPKM) for most new proteins in the translating-mRNA analysis (Figure 1D and E), and the short lengths (Figure 2A). All detailed results of new protein identifications from MaxQuant, Mascot+Scaffold, and X!Tandam searches were listed in Supplementary Table S6.

We referenced the HPP Guideline 2.1 criteria (34) for presenting the extraordinary detection claims, which require to use data-independent acquisition MS methods, including multiple reaction monitoring (MRM) and parallel reaction monitoring (PRM). In general MRM/PRM are to select certain parent peptides for fragmentation and quantification. This can be assisted by addition of synthetic peptides serving as spike-in standards for further quantification of those peptides in samples. With regular MRM (without synthetic peptides), we found evidence for 203 unique peptides for 184 new proteins (Figure 2D), covering 54.6% and 59.7% of all identified unique peptides and new proteins, respectively. All information regarding MRM spectra, Skyline data file names, verified parent ion m/z values and charges, are summarized in Supplementary Figure S2 and Table S7.

For synthetic peptide-based PRM assay, among the 372 new protein coding peptides, we synthesized 313 heavy-labeled standard peptides that were technically allowed. We could identify 206 out of the 313 standards with data-dependent acquisition (DDA) analysis (Figure 2D). With PRM analysis on these 206 peptides, we could only verify the existence of 8 peptides (Figure 2D, Supplementary Figure S3 and Table S10), suggesting that optimized MRM was required. There were 104 heavy peptides that were detected in both DDA and regular MRM (Figure 2D). From there, with one-by-one optimization on the heavy-MRM analysis, we could further verify 40 peptides from 38 proteins existed in the cell lysates (Figure 2D, Supplementary Figure S4 and Table S11). The relative intensities and the elution time of the peak group of these peptides all matched to the synthetic reference peptides, suggesting confident verifications of their existence per HPP Guideline 2.1 criteria (34).

We next prepared polyclonal antibodies for four new proteins detected by shotgun MS using their specific peptides which contained antigen epitopes. Immunoblotting results showed clear and specific bands in various human cell lines and human tissues for all these proteins at their expected molecular weights (Figure 2E), suggesting that they exist in full-length and stable forms *in vivo*. To further ensure the specificity of the antibodies, we used the *in vitro* translated full-length proteins as positive controls, and total protein extracted from the bacteria *Acinetobacter baumannii* and *Streptococcus pneumoniae* as negative controls (Figure 2E). In addition, we prepared polyclonal antibodies for another 6 new proteins that were found in the translating mRNA analysis but not detected by MS, and obtained their confident immunoblotting evidence (Figure 2F). All raw images of the immunoblotting analyses are included in Supplementary Figure S5. These results indicate that the MS can only detect a fraction of the new proteins due to its limitations, and implicate that the size of the hidden proteome should be larger.

In sum, we validated the existence of the hidden human proteome encoded by the translating lncRNAs using translation, MS and antibody evidences.

### Properties of the new proteins

**Figure 3.**
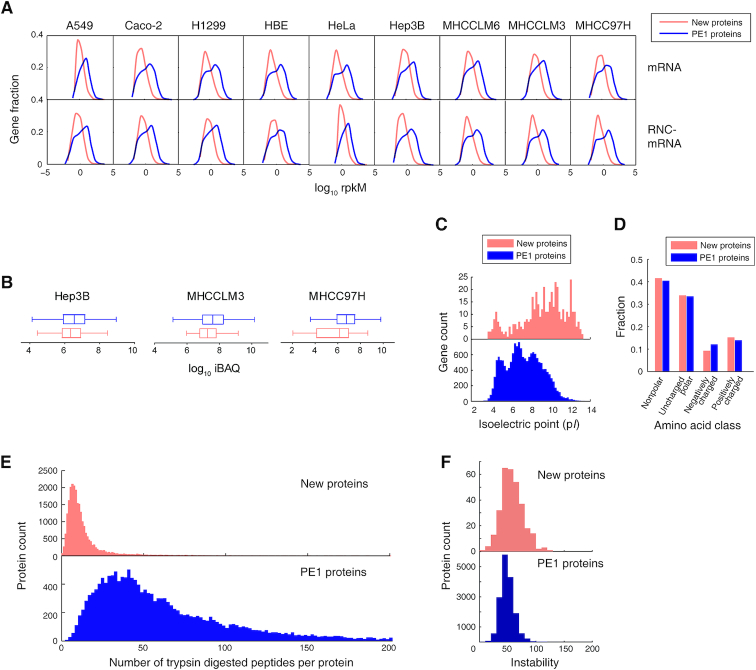
The difficulty of detecting the new proteins. (**A**) Expression level of the new proteins and PE1 proteins in transcription level (mRNA) and translation level (full-length translating mRNA). (**B**) Protein abundance of PE1 proteins and MS-detected new proteins in three hepatocellular carcinoma cell lines. (**C**) The distribution of isoelectric point (p*I*) of MS-detected new proteins and PE1 proteins. (**D**) The amino acid properties of MS-detected new proteins and PE1 proteins. (**E**) Number of trypsin digested peptides per protein of MS-detected new proteins and PE1 proteins. (**F**) The calculated instability of MS-detected new proteins and PE1 proteins.

These new proteins deviate from the canonical PE1 proteins with various properties. First, the transcription and translation levels of these lncRNAs were generally lower than the genes encoding PE1 proteins (Figure 3A). Since their abundances were mostly <1 RPKM in mRNA as well as at the translation level, they were often considered as expression or experimental noises. Using our highly accurate and experimental validated mapping algorithm (22), we could determine their existence at transcription and translation levels. The new proteins were also less abundant in protein level than the PE1 proteins (Figure 3B), which challenged the sensitivity of MS-matching methods. Second, the isoelectric point (p*I*) of the new proteins are significantly higher than the PE1 proteins (*P* < 10^−16^, KS-test, Figure 3C), which explains the difficulty for MS to detect them (26,35). This difference tended to be caused by their amino acid compositions (Figure 3D). The new proteins used a similar amount of uncharged amino acids as PE1 proteins, while they tended to use more positively charged amino acids and less negatively charged amino acids (Figure 3D). Third, the shorter length of the new proteins led to significantly less possibility of tryptic digestion (*P* < 10^−16^, *KS*-test, Figure 3E), thus decreasing the possibility of finding more unique peptides and supportive peptides. Fourth, the new proteins were predicted to be less stable *in vivo* than the PE1 proteins, as calculated by the instability index (36) (*P* < 10^−16^, *KS*-test, Figure 3F), indicating less possibility to find such proteins in intact form in the cells. These properties make the new proteins less probable to be detected.

### Origin of the new proteins

**Figure 4.**
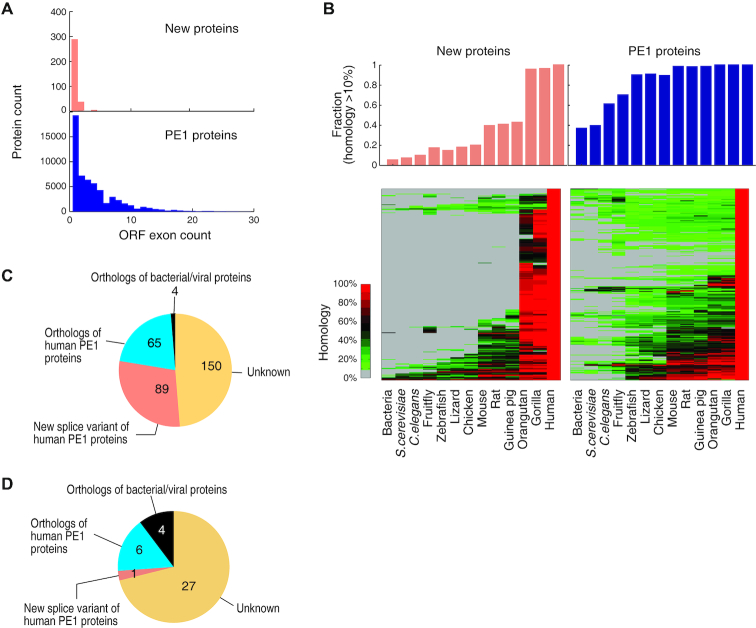
Origin of the new proteins. (**A**) ORF exon counts of MS-detected new proteins and PE1 proteins. (**B**) The homology of the 308 MS-detected new proteins and 308 randomly selected PE1 proteins across the phylogeny. Upper panels: the number of homologous genes found in the species with at least 10% homology. Lower panels: the distribution of the homologous genes across the species. The homology is color-scaled. (**C**) Orthology of the 308 MS-detected new proteins. (**D**) Orthology of the 38 MS confirmed new proteins shown in Figure 2D.

Another interesting question is why these coding genes have been erroneously classified as non-protein-coding genes before. Previous studies used computational approaches to predict genes as protein-coding or non-coding, based on properties of gene and exon structures, potential CDS features (codon bias, *etc*), polyA sites and homology across genomes (reviewed in (37,38)). We found that these new coding genes contained significantly less number of exons than the PE1 proteins (Figure 4A). 88% of the new coding genes contain single-exons. The sequence properties of the new proteins significantly deviated from known proteins (Figures 2A and 3C-E), which might have confused the prediction algorithms based on supervised classification and machine-learning. Poly(A) sites were predicted by aligning EST or RNA-seq reads to genome sequences (reviewed in (37)) and thus the method is unable to distinguish protein-coding mRNAs and long non-coding RNAs with polyA tails.

An important criterion of the coding propensity predicting algorithms lies in the evolutionary conservatism of proteins across the species. Therefore, we performed homology search of these new proteins against various genomes ranging from all genome-sequenced bacteria to primates, in comparison to PE1 proteins. As a result, we could detect homologous genes in all vertebrates for more than 88% of PE1 proteins, with nearly 40% PE1 genes having their ancestors in bacteria. In sharp contrast, more than half of the new proteins emerged in primates and did not exist even in rodents, and only <6% could be found in bacteria (Figure 4B). This implicates that the new proteins are mostly very young genes during evolution, which partially explains why the predicting algorithms have considered these genes as non-coding. Interestingly, most of the new proteins in orangutan and gorilla show very high homology (>60%) to the human counterparts, while only <30% of the PE1 proteins show such high homology to their human counterparts. This suggests that the new proteins are well conserved since their emergence in primates.

We then searched for the evolutionary ancestors for the new proteins within human genome. We found that 28.9% of the new proteins were alternative splice variants of the known protein-coding genes, 21.1% were homologous copy of known protein-coding genes with genetic alterations (Figure 4C). A small fraction of 1.3% was homologous to bacteria and virus, which might indicate a genetic horizontal transfer during symbiosis or infection (Figure 4C). 48.7% could not be explained by any of above origins (Figure 4C). Further comparison with the 38 new proteins (Figure 2D) that fully complied with the HPP Guideline 2.1, we found that 27 (71%) of these proteins were also from unknown origin (Figure 4D).

### Functional potency of new proteins

**Figure 5.**
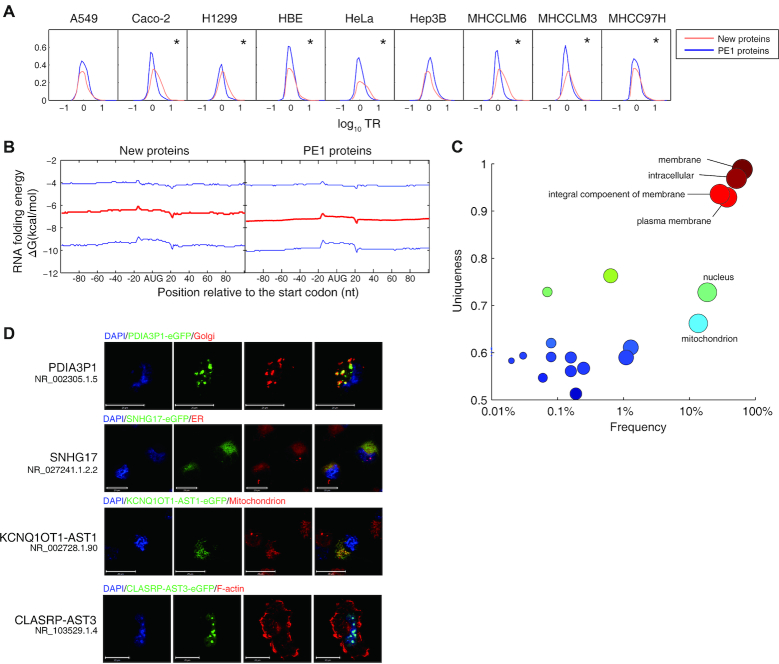
Translation efficiency and subcellular localization of the new proteins. (**A**) Distribution of the translation ratio (TR) of the new proteins and PE1 proteins in 9 cell lines, respectively. * denotes the cell lines in which the new proteins have significantly higher TR than PE1 proteins (*P*< 10–16, Kolmogorov–Smirnov test). (**B**) RNA secondary structure stability near the AUG codons of the MS-detected new proteins and PE1 proteins, calculated with a sliding window of ±19 nt. Red lines show the average Δ*G*, and blue lines denote the upper and lower bounds of the Δ*G* of such category. (**C**) The enrichment of subcellular localizations of the MS-detected new proteins, predicted by BLAST2GO. (**D**) Confocal fluorescent microscopy observation of the subcellular localization of four new proteins. Please refer to Supplementary Figure S6 for more examples.

To further investigate whether these new proteins were potentially functional, we calculated the function-relevant translation ratio (TR) values, which we had previously defined as the translating mRNA *versus* total mRNA ratio of a certain gene (7). TR mainly reflects the translation initiation, and it is known that high TR genes determine the cellular phenotypes (7,20,39). Here, we found that the average TR of the translating lncRNAs was significantly higher than that of known coding genes in seven out of nine tested cell lines, except A549 and Hep3B (Figure 5A). This indicates a more active translation initiation of the new proteins. It is known that more stable RNA secondary structure near the start codon decreases the translation initiation efficiency (24). Our analyses showed that the new proteins have less stable RNA structure near the start codon (Figure 5B), which partially explains the more active translation initiation of the new proteins. As the new proteins exist in a stable and full-length form *in vivo* (Figure 2E and F), they are unlikely to be merely products of erroneous ribosome binding.

Most proteins need to be localized properly to be functional. Therefore, we next predicted the subcellular localization of the new proteins. The Cellular Component terms of gene ontology were exported from BLAST2GO search. REVIGO enrichment analysis (40) showed clear enrichment of the new proteins localized in various types of subcellular structures, including membrane systems, nucleus and mitochondrion (Figure 5C). We then employed confocal fluorescent microscopy to verify these predicted localizations by constructing these genes into pEGFP-N1 plasmids transfected into H1299 cells. As such we successfully validated all 20 new proteins with predicted subcellular localizations (3 mitochondrion, 1 endoreticulum, 1 Golgi apparatus, 2 nucleus and 13 cytosol proteins). Representative images are shown in Figure 5D and Supplementary Figure S6. Our results suggest that the new proteins are highly likely to be functional *in vivo*.

### Cellular phenotypes regulated by new protein UBAP1-AST6

**Figure 6.**
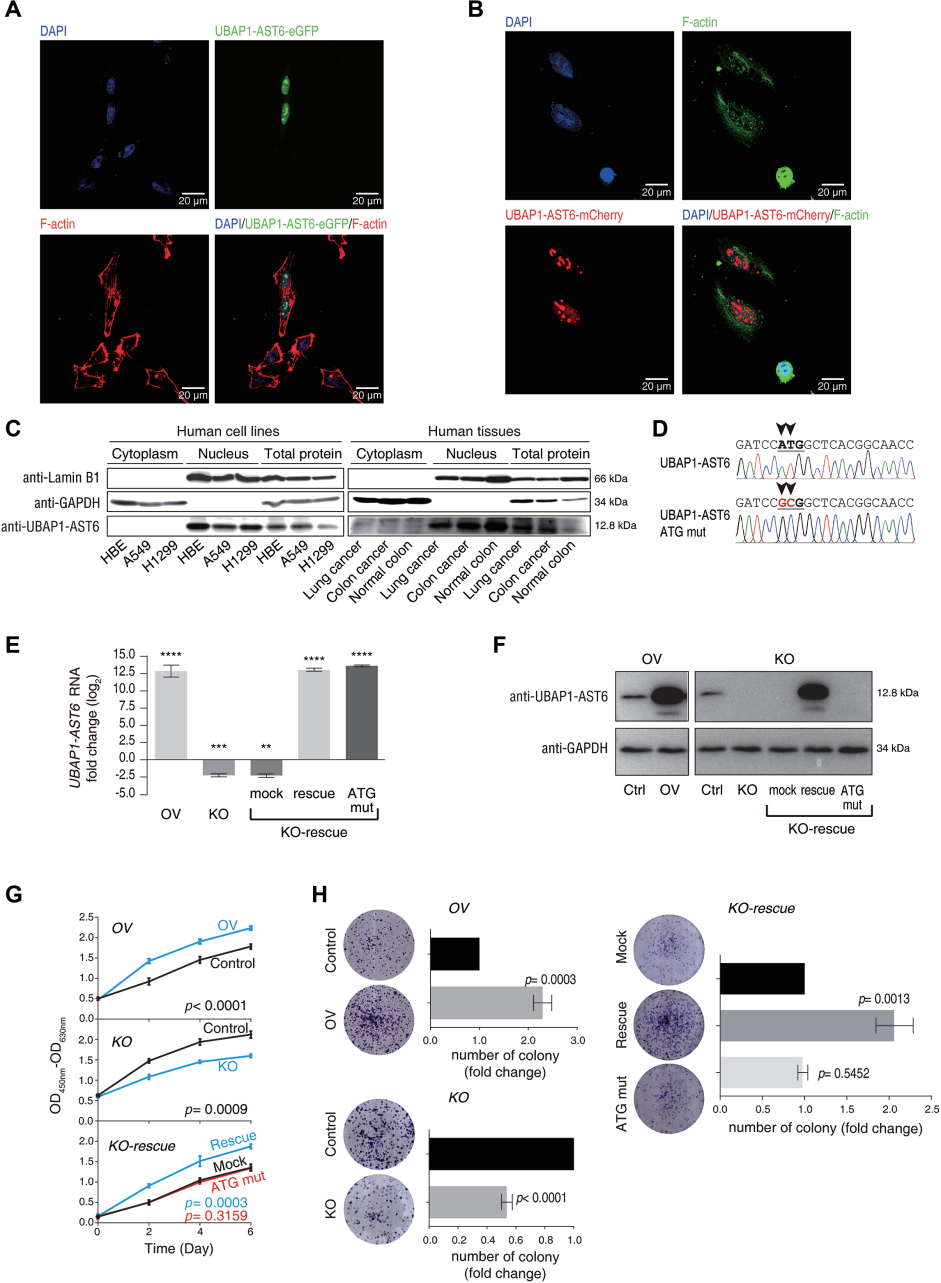
The potential biological function of a new protein UBAP1-AST6. (**A**) Subcellular localization of UBAP1-AST6, fused by EGFP. (**B**) Same as (A), UBAP1-AST6 is fused with mCherry. (**C**) Western blot verification of the nucleus localization of UBAP1-AST6 using three cell lines and three human tissues. (**D**) The DNA sequence UBAP1-AST6-ATG mut plasmid. The start codon ATG of the *UBAP1-AST6* ORF was mutated to GCG to abolish translation initiation. The sequences were verified by Sanger sequencing. (**E**) qRT-PCR analysis of relative *UBAP1-AST6* RNA expression in over-expression (OV) and knock-out (KO) models. A549 cells were infected with LentiViral-flag (OV-Control) or LentiViral-UBAP1-AST6-flag (OV-UBAP1-AST6), followed by qPCR analysis of *UBAP1-AST6* relative to *GAPDH*. Similar analysis was also performed on CRISPR/Cas9 KO and pcDNA3.1-UBAP1-AST6 rescue (KO-rescue) groups. In the rescue models, we included an ATG-mutated pcDNA3.1-UBAP1-AST6 group (KO-rescue-ATG-mut) as a control. (**F**) Immunoblotting validation of UBAP1-AST6 expression. (**G**) Proliferation assays using WST-1. *n* = 3. (**H**) Colony formation assay. *n* = 3.

We next tried to provide direct evidence on the functionality of one new protein UBAP1-AST6. We targeted UBAP1-AST6 because of its interesting nucleoli localization and its high TR in the lung cancer A549 cells. UBAP1-AST6 strictly localized in nucleus using EGFP and mCherry fusion proteins, respectively; our results excluded the influence of the fluorescent tags (Figure 6A and B). We then raised the antibody specifically against the UBAP1-AST6 and found that it also localized in the nucleus in various human cell lines and human tissues (Figure 6C). To show the function of UBAP1-AST6, we built overexpression (OV) and knock-out (KO)/rescue models. To answer whether the function elicited by proteins or RNAs, in the rescue experiments, we included a control using ATG-mutated pcDNA3.1-UBAP1-AST6 plasmid to transfect A549 cells (KO-rescue-ATG-mut) to abolish the translation initiation. The qRT-PCR results showed that all OV or KO groups worked as expected (Figure 6D), and the protein and RNA expression levels matched per immunoblotting assays (Figure 6E). In particular, the UBAP1-AST6 expression was successfully rescued in the KO-rescue group at both mRNA (Figure 6D) and protein (Figure 6E) levels, while in the KO-rescue-ATG-mut group, only *UBAP1-AST6* RNA could be recovered (Figure 6D), but not the protein (Figure 6E).

We next used these cellular models to test the UBAP1-AST6 functions. We found that overexpression of UBAP1-AST6 could significantly promote the cell proliferation (Figure 6F) and clone formation (Figure 6G). In contrast, in the KO groups, cells showed significantly decreased proliferation (Figure 6F) and clone formation (Figure 6G). Such a decease could be significantly recovered by rescuing UBAP1-AST6 protein as indicated in the KO-rescue groups (Figure 6F and G). However, in the KO-rescue-ATG-mut group, no rescuing effect was observed in both proliferation (Figure 6F) and clone formation (Figure 6G) assays. Thus, we evidenced that UBAP1-AST6 is functional in its protein form as a possible tumor promoter, but not in its *UBAP1-AST6* RNA form.

## DISCUSSION

Increasing evidence has shown that some lncRNAs encode proteins bearing biological functions (11,12). This strengthens a debating question that the human genome may contain more coding genes than we usually believe. These missing coding genes may somehow be systematically omitted by the conventional annotation algorisms in error. If so, finding these missing coding genes may represent a discovery of a biological treasury never being known. This finding may also re-define the conventional annotation of human genome and fundamentally update the basic biology of human coding genes.

With our established full-length translating mRNA (RNC-seq) analysis, our systematical screening revealed that at least 1300 lncRNAs bound to ribosome may be coding genes (7,41). Based on the experimental evidence acquired from translation, mass spectrometry, antibody and bioinformatics, we here demonstrated the actual existence of a hidden human proteome containing 308 proteins encoded by these lncRNAs. Among them 5 lncRNAs have been found to encode microproteins by other groups, including *SNHG17, PAX8-AS1, OTUD6B-AS1, MBNL1-AS1* and *MAPKAPK5-AS1* (18).

We could now summarize the reason why previous studies had missed the identification of these new protein-coding genes as follows. First, the physical-chemical features of these gene products are not suitable for mass spectrometry. Specifically, they are shorter in amino acid length and more basic, which are typical features for those known coding genes where there is a lack of mass spectrometry evidence (1,26). Second, the three-frame translation largely expanded the reference protein database, resulting in an elevated threshold to control FDR, thus reducing the sensitivity of peptide identifications (42). In this study, the use of cell-type specific translating mRNAs to serve as ORF prediction and reference database for MS data search ensured the accurate matches. Third, the new coding genes are generally not conserved. More than half of these genes emerged in primates, representing very young genes in evolution. Young genes were considered to be connected to the primate-specific phenotypes (41,43). Therefore, this hidden proteome can serve as a rich source especially for human biology and disease research.

Equally important is that we have experimentally shown that these new proteins are biologically functional, such as those organelle-specific localized proteins. As an example, we showed that UBAP1-AST6 is a protein coding gene and its protein product functions in cell proliferation. In eukaryotic cells, the initiation efficiency has been deemed the rate-limiting step of translation (44); we have further proved the tight relevance of translation initiation with phenotype (7). In this study, we found that TRs of new protein coding genes are statistically higher than those of known protein coding genes. More importantly, we demonstrated that these new proteins as validated by Western blotting are all stable in their full-length form in human cell lines and human tissues, implicating that this hidden proteome is not a noise and of biological significance. Such a conclusion is partially favored by the studies from other groups, showing that the properties of the coding RNAs and ncRNAs overlap ((14) and reviewed in (45)).

lncRNAs have been frequently connected to cancer progression, cardiovascular and neurodegenerative diseases and many other diseases (46,47). Their functioning modes include competitive binding of mRNAs and interaction with miRNAs. Here, our experimental validation of the hidden human proteome encoded by the ‘non-coding’ RNAs will move the field forward by distinguishing a remarkable proportion of the lncRNAs that function with their encoded proteins. This will fundamentally improve our understanding on disease models. In addition, we believe that our current finding only opens a small gate of finding new proteins from putative ncRNAs. It will be interesting to expand discoveries in the field and promote both the biology- and disease-driven studies with the recognition of such unique proteomes.

## DATA AVAILABILITY

All MS-detected new proteins are listed in Supplementary Table S13 (in FASTA format).

The mRNA, RNC-mRNA and ribosome profiling sequencing datasets was deposited in Gene Expression Omnibus database, accession numbers: GSE42006, GSE46613, GSE48603, GSE49994, GSE79539 and GSE79539. The MS raw data are publicly available in iProX (accession number: IPX00076300).

## Supplementary Material

gkz646_Supplemental_Files
