# Supplementary material for: A hidden human proteome encoded by ‘non-coding’ genes
Source: Nucleic Acids Res. 2019 Jul 24;47(15):8111–25. doi: 10.1093/nar/gkz646 (PMC6735797; doi:10.1093/nar/gkz646)
Supplement: gkz646_Supplemental_Files [file gkz646_supplemental_files.zip › Supplementary Fig S2.pdf]

NR_001446.2.1
PEDGSVVDYELIDQDAR

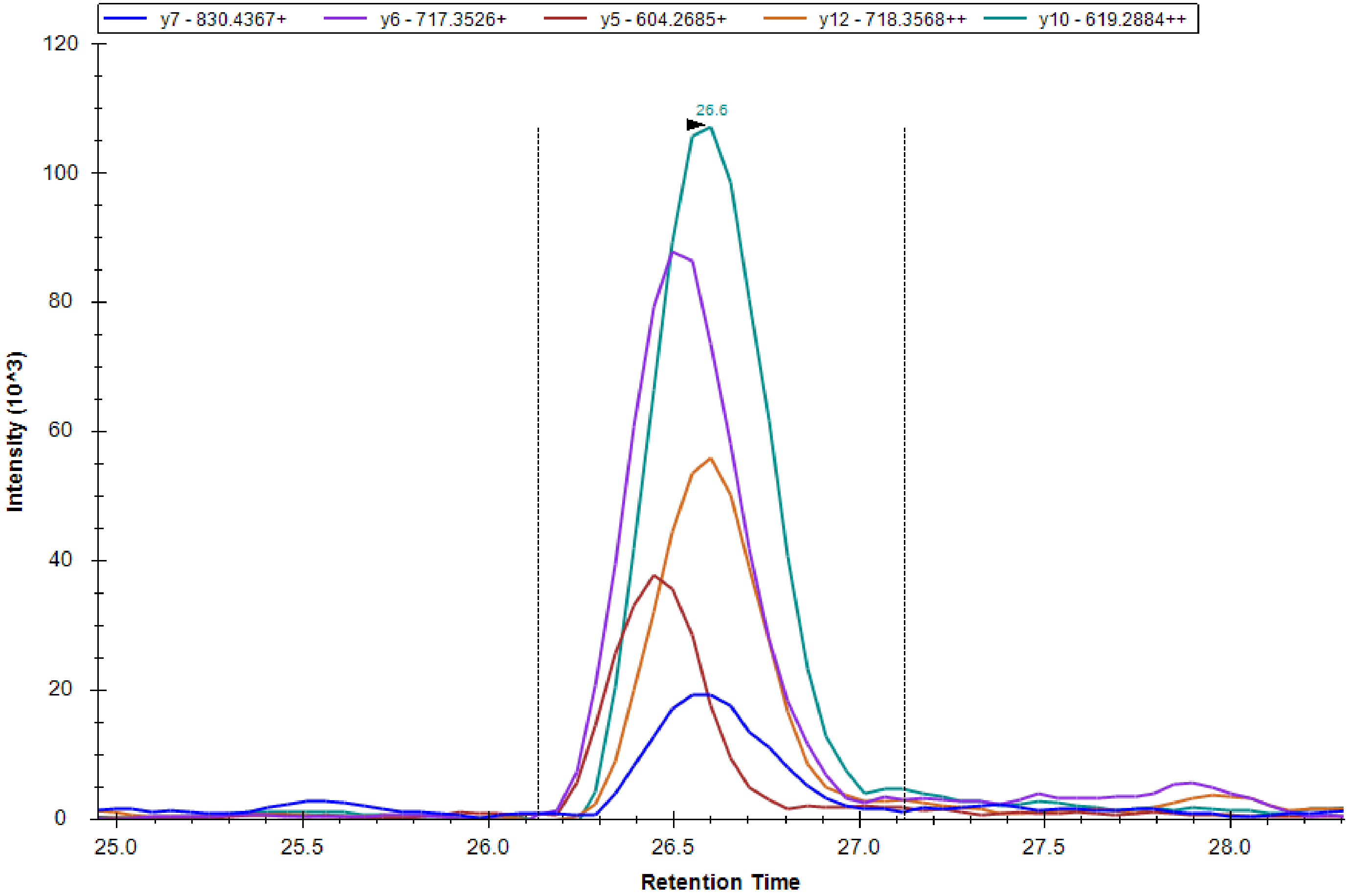

File name: 1604_JinanU(GZ)_MRM_#A_Full-Screen_0421.skyd

Parent ion m/z and charges: 640.9656+++

NR_001446.2.1
FCKLMFALANVRRPEDGSVVDYELIDQDAR

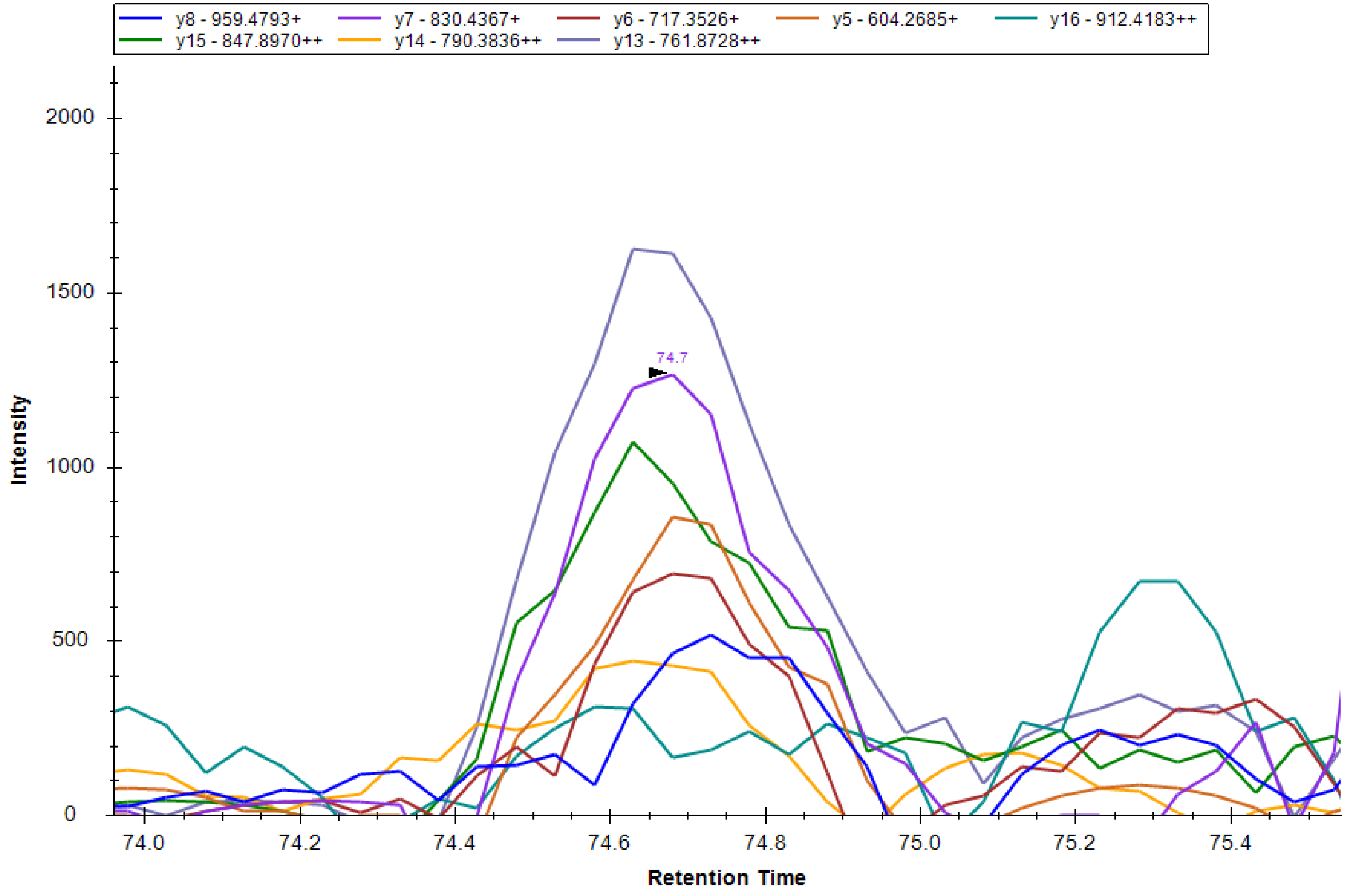

File name: 1604_JinanU(GZ)_MRM_#A_Full-Screen_0421.skyd

Parent ion m/z and charges: 1176.5852+++

NR_001446.2.1
PEDGSVVDYELIDQDAR

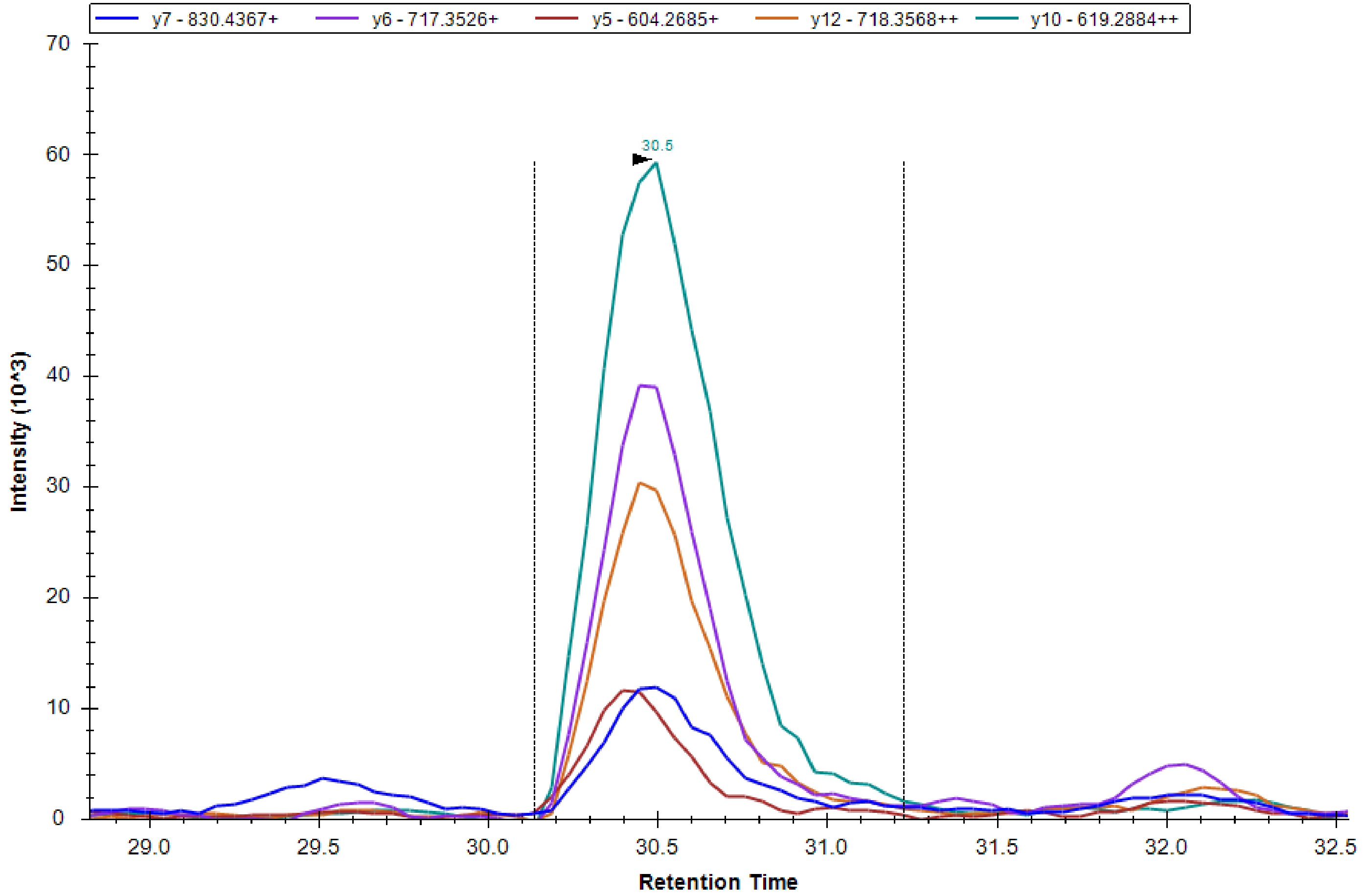

File name: 1604_JinanU(GZ)_MRM_#B_Full-Screen_0421.skyd

Parent ion m/z and charges: 640.9656+++

NR_001446.2.1
PEDGSVVDYELIDQDAR

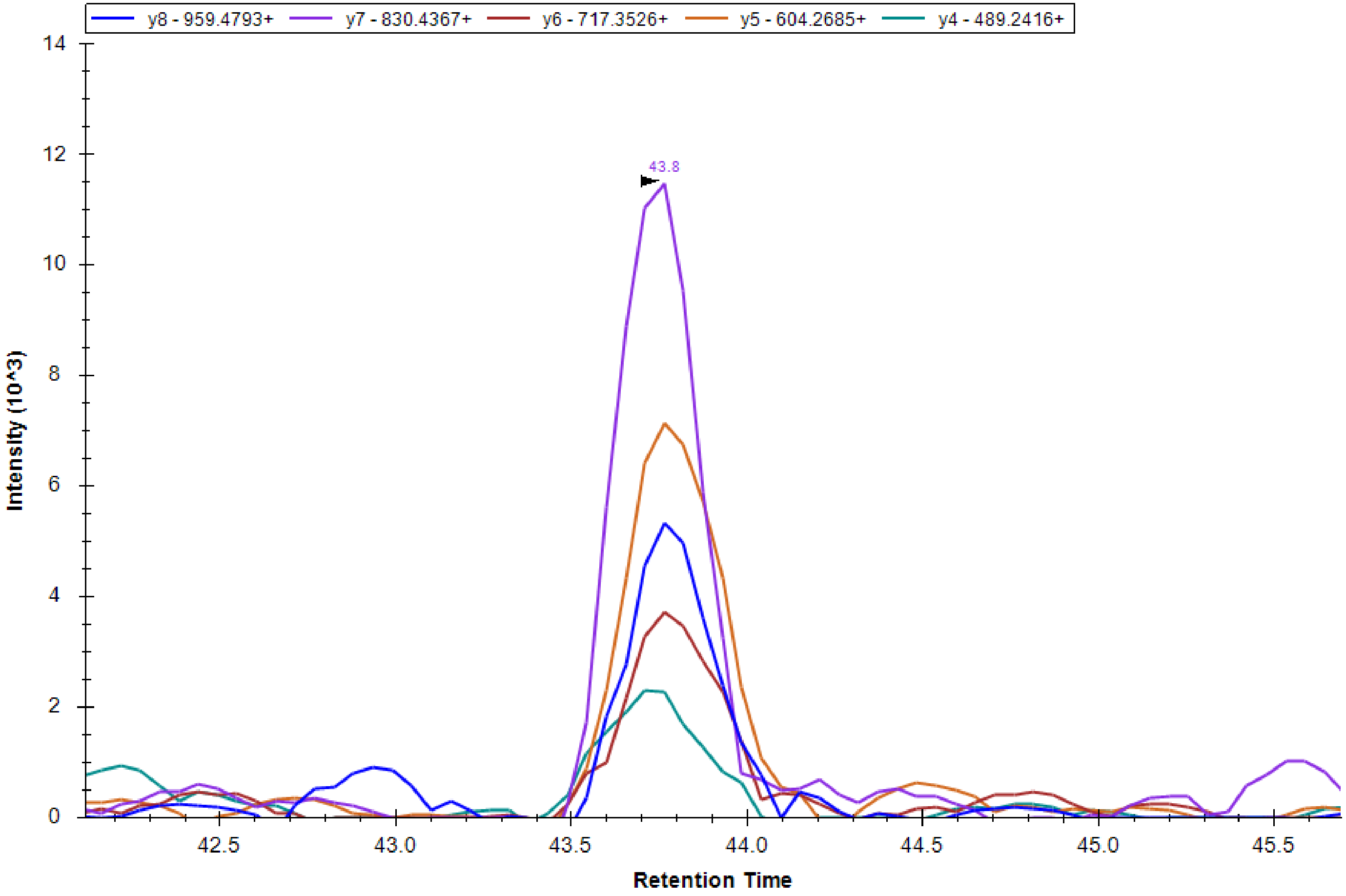

File name: 1604_JinanU(GZ)_MRM_#B_2nd_Round_Screen.skyd

Parent ion m/z and charges: 960.9447++

NR_001446.2.1
PEDGSVVDYELIDQDAR

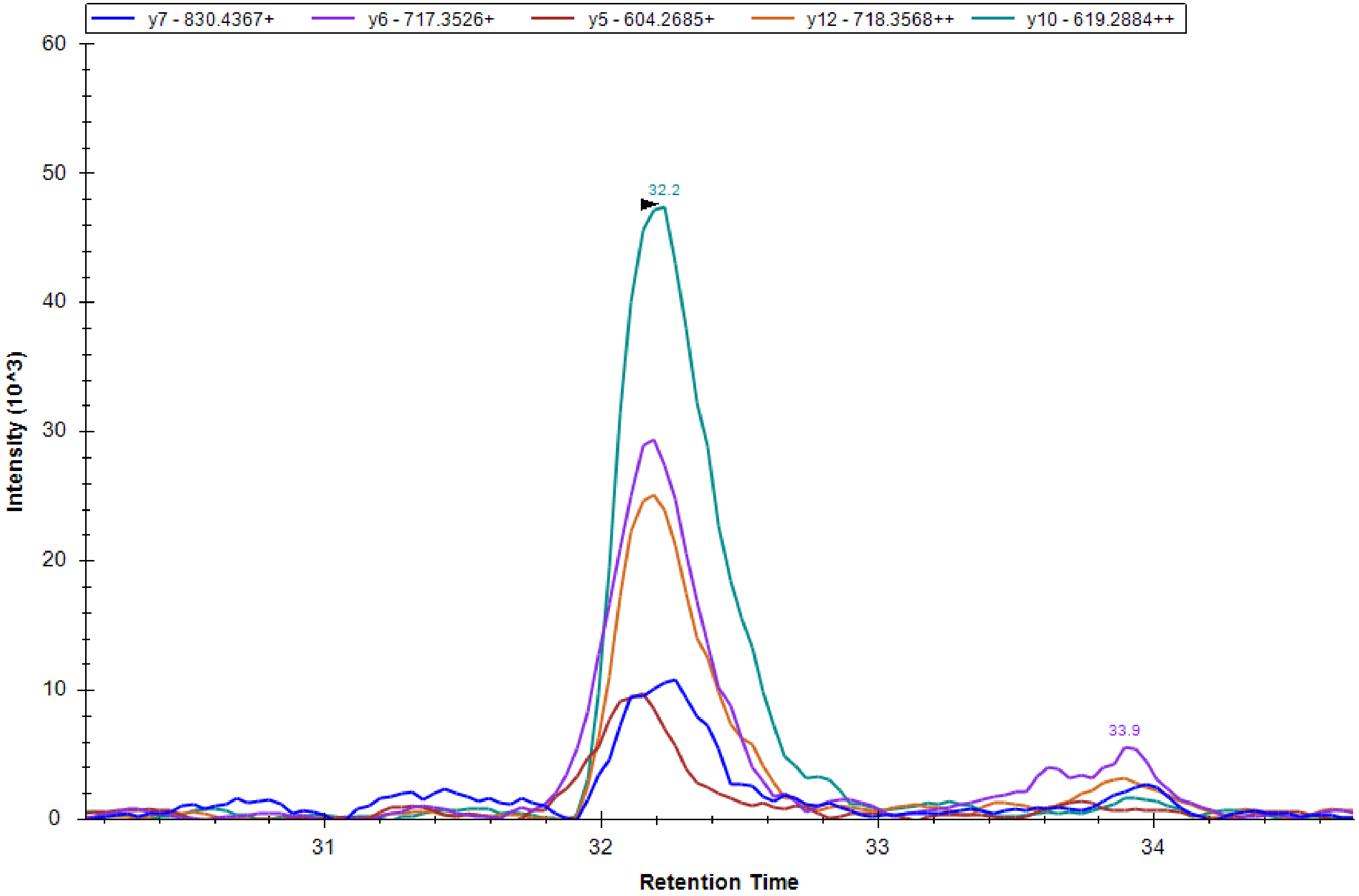

File name: 1604_JinanU(GZ)_MRM_#A_#B_Positive_Screen.skyd

Parent ion m/z and charges: 640.9656+++

NR_001562.3.3
LGTNKDSLIEII**C**SR

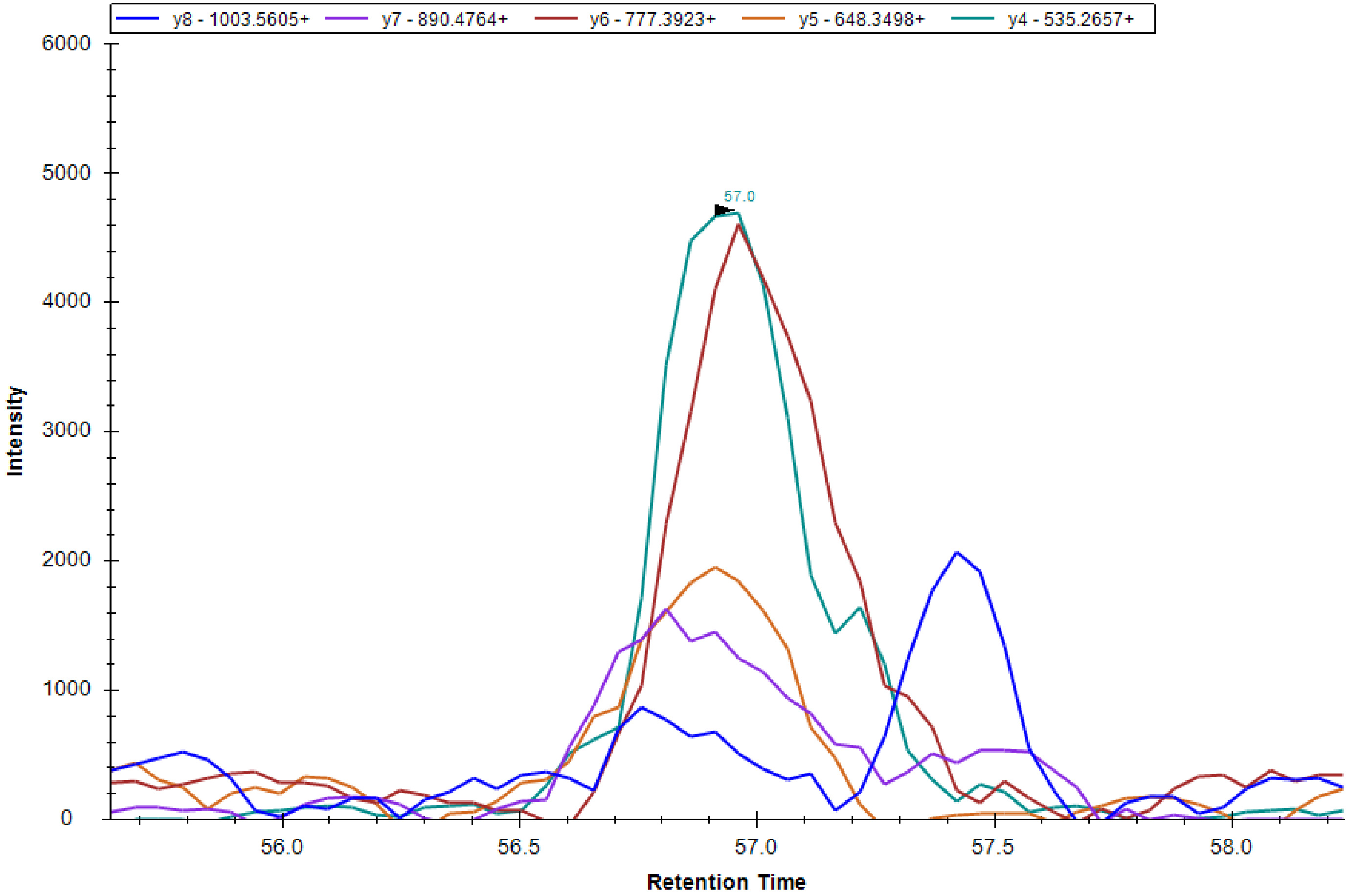

File name: 1604_JinanU(GZ)_MRM_#A_Full-Screen_0421.skyd

Parent ion m/z and charges: 859.9589++

NR_001562.3.3
GLGTNKDSLIEIICSR

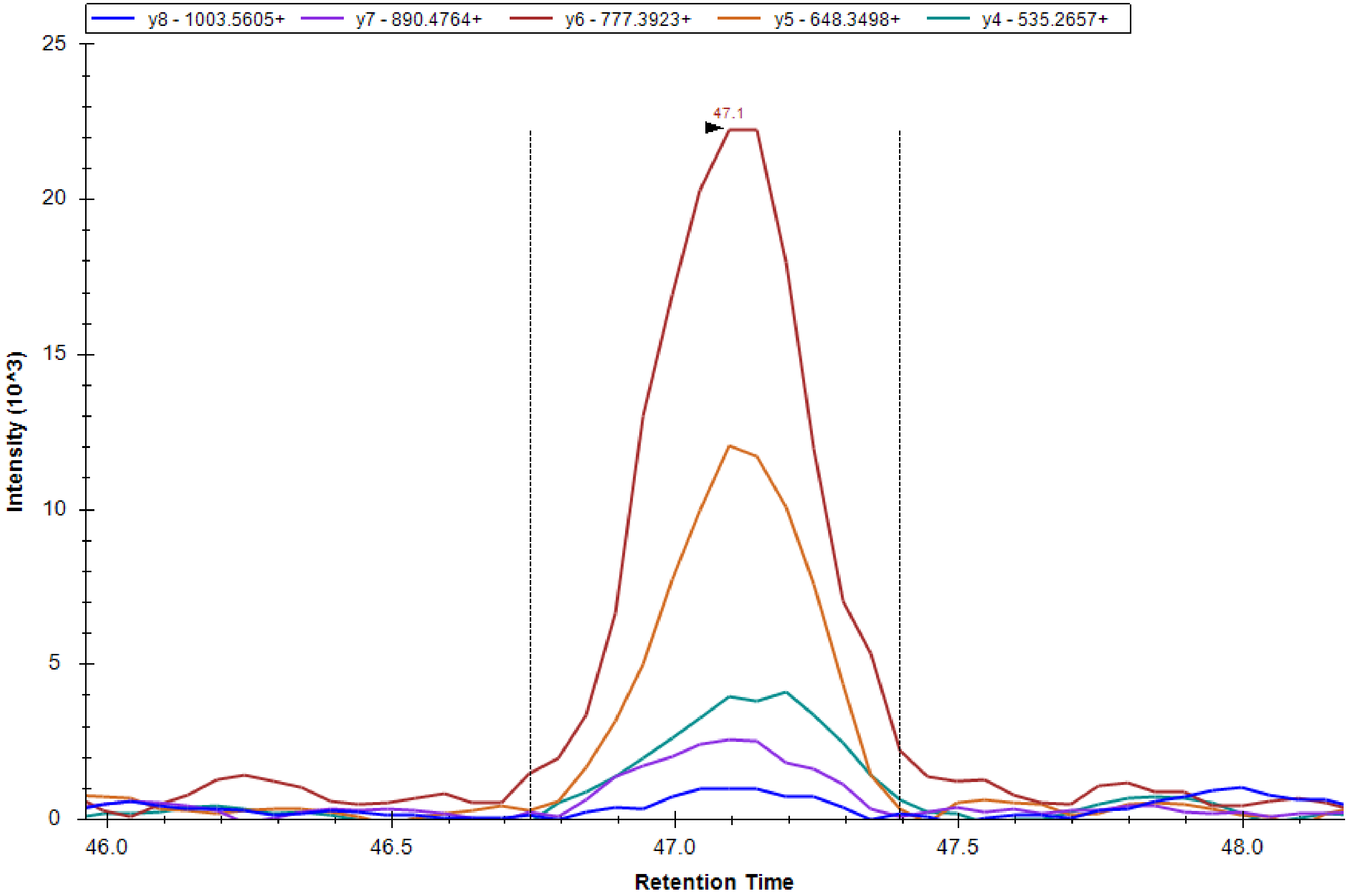

File name: 1604_JinanU(GZ)_MRM_#A_Full-Screen_0421.skyd

Parent ion m/z and charges: 888.4696++

NR_001562.3.3
LGTNKDSLIEIICSR

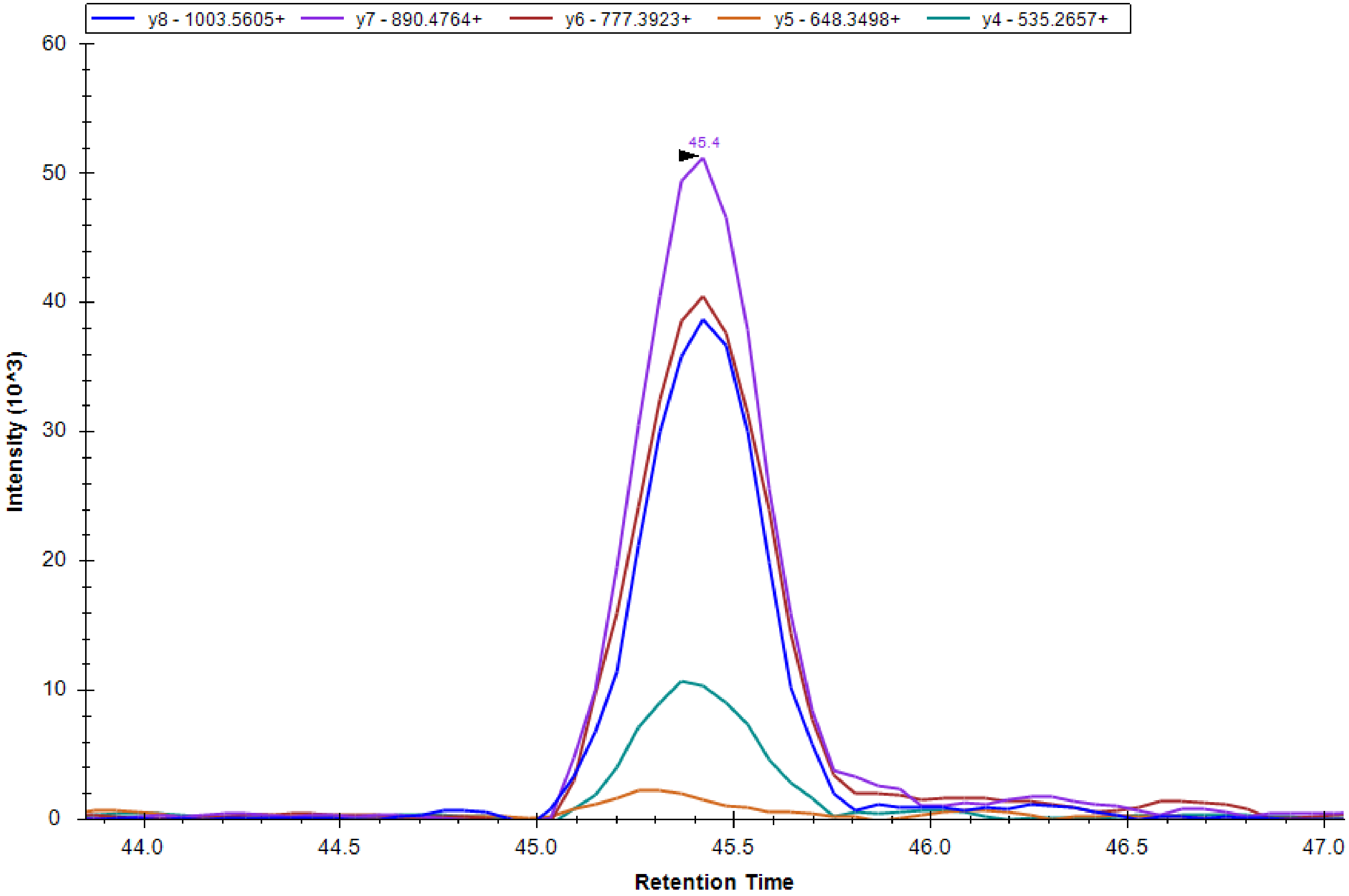

File name: 1604_JinanU(GZ)_MRM_#B_2nd_Round_Screen.skyd

Parent ion m/z and charges: 859.9589++

NR_001562.3.3
GLGTNKDSLIEIICSR

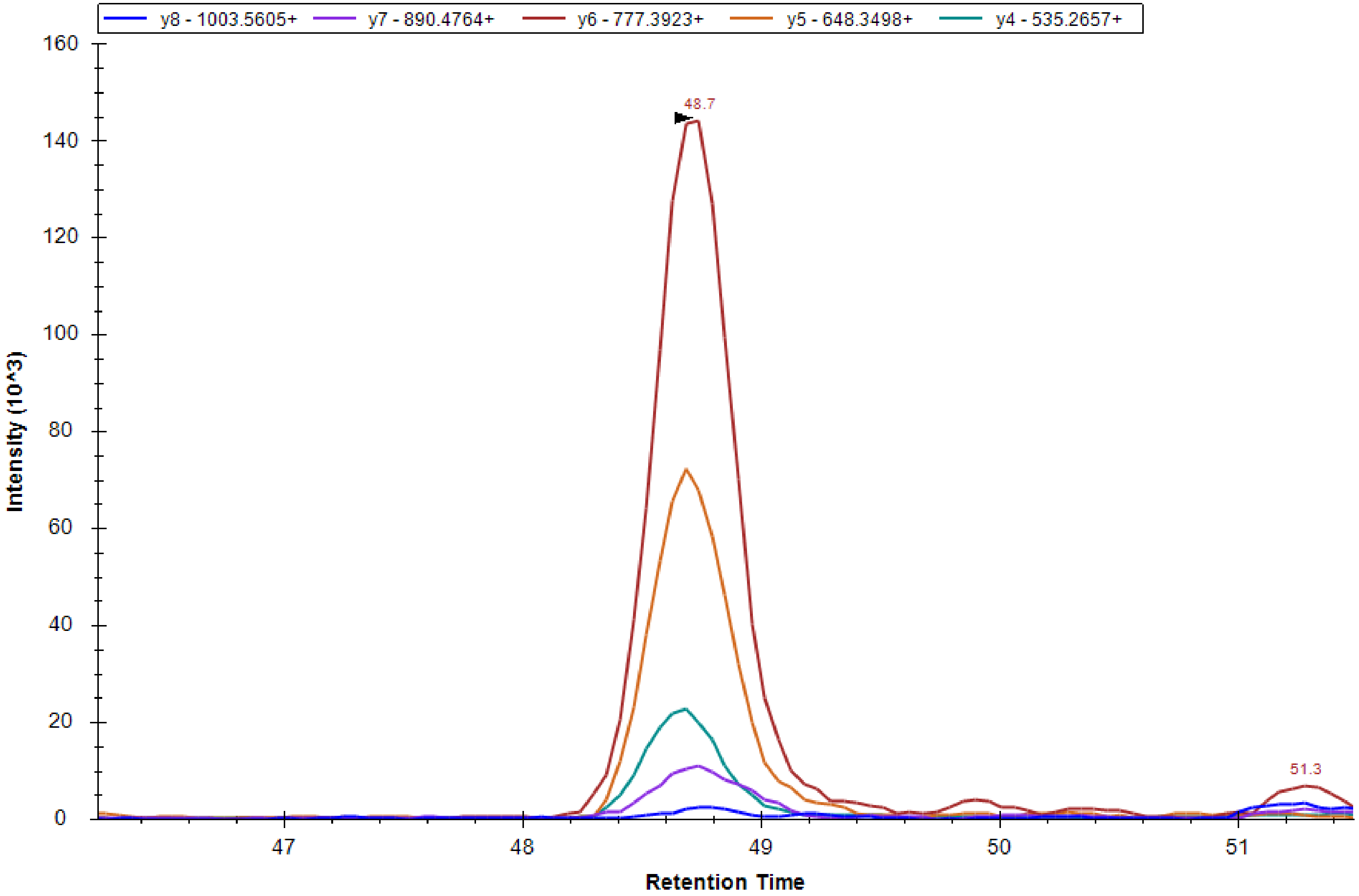

File name: 1604_JinanU(GZ)_MRM_#B_2nd_Round_Screen.skyd

Parent ion m/z and charges: 888.4696++

NR_002186.2.7
KVPDSAYEMIGR

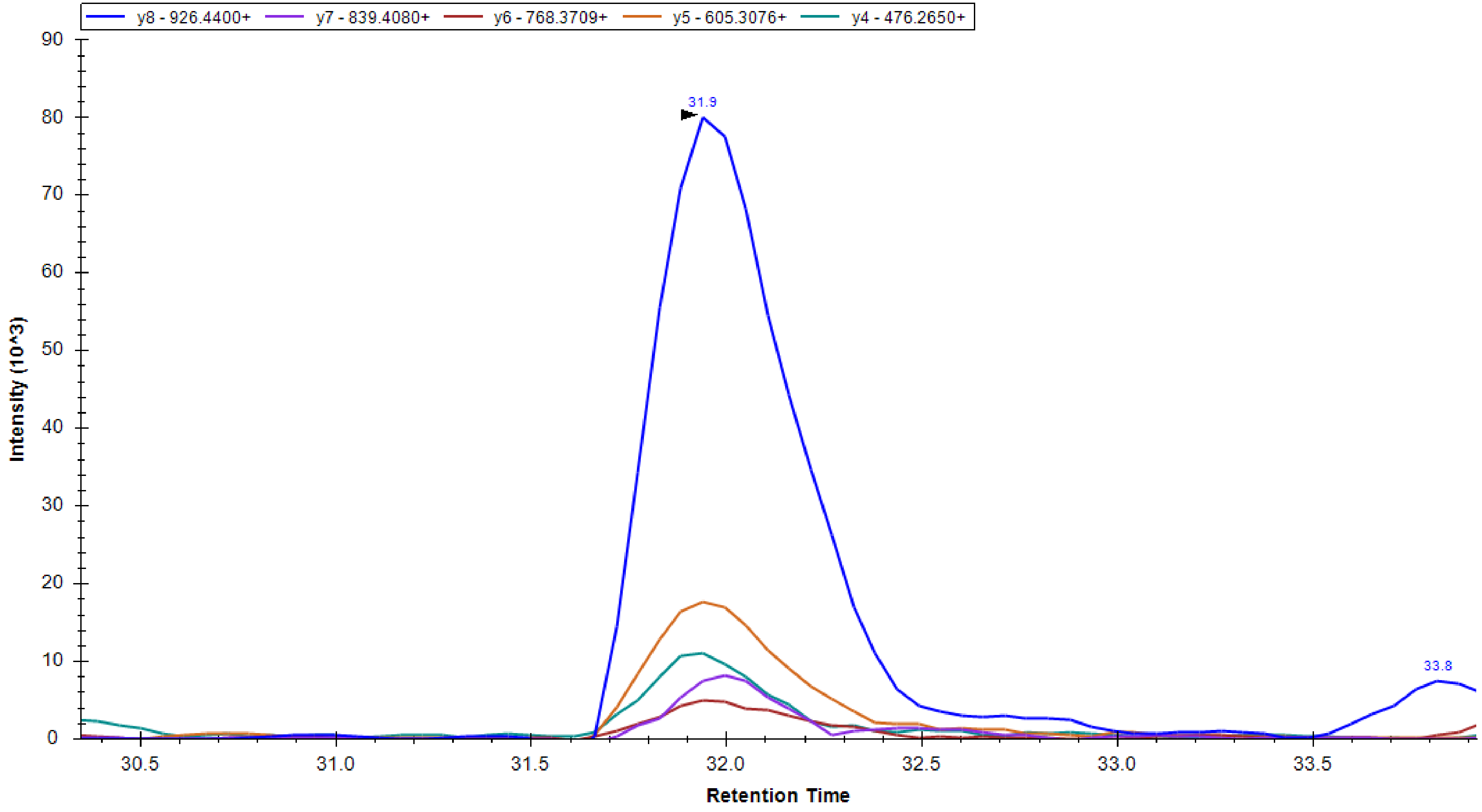

File name: 1604_JinanU(GZ)_MRM_#B_2nd_Round_Screen.skyd

Parent ion m/z and charges: 683.3452++

NR_002826.2.1
FTSPGAIPFISFMQR

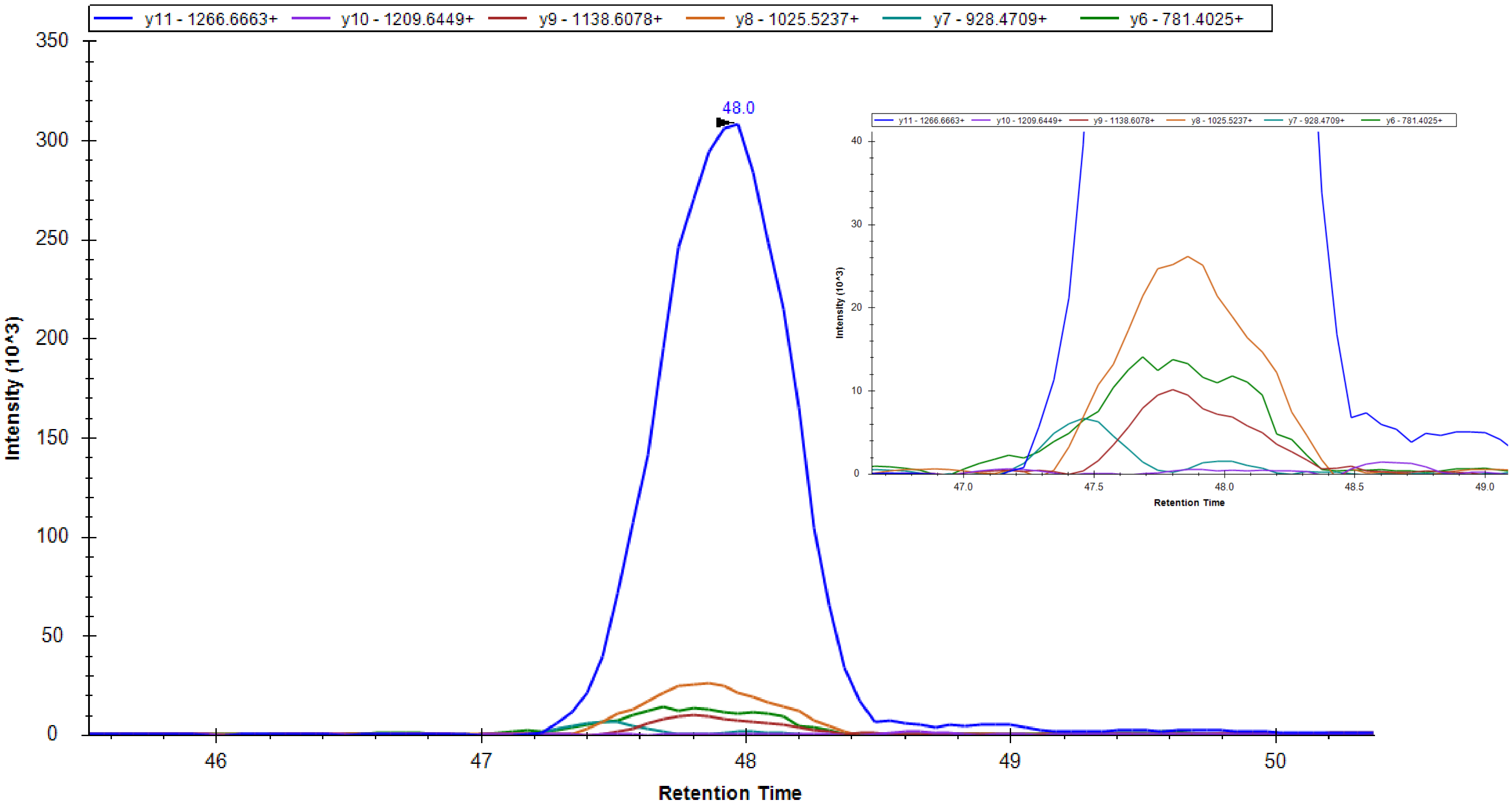

File name: 391-new#B-Round02_Negative-screen.skyd

Parent ion m/z and charges: 849.9373++

NR_002929.2.1
EEIMALVIDNGSSTCK

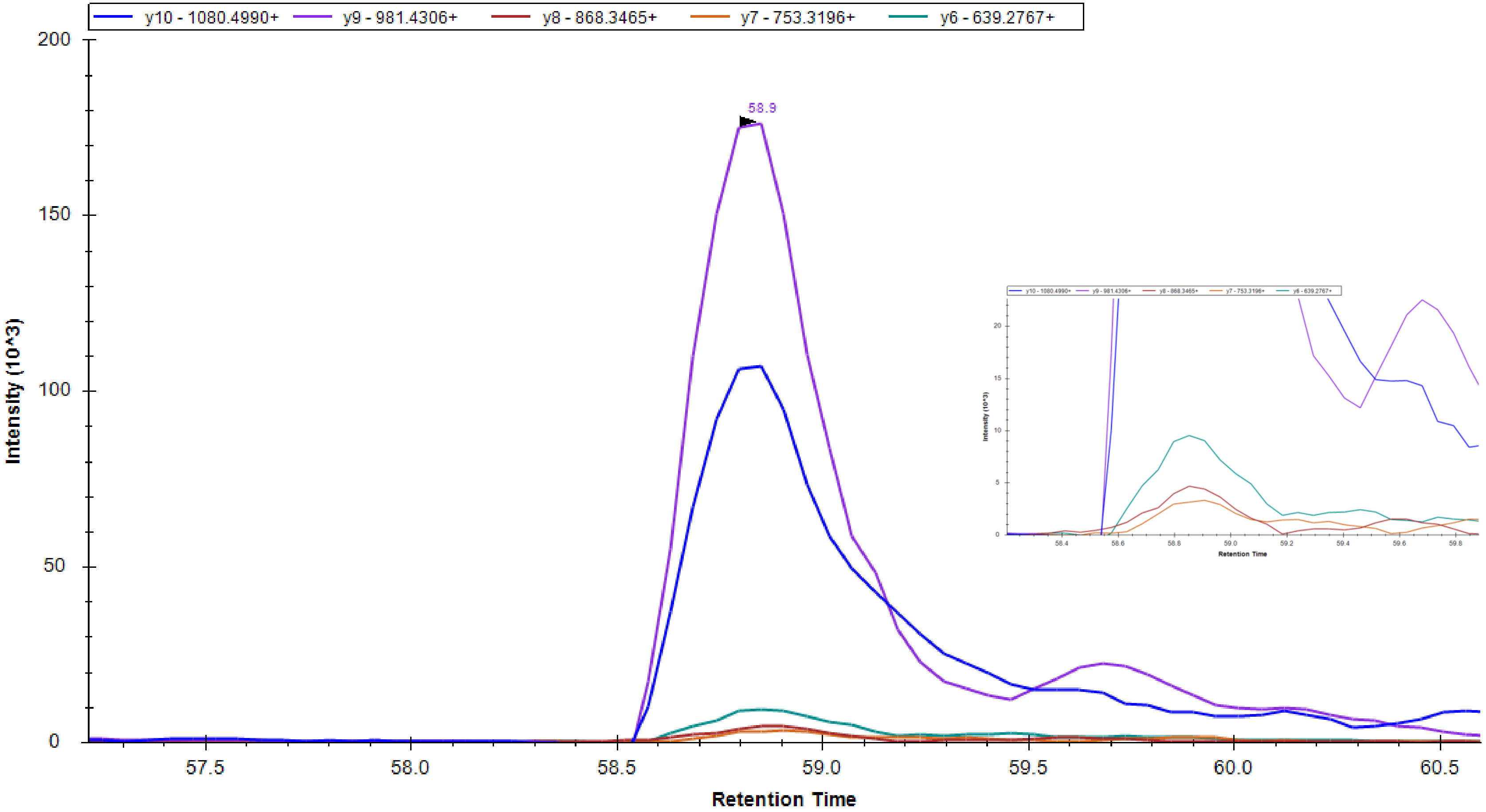

File name: 1604_JinanU(GZ)_MRM_#B_2nd_Round_Screen.skyd

Parent ion m/z and charges: 883.9186++

NR_003587.1.1 LAGLAGLGGMPRASPGGR

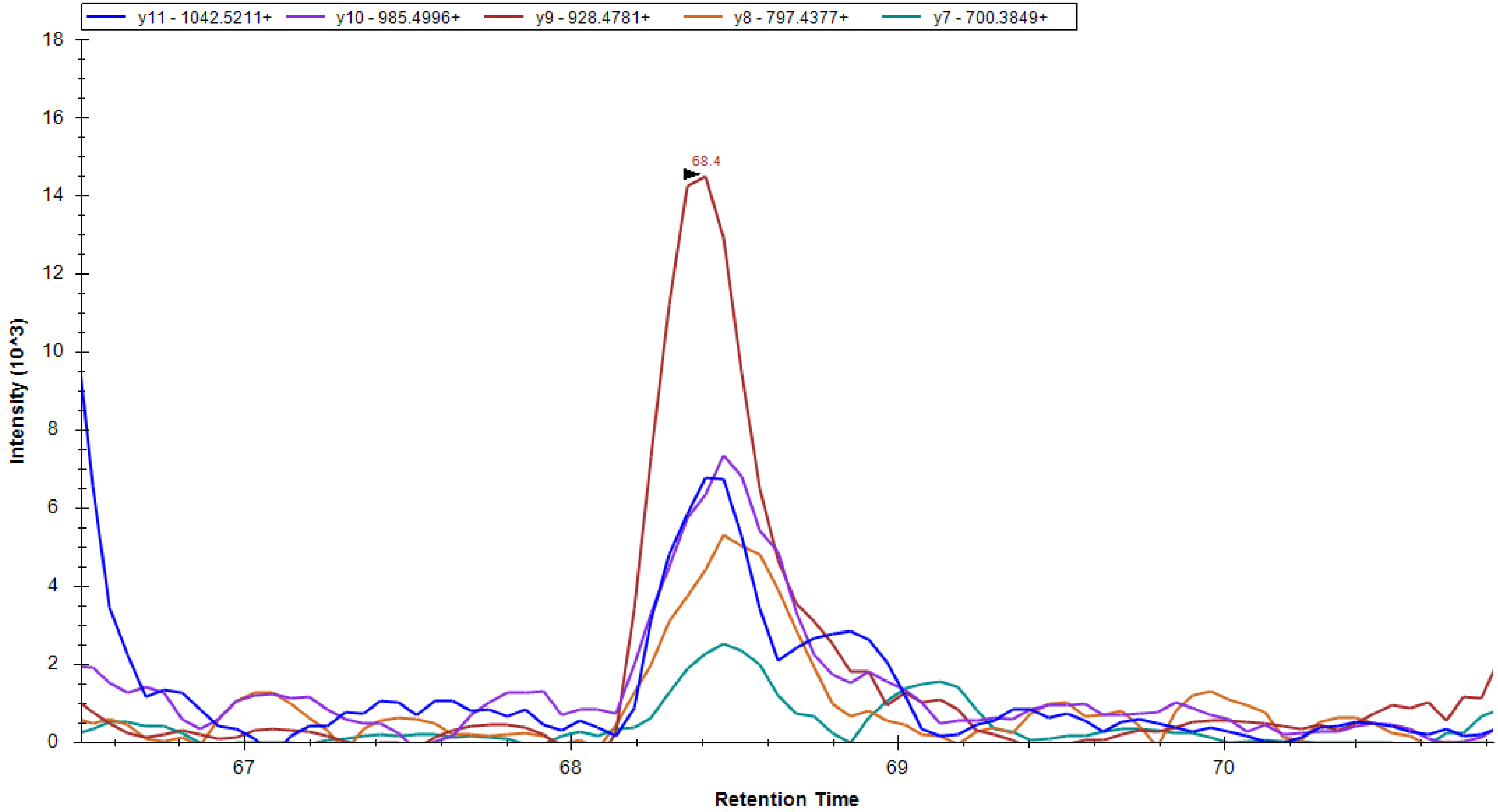

File name: 1604_JinanU(GZ)_MRM_#B_2nd_Round_Screen.skyd

Parent ion m/z and charges: 819.4488++

NR_003587.1.1
IIAGIDSIER

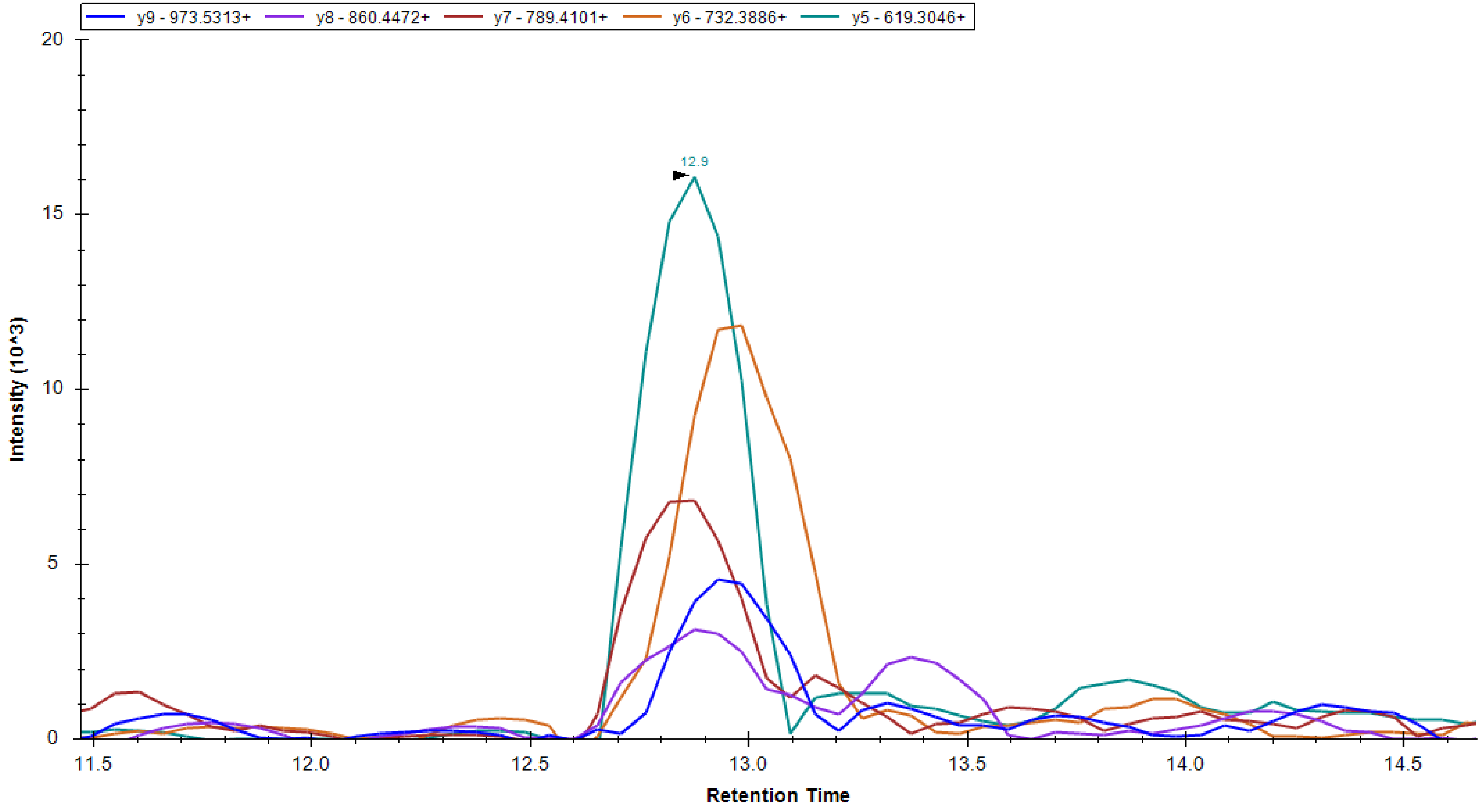

File name: 1604_JinanU(GZ)_MRM_#B_2nd_Round_Screen.skyd

Parent ion m/z and charges: 543.8113++

NR_003587.1.1
IAGIAGIGGMPRASPGGR

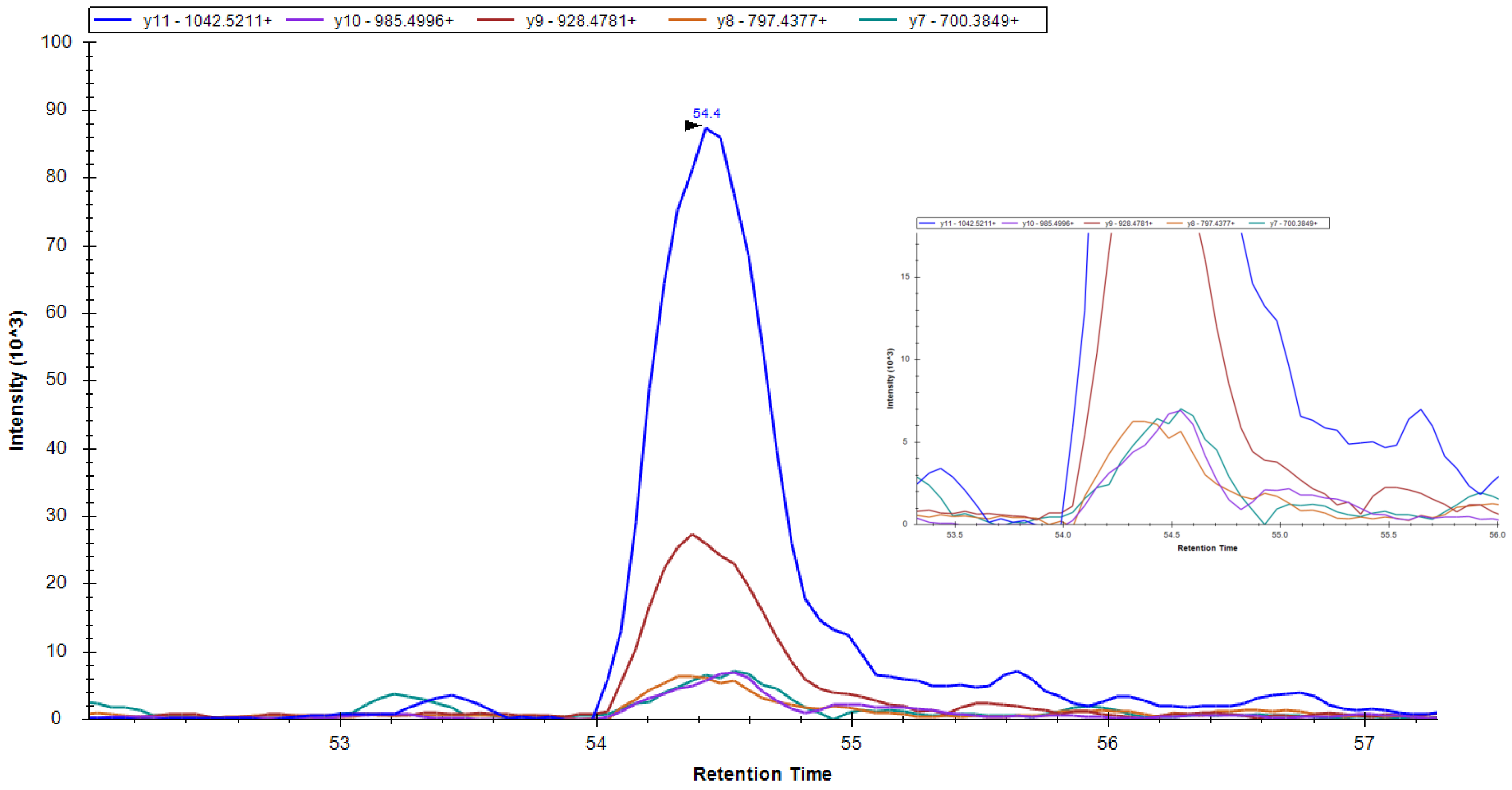

File name: 1604_JinanU(GZ)_MRM_#B_2nd_Round_Screen.skyd

Parent ion m/z and charges: 819.4488++

NR_003587.2.12
FFPVSGESGSDVQLLAVSHRALAHDPGLEVGAGGKVTSVH

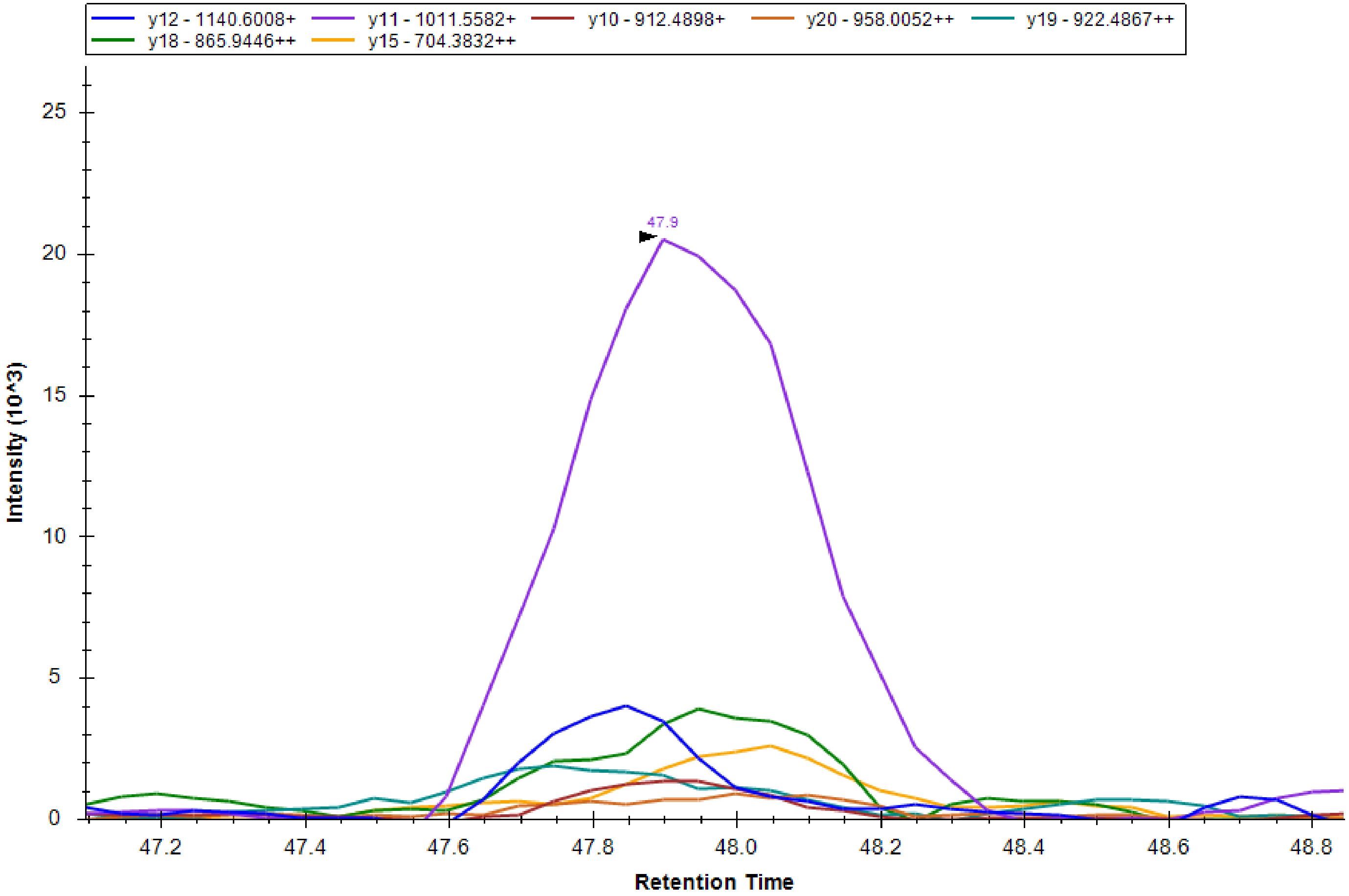

File name: 1604_JinanU(GZ)_MRM_#A_Full-Screen_0421.skyd

Parent ion m/z and charges: 1007.7711++++

NR_003587.2.12
FFPVSGESGSDVQLLAVSHRALAHDPGLEVGAGGKVTSVH

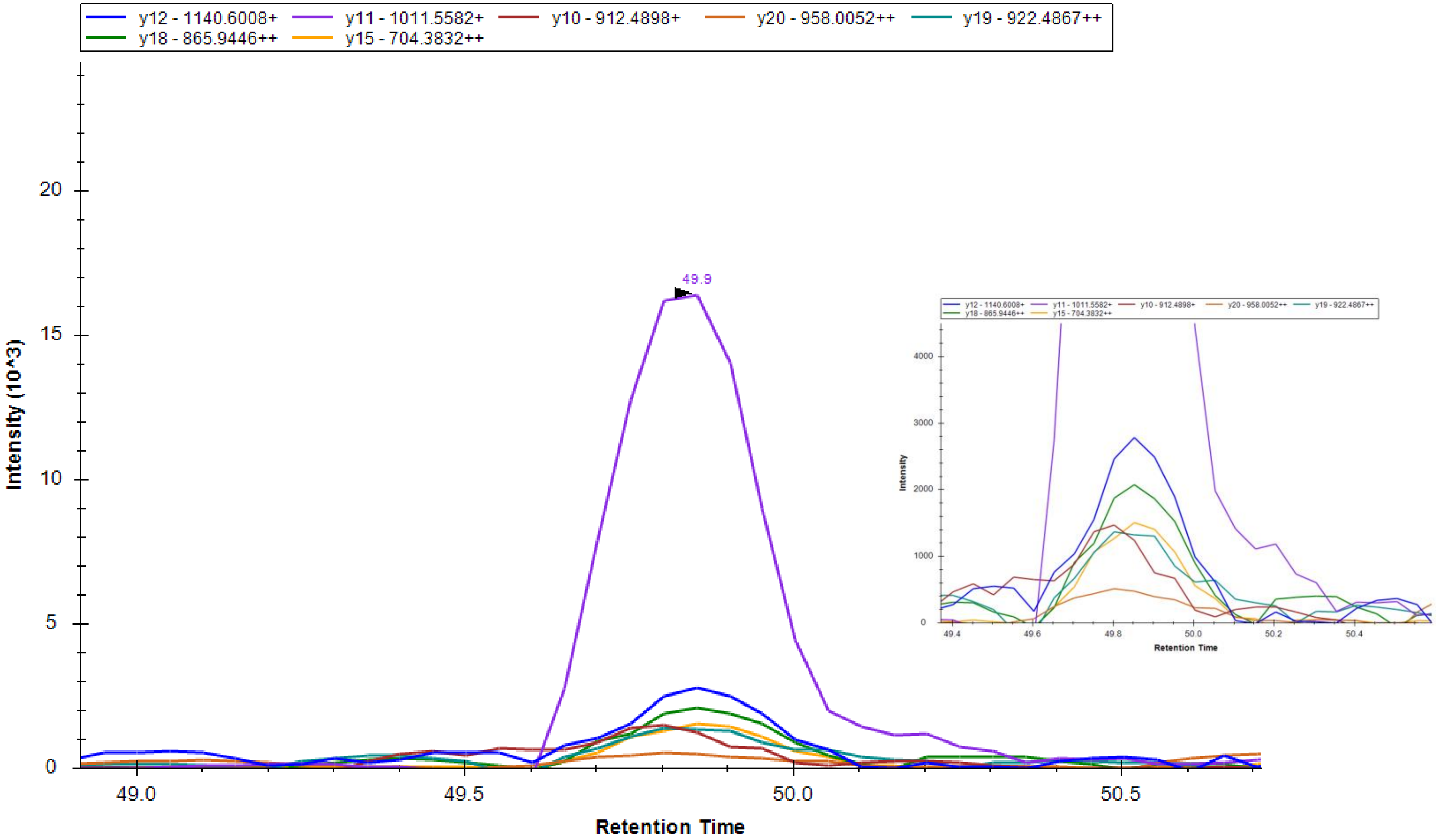

File name: 1604_JinanU(GZ)_MRM_#B_Full-Screen_0421.skyd

Parent ion m/z and charges: 1007.7711++++

NR_003587.2.12
SLRSFAEVLGVECR

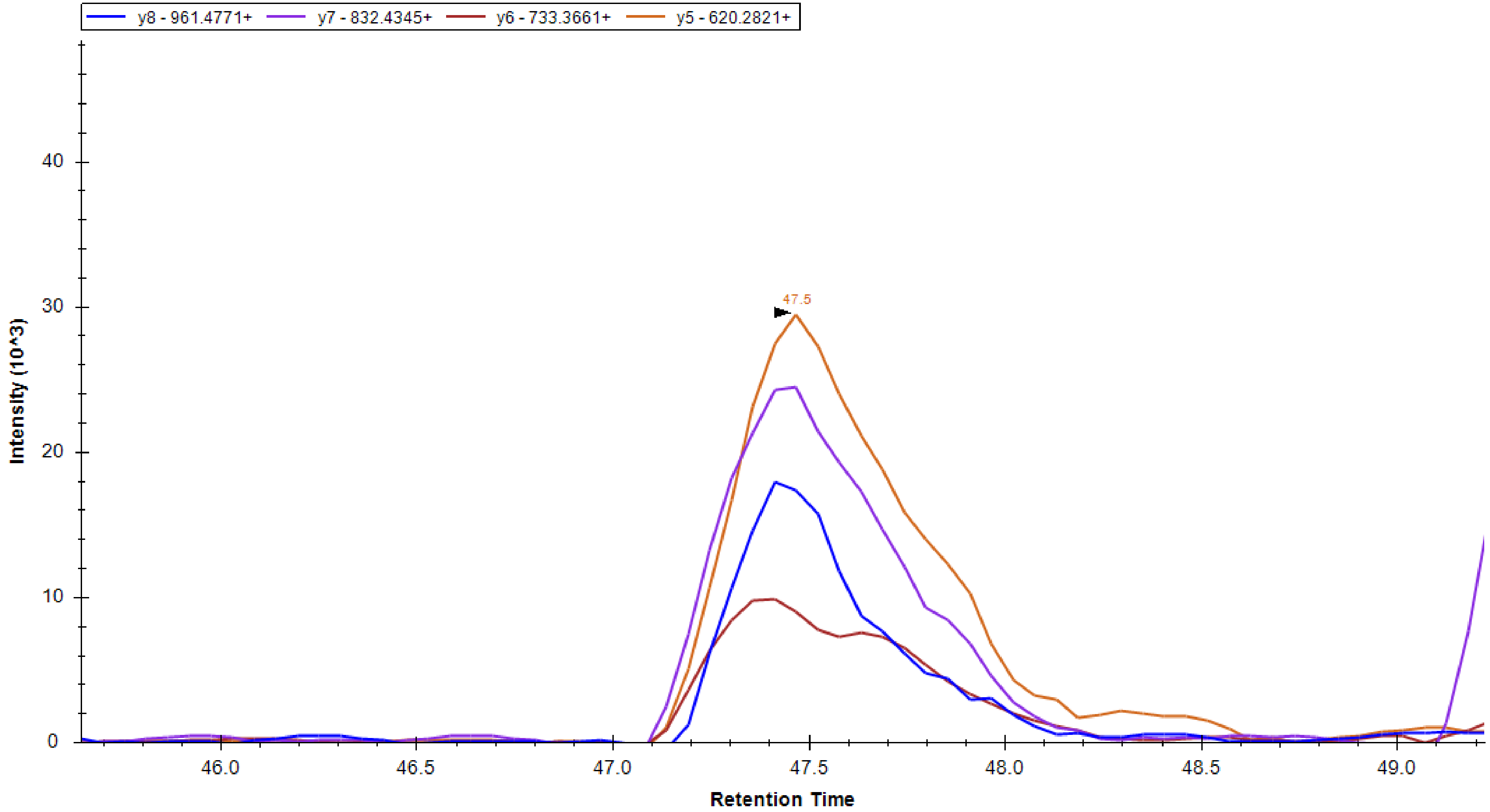

File name: 1604_JinanU(GZ)_MRM_#B_2nd_Round_Screen.skyd

Parent ion m/z and charges: 811.9196++

NR_003587.2.12
FFPVSGESGSDVQLLAVSHRALAHDPGLEVGAGGKVTSVH

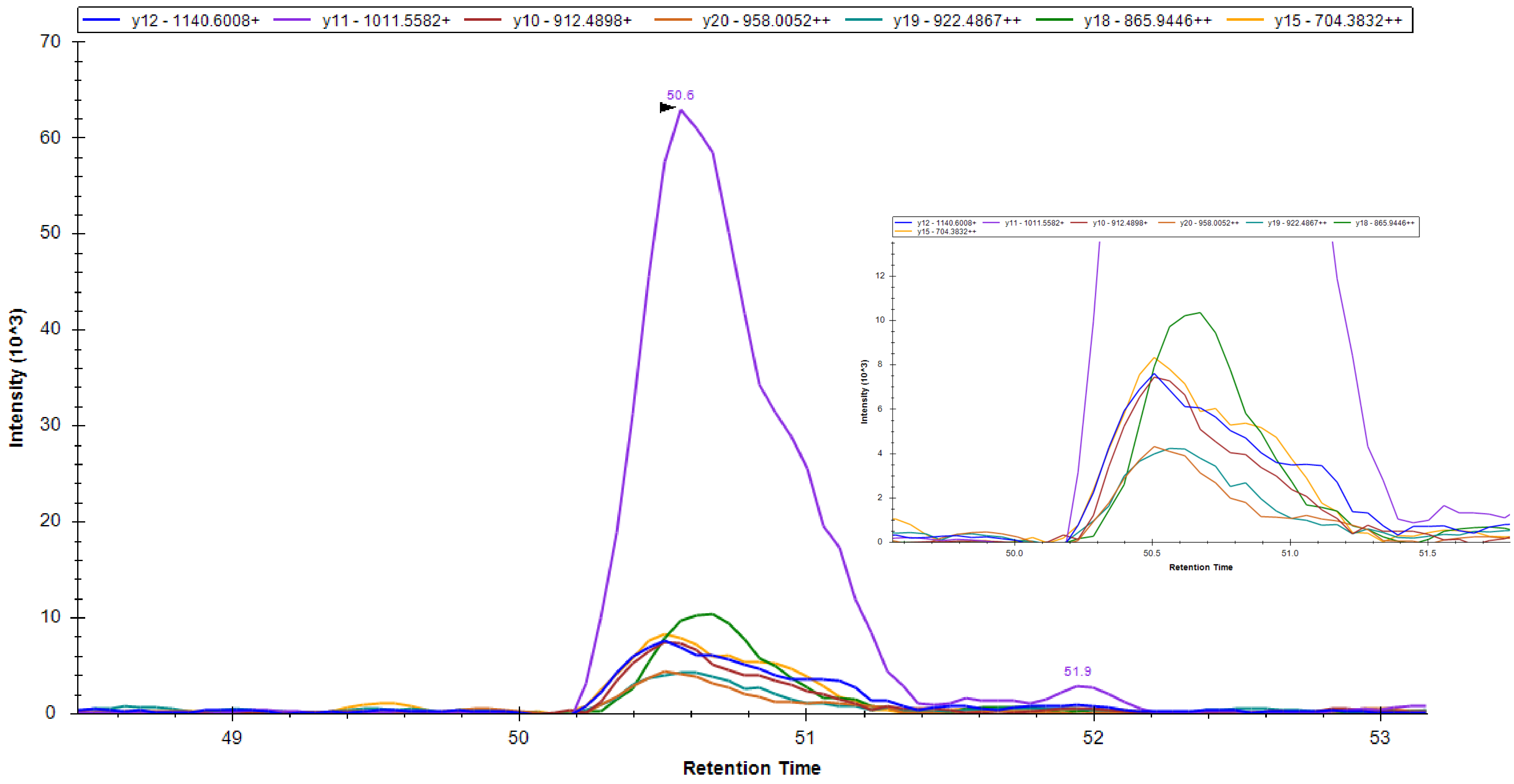

File name: 1604_JinanU(GZ)_MRM_#B_2nd_Round_Screen.skyd

Parent ion m/z and charges: 1007.7711++++

NR_003677.3.1
LYPAAVDTIVAVTAEGK

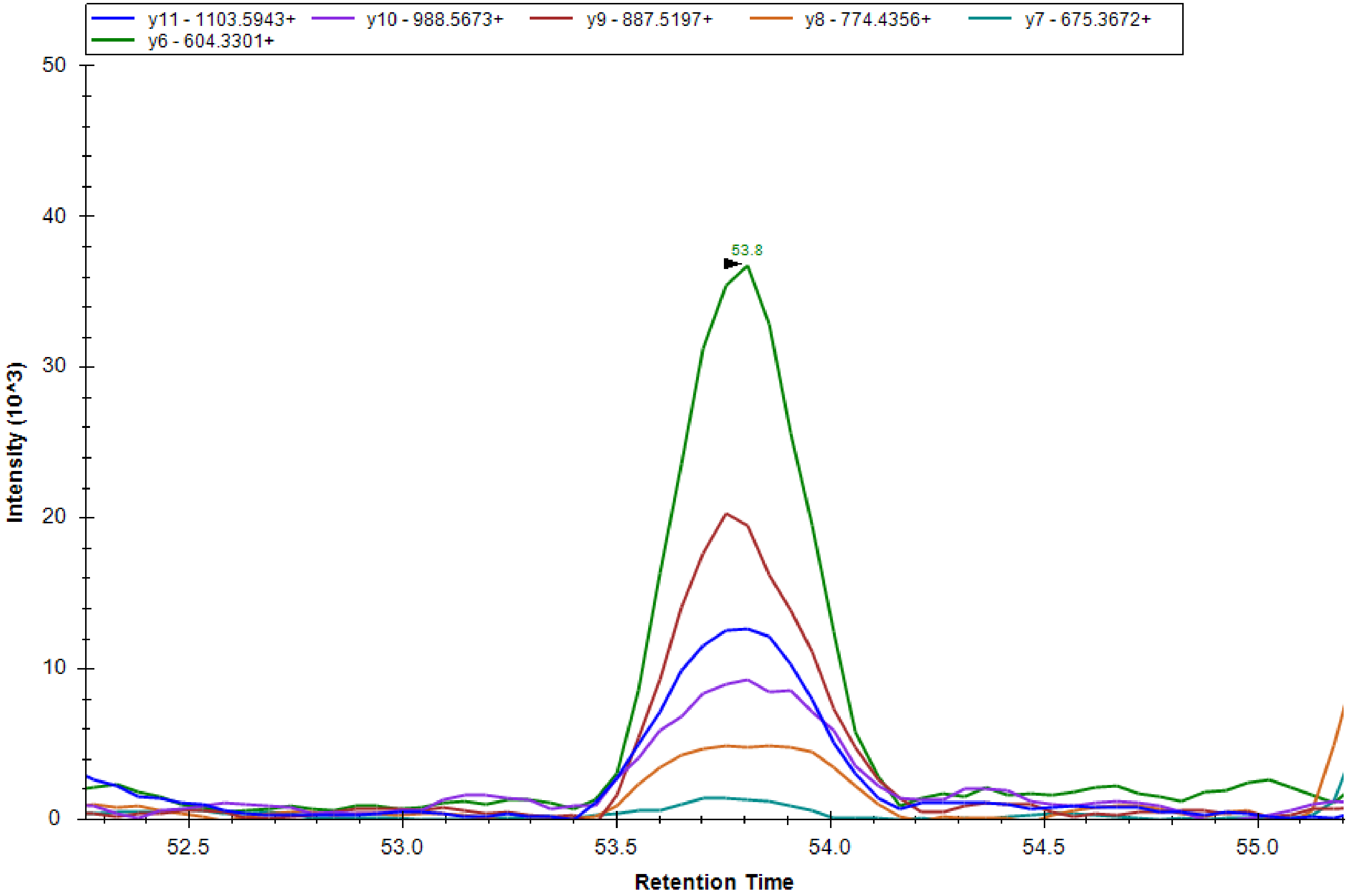

File name: 1604_JinanU(GZ)_MRM_#A_Full-Screen_0421.skyd

Parent ion m/z and charges: 859.4722++

NR_003677.3.1
SQLVEQFPGIEPWLNQIMPK

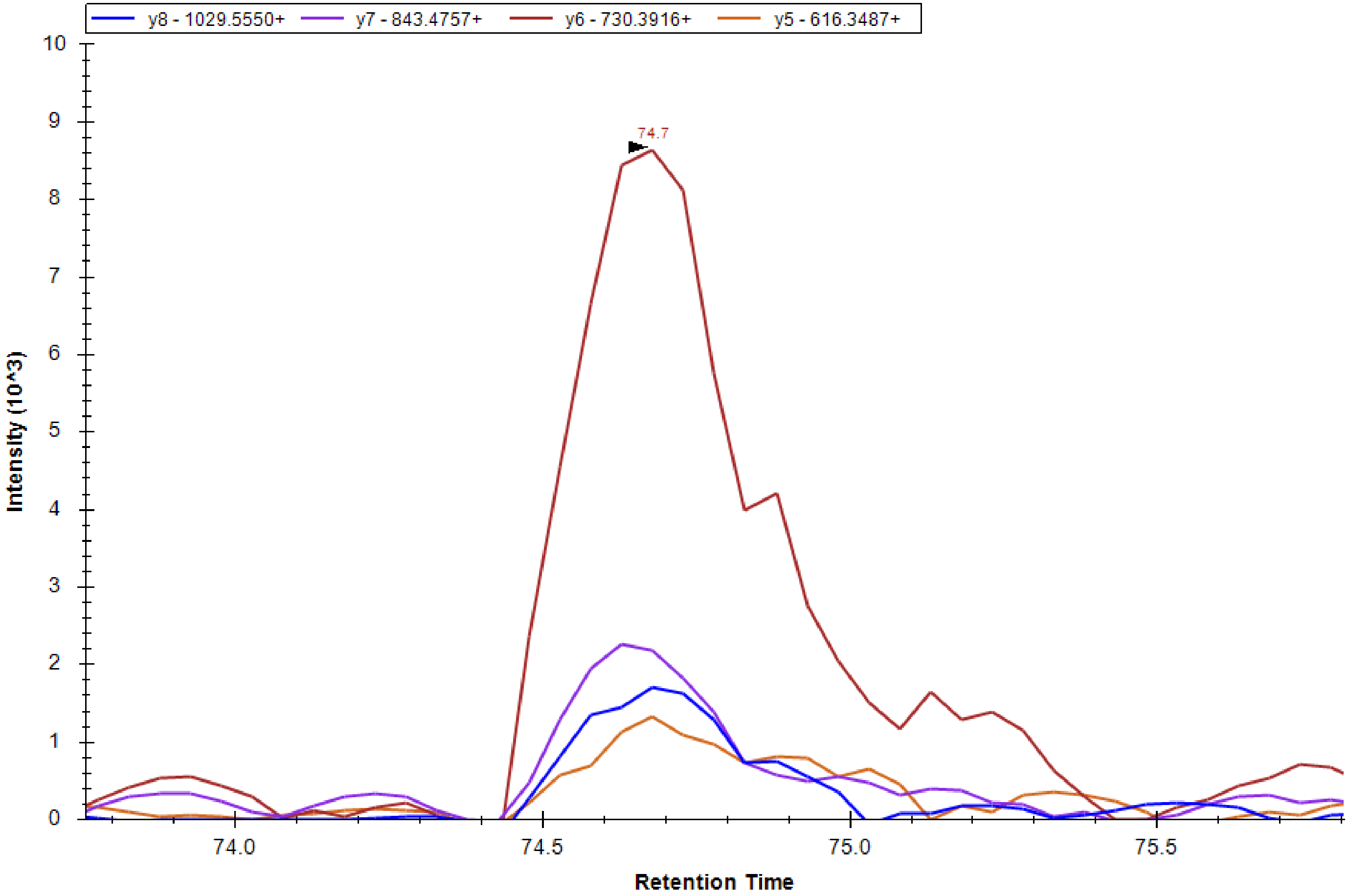

File name: 1604_JinanU(GZ)_MRM_#A_Full-Screen_0421.skyd

Parent ion m/z and charges: 1177.6143++

NR_003677.3.1
LYPAAVDTIVAVTAEGK

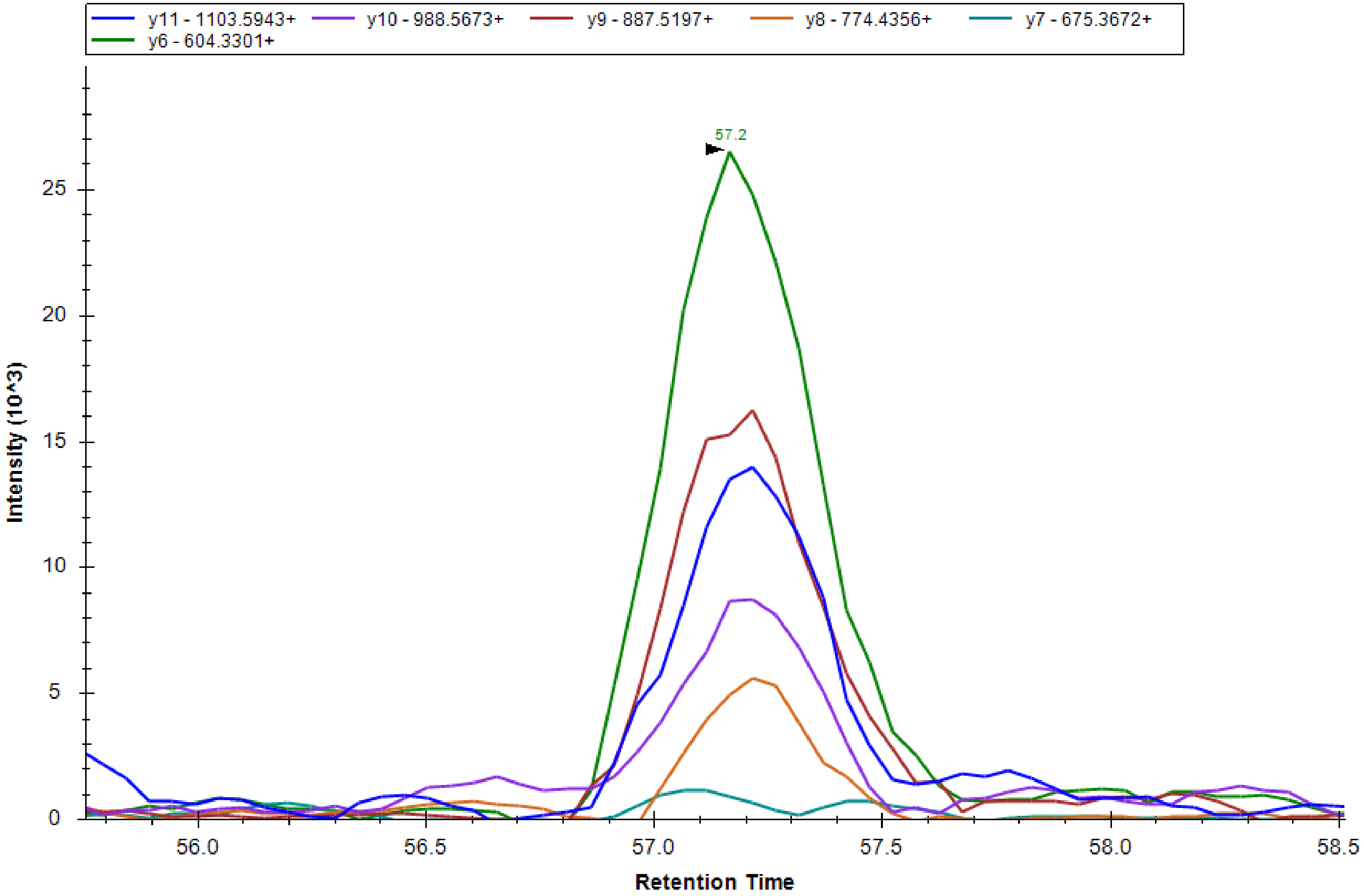

File name: 1604_JinanU(GZ)_MRM_#B_Full-Screen_0421.skyd

Parent ion m/z and charges: 859.4722++

NR_003677.3.1
LYPAAVDTIVAVTAEGK

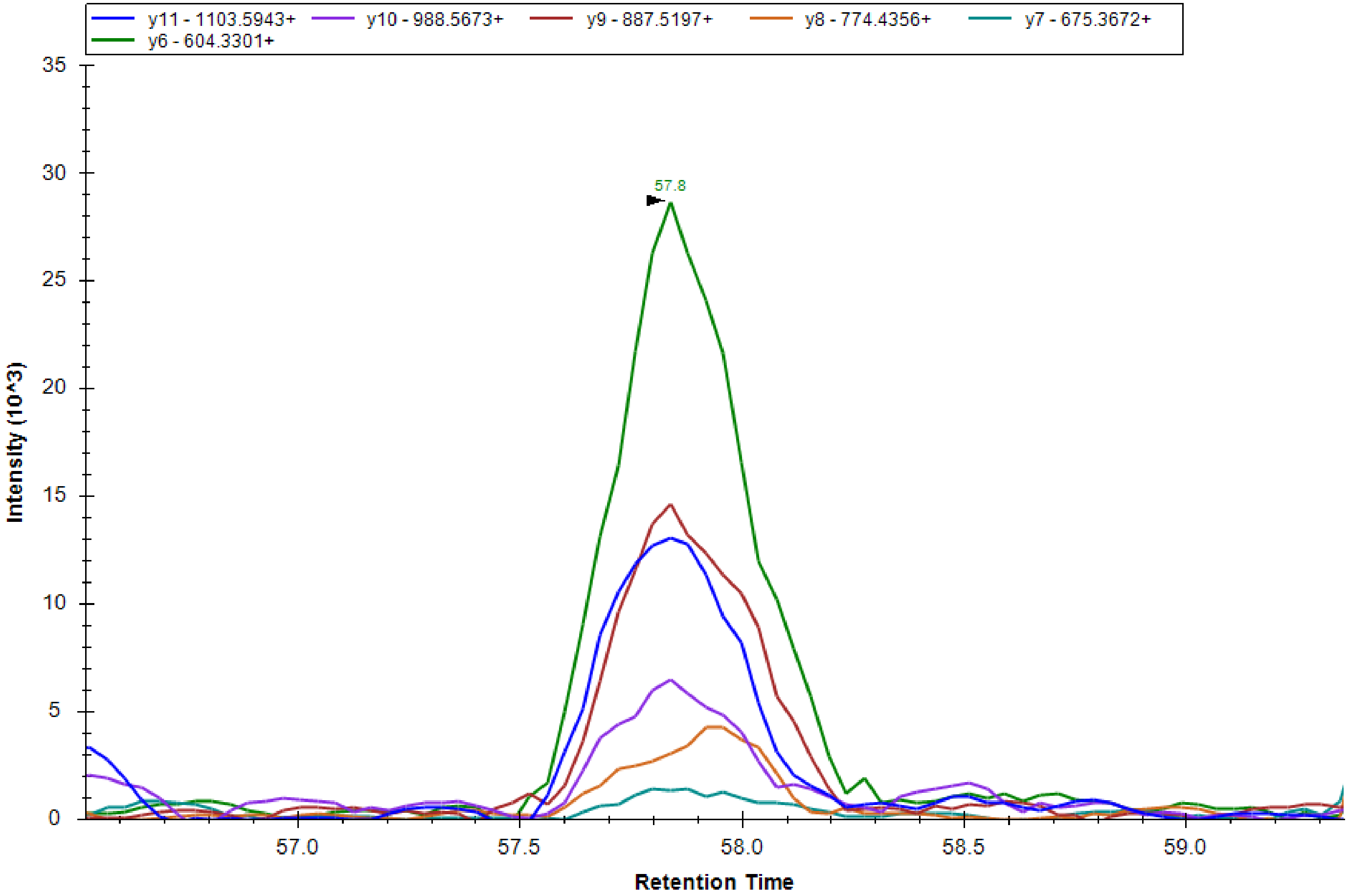

File name: 1604_JinanU(GZ)_MRM_#A_#B_Positive_Screen.skyd

Parent ion m/z and charges: 859.4722++

NR_015367.1.1
WDYPEGTPNGGSTTLPSAPPPASAGLK

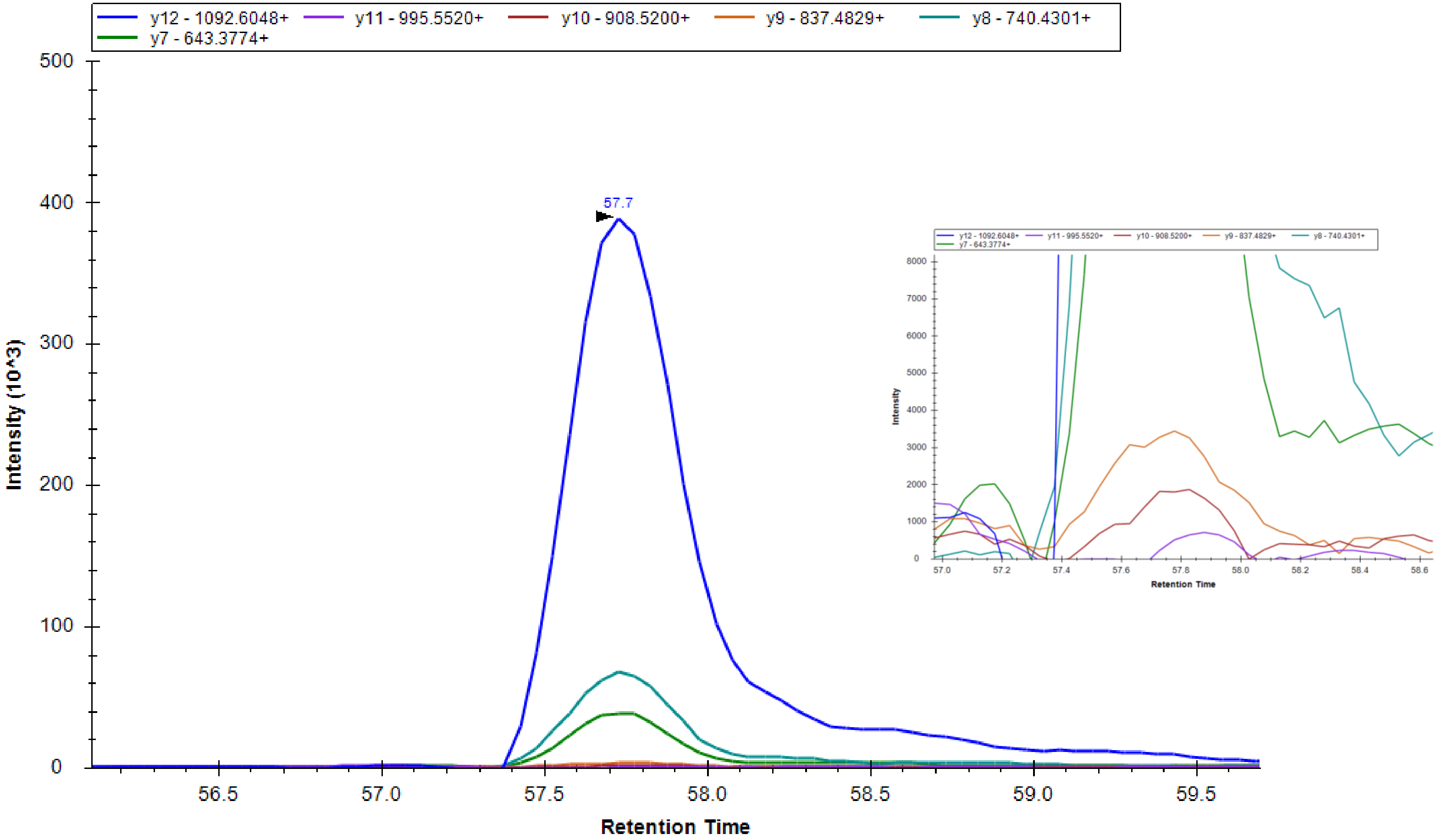

File name: 1604_JinanU(GZ)_MRM_#A_Full-Screen_0421.skyd

Parent ion m/z and charges: 890.1012+++

NR_015367.1.1
GDQPCASGR

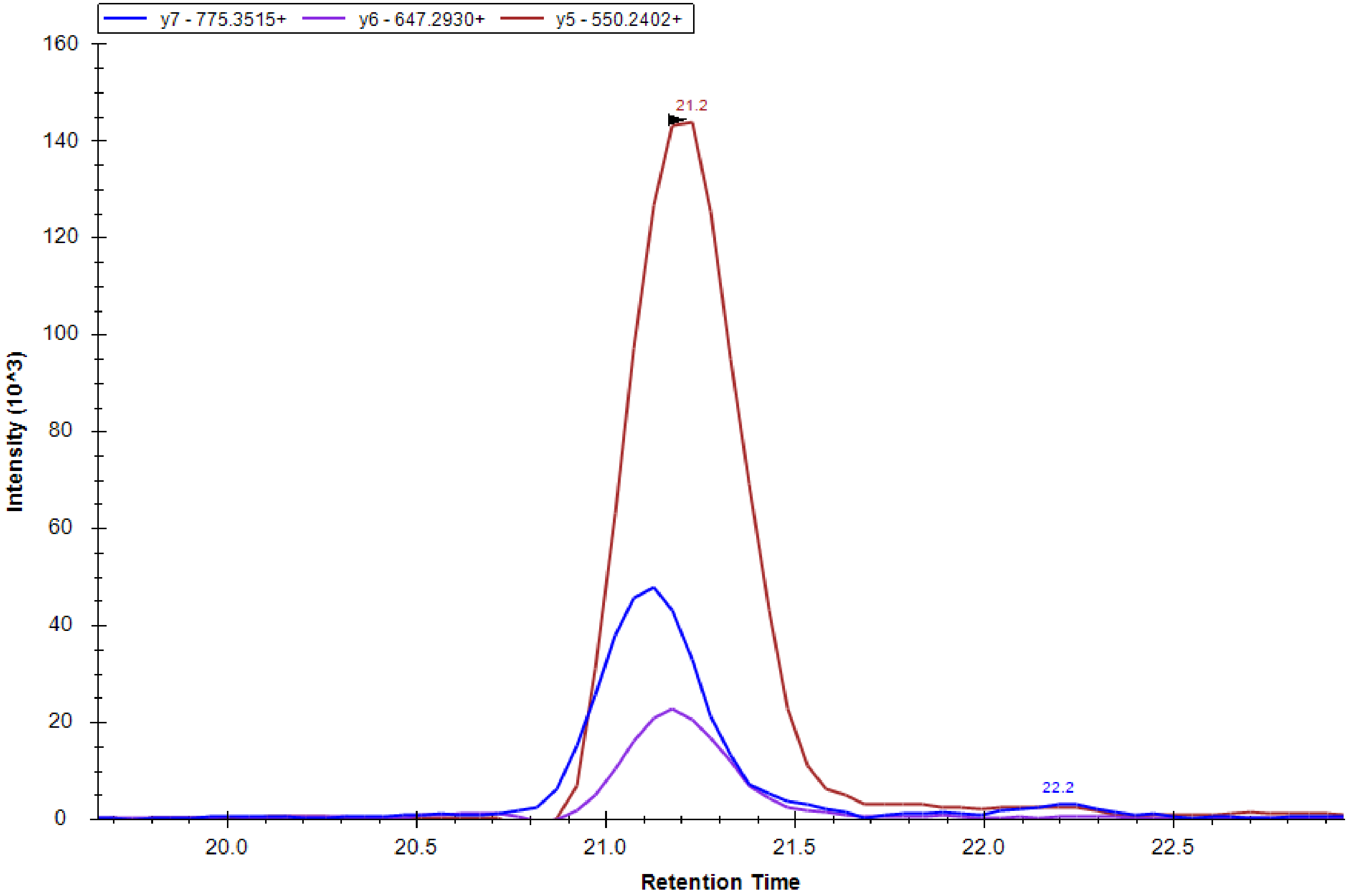

File name: 1604_JinanU(GZ)_MRM_#A_Full-Screen_0421.skyd

Parent ion m/z and charges: 474.2036++

NR_015367.1.1
WDYPEGTPNGGSTTLPSAPPPASAGLK

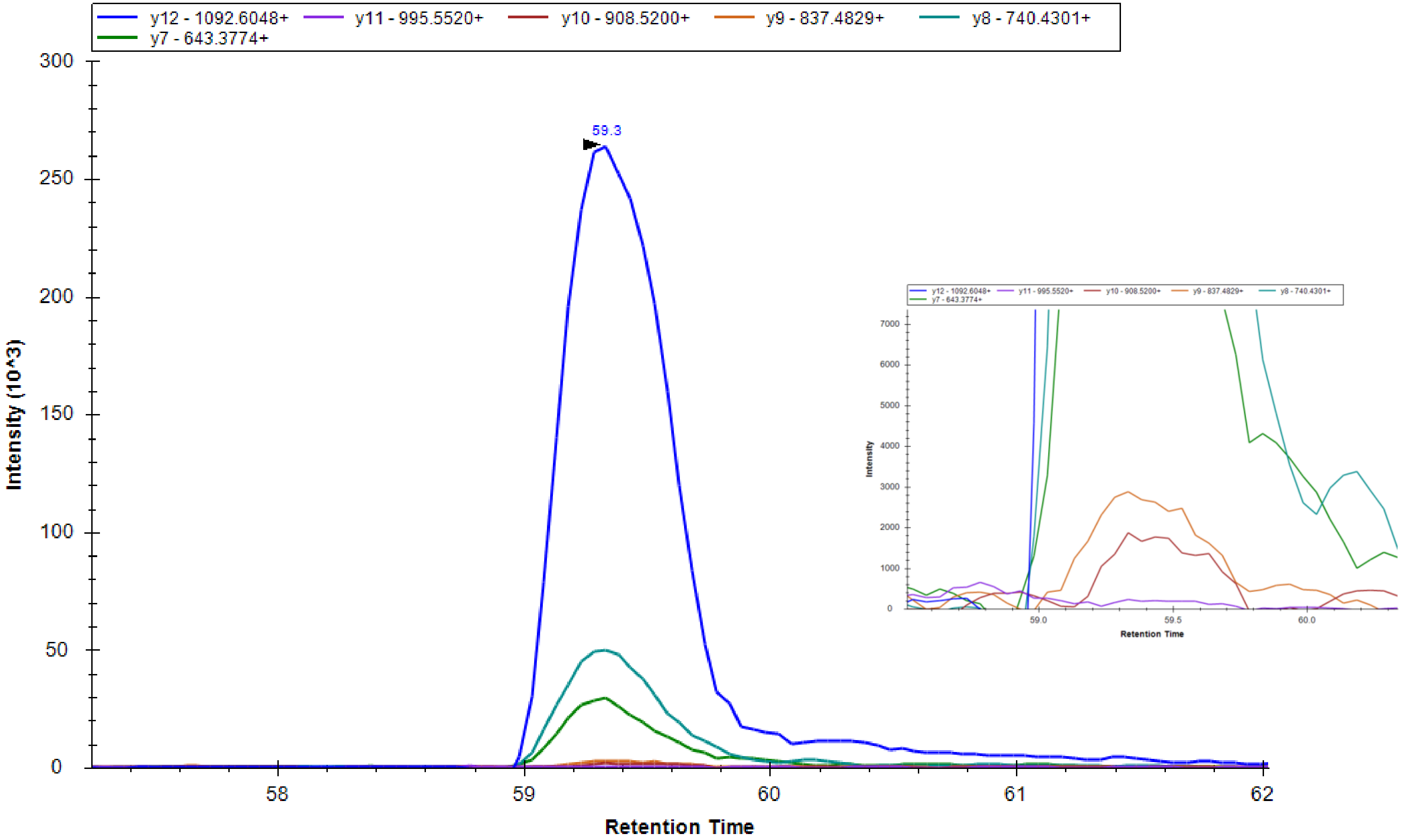

File name: 1604_JinanU(GZ)_MRM_#B_Full-Screen_0421.skyd

Parent ion m/z and charges: 890.1012+++

NR_015432.3.1
GFYVETVVTYKEDFVPNTEK

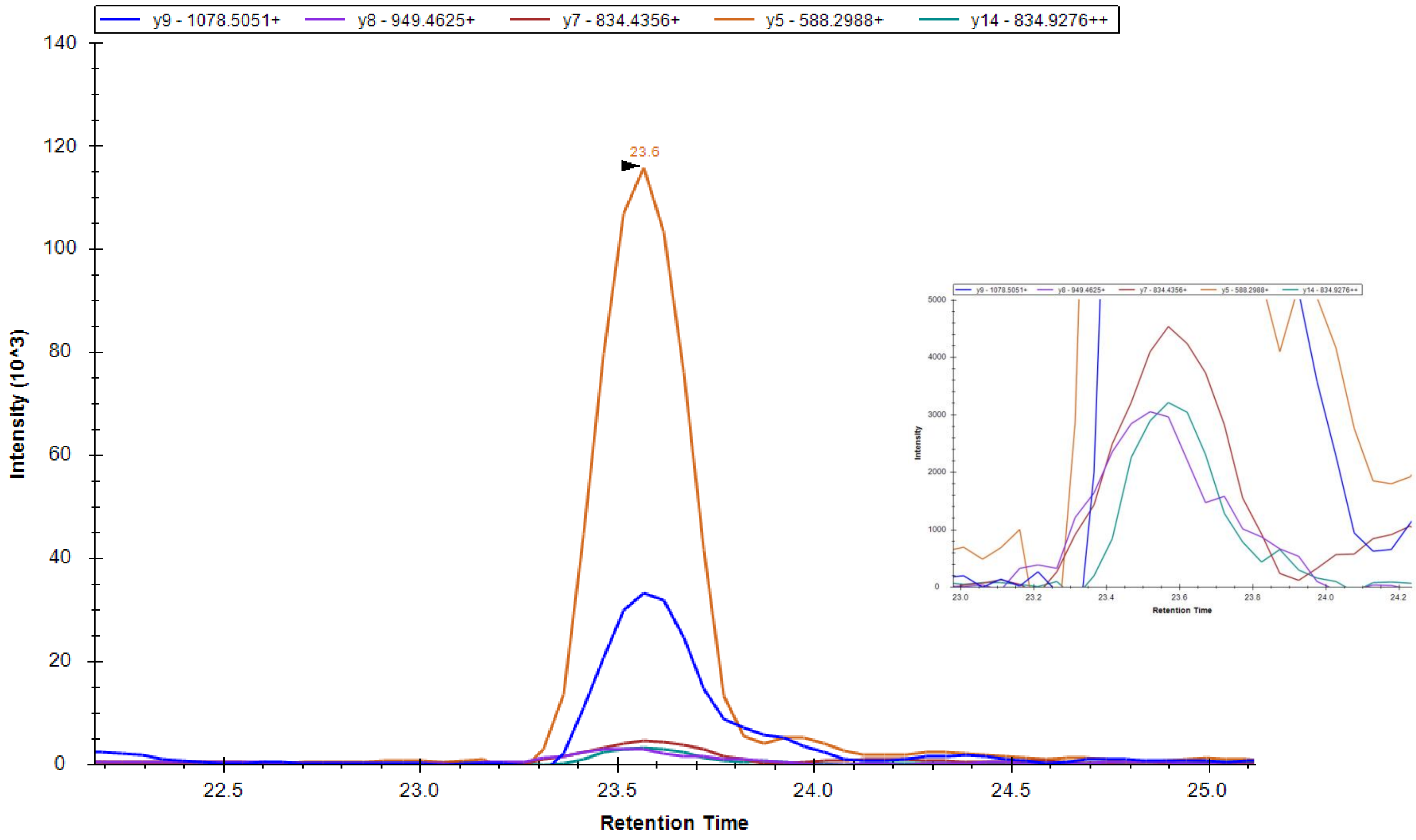

File name: 1604_JinanU(GZ)_MRM_#A_Full-Screen_0421.skyd

Parent ion m/z and charges: 789.0581+++

NR_015432.3.1
EDFVPNTEKIL

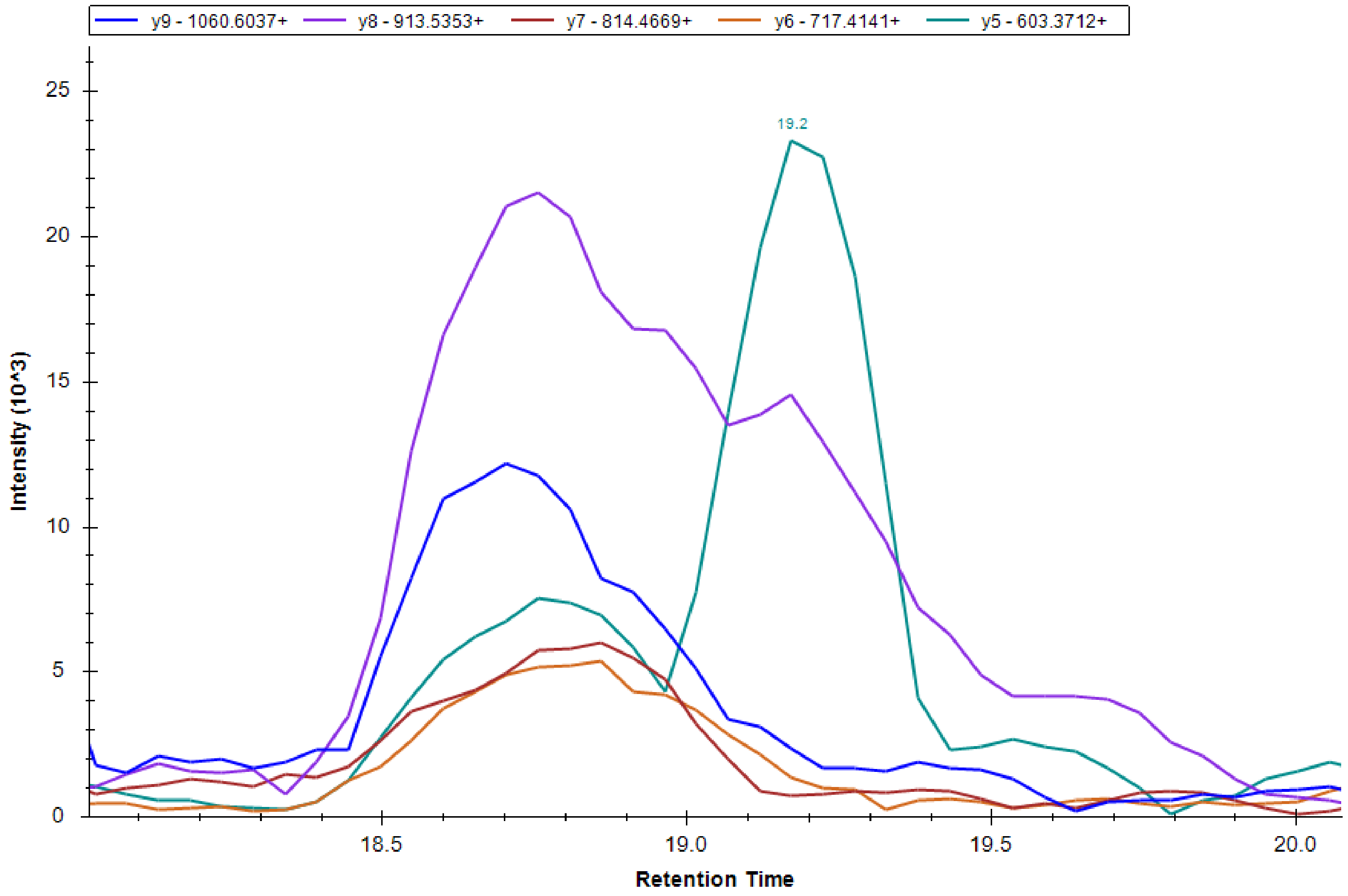

File name: 1604_JinanU(GZ)_MRM_#A_Full-Screen_0421.skyd

Parent ion m/z and charges: 652.8403++

NR_015432.3.1
EDFVPNTEK

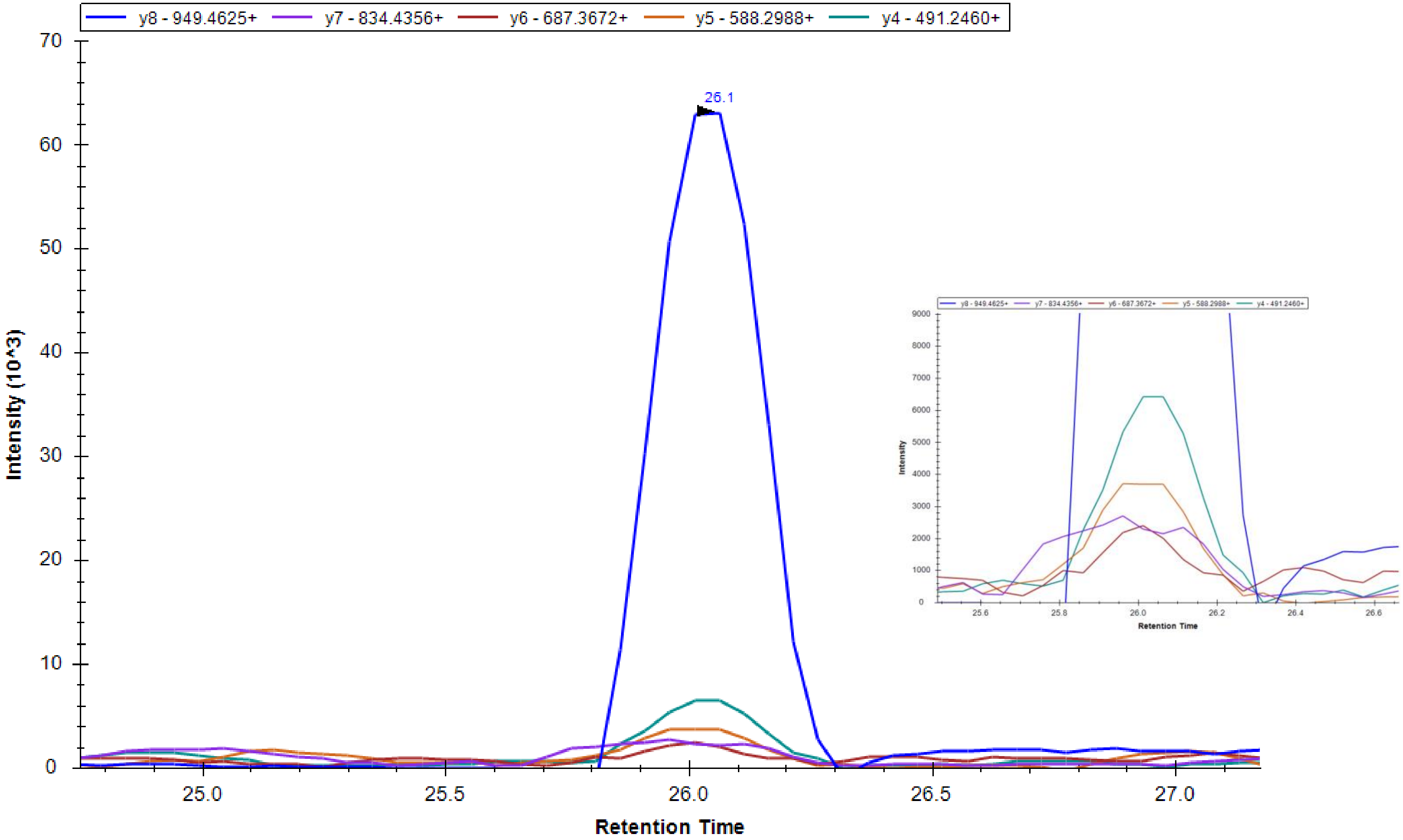

File name: 1604_JinanU(GZ)_MRM_#B_Full-Screen_0421.skyd

Parent ion m/z and charges: 539.7562++

NR_015432.3.1
GFYVETVVTYKEDFVPNTEK

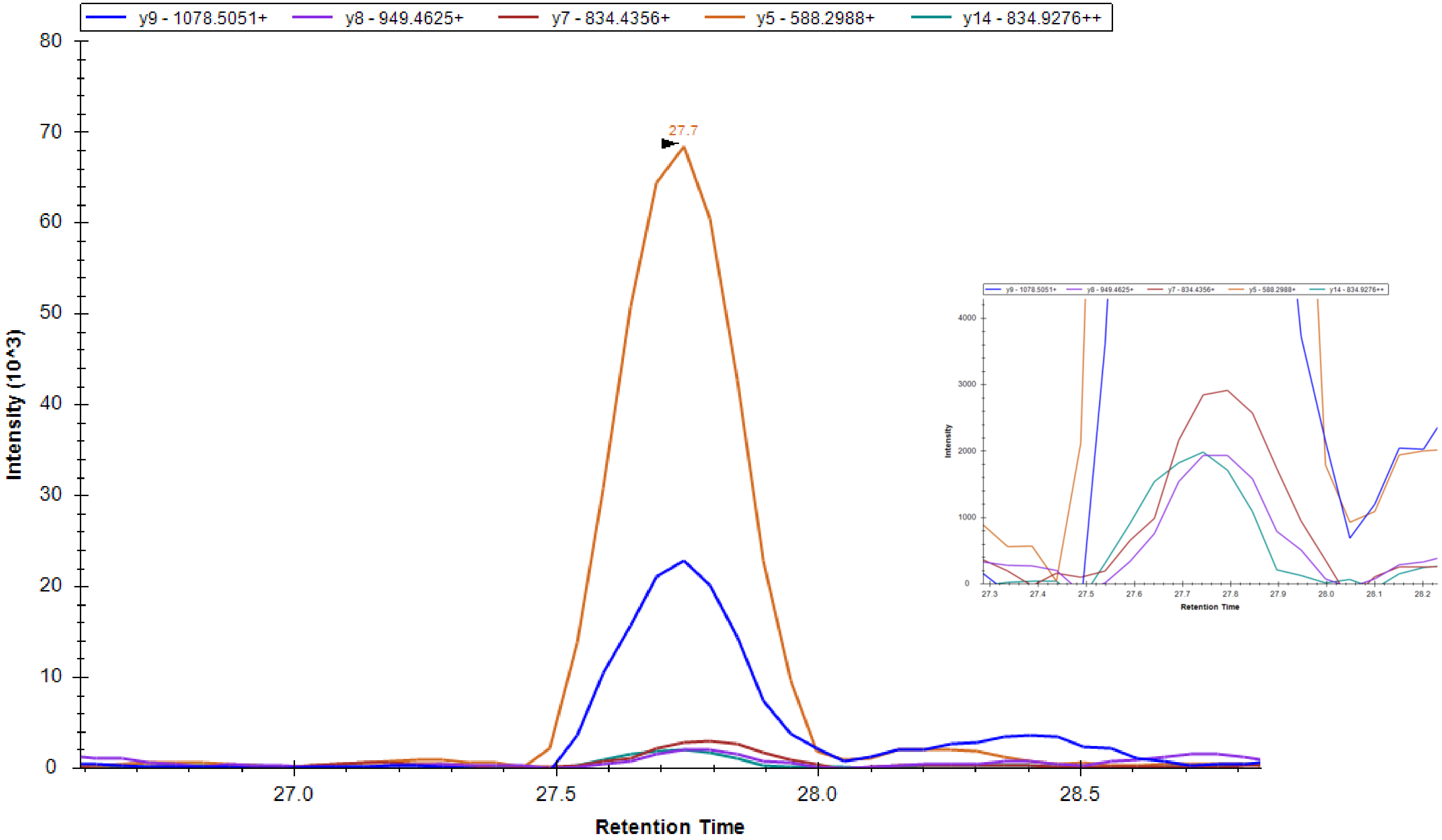

File name: 1604_JinanU(GZ)_MRM_#B_Full-Screen_0421.skyd

Parent ion m/z and charges: 789.0581+++

NR_015432.3.1
DGSASEVPSELSERPK

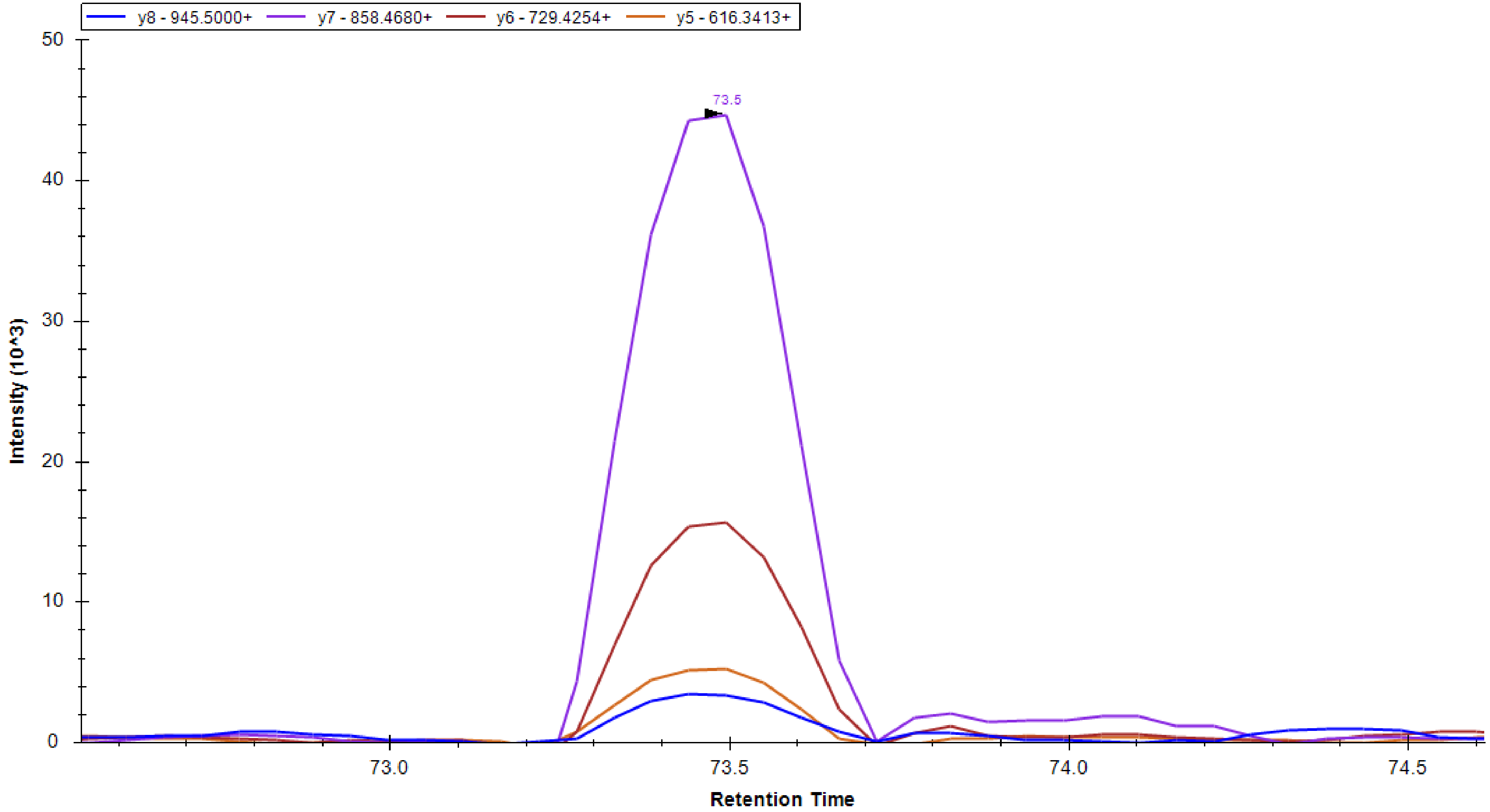

File name: 1604_JinanU(GZ)_MRM_#B_2nd_Round_Screen.skyd

Parent ion m/z and charges: 844.4103++

NR_015432.3.1
EDFVPNTEK

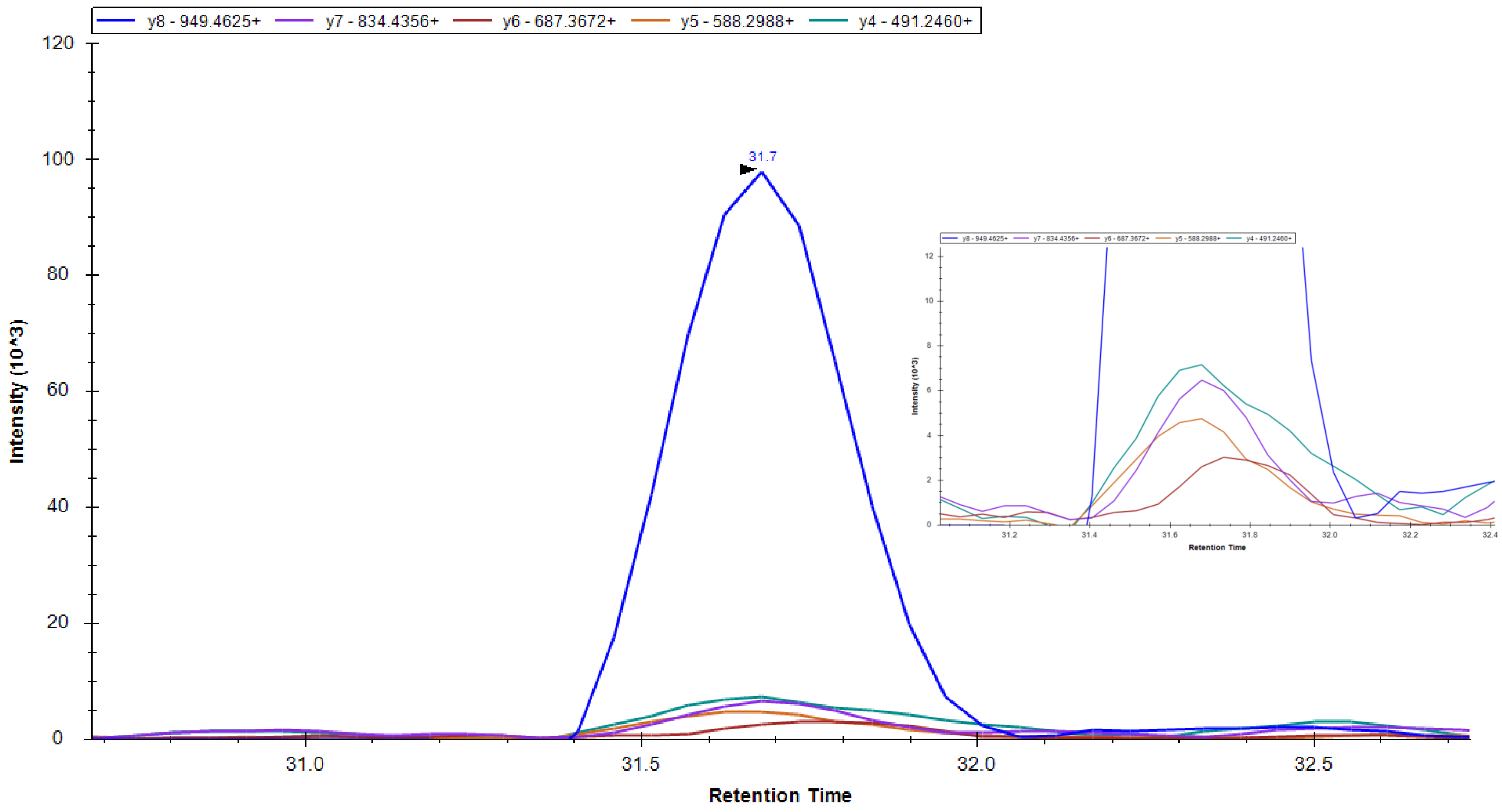

File name: 1604_JinanU(GZ)_MRM_#B_2nd_Round_Screen.skyd

Parent ion m/z and charges: 539.7562++

NR_015432.3.1
GFYVETVVTYKEDFVPNTEK

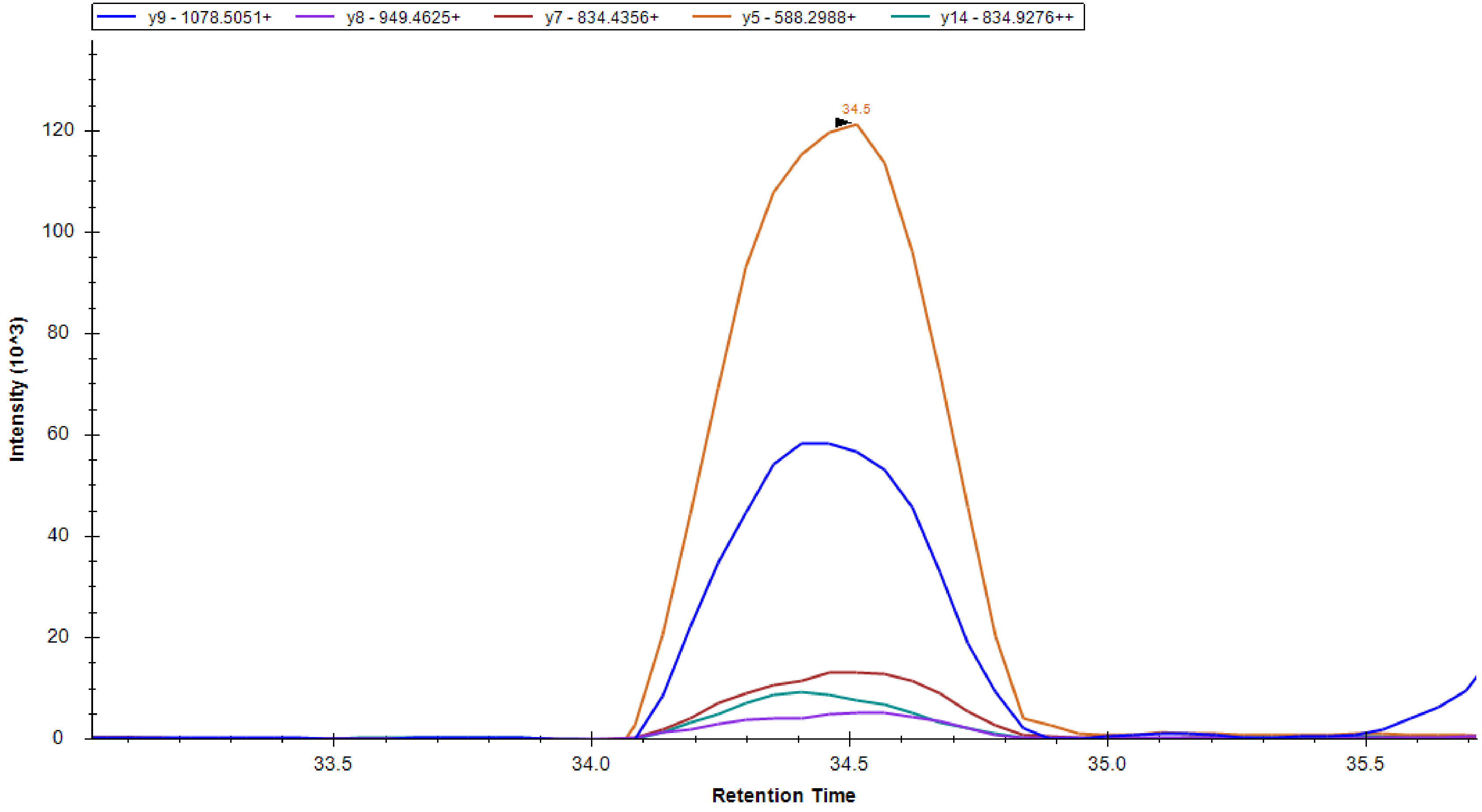

File name: 1604_JinanU(GZ)_MRM_#B_2nd_Round_Screen.skyd

Parent ion m/z and charges: 789.0581+++

NR_024022.3.1
PRPGPFSVDPRHHLSKGVVLFSPFPEAQNR

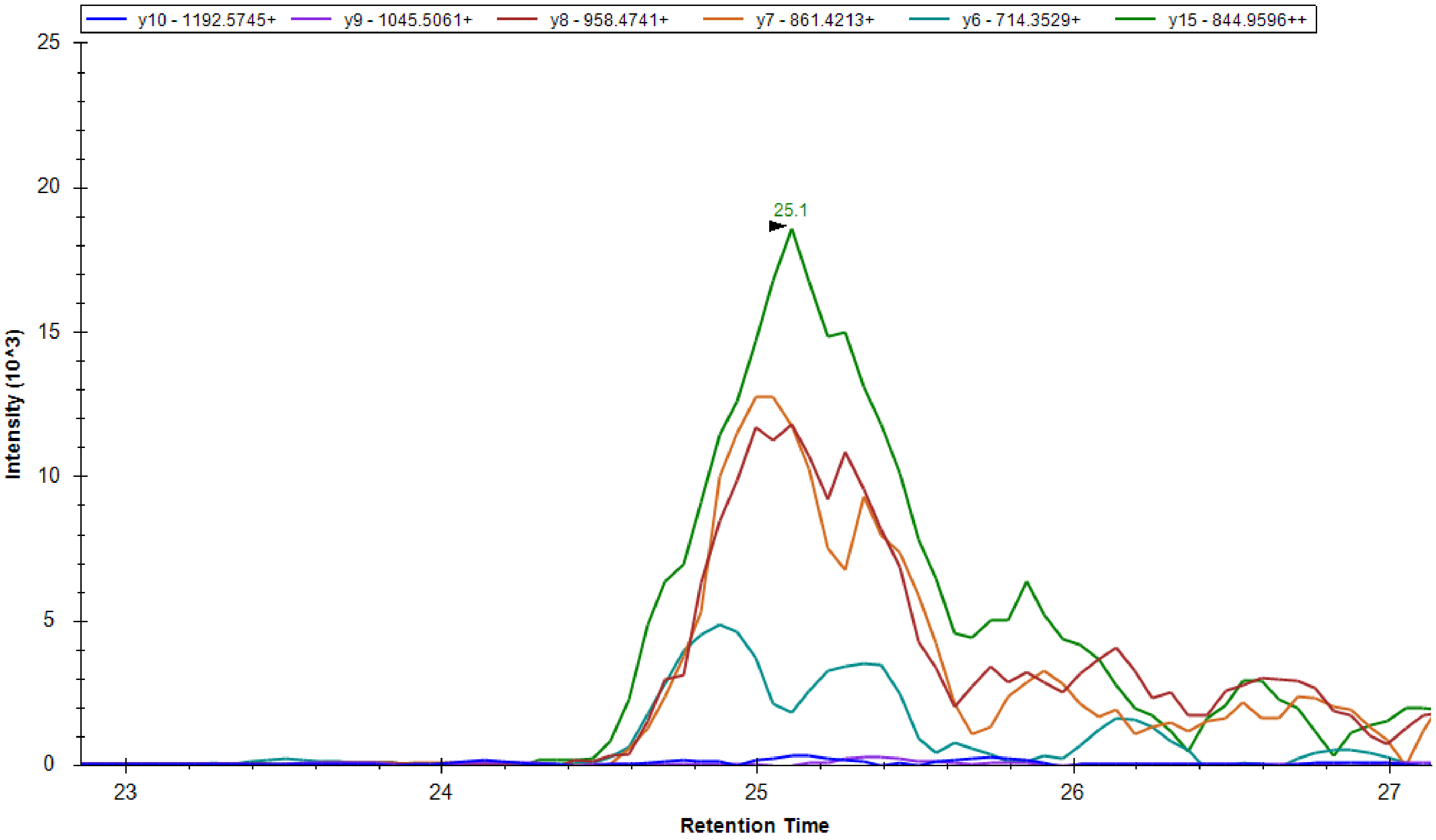

File name: 391-new#B-Round02_Negative-screen.skyd

Parent ion m/z and charges: 1123.5970+++

NR_024157.2.7
VLKNRDTITK

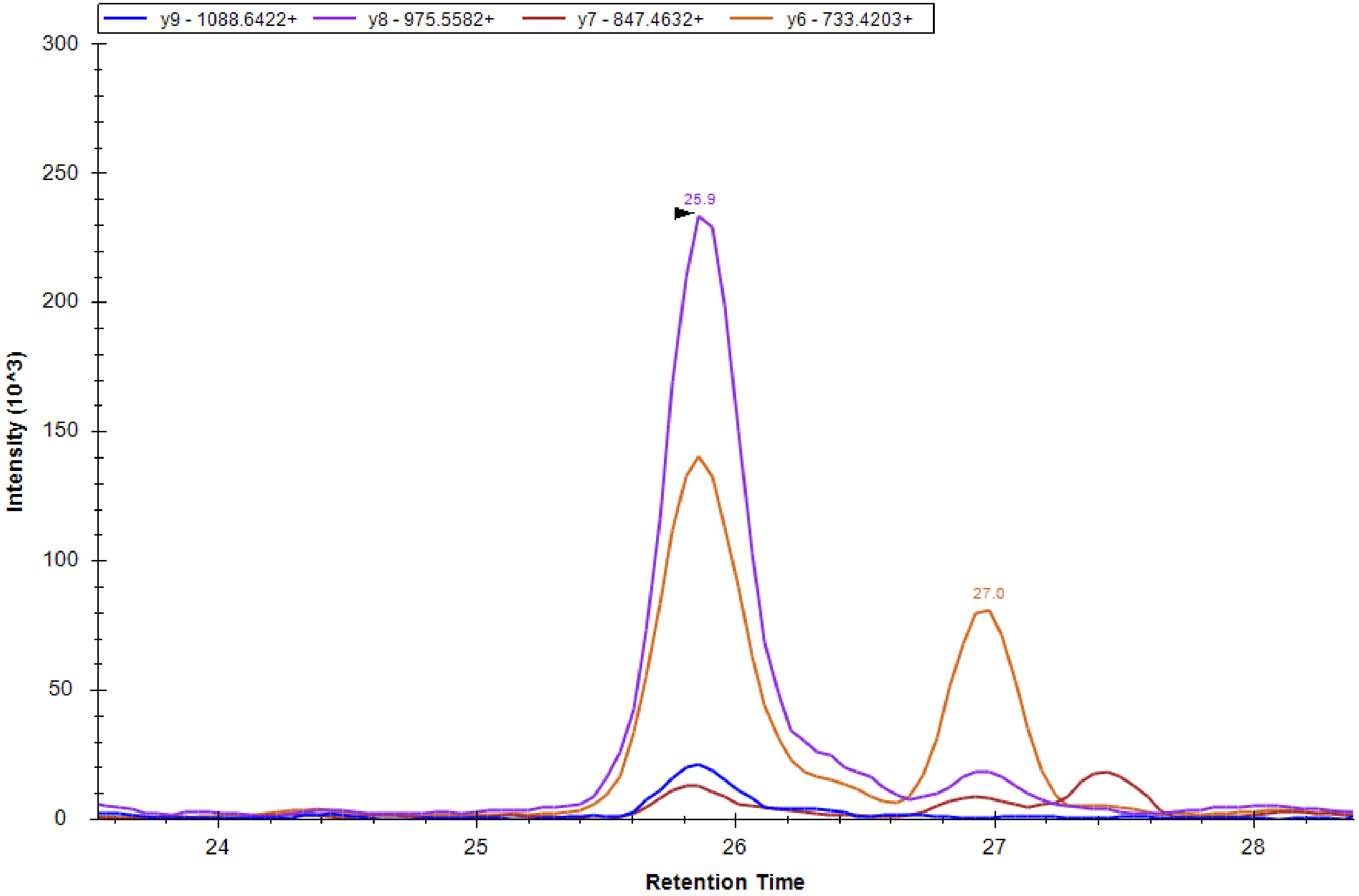

File name: 1604_JinanU(GZ)_MRM_#A_Full-Screen_0421.skyd

Parent ion m/z and charges: 594.3590++

NR_024157.2.7
VLKNRDTITK

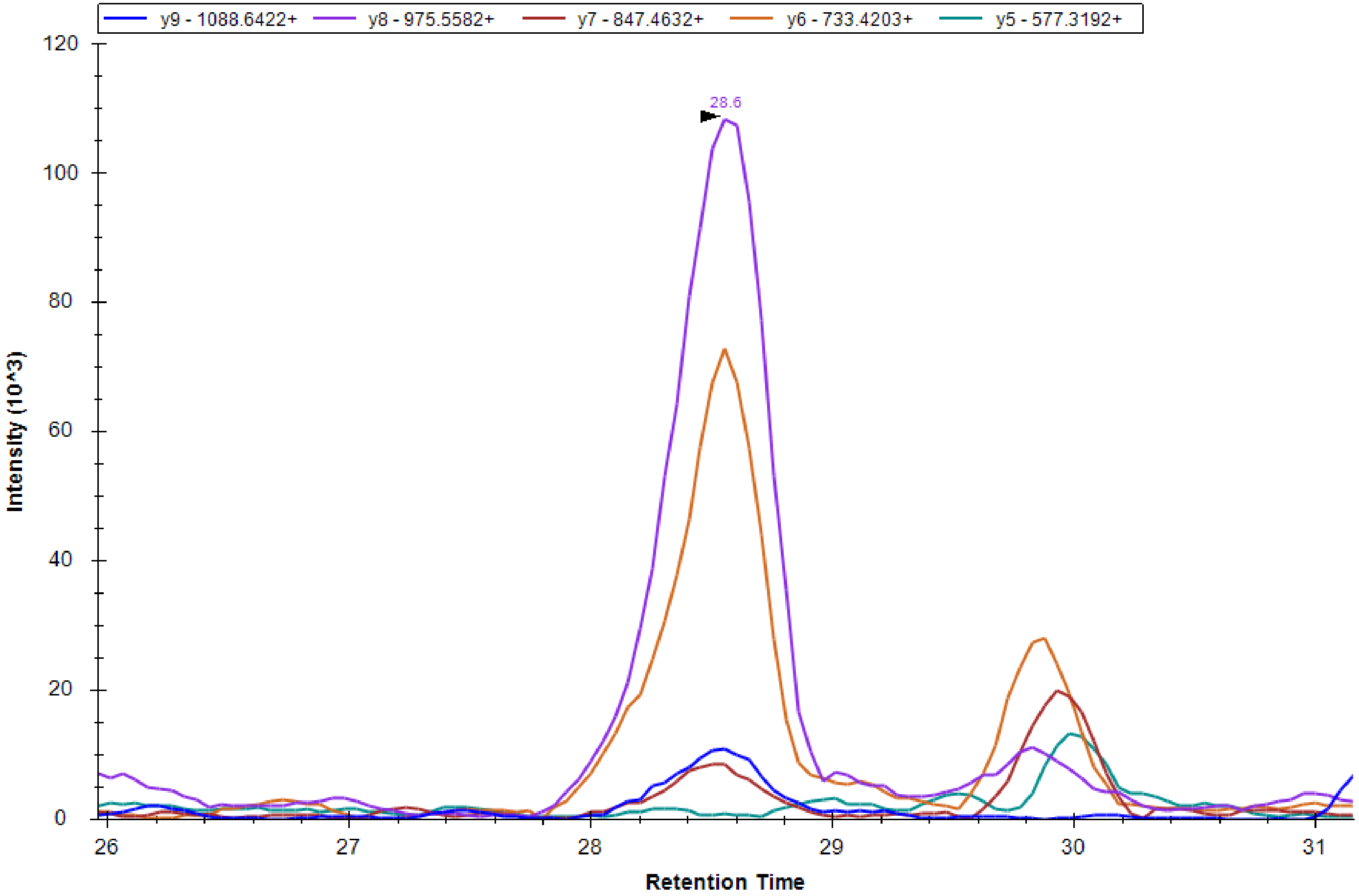

File name: 1604_JinanU(GZ)_MRM_#B_Full-Screen_0421.skyd

Parent ion m/z and charges: 594.3590++

NR_024157.2.7
VLKNRDTITK

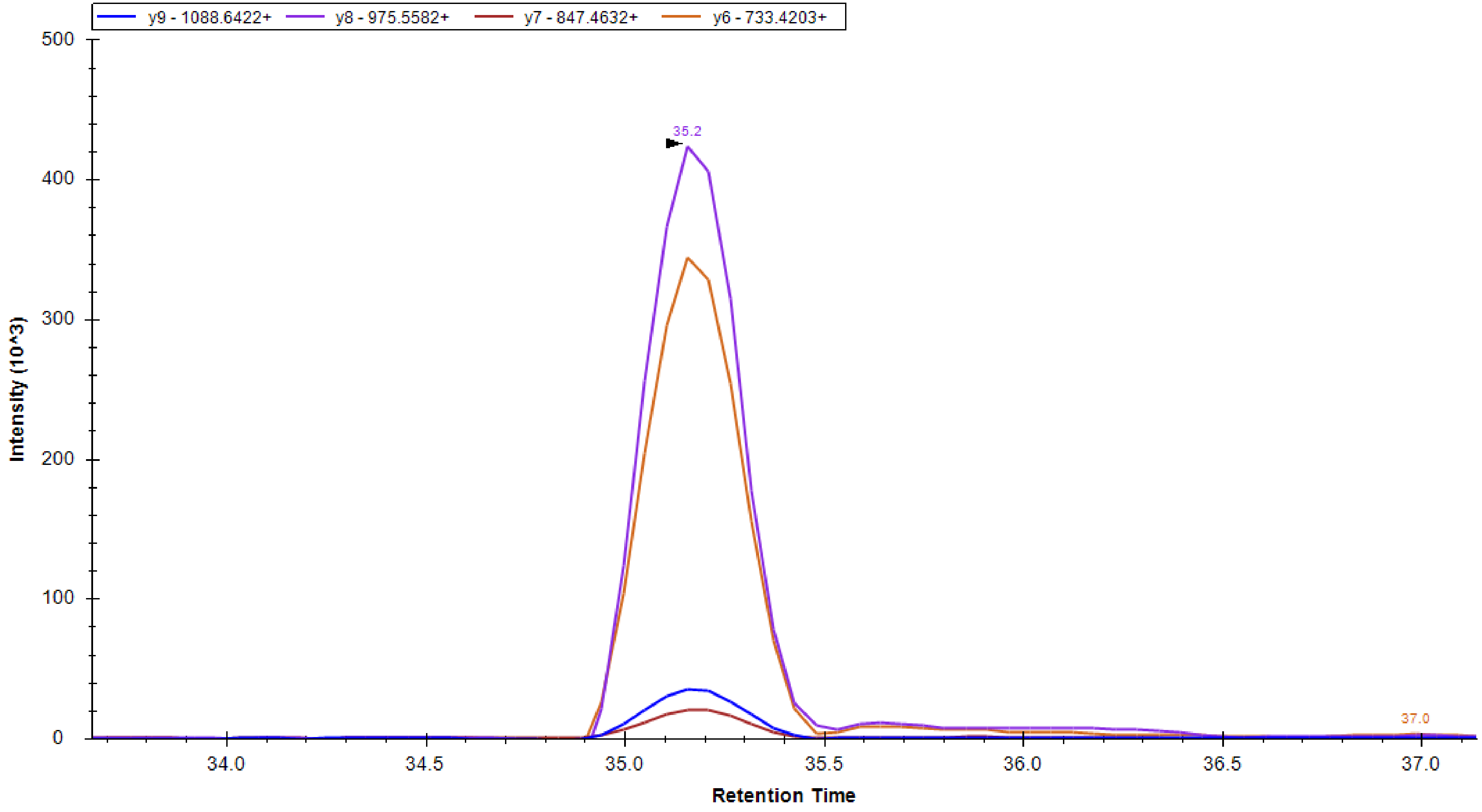

File name: 1604_JinanU(GZ)_MRM_#B_2nd_Round_Screen.skyd

Parent ion m/z and charges: 594.3590++

NR_024207.2.4
SNGWGRSLGRRGR

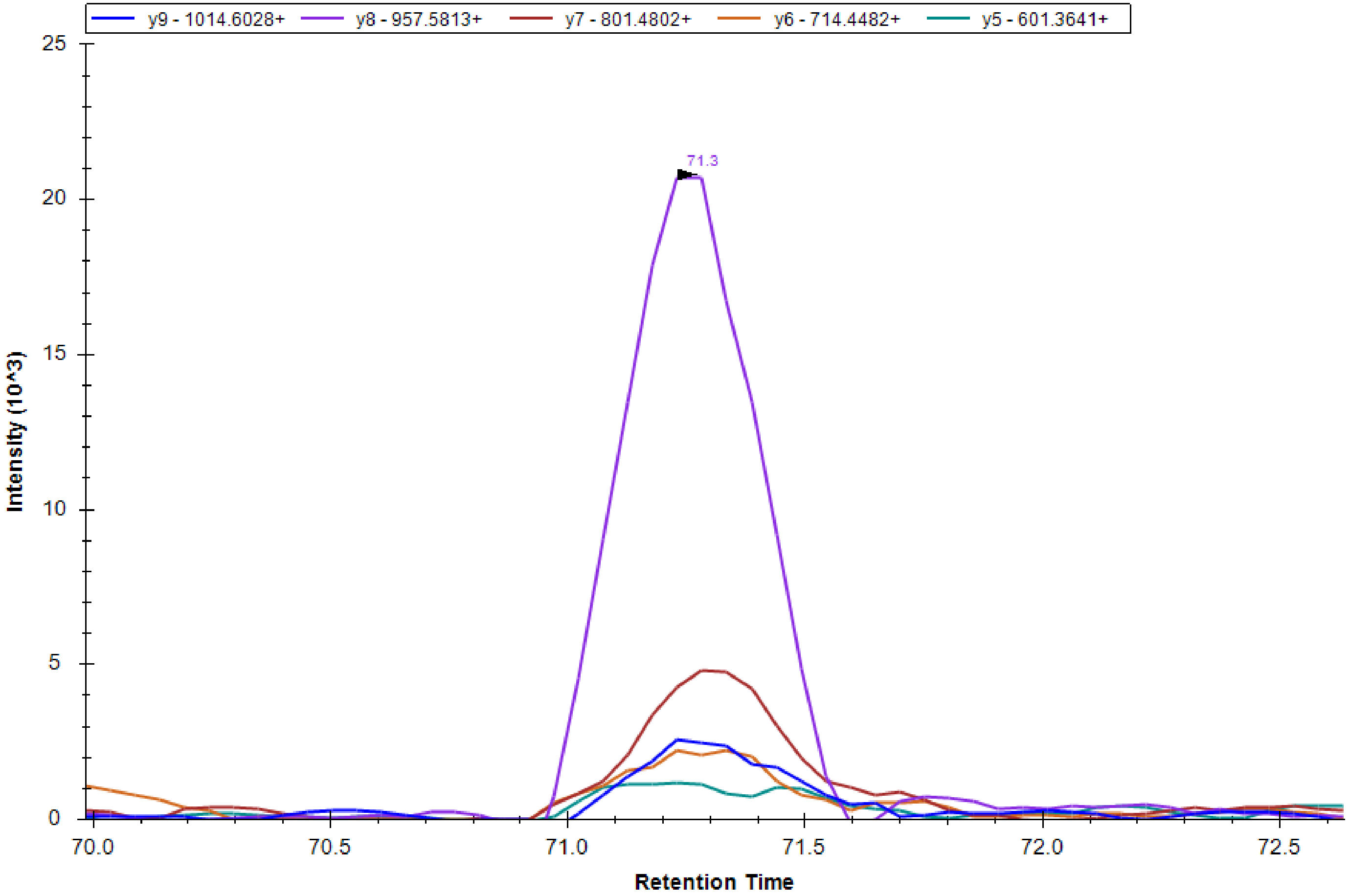

File name: 1604_JinanU(GZ)_MRM_#A_Full-Screen_0421.skyd

Parent ion m/z and charges: 729.8929++

NR_024207.2.4
KTRSWTGPWLP

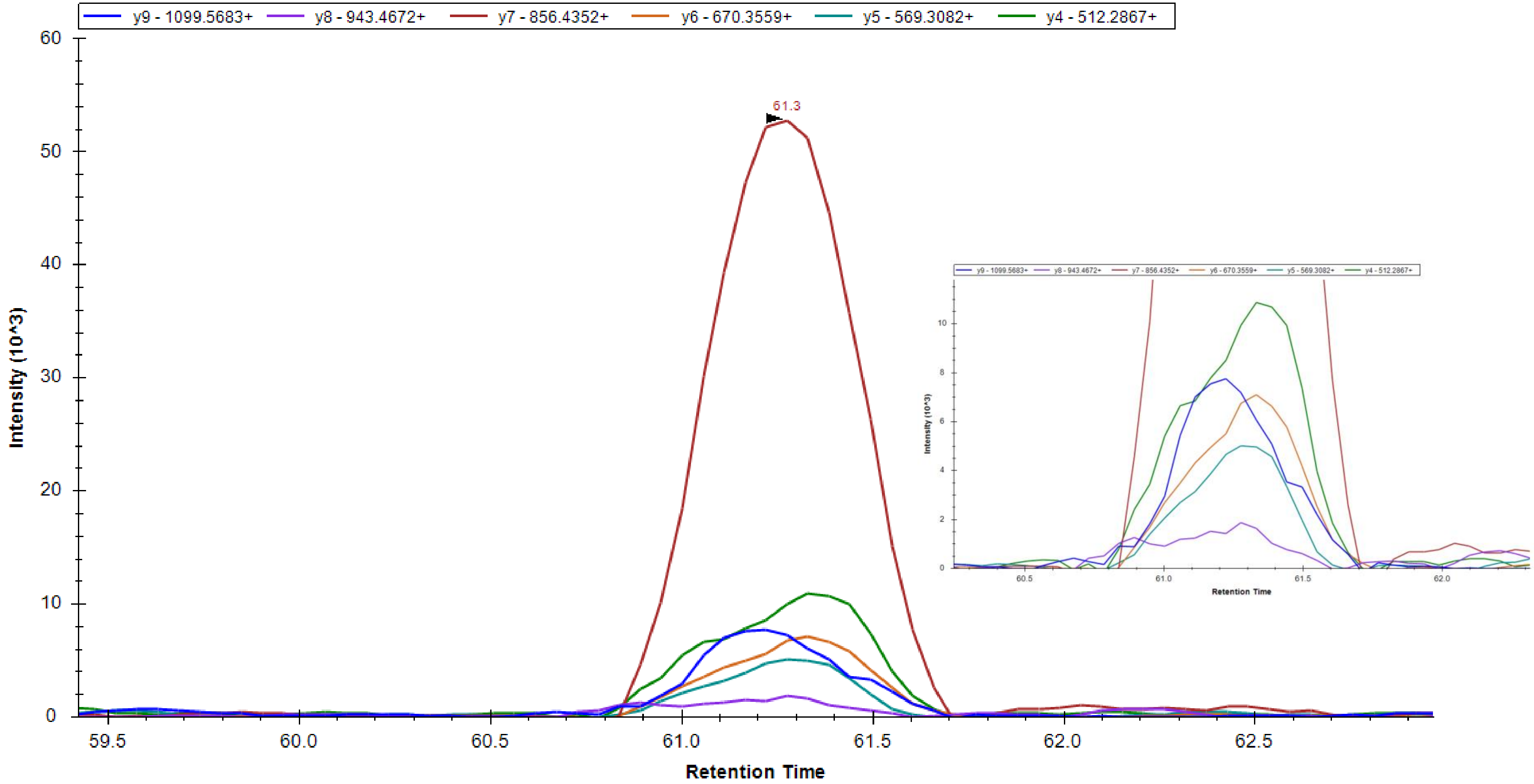

File name: 1604_JinanU(GZ)_MRM_#B_2nd_Round_Screen.skyd

Parent ion m/z and charges: 664.8591++

NR_024396.1.1
MEALPEACQNPSLTTK

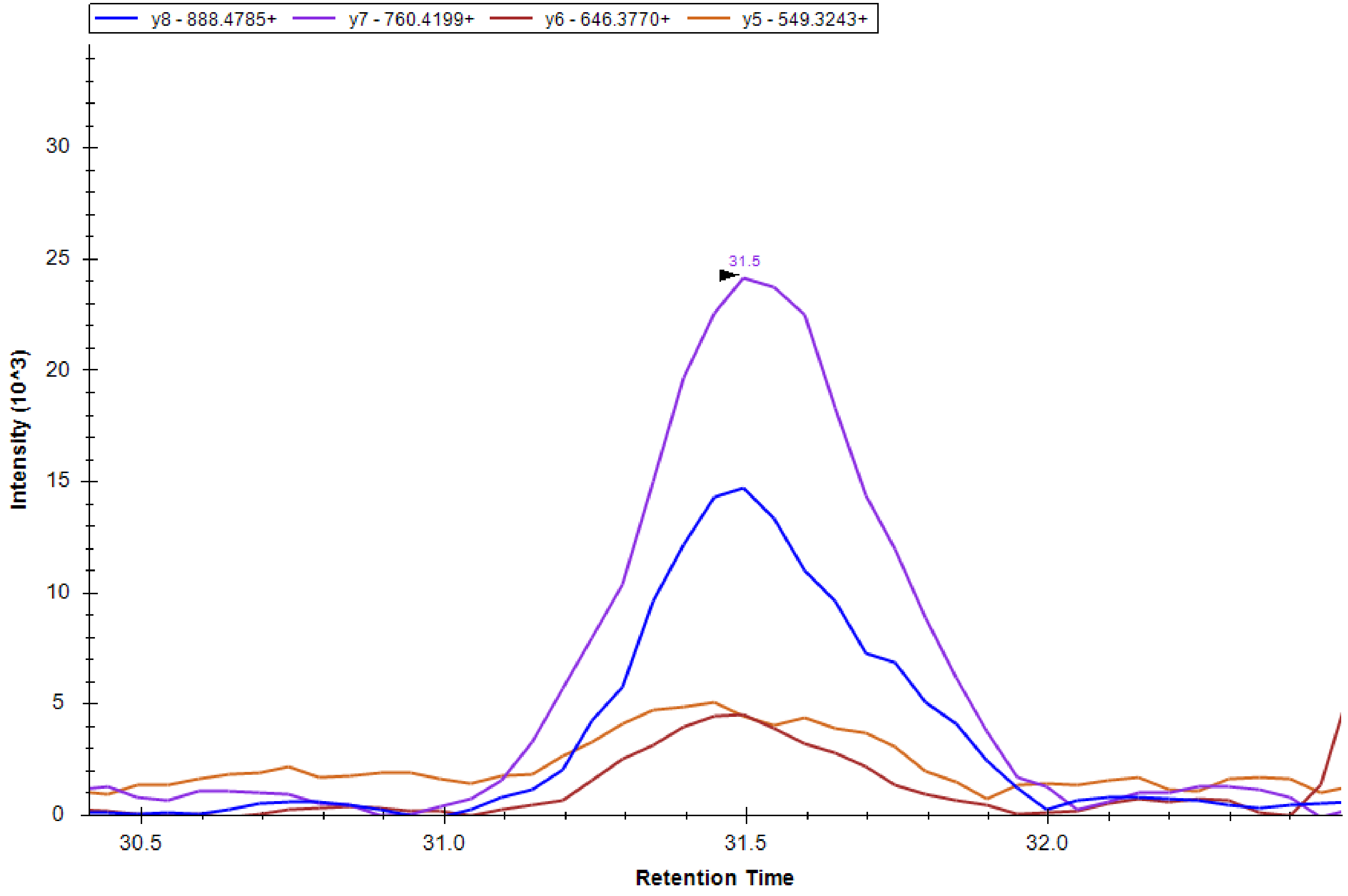

File name: 1604_JinanU(GZ)_MRM_#A_Full-Screen_0421.skyd

Parent ion m/z and charges: 895.4266++

NR_024396.1.1
RQTPGRGLPSGK

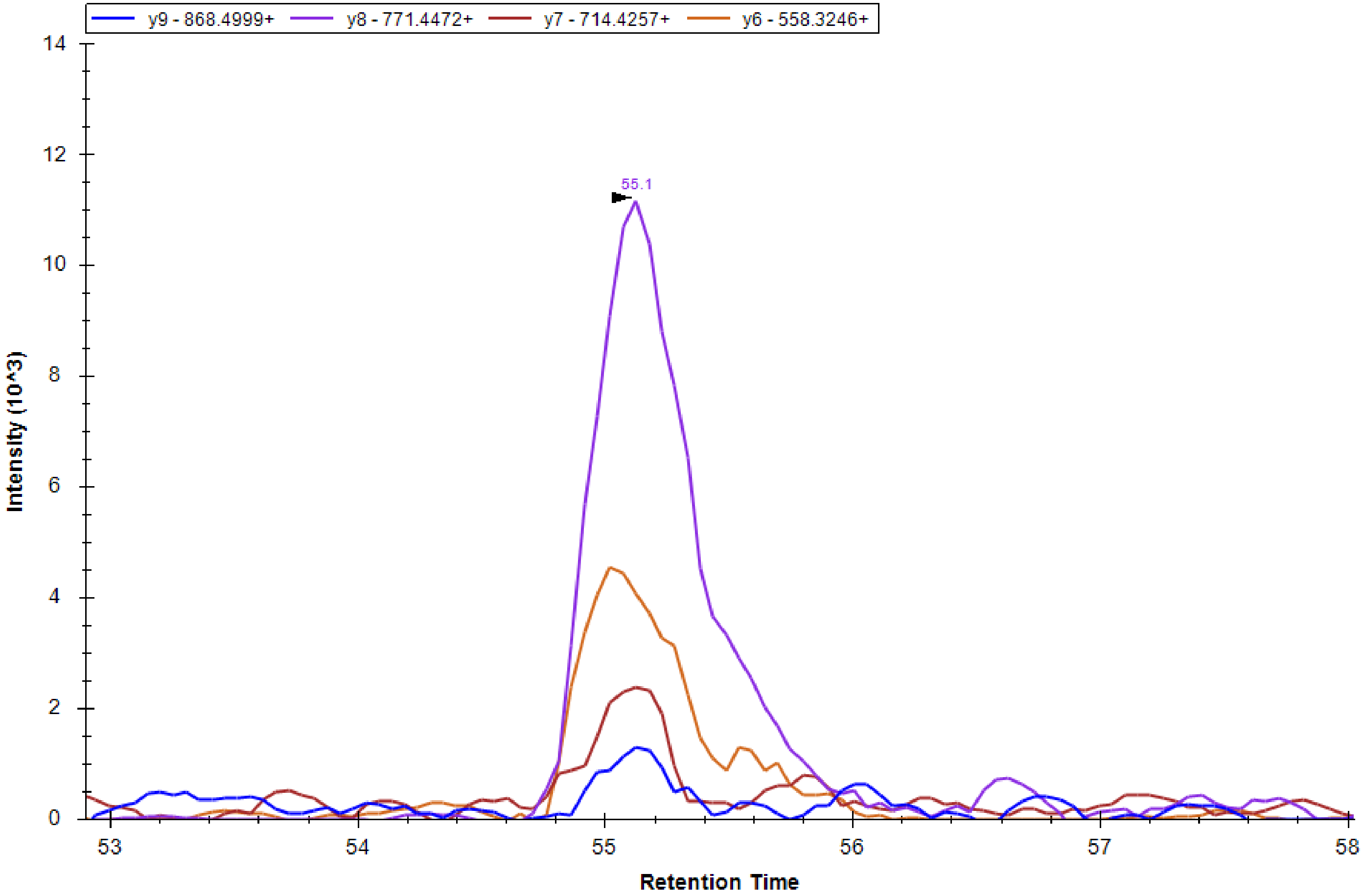

File name: 1604_JinanU(GZ)_MRM_#B_Full-Screen_0421.skyd

Parent ion m/z and charges: 627.3573++

NR_024396.1.1
MEALPEA**C**QNPSLTTK

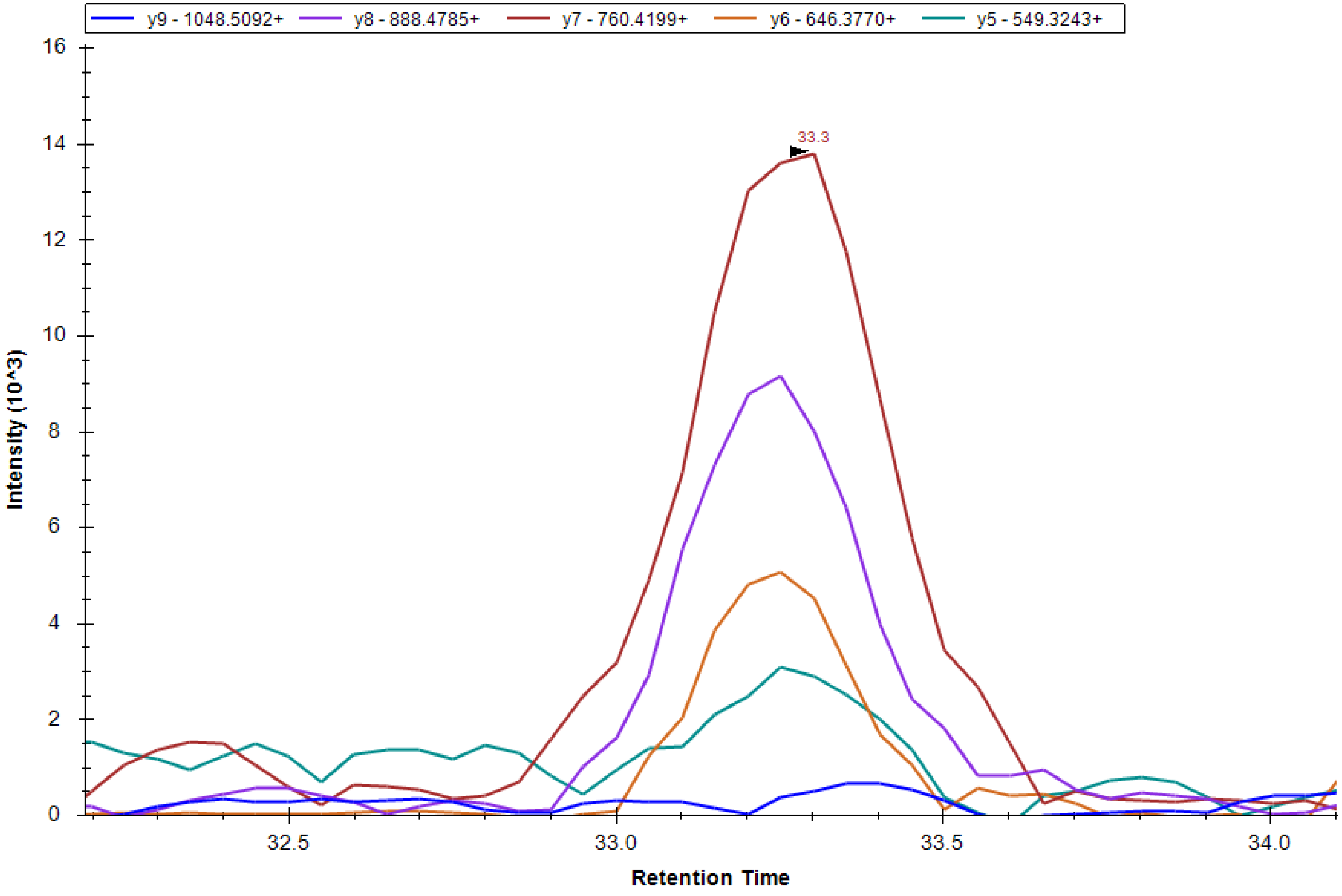

File name: 1604_JinanU(GZ)_MRM_#B_Full-Screen_0421.skyd

Parent ion m/z and charges: 895.4266++

NR_024456.3.4
KEQKGMSSMAAGK

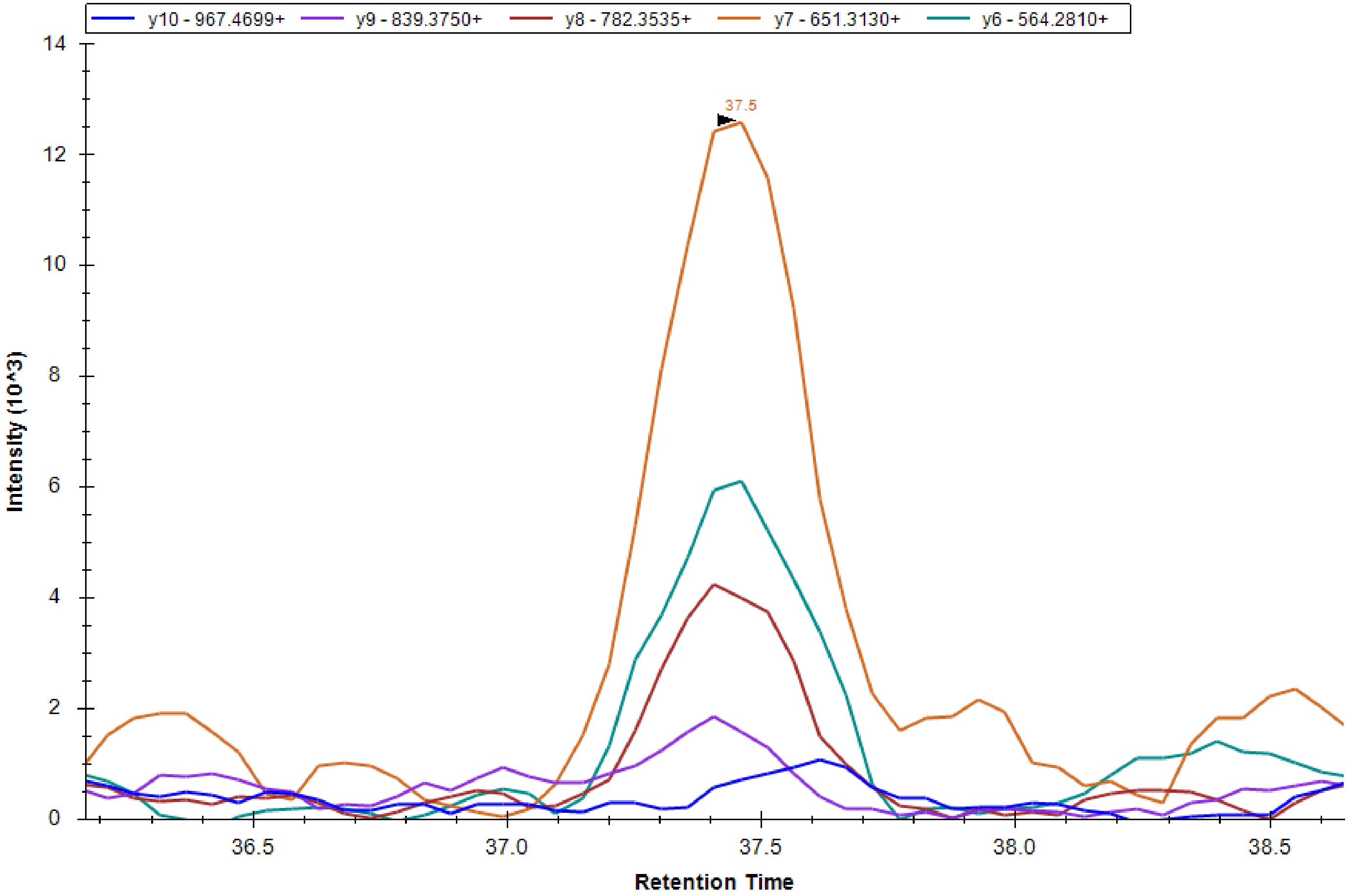

File name: 1604_JinanU(GZ)_MRM_#A_Full-Screen_0421.skyd

Parent ion m/z and charges: 676.8367++

NR_026762.3.1
MPISPAANSAPEIR

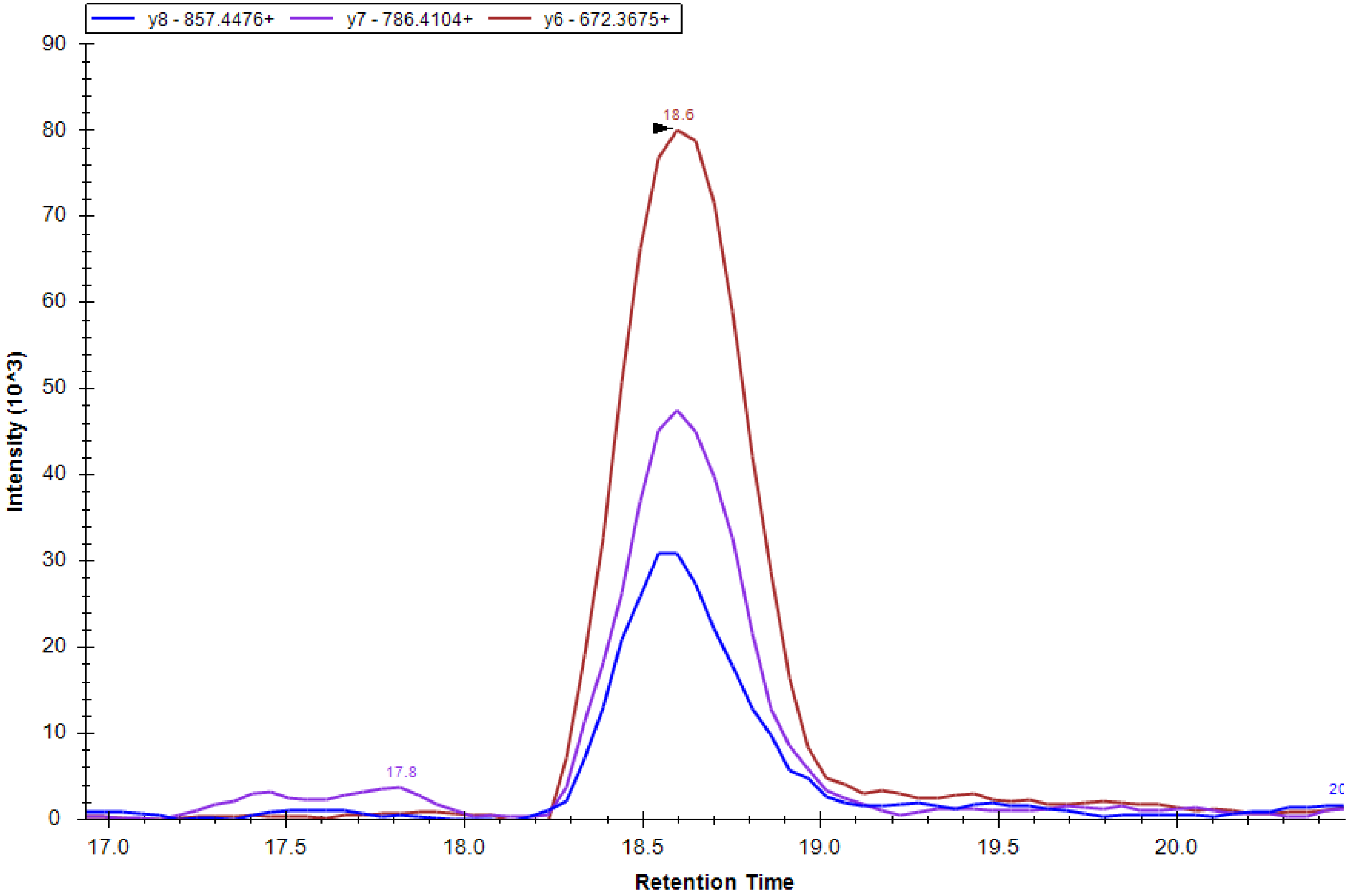

File name: 1604_JinanU(GZ)_MRM_#A_Full-Screen_0421.skyd

Parent ion m/z and charges: 727.3770++

NR_026762.3.1
RVEIAFPARR

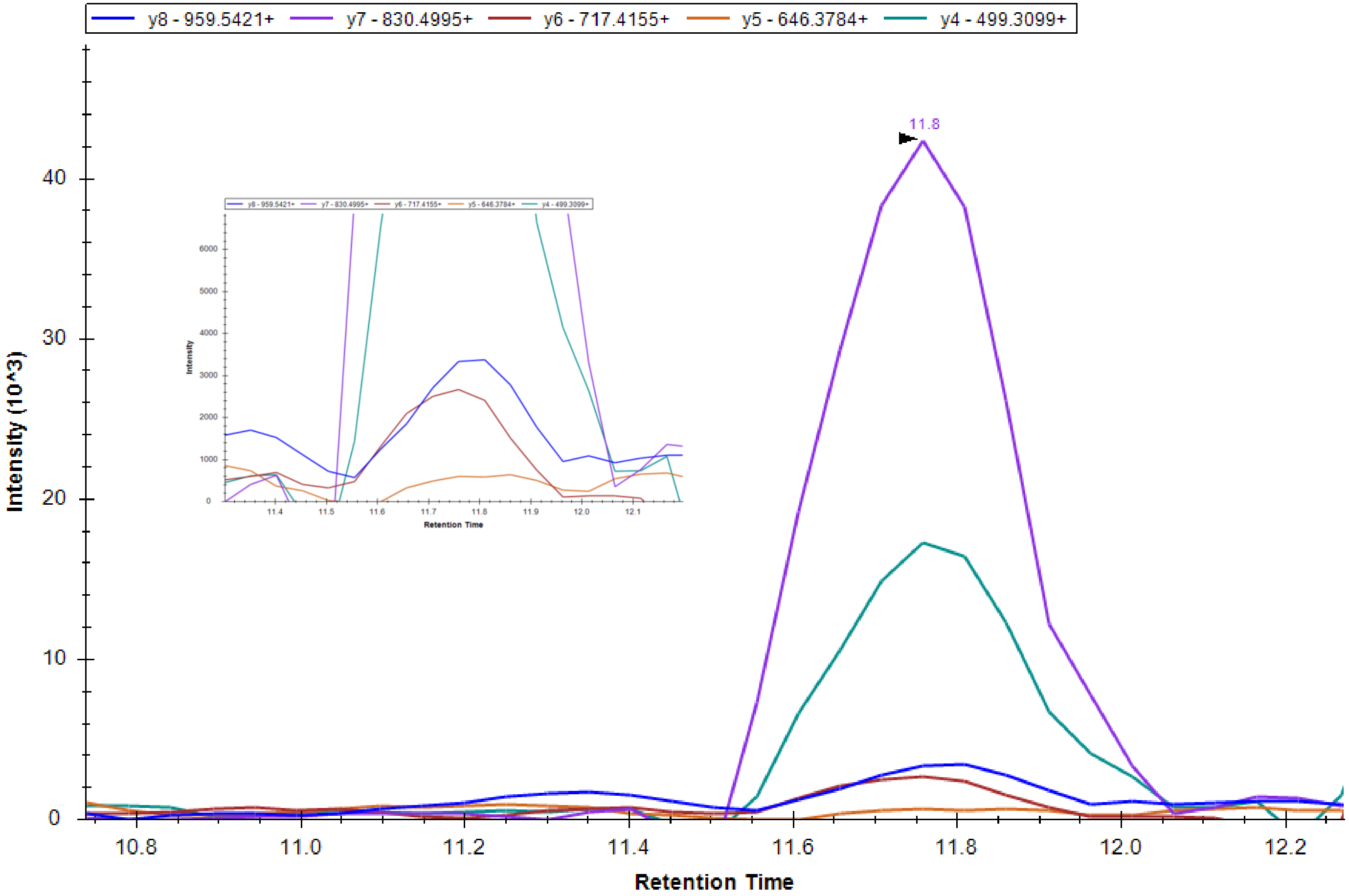

File name: 1604_JinanU(GZ)_MRM_#B_Full-Screen_0421.skyd

Parent ion m/z and charges: 607.8595++

NR_026762.3.1
MPISPAANSAPEIR

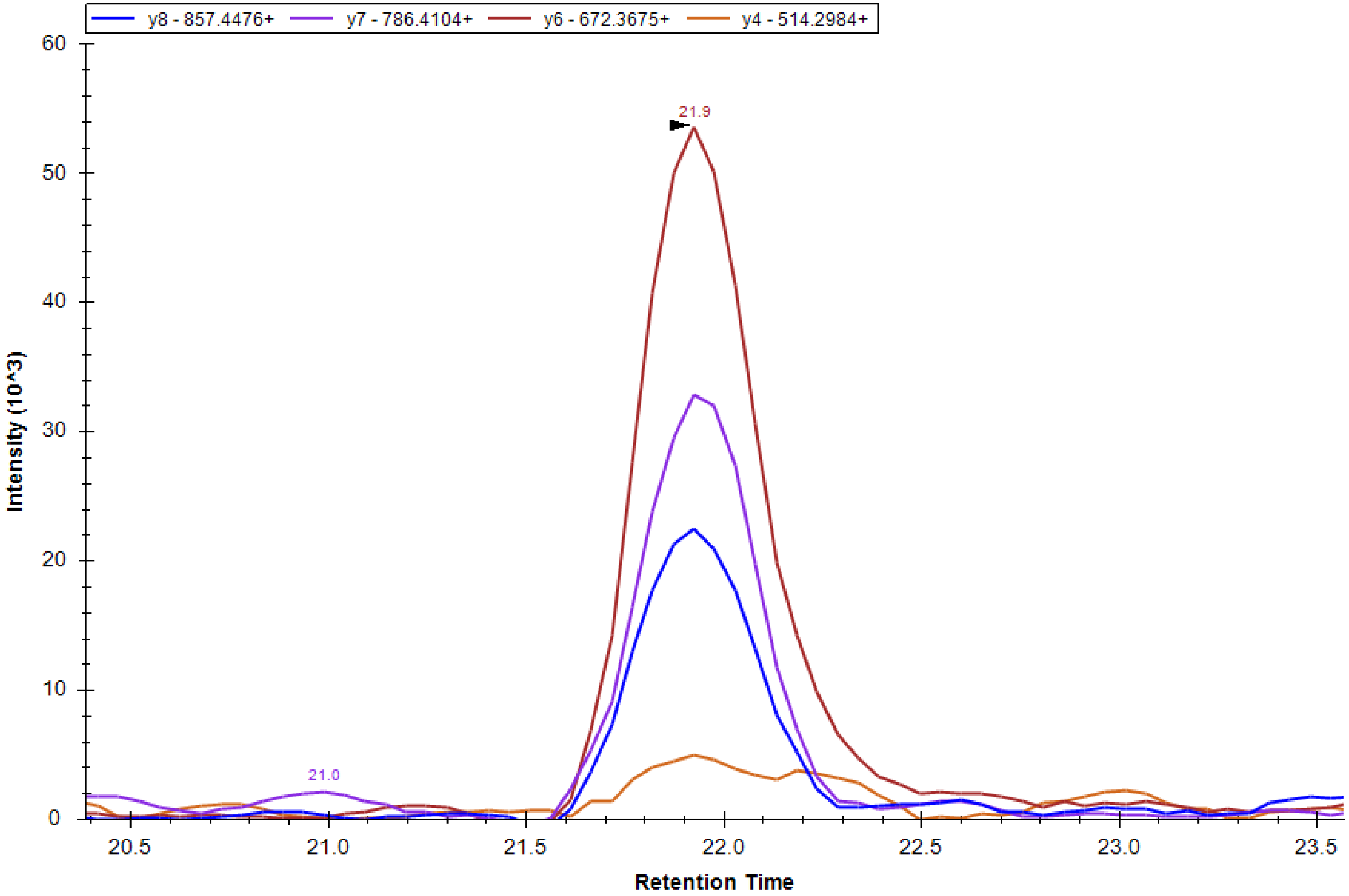

File name: 1604_JinanU(GZ)_MRM_#B_Full-Screen_0421.skyd

Parent ion m/z and charges: 727.3770++

NR_026762.3.1
MPISPAANSAPEIR

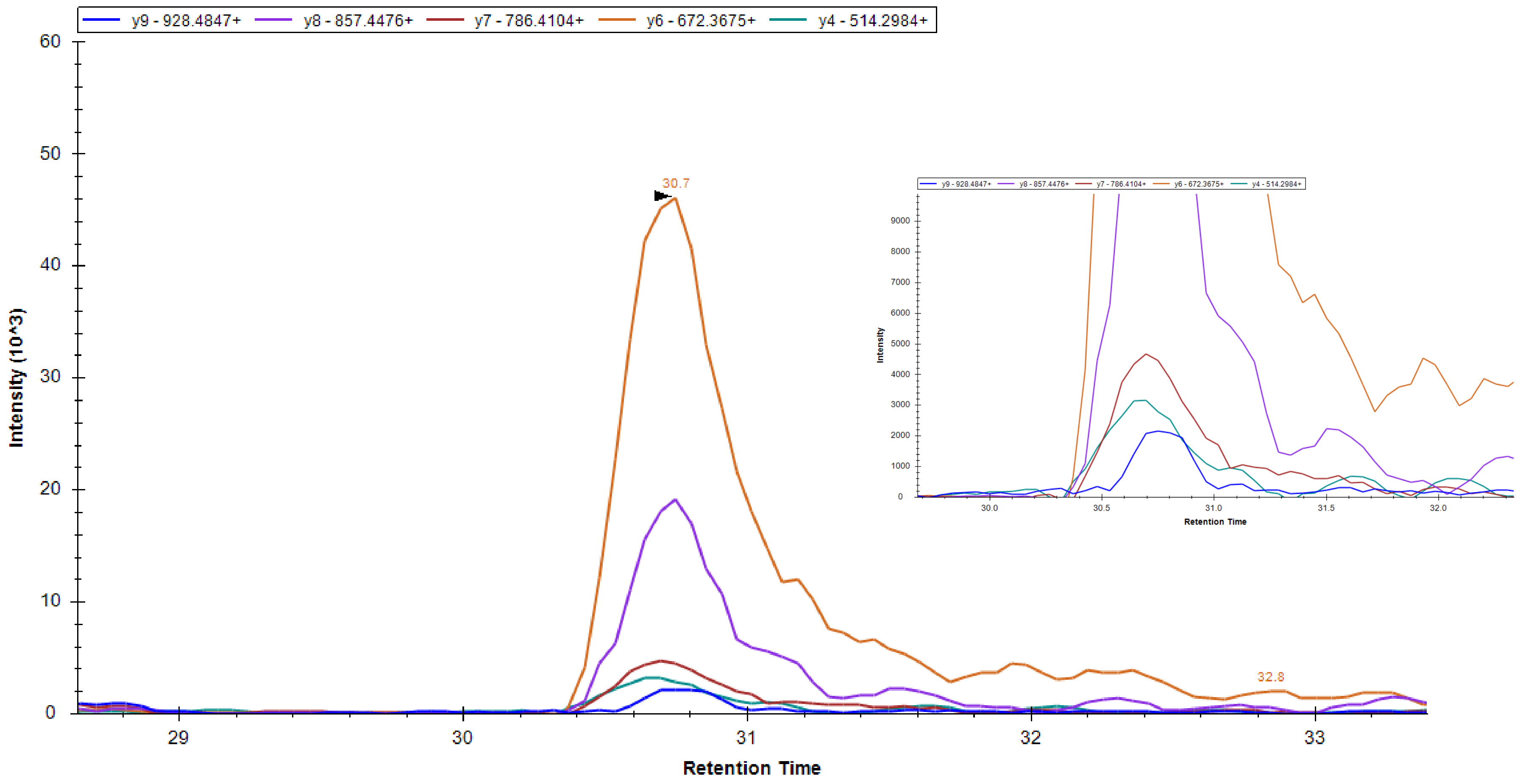

File name: 1604_JinanU(GZ)_MRM_#B_2nd_Round_Screen.skyd

Parent ion m/z and charges: 727.3770++

NR_026961.2.9
MTIIGICPR

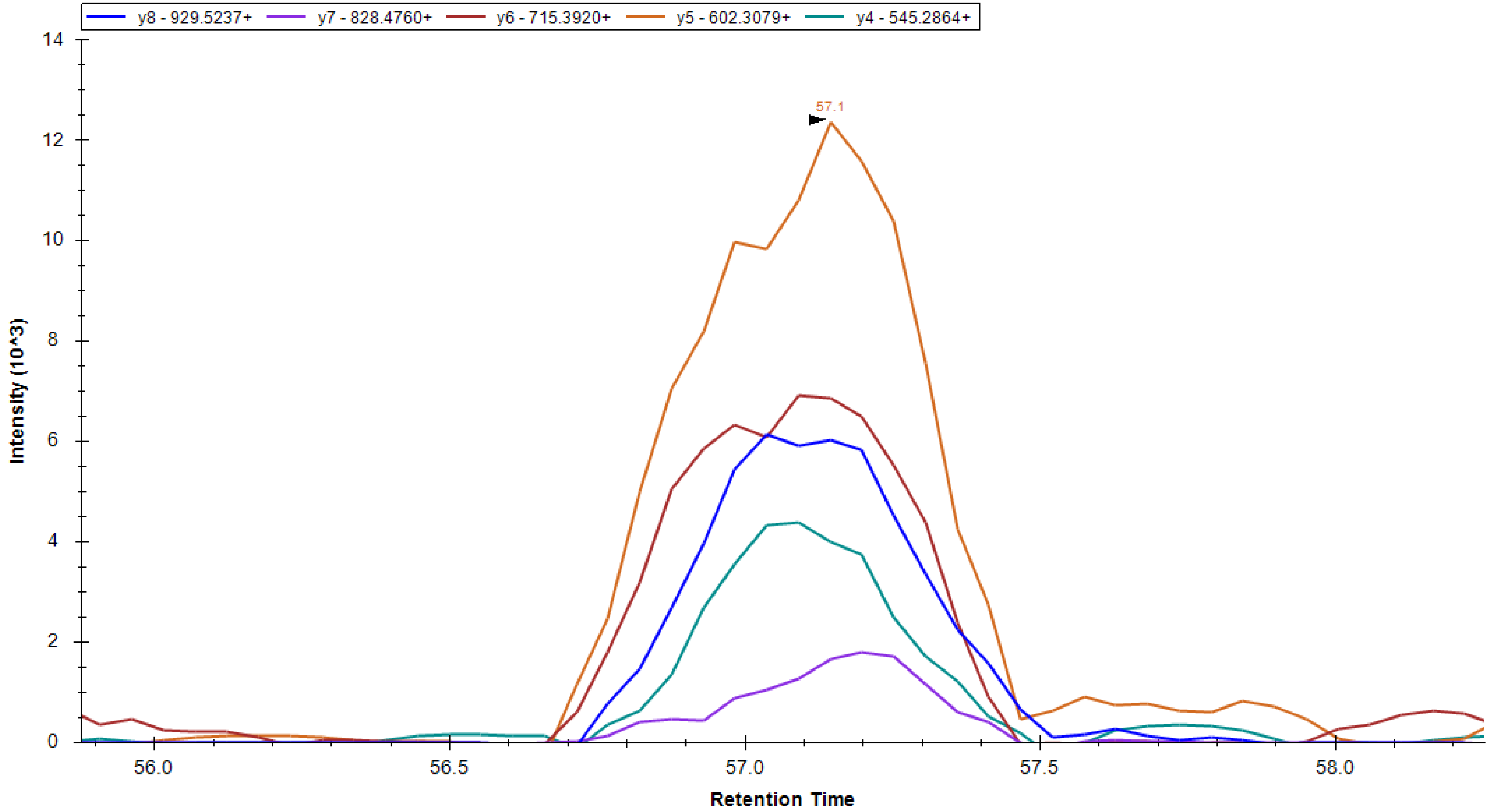

File name: 1604_JinanU(GZ)_MRM_#B_2nd_Round_Screen.skyd

Parent ion m/z and charges: 530.7857++

NR_027130.3.25
DINICLHLNTR

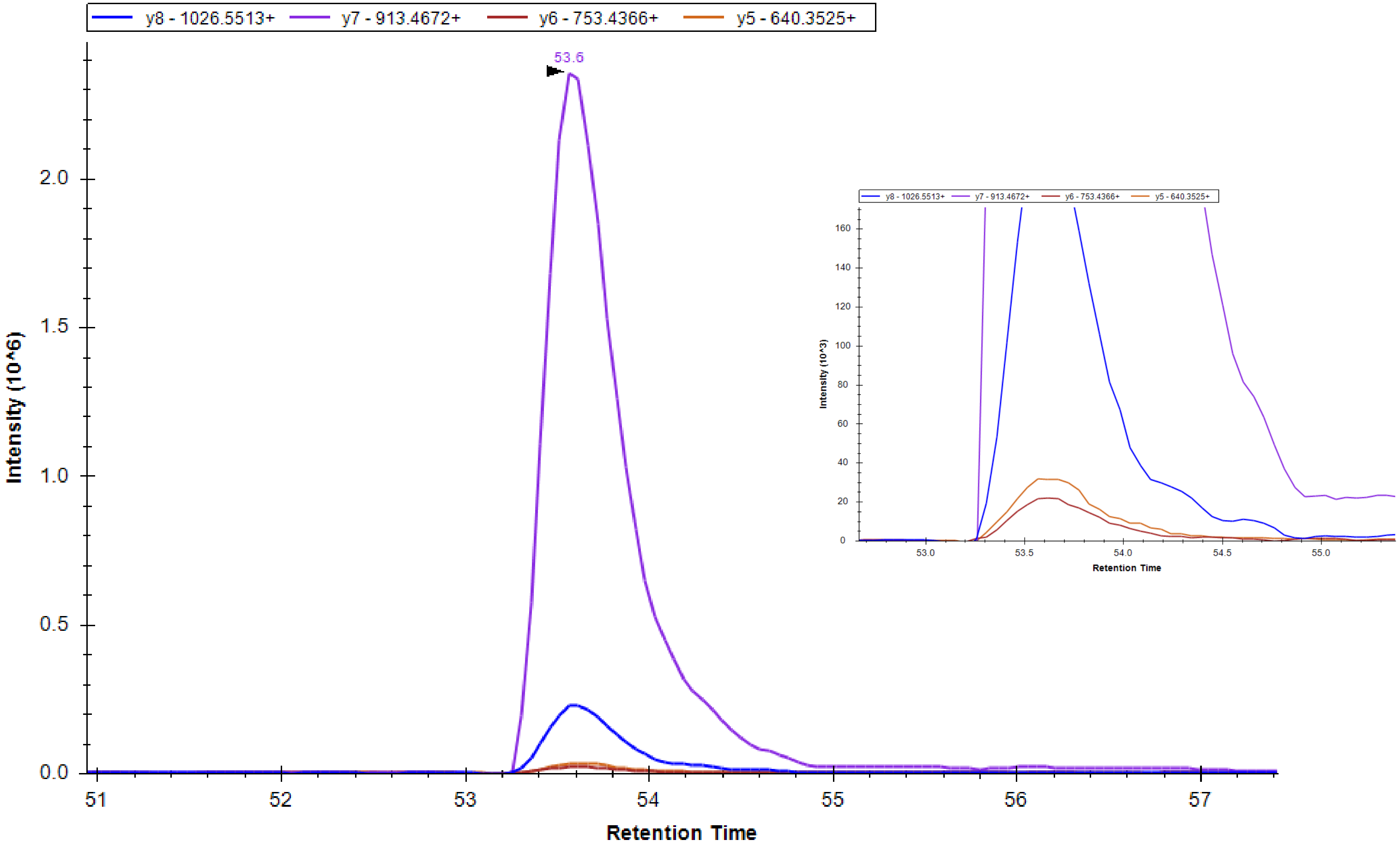

File name: 1604_JinanU(GZ)_MRM_#A_Full-Screen_0421.skyd

Parent ion m/z and charges: 684.8563++

NR_027130.3.25
DINICLHLNTR

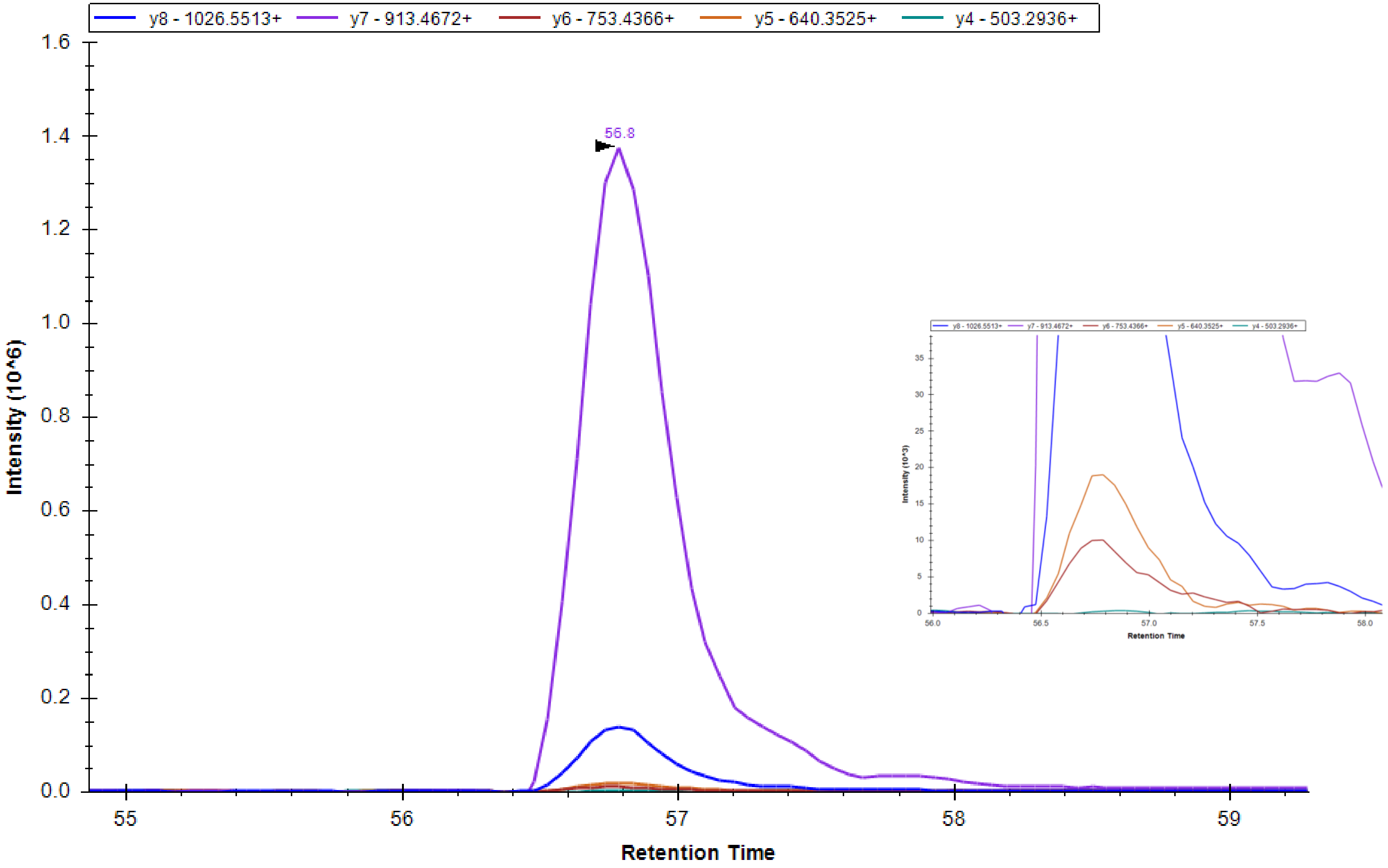

File name: 1604_JinanU(GZ)_MRM_#B_Full-Screen_0421.skyd

Parent ion m/z and charges: 684.8563++

NR_027130.3.25
DINI<u>C</u>LHLNTR

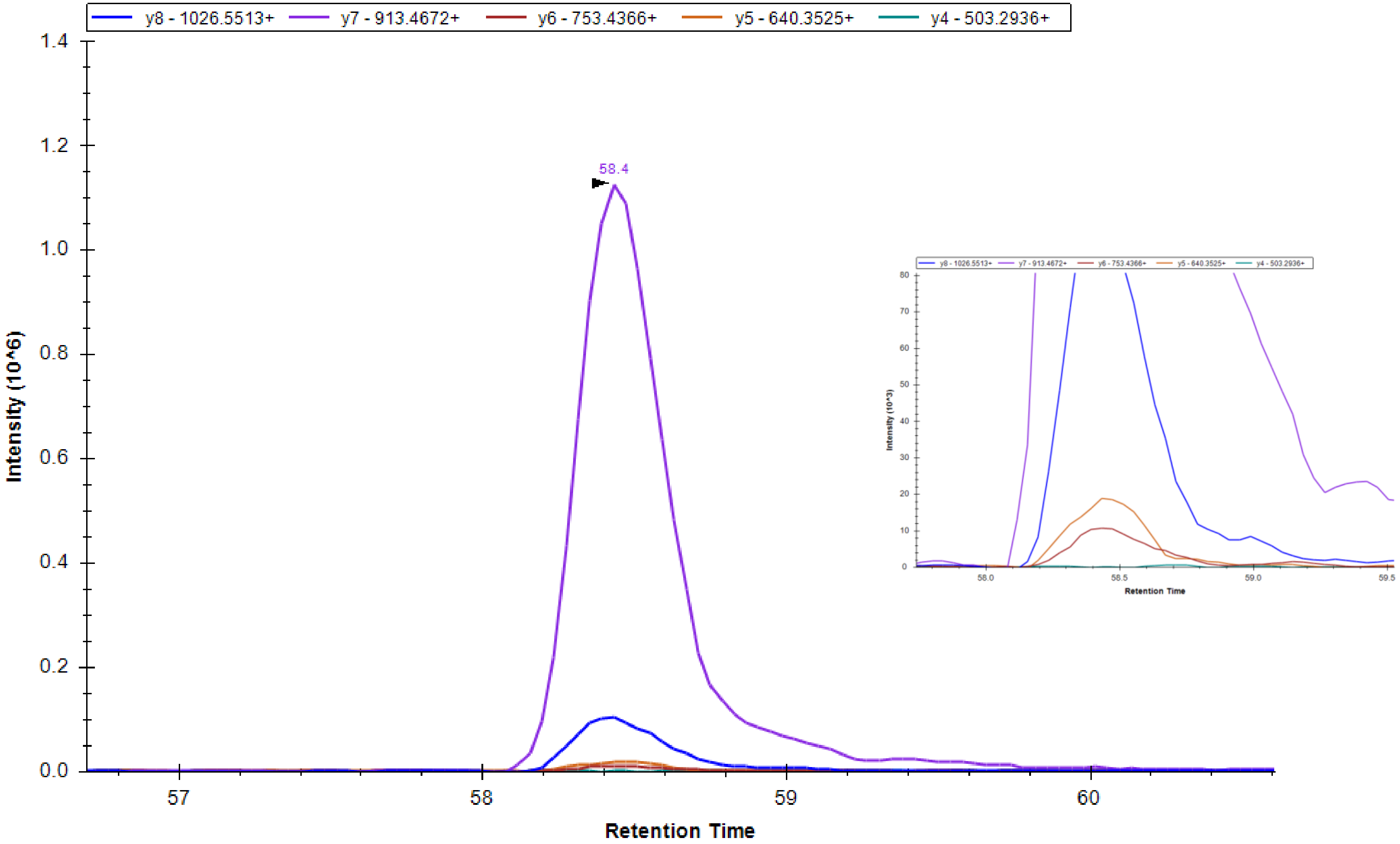

File name: 1604_JinanU(GZ)_MRM_#A_#B_Positive_Screen.skyd

Parent ion m/z and charges: 684.8563++

NR_027241.2.2
MLSVAVAEEEPPEICVGKVSALDPR

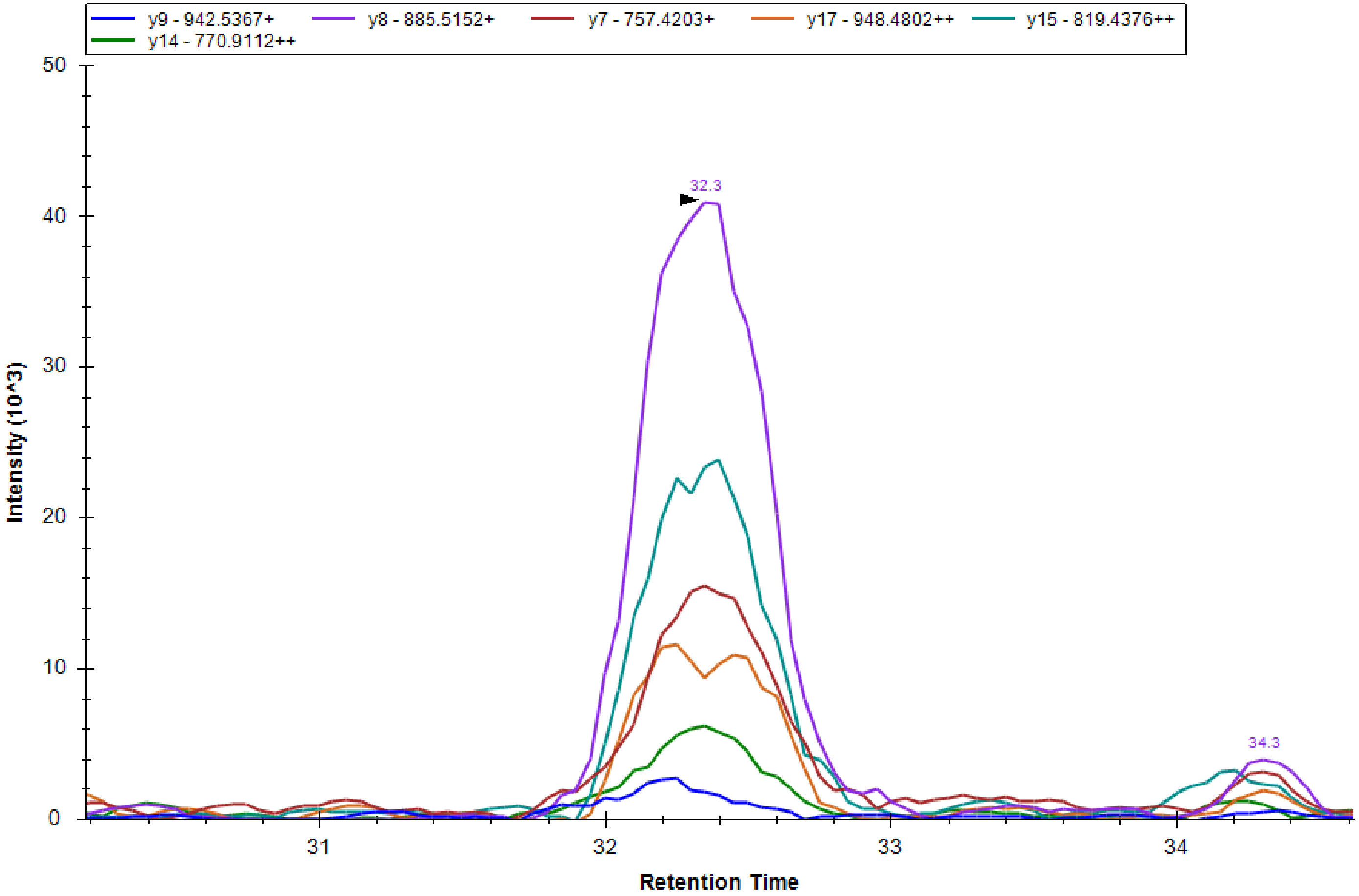

File name: 1604_JinanU(GZ)_MRM_#A_Full-Screen_0421.skyd

Parent ion m/z and charges: 899.4593+++

NR_027241.2.2
MLSVAVAEEEPPEICVGKVSALDPR

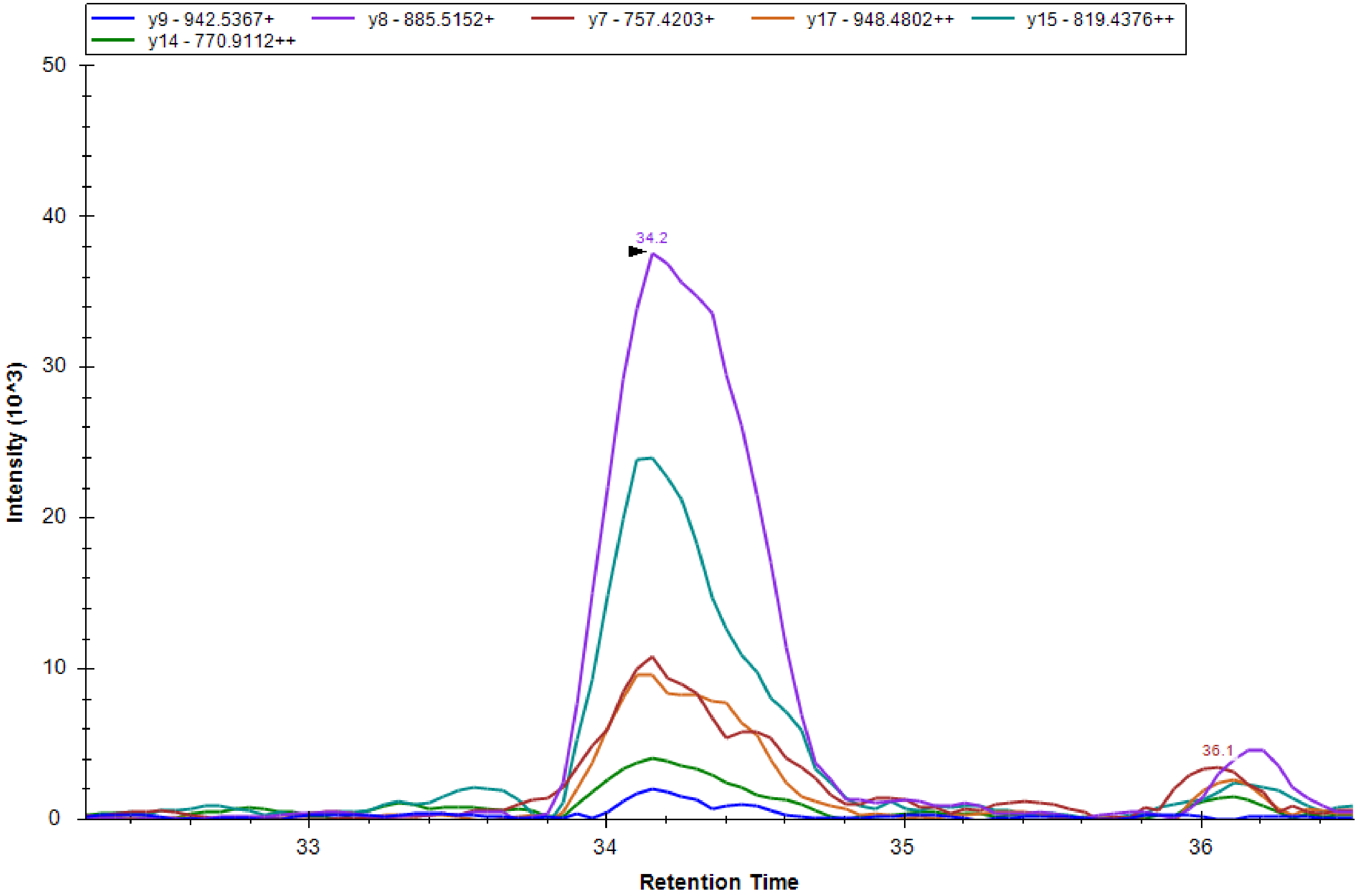

File name: 1604_JinanU(GZ)_MRM_#B_Full-Screen_0421.skyd

Parent ion m/z and charges: 899.4593+++

NR_027241.2.2
MLSVAVAEEEPPEICVGKVSALDPR

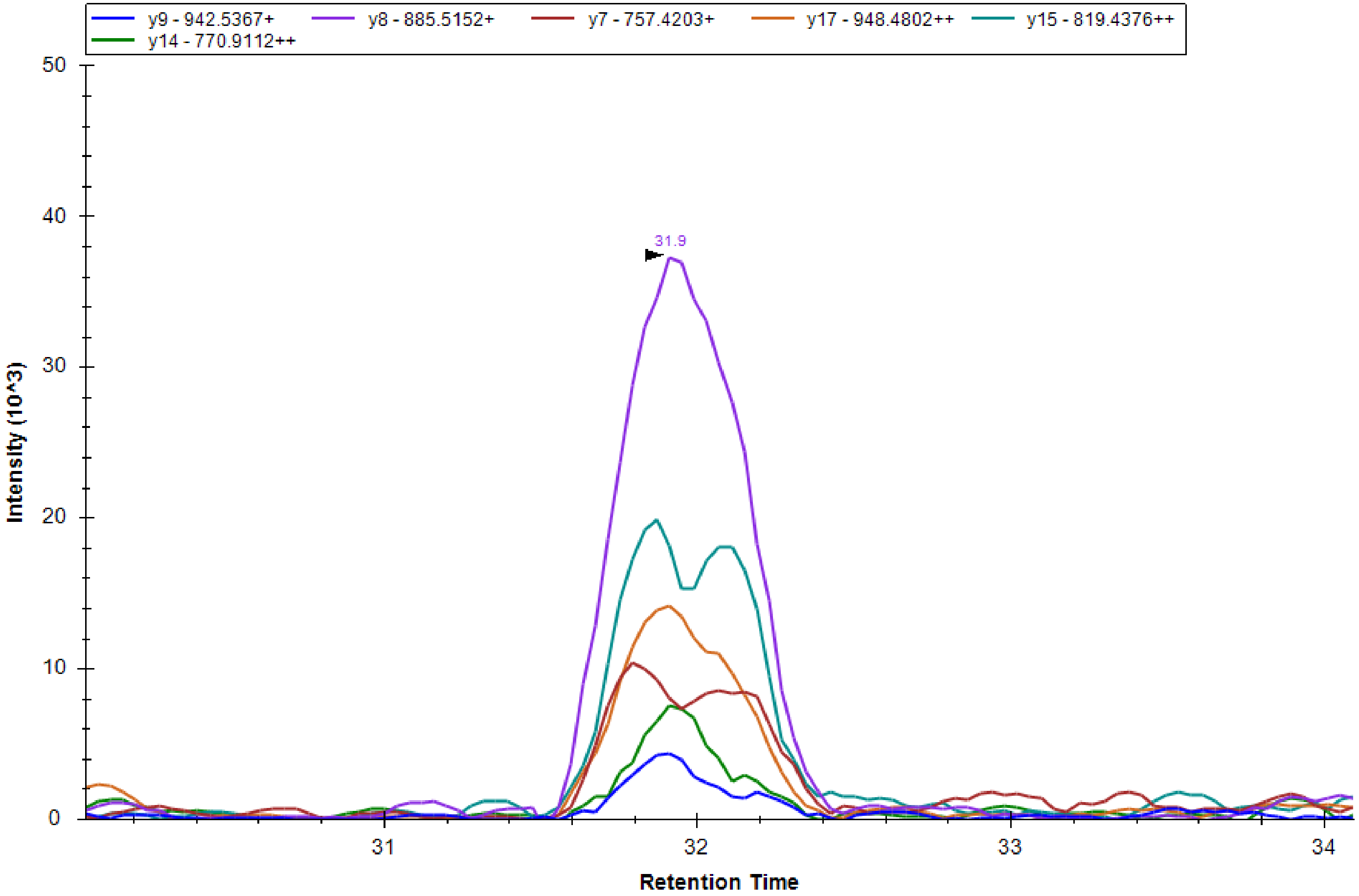

File name: 1604_JinanU(GZ)_MRM_#A_#B_Positive_Screen.skyd

Parent ion m/z and charges: 899.4593+++

NR_027433.2.2
PSALPSGVGDAMPPGPPSPTPR

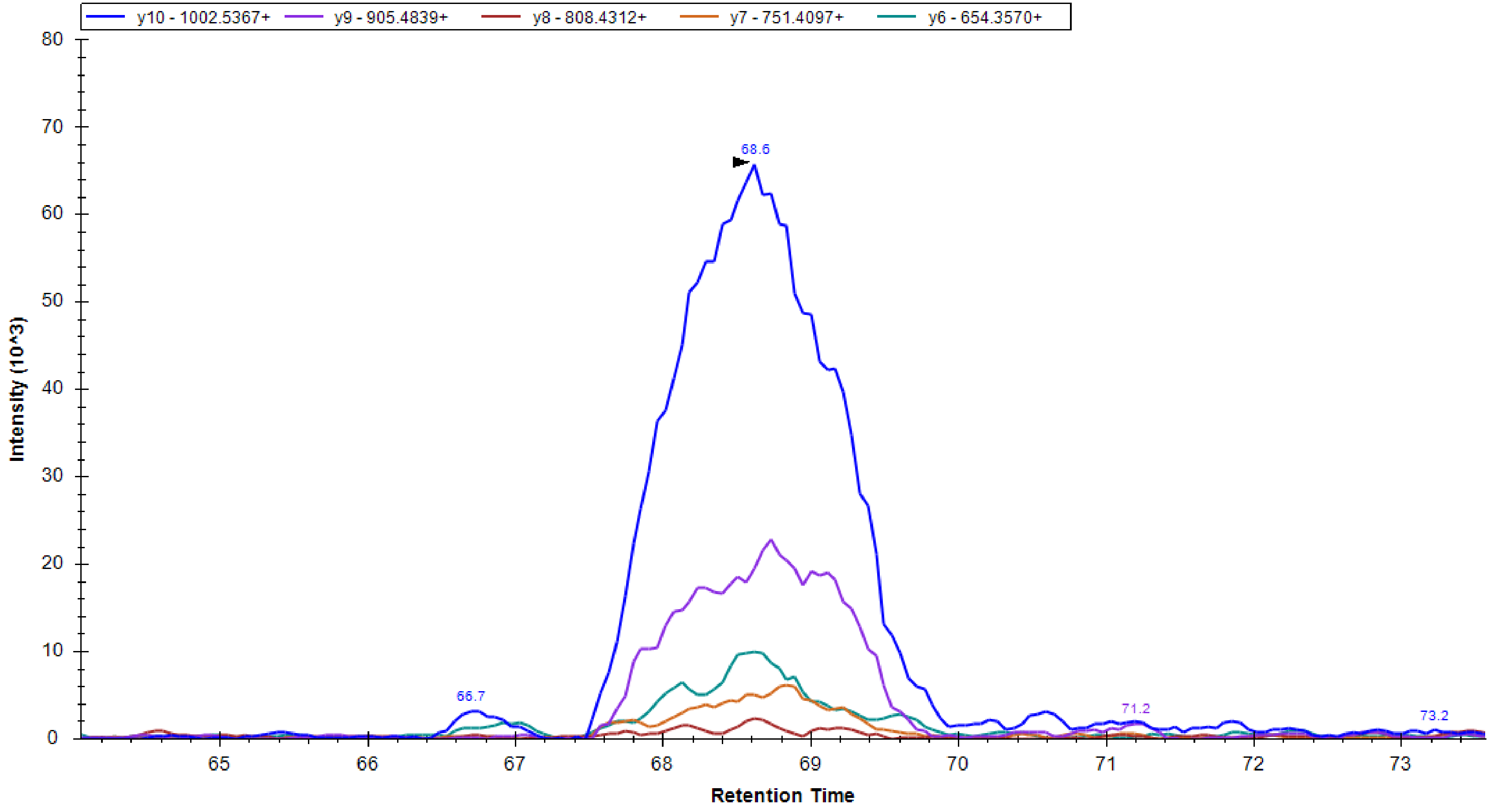

File name: 1604_JinanU(GZ)_MRM_#B_2nd_Round_Screen.skyd

Parent ion m/z and charges: 1043.0253++

NR_027433.2.2
MPSALPSGVGDAMPPGPPSPTPR

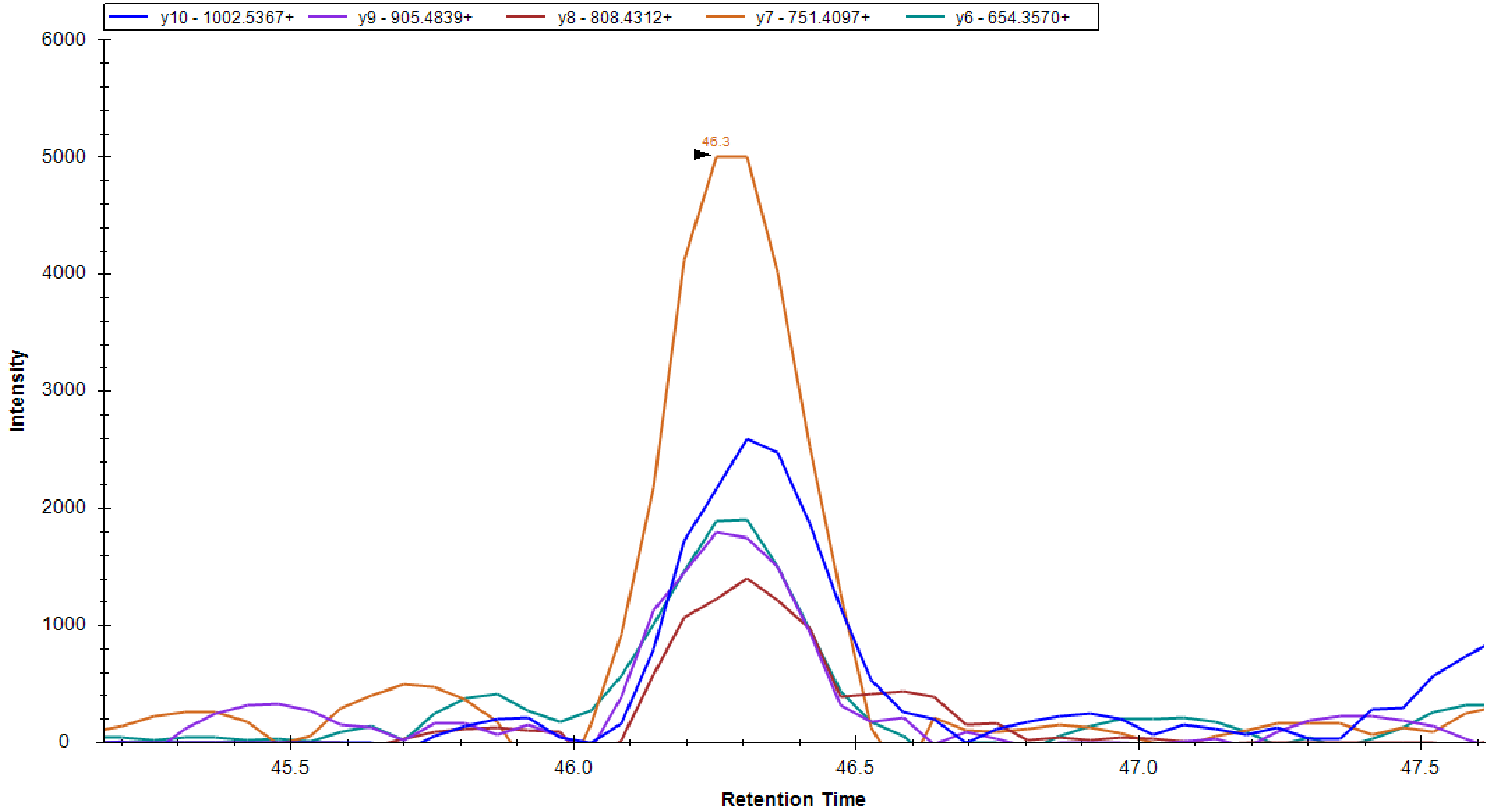

File name: 1604_JinanU(GZ)_MRM_#B_2nd_Round_Screen.skyd

Parent ion m/z and charges: 1108.5456++

NR_028350.3.3
SDAAVDTSSEIIAK

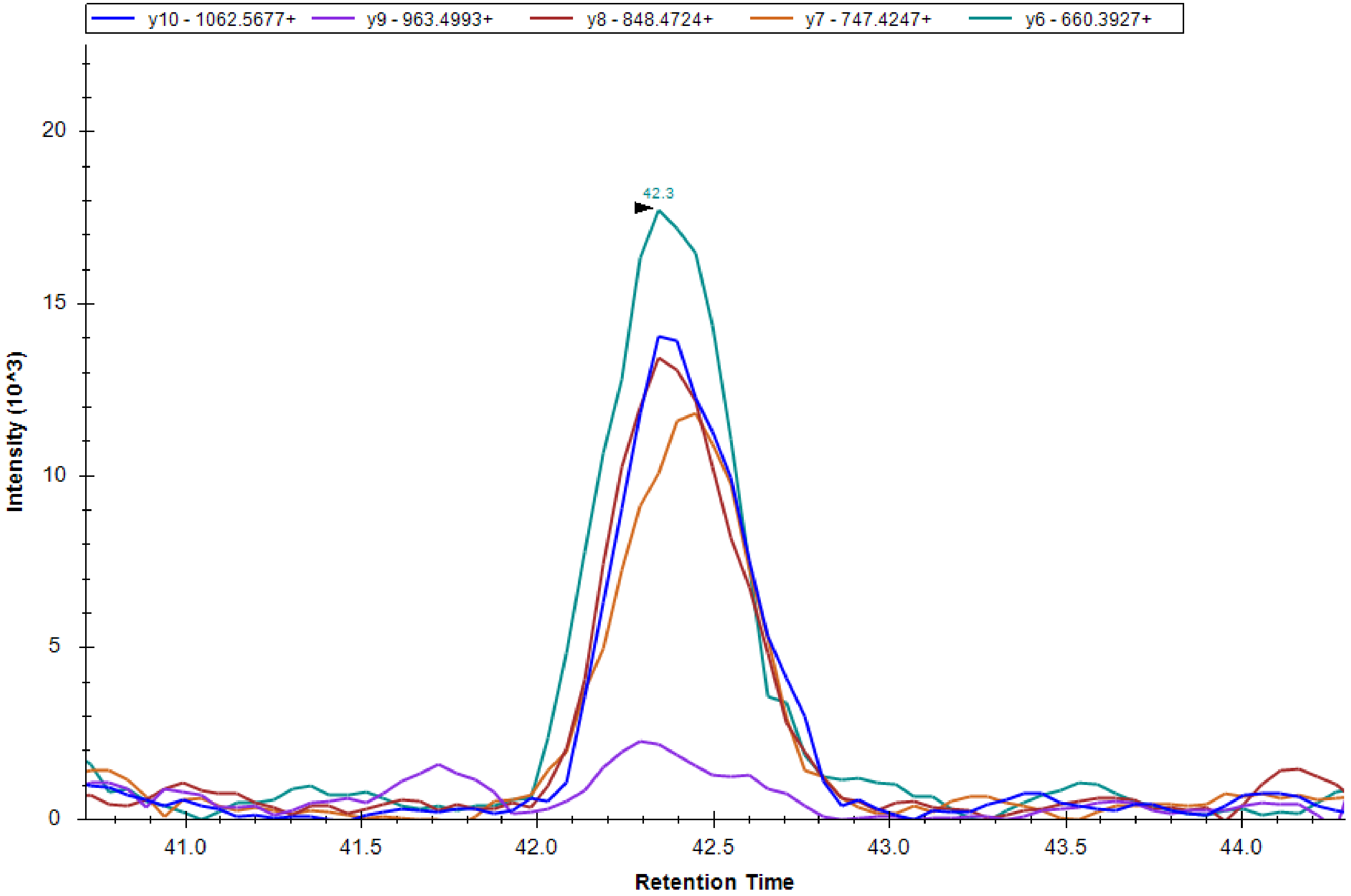

File name: 1604_JinanU(GZ)_MRM_#A_Full-Screen_0421.skyd

Parent ion m/z and charges: 703.8541++

NR_028350.3.3
SDAAVDTSSEIIAK

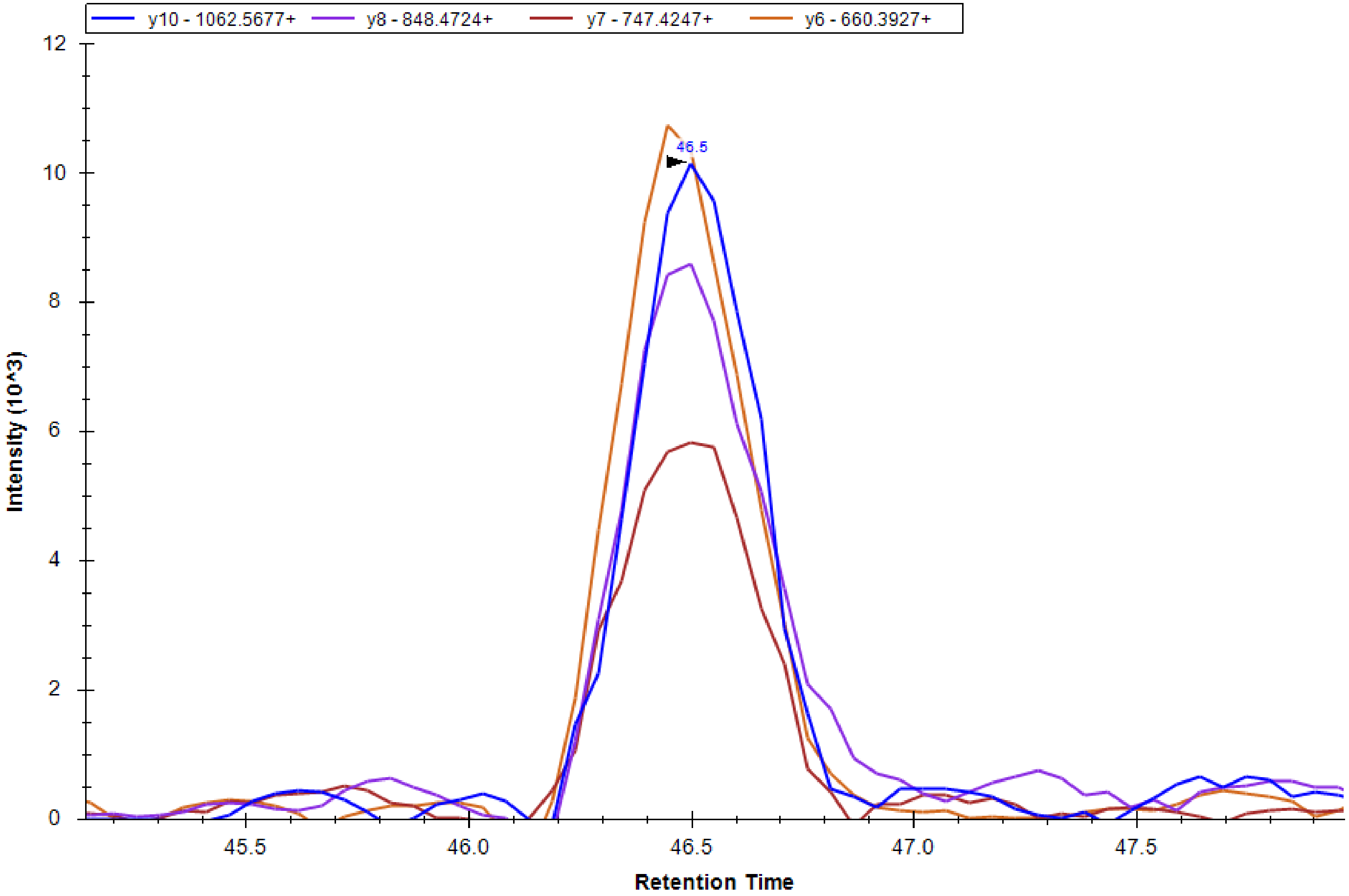

File name: 1604_JinanU(GZ)_MRM_#B_Full-Screen_0421.skyd

Parent ion m/z and charges: 703.8541++

NR_028350.3.3
SDAAVDTSSEIIAK

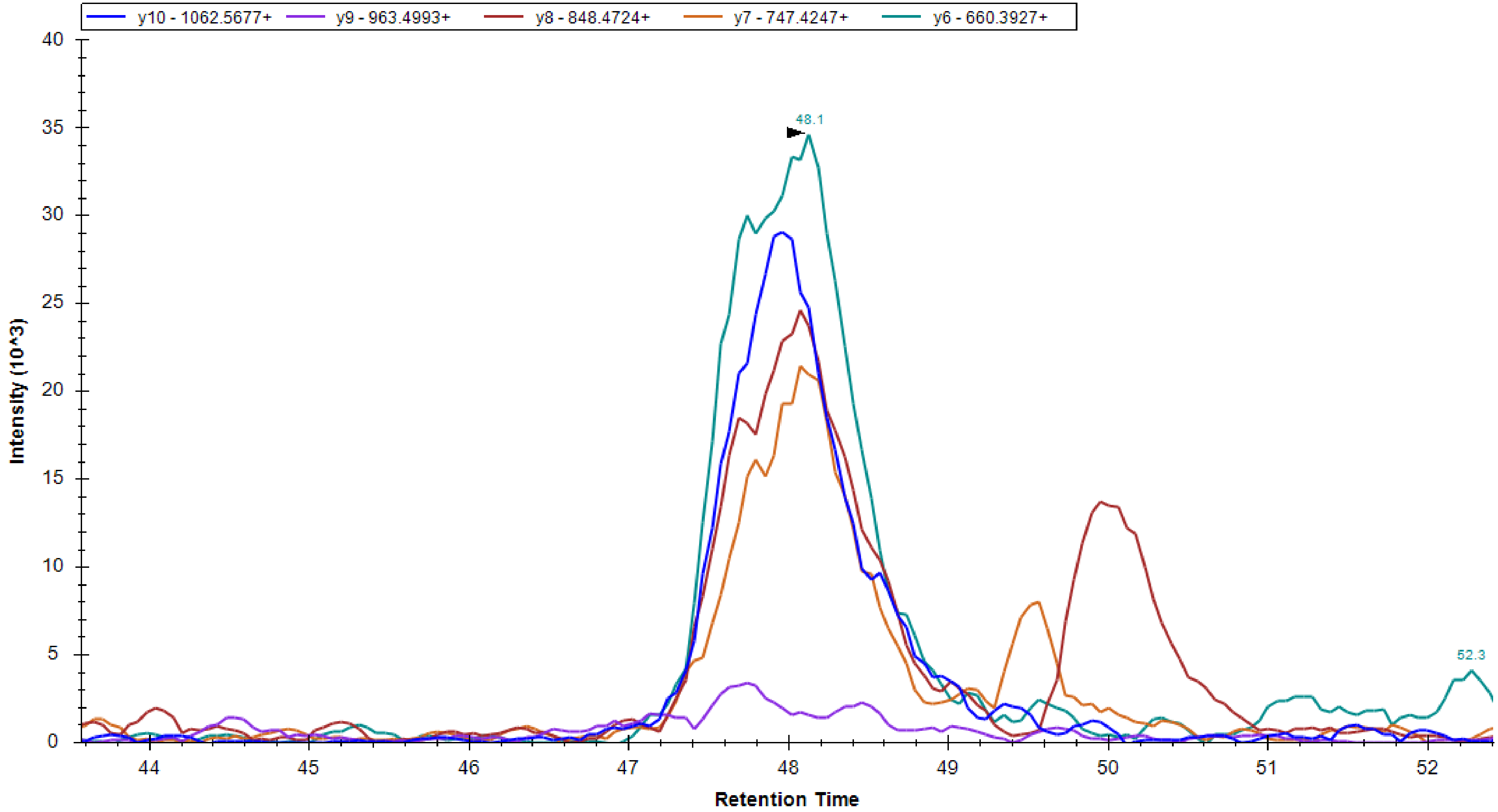

File name: 1604_JinanU(GZ)_MRM_#B_2nd_Round_Screen.skyd

Parent ion m/z and charges: 703.8541++

NR_033191.1.1
SLPGGGGGGGAGGGGGPGAERSAAAAR

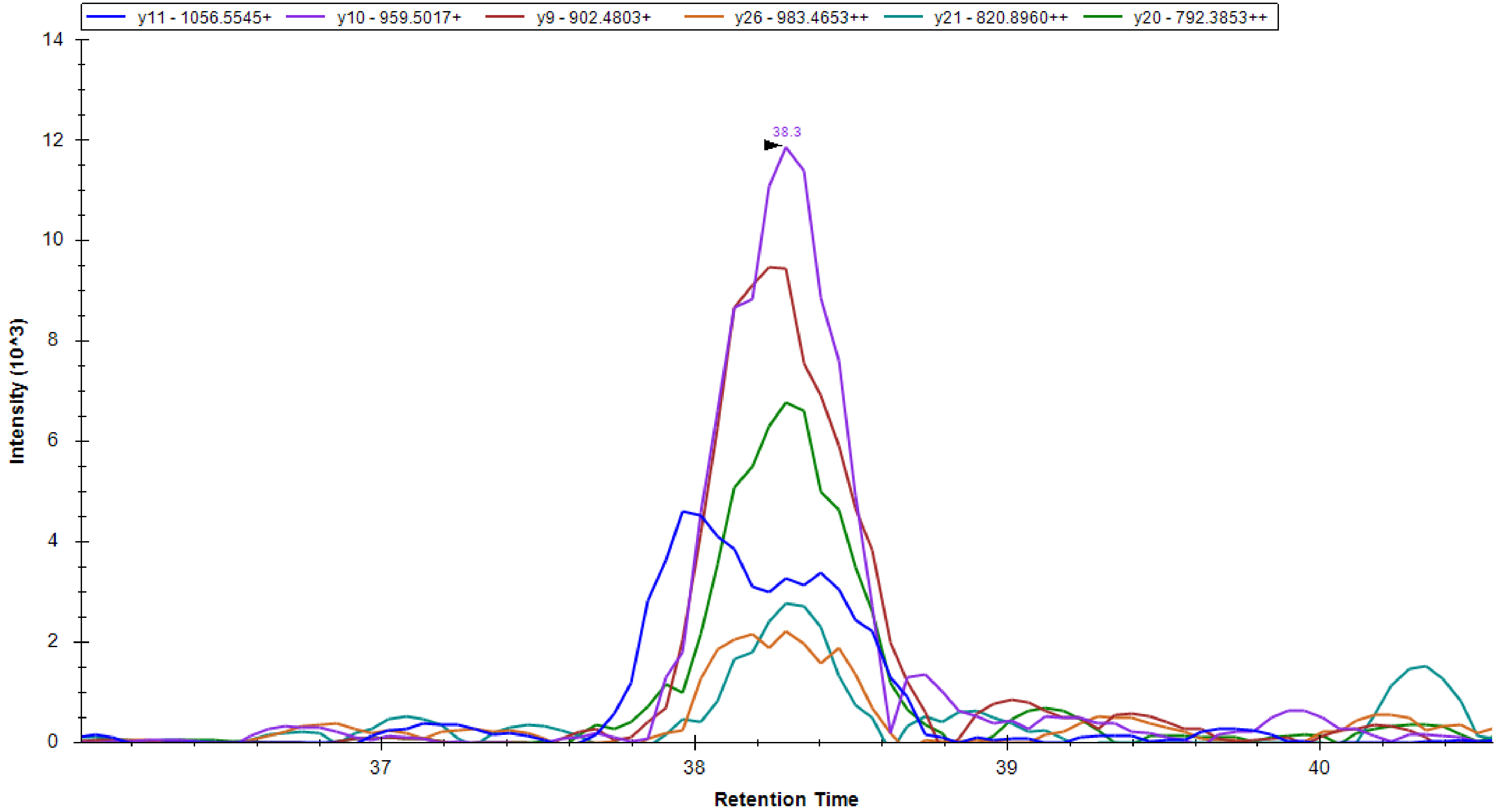

File name: 1604_JinanU(GZ)_MRM_#B_2nd_Round_Screen.skyd

Parent ion m/z and charges: 722.6847+++

NR_033863.1.1
AEPMQDLWPWKPK

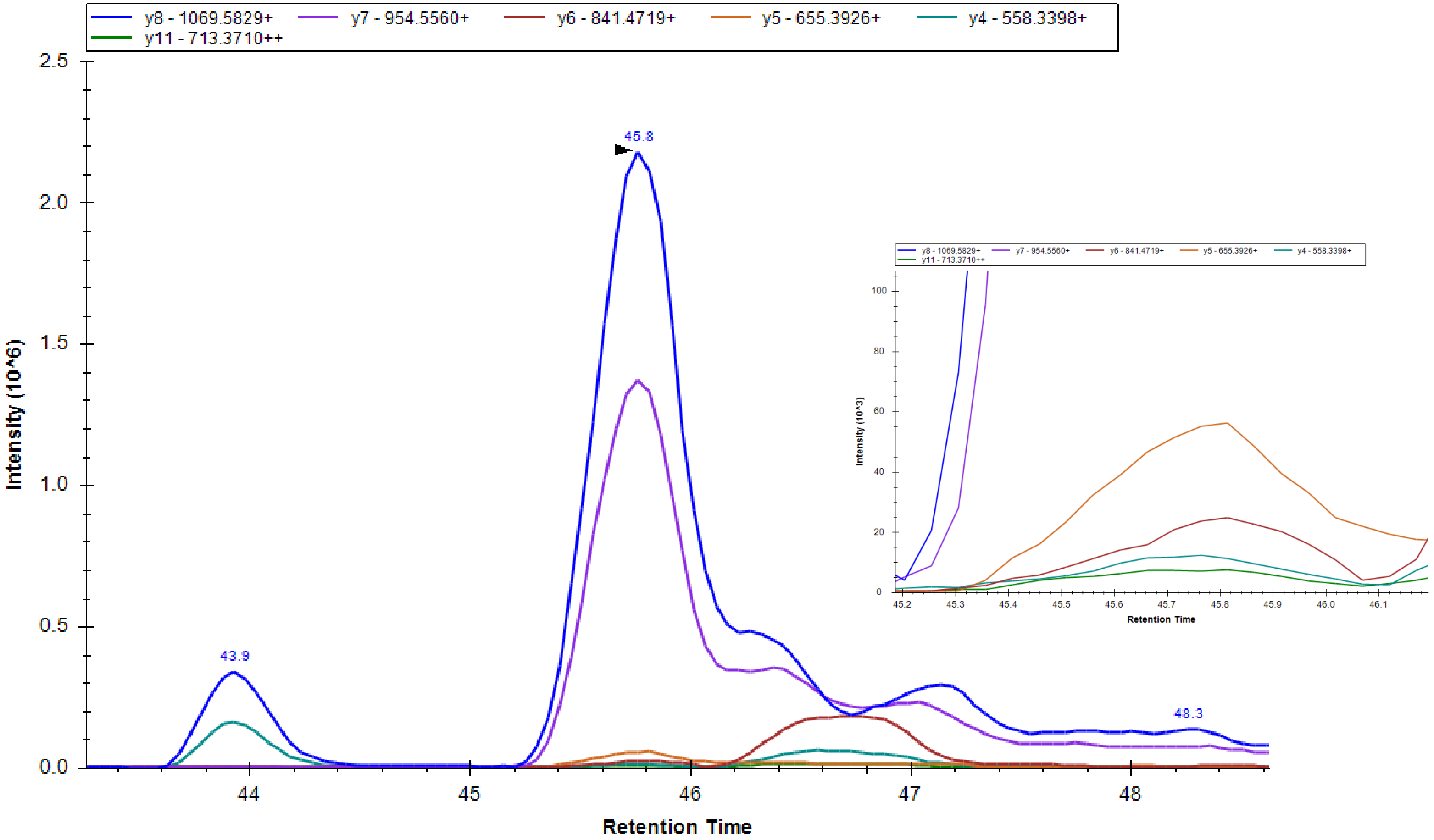

File name: 1604_JinanU(GZ)_MRM_#A_Full-Screen_0421.skyd

Parent ion m/z and charges: 813.4109++

NR_033863.1.1
AEPMQDLWPWKPK

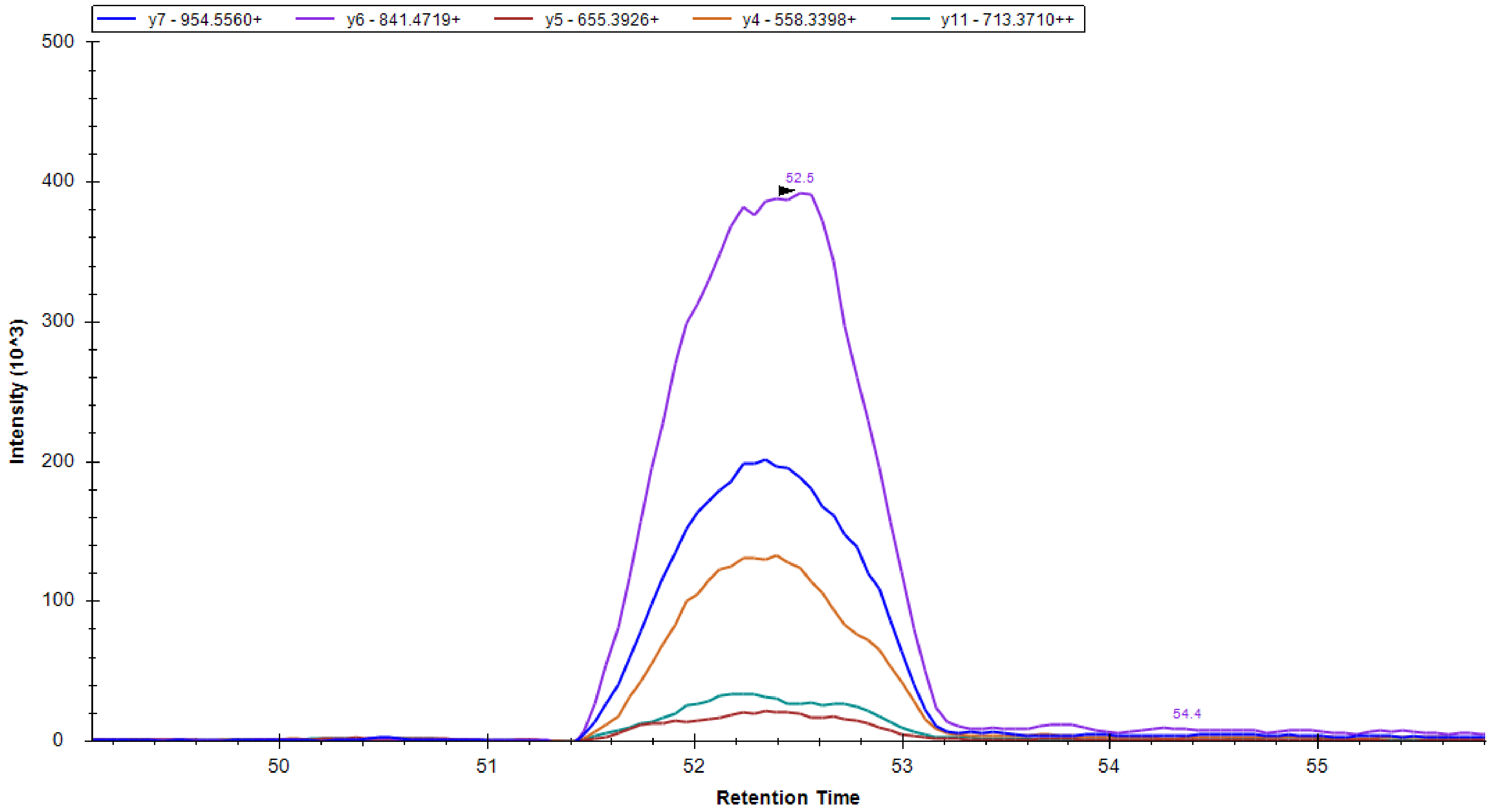

File name: 1604_JinanU(GZ)_MRM_#B_2nd_Round_Screen.skyd

Parent ion m/z and charges: 813.4109++

NR_033942.2.4
MPKQDLLVK

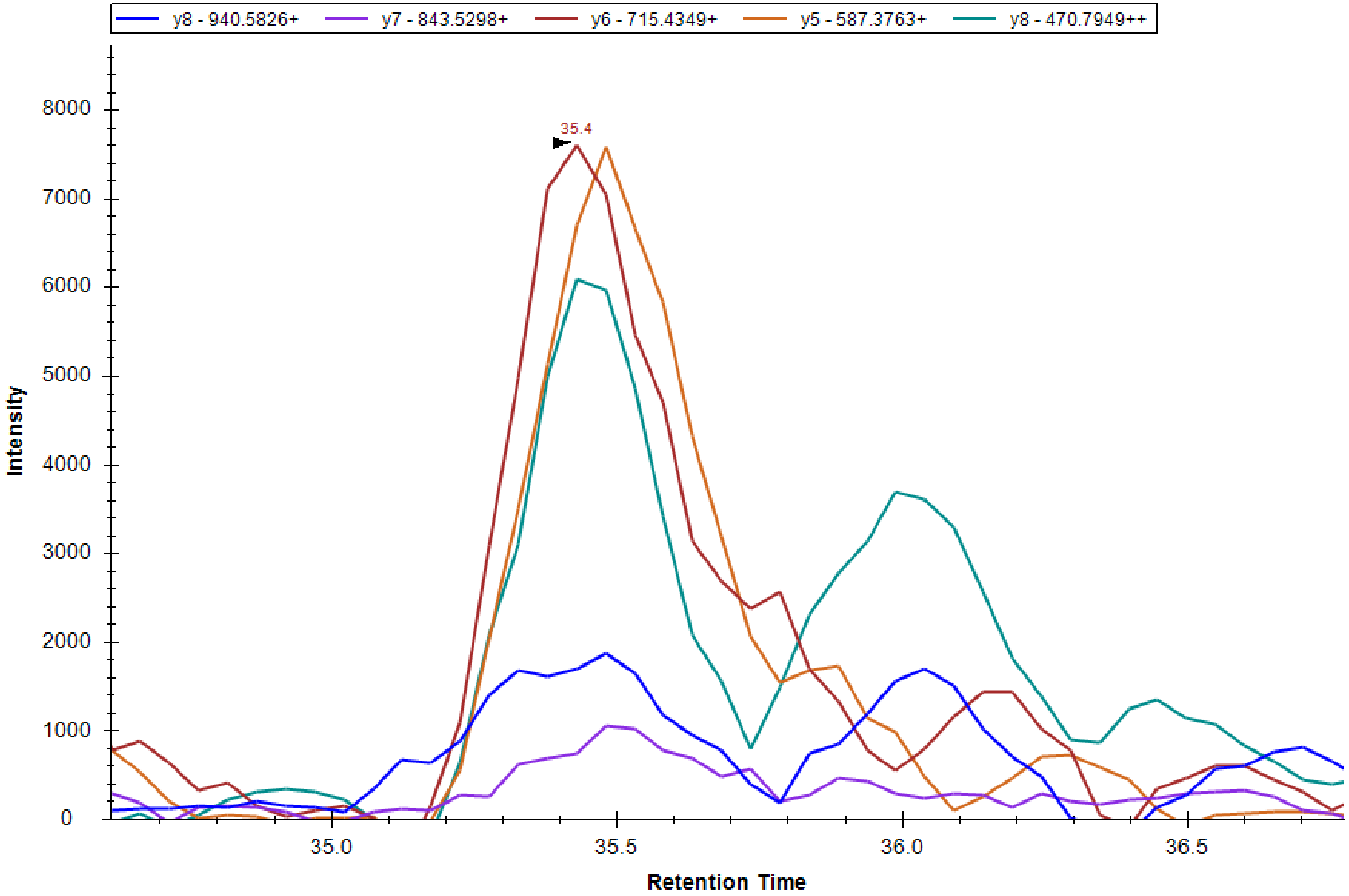

File name: 1604_JinanU(GZ)_MRM_#A_Full-Screen_0421.skyd

Parent ion m/z and charges: 536.3152++

NR_033942.2.4
LDQQSLSLPPAGVR

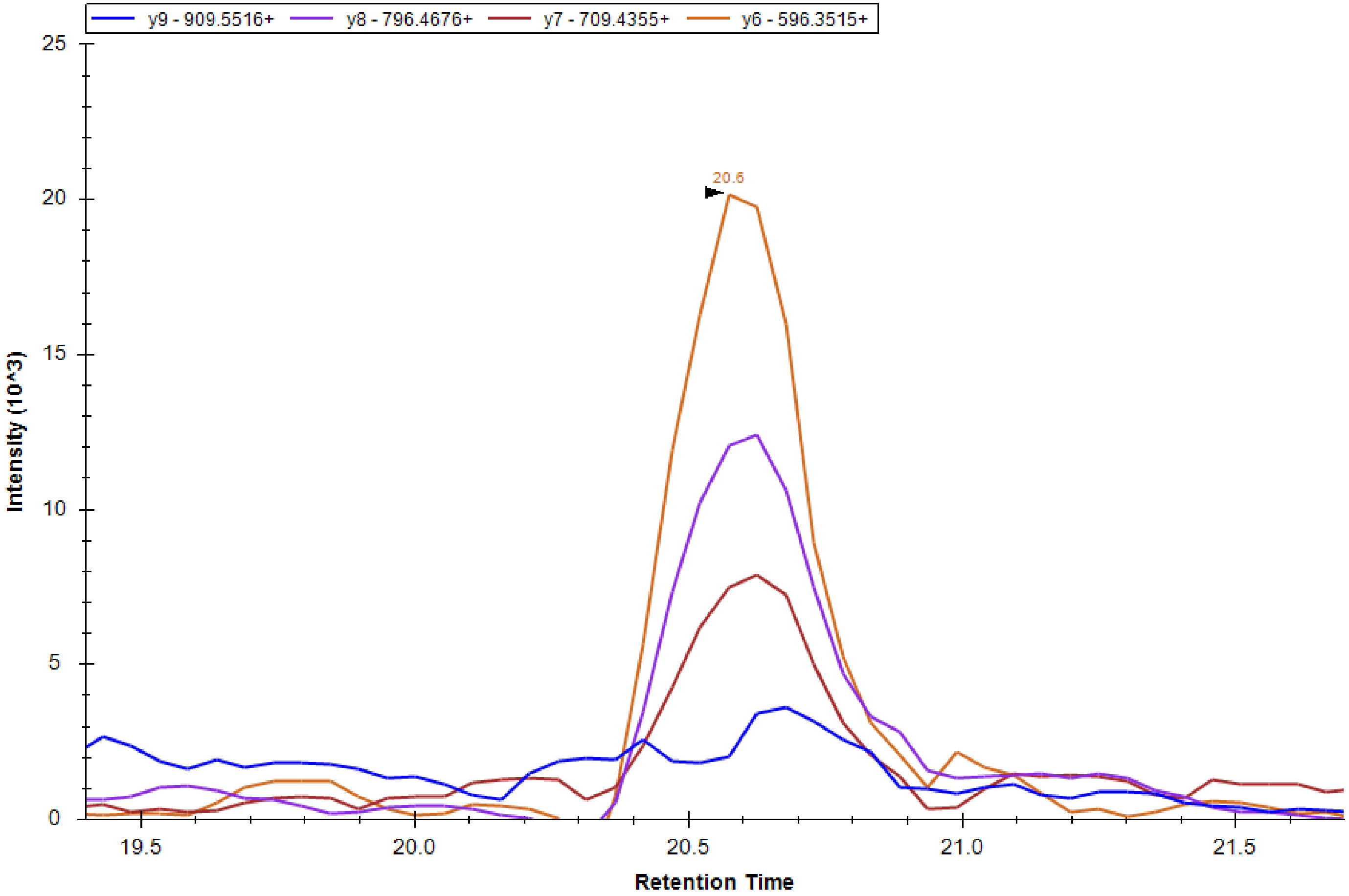

File name: 1604_JinanU(GZ)_MRM_#A_Full-Screen_0421.skyd

Parent ion m/z and charges: 740.9095++

NR_033942.2.4
LDQQSLSLPPAGVR

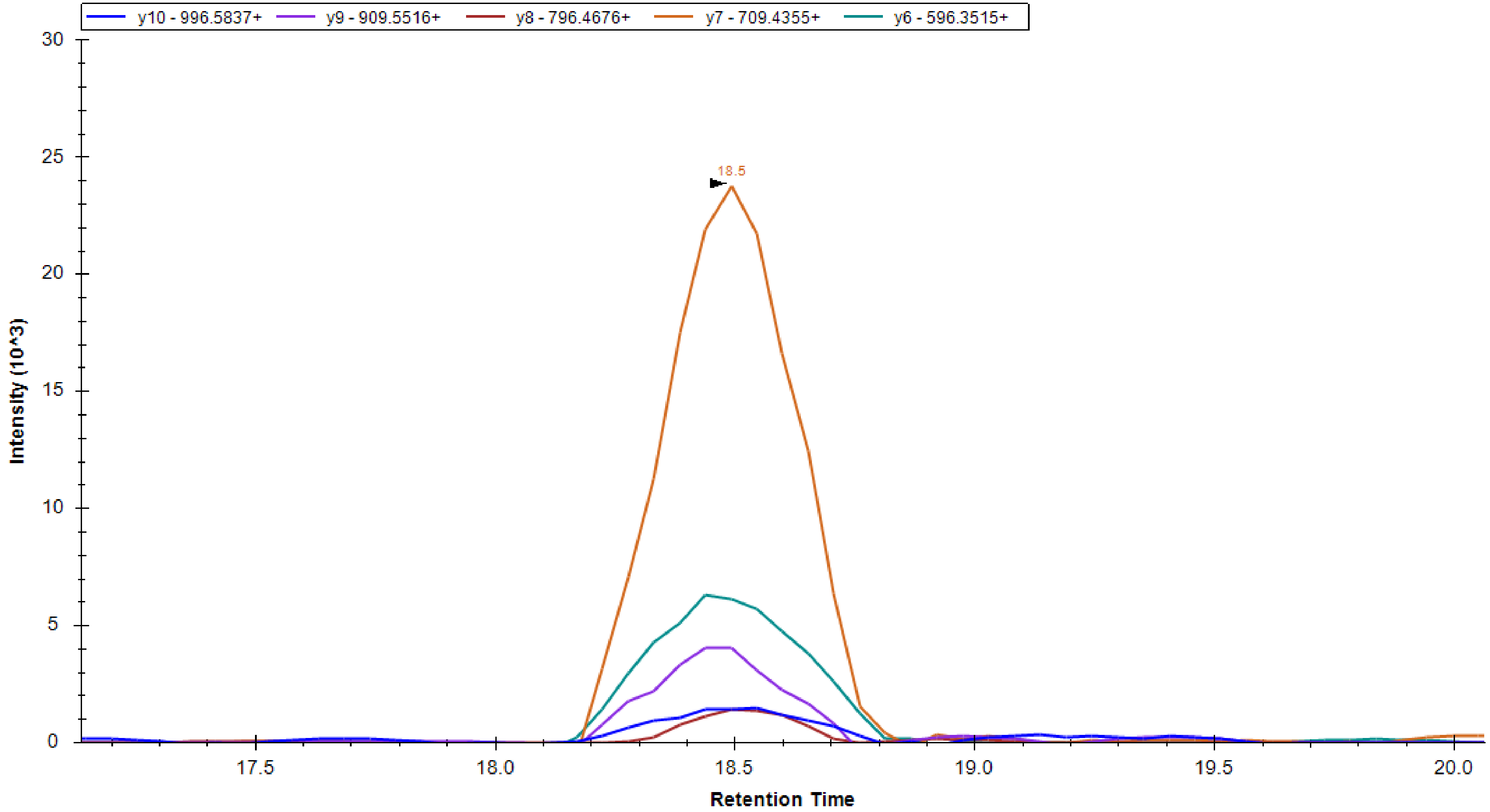

File name: 1604_JinanU(GZ)_MRM_#B_2nd_Round_Screen.skyd

Parent ion m/z and charges: 740.9095++

NR_034009.2.2
MSMRNLEAGLGPDPLKK

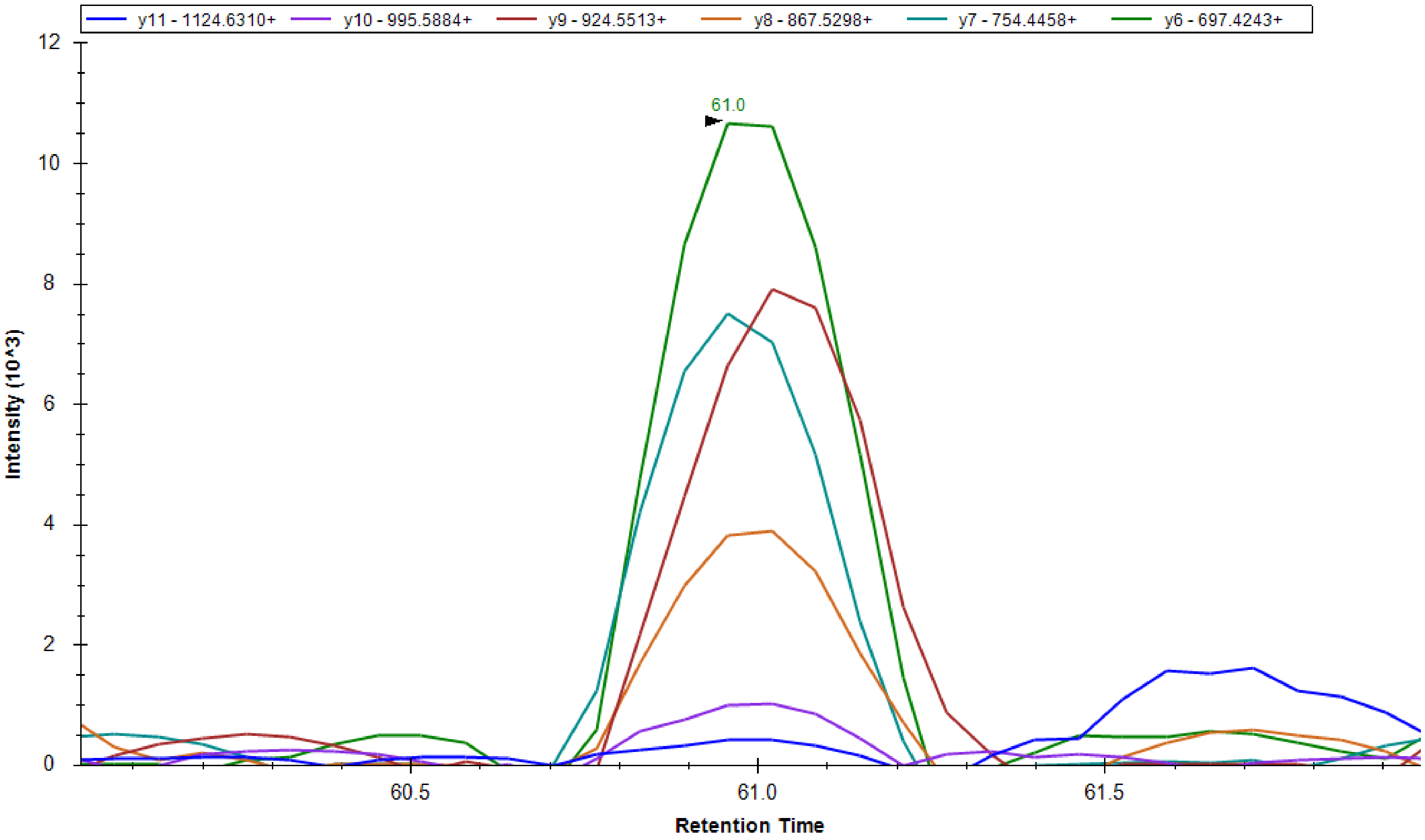

File name: 391-new#A-Round02_Negative-screen.skyd

Parent ion m/z and charges: 928.9897++

NR_034022.1.26
SELQFTIATENKITR

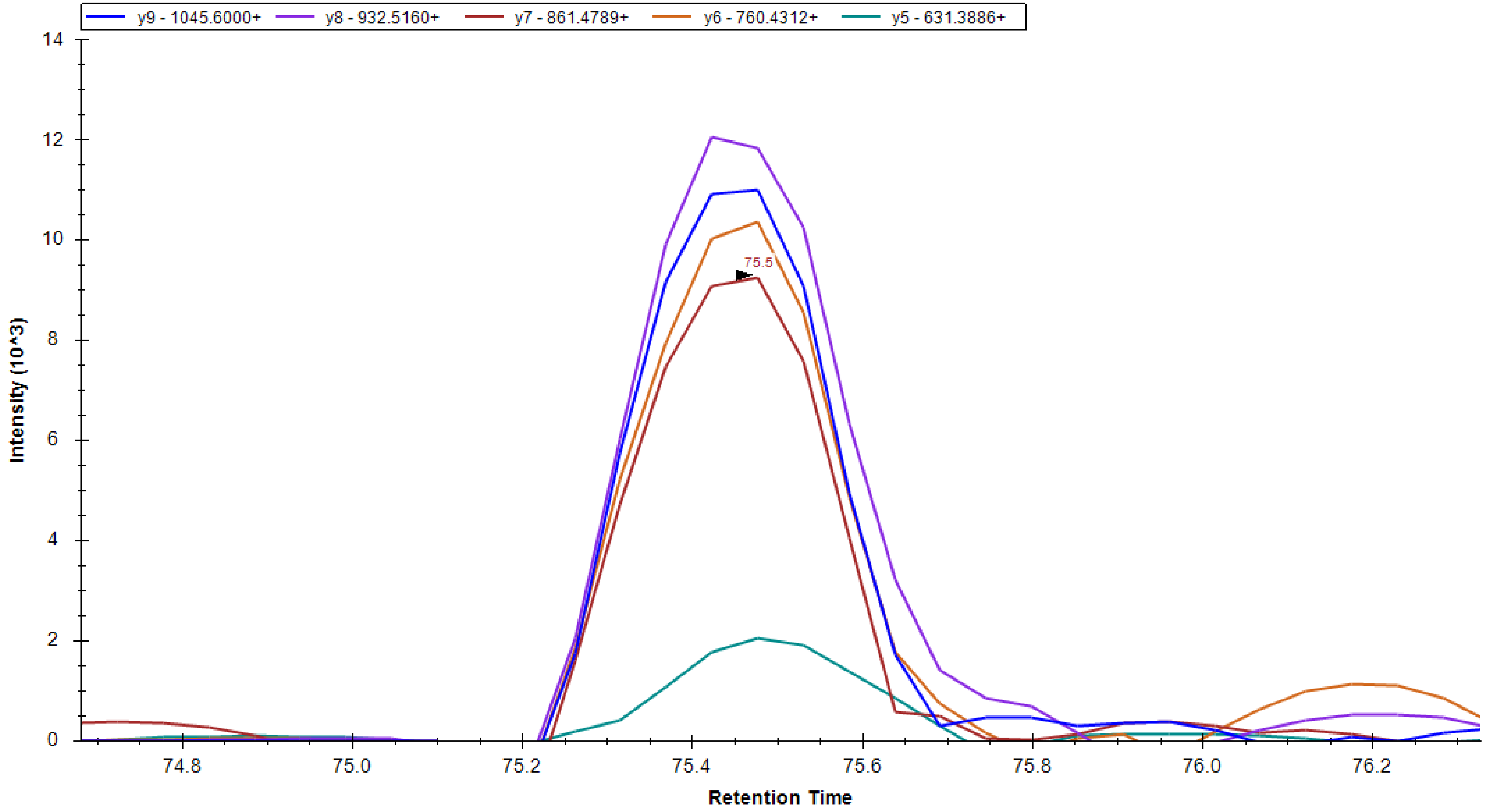

File name: 1604_JinanU(GZ)_MRM_#B_2nd_Round_Screen.skyd

Parent ion m/z and charges: 875.9703++

NR_037893.2.1
SLMLRNRFWEDPEDPLVAAMLR

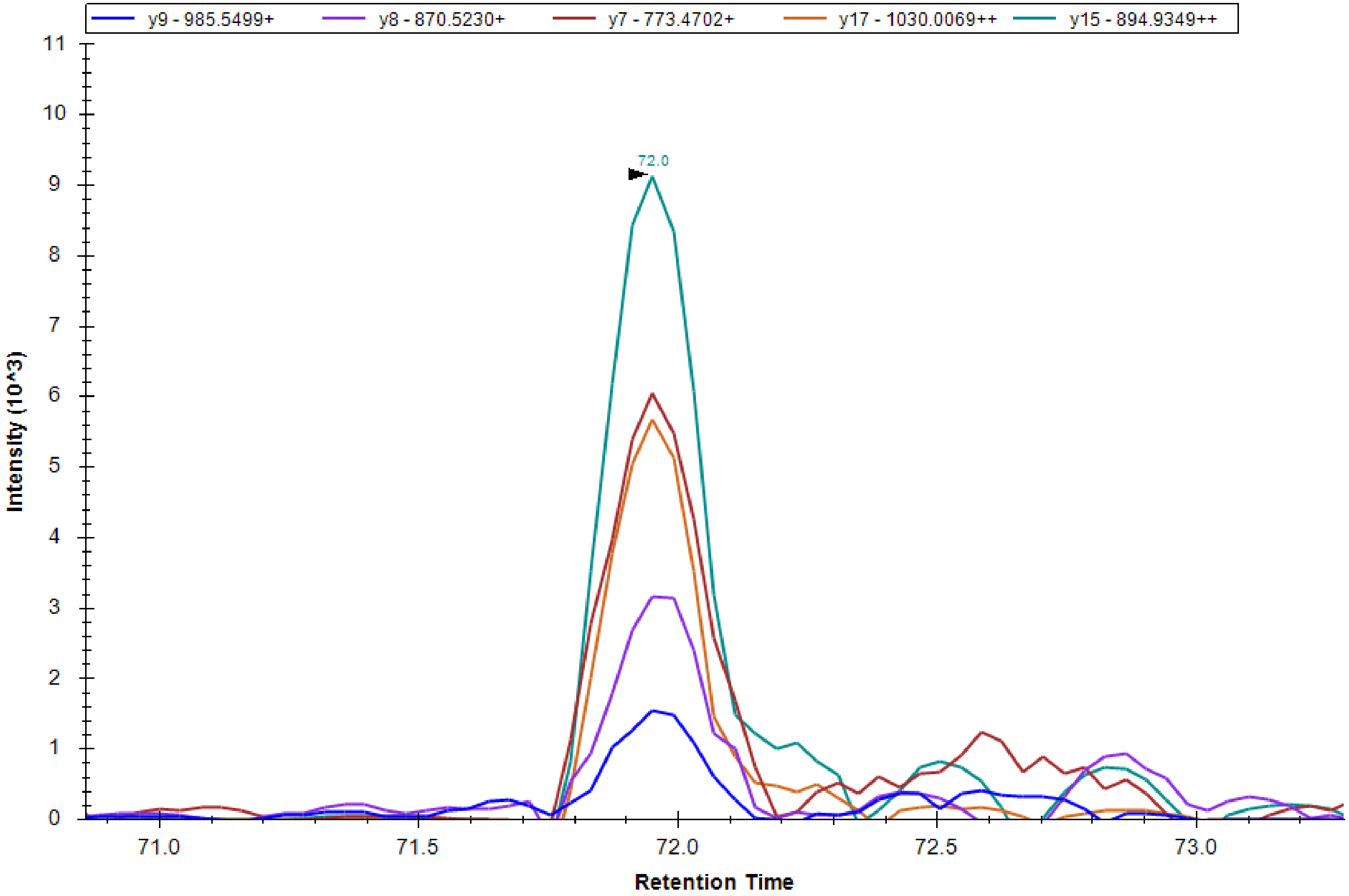

File name: 1604_JinanU(GZ)_MRM_#A_#B_Positive_Screen.skyd

Parent ion m/z and charges: 887.1210+++

NR_038275.3.4
SGSRLS**C**QHFGRPR

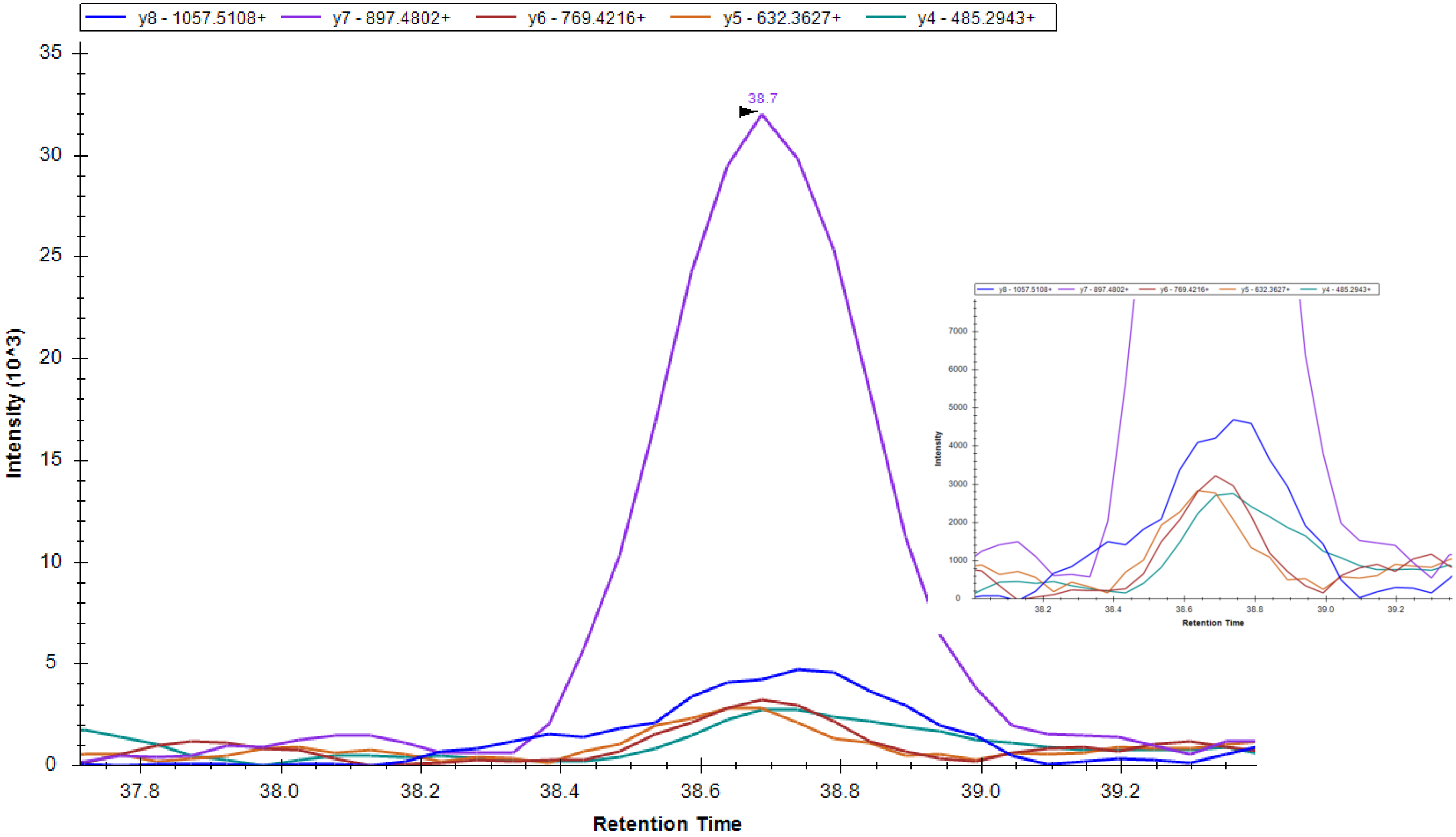

File name: 1604_JinanU(GZ)_MRM_#B_Full-Screen_0421.skyd

Parent ion m/z and charges: 822.9104++

NR_038275.3.4
SGSRLS**C**QHFGRPR

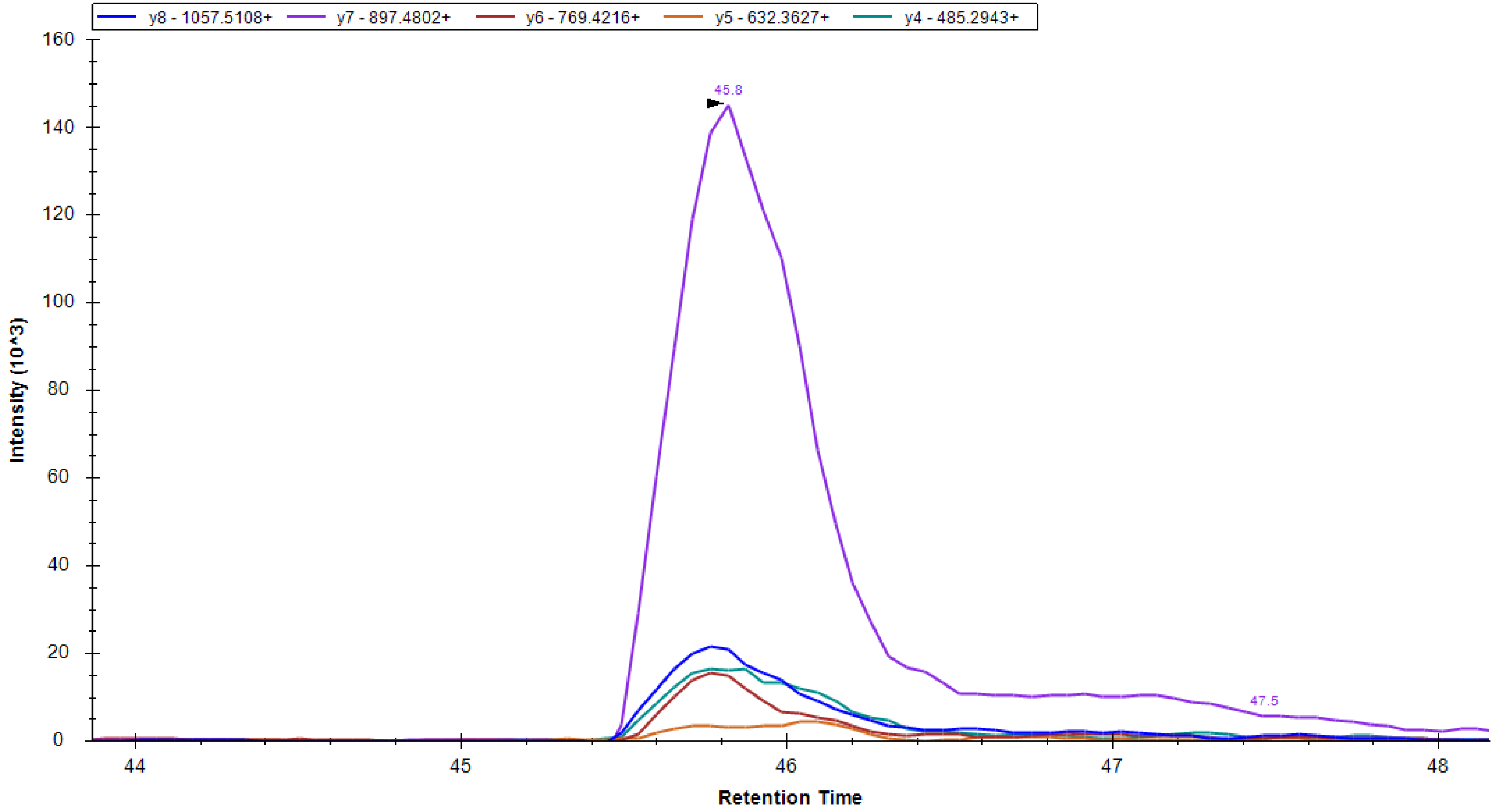

File name: 1604_JinanU(GZ)_MRM_#B_2nd_Round_Screen.skyd

Parent ion m/z and charges: 822.9104++

NR_038275.3.4
KQKPPSSPK

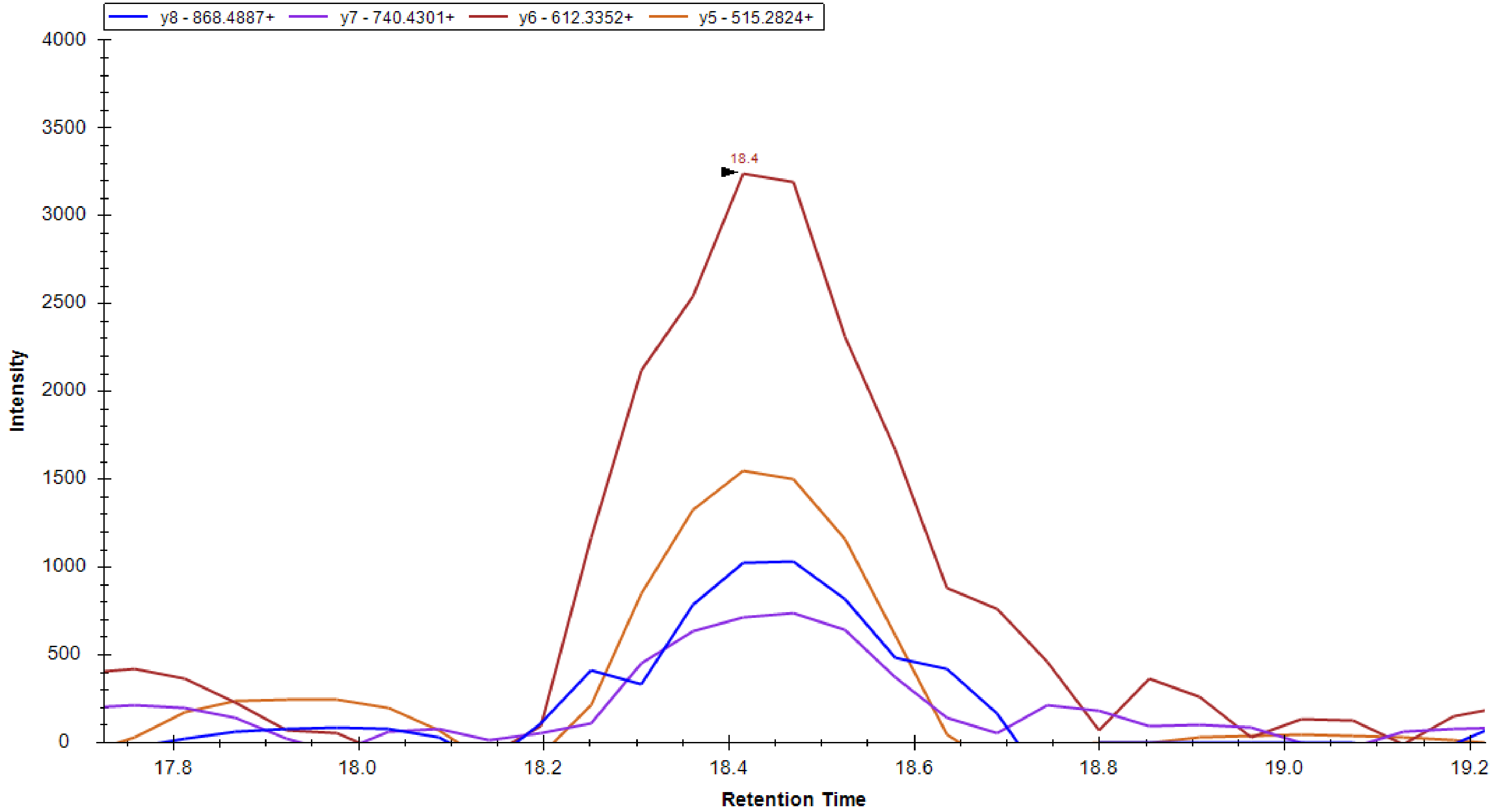

File name: 1604_JinanU(GZ)_MRM_#B_2nd_Round_Screen.skyd

Parent ion m/z and charges: 498.7955++

NR_038444.1.10
EPSQLTAPSASR

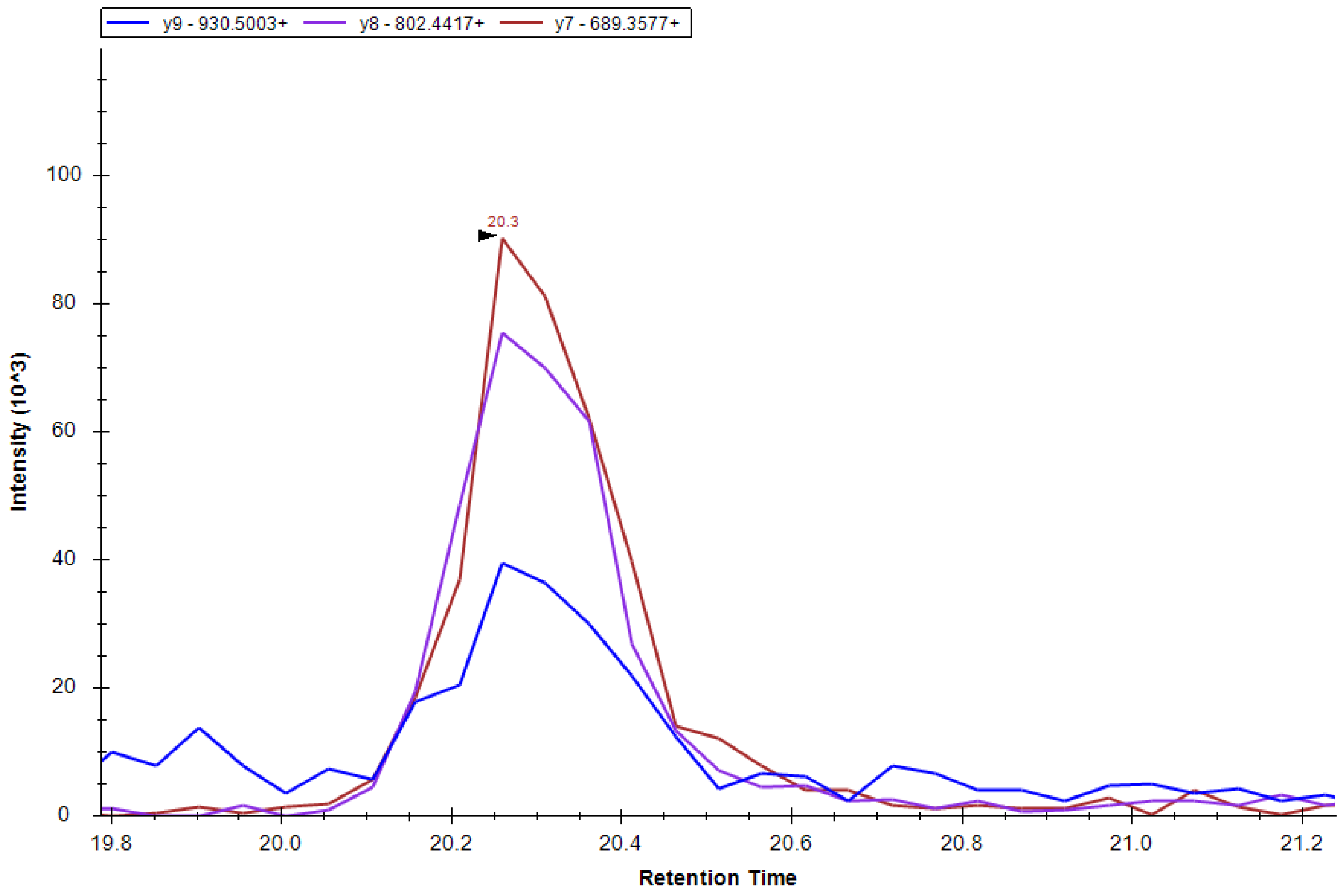

File name: 1604_JinanU(GZ)_MRM_#B_Full-Screen_0421.skyd

Parent ion m/z and charges: 622.3175++

NR_038444.1.10
MEPSQLTAPSASR

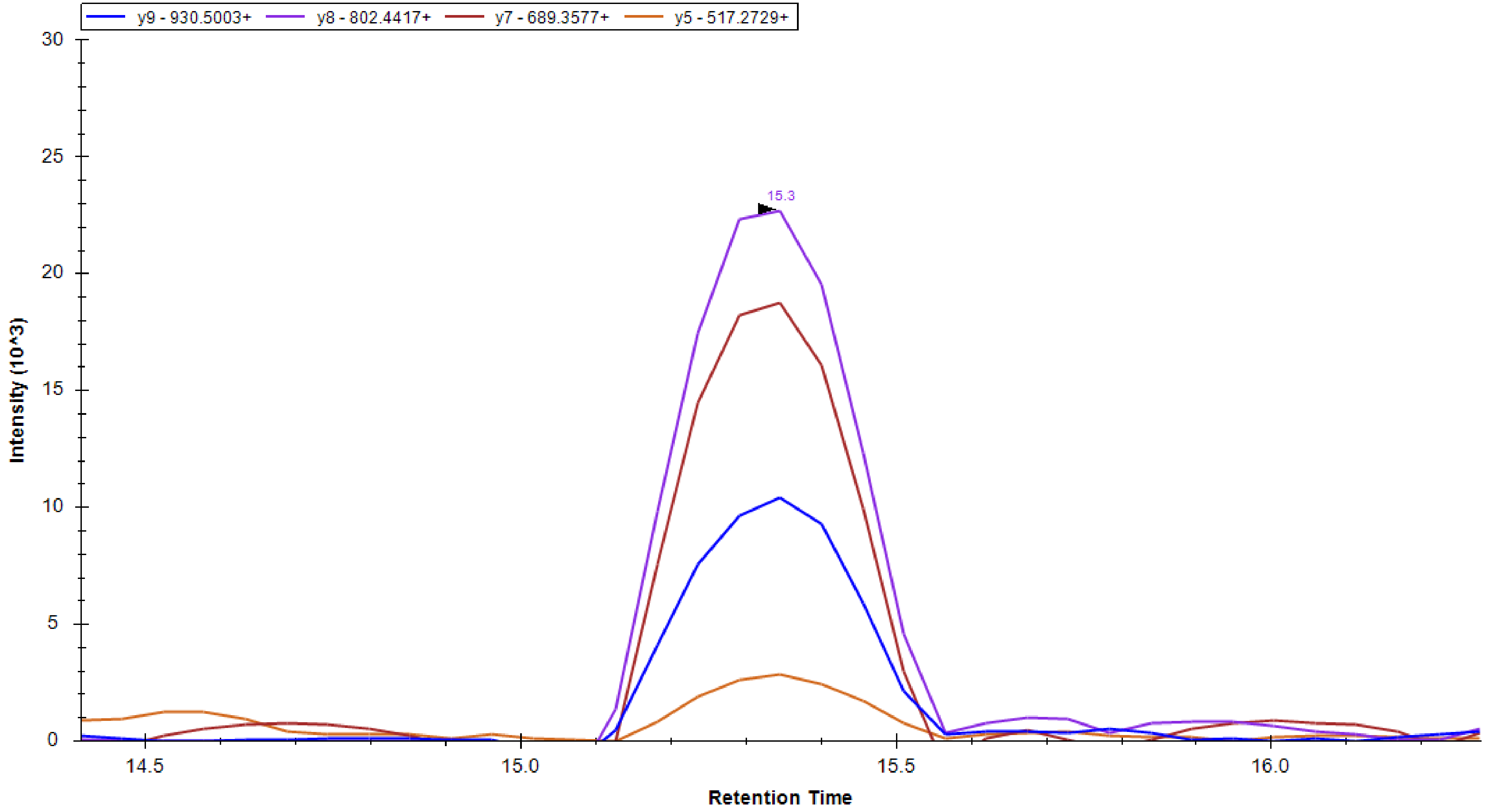

File name: 1604_JinanU(GZ)_MRM_#B_2nd_Round_Screen.skyd

Parent ion m/z and charges: 687.8377++

NR_040022.1.1
VGISIPTVR

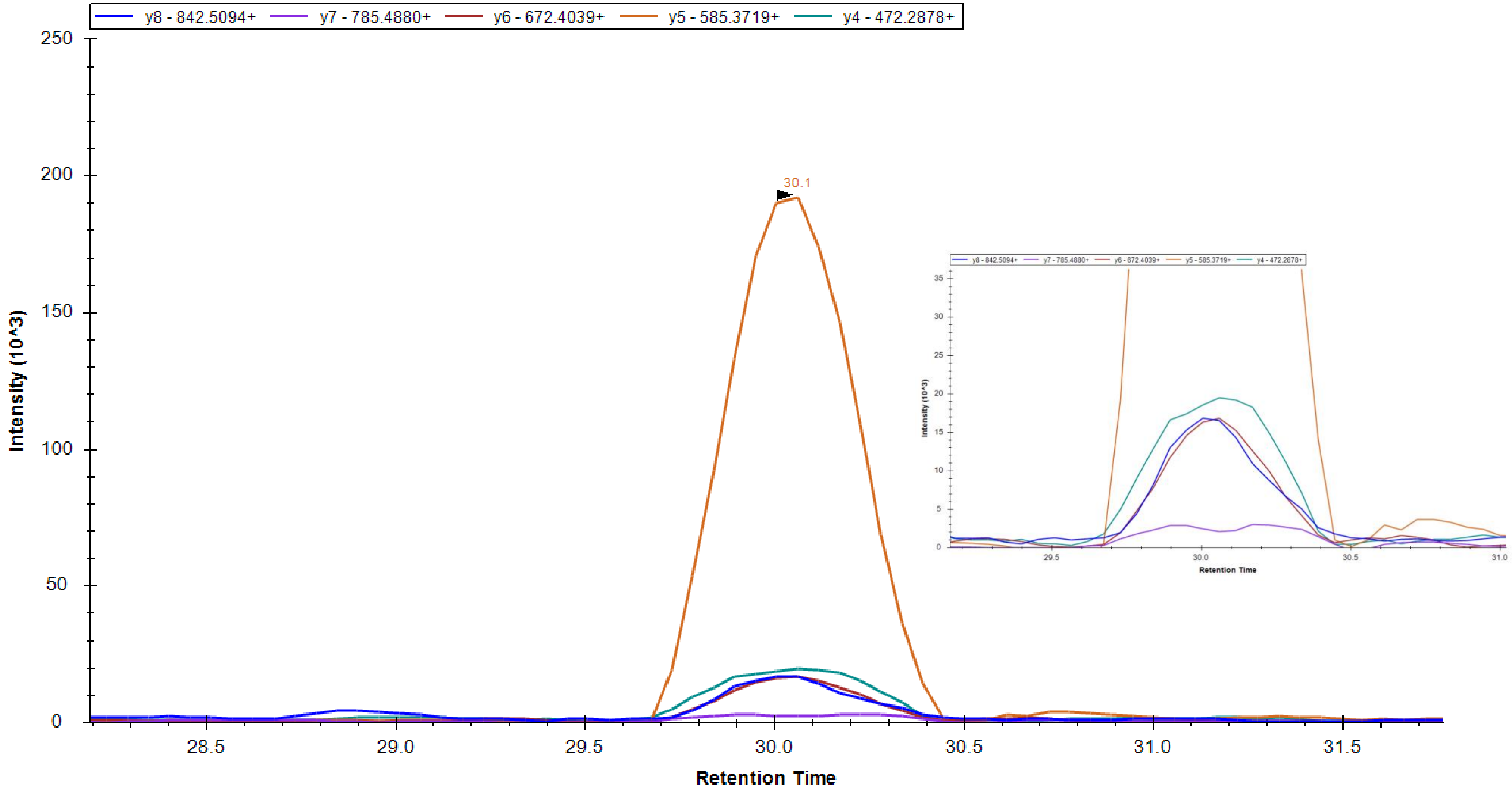

File name: 1604_JinanU(GZ)_MRM_#B_2nd_Round_Screen.skyd

Parent ion m/z and charges: 471.2926++

NR_040063.2.1
DPAWTGSLREESGTRQPR

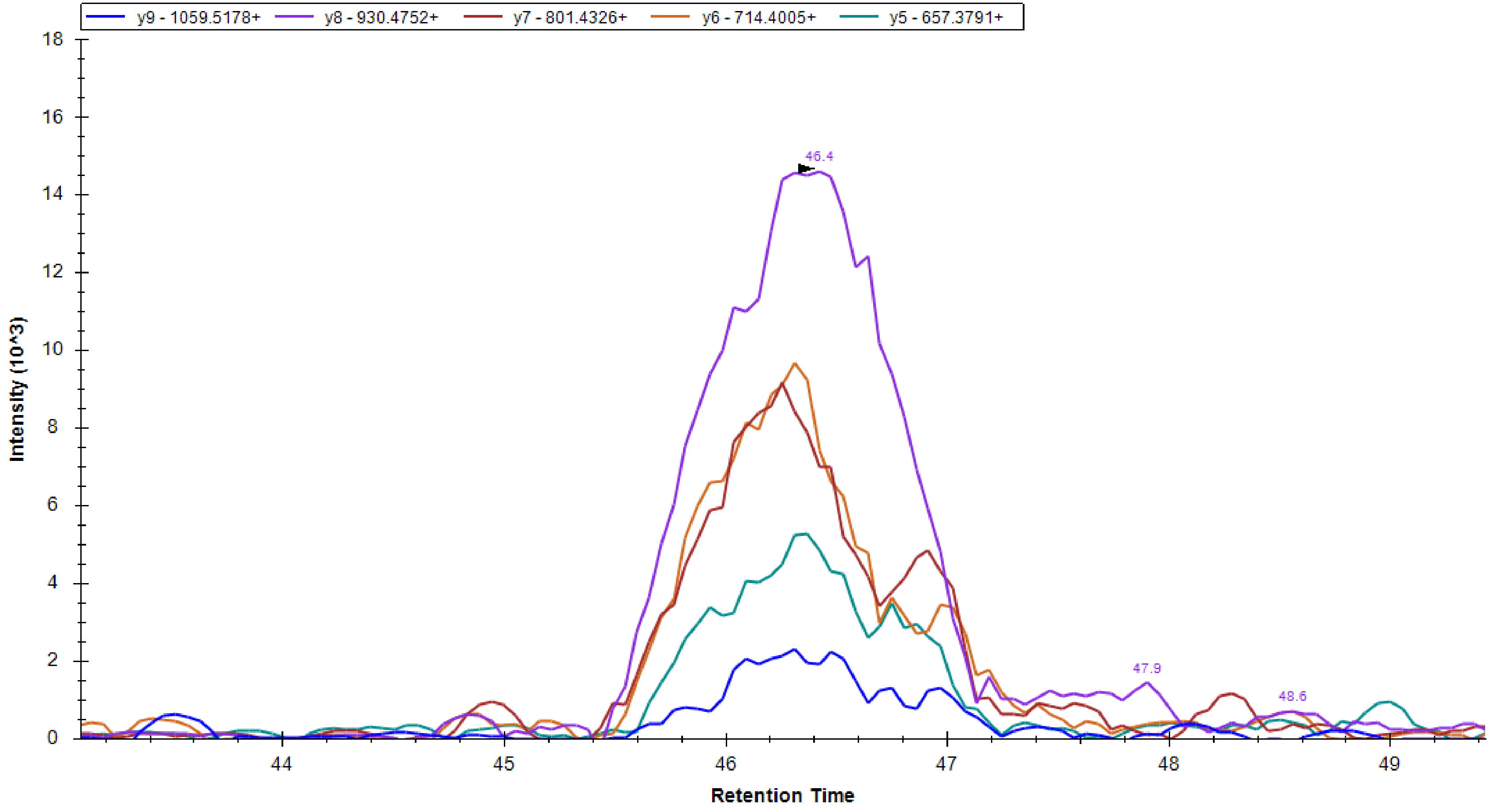

File name: 1604_JinanU(GZ)_MRM_#B_2nd_Round_Screen.skyd

Parent ion m/z and charges: 1022.0038++

NR_046216.2.27
MKTNMGTFFFFFER

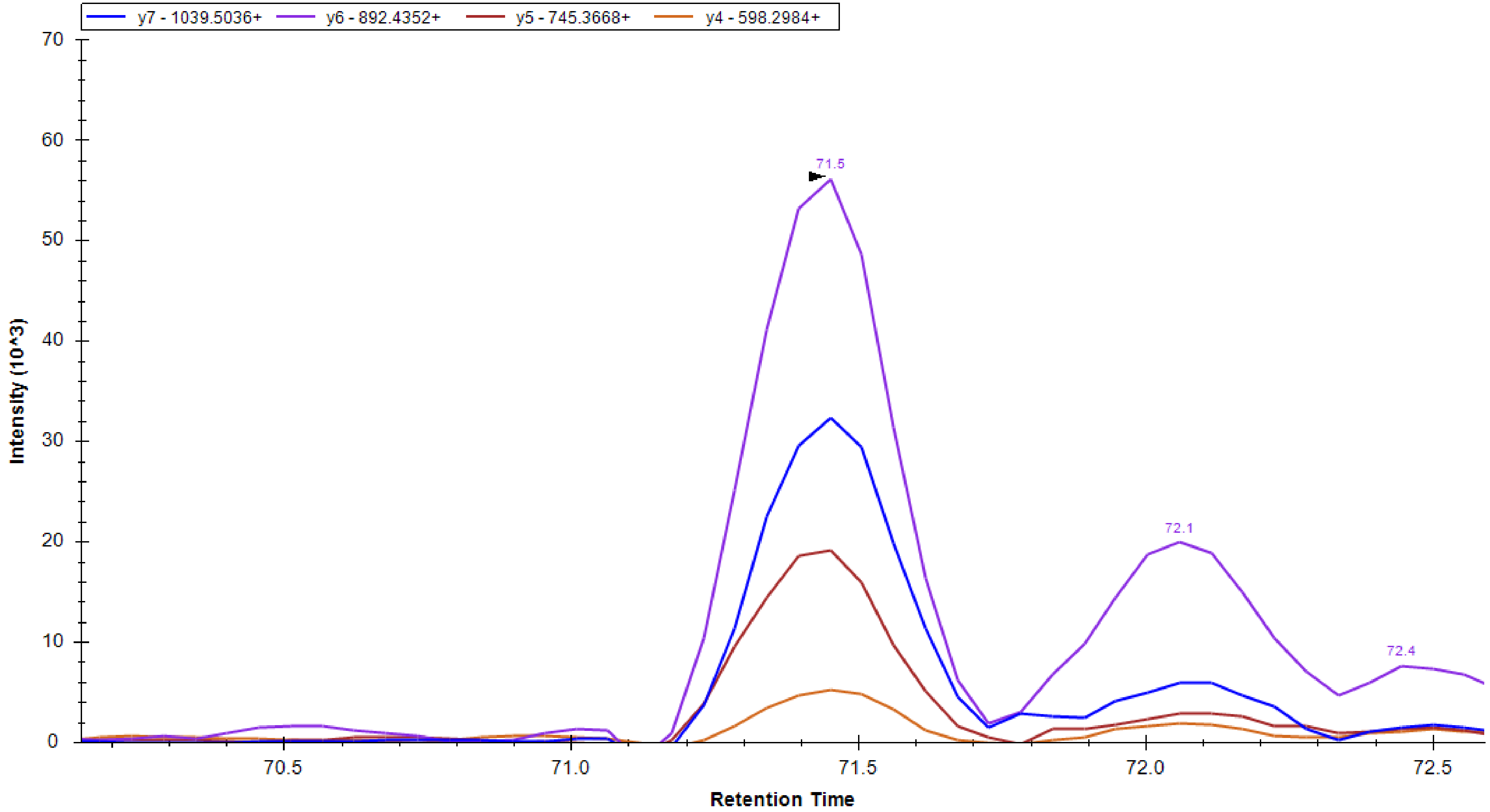

File name: 1604_JinanU(GZ)_MRM_#B_2nd_Round_Screen.skyd

Parent ion m/z and charges: 901.9233++

NR_046216.2.27
KTNMGTFFFFFER

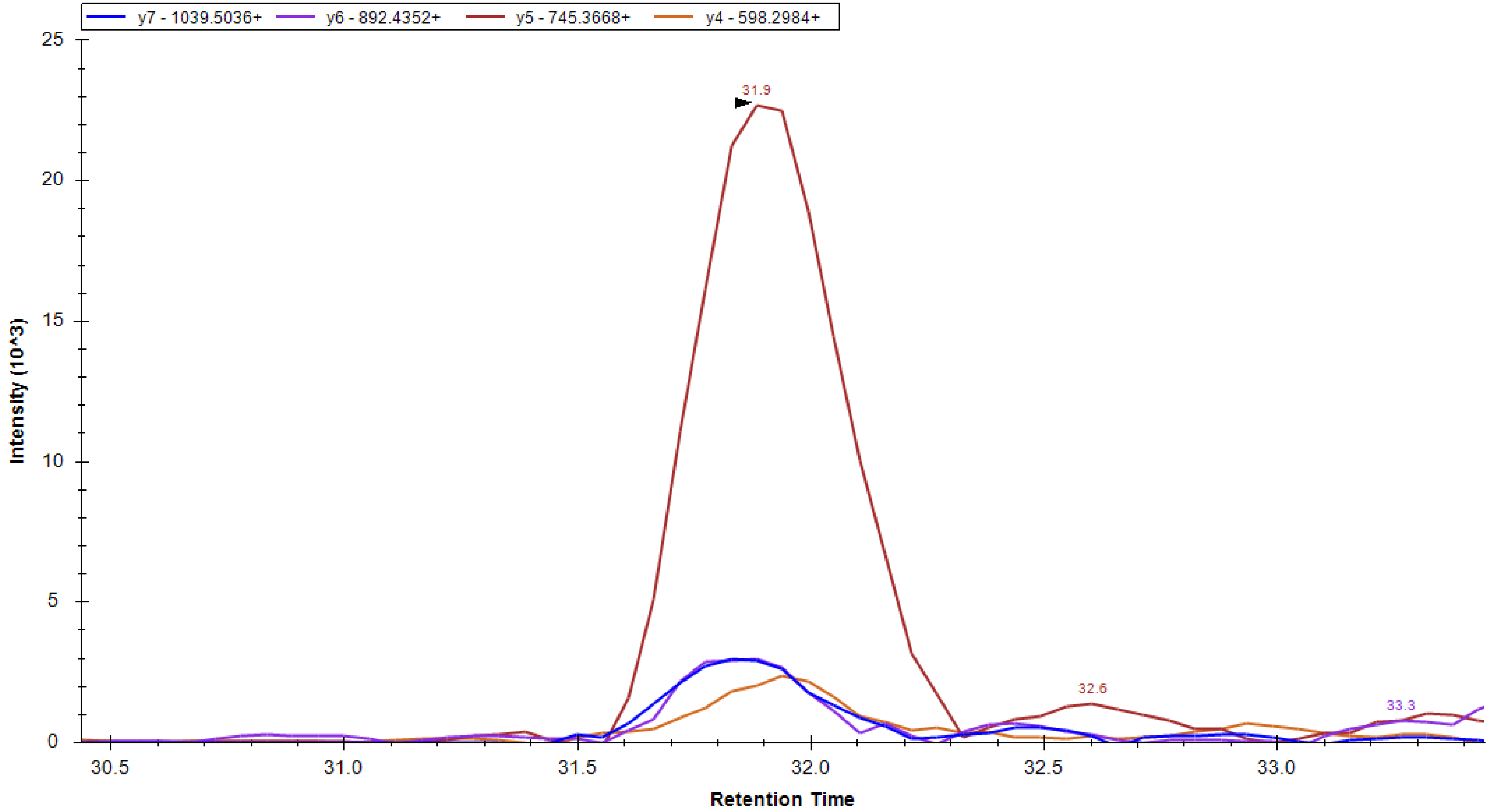

File name: 1604_JinanU(GZ)_MRM_#B_2nd_Round_Screen.skyd

Parent ion m/z and charges: 836.4030++

NR_046312.2.2
VLPCEGGAR

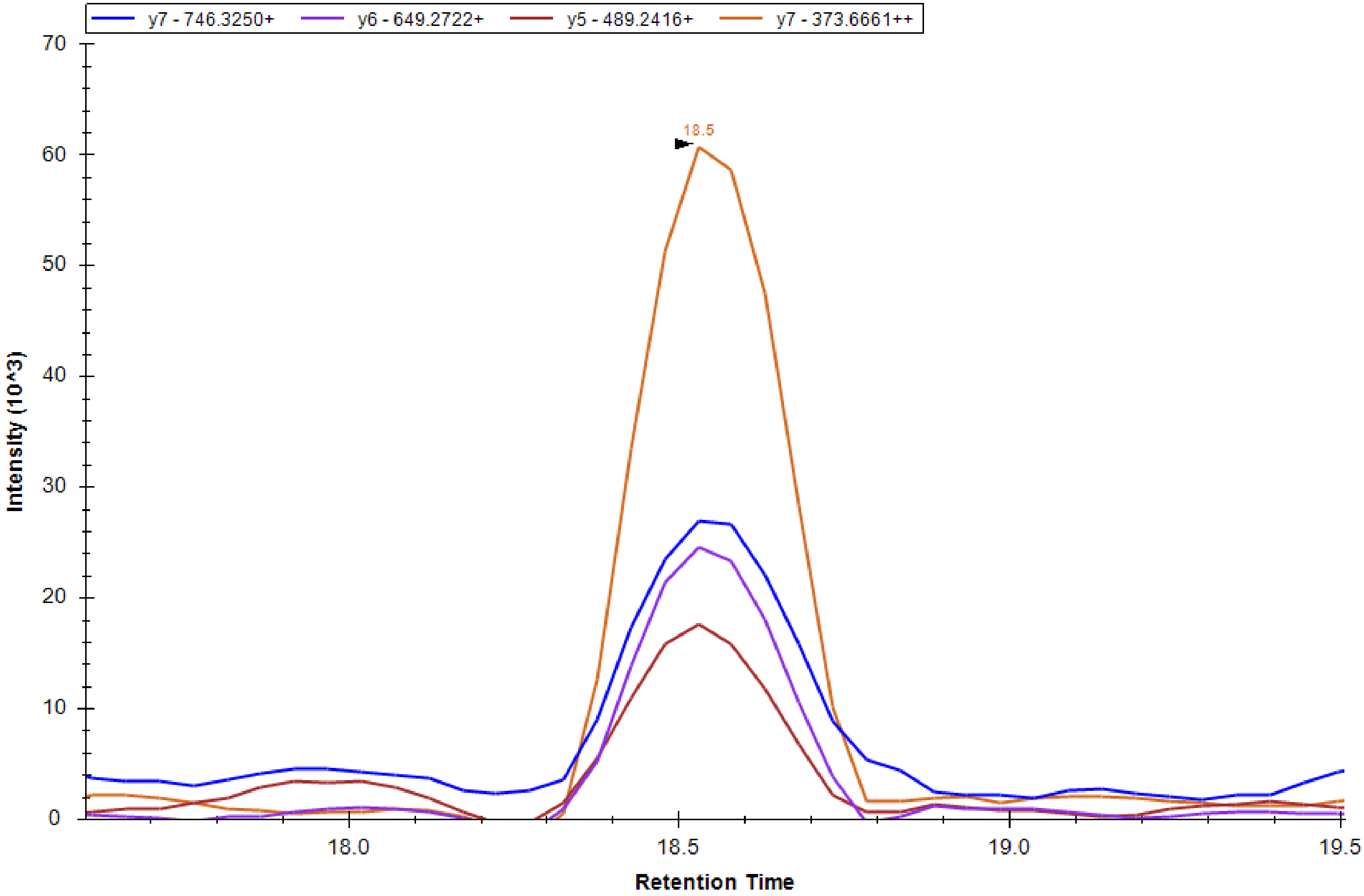

File name: 1604_JinanU(GZ)_MRM_#A_Full-Screen_0421.skyd

Parent ion m/z and charges: 479.7424++

NR_046312.2.2
MVLP<u>**C**</u>EGGARGTPASLR

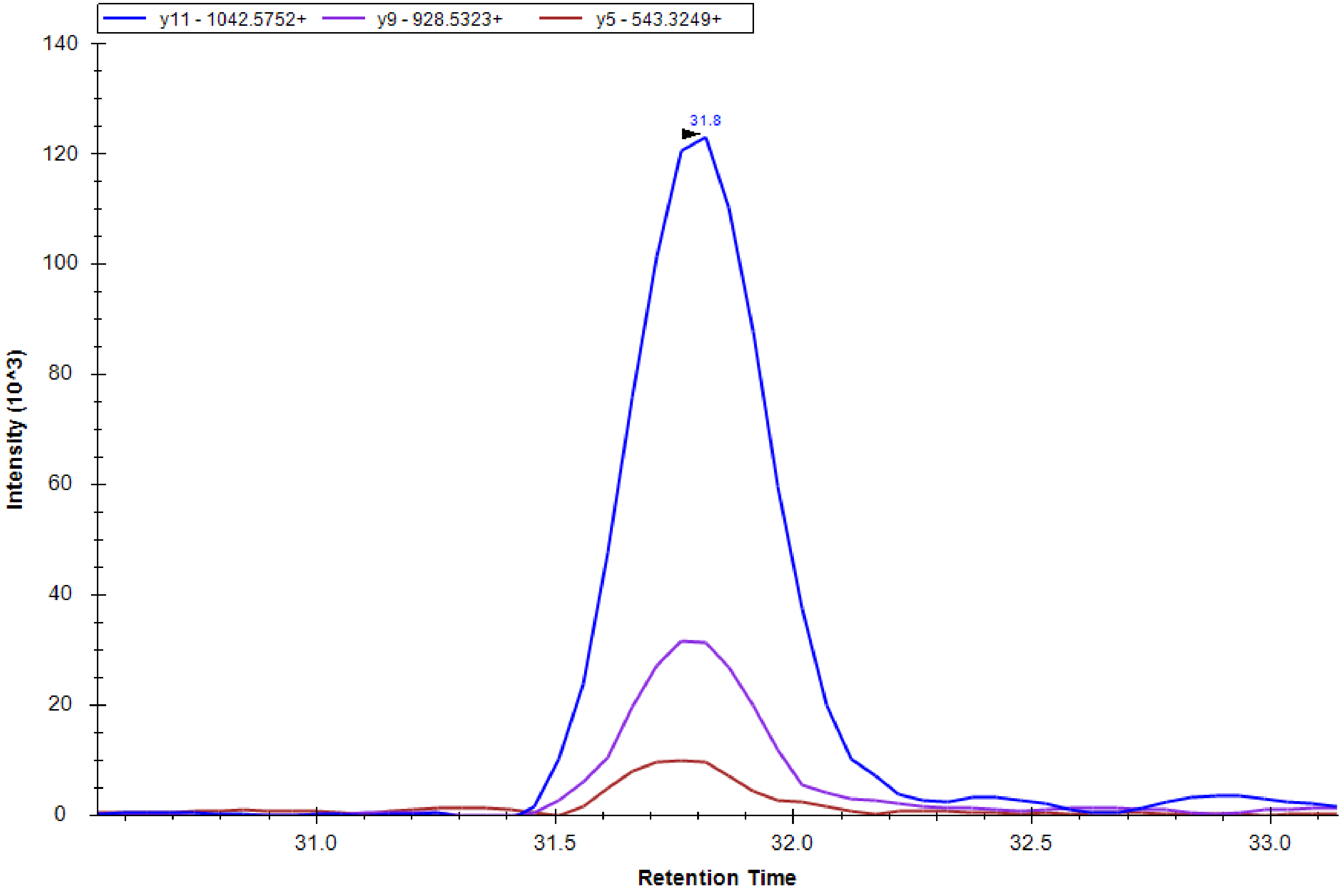

File name: 1604_JinanU(GZ)_MRM_#A_Full-Screen_0421.skyd

Parent ion m/z and charges: 886.4507++

NR_046312.2.2
VLPCEGGAR

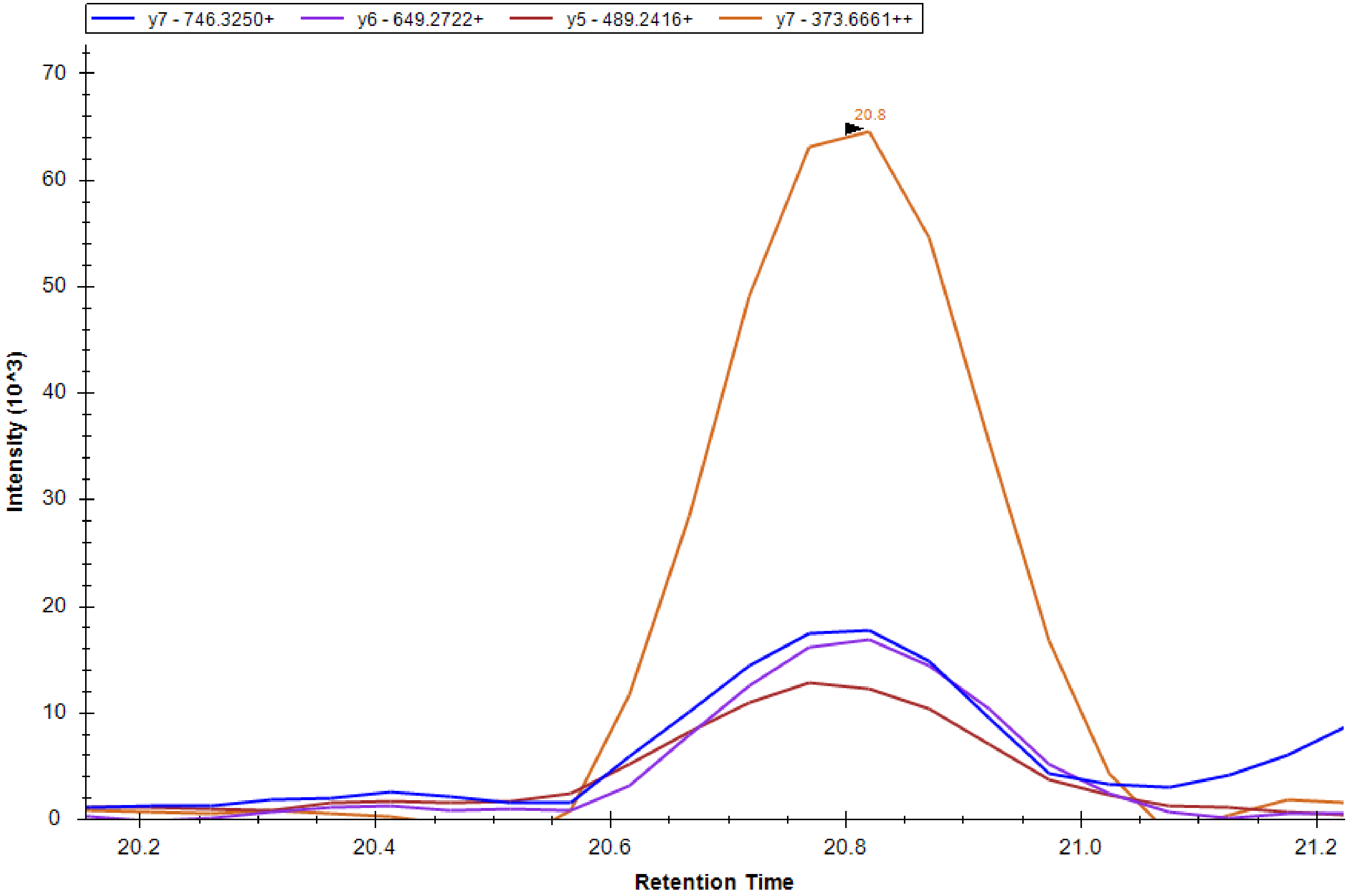

File name: 1604_JinanU(GZ)_MRM_#B_Full-Screen_0421.skyd

Parent ion m/z and charges: 479.7424++

NR_046312.2.2
MVLP**C**EGGARGTPASLR

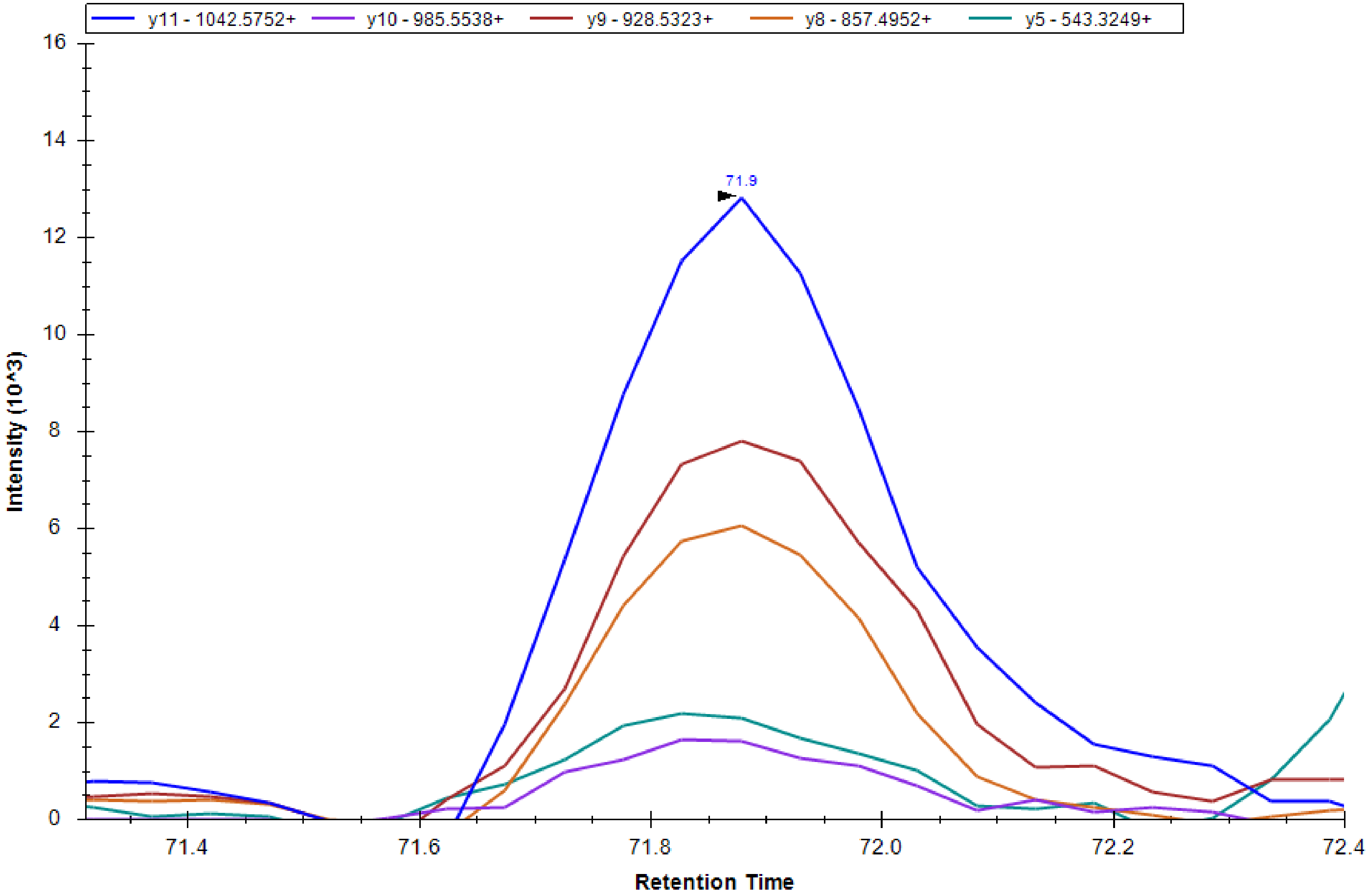

File name: 1604_JinanU(GZ)_MRM_#B_Full-Screen_0421.skyd

Parent ion m/z and charges: 886.4507++

NR_046312.2.2
MVLPCEGGARGTPASLR

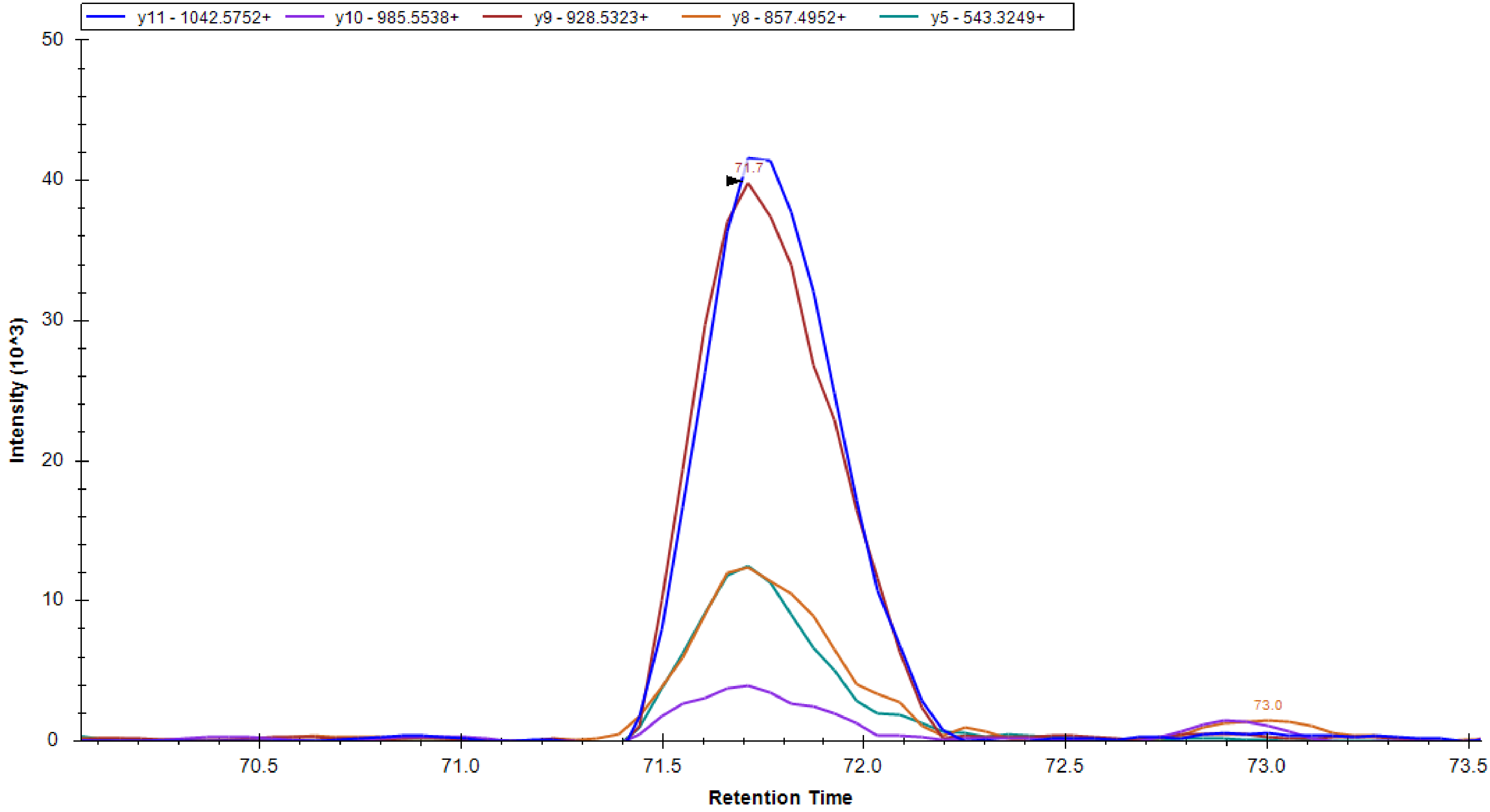

File name: 1604_JinanU(GZ)_MRM_#B_2nd_Round_Screen.skyd

Parent ion m/z and charges: 886.4507++

NR_049769.1.3
MAKQAQQGPGATLDPKGR

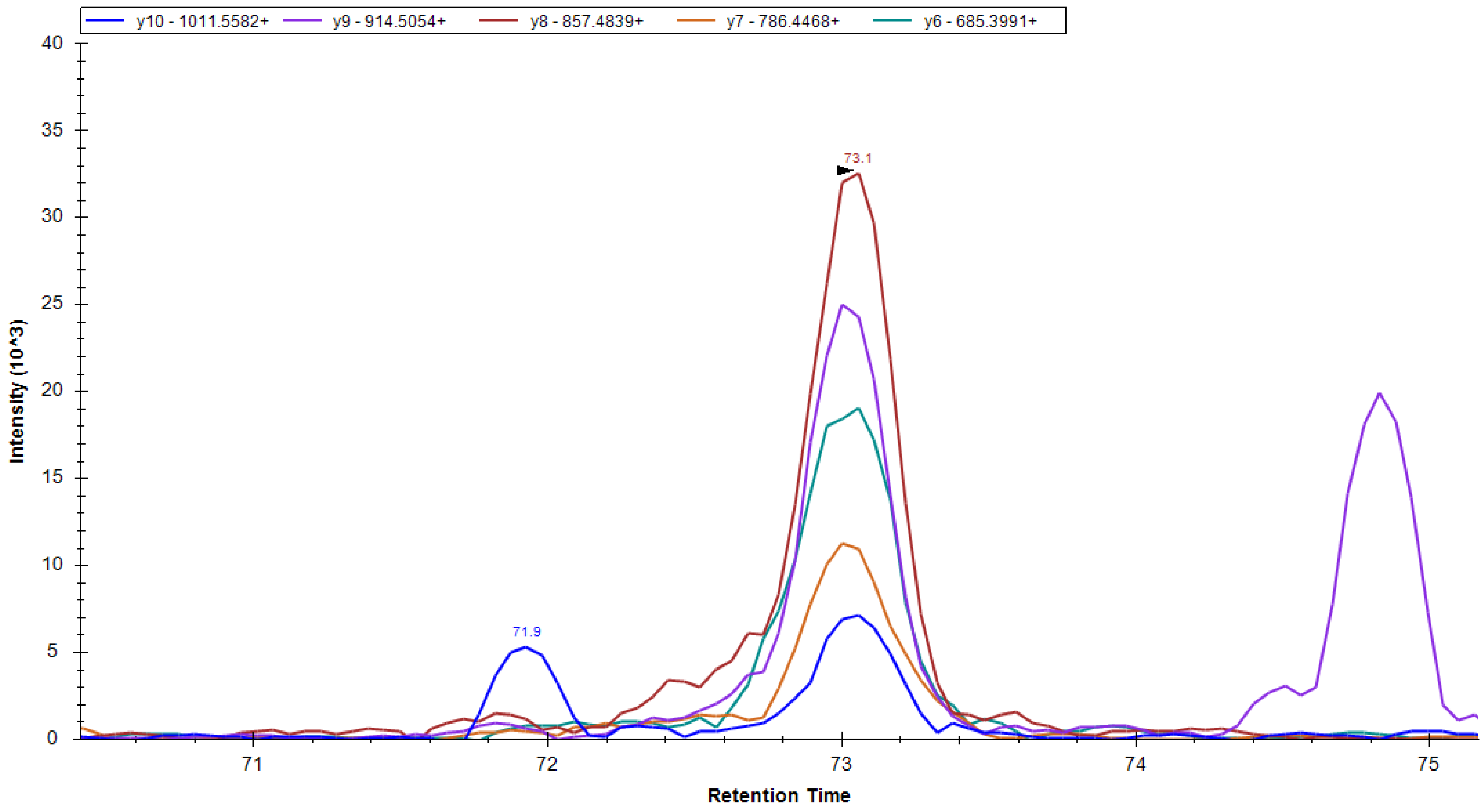

File name: 1604_JinanU(GZ)_MRM_#B_2nd_Round_Screen.skyd

Parent ion m/z and charges: 927.4862++

NR_072977.1.1
SSPVFQIPKNDDIPEQDSLGLSNLQK

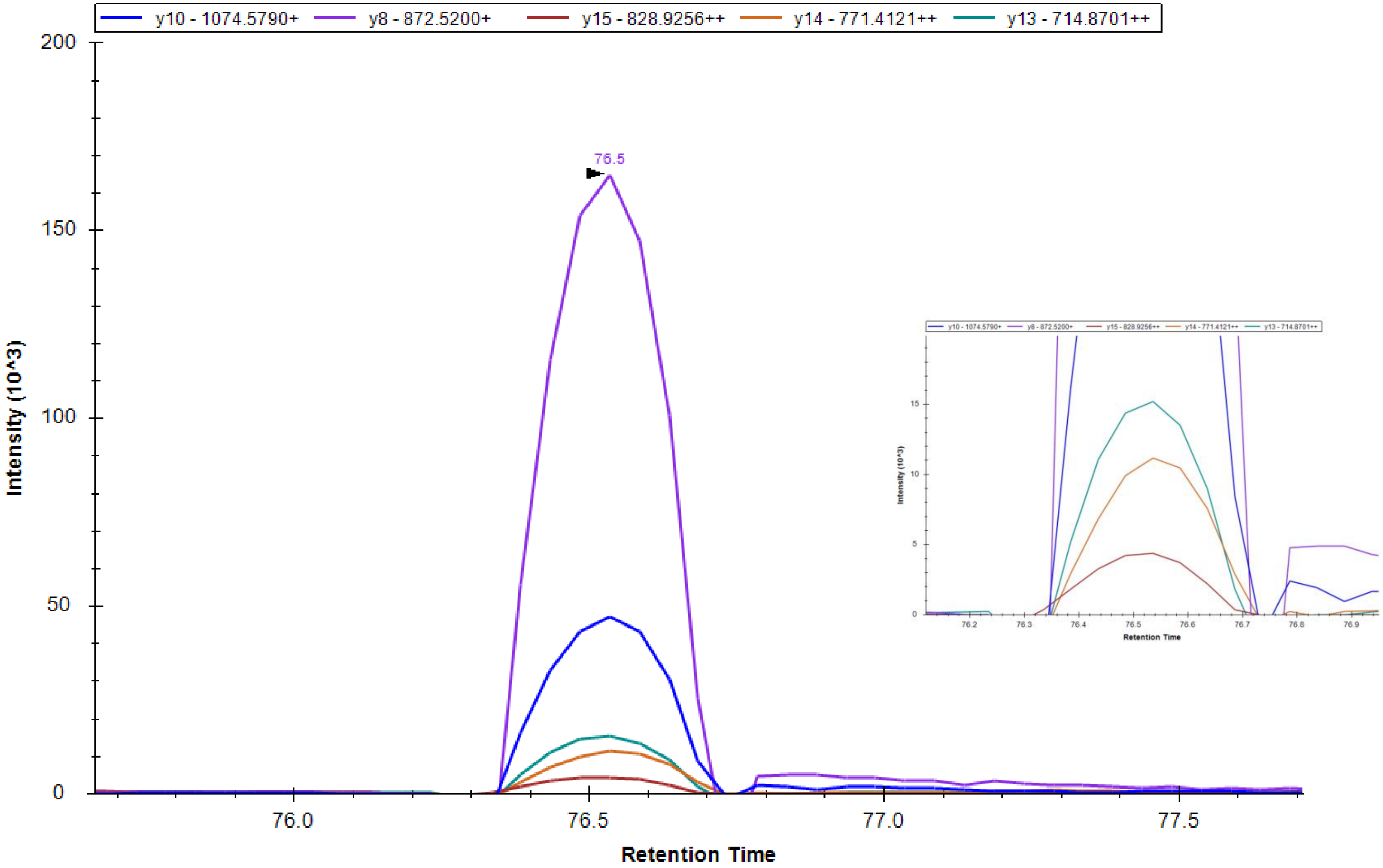

File name: 1604_JinanU(GZ)_MRM_#A_Full-Screen_0421.skyd

Parent ion m/z and charges: 957.1574+++

NR_072977.1.1
NDDIPEQDSLGLSNLQK

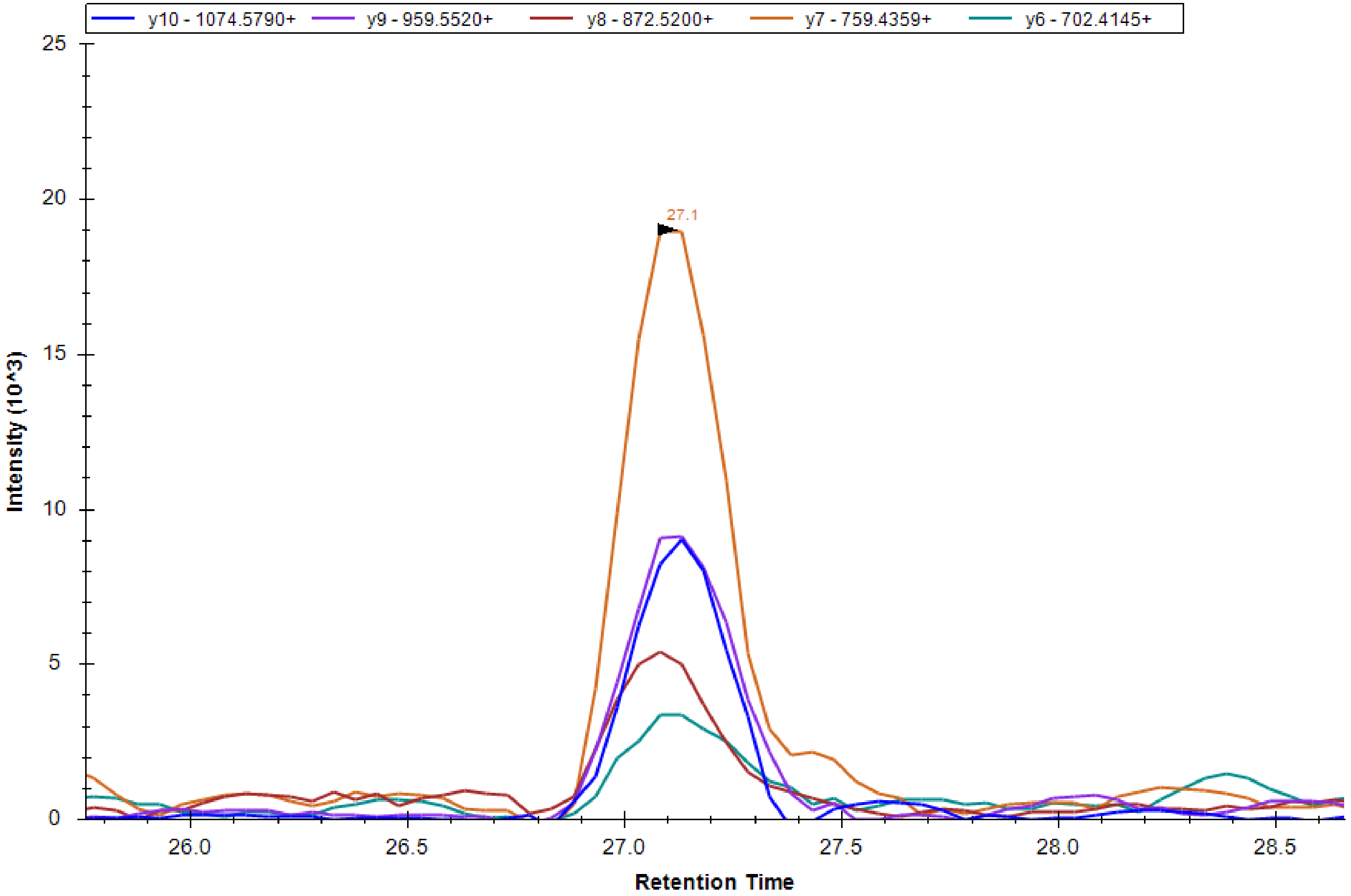

File name: 1604_JinanU(GZ)_MRM_#A_Full-Screen_0421.skyd

Parent ion m/z and charges: 943.4605++

NR_072977.1.1
SSPVFQIPK

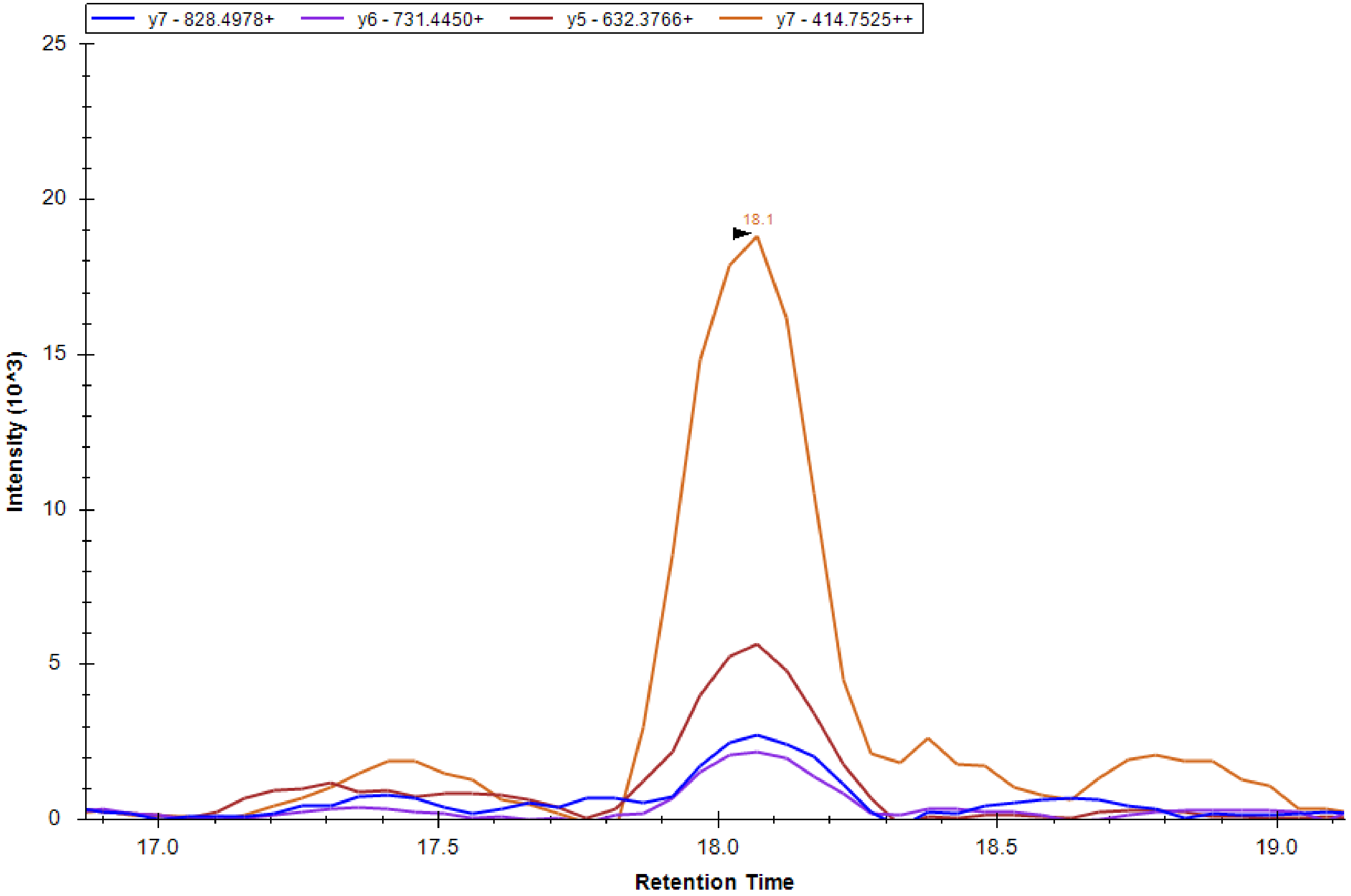

File name: 1604_JinanU(GZ)_MRM_#A_Full-Screen_0421.skyd

Parent ion m/z and charges: 501.7846++

NR_072977.1.1
SSPVFQIPKNDDIPEQDSLGLSNLQK

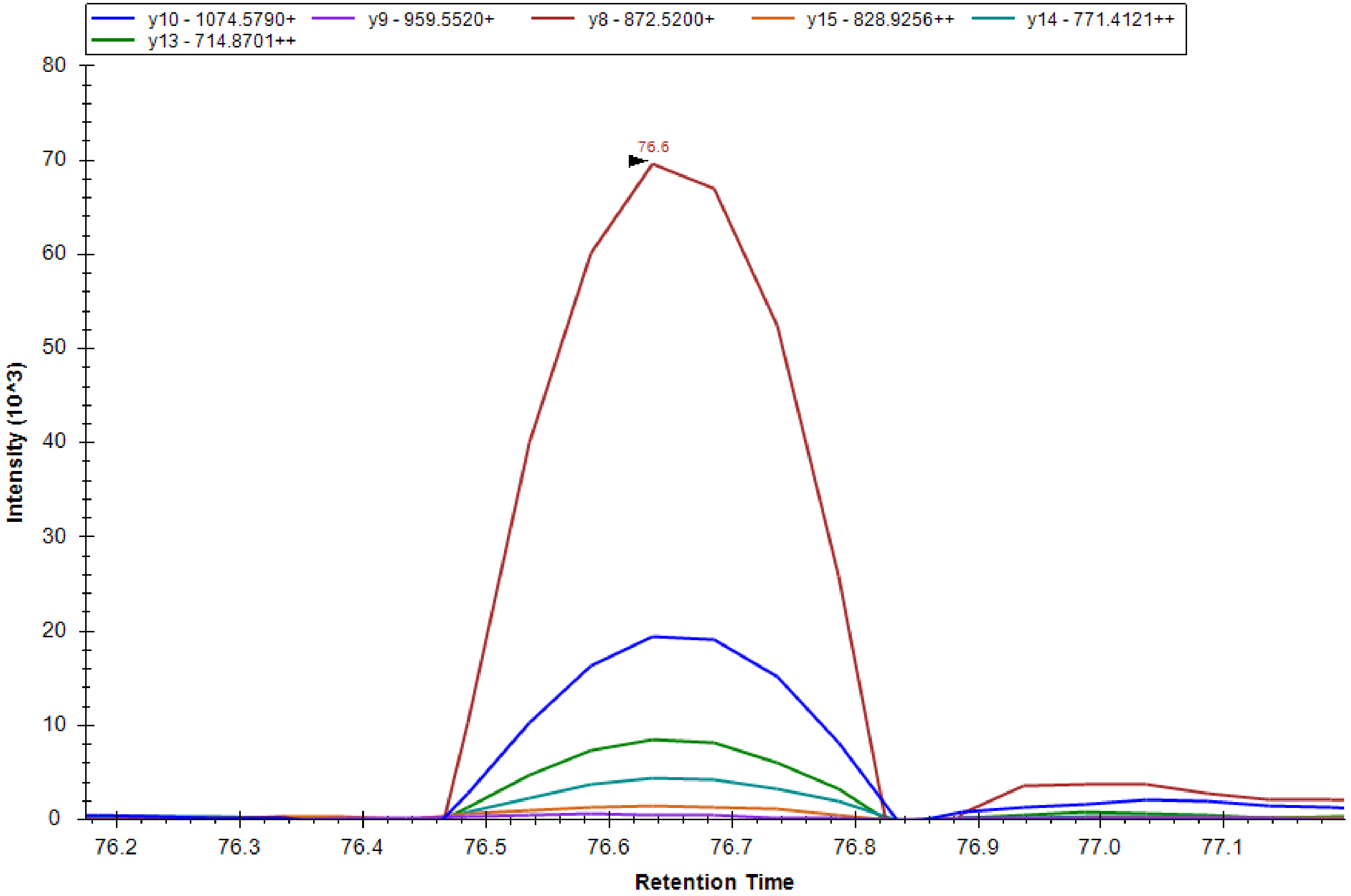

File name: 1604_JinanU(GZ)_MRM_#B_Full-Screen_0421.skyd

Parent ion m/z and charges: 957.1574+++

NR_072977.1.1
NDDIPEQDSLGLSNLQK

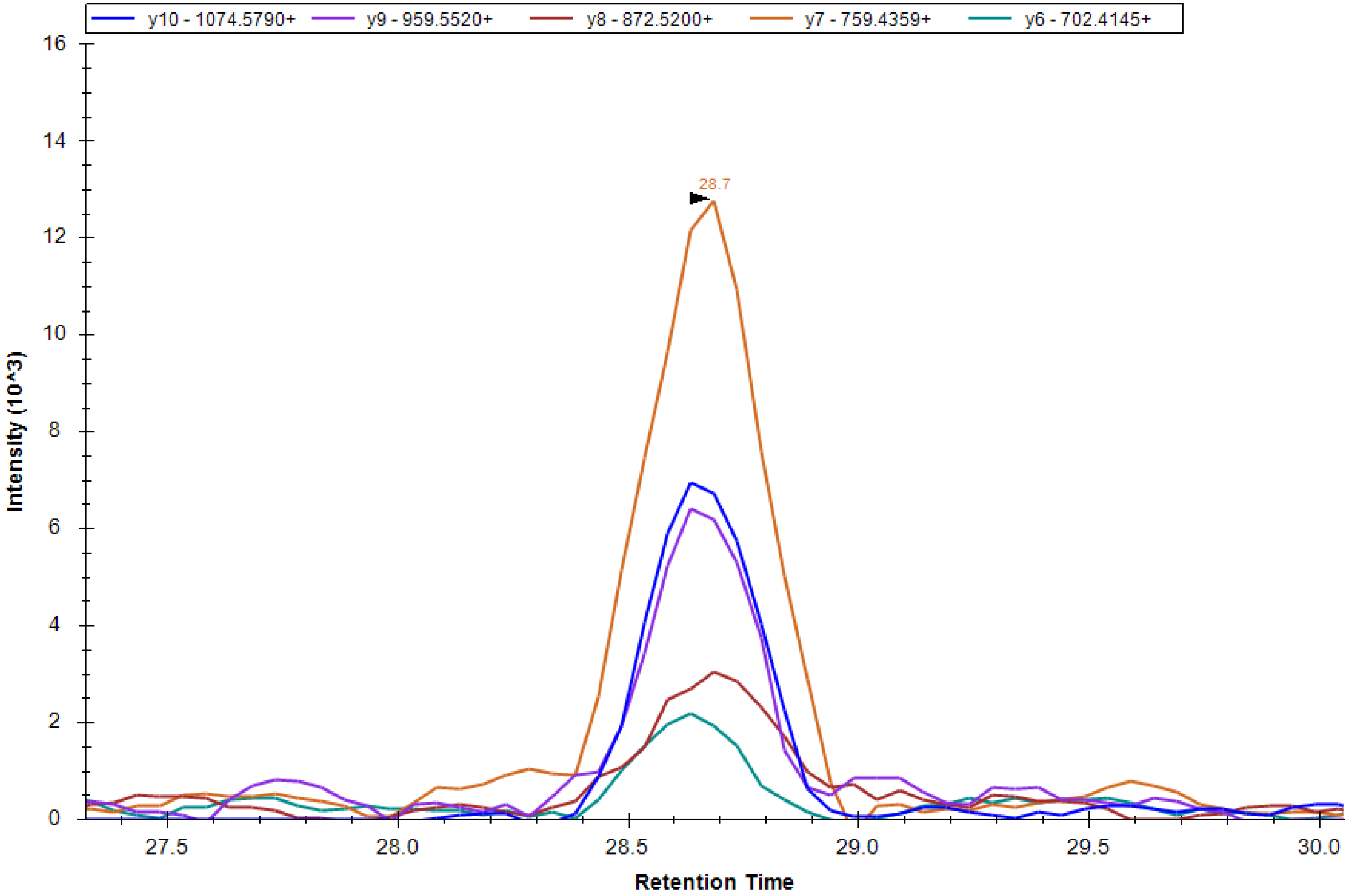

File name: 1604_JinanU(GZ)_MRM_#B_Full-Screen_0421.skyd

Parent ion m/z and charges: 943.4605++

NR_072977.1.1
SSPVFQIPK

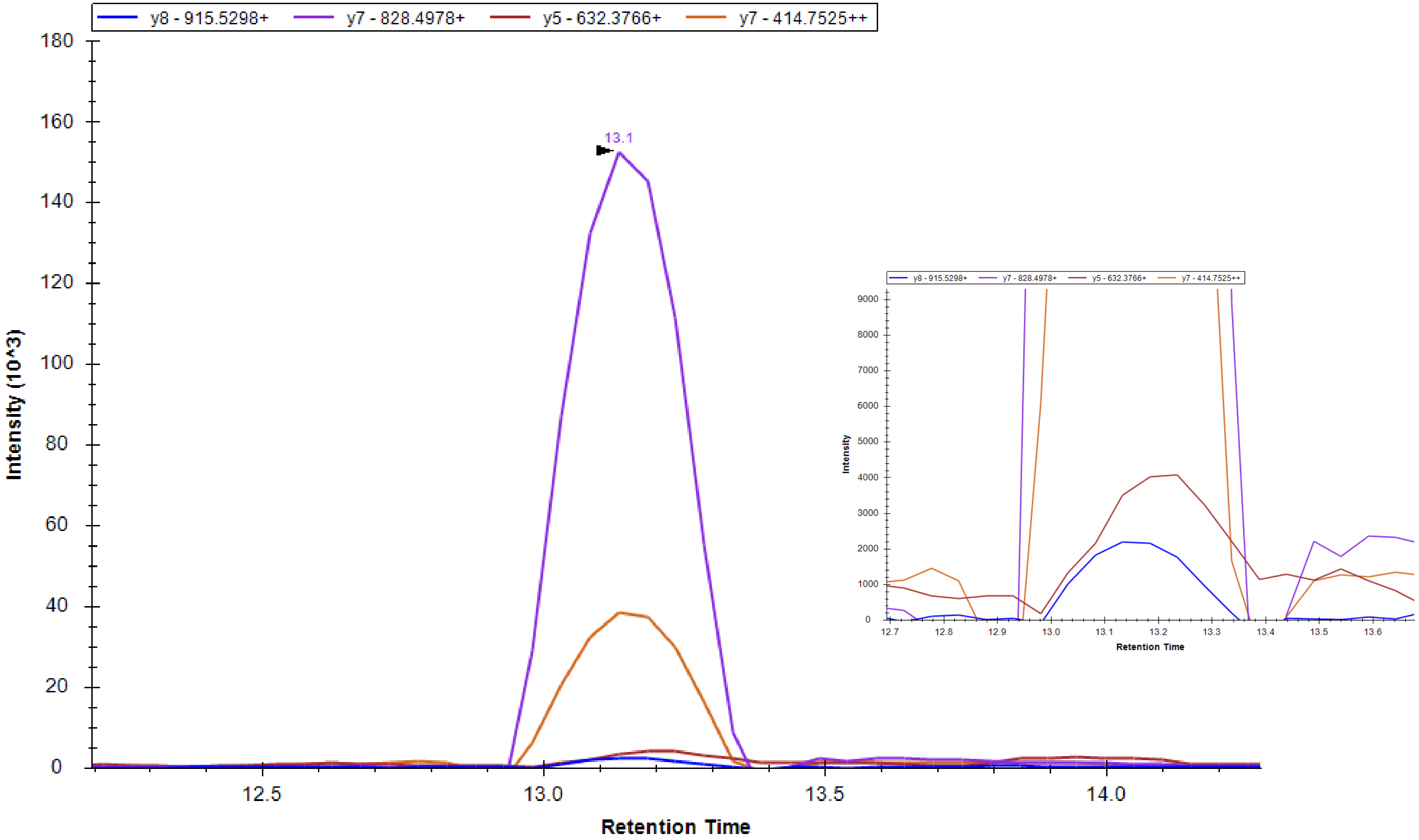

File name: 1604_JinanU(GZ)_MRM_#B_Full-Screen_0421.skyd

Parent ion m/z and charges: 501.7846++

NR_072977.1.1
SSPVFQIPKNDDIPEQDSLGLSNLQK

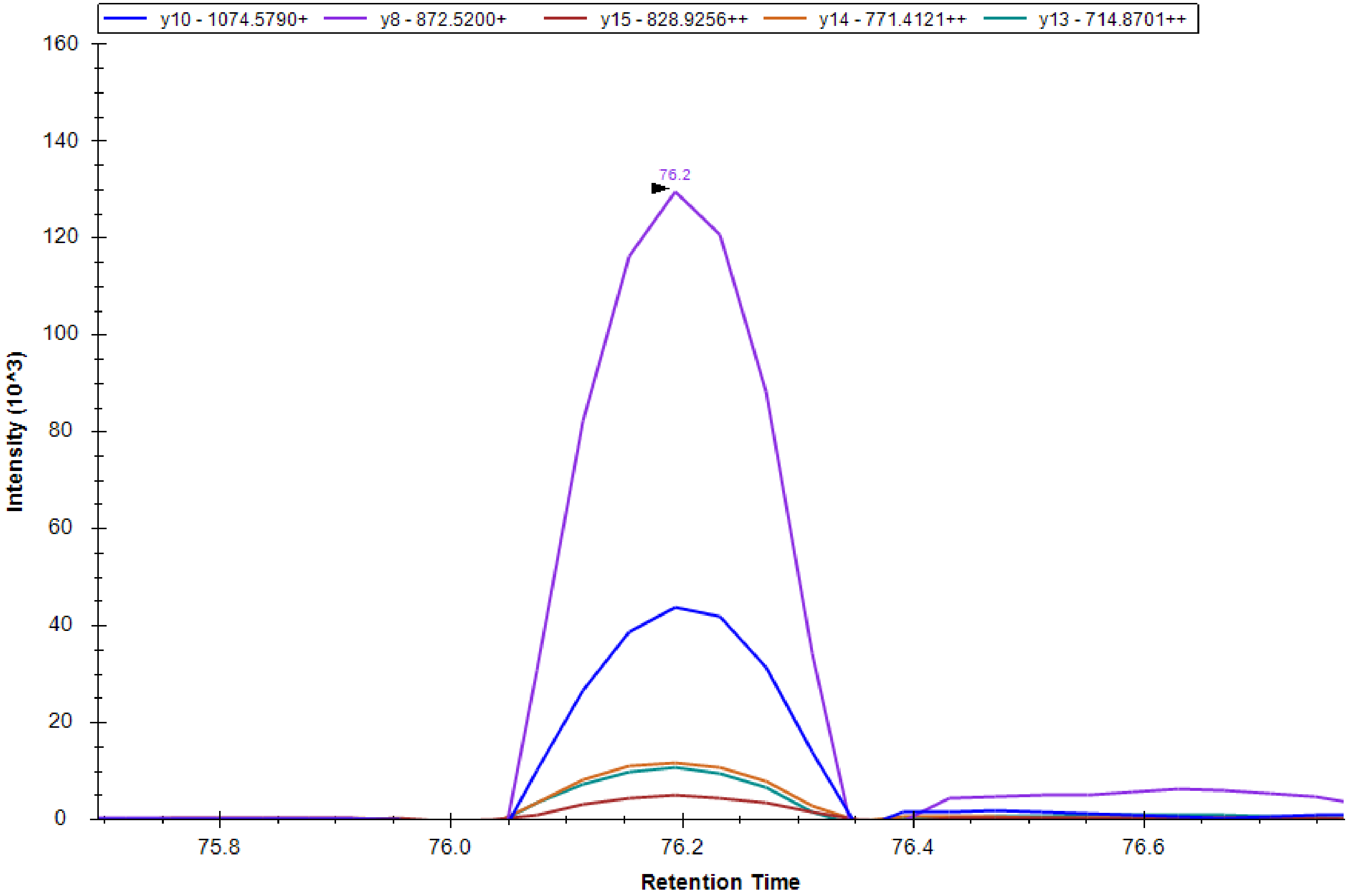

File name: 1604_JinanU(GZ)_MRM_#A_#B_Positive_Screen.skyd

Parent ion m/z and charges: 957.1574+++

NR_072977.1.1
NDDIPEQDSLGLSNLQK

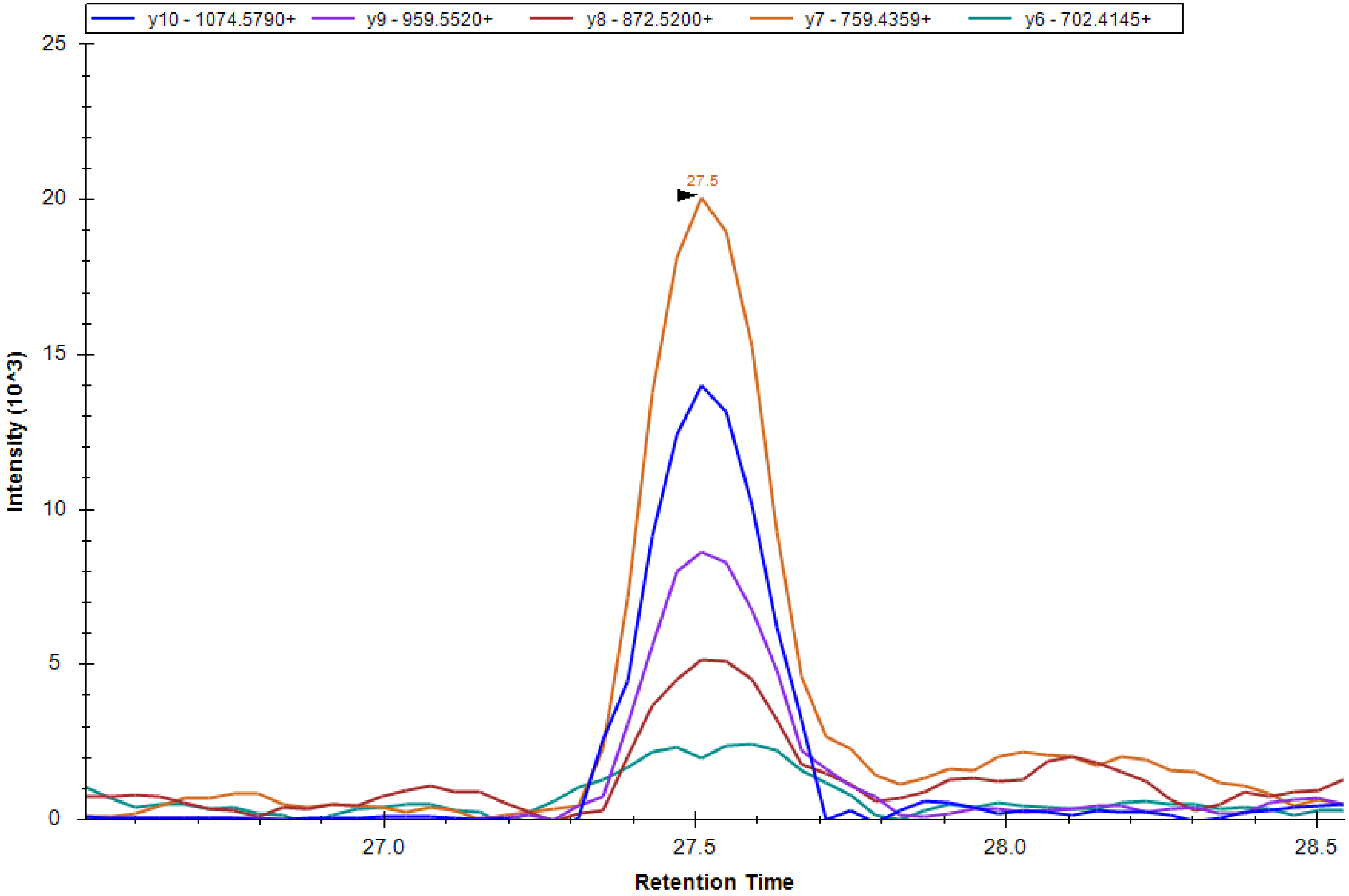

File name: 1604_JinanU(GZ)_MRM_#A_#B_Positive_Screen.skyd

Parent ion m/z and charges: 943.4605++

NR_104292.3.10
MDRFLHGPVPPWTGSAMDRFR

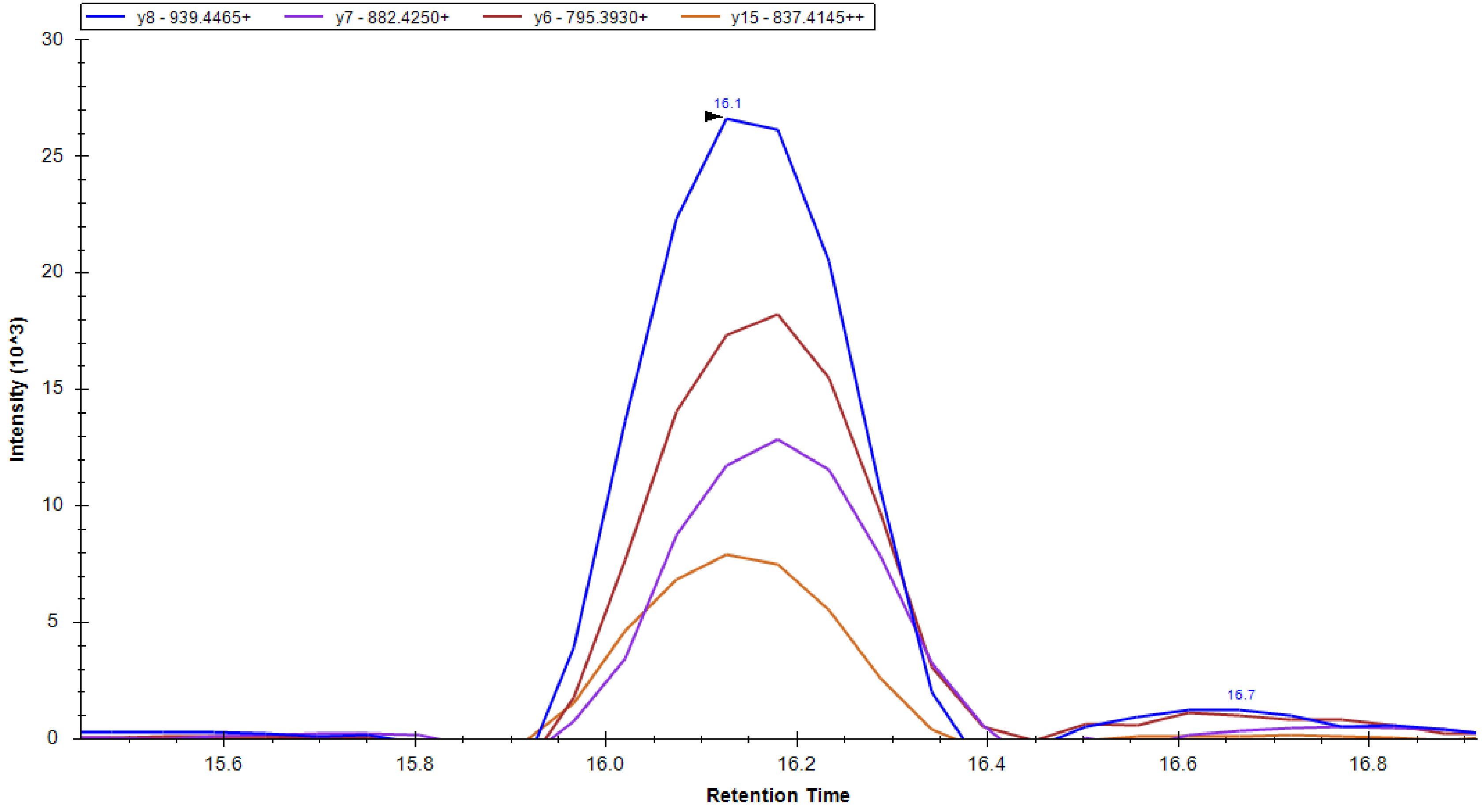

File name: 1604_JinanU(GZ)_MRM_#B_2nd_Round_Screen.skyd

Parent ion m/z and charges: 825.0721+++

NR_110804.2.4
MYALPSIGLKSLK

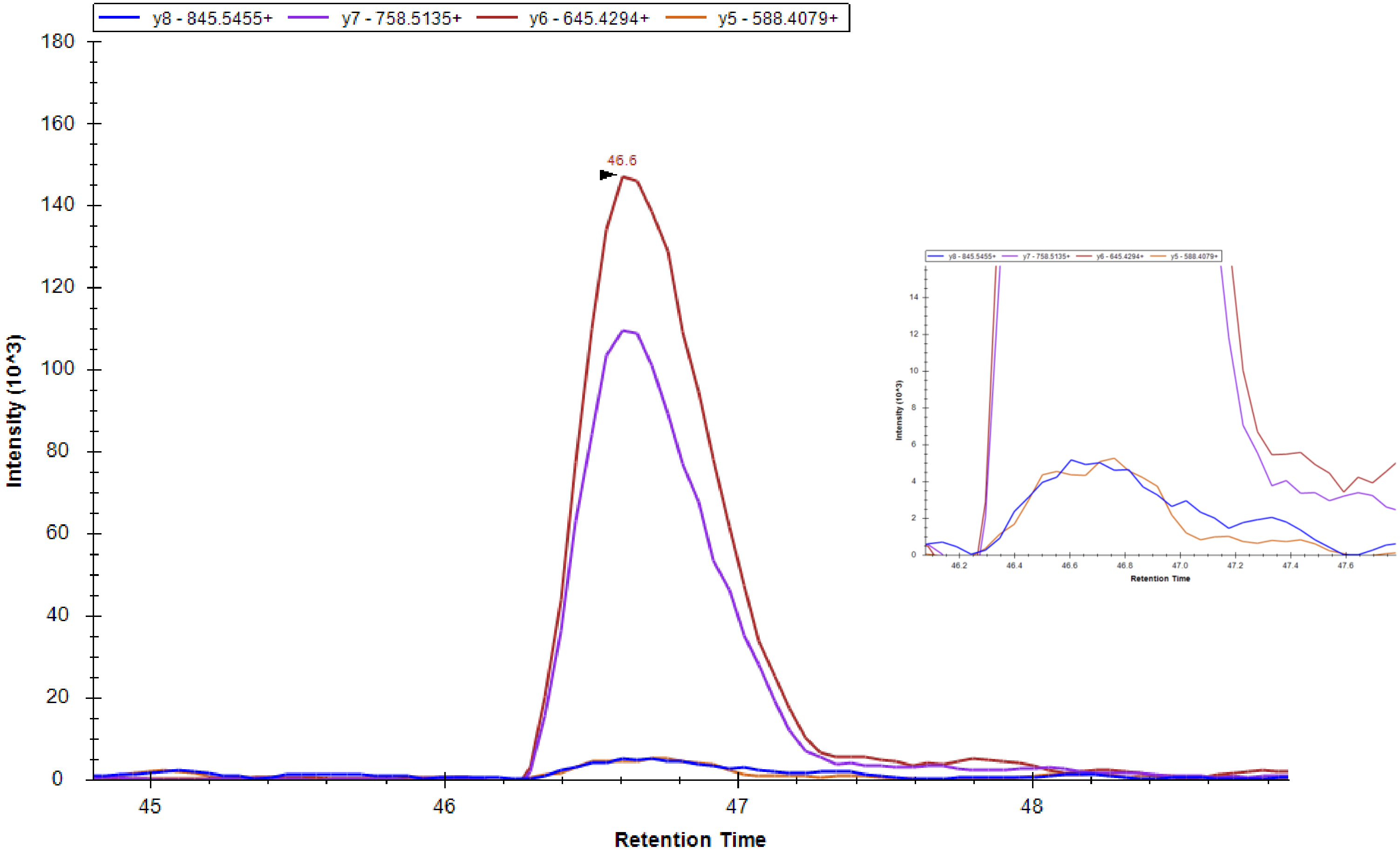

File name: 1604_JinanU(GZ)_MRM_#A_Full-Screen_0421.skyd

Parent ion m/z and charges: 710.9153++

NR_110804.2.4
EVSMDRALVER

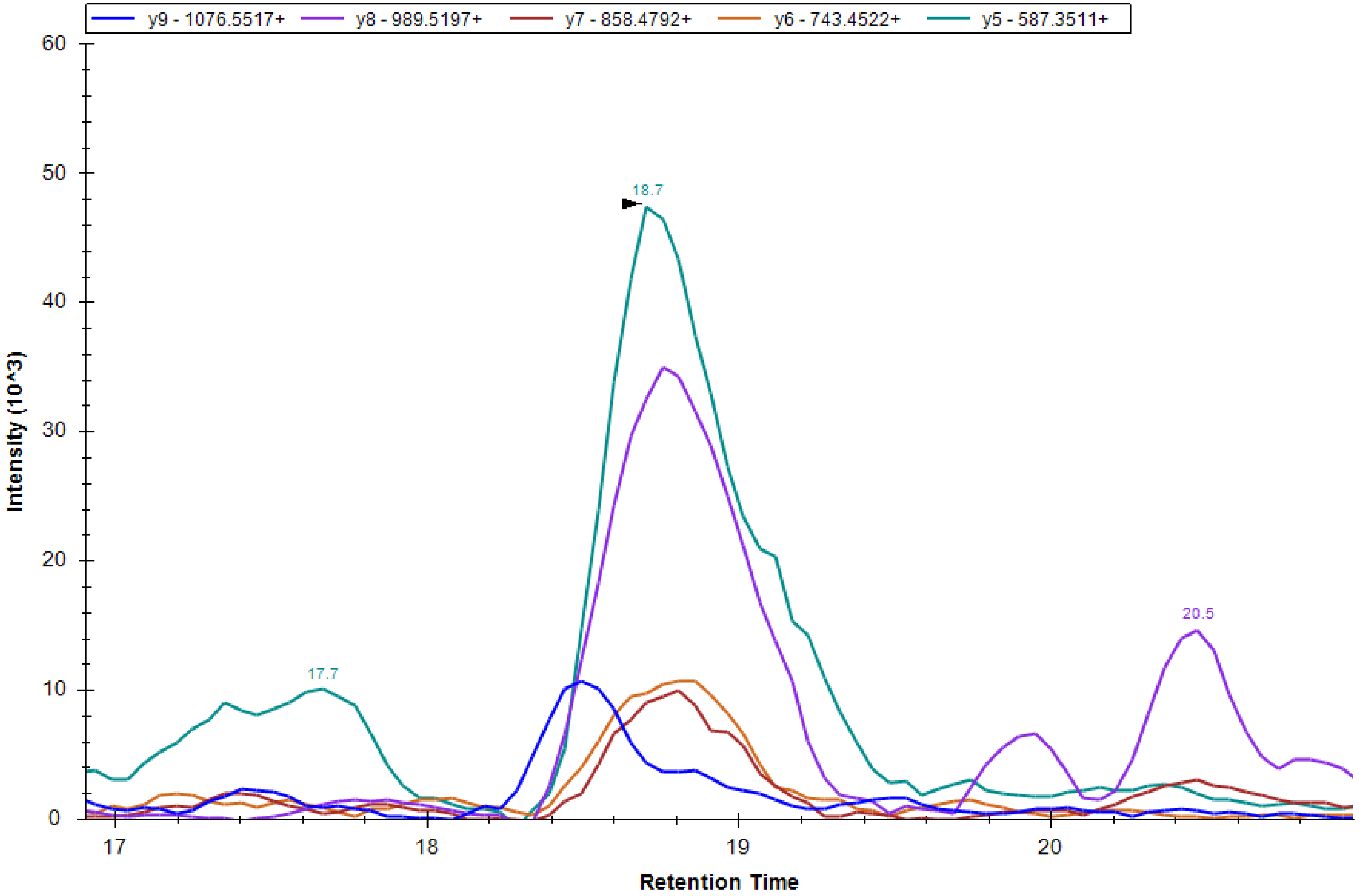

File name: 1604_JinanU(GZ)_MRM_#A_Full-Screen_0421.skyd

Parent ion m/z and charges: 652.8350++

NR_110804.2.4
MYALPSIGLKSLK

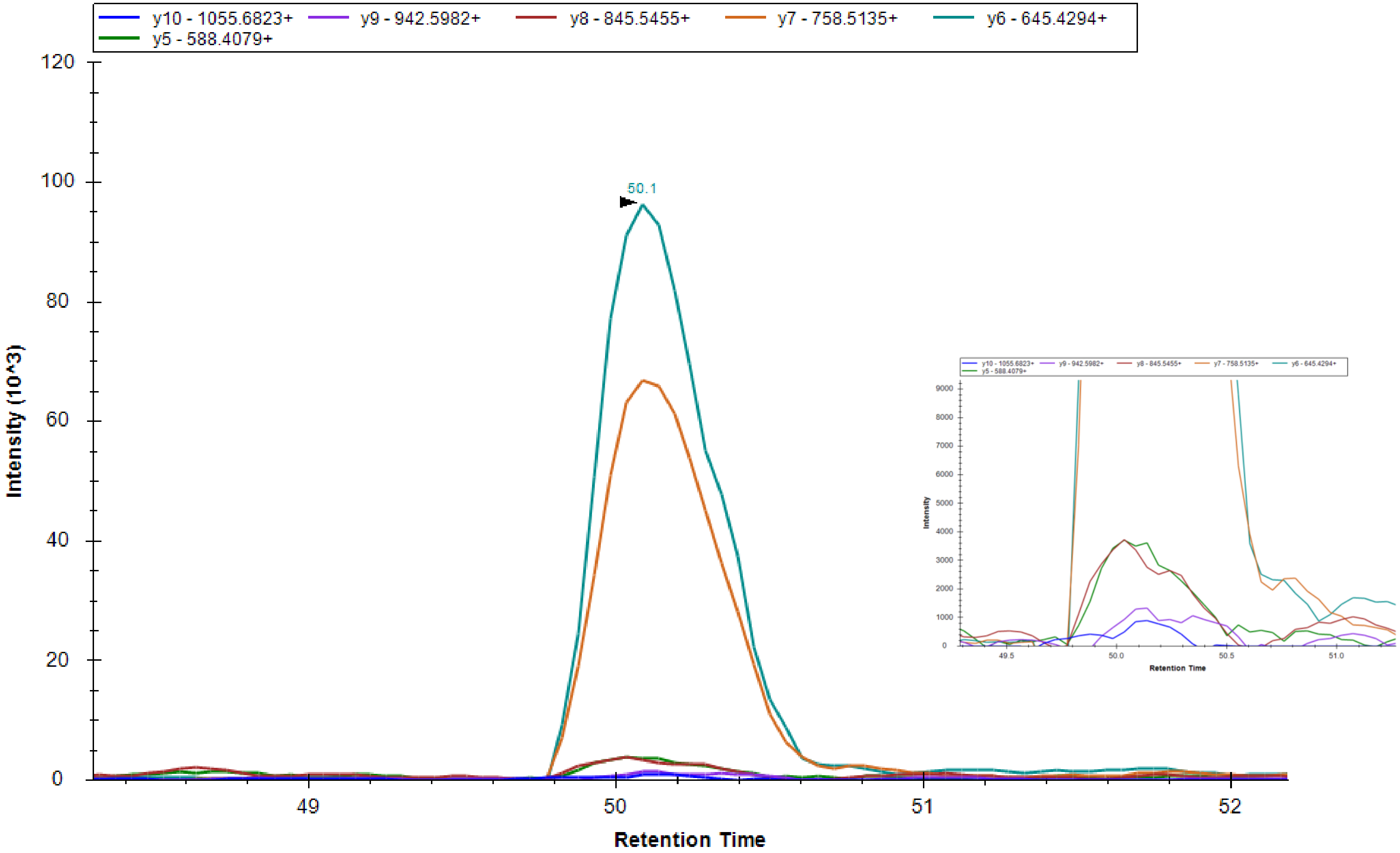

File name: 1604_JinanU(GZ)_MRM_#B_Full-Screen_0421.skyd

Parent ion m/z and charges: 710.9153++

NR_110804.2.4
EVSMDRALVER

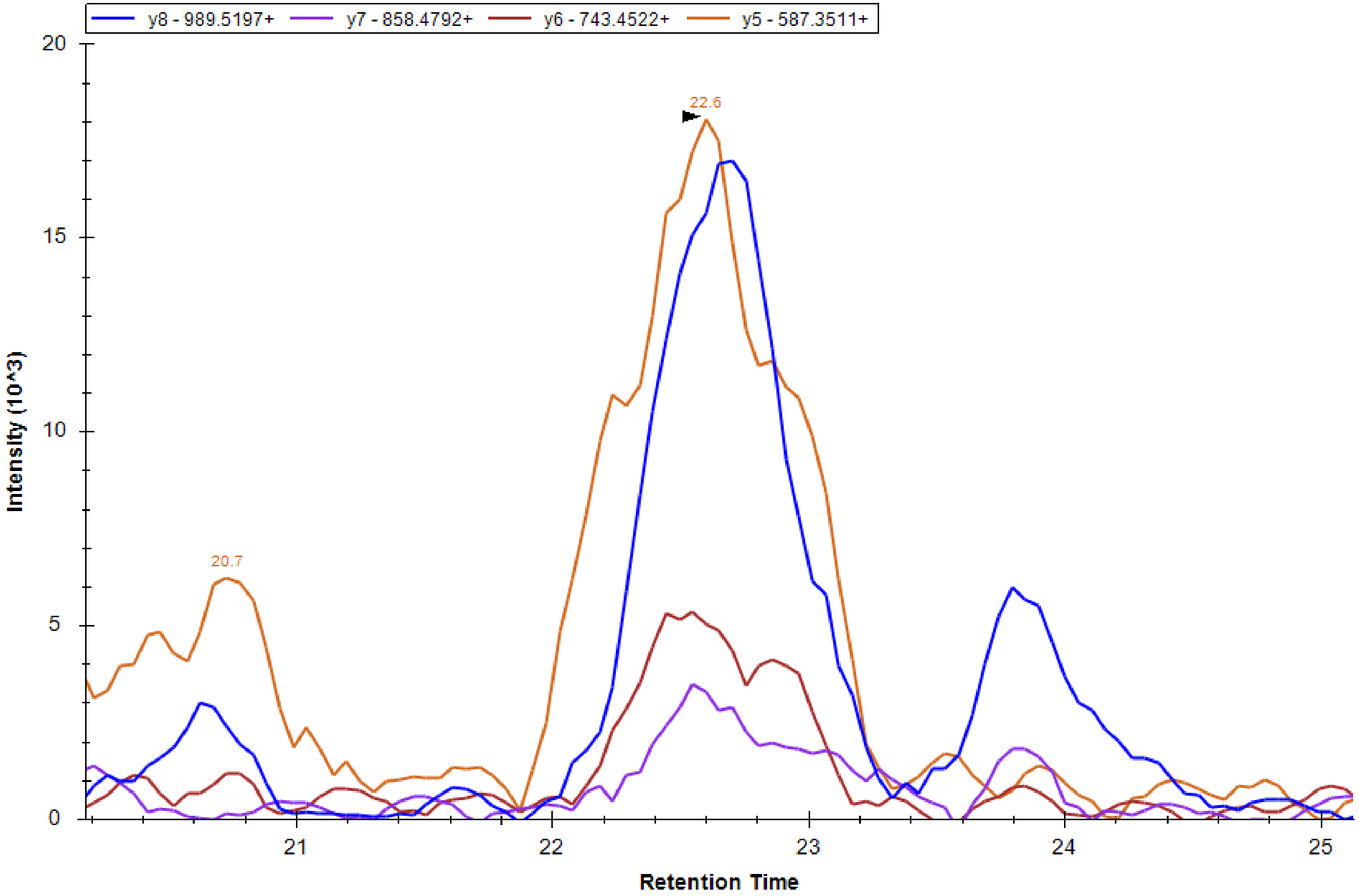

File name: 1604_JinanU(GZ)_MRM_#B_Full-Screen_0421.skyd

Parent ion m/z and charges: 652.8350++

NR_110804.2.4
EVSMDRALVER

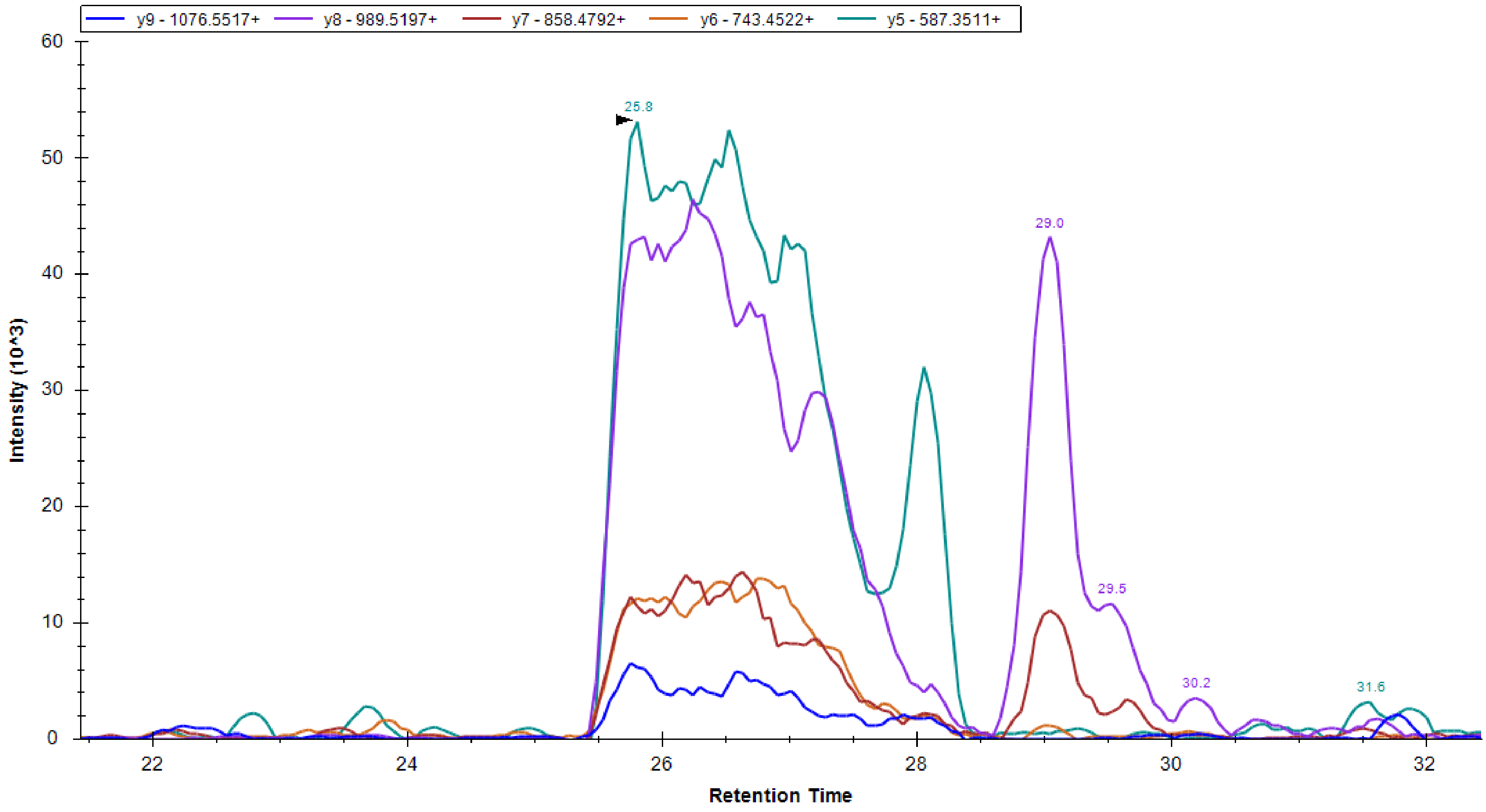

File name: 1604_JinanU(GZ)_MRM_#B_2nd_Round_Screen.skyd

Parent ion m/z and charges: 652.8350++

NR_120386.3.5
MAPIEPGTDSISAMIRK

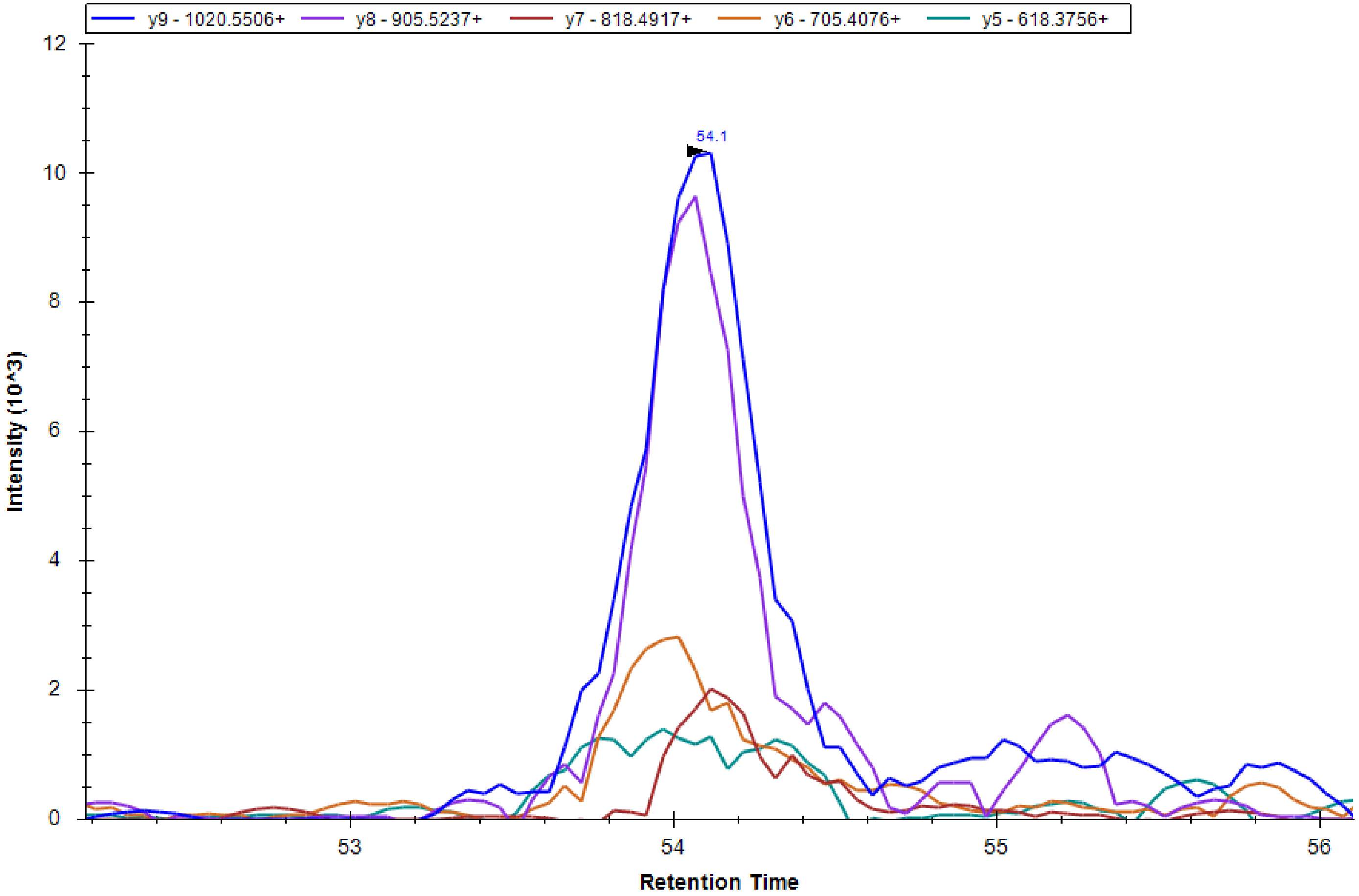

File name: 1604_JinanU(GZ)_MRM_#B_Full-Screen_0421.skyd

Parent ion m/z and charges: 908.9684++

NR_120386.3.5
APLEPGTDSLSAMLRK

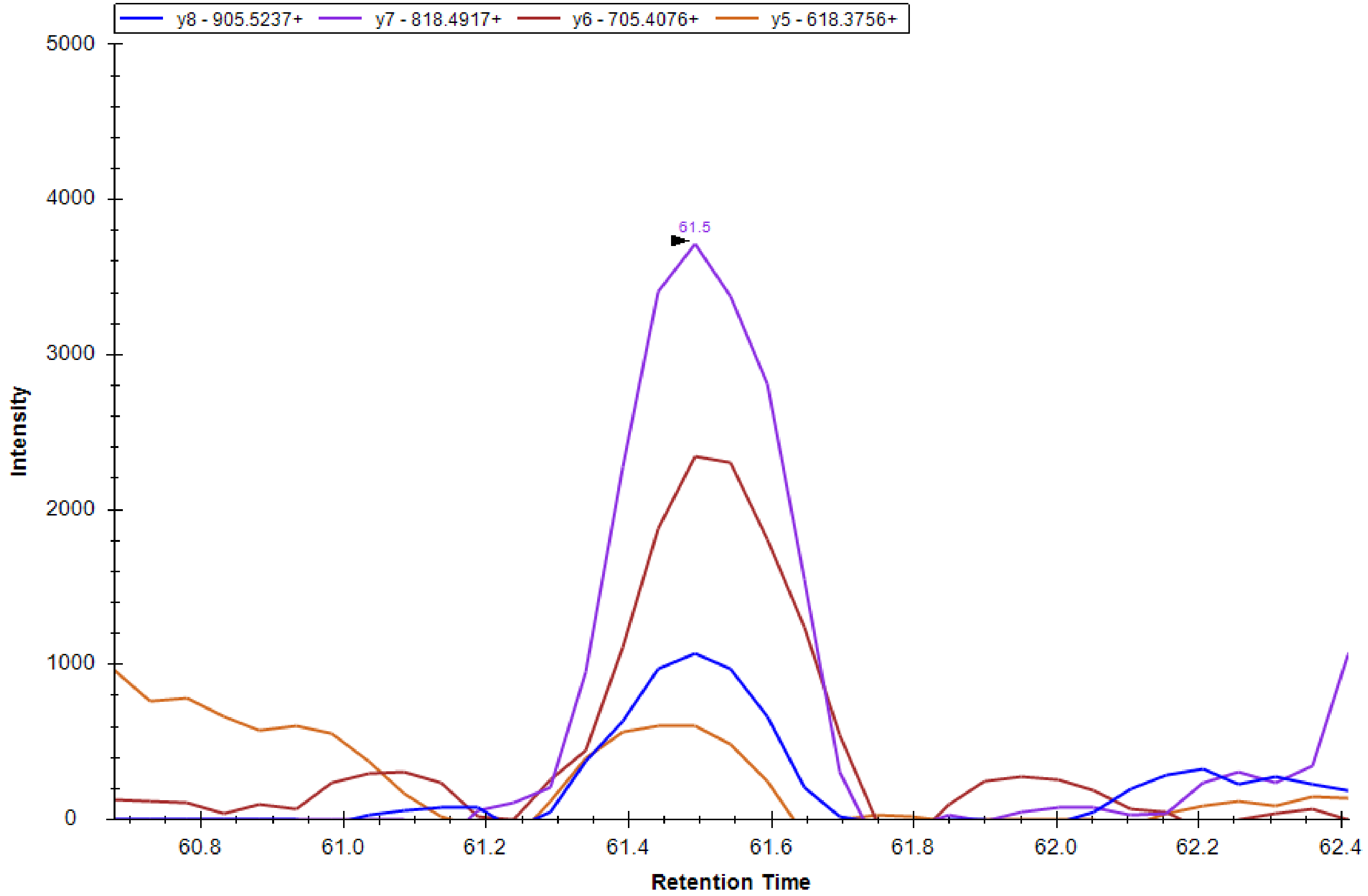

File name: 1604_JinanU(GZ)_MRM_#B_Full-Screen_0421.skyd

Parent ion m/z and charges: 843.4482++

NR_001282.3.5
TKPPPSPKPPTPCSSAR

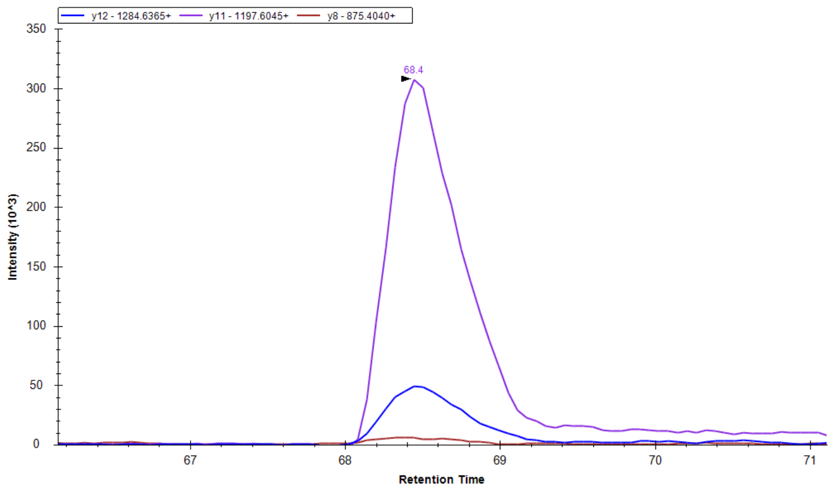

File name: 391-new#B-Round01_All-screening_Positive result.skyd

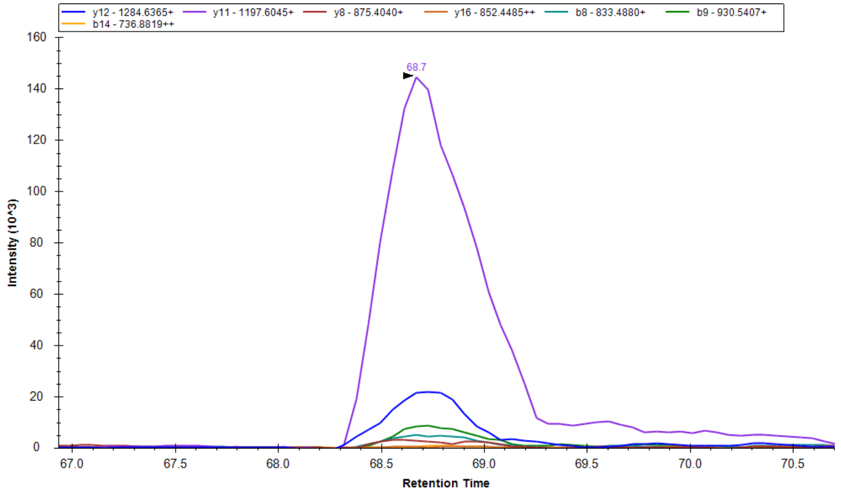

File name: 391-new#B-Round01_Positive-confirm.skyd

Parent ion m/z and charges: 902.9724++

NR_002144.2.1
EIEAIFGQPVVDR

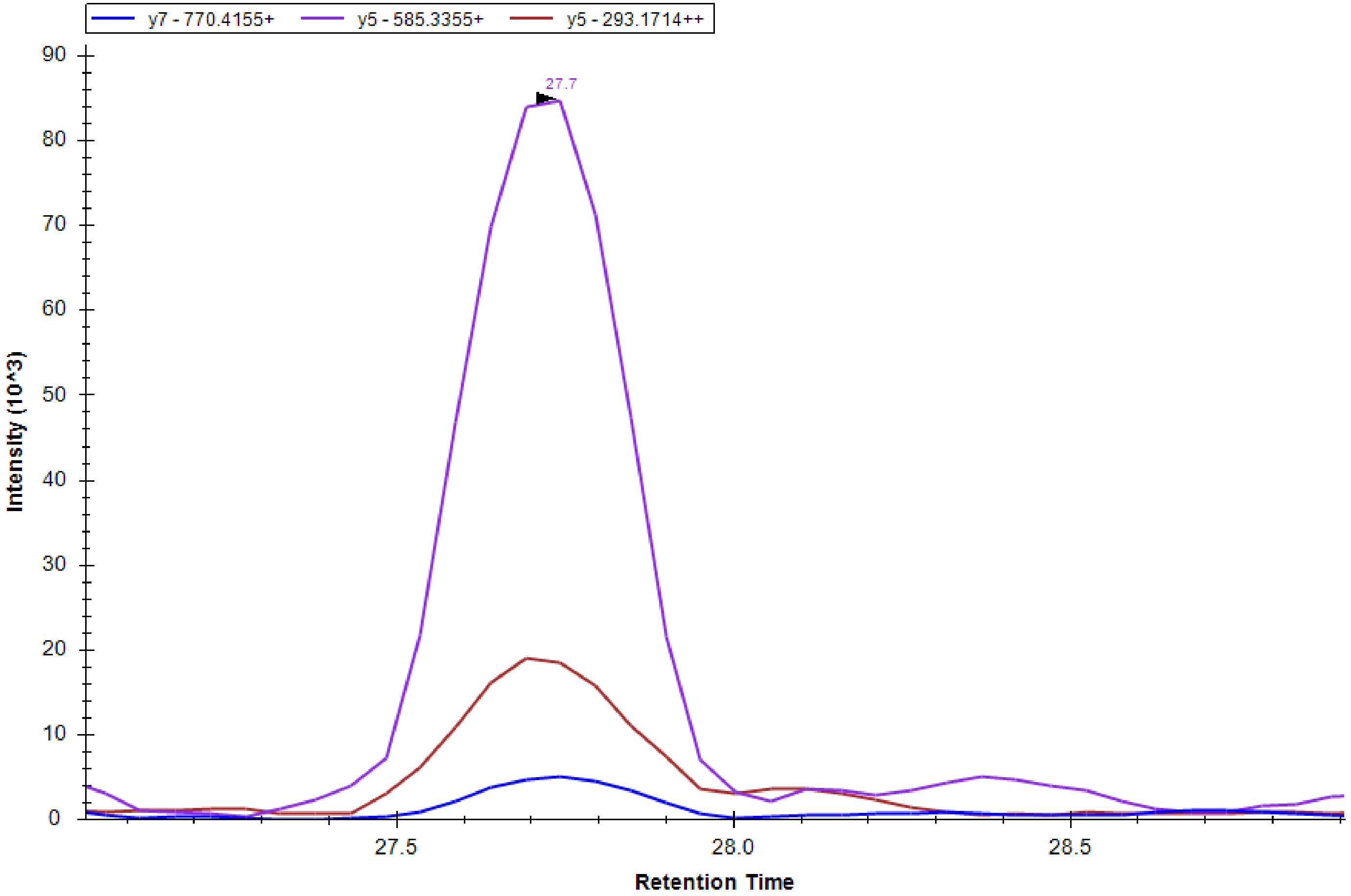

File name: 1604_JinanU(GZ)_MRM_#A_Full-Screen_0421.skyd

Parent ion m/z and charges: 736.8908++

NR_002144.2.1
EIEAIFGQPVVDR

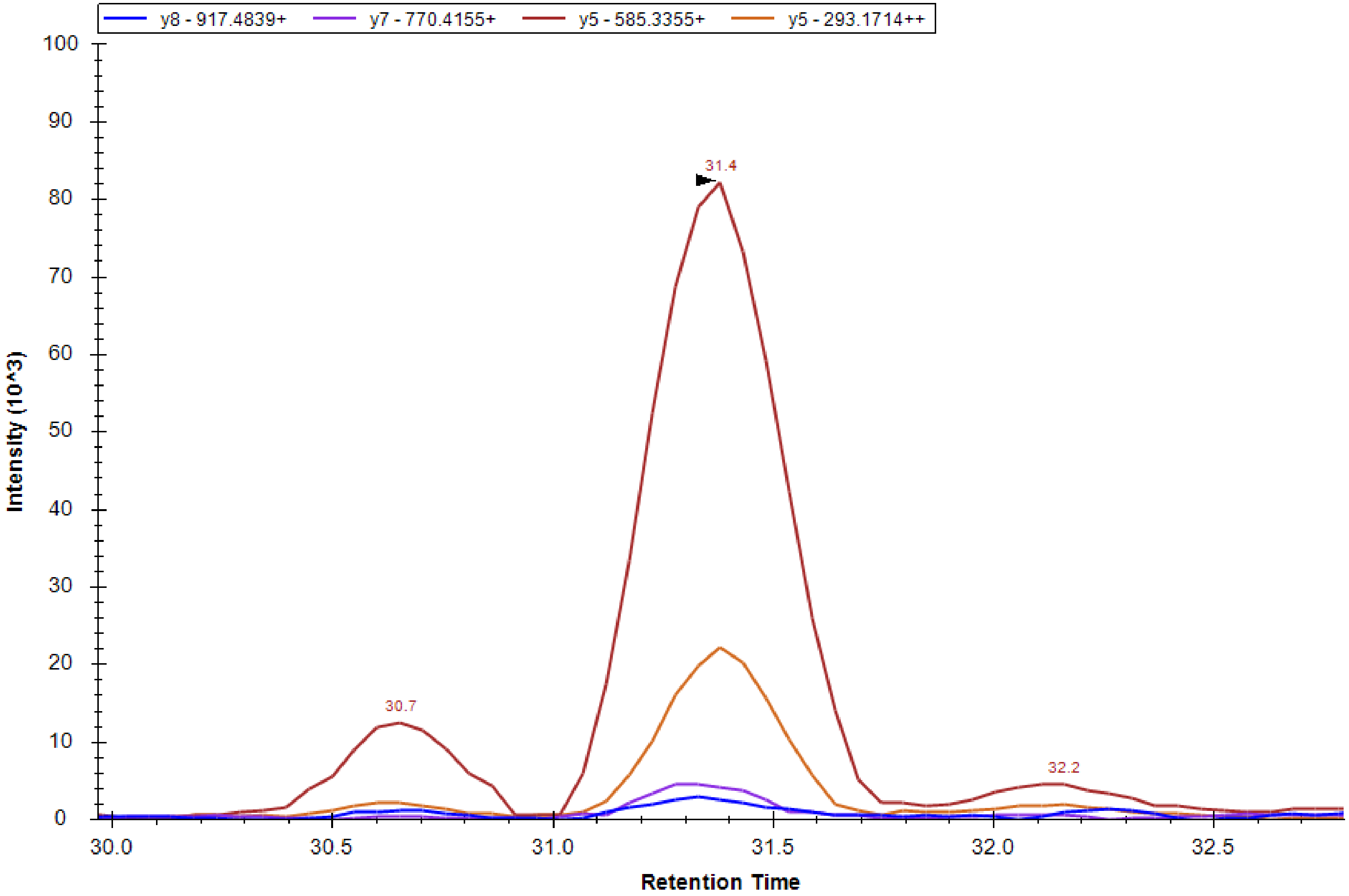

File name: 1604_JinanU(GZ)_MRM_#B_Full-Screen_0421.skyd

Parent ion m/z and charges: 736.8908++

NR_002144.2.1
EIEAIFGQPVVDR

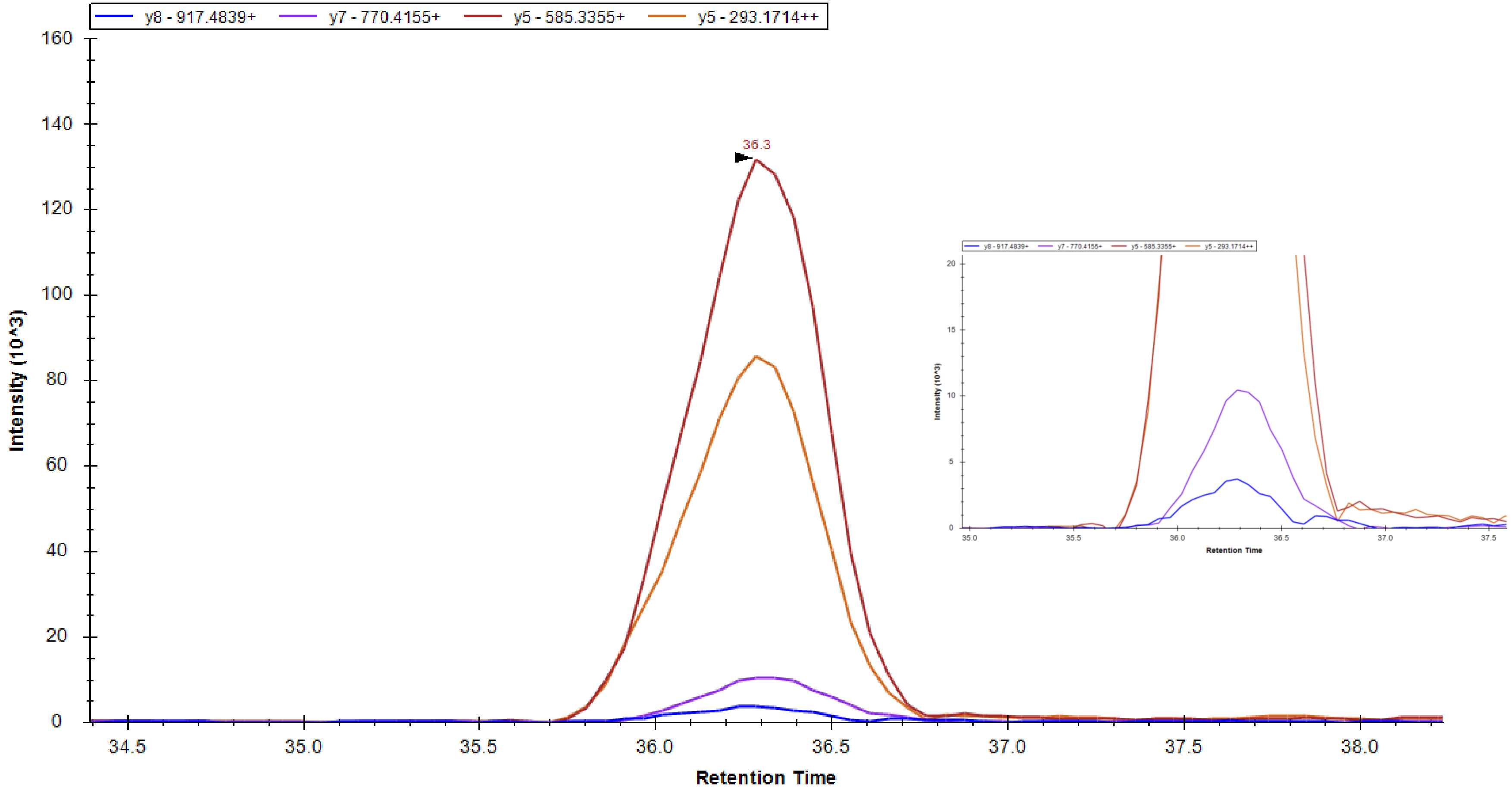

File name: 1604_JinanU(GZ)_MRM_#B_2nd_Round_Screen.skyd

Parent ion m/z and charges: 736.8908++

NR_003662.1.2
WEPRLLVVTDPRADHQPLTEASYVNLPTIALCNTDSPLR

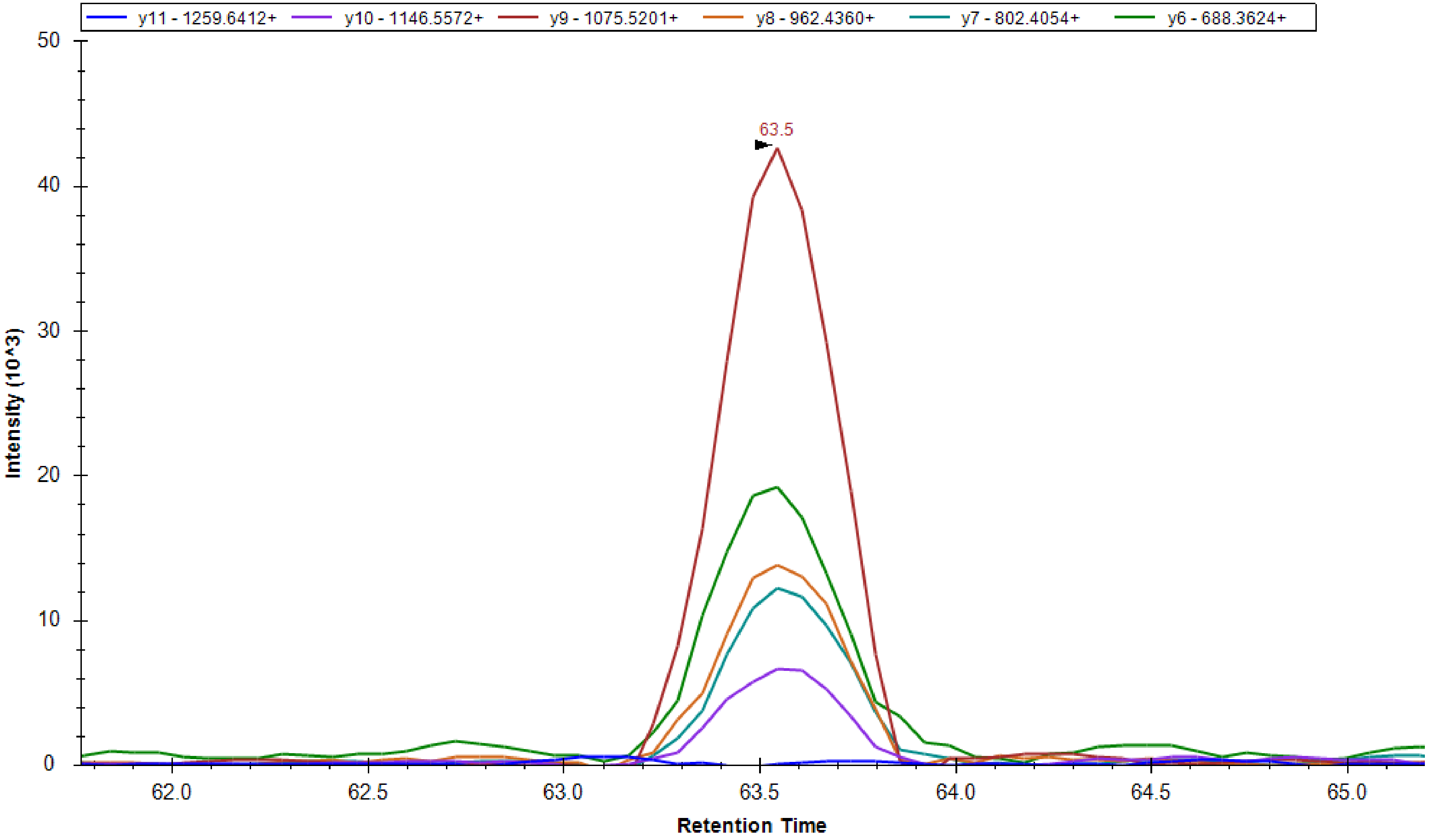

File name: 391-new#A-Round02_Negative-screen.skyd

Parent ion m/z and charges: 1115.3273++++

NR_003662.1.2
WEPRLLVVTDPRADHQPLTEASYVNLPTIALCNTDSPLR

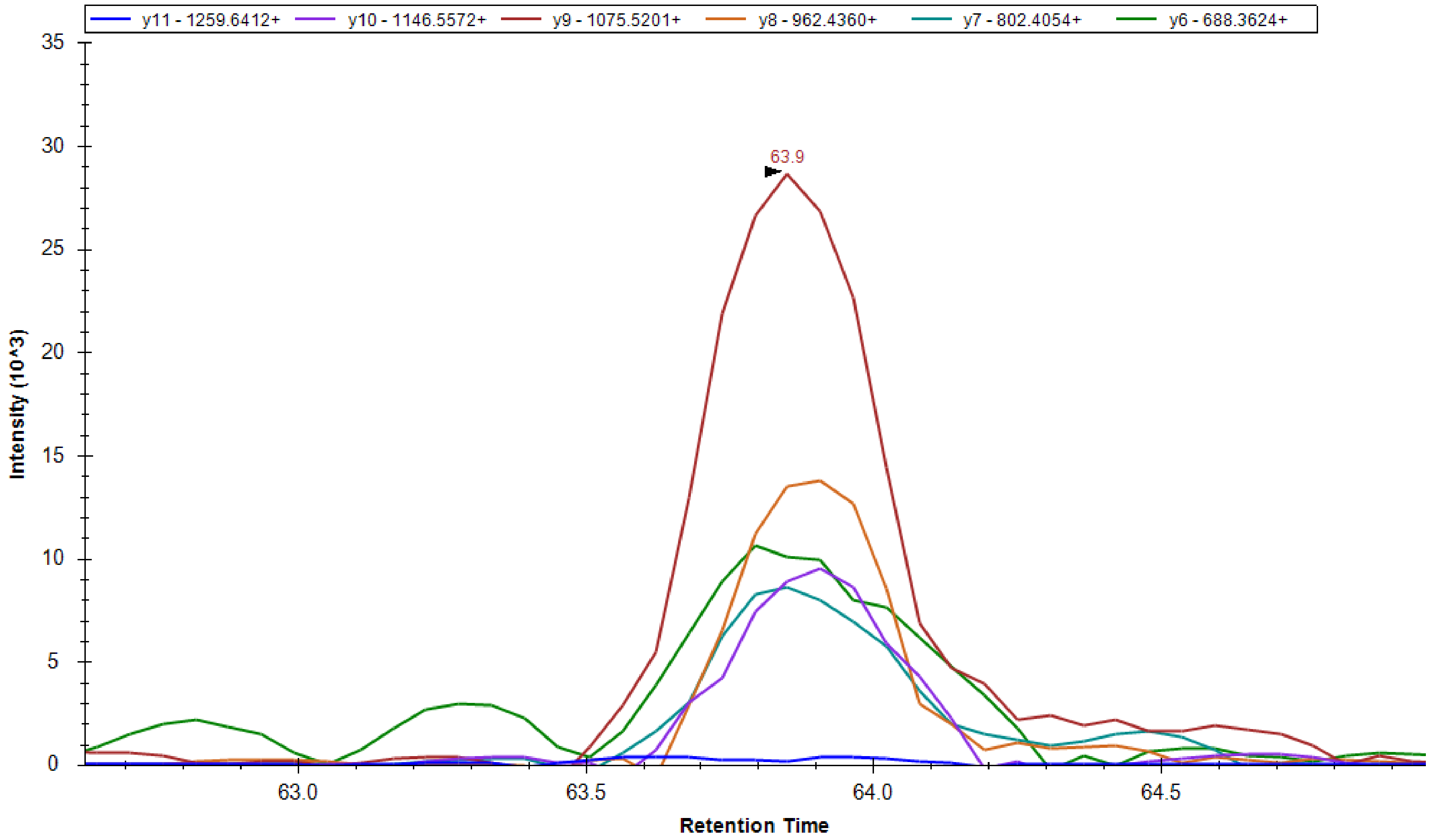

File name: 391-new#B-Round02_Negative-screen.skyd

Parent ion m/z and charges: 1115.3273++++

NR_004855.1.1
TVIQNAYVSIEETIK

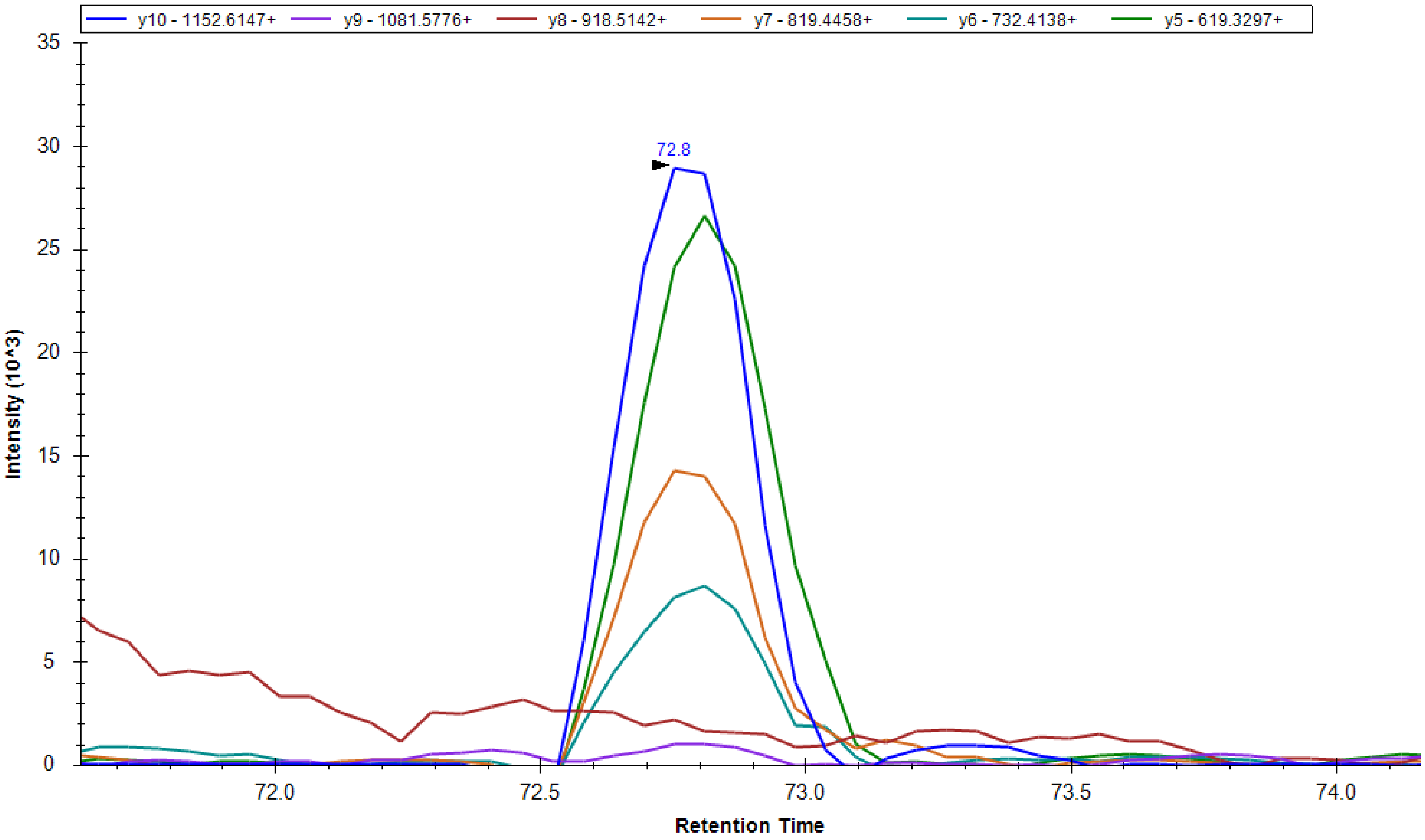

File name: 391-new#B-Round02_Negative-screen.skyd

Parent ion m/z and charges: 854.4618++

NR_004855.1.1
TVIQNAYVSIEETIK

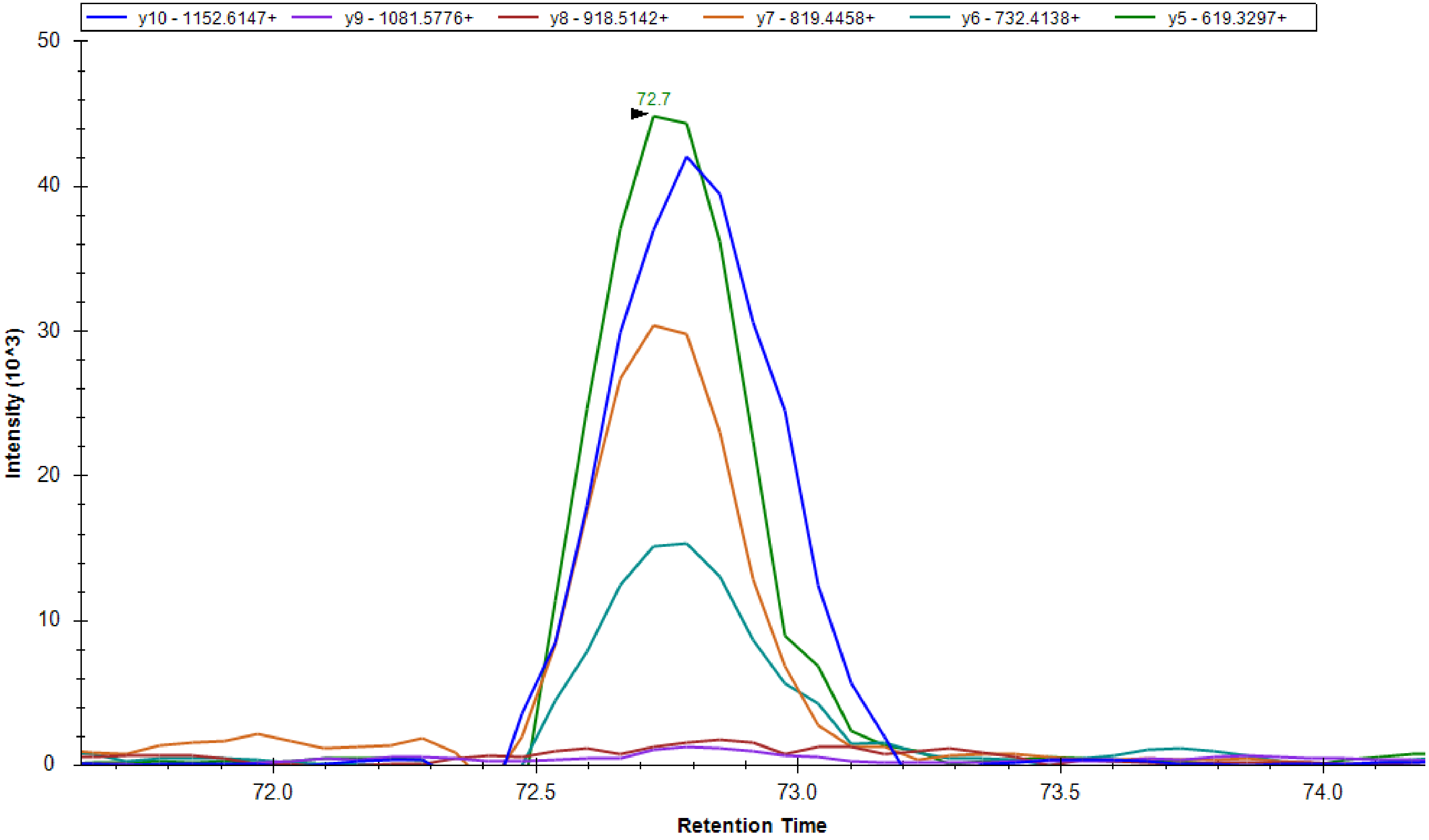

File name: 391-new#A-Round02_Negative-screen.skyd

Parent ion m/z and charges: 854.4618++

NR_015377.3.7
METSGVTGSLK

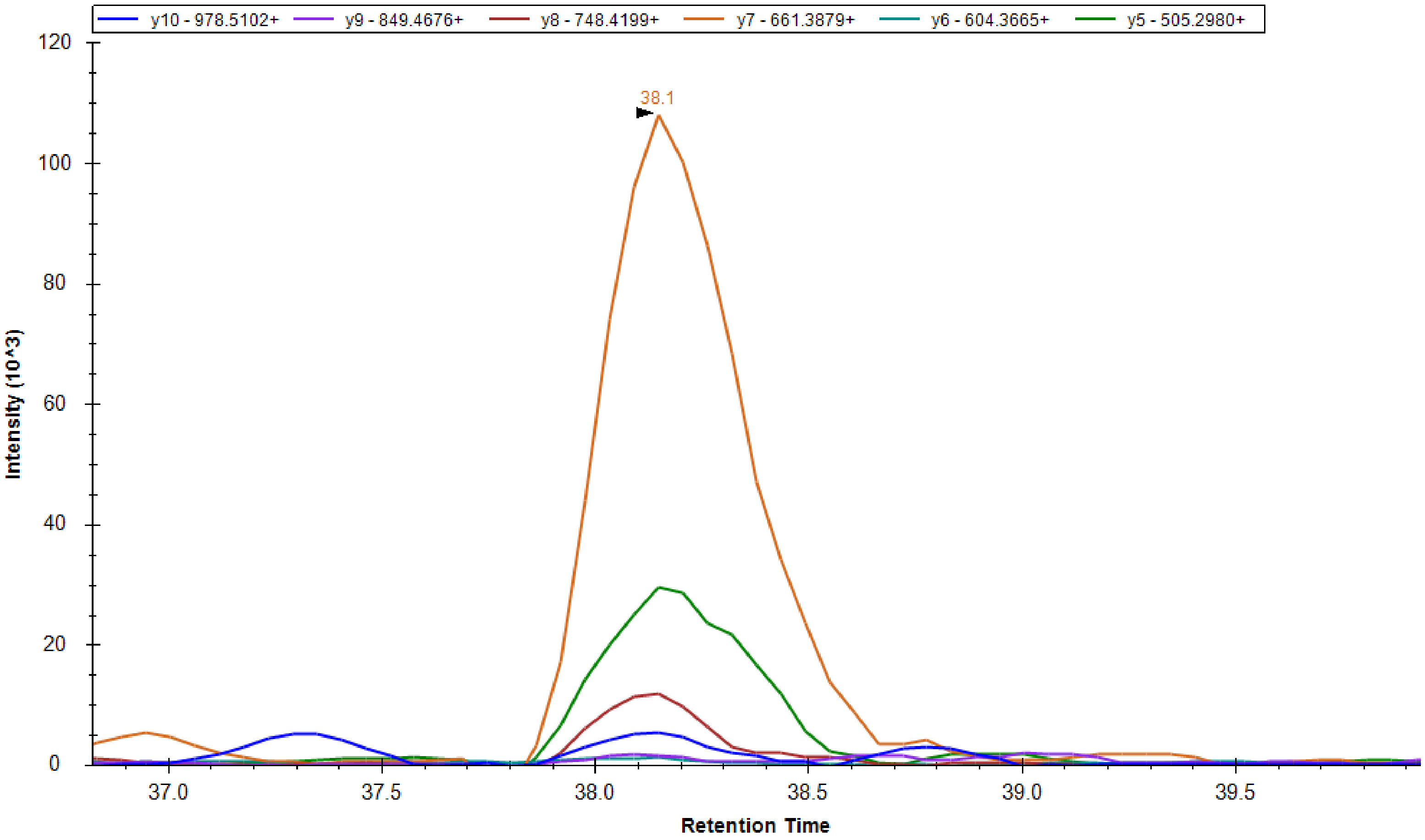

File name: 391-new#B-Round02_Negative-screen.skyd

Parent ion m/z and charges: 555.2790++

NR_015380.3.1
ACWGHNHVFITTQTSSPFDR

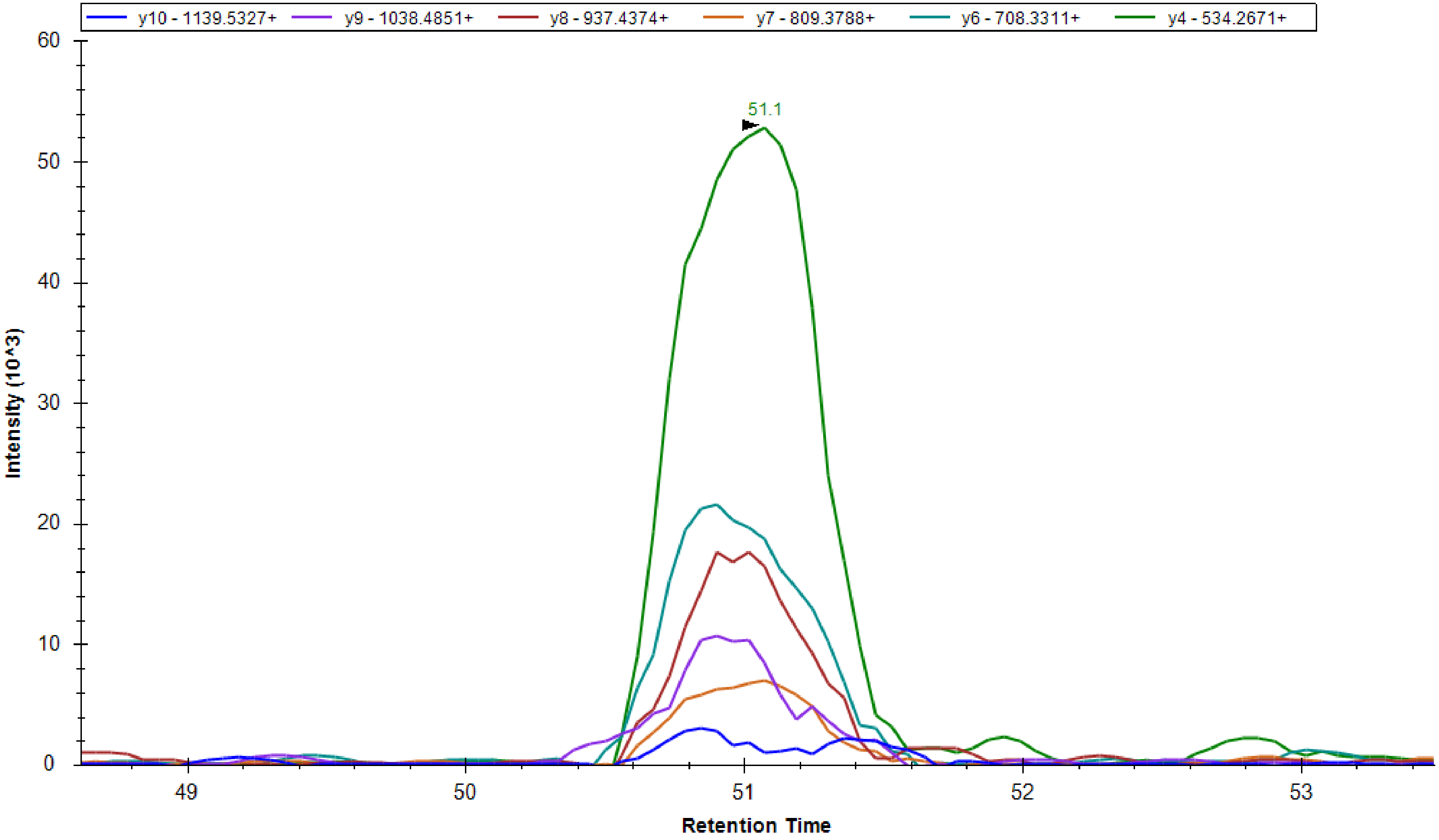

File name: 391-new#B-Round02_Negative-screen.skyd

Parent ion m/z and charges: 787.6992+++

NR_015404.2.1
MEPLTAKSPCFLR

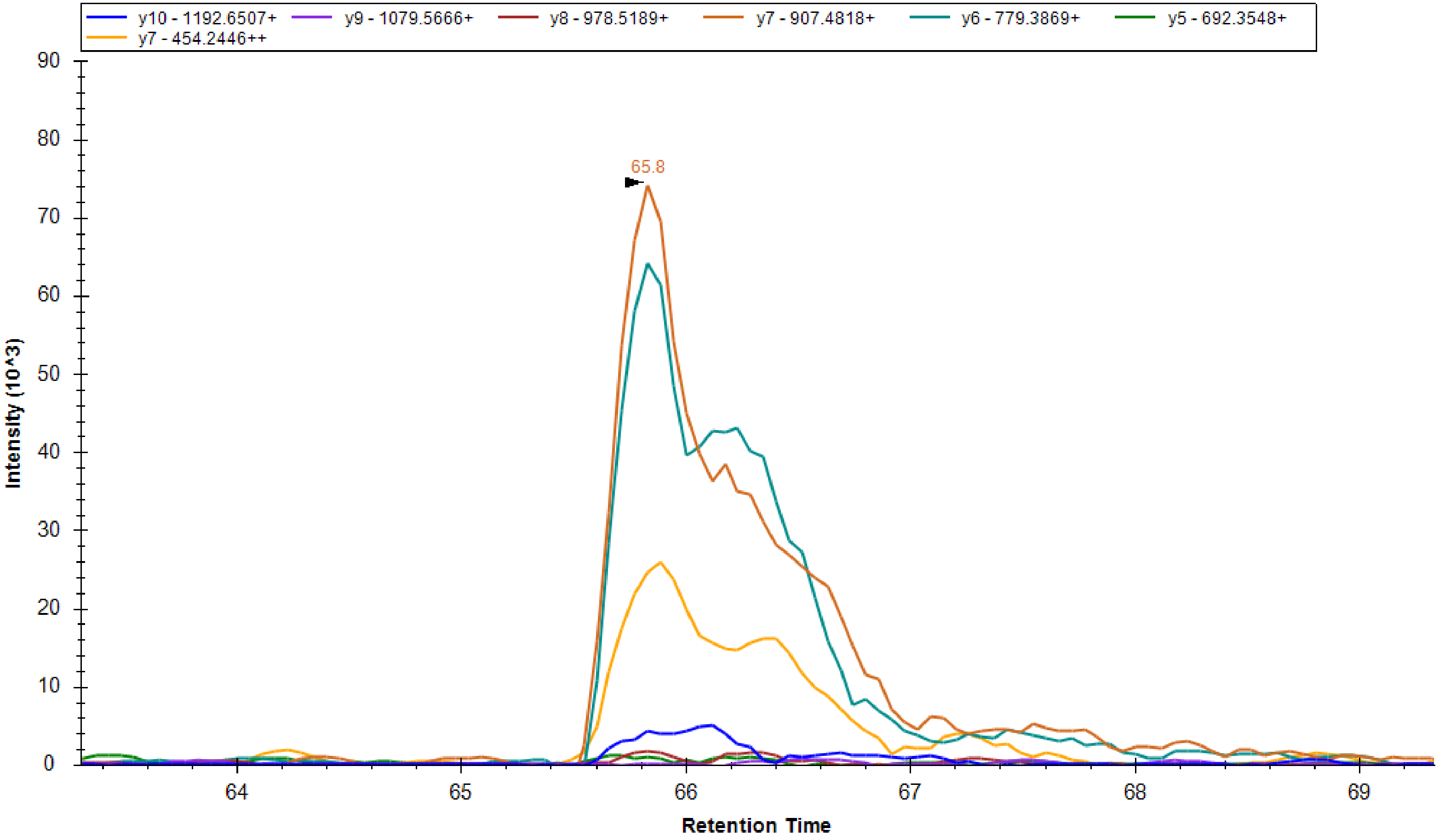

File name: 391-new#B-Round02_Negative-screen.skyd

Parent ion m/z and charges: 775.3969++

NR_015410.1.3
MDGCSPLSVSPDVLK

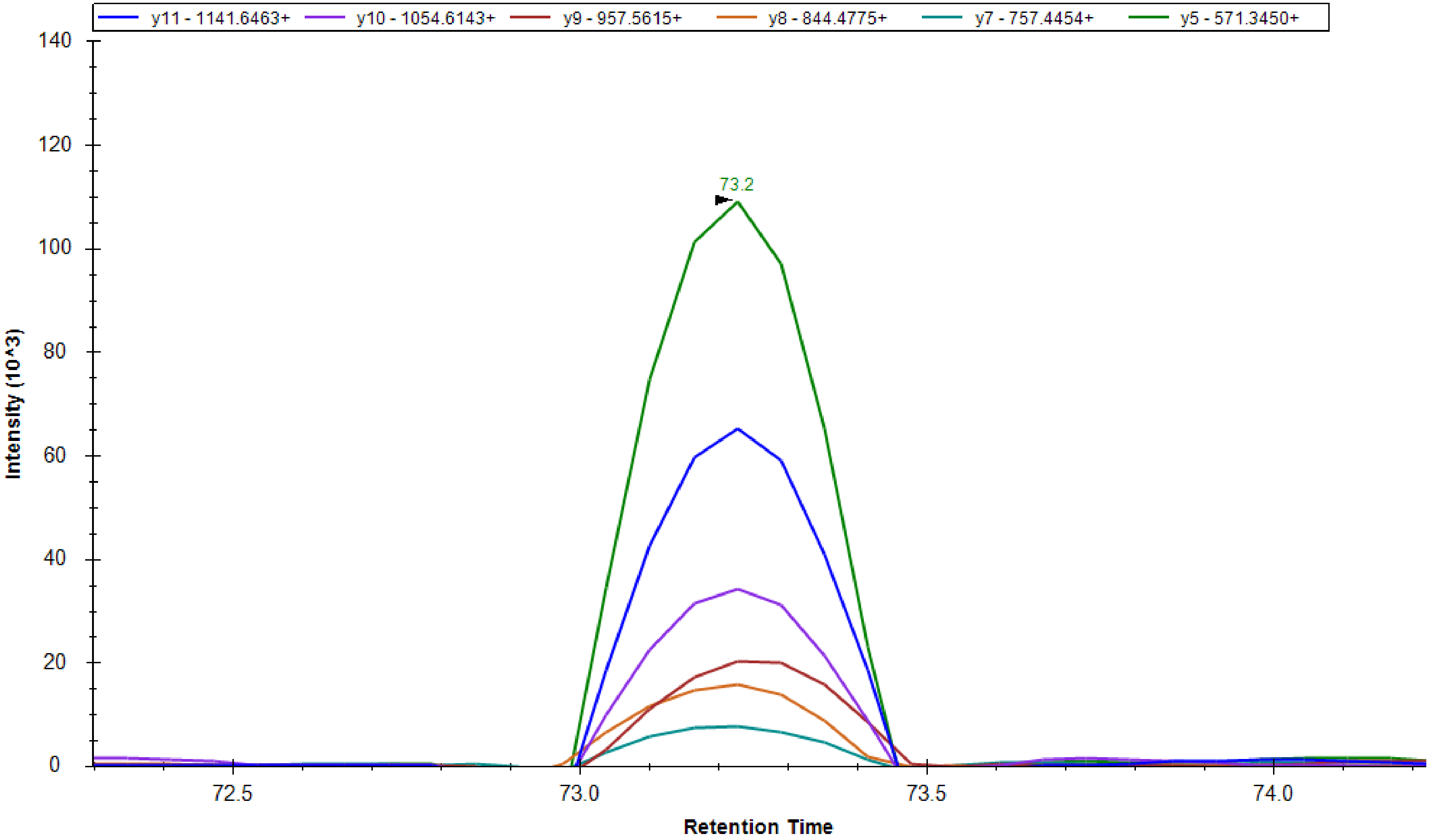

File name: 391-new#A-Round02_Negative-screen.skyd

Parent ion m/z and charges: 802.8866++

NR_015410.1.3
MDGCSPLSVSPDVLK

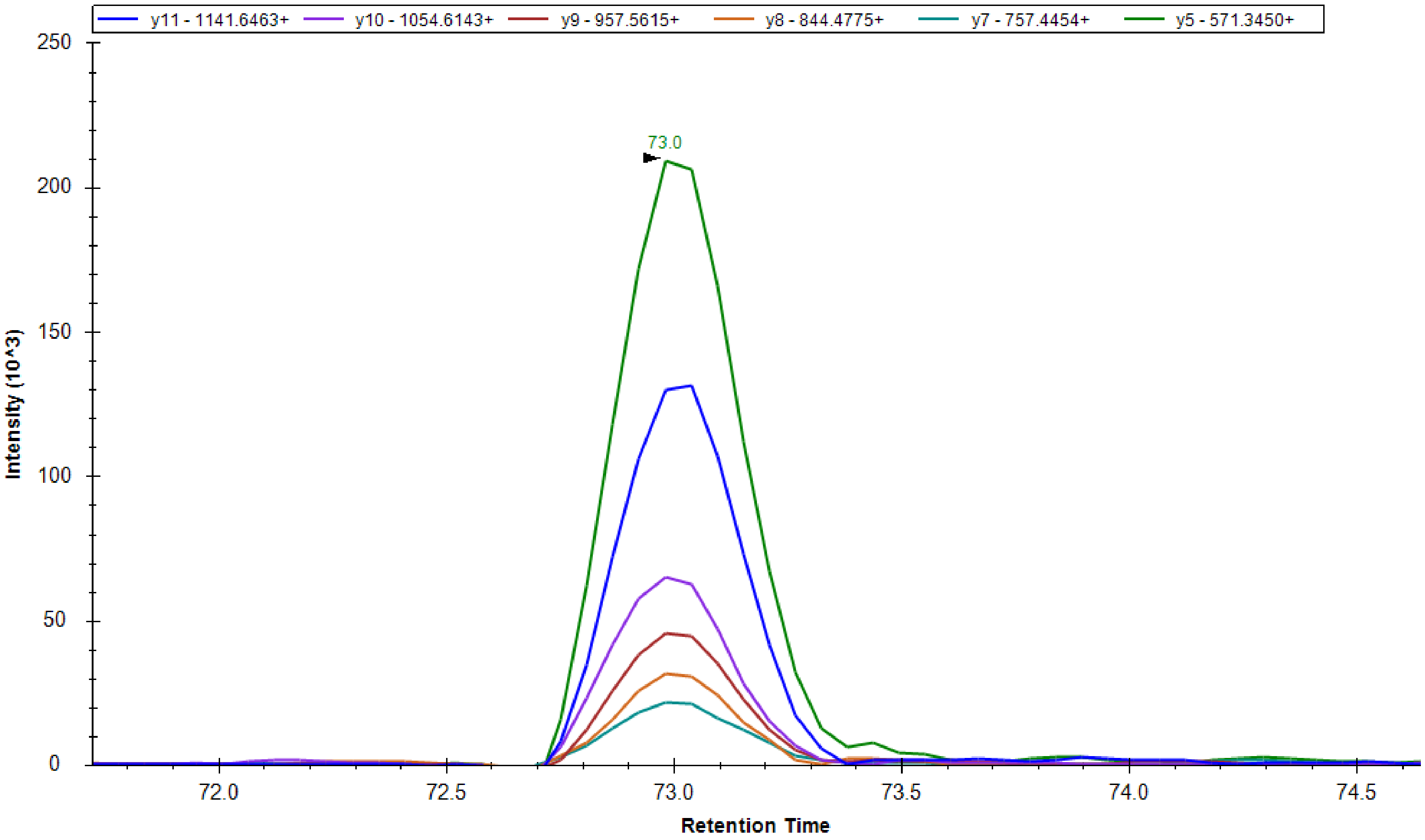

File name: 391-new#B-Round02_Negative-screen.skyd

Parent ion m/z and charges: 802.8866++

NR_015434.1.4
AGTVHAAIEGSQGGWGSPTVCR

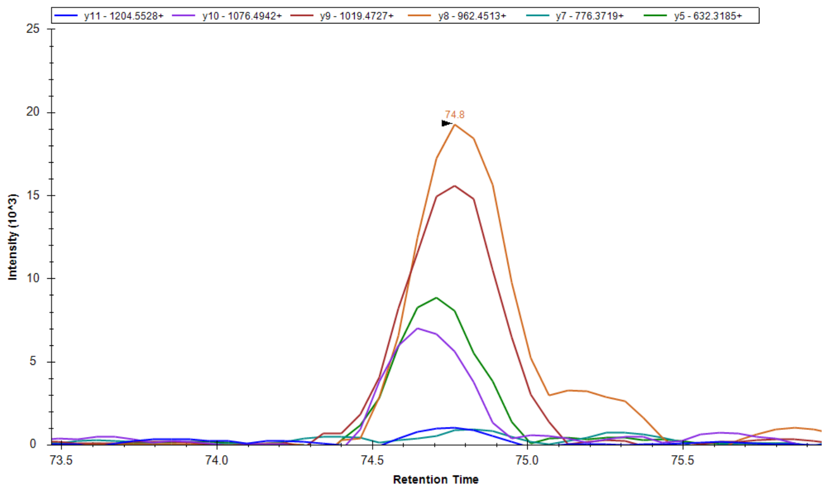

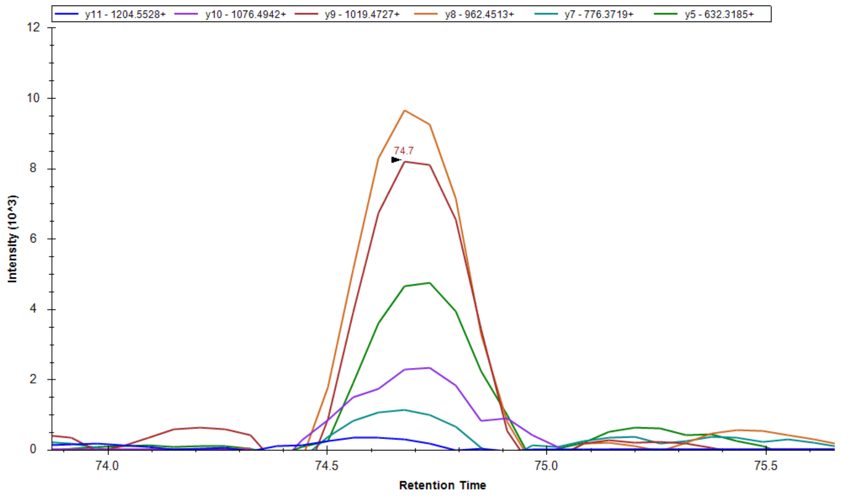

File name: 391-new#B-Round01_All-screening_Positive result.skyd

File name: 391-new#B-Round01_Positive-confirm.skyd

Parent ion m/z and charges: 1099.5240++

NR_015440.3.4
TPPSTPNPAECGGDLGRR

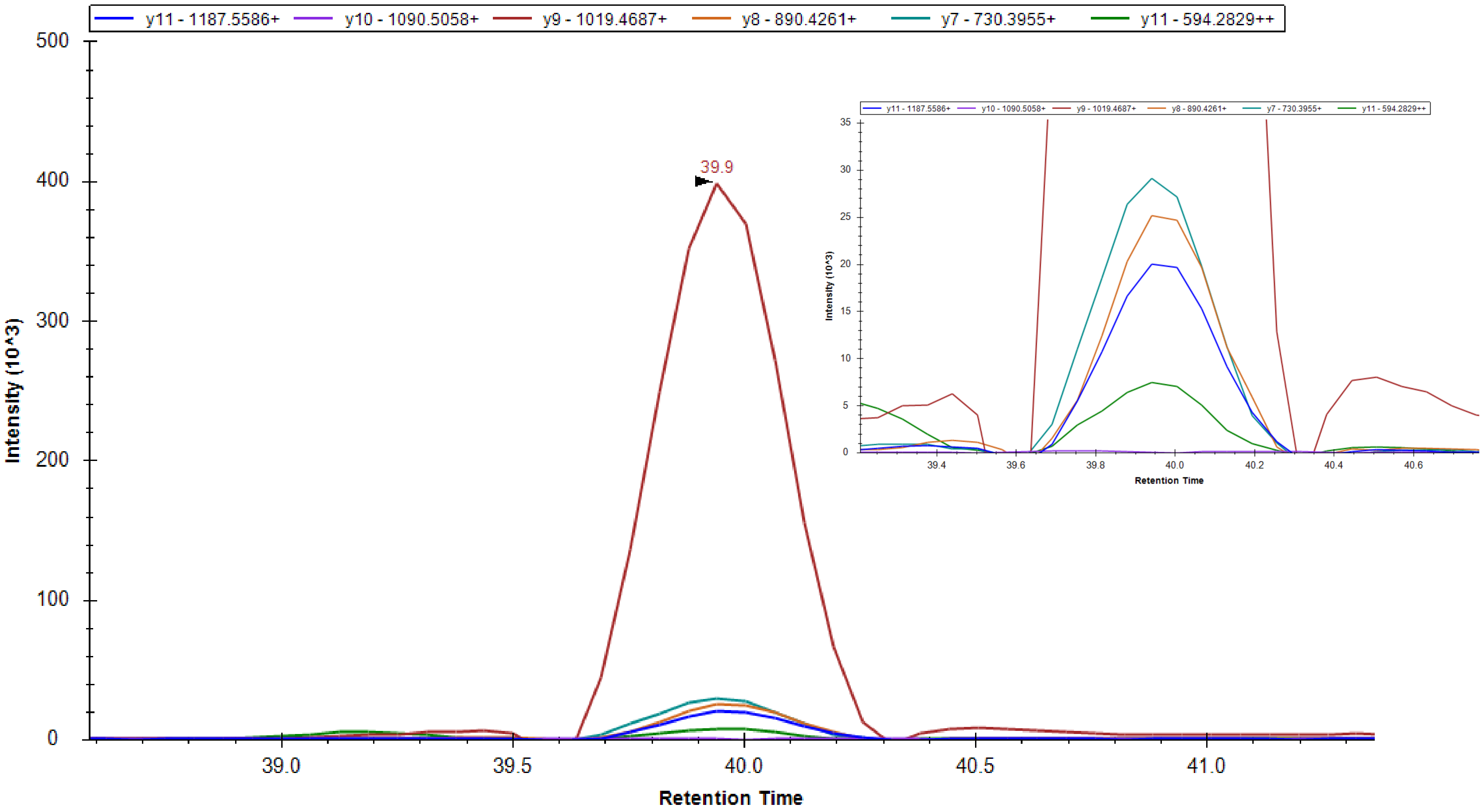

File name: 391-new#A-Round02_Negative-screen.skyd

Parent ion m/z and charges: 941.4472++

NR_015440.3.4
TPPSTPNPAECGGDLGRR

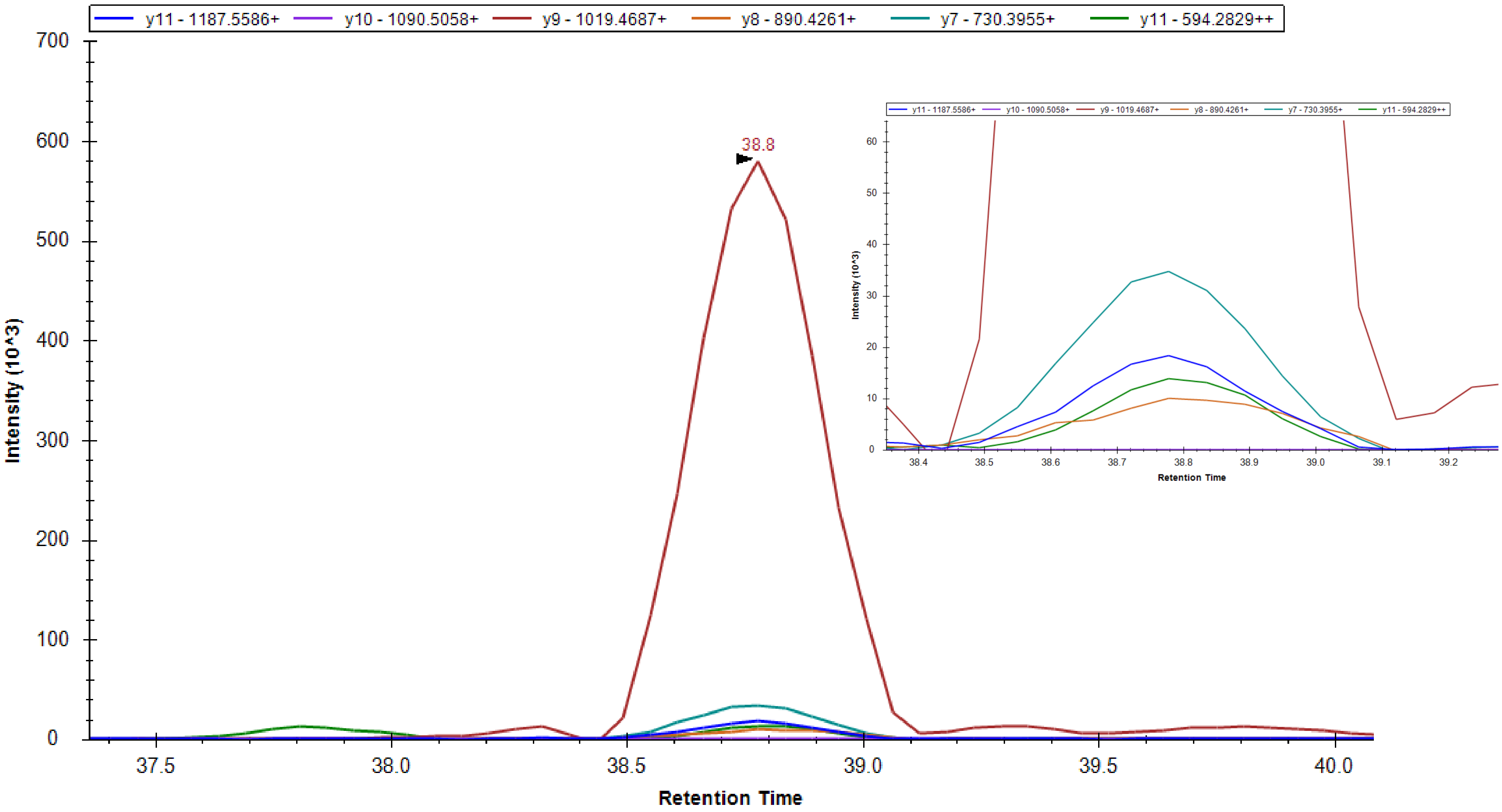

File name: 391-new#B-Round02_Negative-screen.skyd

Parent ion m/z and charges: 941.4472++

NR_024011.1.9 VCTRLLPEEMPVGLK

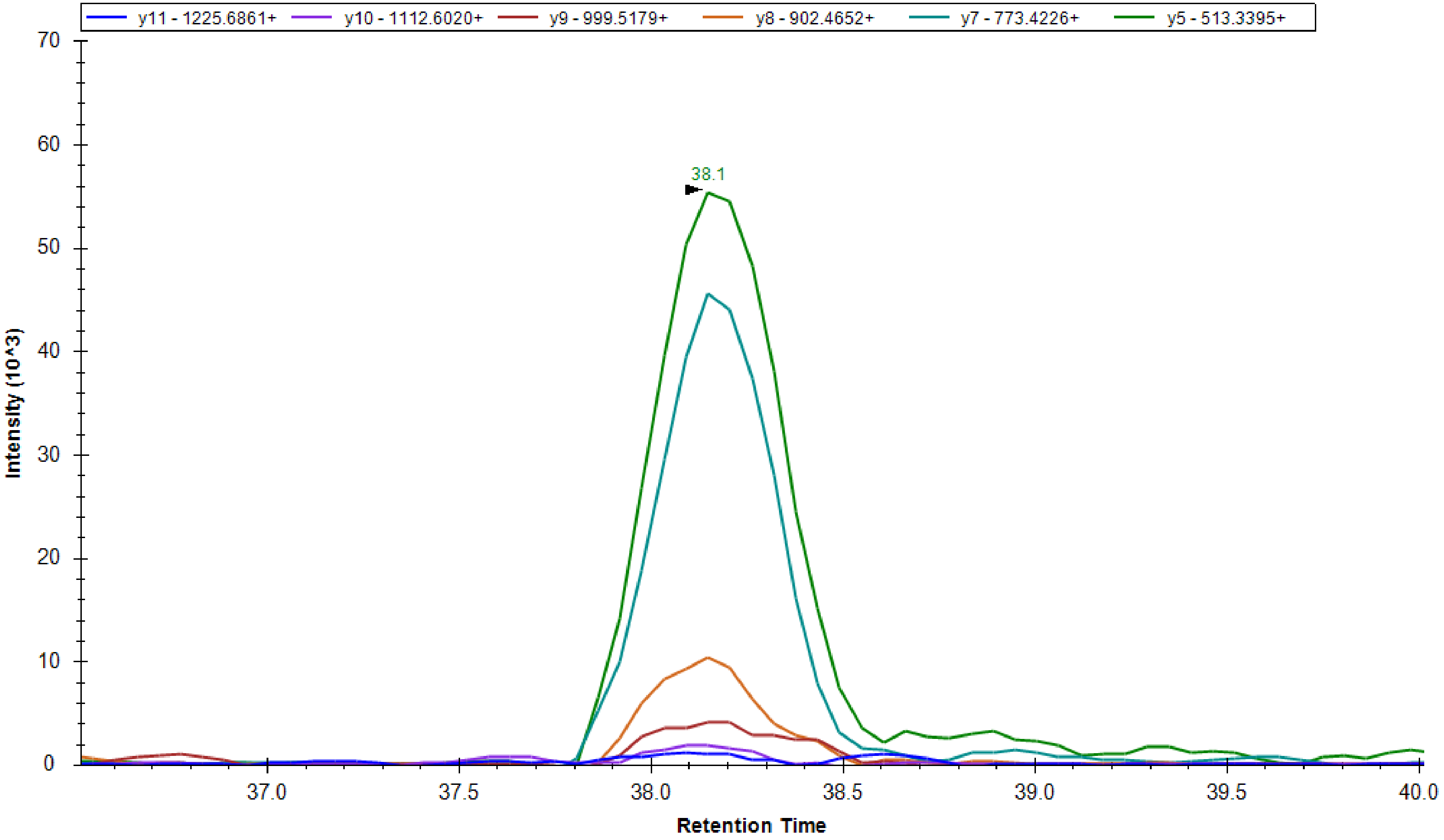

File name: 391-new#B-Round02_Negative-screen.skyd

Parent ion m/z and charges: 871.4706++

NR_024116.1.1
NFGSSHVMQVIYTDDAGRPQTAYLQCK

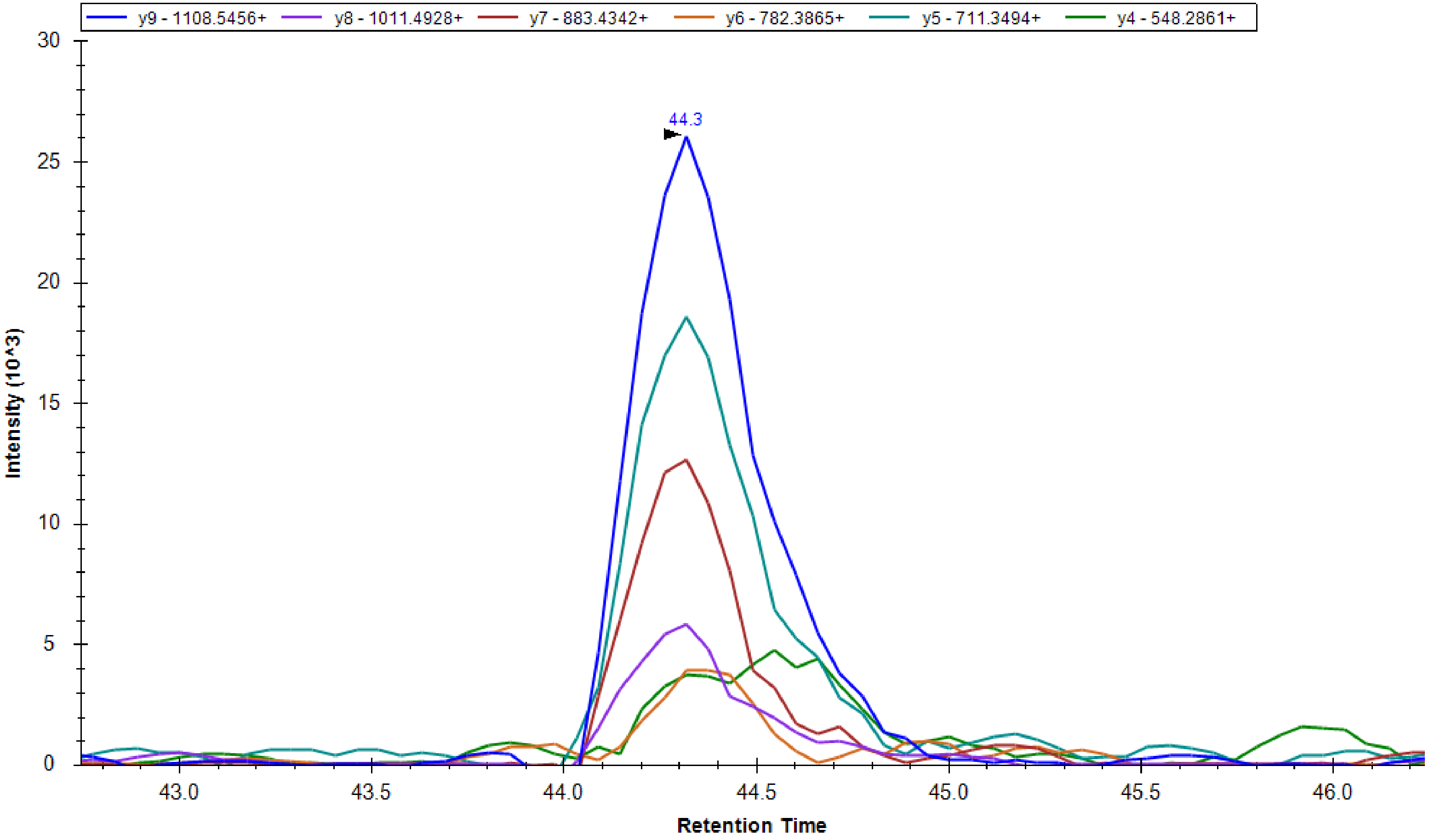

File name: 391-new#B-Round02_Negative-screen.skyd

Parent ion m/z and charges: 772.3669++++

NR_024130.3.2
ILSGSVPGPAK

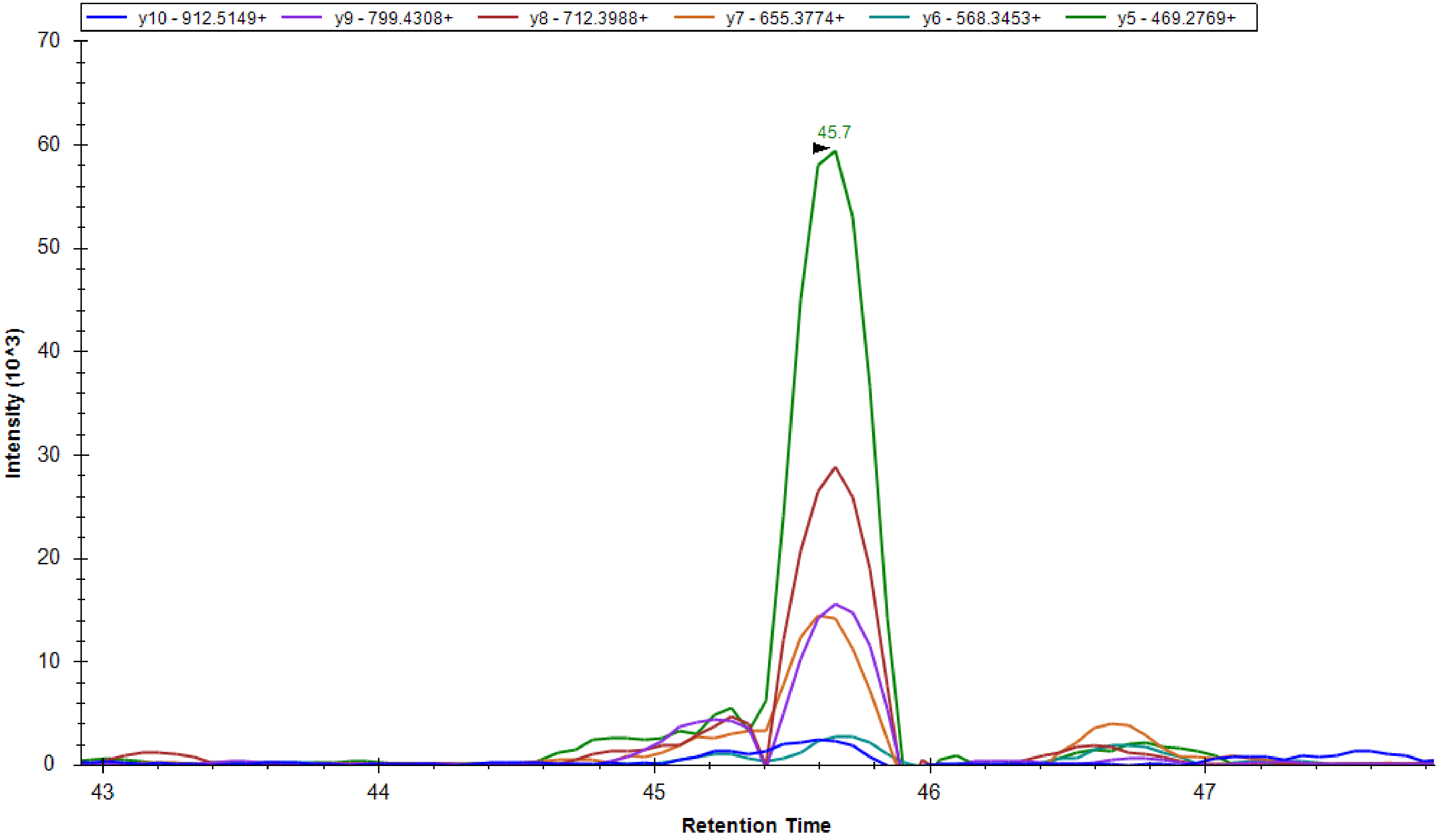

File name: 391-new#A-Round02_Negative-screen.skyd

Parent ion m/z and charges: 513.3031++

NR_024159.3.6
MSPSLPGPEPQELSGLSPRPGSGPAKR

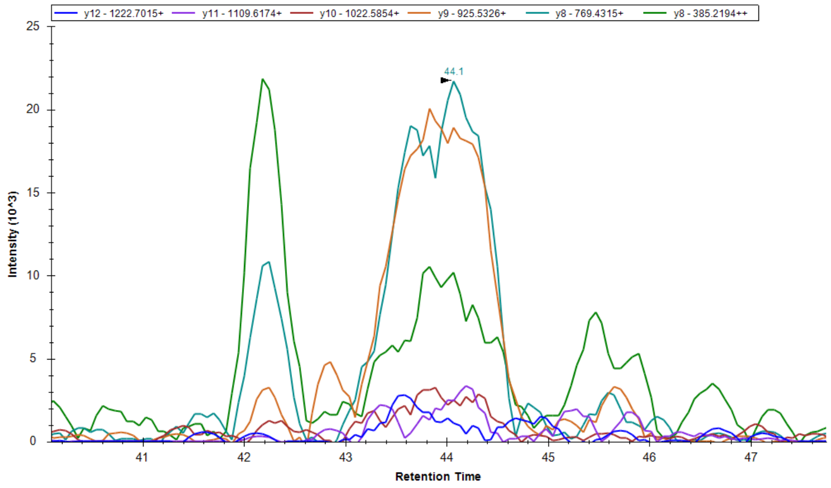

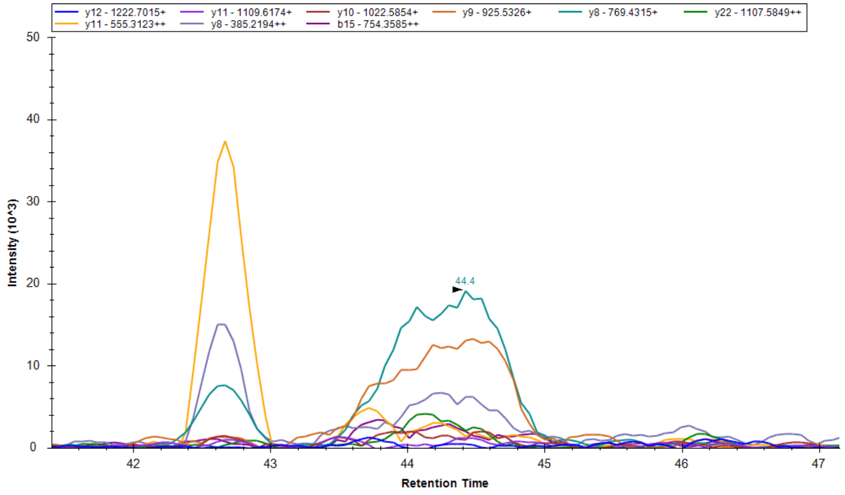

File name: 391-new#B-Round01_All-screening_Positive result.skyd

File name: 391-new#B-Round01_Positive-confirm.skyd

Parent ion m/z and charges: 910.4728+++

NR_024247.1.4
MDLPFQRTTR

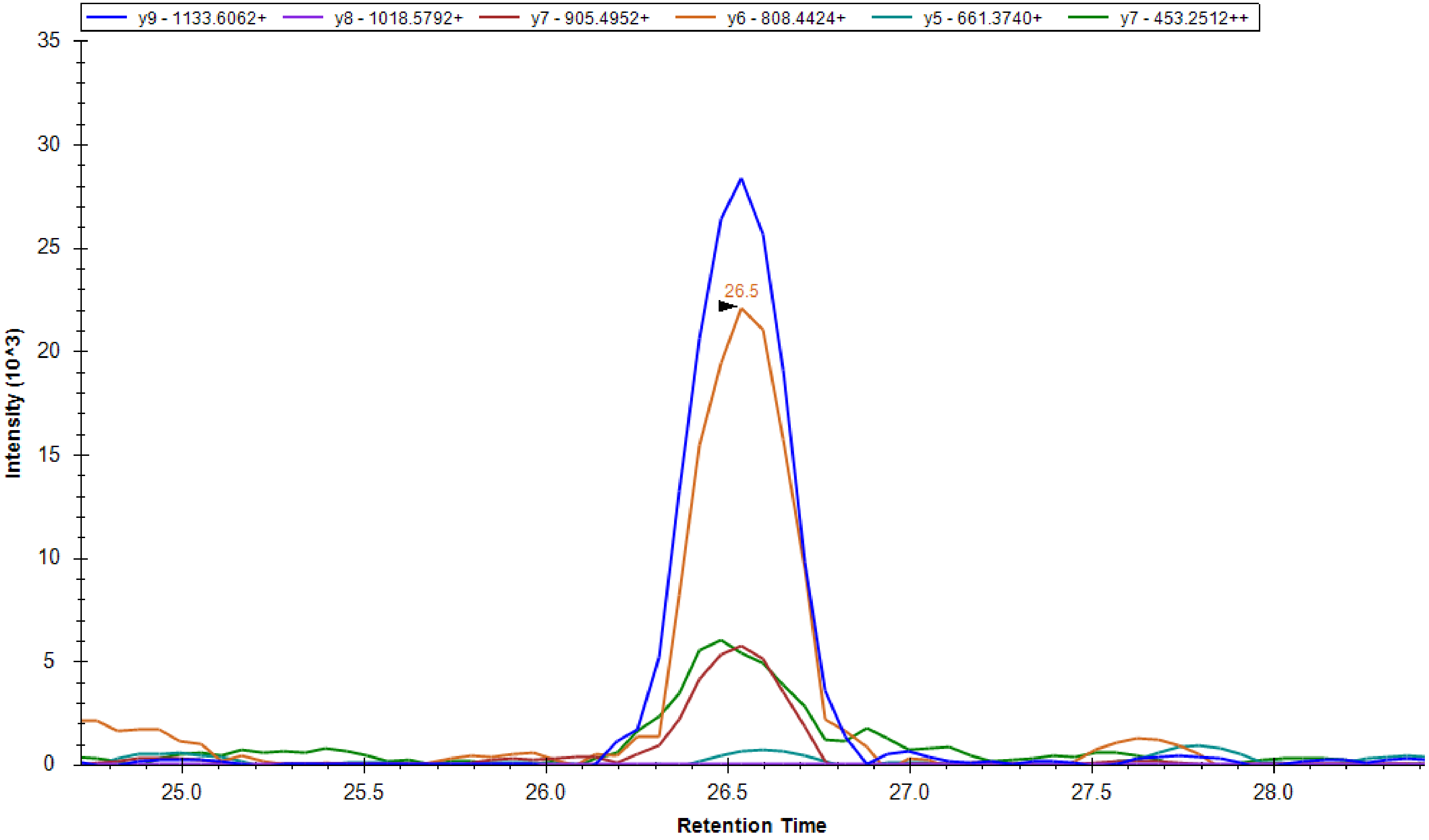

File name: 391-new#B-Round02_Negative-screen.skyd

Parent ion m/z and charges: 632.8270++

NR_024252.2.2
ISIDWNVWRMINSK

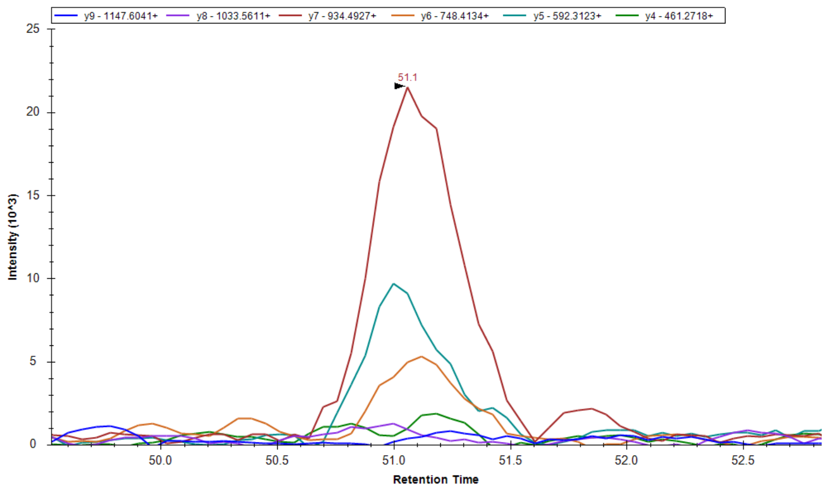

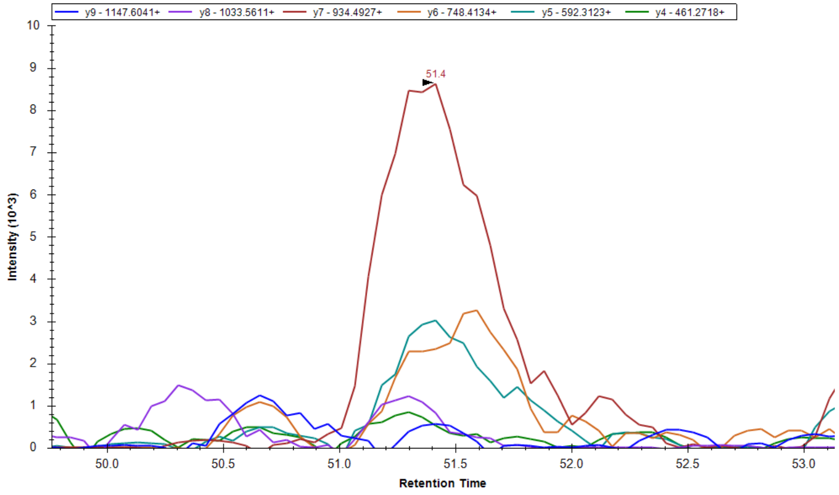

File name: 391-new#B-Round01_All-screening_Positive result.skyd

File name: 391-new#B-Round01_Positive-confirm.skyd

Parent ion m/z and charges: 881.4589++

NR_024252.2.2
ISIDWNVWRMINSK

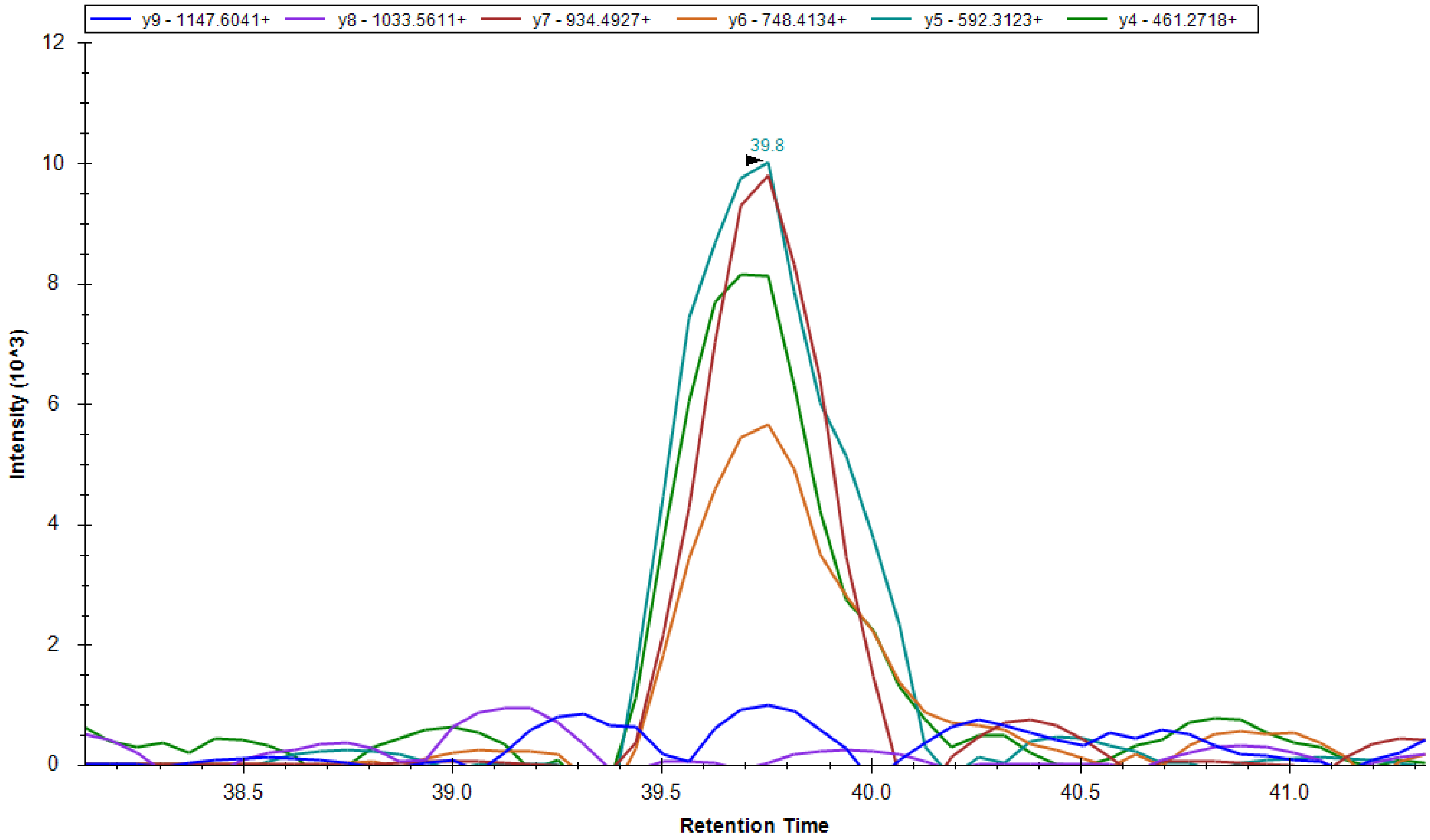

File name: 391-new#A-Round02_Negative-screen.skyd

Parent ion m/z and charges: 881.4589++

NR_024438.1.2
CPRHQGMMVGMGQR

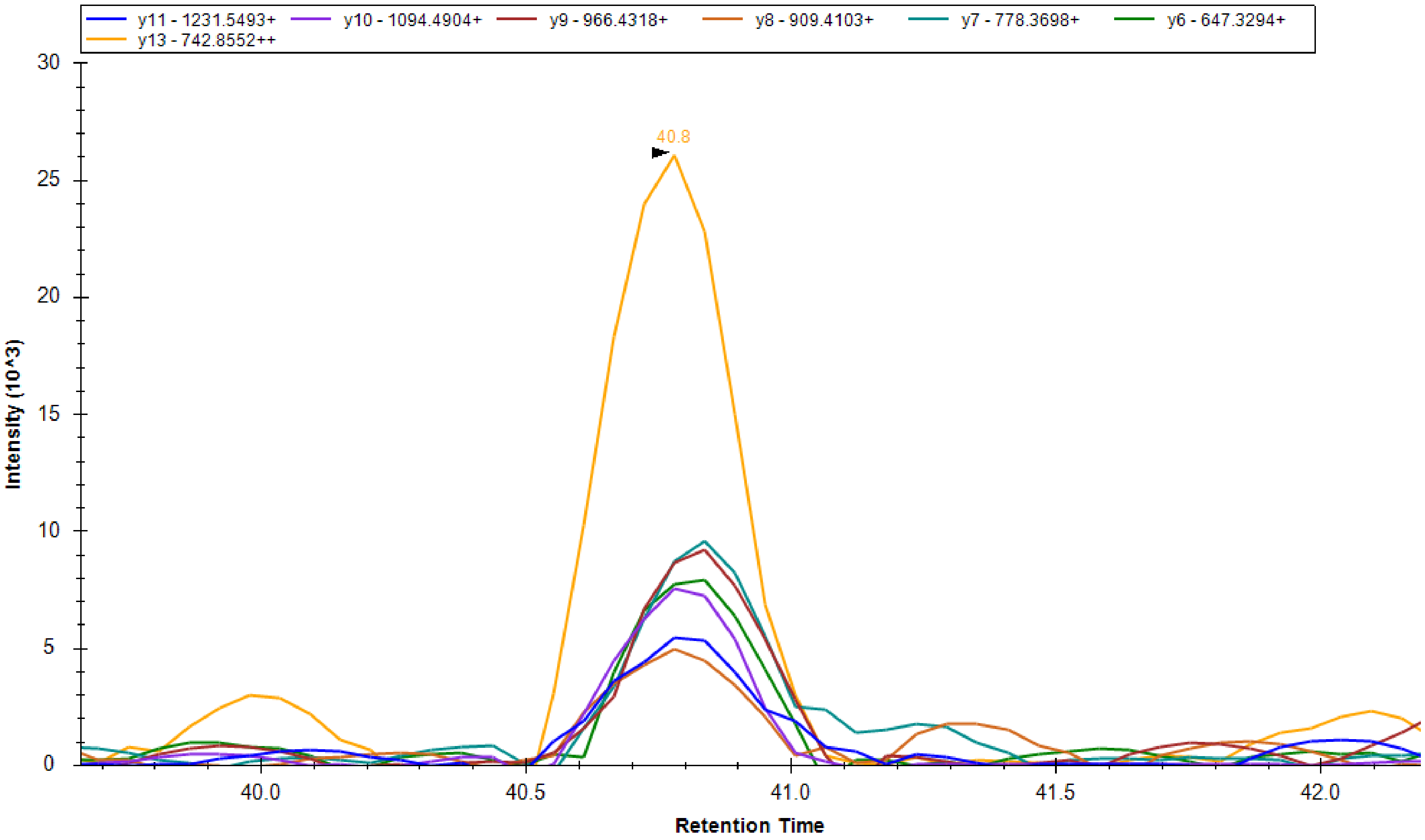

File name: 391-new#B-Round02_Negative-screen.skyd

Parent ion m/z and charges: 822.8705++

NR_024557.1.3
MVIRTGTVNIIPNINIFEIHISR

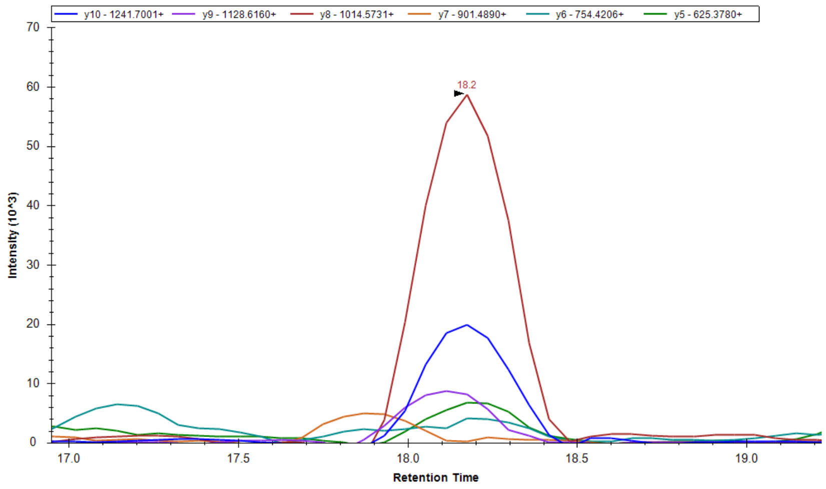

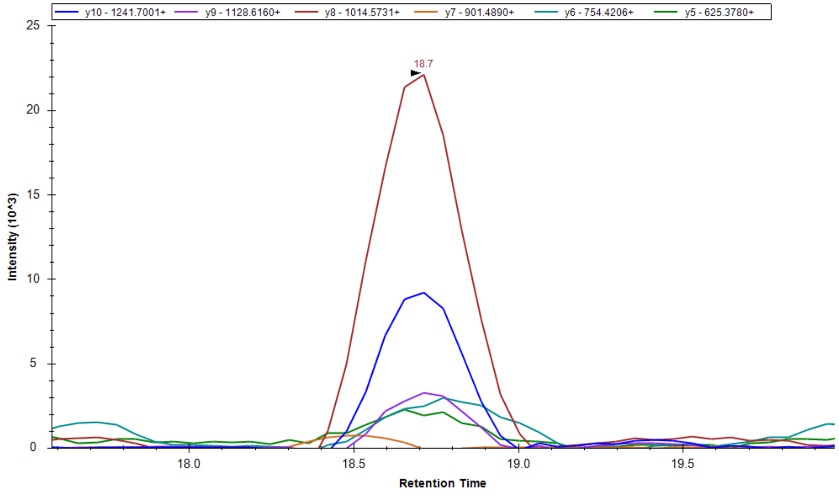

File name: 391-new#B-Round01_All-screening_Positive result.skyd

File name: 391-new#B-Round01_Positive-confirm.skyd

Parent ion m/z and charges: 884.1669+++

NR_024583.1.4
MLCPLLSRAGMASSRK

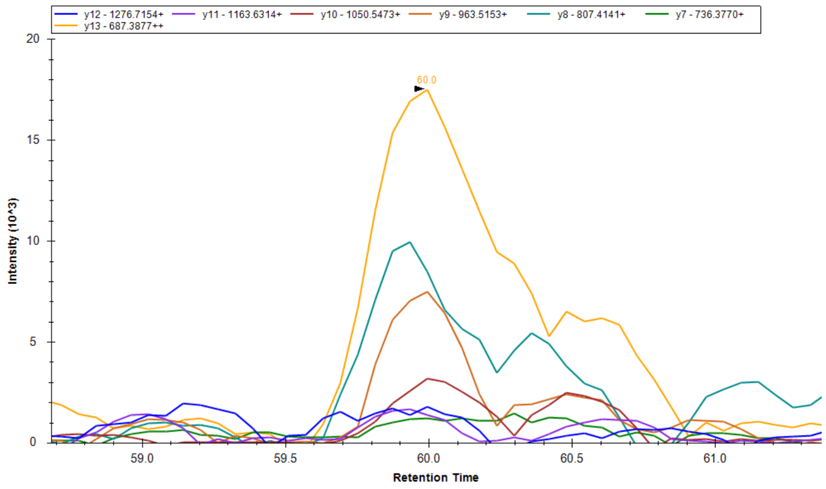

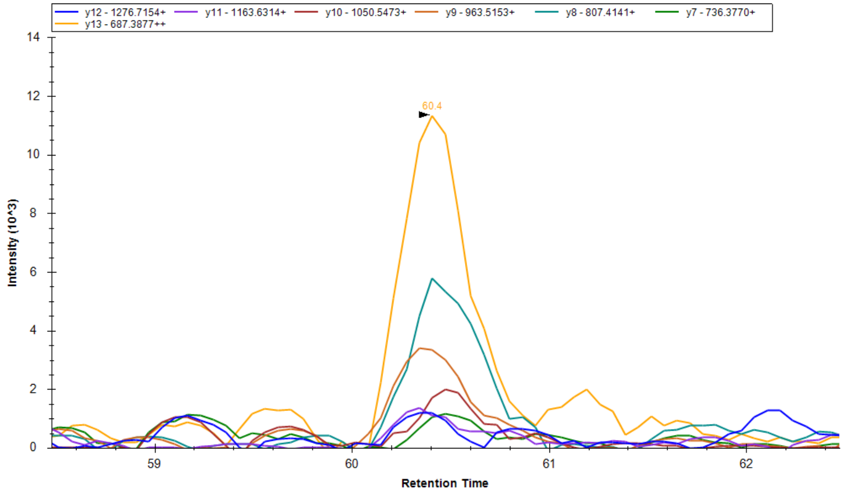

File name: 391-new#A-Round01_All-screening_Positive result.skyd

File name: 391-new#A-Round01_Positive-confirm.skyd

Parent ion m/z and charges: 889.4653++

NR_024583.1.4
MLCPLLSRAGMASSRK

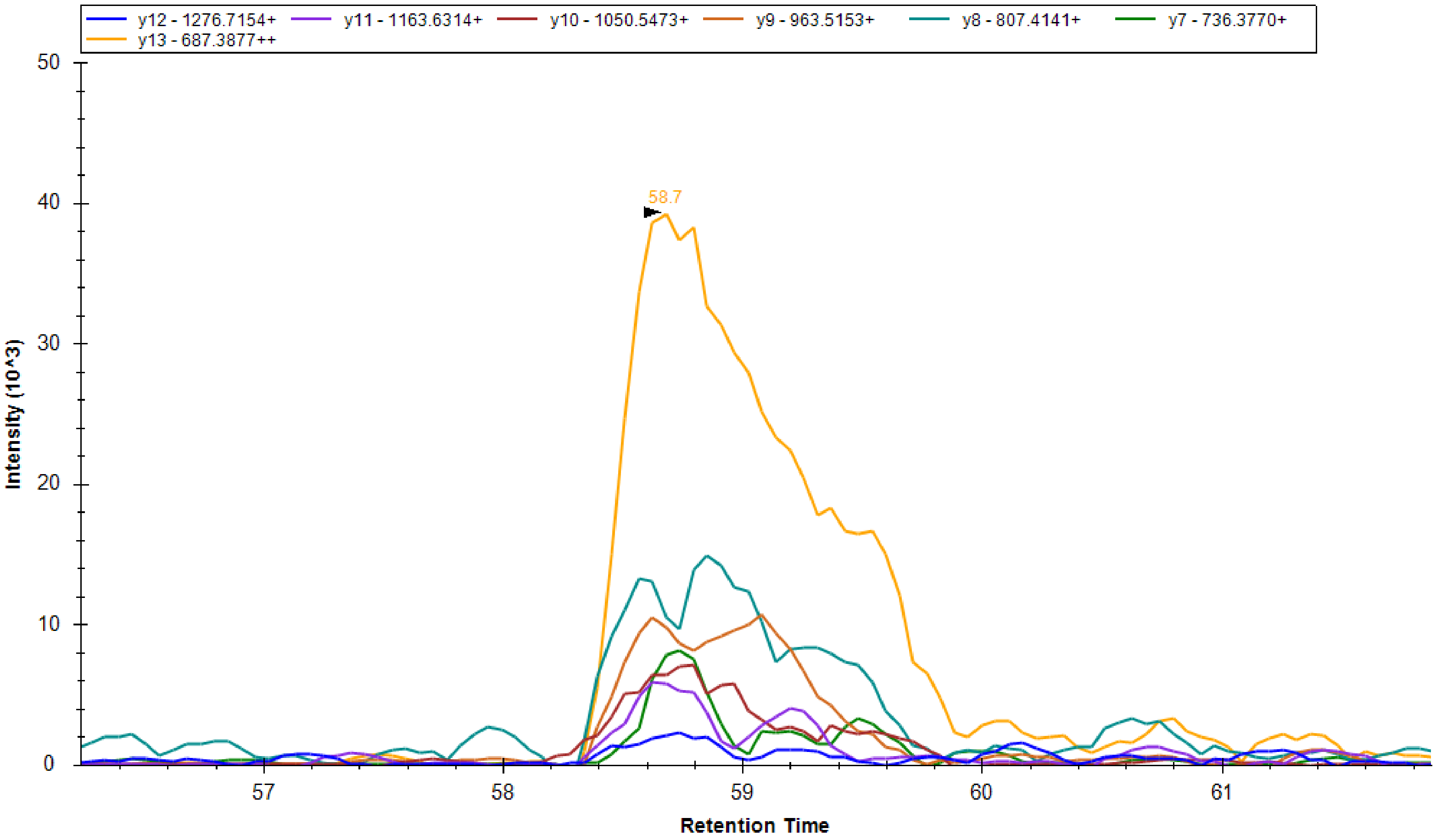

File name: 391-new#B-Round02_Negative-screen.skyd

Parent ion m/z and charges: 889.4653++

NR_026550.2.1
APPSLLPGAGGGPGAGSRQPPPPQPSR

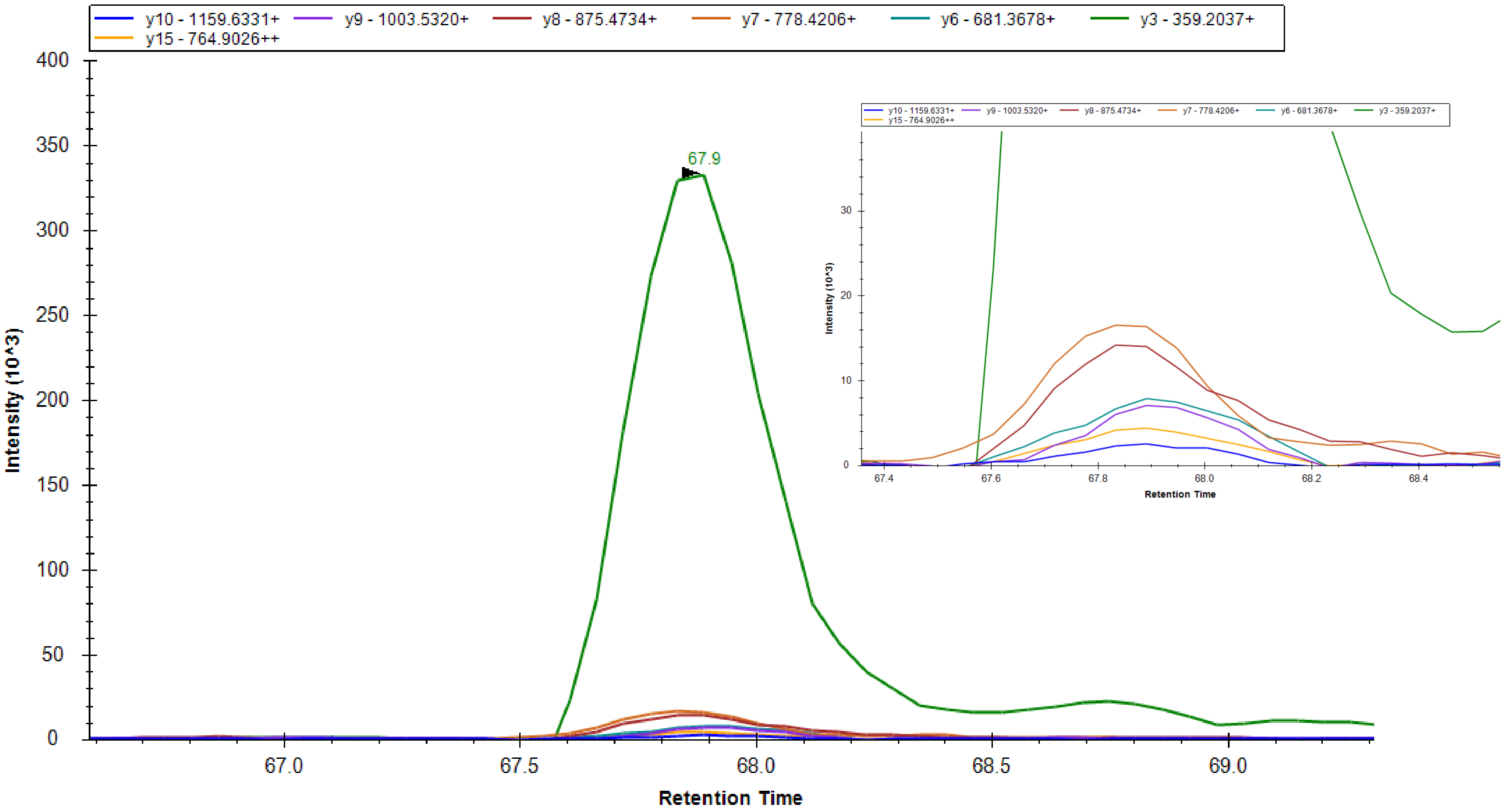

File name: 391-new#B-Round02_Negative-screen.skyd

Parent ion m/z and charges: 835.1103+++

NR_026718.2.2
MREIARAIGGSSTIMGGR

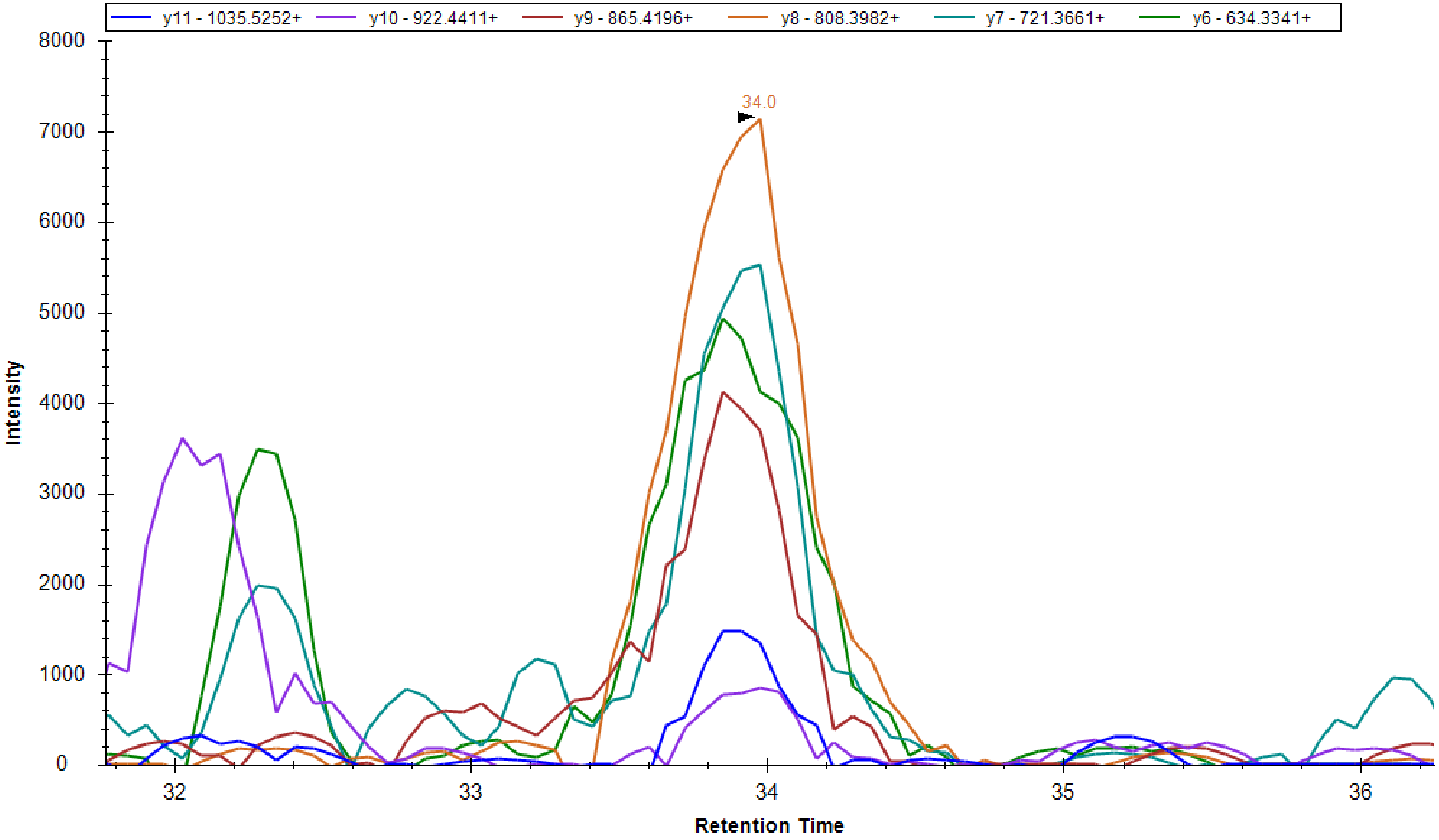

File name: 391-new#A-Round02_Negative-screen.skyd

Parent ion m/z and charges: 621.6611+++

NR_026730.2.13
EFTIALYYNQTVESQR

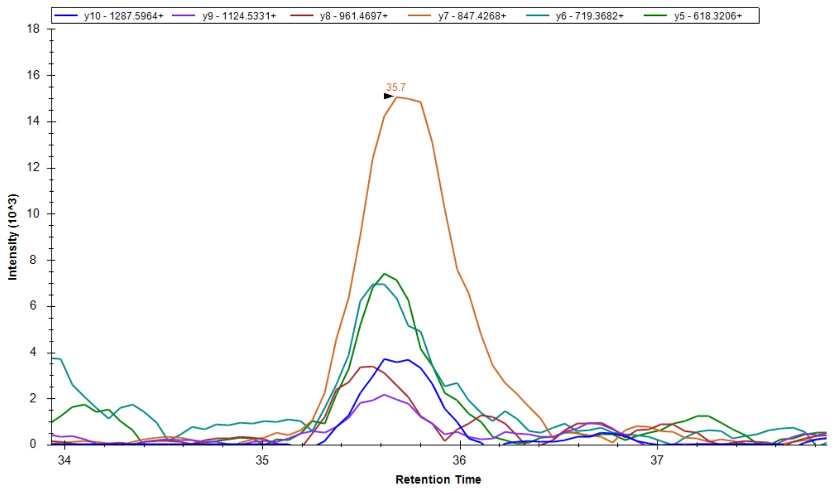

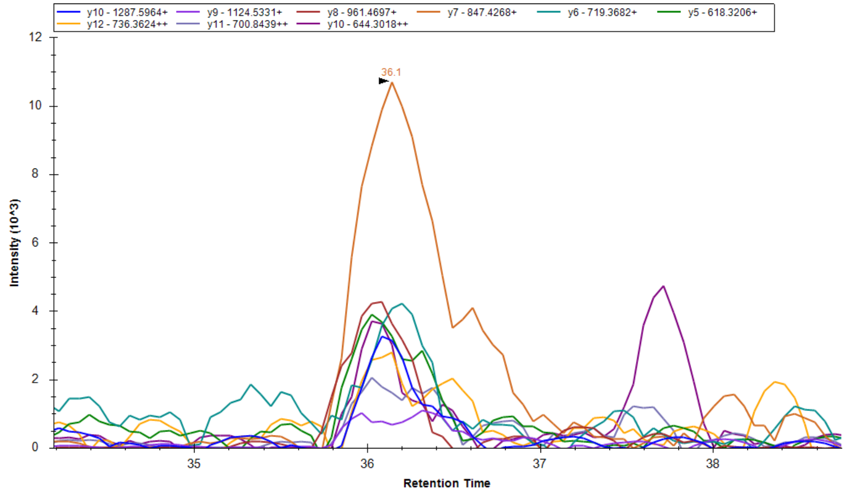

File name: 391-new#B-Round01_All-screening_Positive result.skyd

File name: 391-new#B-Round01_Positive-confirm.skyd

Parent ion m/z and charges: 981.4838++

NR_026730.2.13
EFTIALYYNQTVESQR

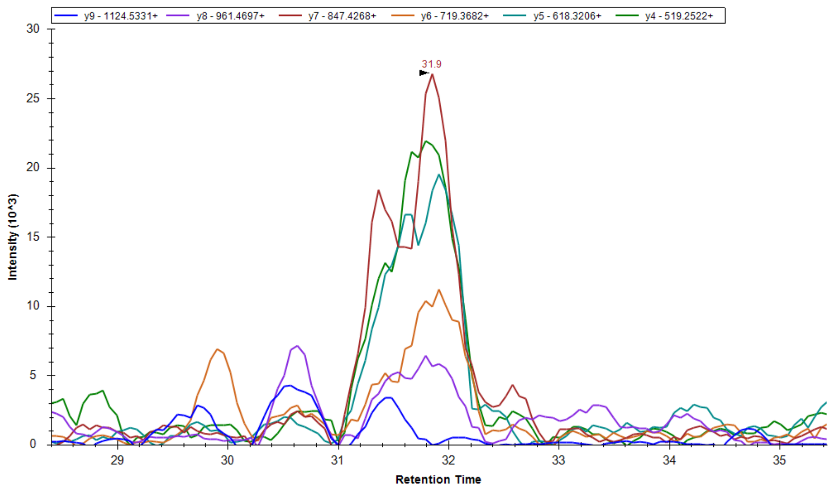

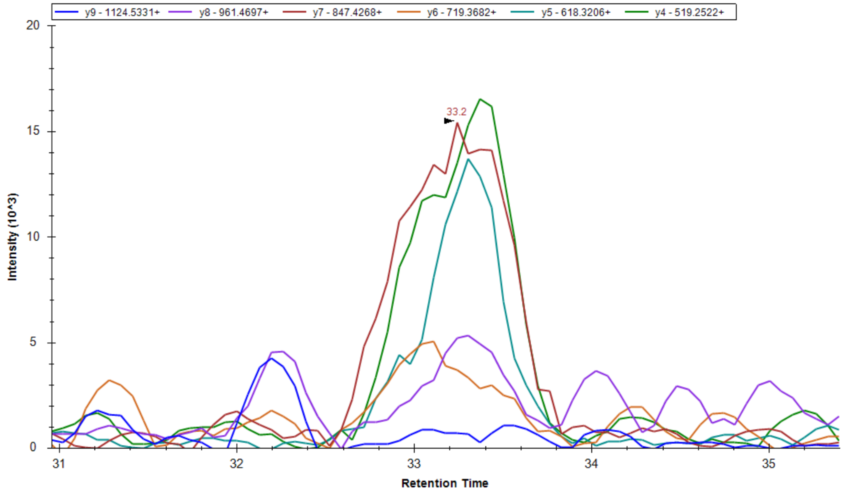

File name: 391-new#A-Round01_All-screening_Positive result.skyd

File name: 391-new#A-Round01_Positive-confirm.skyd

Parent ion m/z and charges: 654.6583+++

NR_026804.3.4
MMLSVPSFSSQDIGHVNESDVASITGTGK

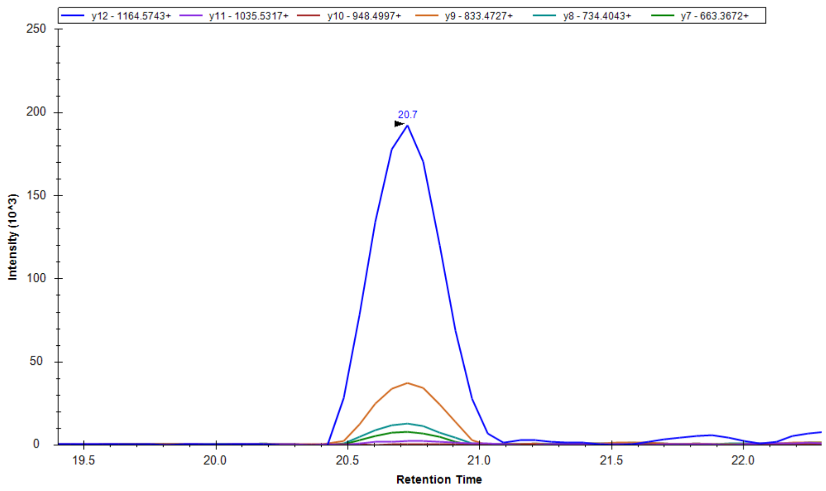

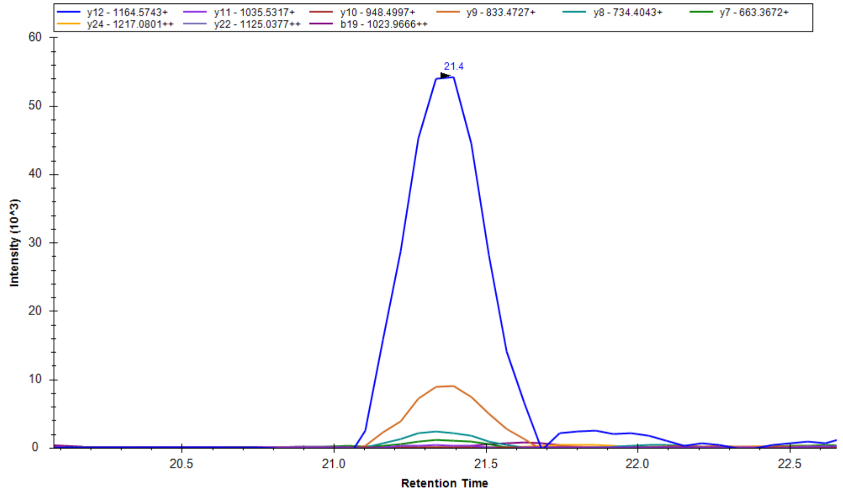

File name: 391-new#B-Round01_All-screening_Positive result.skyd

File name: 391-new#B-Round01_Positive-confirm.skyd

Parent ion m/z and charges: 998.8110+++

NR_026804.3.4
MMLSVPSFSSQDIGHVNESDVASITGTGK

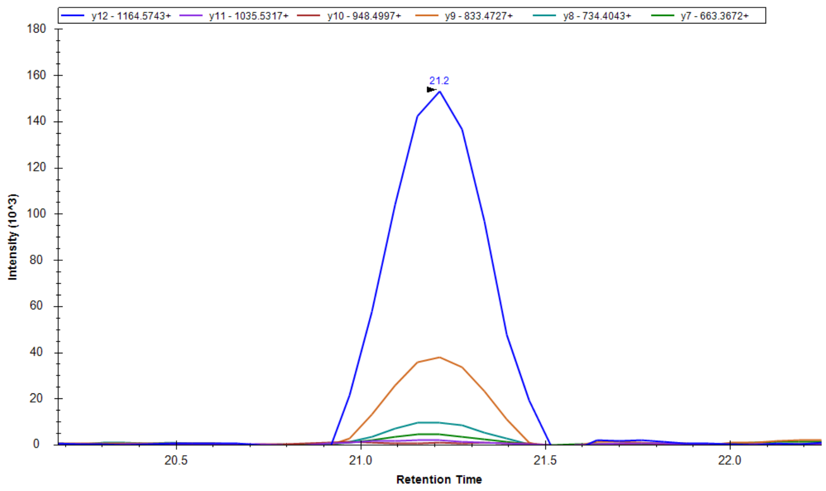

File name: 391-new#A-Round01_All-screening_Positive result.skyd

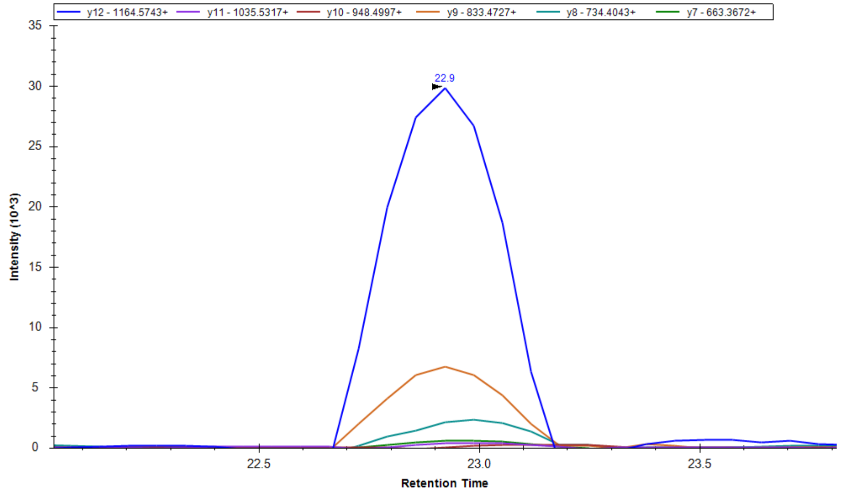

File name: 391-new#A-Round01_Positive-confirm.skyd

Parent ion m/z and charges: 998.8110+++

NR_026827.1.3
ITQMAEENIKK

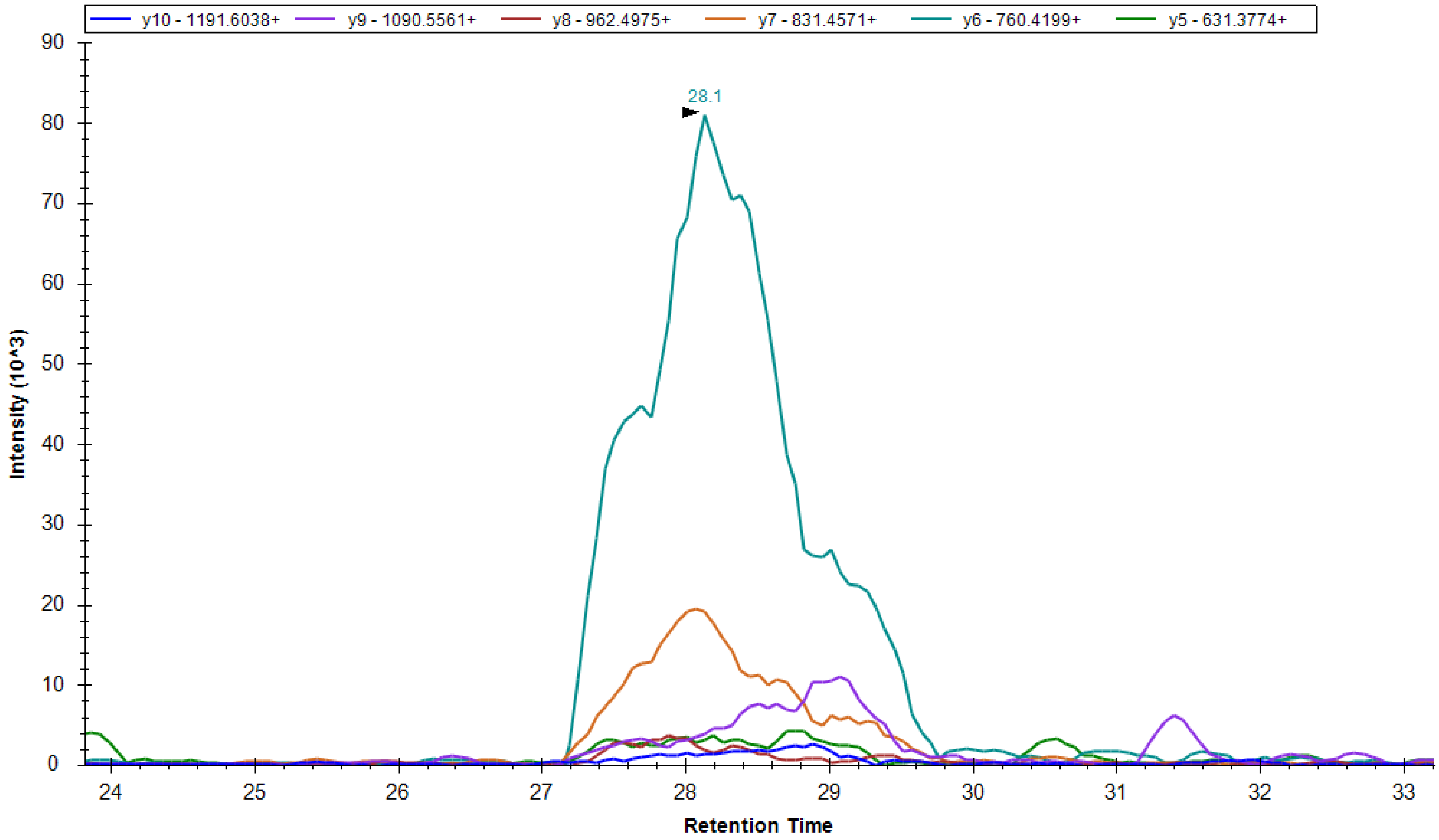

File name: 391-new#A-Round02_Negative-screen.skyd

Parent ion m/z and charges: 652.8476++

NR_026835.2.10
MVKMCDRIIAGIER

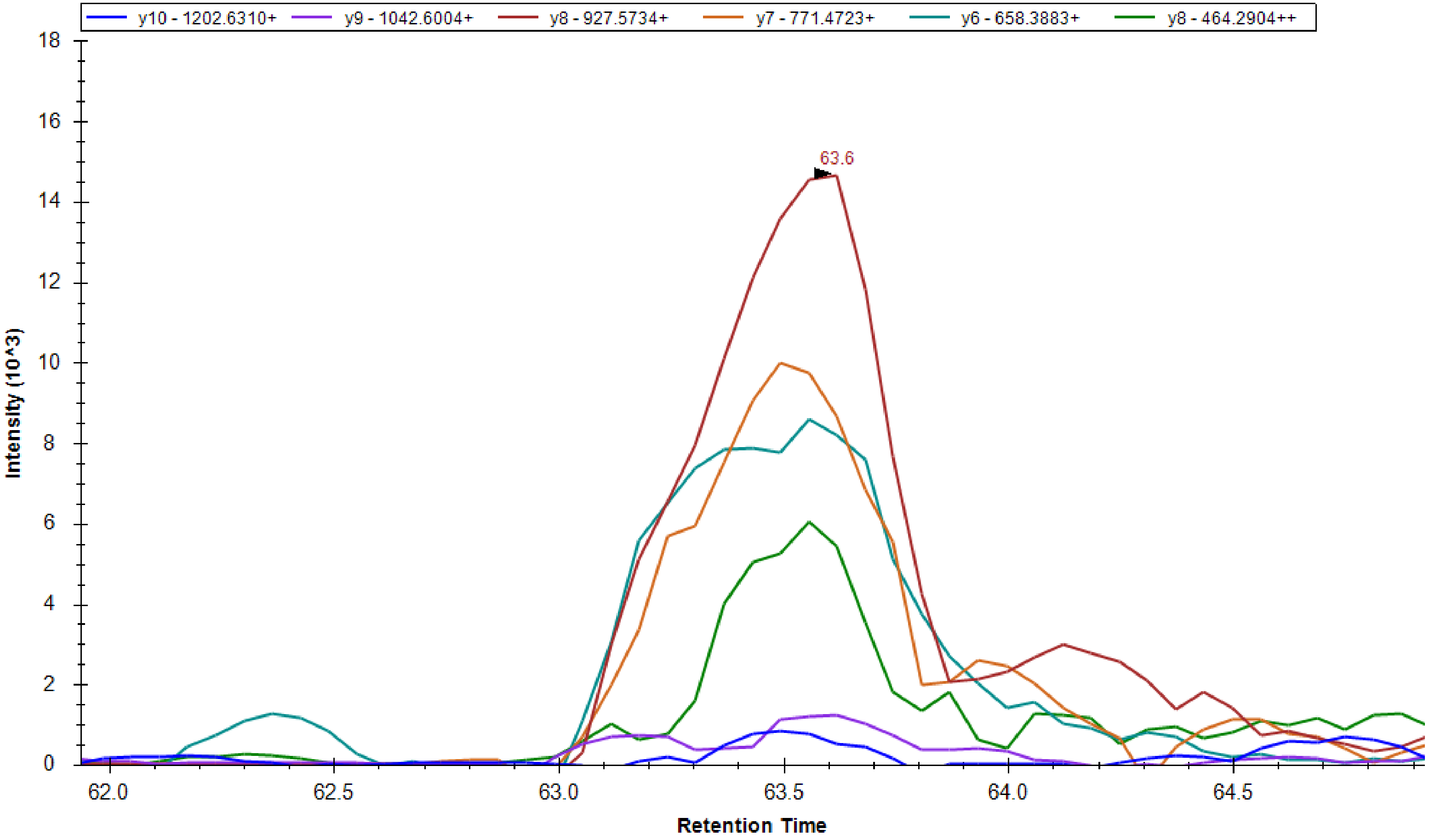

File name: 391-new#A-Round02_Negative-screen.skyd

Parent ion m/z and charges: 846.4413++

NR_026864.1.4
MGLRAGPILLLLLLWLLPGK

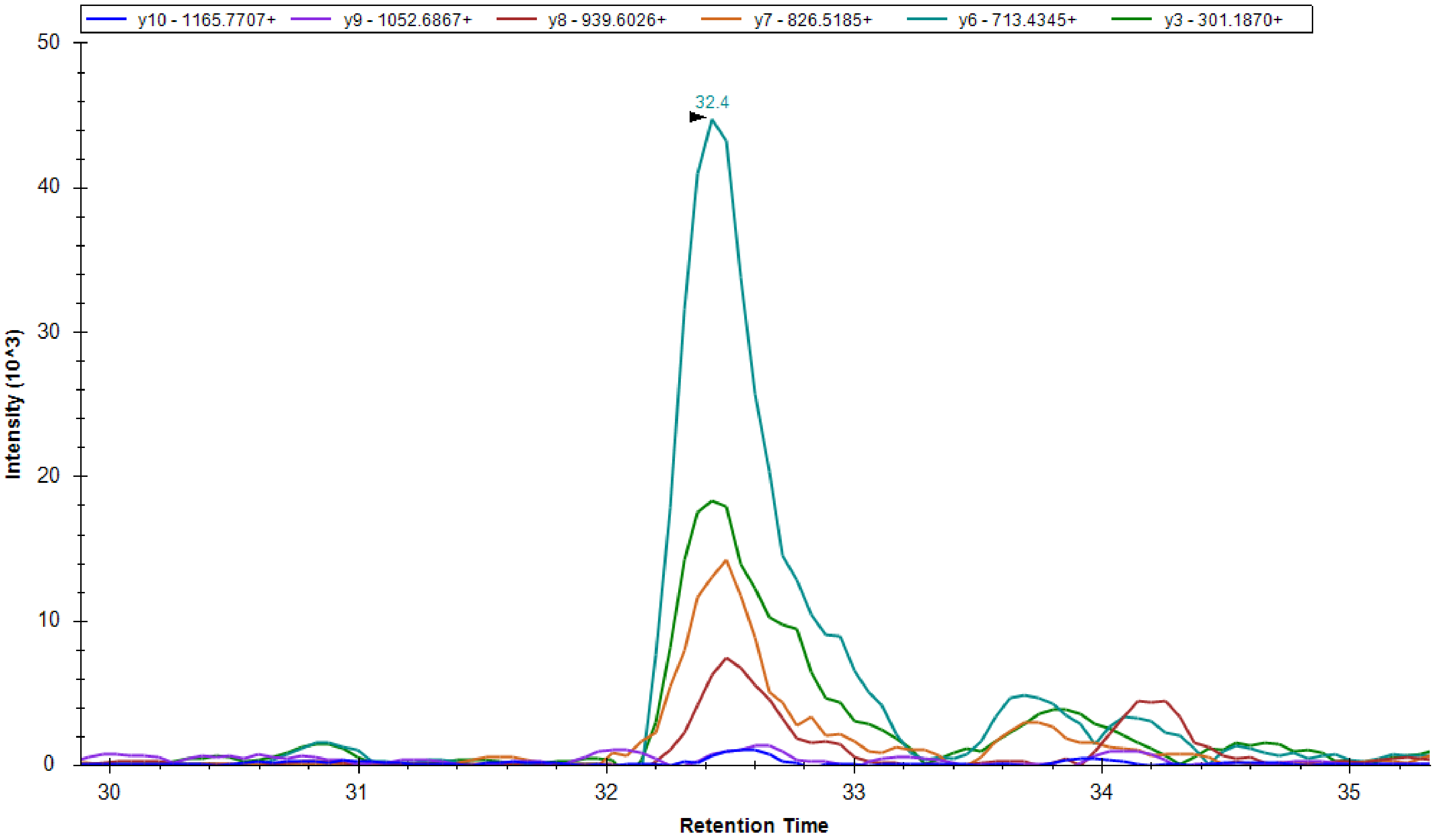

File name: 391-new#B-Round02_Negative-screen.skyd

Parent ion m/z and charges: 692.1040+++

NR_026864.1.4
MGLRAGPILLLLLLWLLPGK

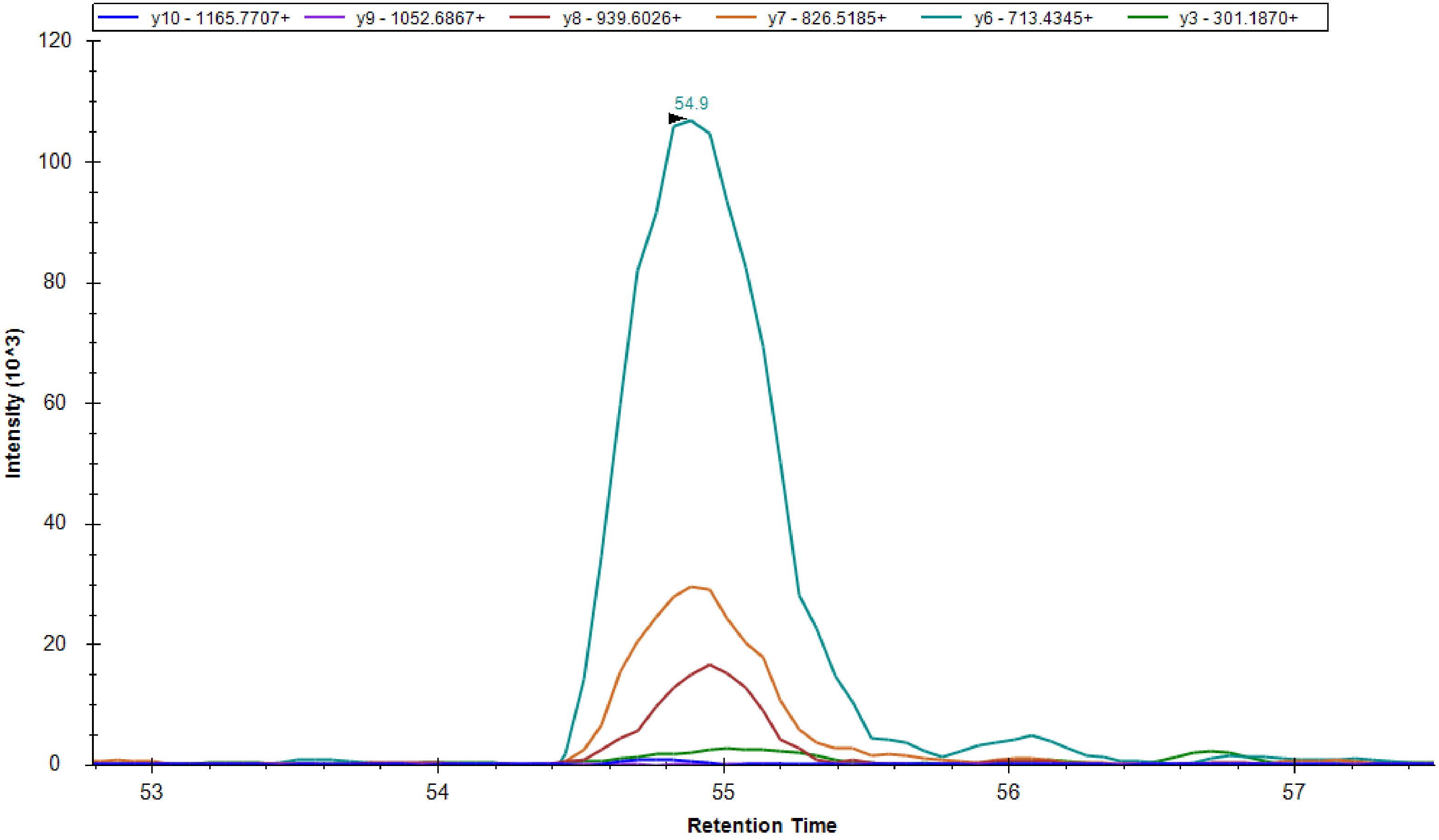

File name: 391-new#A-Round02_Negative-screen.skyd

Parent ion m/z and charges: 692.1040+++

NR_026883.2.1
CITDCIQSRFNQRDGSIK

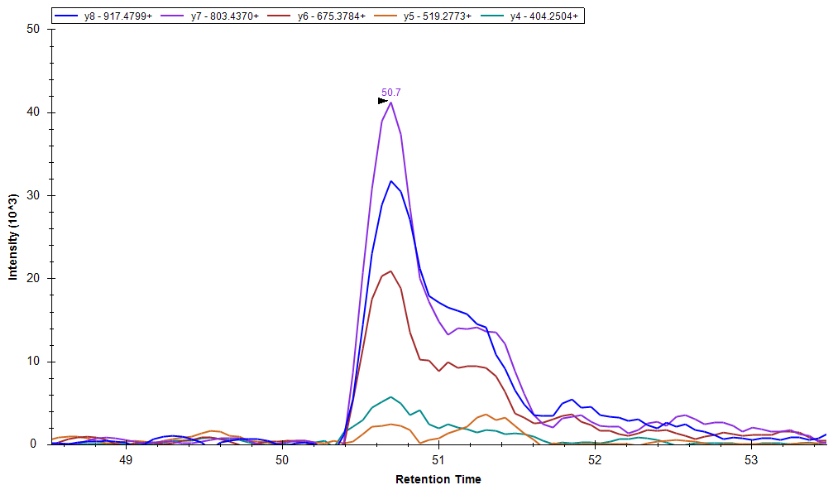

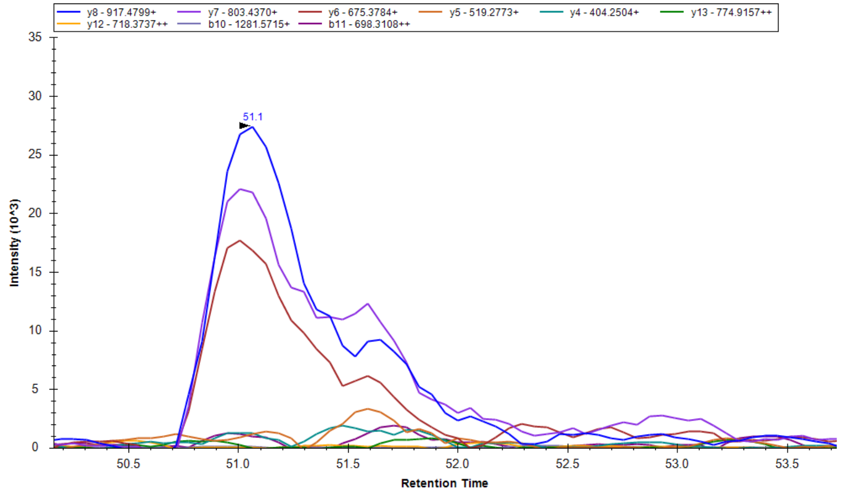

File name: 391-new#B-Round01_All-screening_Positive result.skyd

File name: 391-new#B-Round01_Positive-confirm.skyd

Parent ion m/z and charges: 733.3529+++

NR_026883.2.1
CITDCIQSRFNQRDGSIK

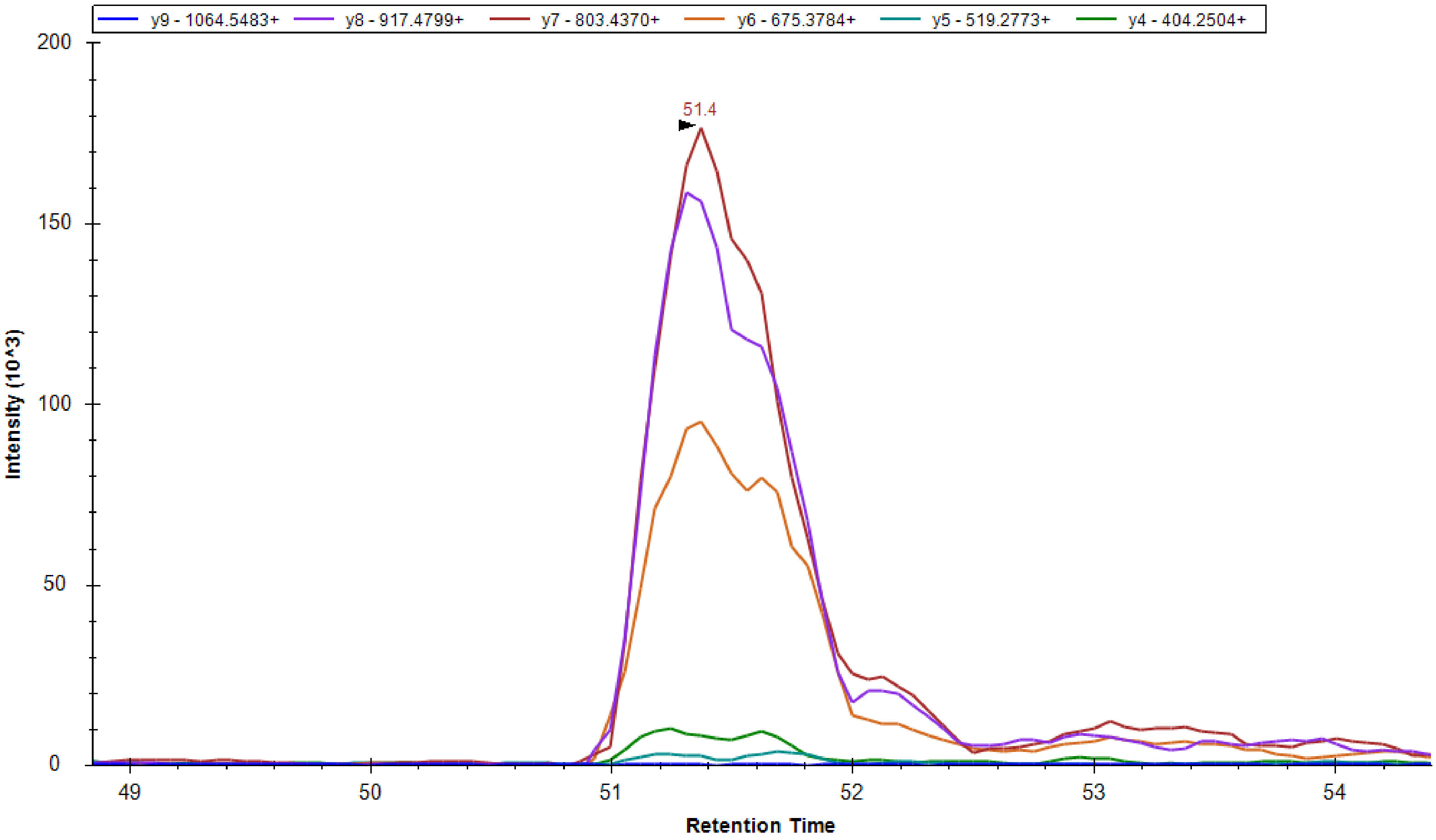

File name: 391-new#A-Round02_Negative-screen.skyd

Parent ion m/z and charges: 733.3529+++

NR_026892.1.6
HLKPPSQPRPVQSQSKEPVFR

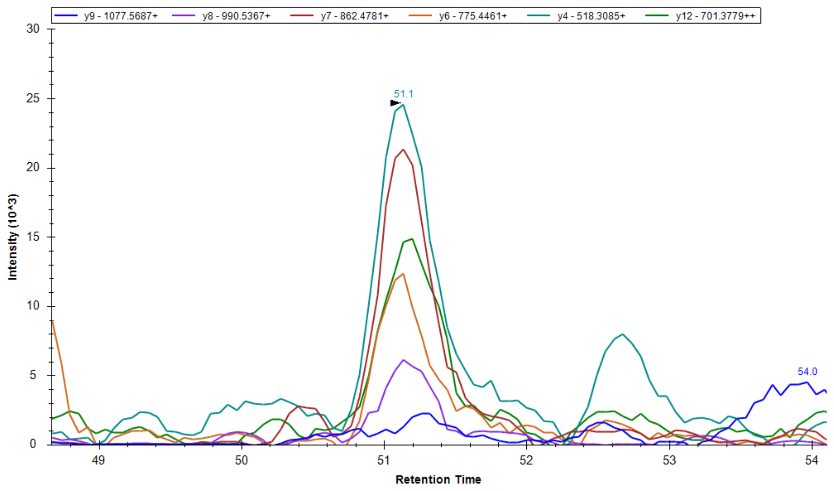

File name: 391-new#A-Round01_All-screening_Positive result.skyd

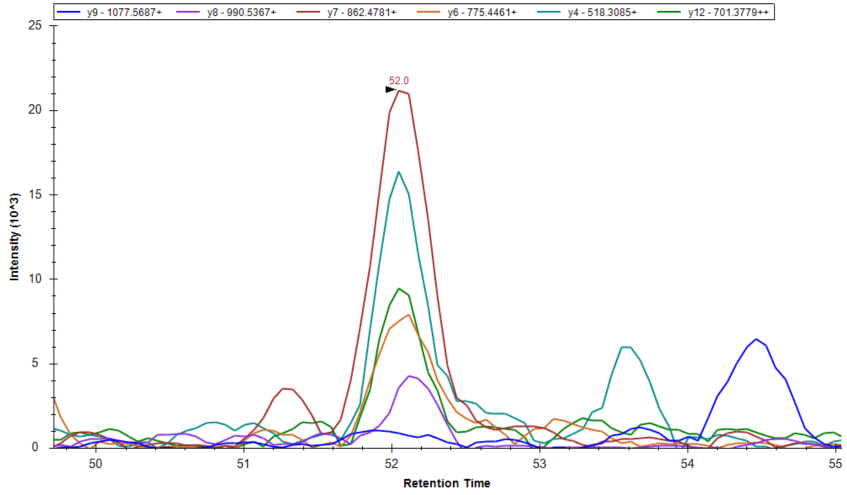

File name: 391-new#A-Round01_Positive-confirm.skyd

Parent ion m/z and charges: 814.7837+++

NR_026892.1.6
HLKPPSQPRPVQSQSKEPVFR

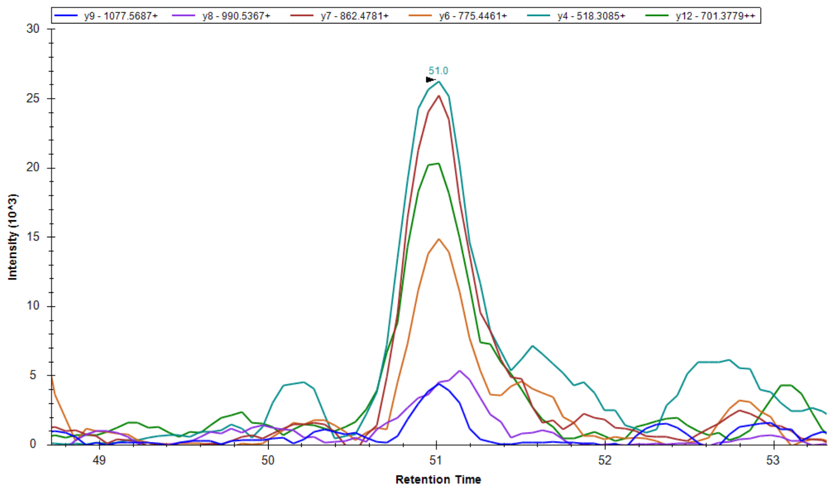

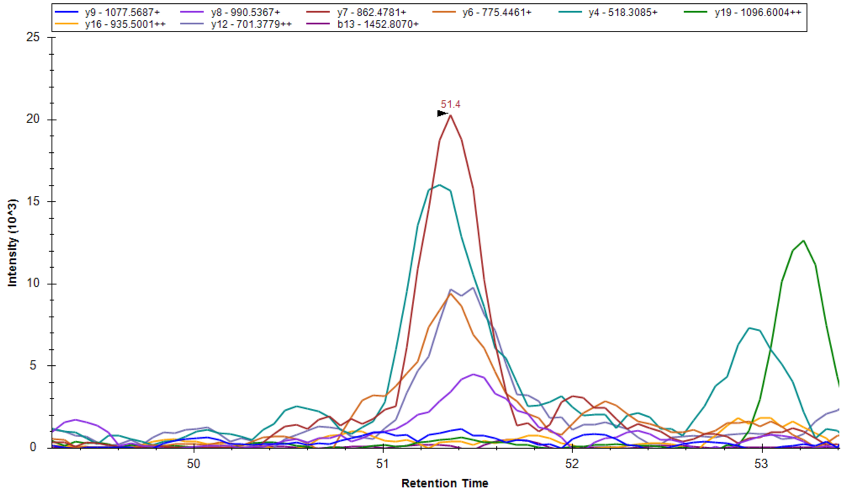

File name: 391-new#B-Round01_All-screening_Positive result.skyd

File name: 391-new#B-Round01_Positive-confirm.skyd

Parent ion m/z and charges: 814.7837+++

NR_026900.2.1
SQPGLESLPLIRPAGFYK

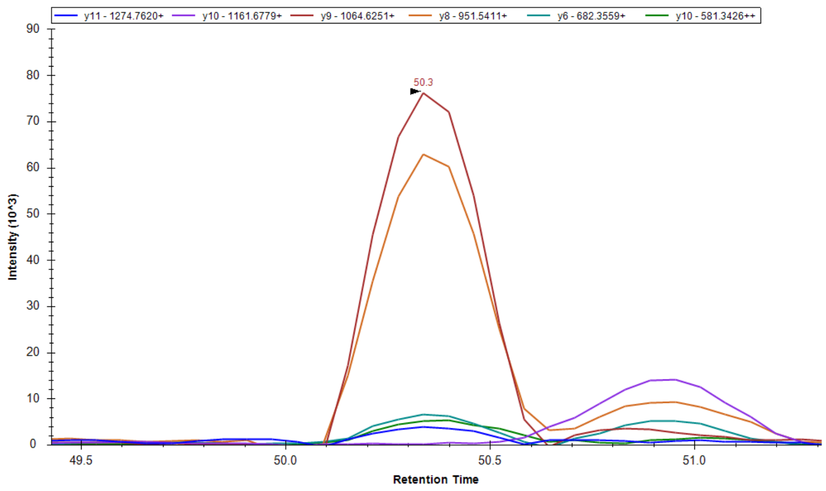

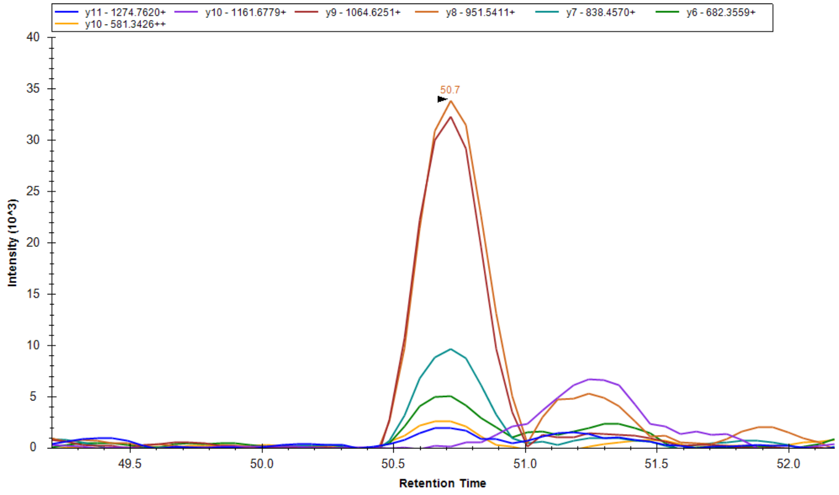

File name: 391-new#B-Round01_All-screening_Positive result.skyd

File name: 391-new#B-Round01_Positive-confirm.skyd

Parent ion m/z and charges: 987.0464++

NR_026941.3.2
MAFICSQCLALRSSGHPLDSSLR

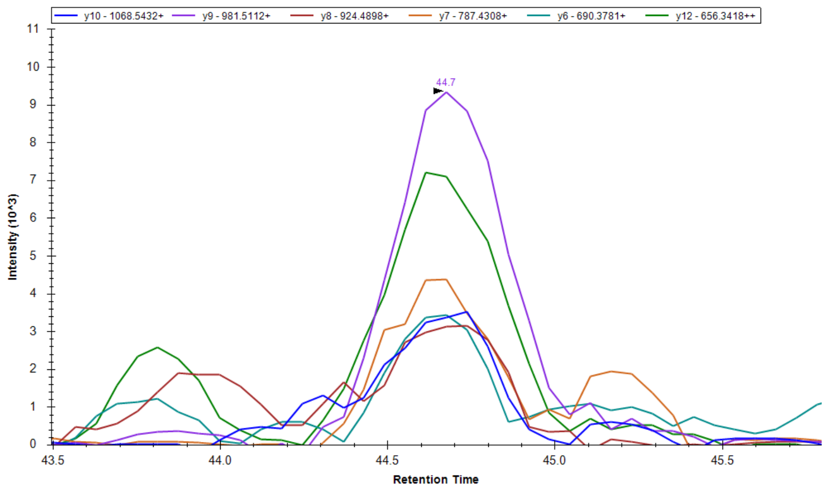

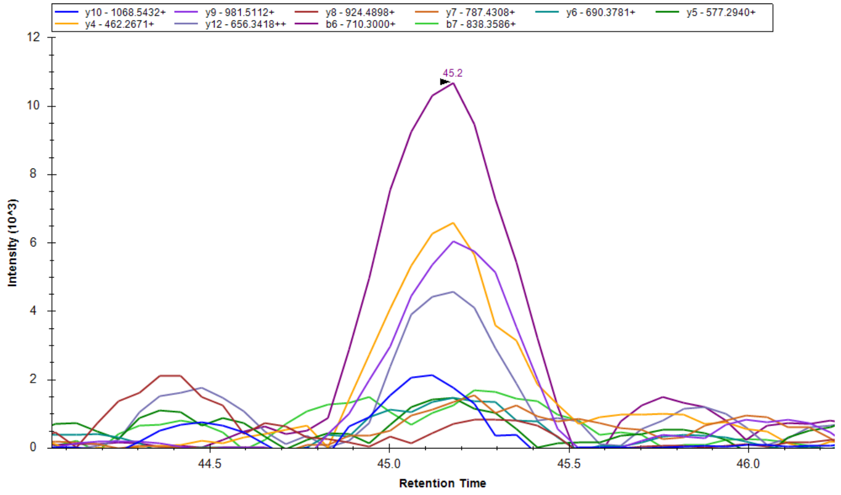

File name: 391-new#B-Round01_All-screening_Positive result.skyd

File name: 391-new#B-Round01_Positive-confirm.skyd

Parent ion m/z and charges: 869.4261+++

NR_026961.2.6
AGGPGDGPSK

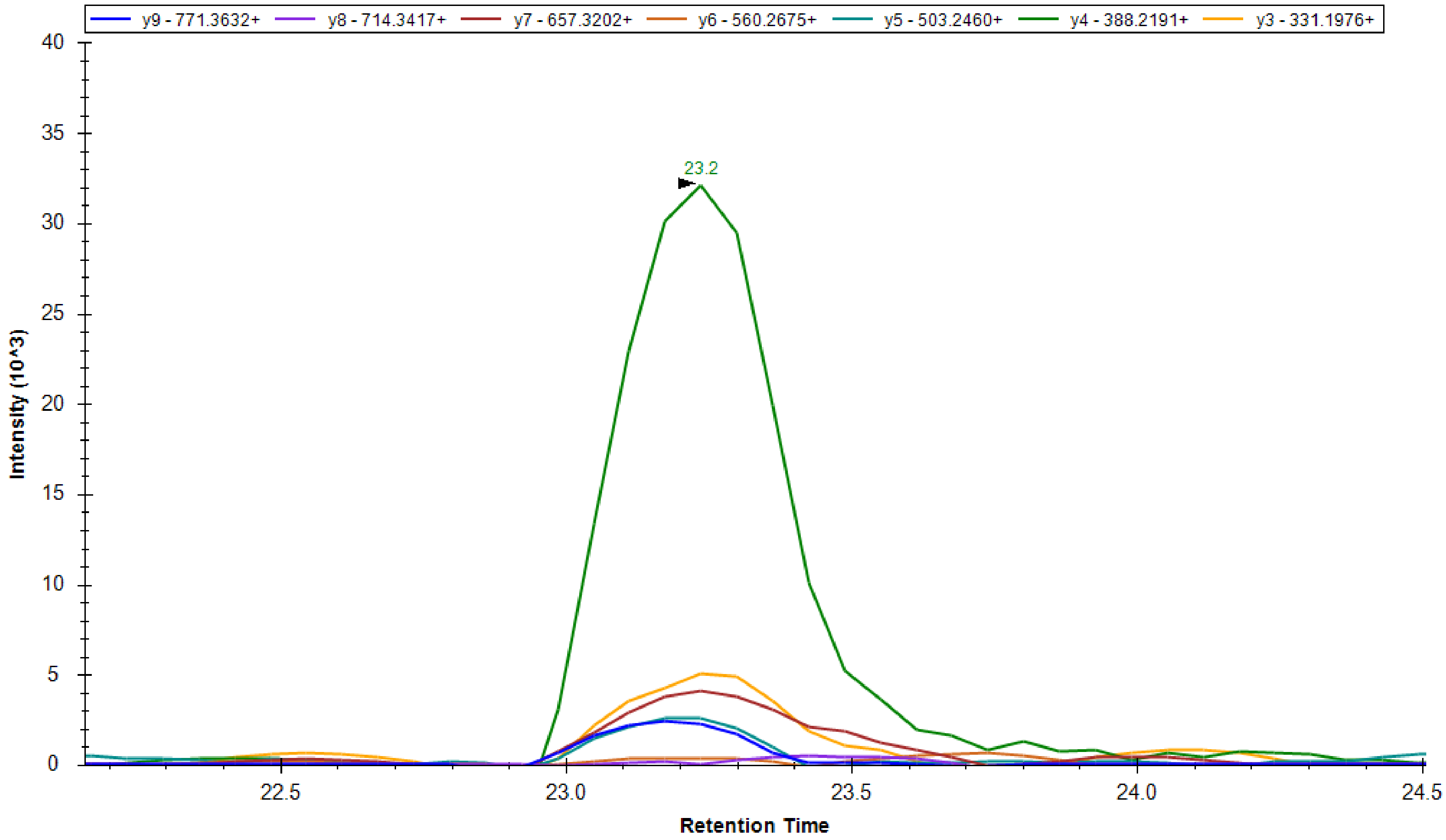

File name: 391-new#A-Round02_Negative-screen.skyd

Parent ion m/z and charges: 421.7038++

NR_026961.2.6
AGGPGDGPSK

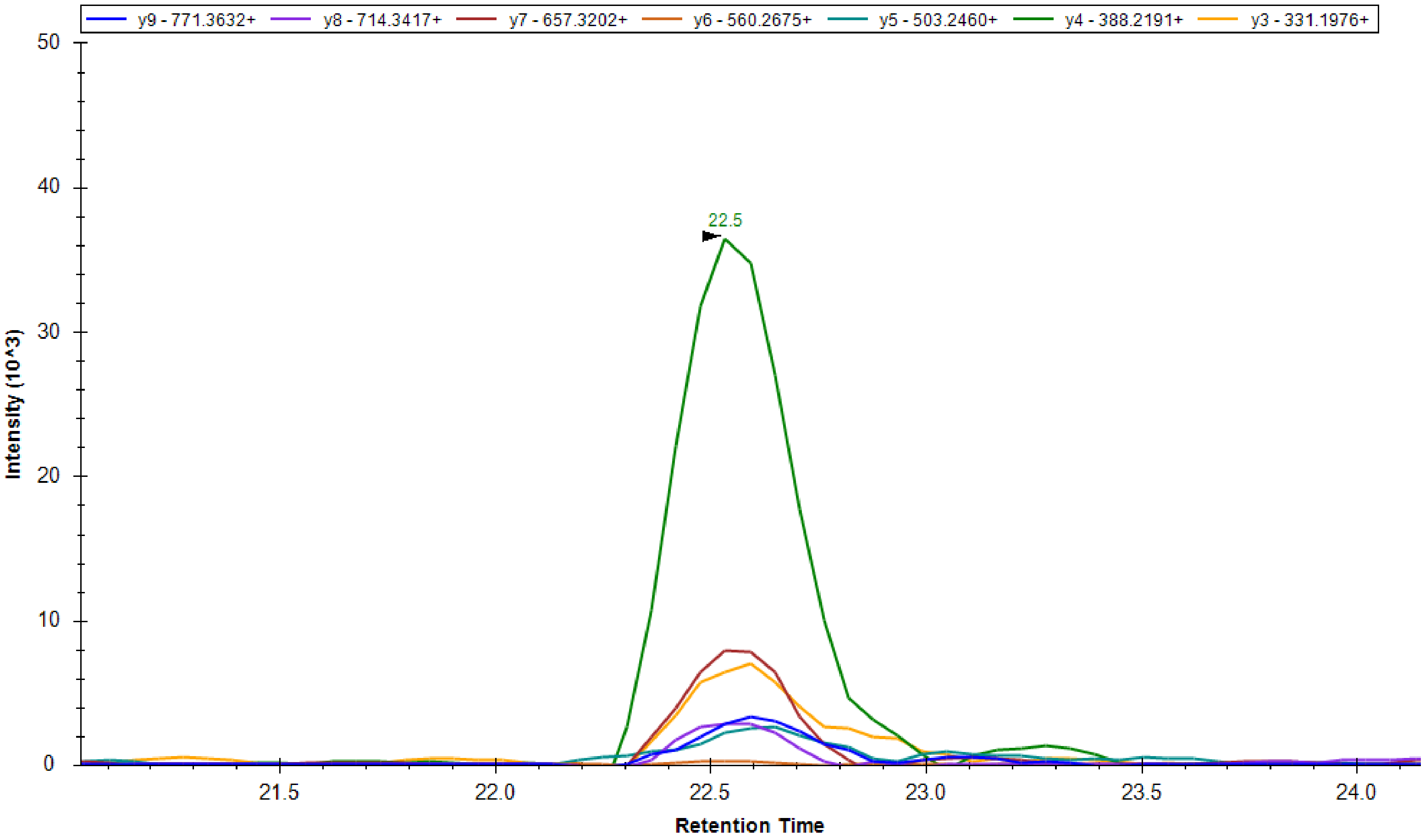

File name: 391-new#B-Round02_Negative-screen.skyd

Parent ion m/z and charges: 421.7038++

NR_026999.2.7
MVSGQWAPPRPAWASR

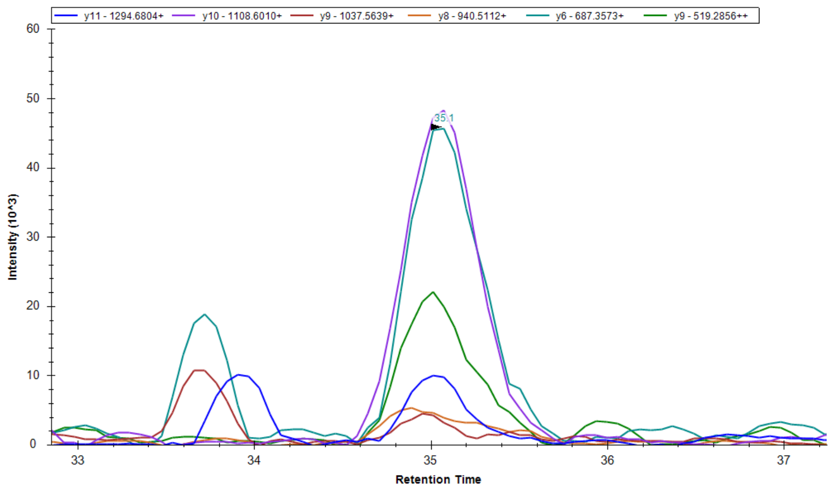

File name: 391-new#B-Round01_All-screening_Positive result.skyd

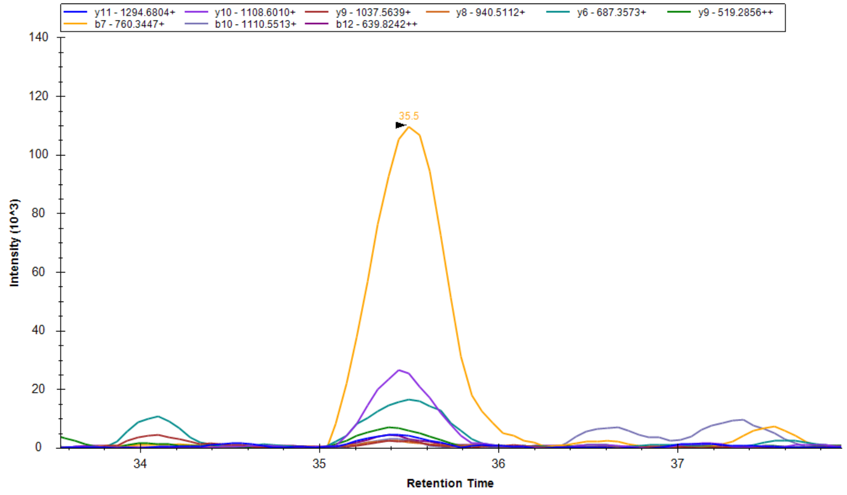

File name: 391-new#B-Round01_Positive-confirm.skyd

Parent ion m/z and charges: 898.9543++

NR_026999.2.7
MVSGQWAPPRPAWASR

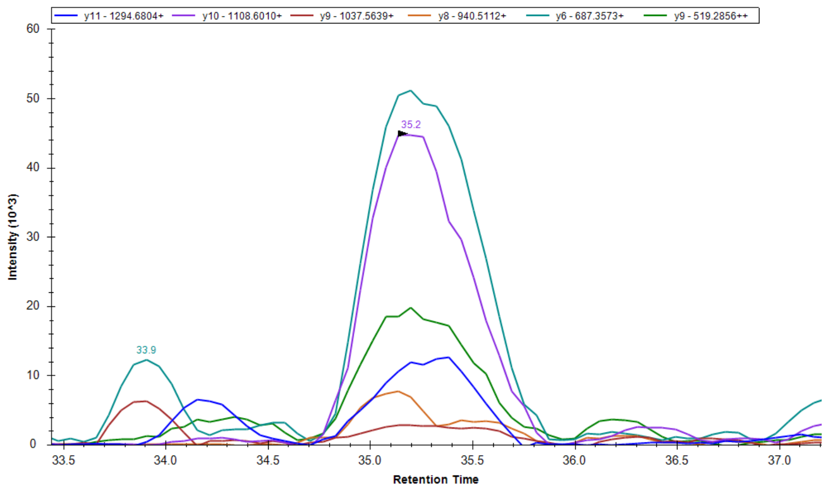

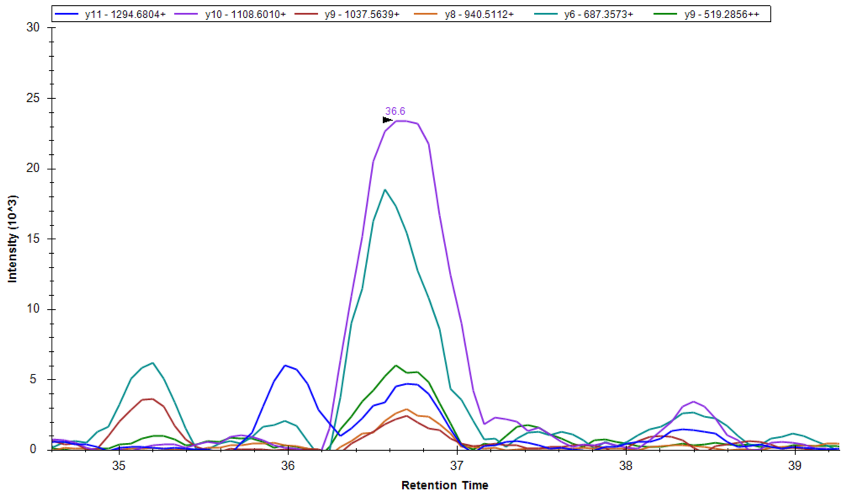

File name: 391-new#A-Round01_All-screening_Positive result.skyd

File name: 391-new#A-Round01_Positive-confirm.skyd

Parent ion m/z and charges: 898.9543++

NR_027023.2.3
CGSPPSAPPPPPDRGER

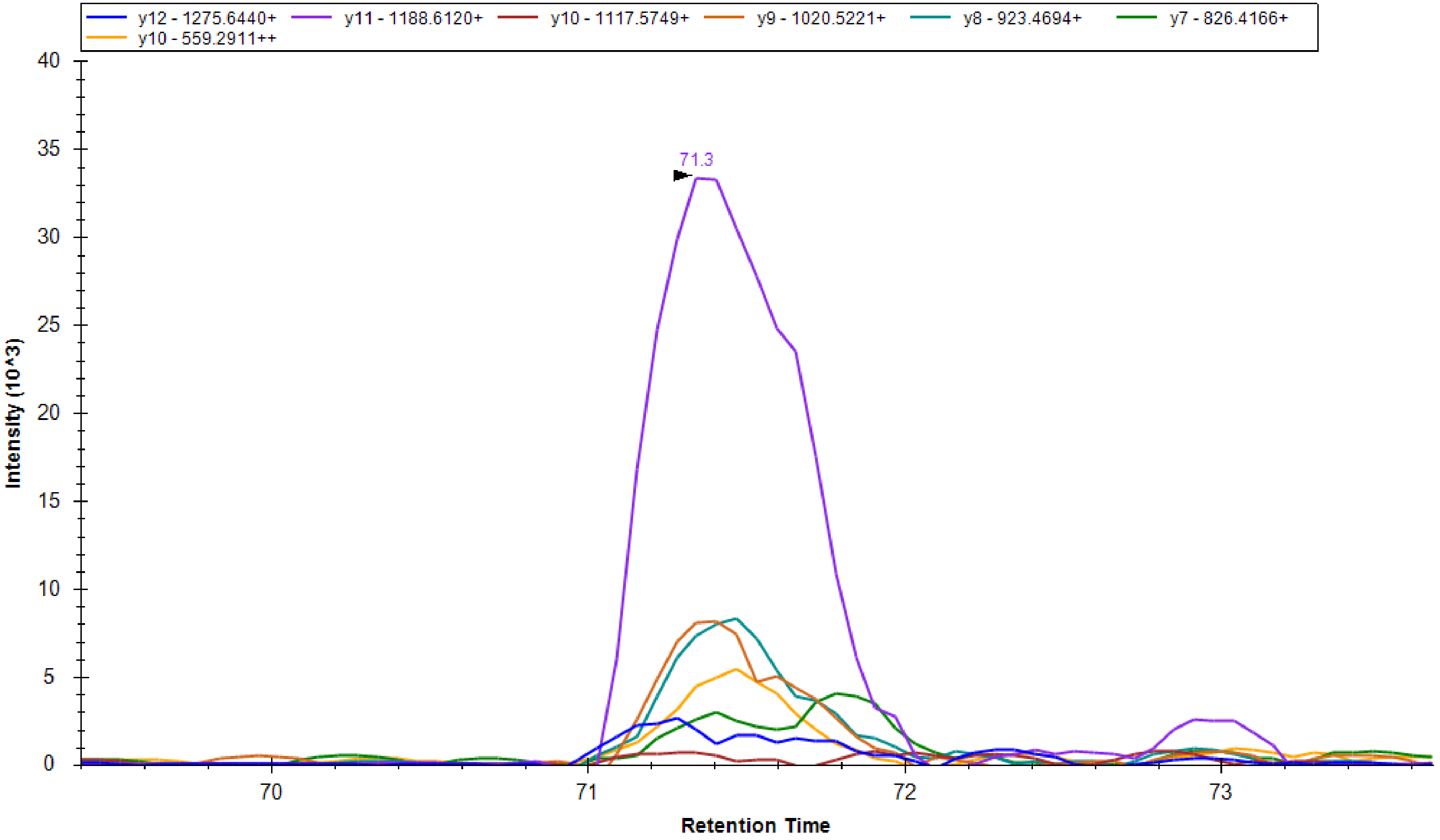

File name: 391-new#A-Round02_Negative-screen.skyd

Parent ion m/z and charges: 887.4205++

NR_027156.1.1
RRGADGVSDGR

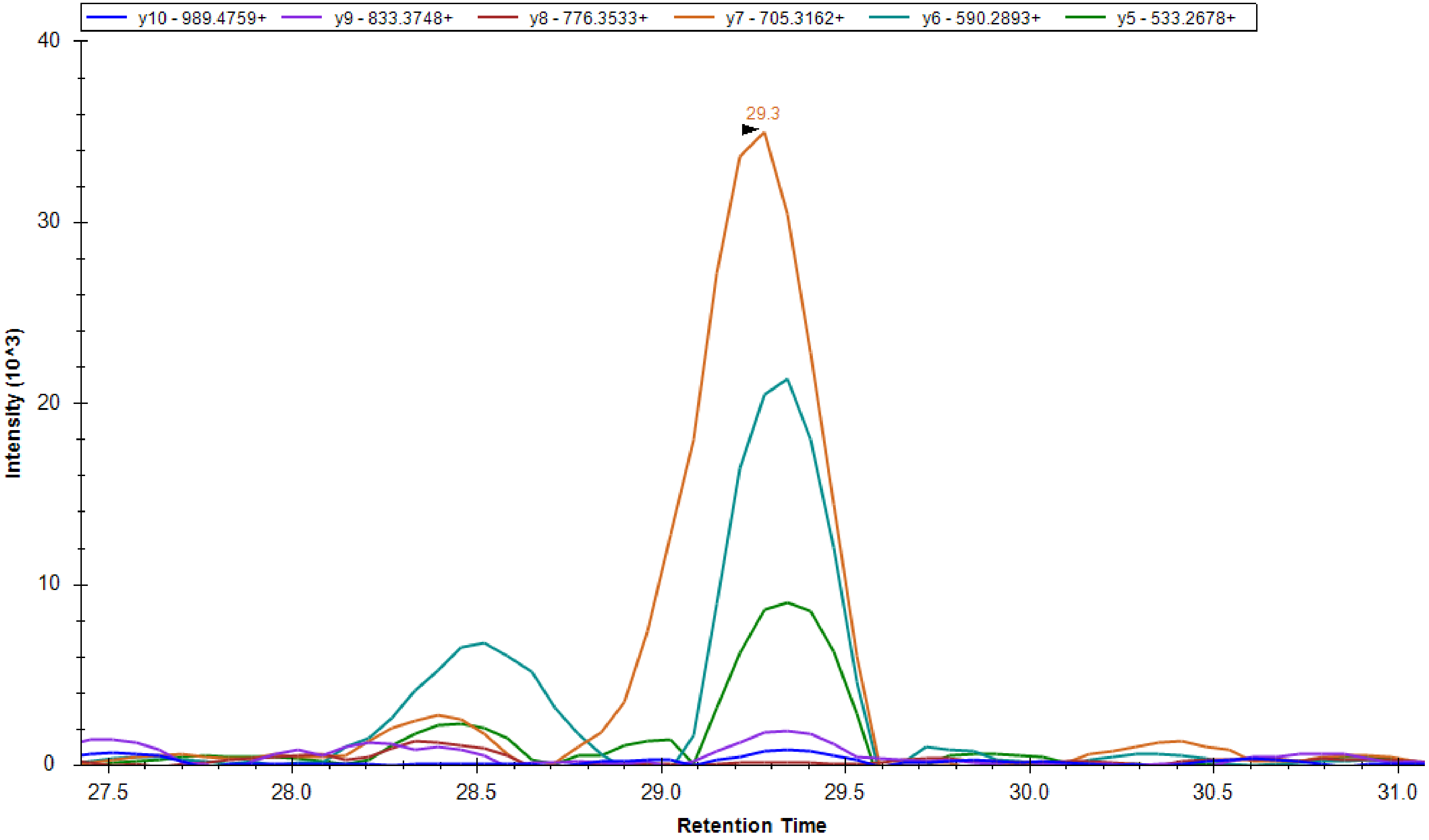

File name: 391-new#A-Round02_Negative-screen.skyd

Parent ion m/z and charges: 573.2921++

NR_027237.3.4
MSPFLSSTQLSPSDPR

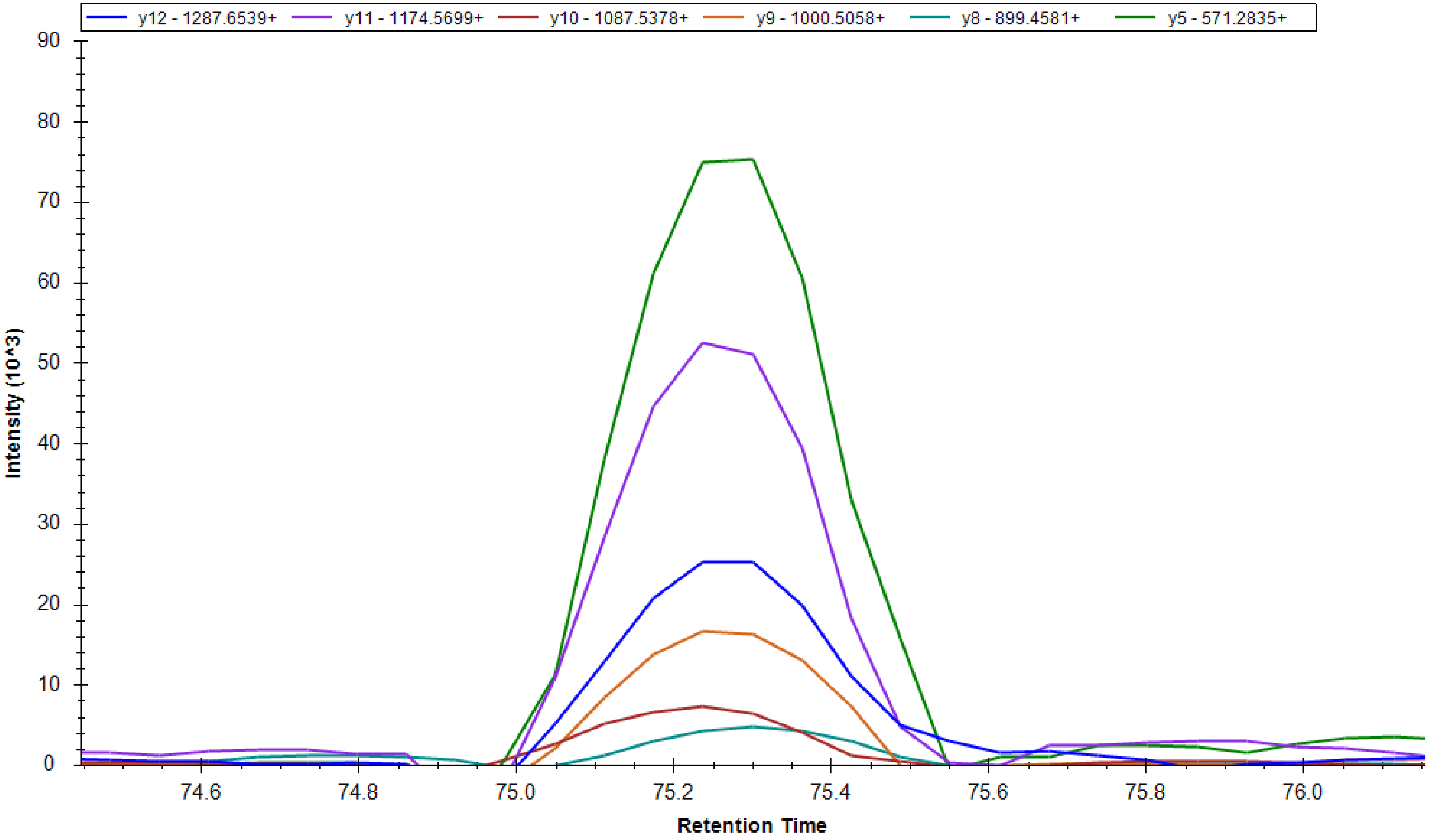

File name: 391-new#A-Round02_Negative-screen.skyd

Parent ion m/z and charges: 875.4274++

NR_027242.3.1
ATAALWPPLHPDGAWPGR

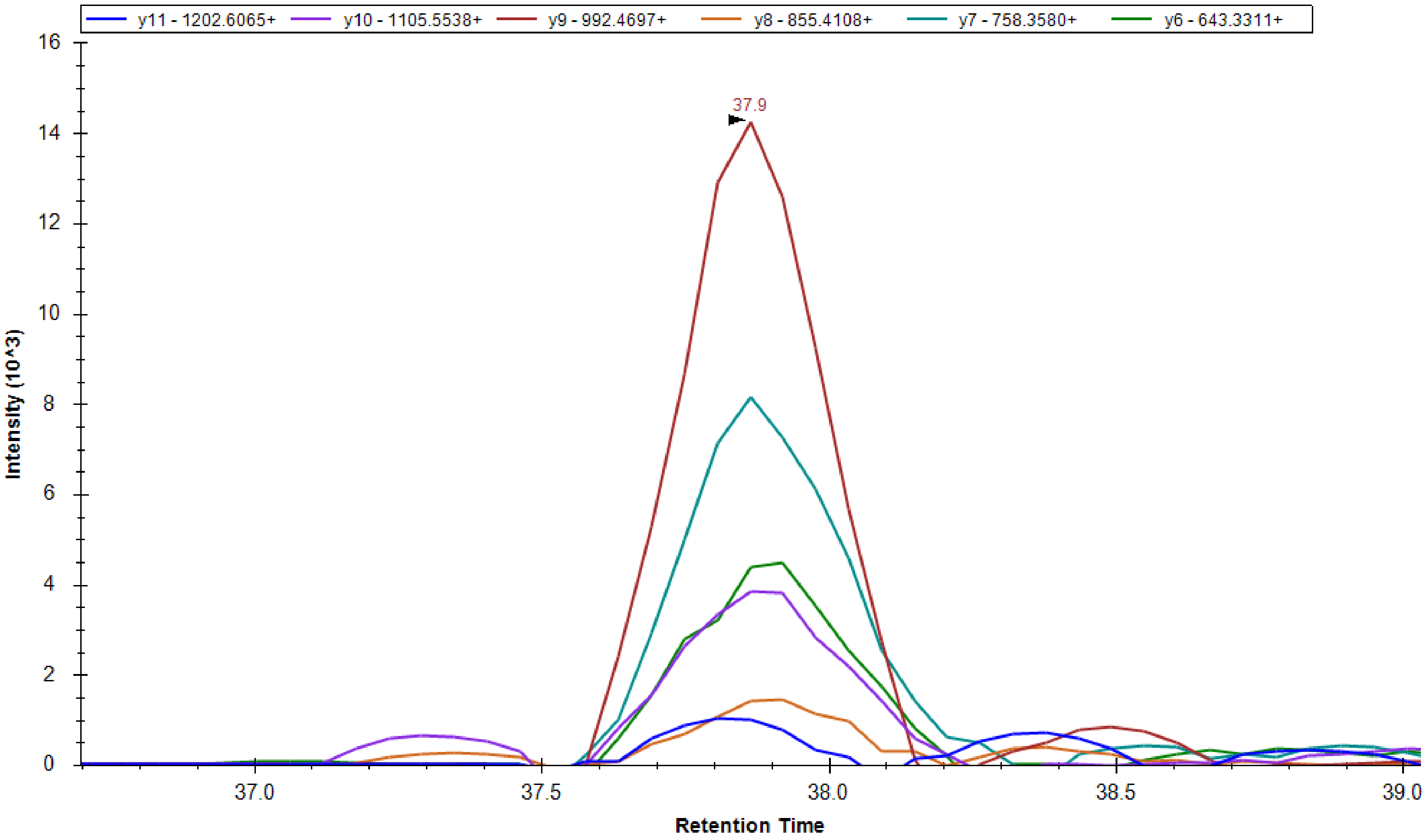

File name: 391-new#B-Round02_Negative-screen.skyd

Parent ion m/z and charges: 956.9945++

NR_027247.1.6
LCYITLDFEKEMATAASSSSLEK

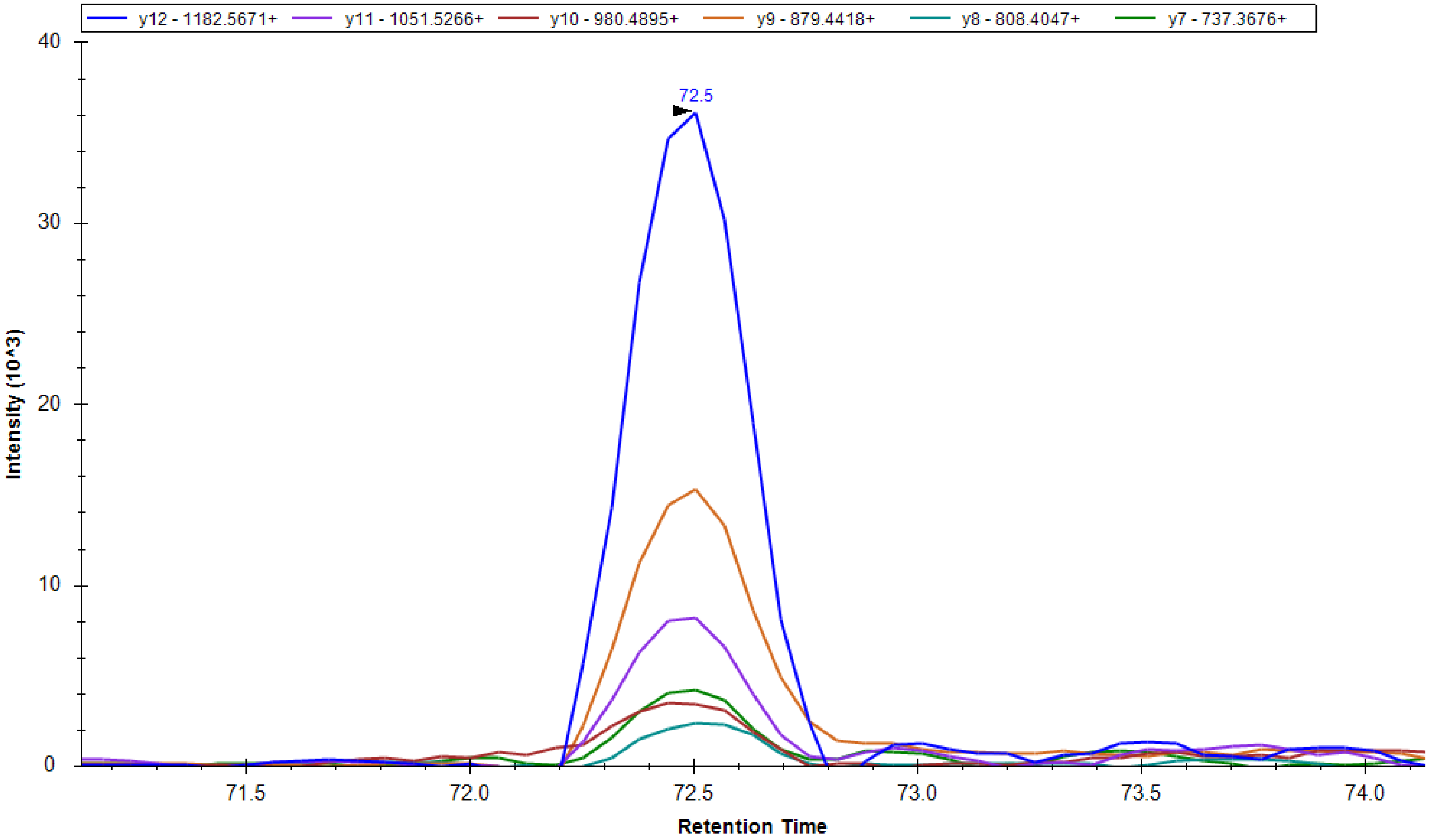

File name: 391-new#A-Round02_Negative-screen.skyd

Parent ion m/z and charges: 865.4170+++

NR_027252.3.3
MALETKTDEER

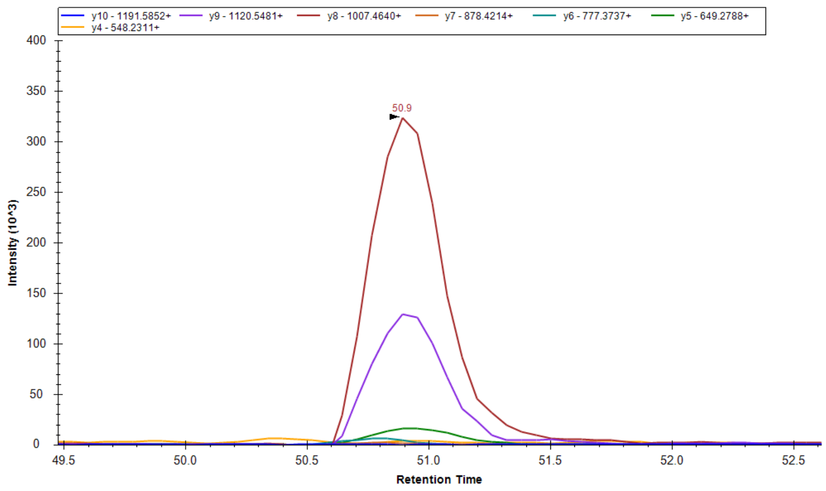

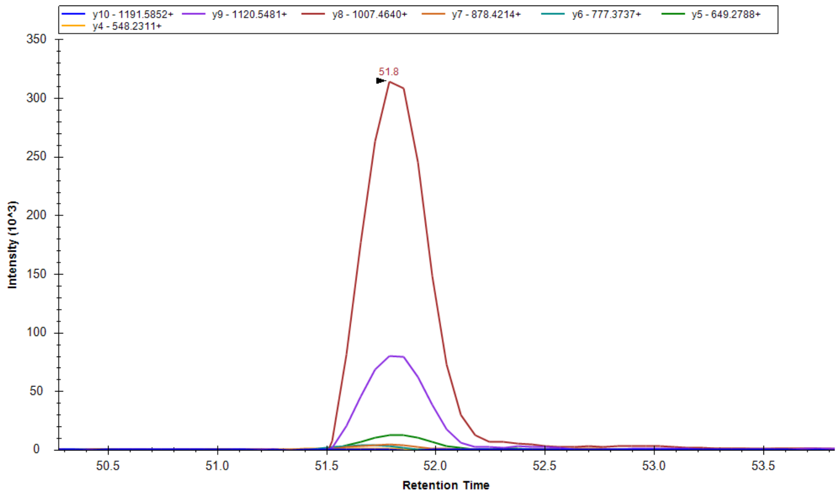

File name: 391-new#A-Round01_All-screening_Positive result.skyd

File name: 391-new#A-Round01_Positive-confirm.skyd

Parent ion m/z and charges: 661.8165++

NR_027252.3.3
MALETKTDEER

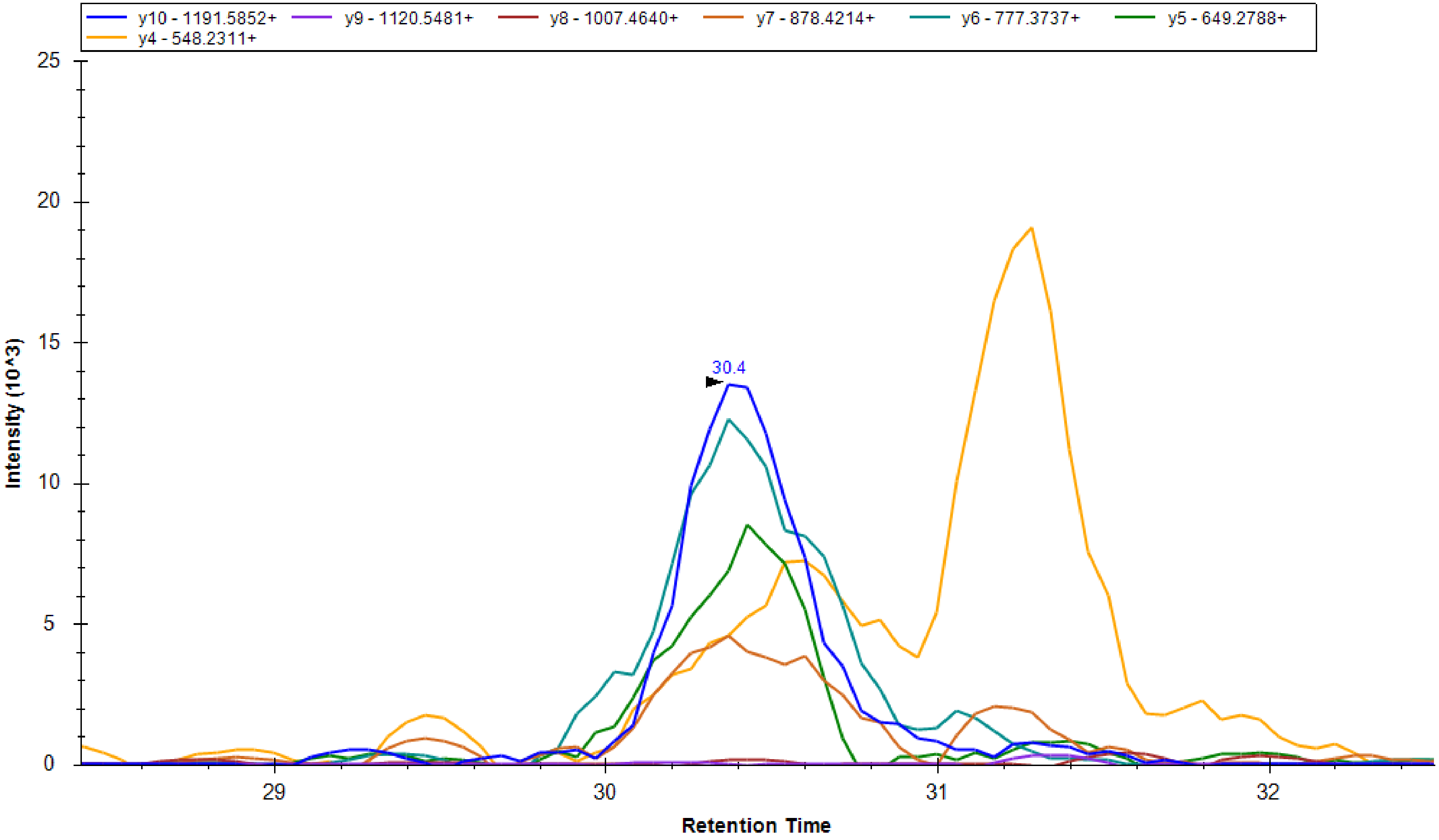

File name: 391-new#B-Round02_Negative-screen.skyd

Parent ion m/z and charges: 661.8165++

NR_027291.1.2
MSIMRWPVIIMIR

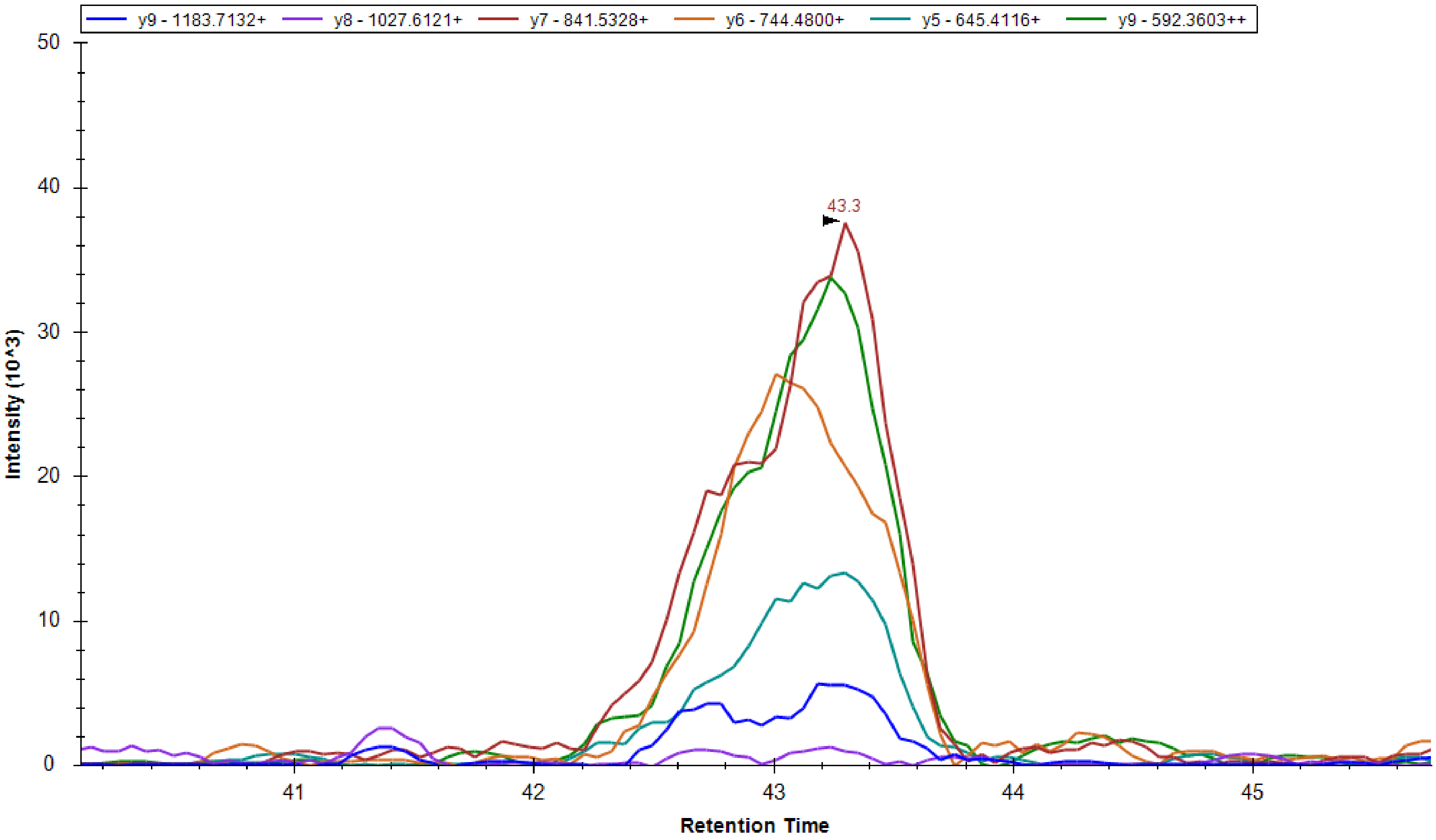

File name: 391-new#B-Round02_Negative-screen.skyd

Parent ion m/z and charges: 823.4588++

NR_027436.2.1
MRSLPSLCSR

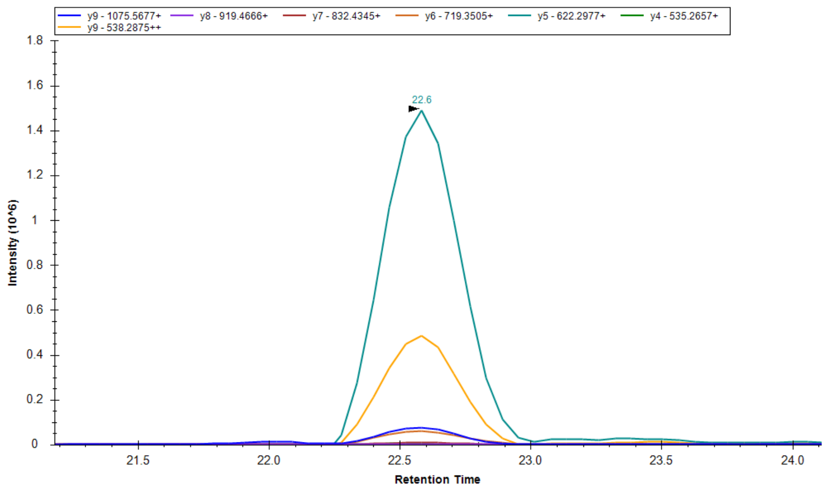

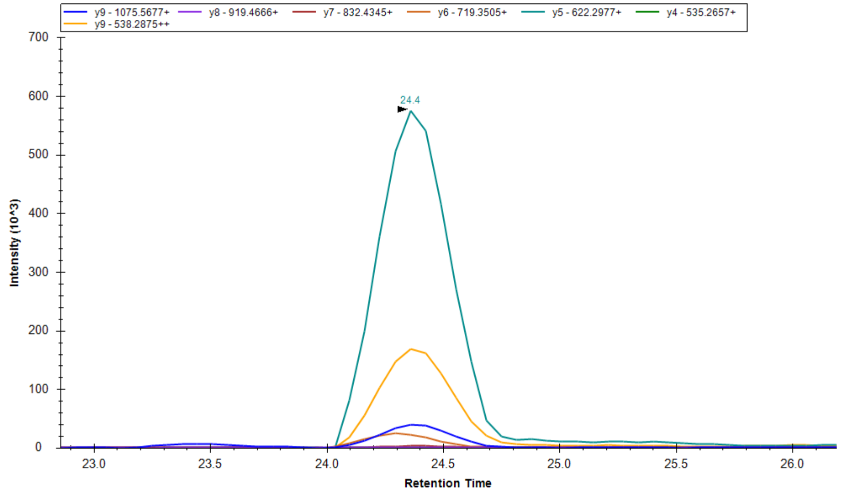

File name: 391-new#A-Round01_All-screening_Positive result.skyd

File name: 391-new#A-Round01_Positive-confirm.skyd

Parent ion m/z and charges: 603.8077++

NR_027436.2.1
MRSLPSLCSR

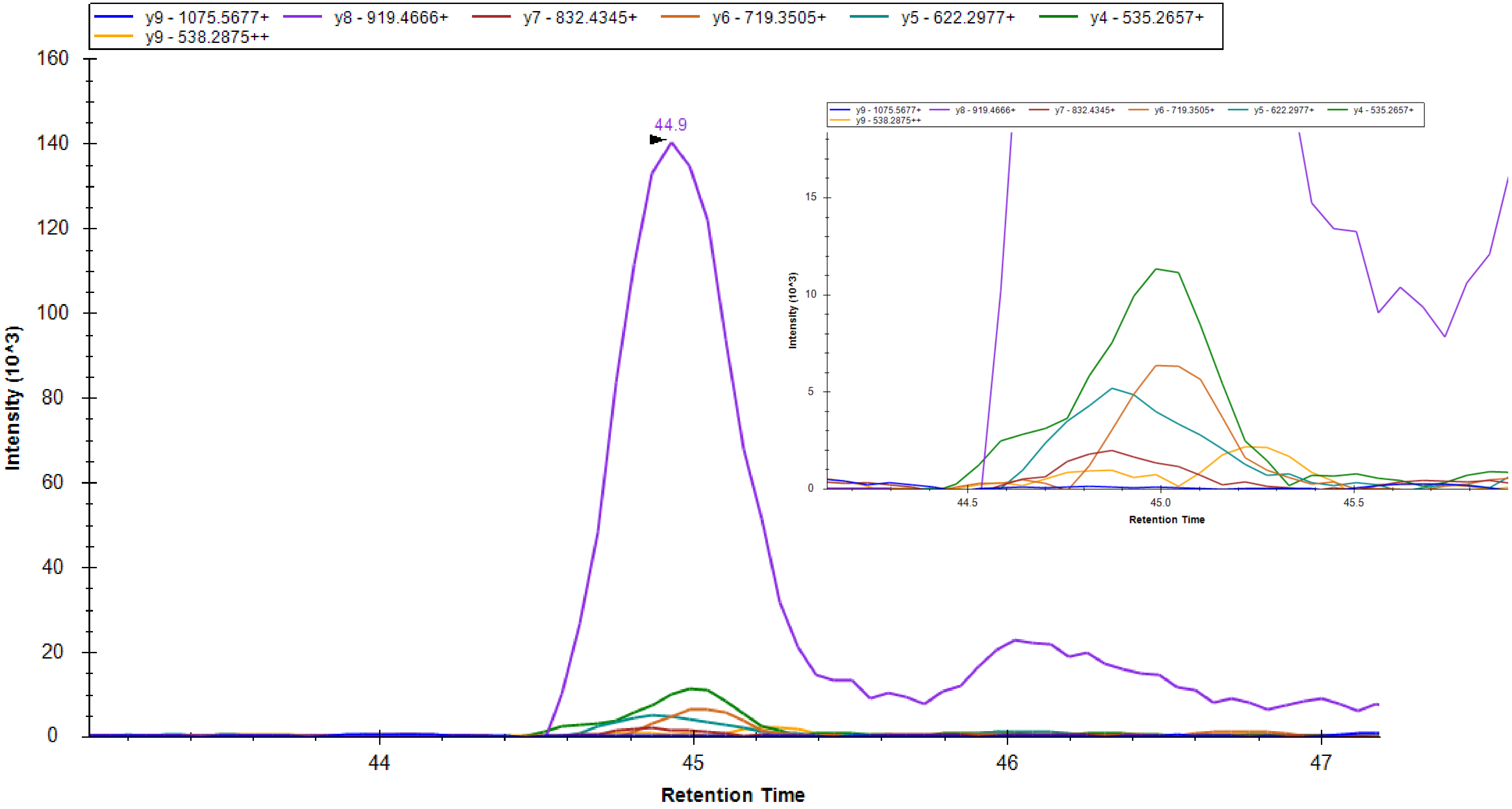

File name: 391-new#B-Round02_Negative-screen.skyd

Parent ion m/z and charges: 603.8077++

NR_027451.2.6
LKRLPVYDK

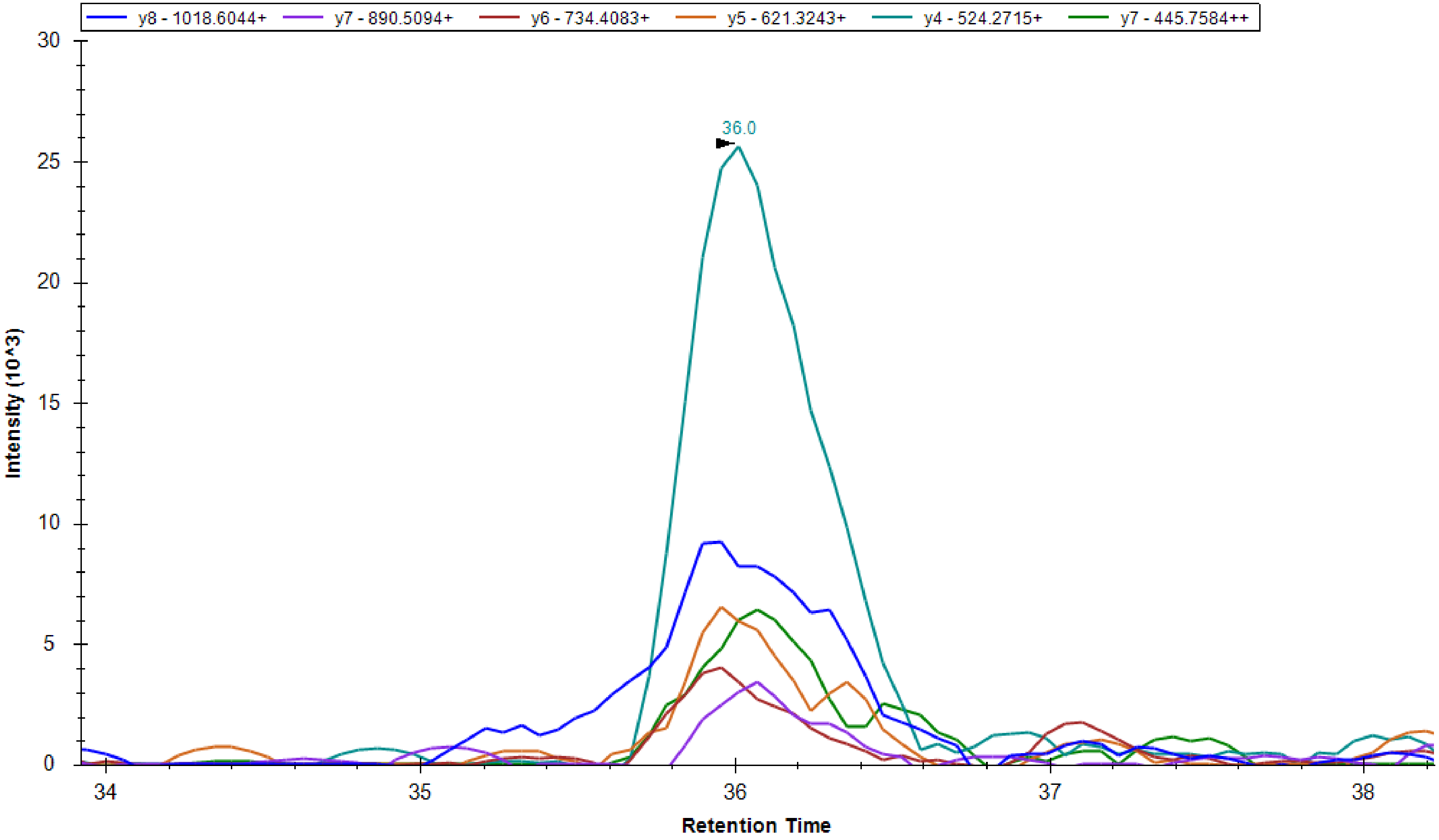

File name: 391-new#B-Round02_Negative-screen.skyd

Parent ion m/z and charges: 566.3479++

NR_027622.3.8
MAGPIMVMSTEASSAGASAVVAD

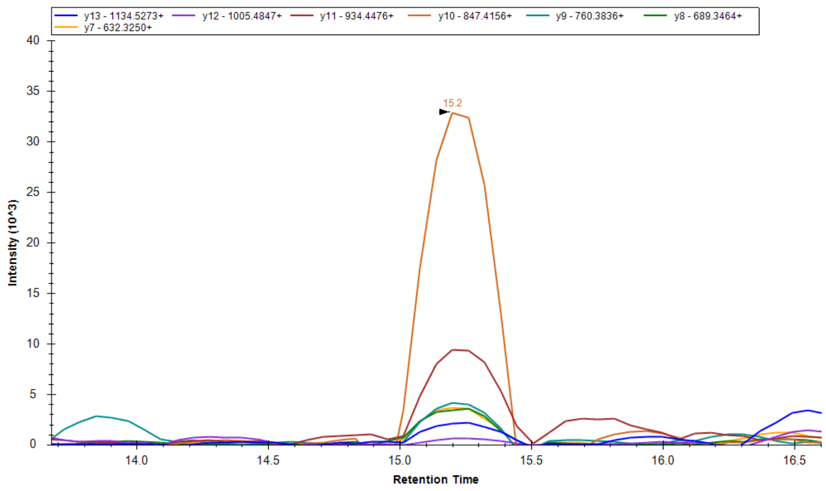

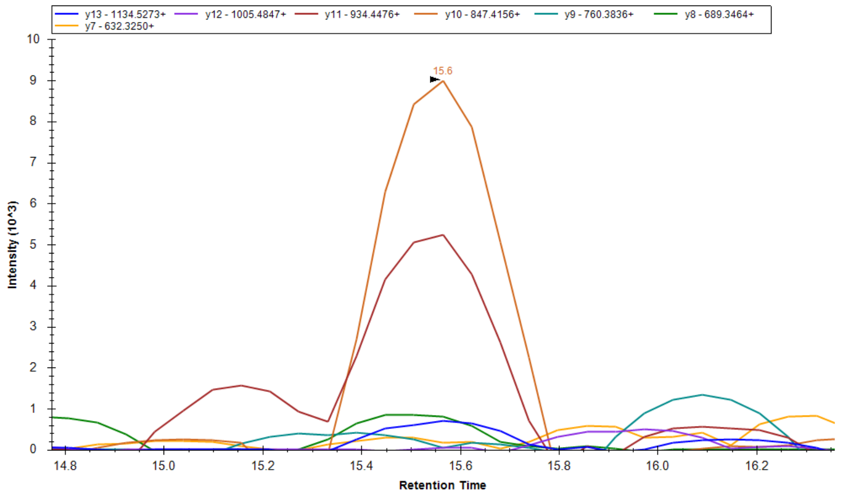

File name: 391-new#B-Round01_All-screening_Positive result.skyd

File name: 391-new#B-Round01_Positive-confirm.skyd

Parent ion m/z and charges: 718.3356+++

NR_027655.2.2
MNLLCIAPMFYSALMTSGR

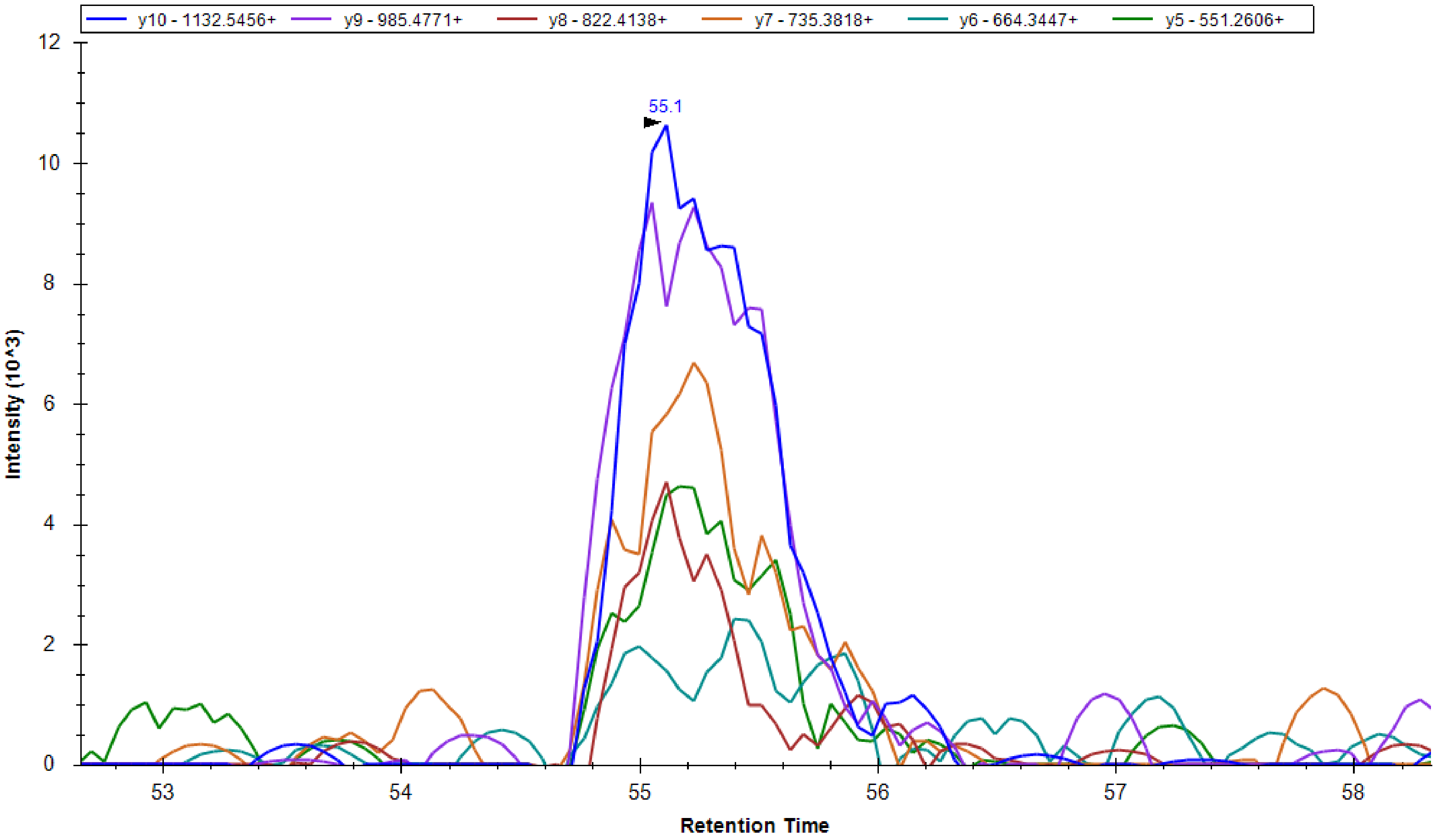

File name: 391-new#B-Round02_Negative-screen.skyd

Parent ion m/z and charges: 726.0189+++

NR_027715.3.1
IITQIPIISR

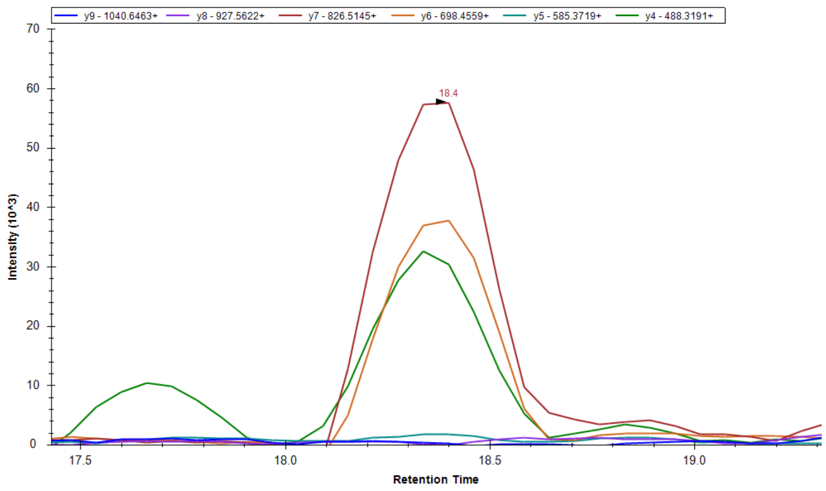

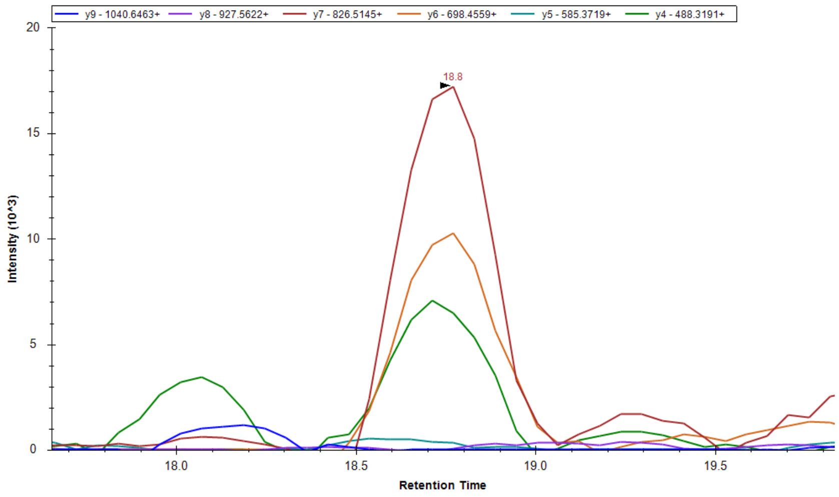

File name: 391-new#B-Round01_All-screening_Positive result.skyd

File name: 391-new#B-Round01_Positive-confirm.skyd

Parent ion m/z and charges: 577.3688++

NR_027715.3.1
IITQIPIISR

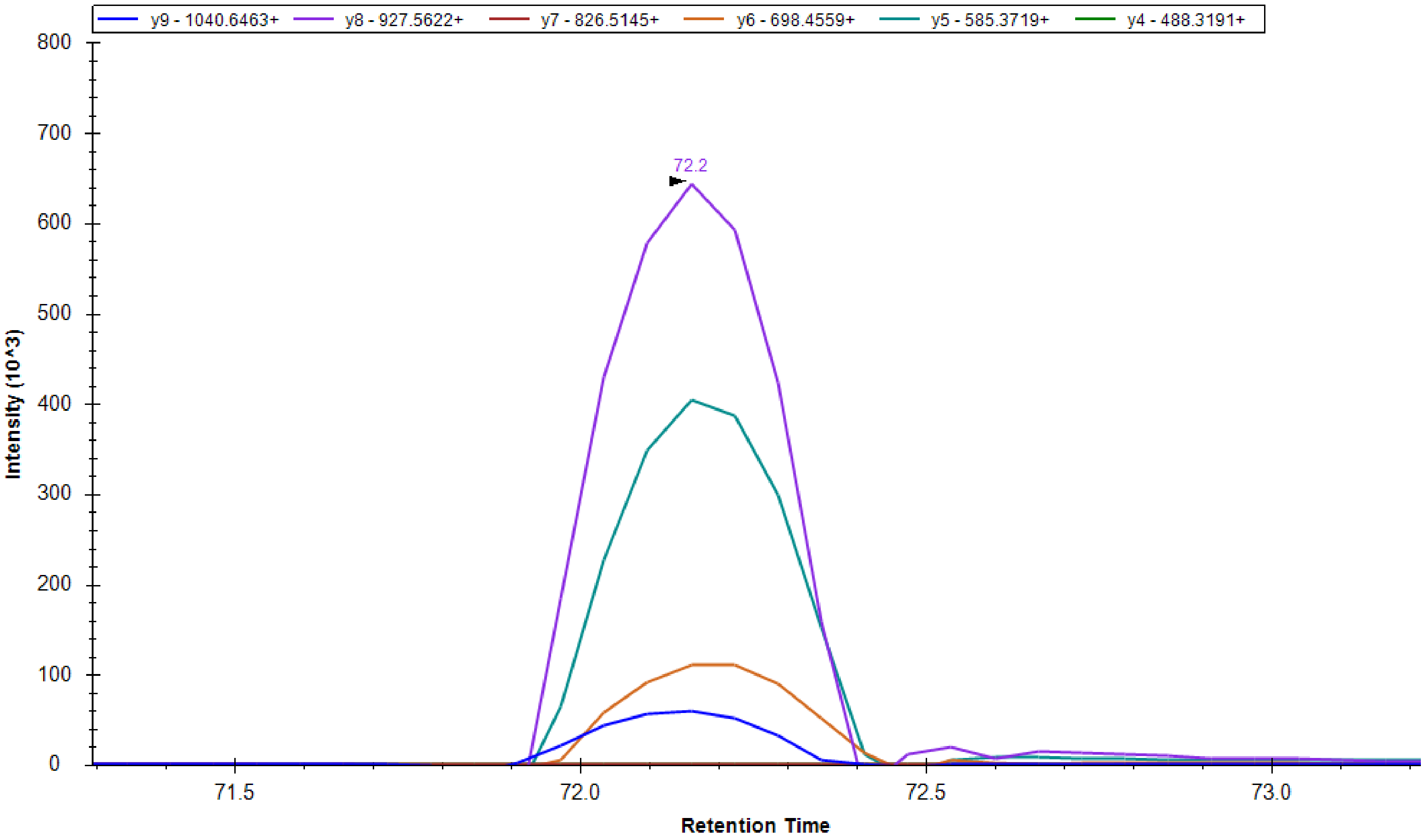

File name: 391-new#A-Round02_Negative-screen.skyd

Parent ion m/z and charges: 577.3688++

NR_027995.2.9
IMKISWANHNSSR

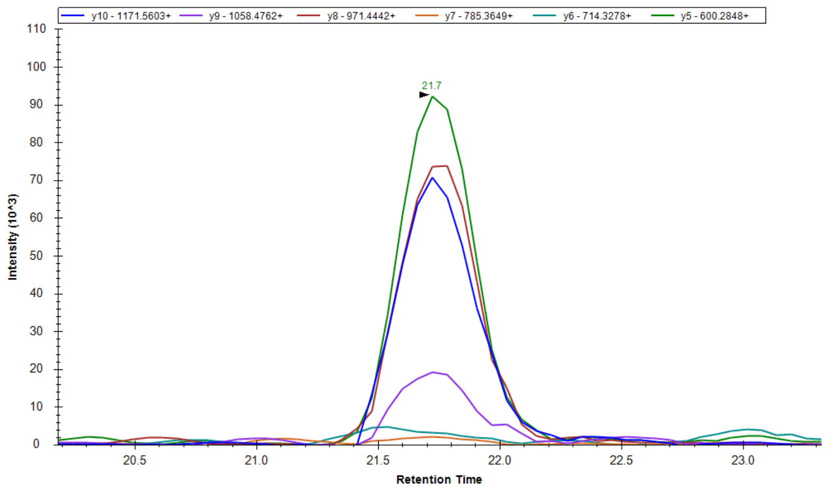

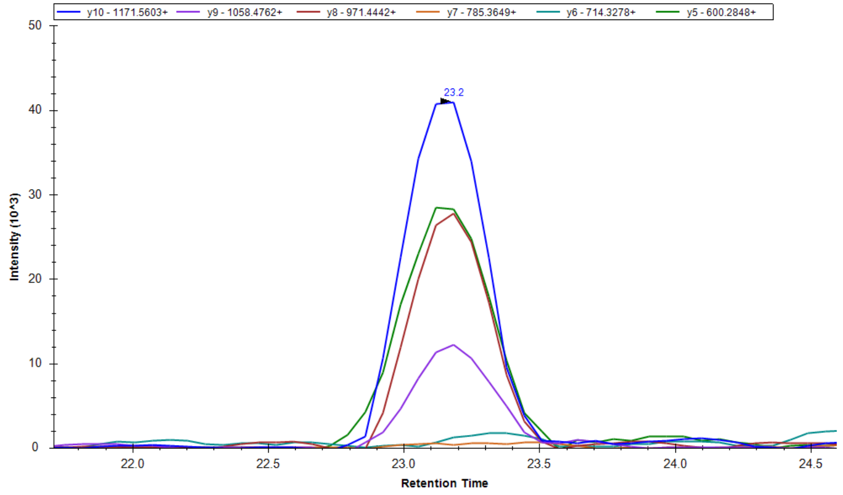

File name: 391-new#A-Round01_All-screening_Positive result.skyd

File name: 391-new#A-Round01_Positive-confirm.skyd

Parent ion m/z and charges: 772.3935++

NR_027995.2.9
IMKISWANHNSSR

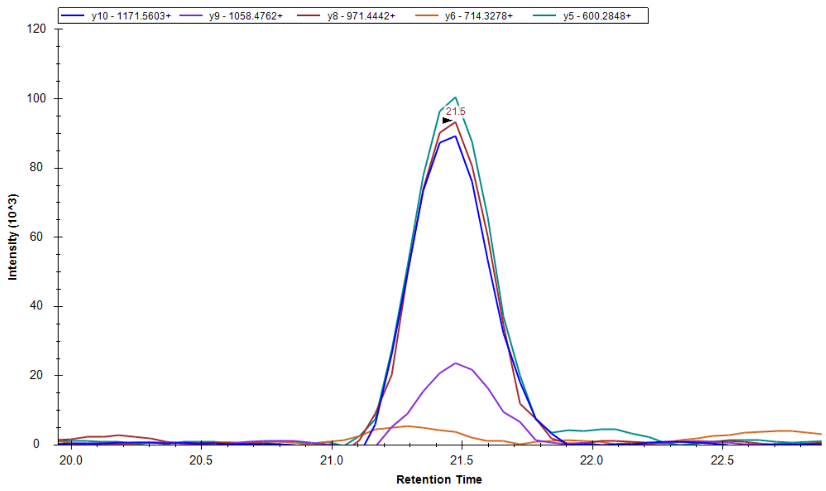

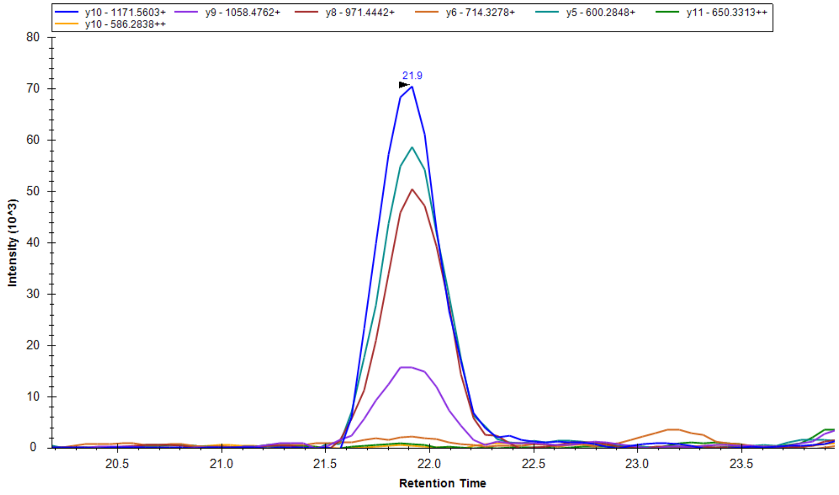

File name: 391-new#B-Round01_All-screening_Positive result.skyd

File name: 391-new#B-Round01_Positive-confirm.skyd

Parent ion m/z and charges: 772.3935++

NR_028038.1.6
IGTQTPRFPPWPHPWAK

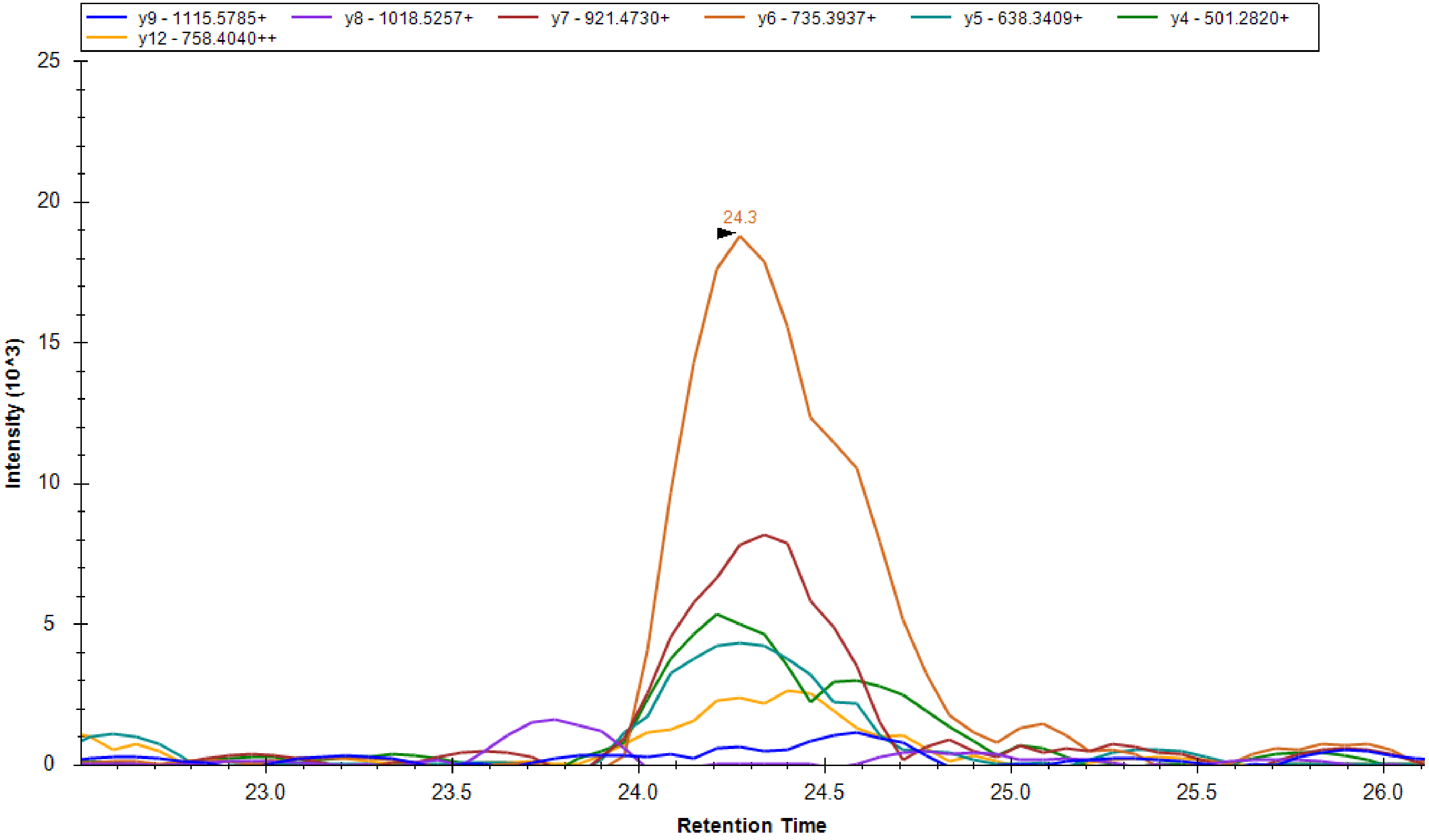

File name: 391-new#A-Round02_Negative-screen.skyd

Parent ion m/z and charges: 672.6916+++

NR_028038.1.6
IGTQTPRFPPWPHPWAK

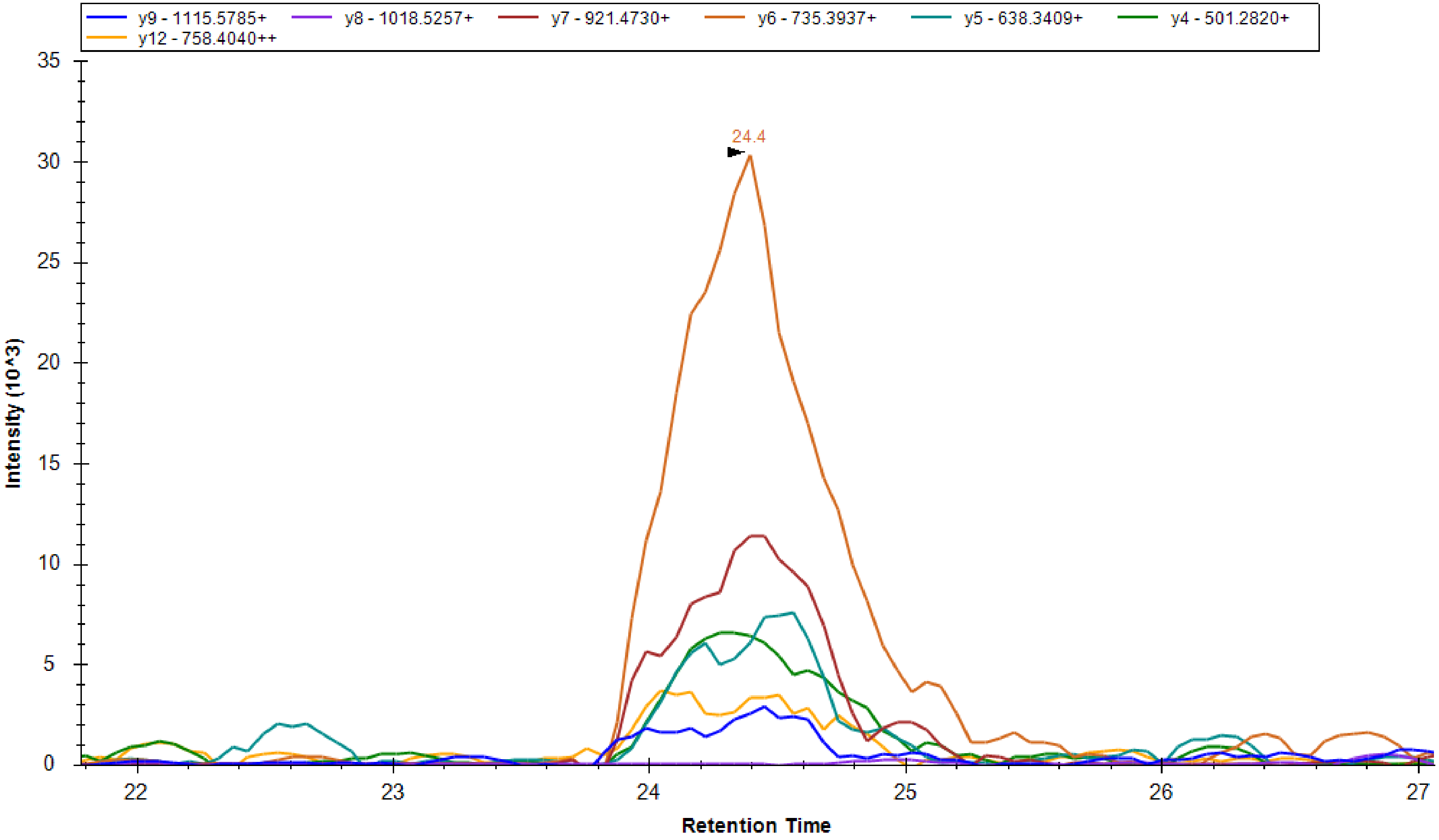

File name: 391-new#B-Round02_Negative-screen.skyd

Parent ion m/z and charges: 672.6916+++

NR_028350.1.6
MMRMMMSIPR

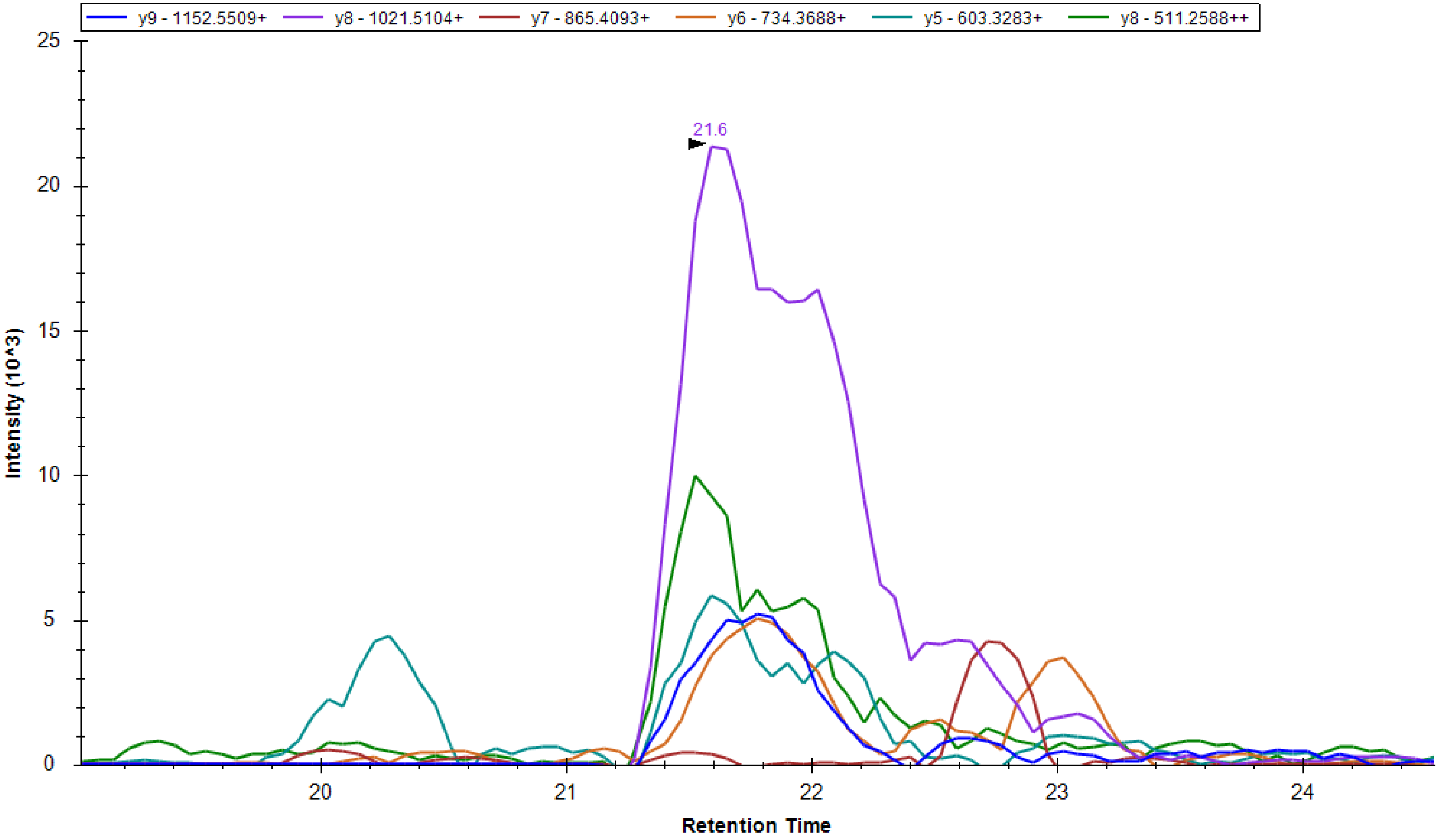

File name: 391-new#A-Round02_Negative-screen.skyd

Parent ion m/z and charges: 642.2993++

NR_028350.1.6
MMRMMMSIPR

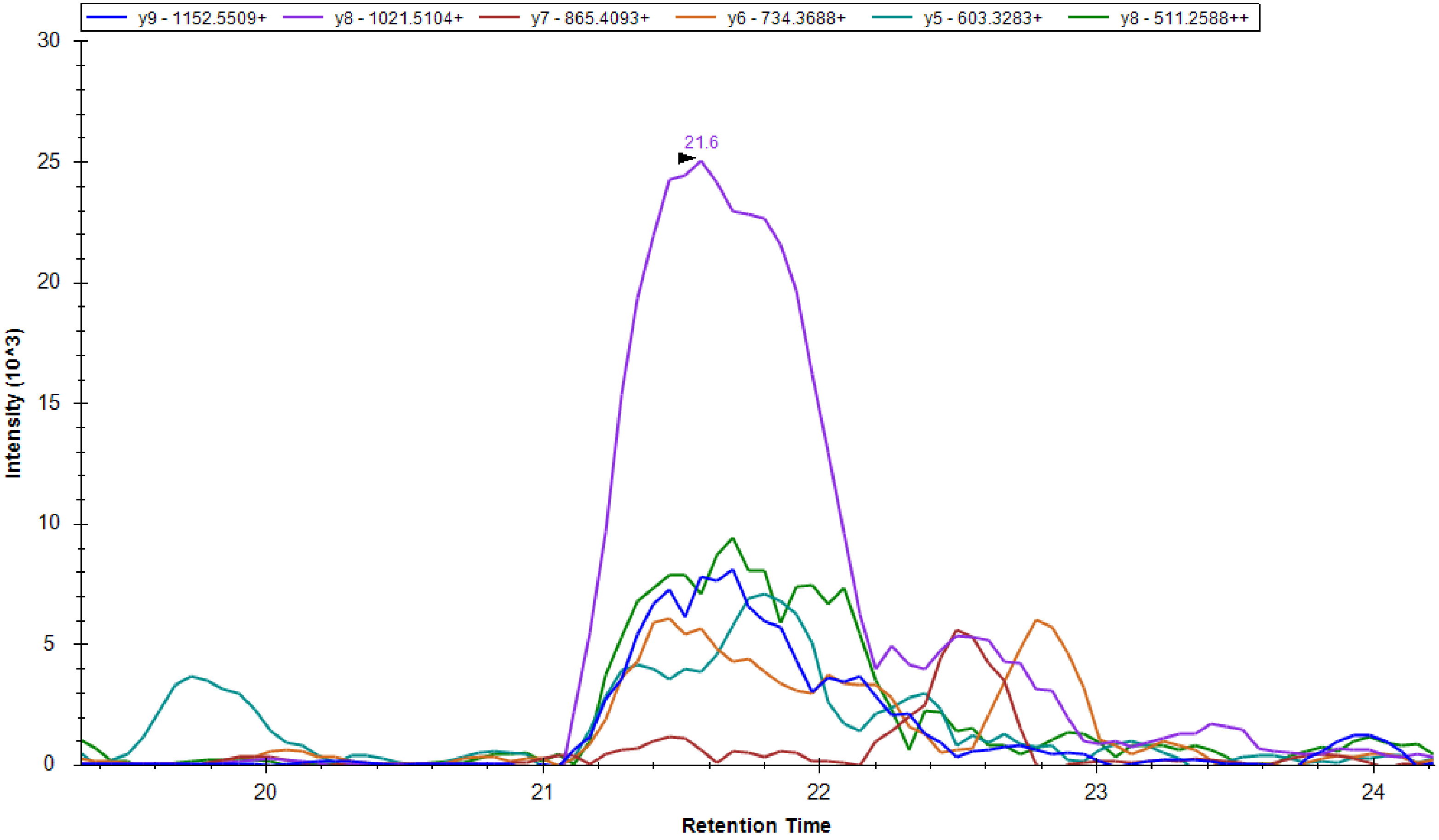

File name: 391-new#B-Round02_Negative-screen.skyd

Parent ion m/z and charges: 642.2993++

NR_028415.2.4
MLLQQGQGPCQPPTRHTR

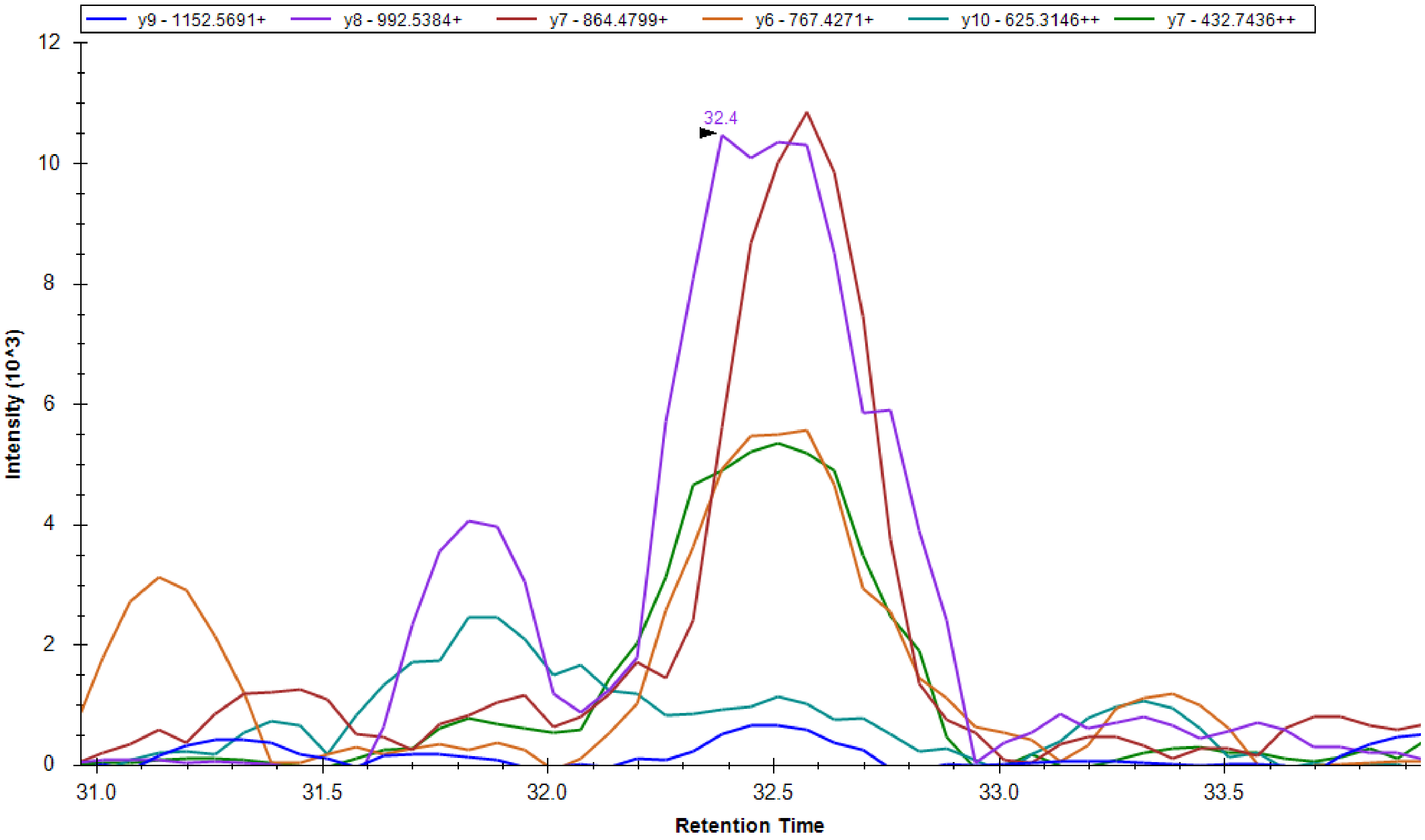

File name: 391-new#A-Round02_Negative-screen.skyd

Parent ion m/z and charges: 702.3546+++

NR_033344.3.6
LQMIVSQSSFISPK

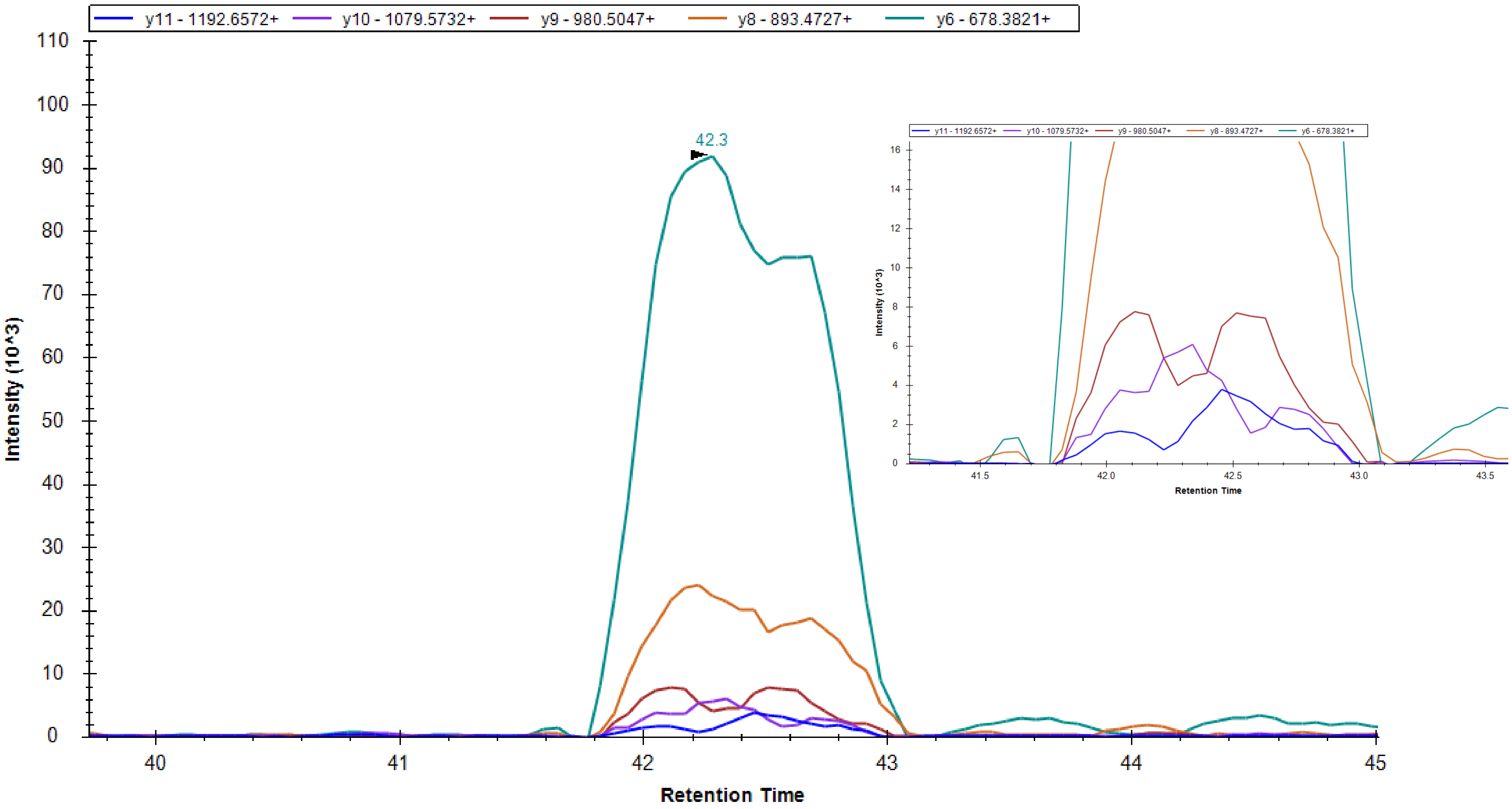

File name: 391-new#B-Round02_Negative-screen.skyd

Parent ion m/z and charges: 782.9238++

NR_033344.3.6
LQMIVSQSSFISPK

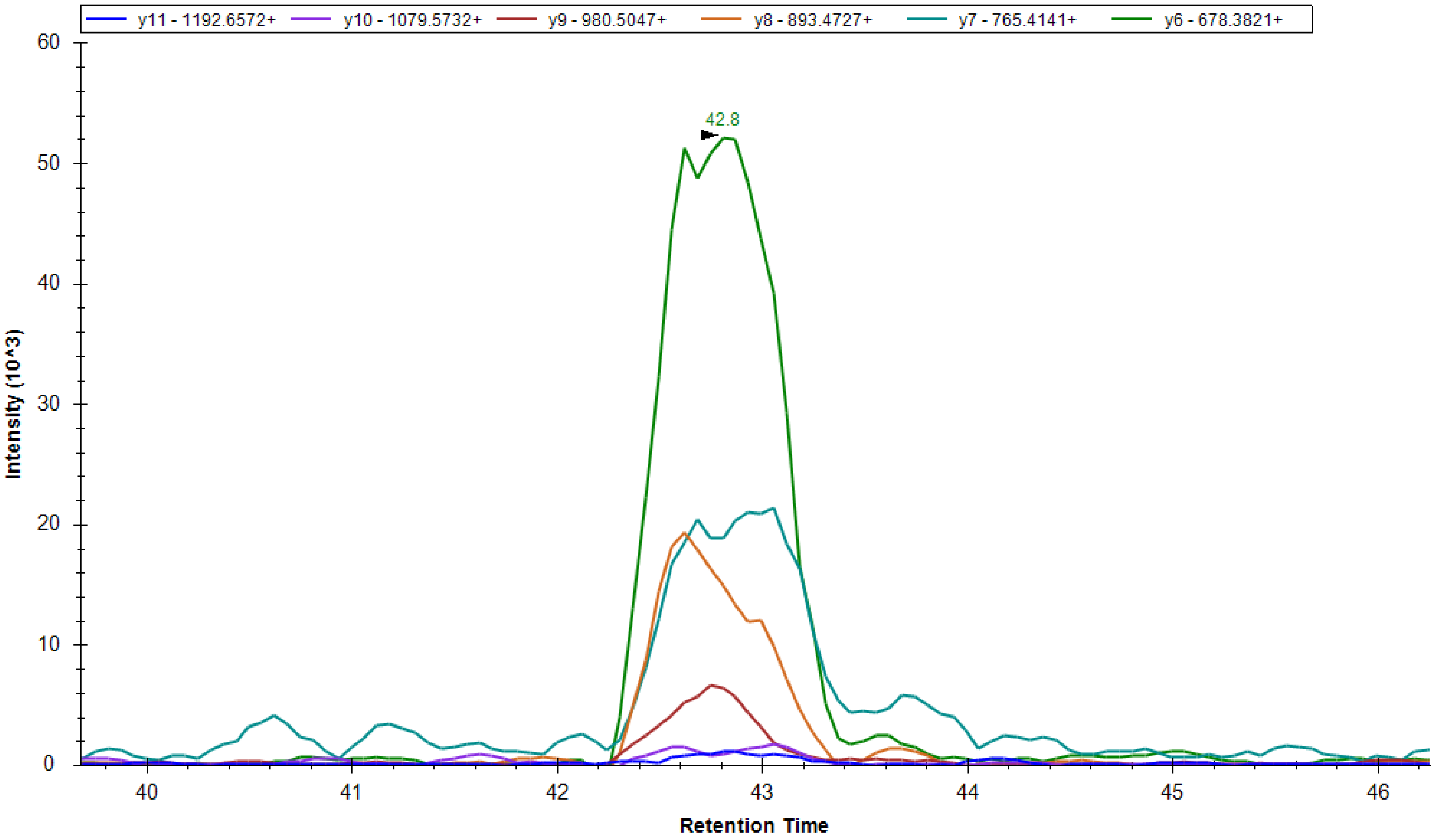

File name: 391-new#A-Round02_Negative-screen.skyd

Parent ion m/z and charges: 782.9238++

NR_033361.1.3
MPDAHLQDGAAAAAAAGGAARR

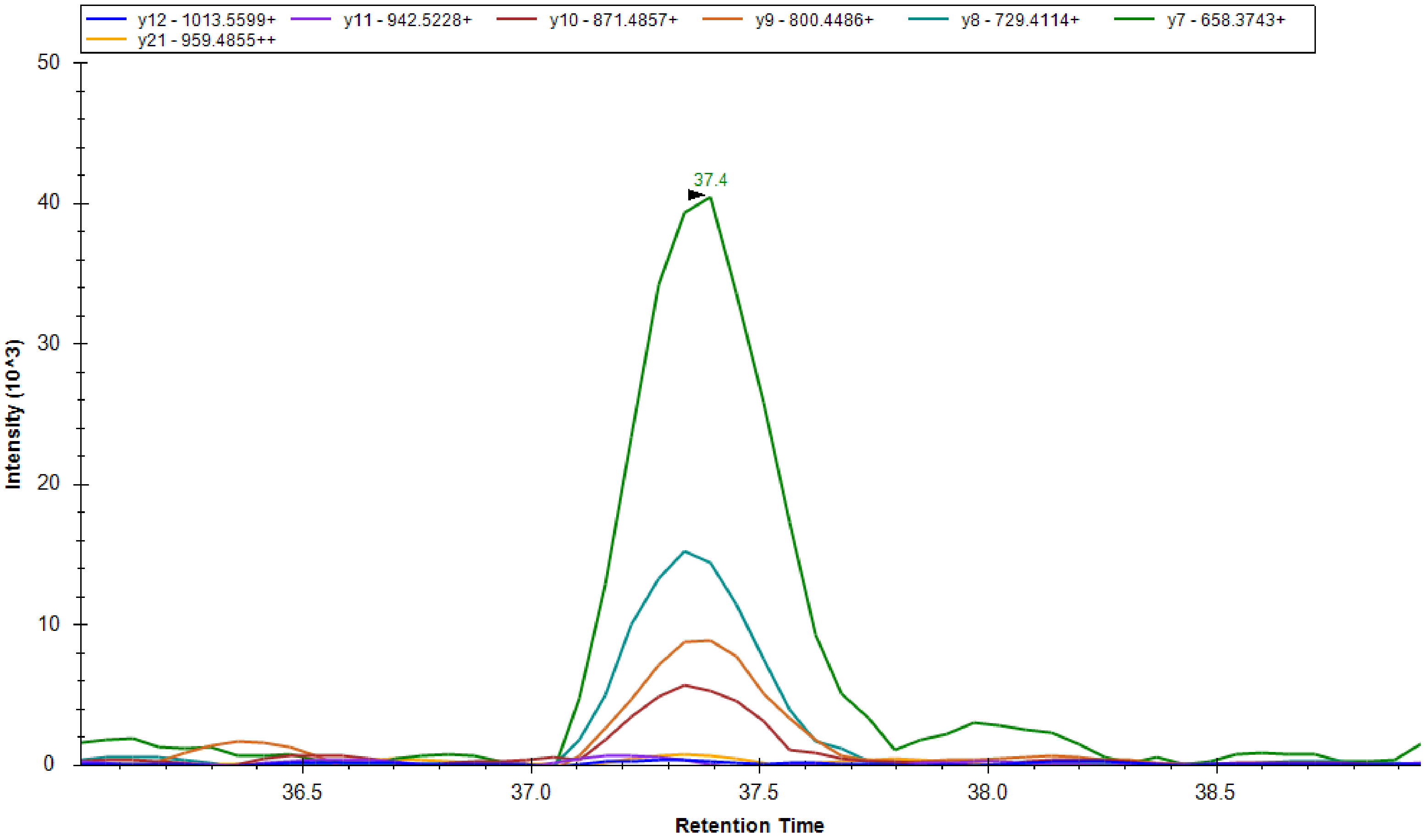

File name: 391-new#B-Round02_Negative-screen.skyd

Parent ion m/z and charges: 683.6729+++

NR_033361.1.3
MPDAHLQDGAAAAAAAGGAARR

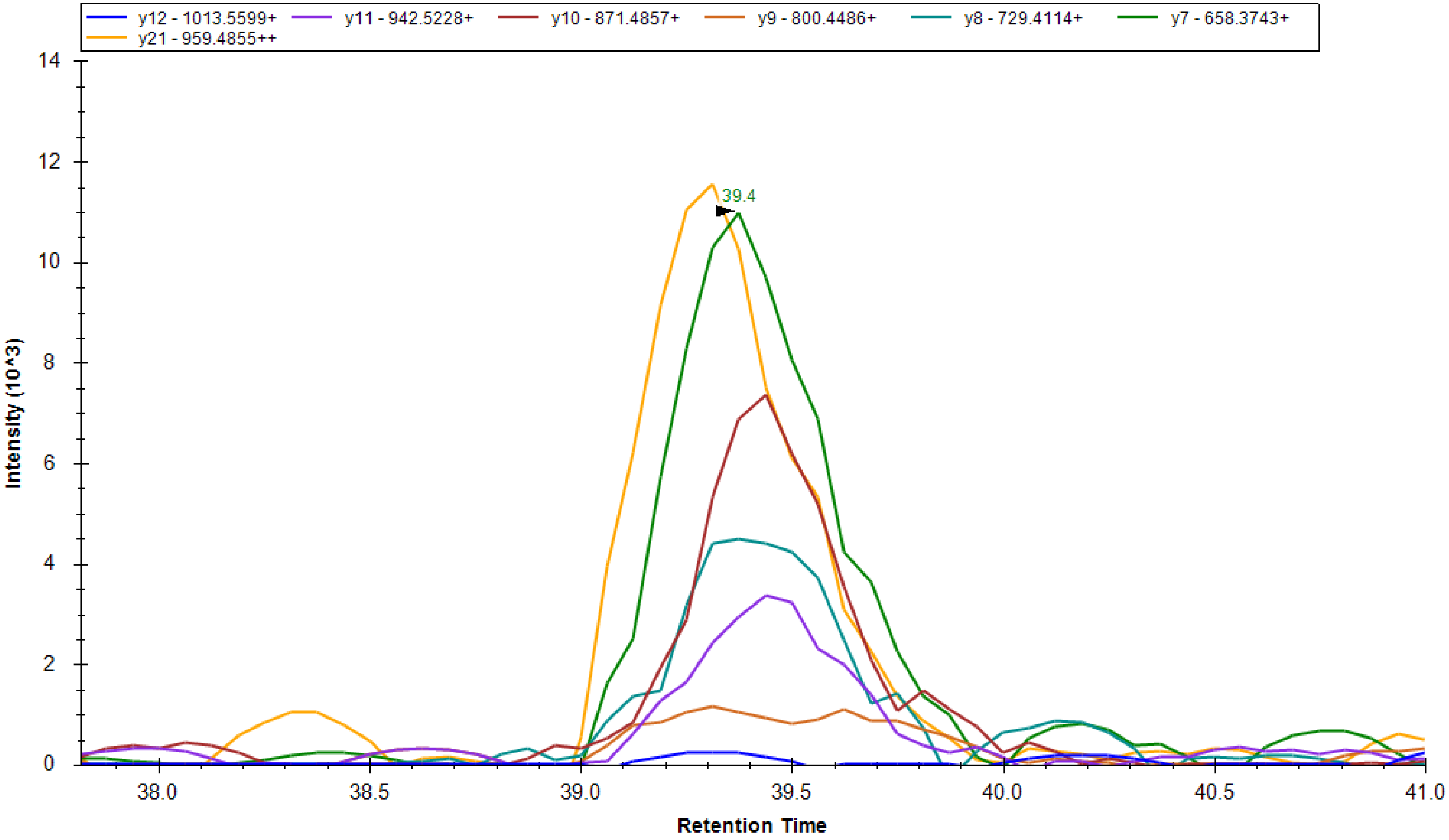

File name: 391-new#A-Round02_Negative-screen.skyd

Parent ion m/z and charges: 683.6729+++

NR_033787.1.6
PHCLLHEWSACPAAPAPHR

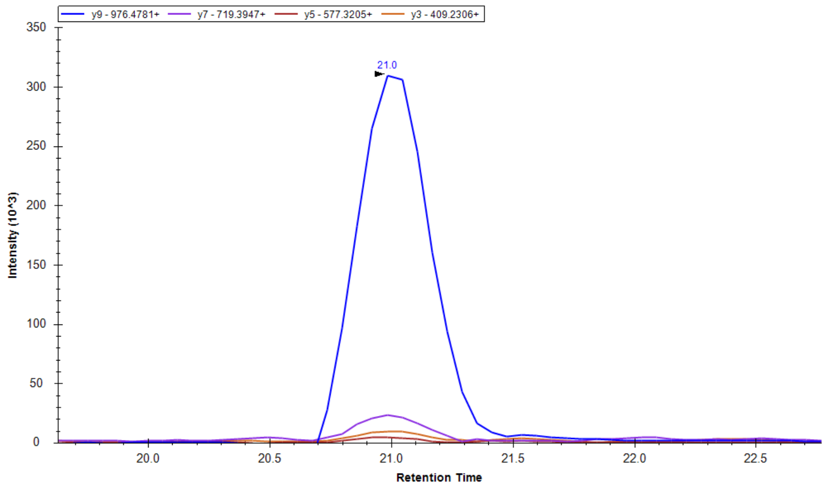

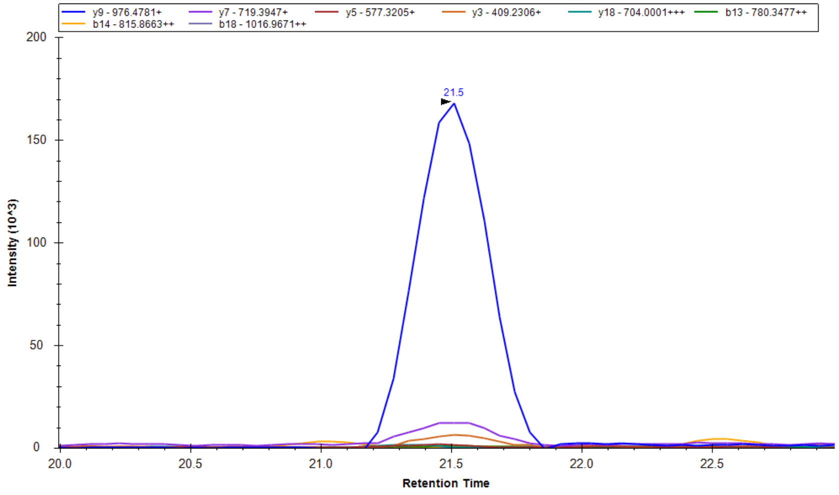

File name: 391-new#B-Round01_All-screening_Positive result.skyd

File name: 391-new#B-Round01_Positive-confirm.skyd

Parent ion m/z and charges: 736.3510+++

NR_033828.2.4
MEHTEGLSWEQGAR

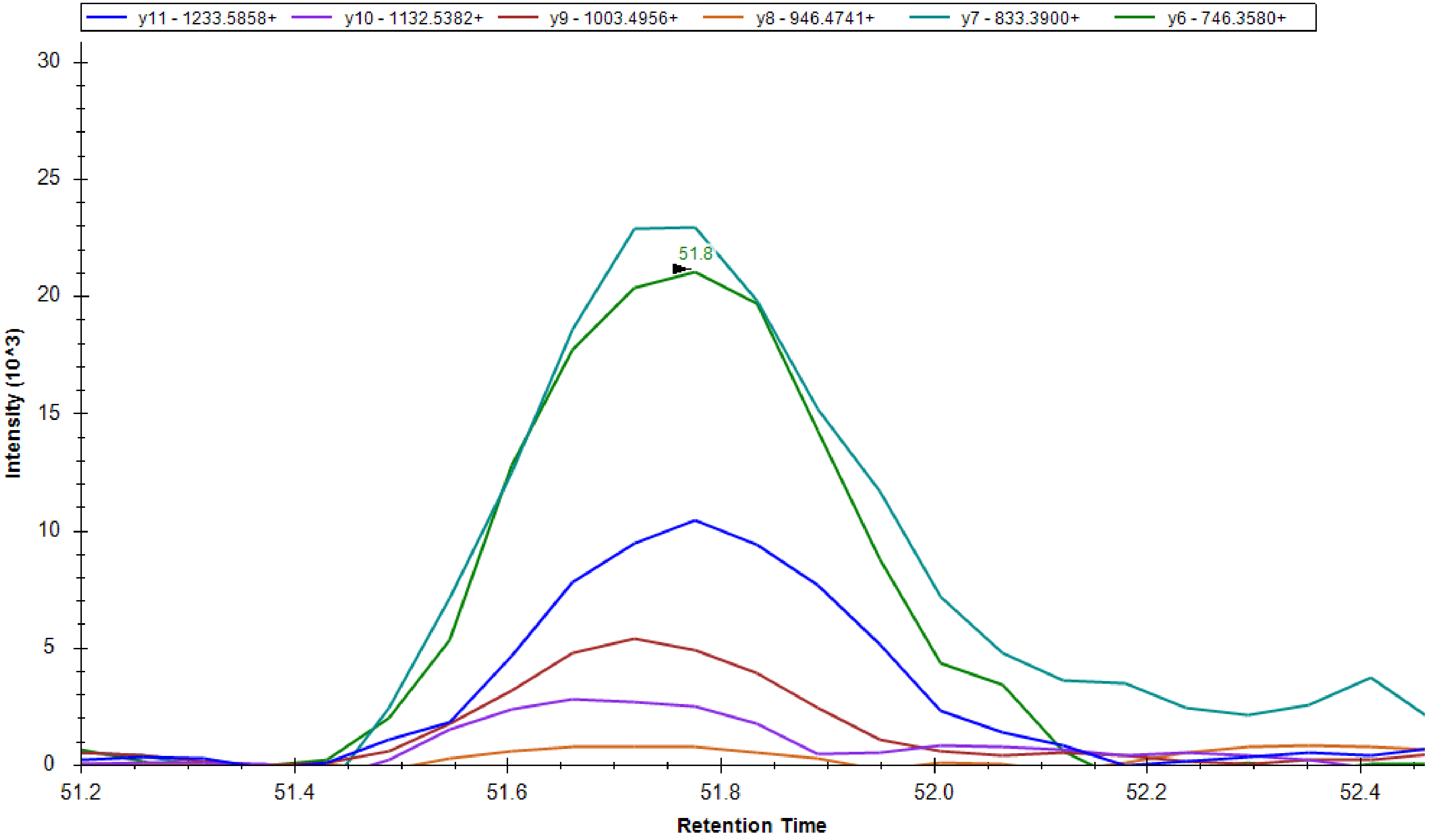

File name: 391-new#B-Round02_Negative-screen.skyd

Parent ion m/z and charges: 815.8676++

NR_033887.3.3
EFGGGGVGQAGKDQEVTFSFPAPDAFGR

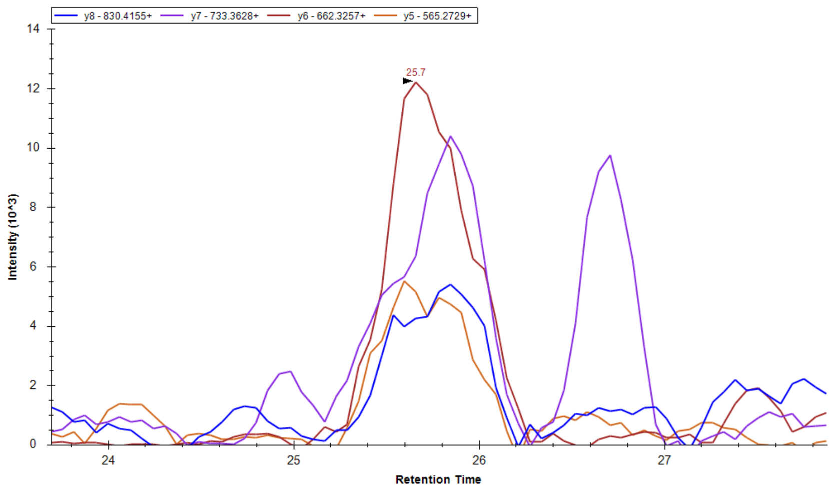

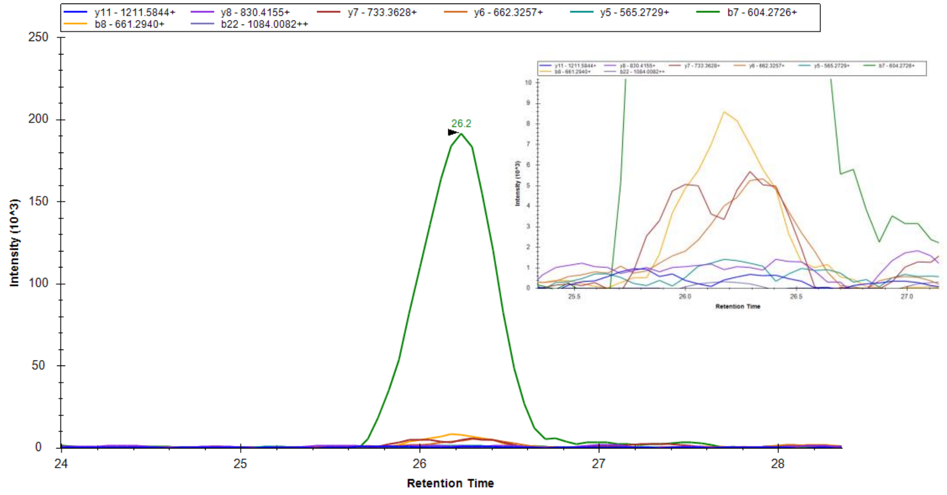

File name: 391-new#B-Round01_All-screening_Positive result.skyd

File name: 391-new#B-Round01_Positive-confirm.skyd

Parent ion m/z and charges: 943.4473+++

NR_033903.1.1
MCMALPMPVSQGR

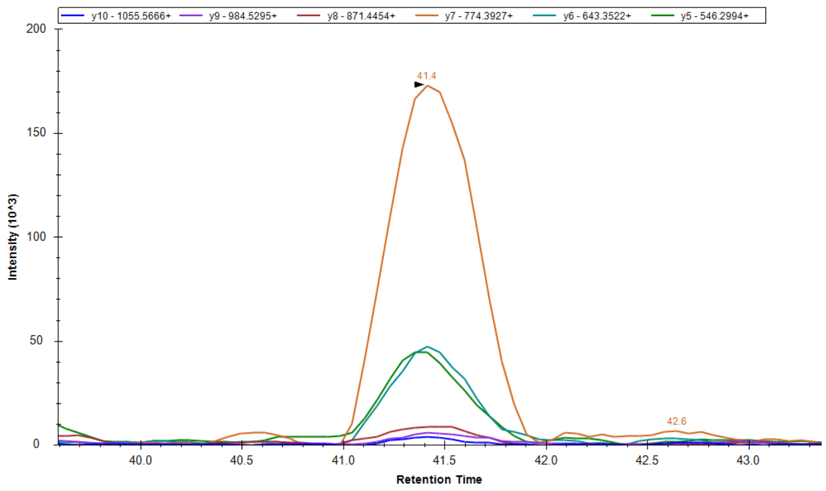

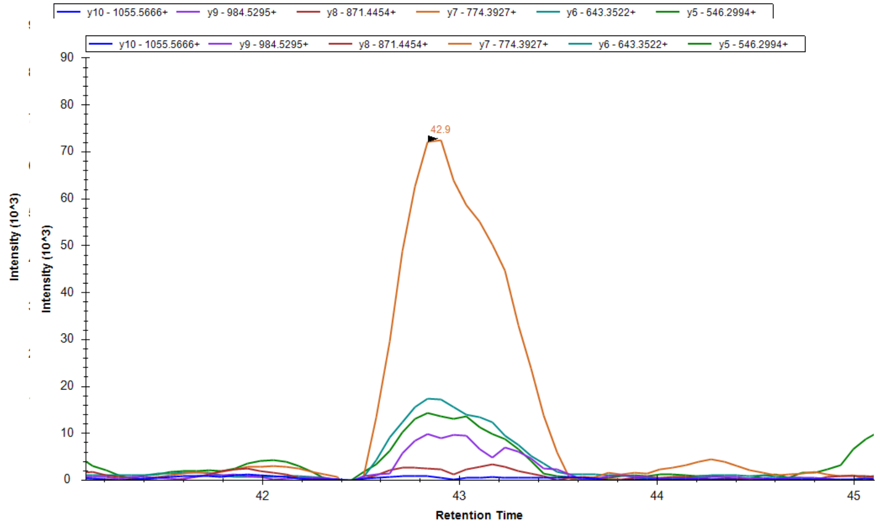

File name: 391-new#A-Round01_All-screening_Positive result.skyd

File name: 391-new#A-Round01_Positive-confirm.skyd

Parent ion m/z and charges: 739.3428++

NR_033903.1.1
MCMALPMPVSQGR

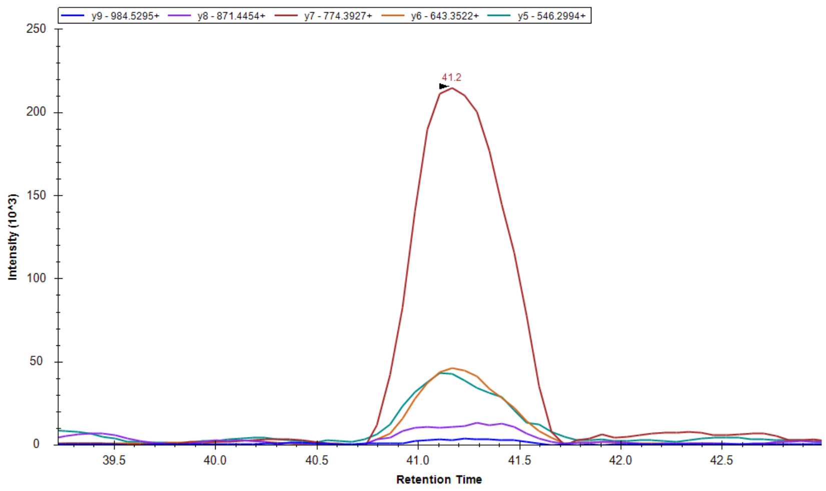

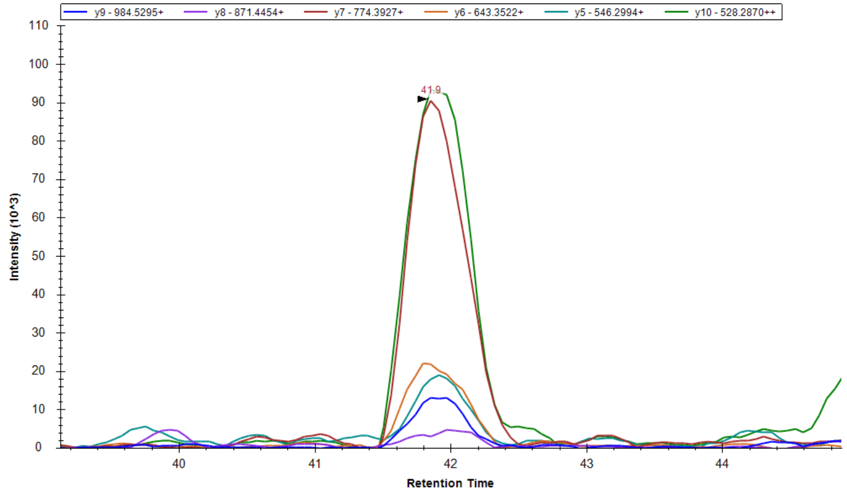

File name: 391-new#B-Round01_All-screening_Positive result.skyd

File name: 391-new#B-Round01_Positive-confirm.skyd

Parent ion m/z and charges: 739.3428++

NR_034002.2.1
MVGTKPHCAIDTR

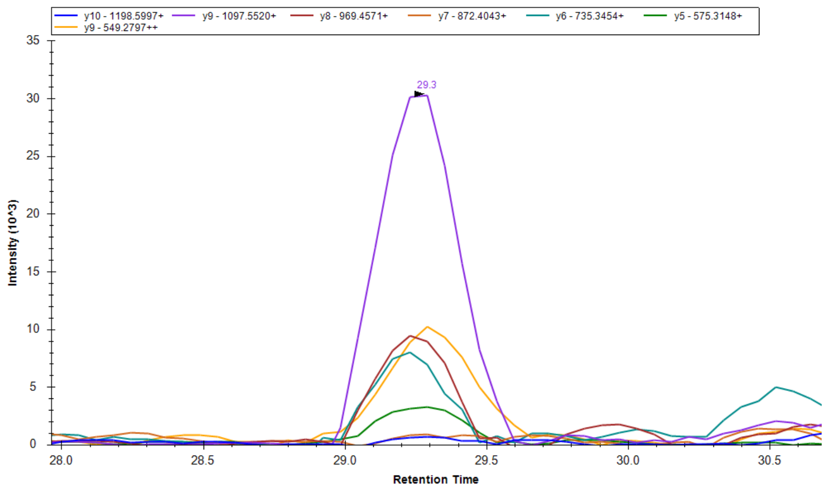

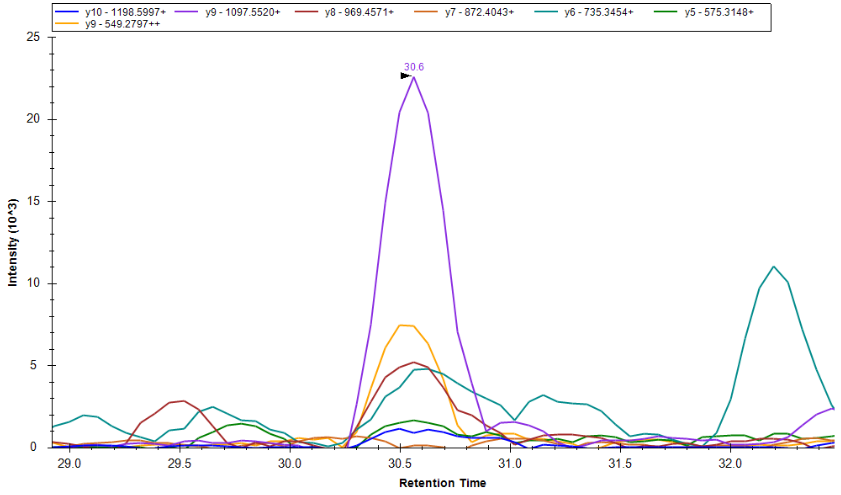

File name: 391-new#A-Round01_All-screening_Positive result.skyd

File name: 391-new#A-Round01_Positive-confirm.skyd

Parent ion m/z and charges: 743.3687++

NR_034002.2.1
MVGTKPHCAIDTR

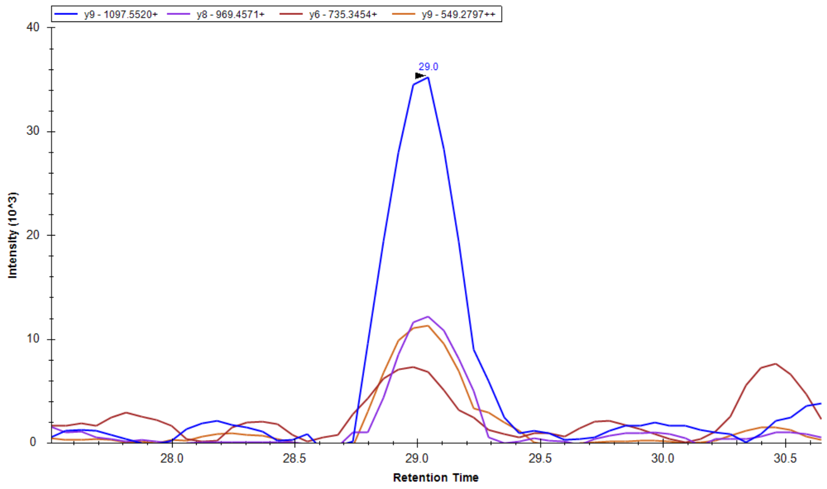

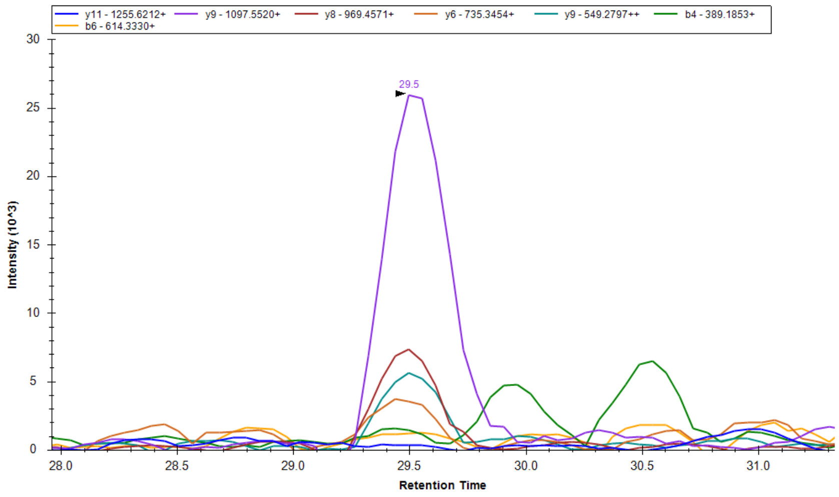

File name: 391-new#B-Round01_All-screening_Positive result.skyd

File name: 391-new#B-Round01_Positive-confirm.skyd

Parent ion m/z and charges: 743.3687++

NR_034022.2.36
MAHYHYMSMRYR

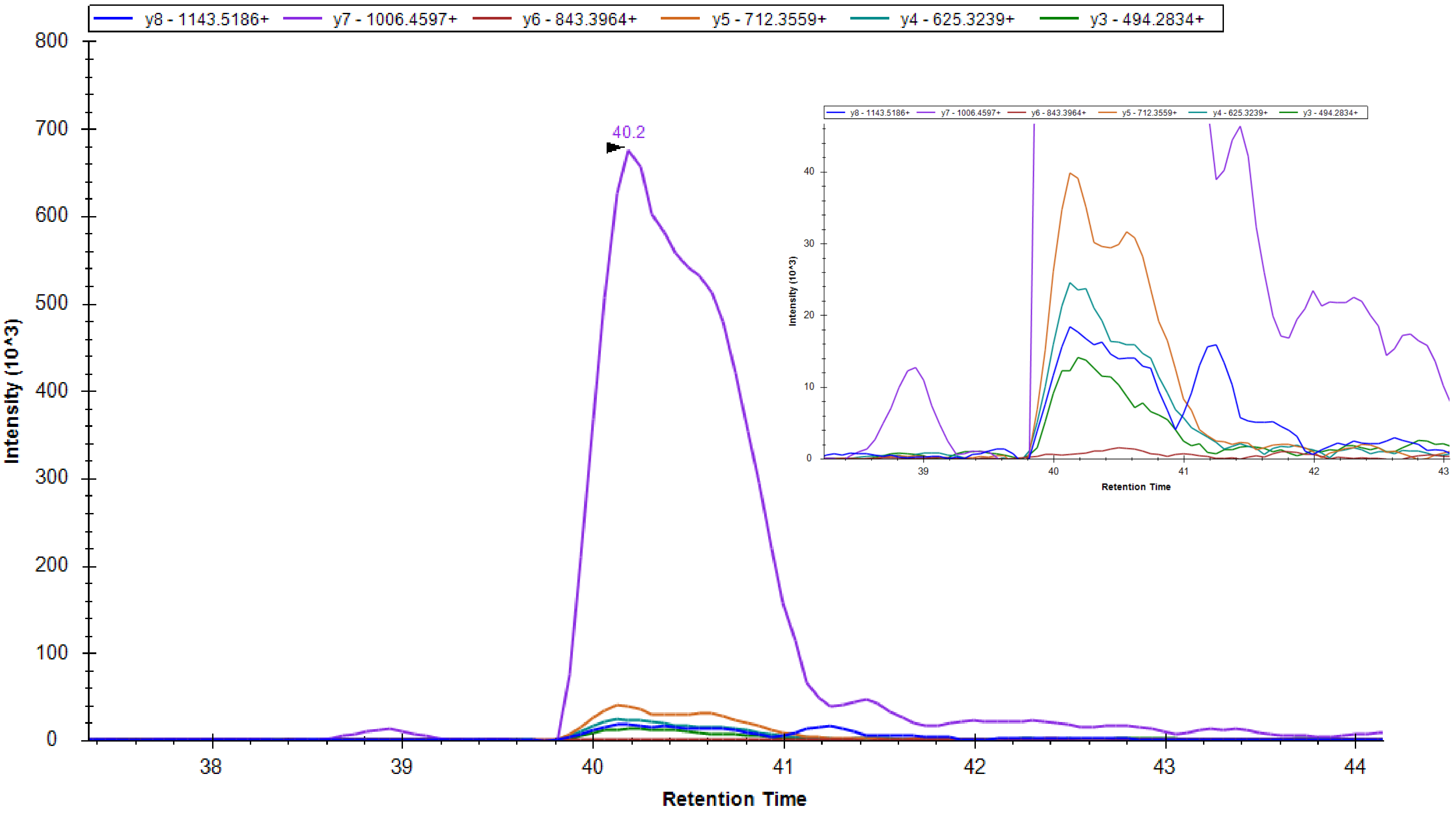

File name: 391-new#A-Round02_Negative-screen.skyd

Parent ion m/z and charges: 823.3629++

NR_034022.2.36
MAHYHYMSMRYR

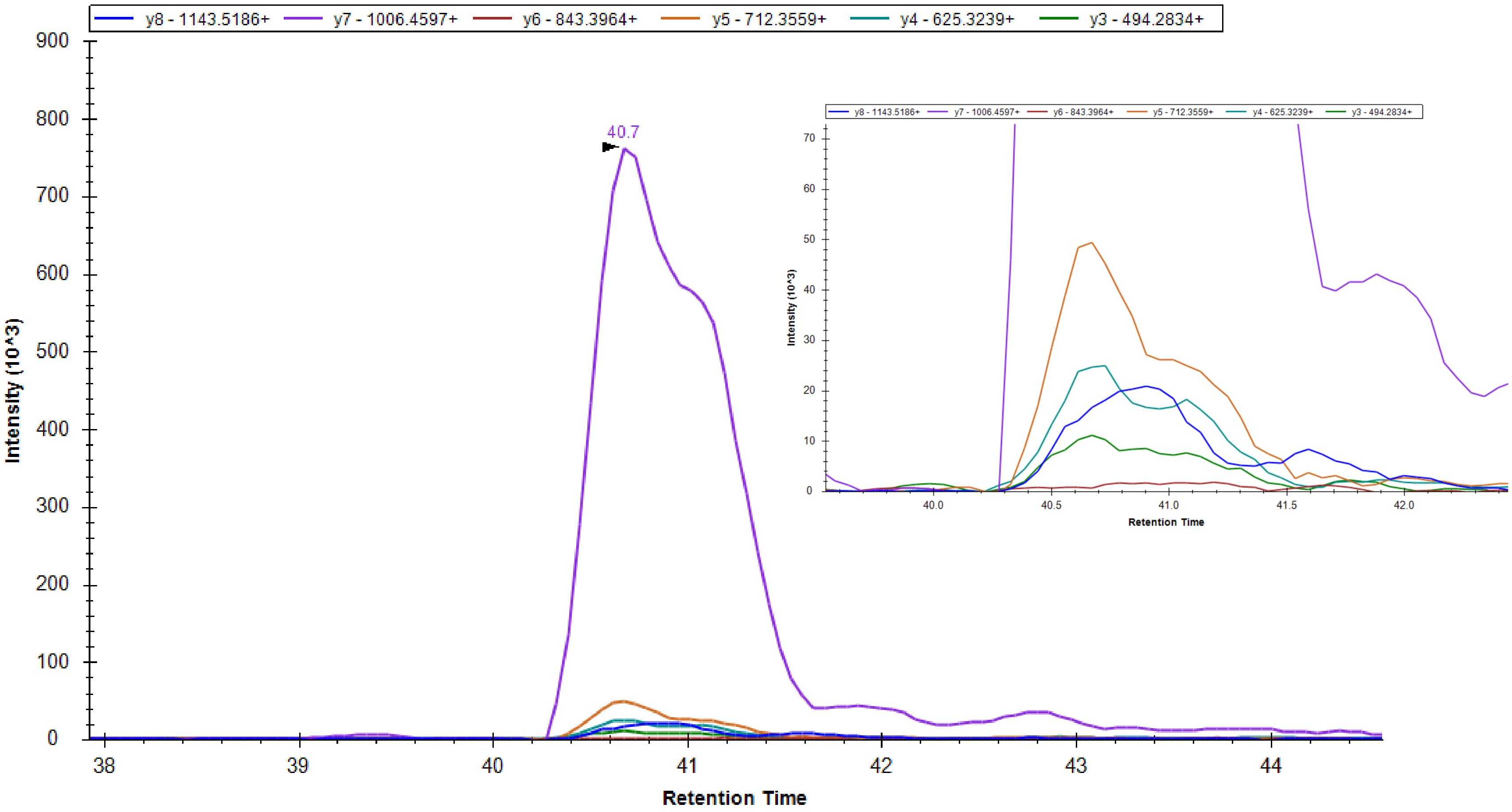

File name: 391-new#B-Round02_Negative-screen.skyd

Parent ion m/z and charges: 823.3629++

NR_034022.2.49
RTPVREHTWFNIGAFIPK

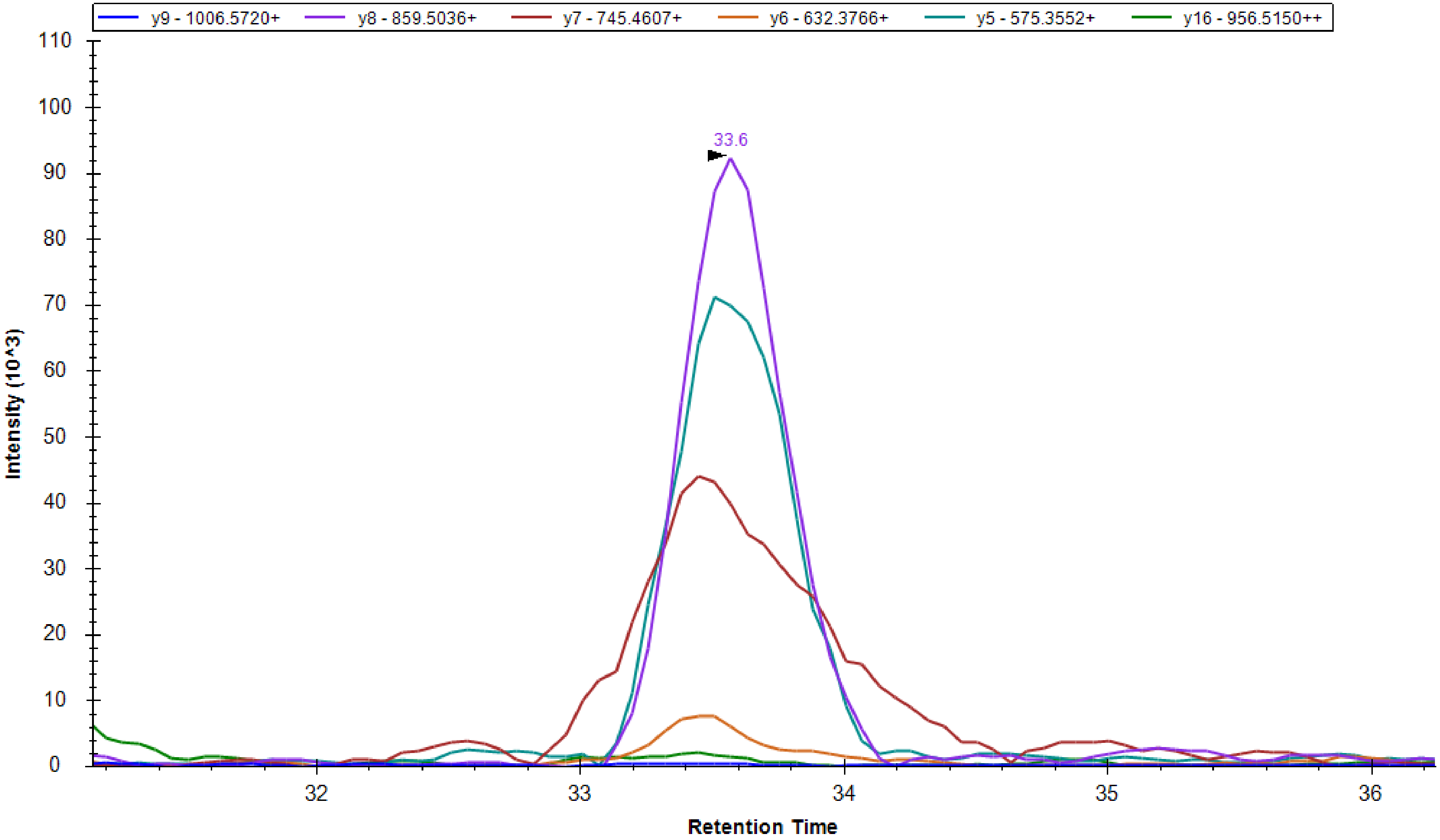

File name: 391-new#A-Round02_Negative-screen.skyd

Parent ion m/z and charges: 723.7287+++

NR_034031.1.2
MANVPTGHSPAPICK

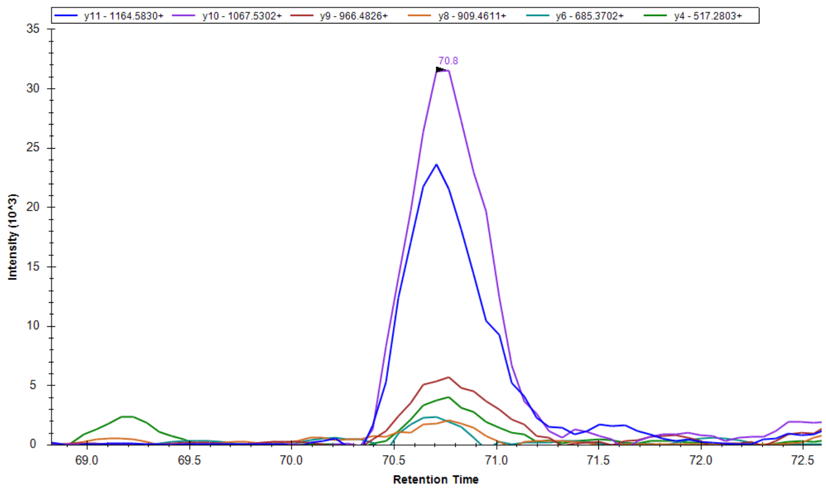

File name: 391-new#B-Round01_All-screening_Positive result.skyd

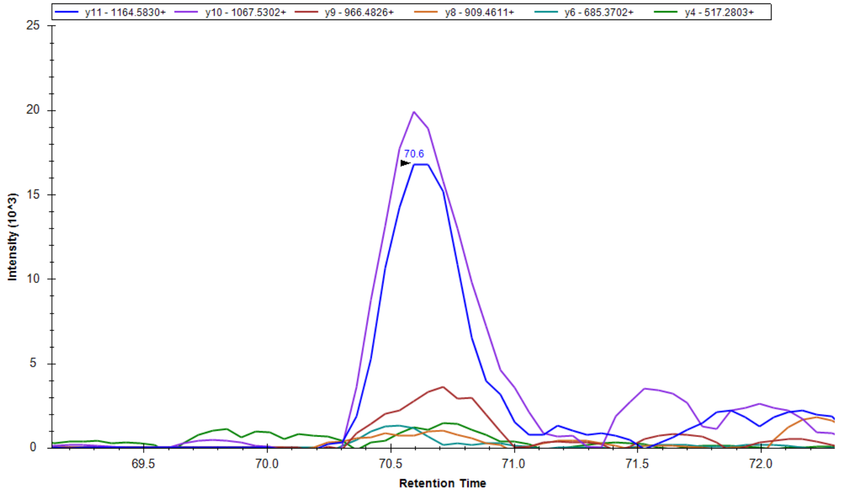

File name: 391-new#B-Round01_Positive-confirm.skyd

Parent ion m/z and charges: 790.3896++

NR_034084.1.1
KPSVPETEADGFVIIGDTTDEQR

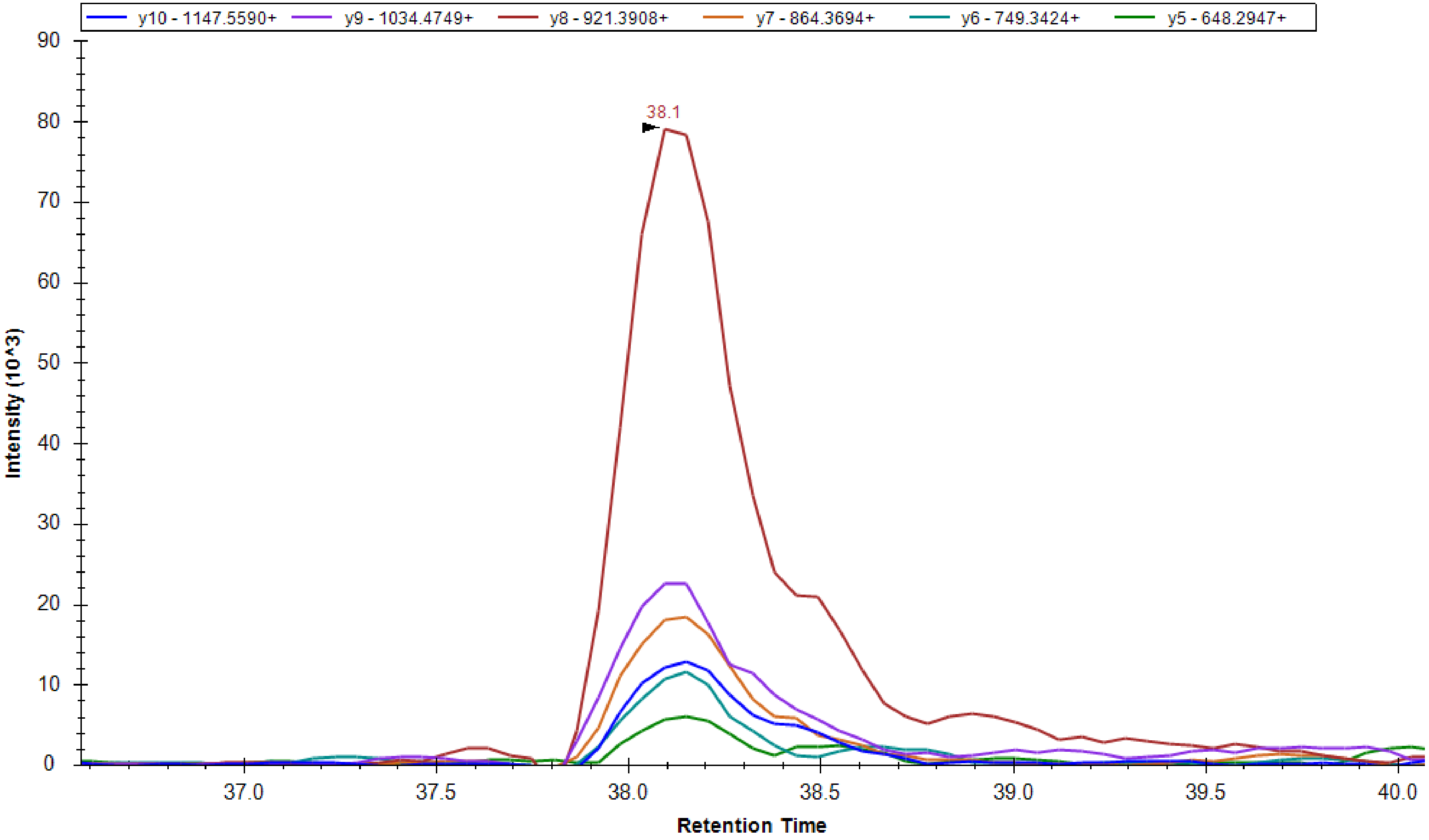

File name: 391-new#B-Round02_Negative-screen.skyd

Parent ion m/z and charges: 835.4099+++

NR_034113.2.7
MHTARRTMLSAEPLGDSR

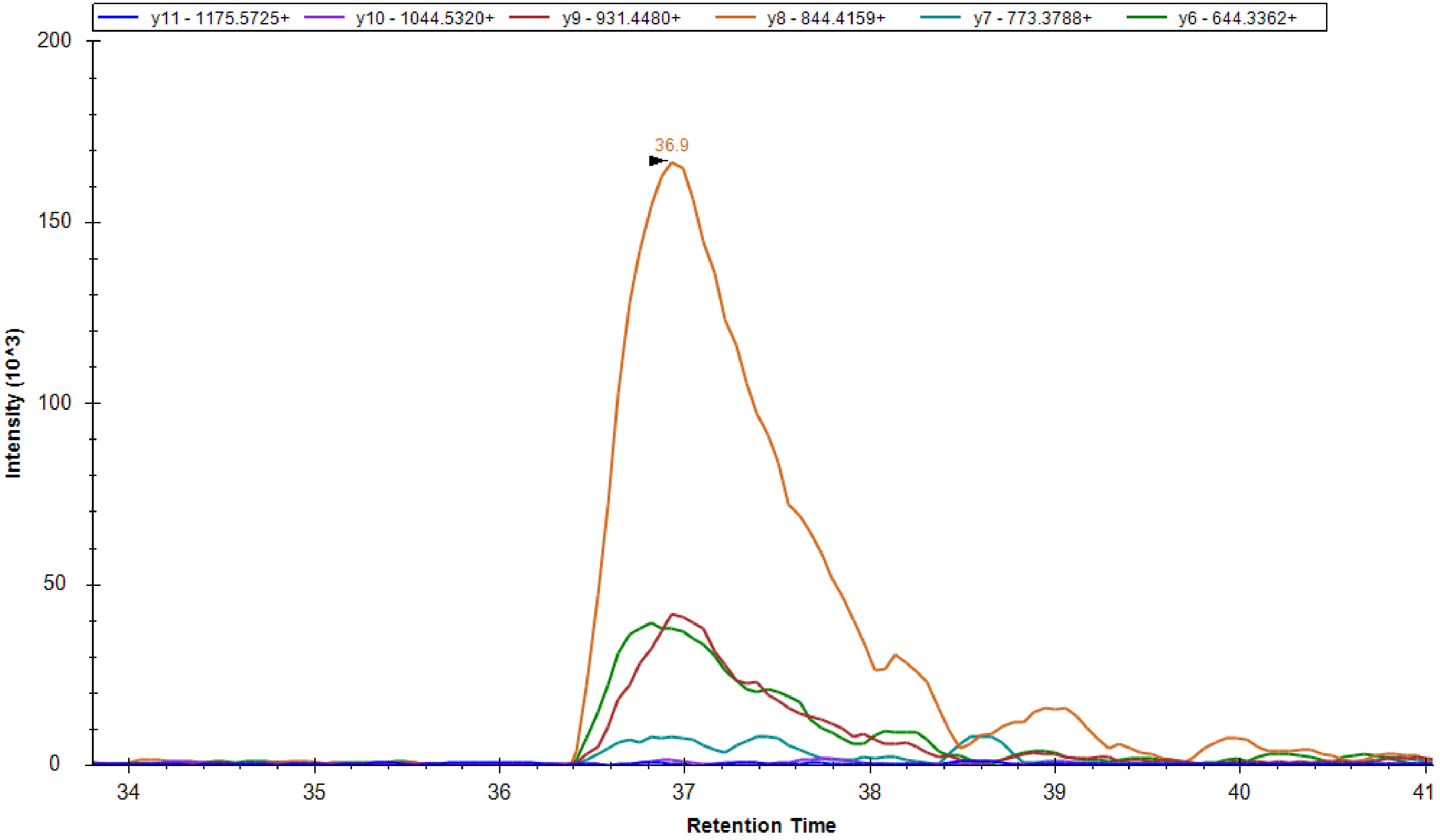

File name: 391-new#B-Round02_Negative-screen.skyd

Parent ion m/z and charges: 677.0070+++

NR_034114.3.2
MGSAGESPGLALRLSSQR

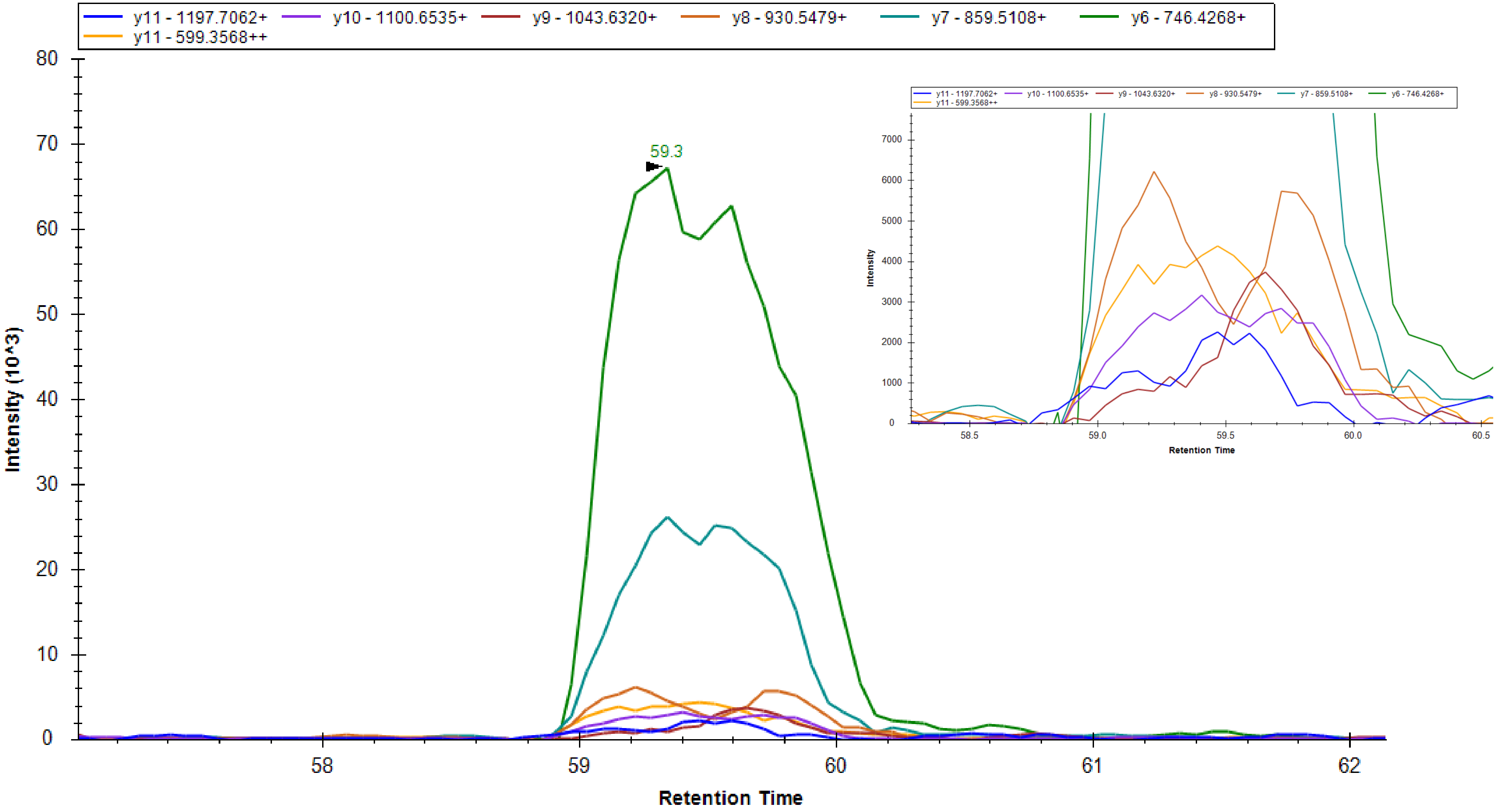

File name: 391-new#A-Round02_Negative-screen.skyd

Parent ion m/z and charges: 908.9703++

NR_034114.3.2
MGSAGESPGLALRLSSQR

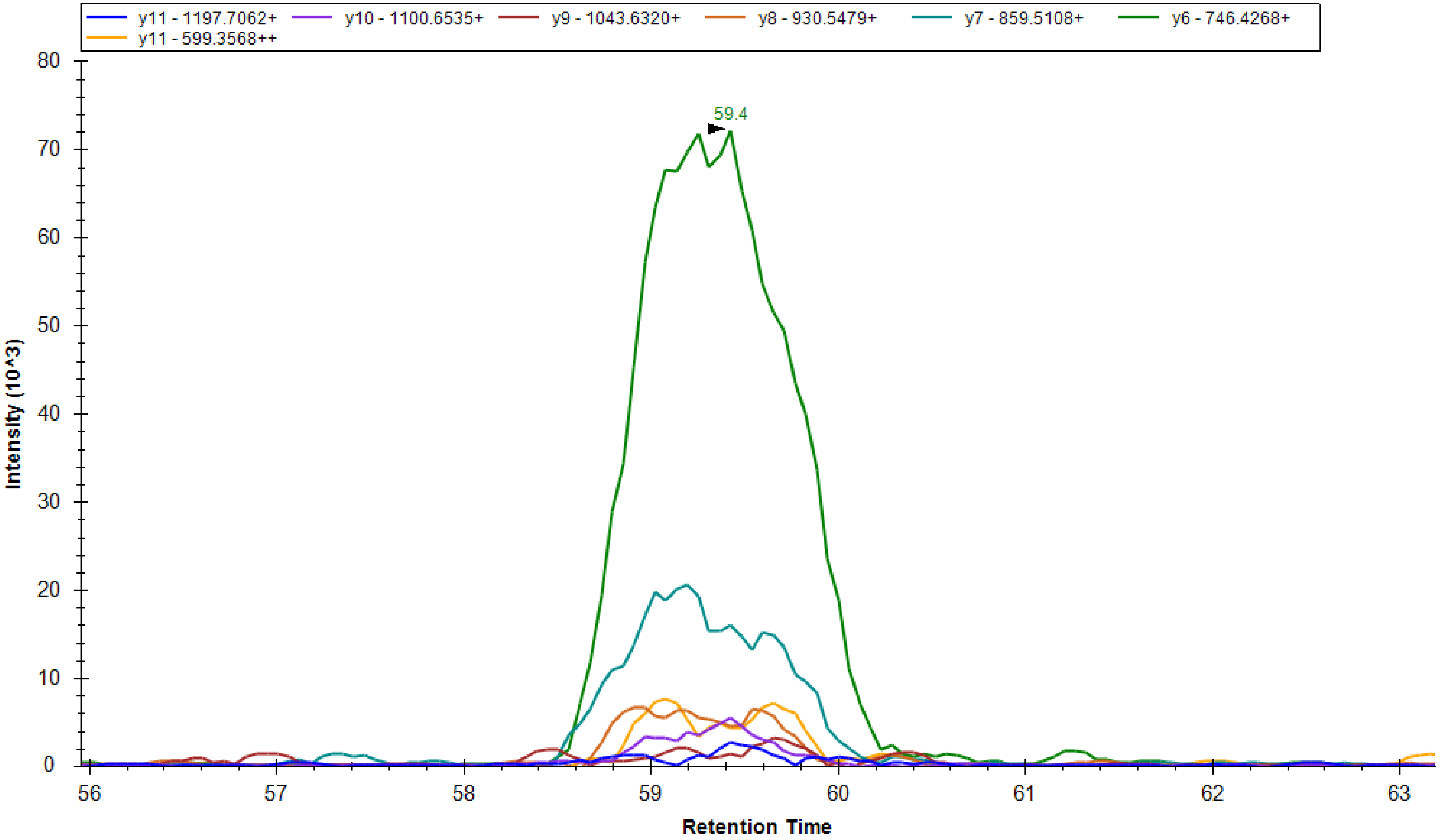

File name: 391-new#B-Round02_Negative-screen.skyd

Parent ion m/z and charges: 908.9703++

NR_034140.3.1
VAAQSAALGSQGTGDARHR

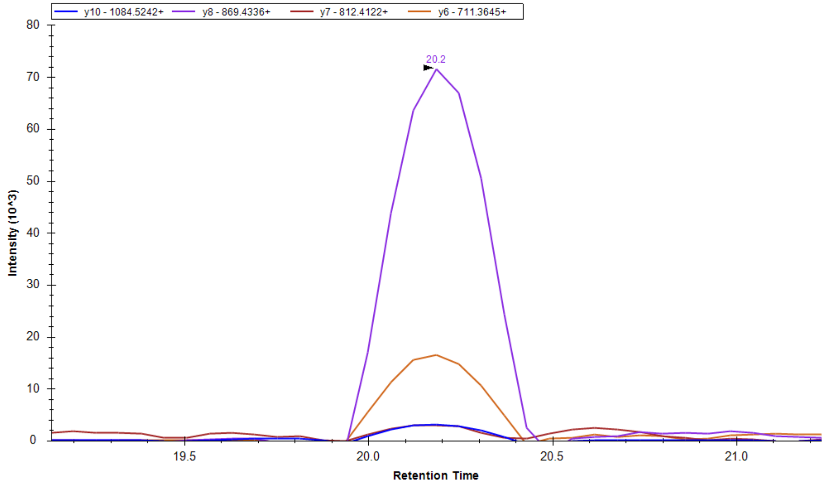

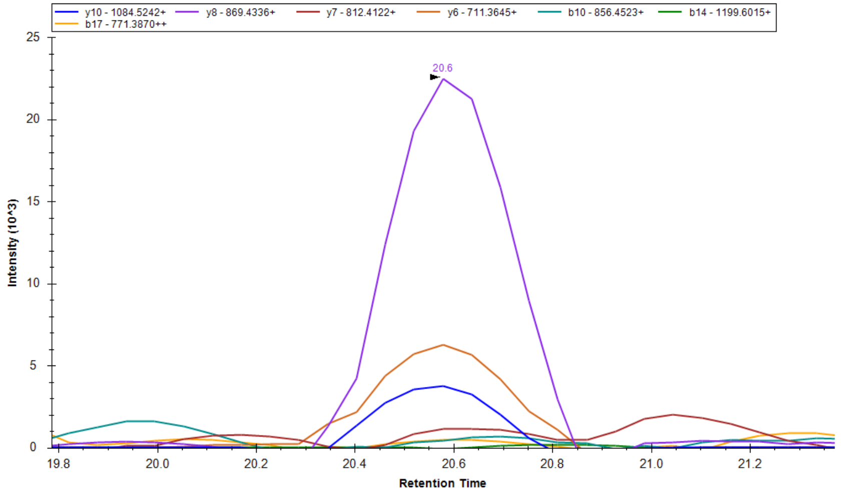

File name: 391-new#B-Round01_All-screening_Positive result.skyd

File name: 391-new#B-Round01_Positive-confirm.skyd

Parent ion m/z and charges: 618.3173+++

NR_036447.3.5
MPAGAAPSPSVGSGAASSTR

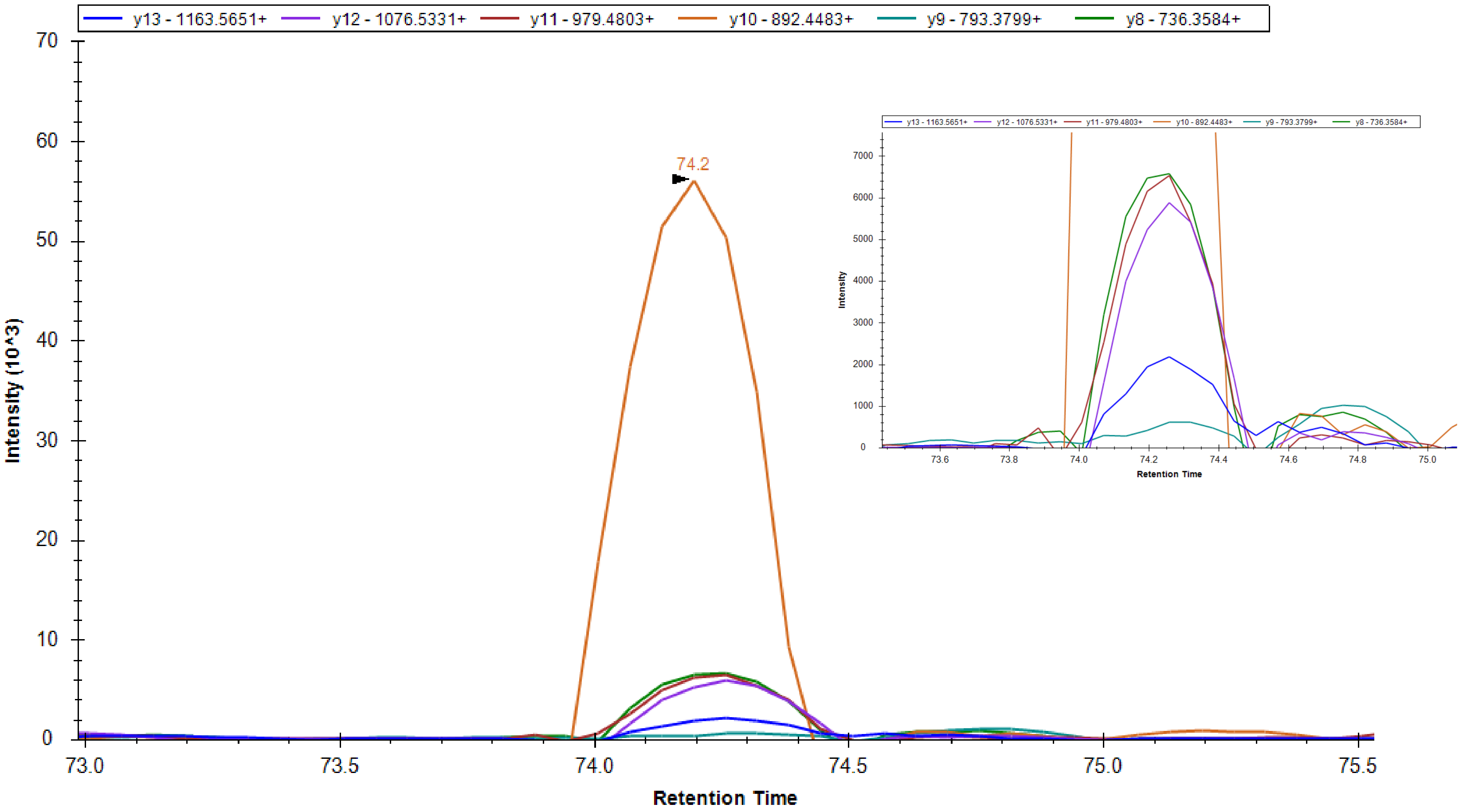

File name: 391-new#A-Round02_Negative-screen.skyd

Parent ion m/z and charges: 879.9256++

NR_036448.3.1
RQASWSCKSSSSSSQL

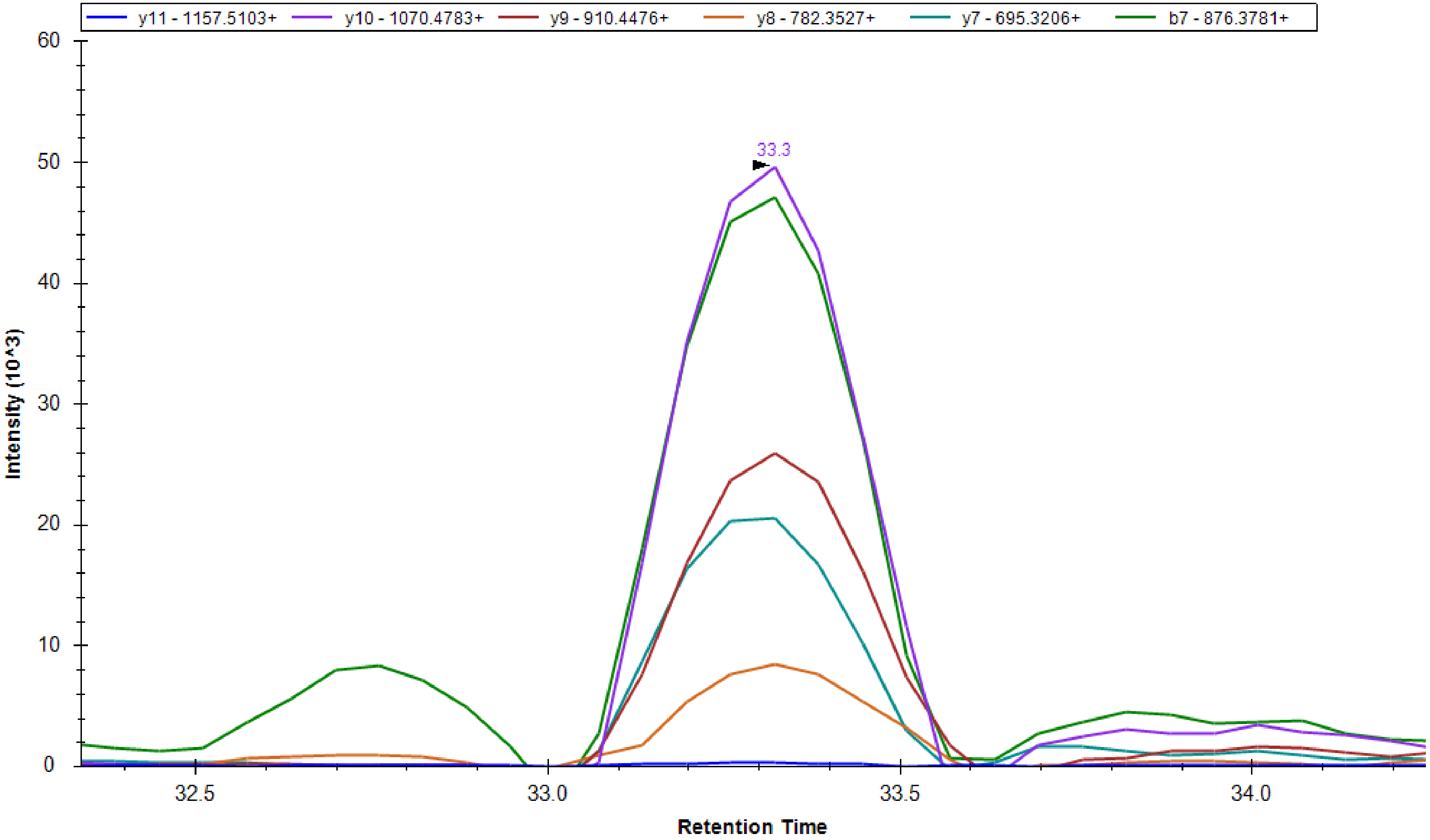

File name: 391-new#A-Round02_Negative-screen.skyd

Parent ion m/z and charges: 893.4129++

NR_036521.3.1
FLLANSGDSPAR

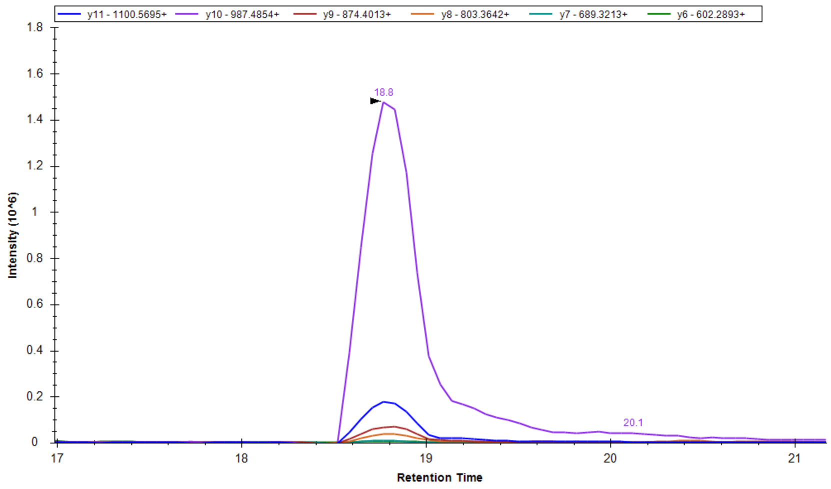

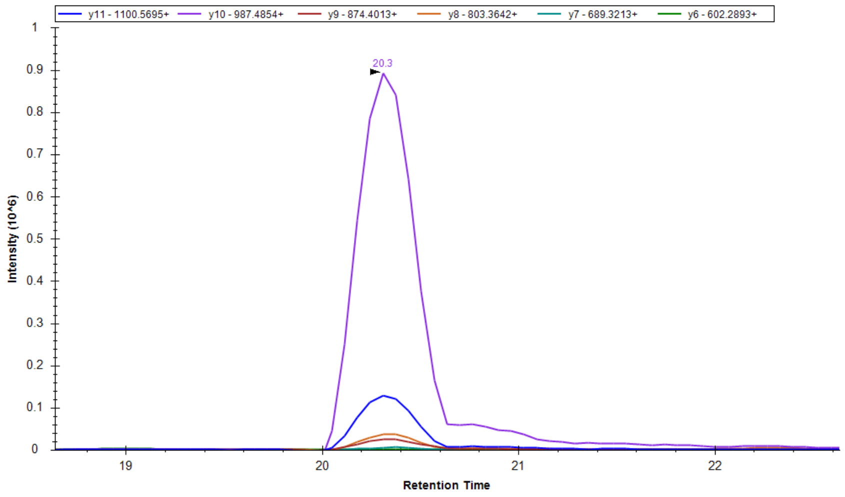

File name: 391-new#A-Round01_All-screening_Positive result.skyd

File name: 391-new#A-Round01_Positive-confirm.skyd

Parent ion m/z and charges: 624.3226++

NR_036521.3.1
FLLANSGDSPAR

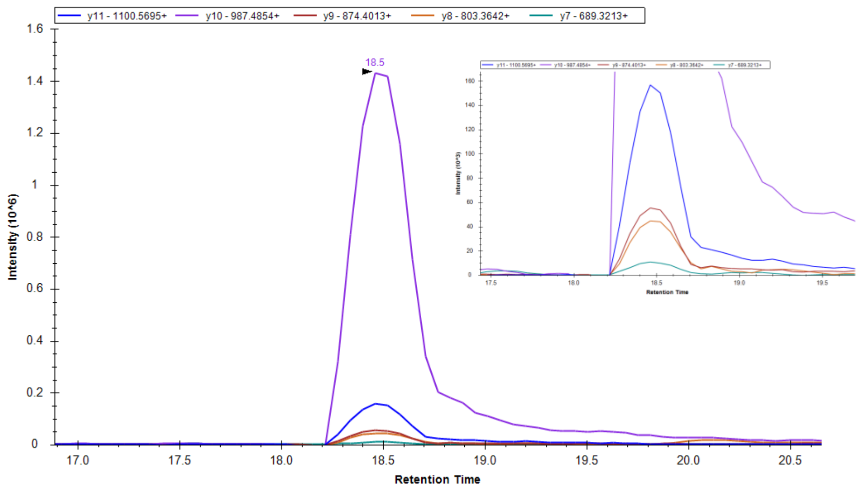

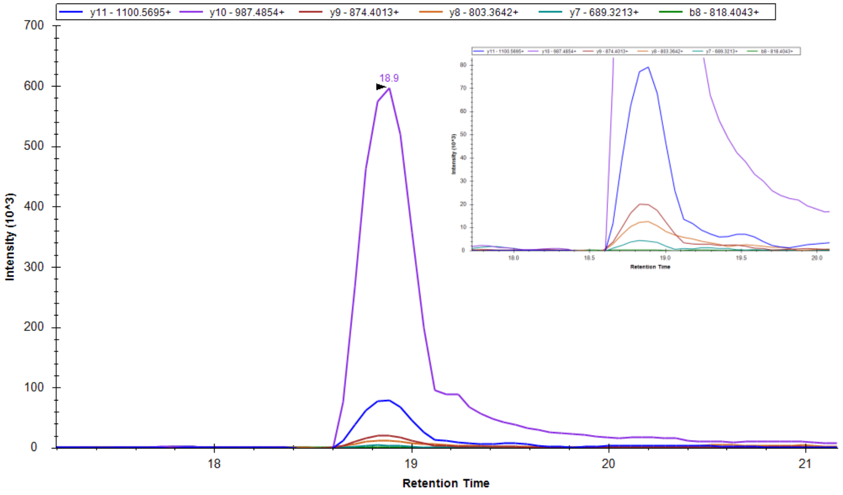

File name: 391-new#B-Round01_All-screening_Positive result.skyd

File name: 391-new#B-Round01_Positive-confirm.skyd

Parent ion m/z and charges: 624.3226++

NR_037163.3.18
MWYLHLSPVKDK

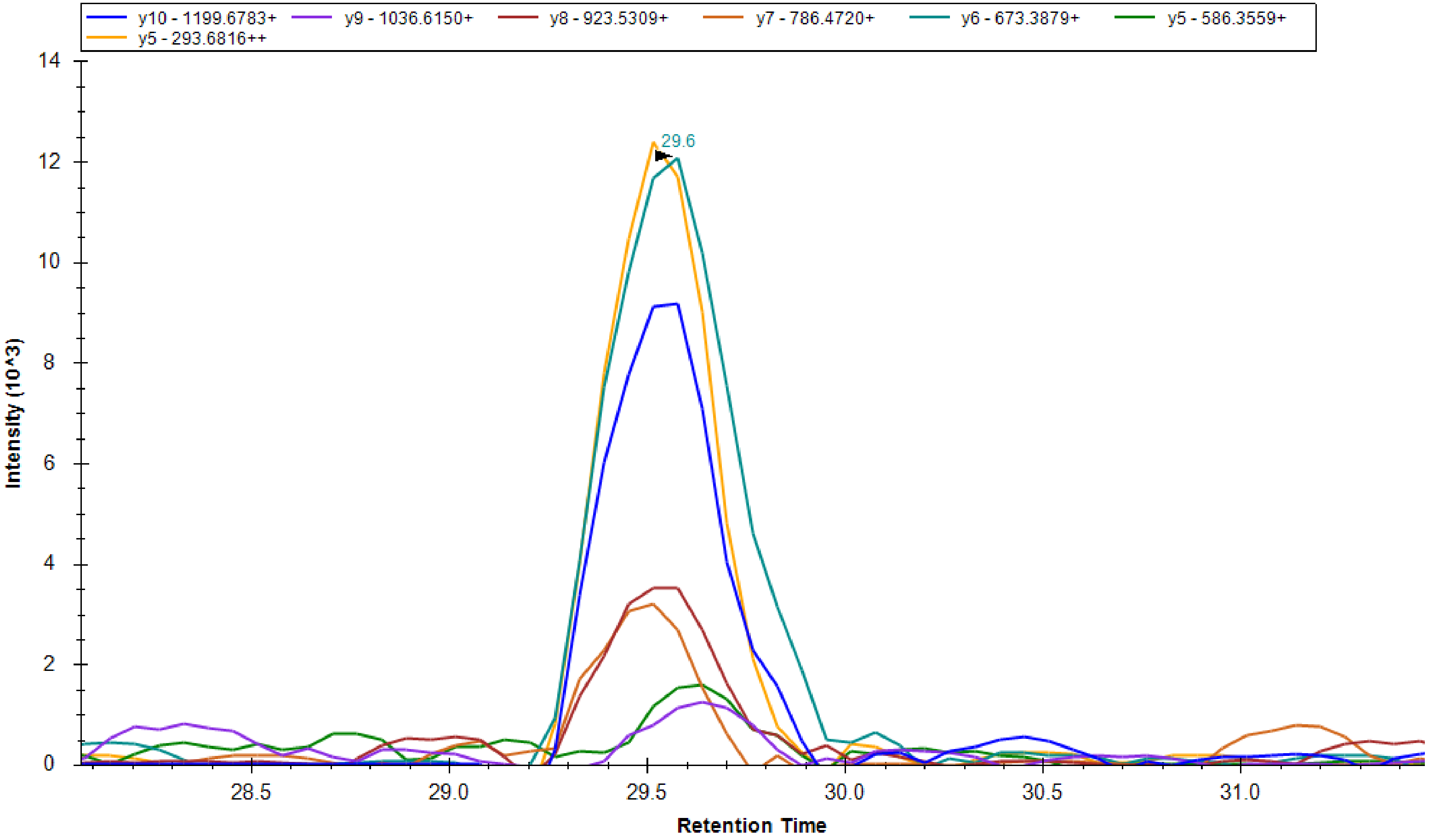

File name: 391-new#A-Round02_Negative-screen.skyd

Parent ion m/z and charges: 758.9027++

NR_037163.3.18
MWYLHLSPVKDK

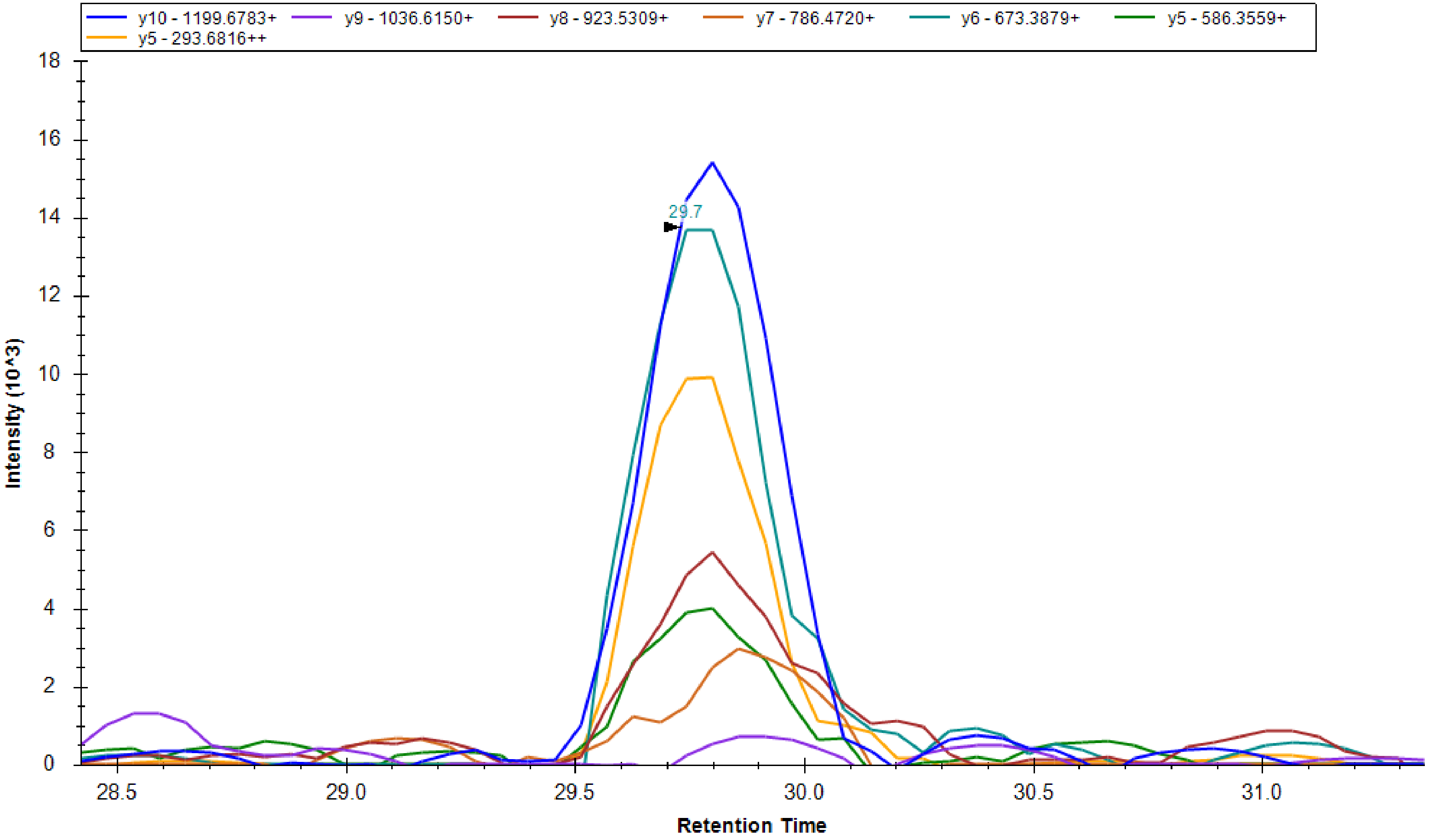

File name: 391-new#B-Round02_Negative-screen.skyd

Parent ion m/z and charges: 758.9027++

NR_037607.3.1
EGSLTGSDSHSNSNSPTAAGDSR

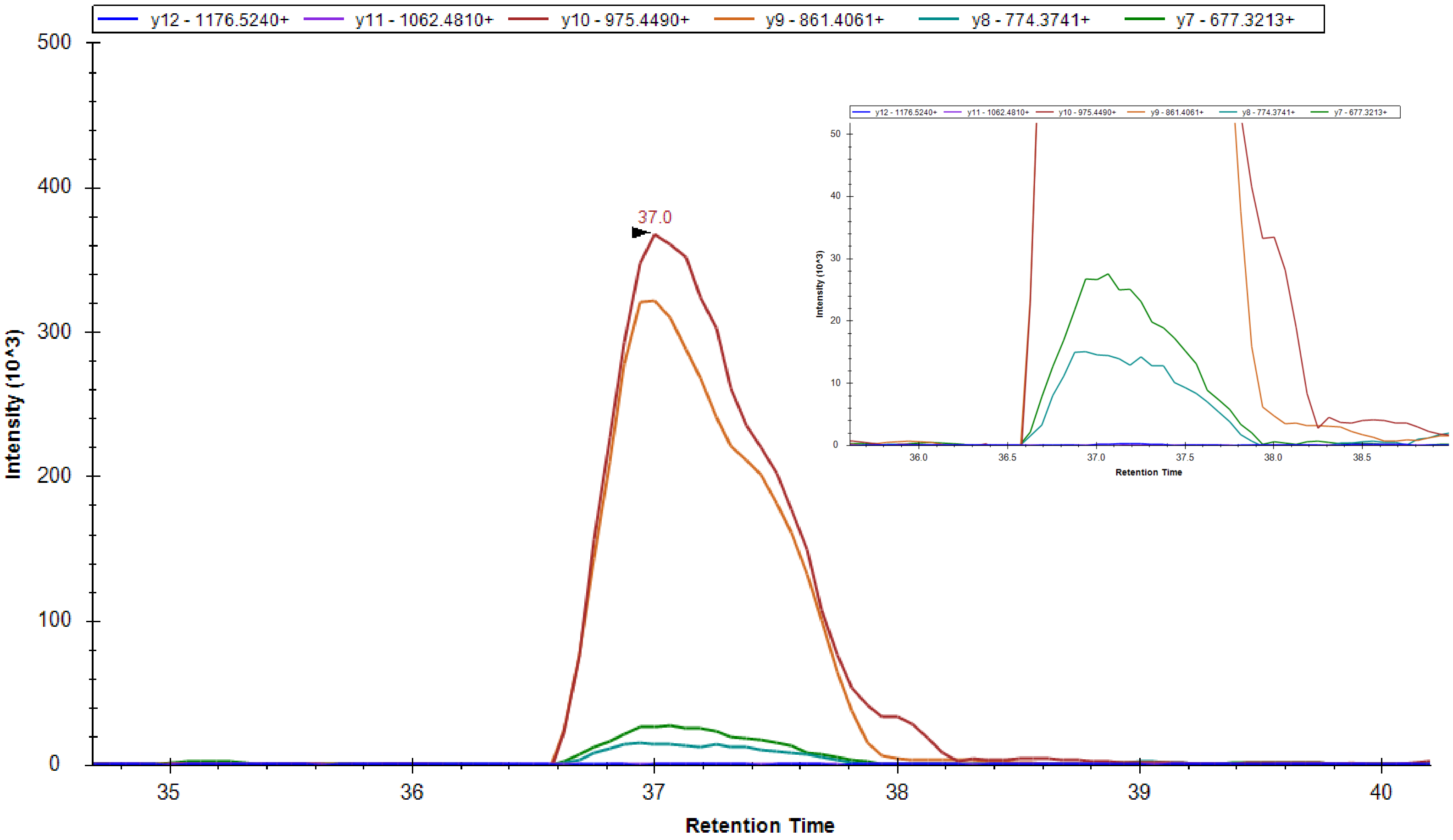

File name: 391-new#A-Round02_Negative-screen.skyd

Parent ion m/z and charges: 745.3233+++

NR_037644.1.2
MTLAALRDAEIQK

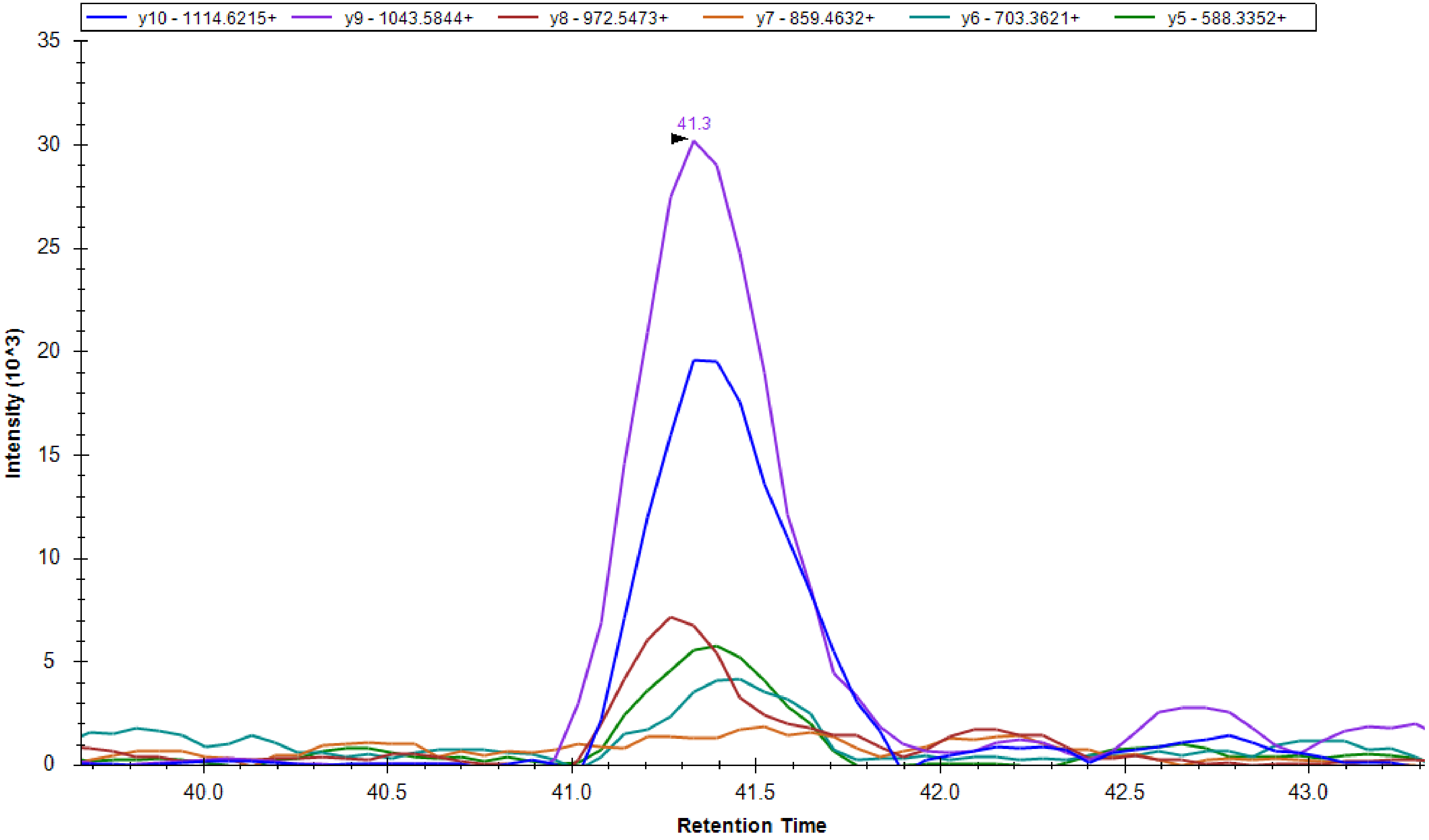

File name: 391-new#A-Round02_Negative-screen.skyd

Parent ion m/z and charges: 730.4005++

NR_037709.1.1
MCTQSLGLAGLGSDPGGK

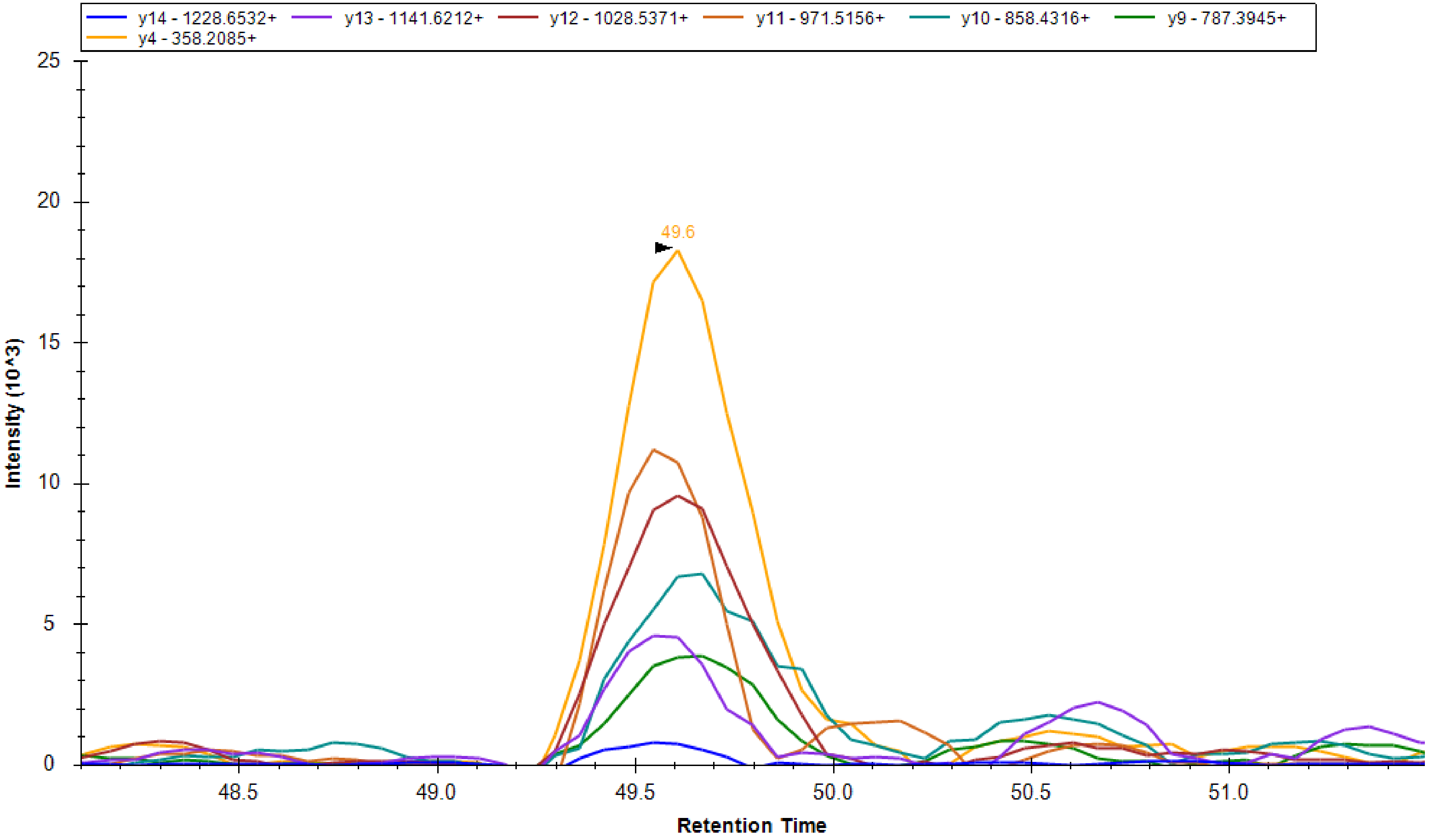

File name: 391-new#A-Round02_Negative-screen.skyd

Parent ion m/z and charges: 874.9189++

NR_037847.1.3
MLGPRNQGGK

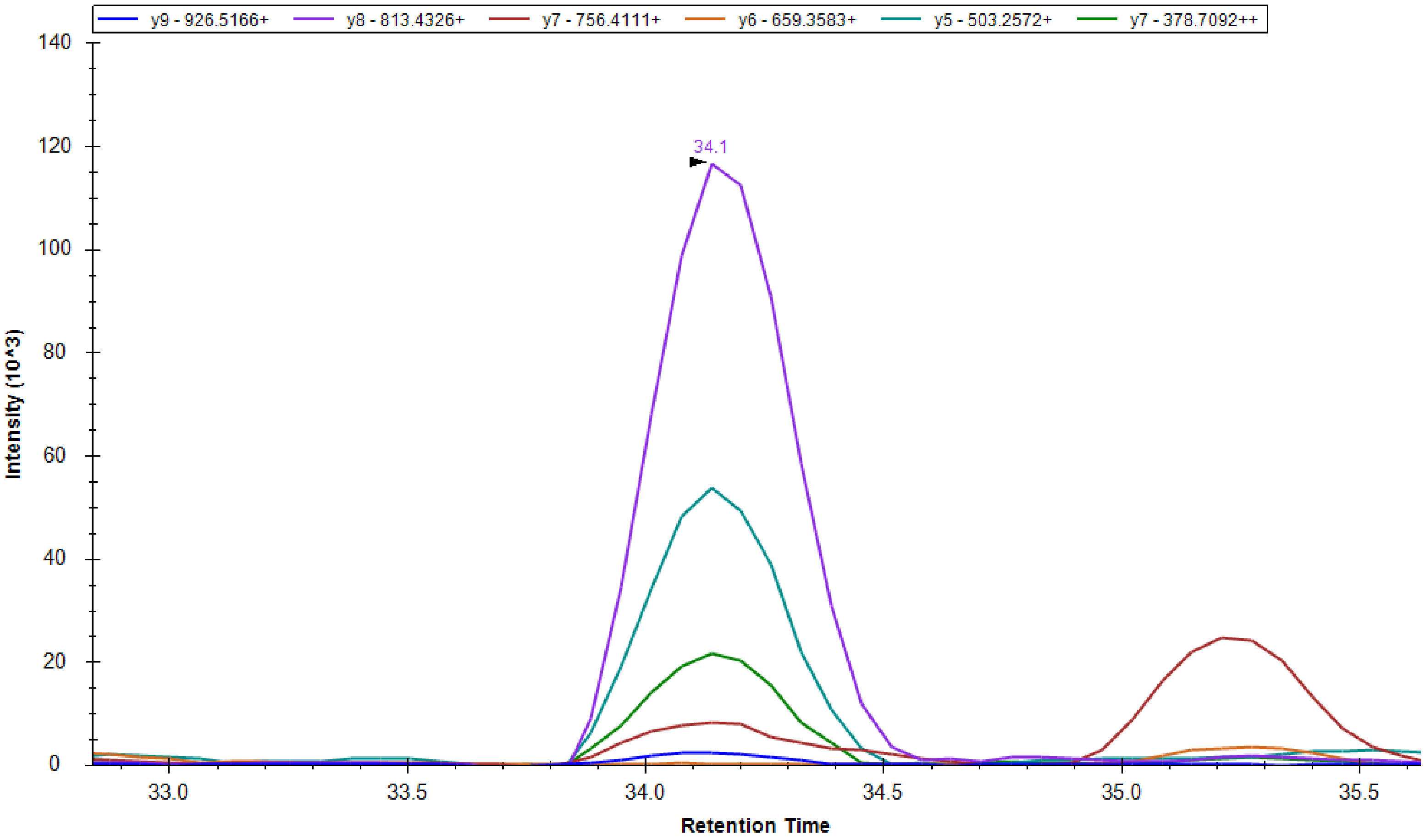

File name: 391-new#A-Round02_Negative-screen.skyd

Parent ion m/z and charges: 529.2822++

NR_038354.2.1
WAGSCTSISTRGSARQSHEVGR

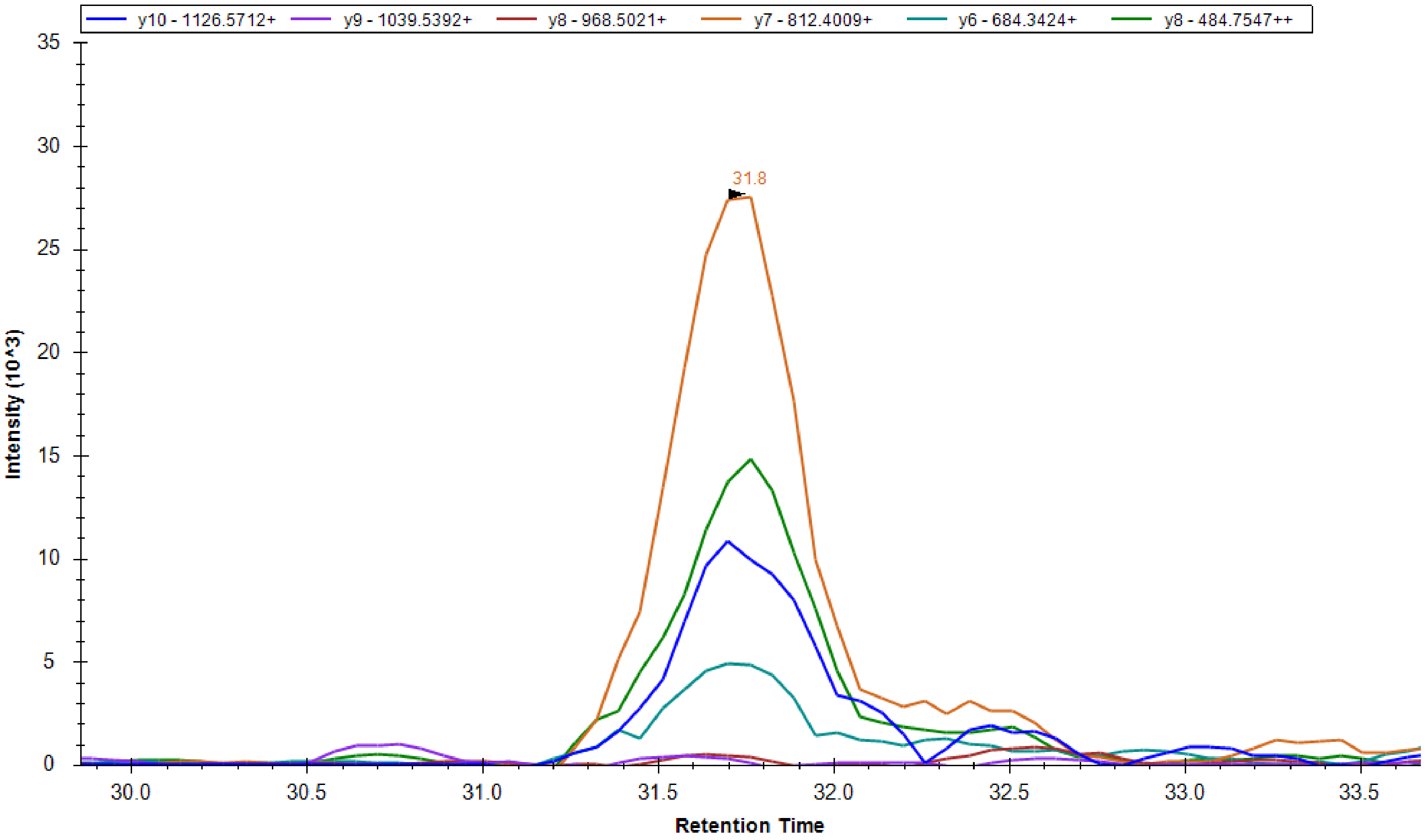

File name: 391-new#A-Round02_Negative-screen.skyd

Parent ion m/z and charges: 797.3841+++

NR_038436.2.1
EAIPIAQGHR

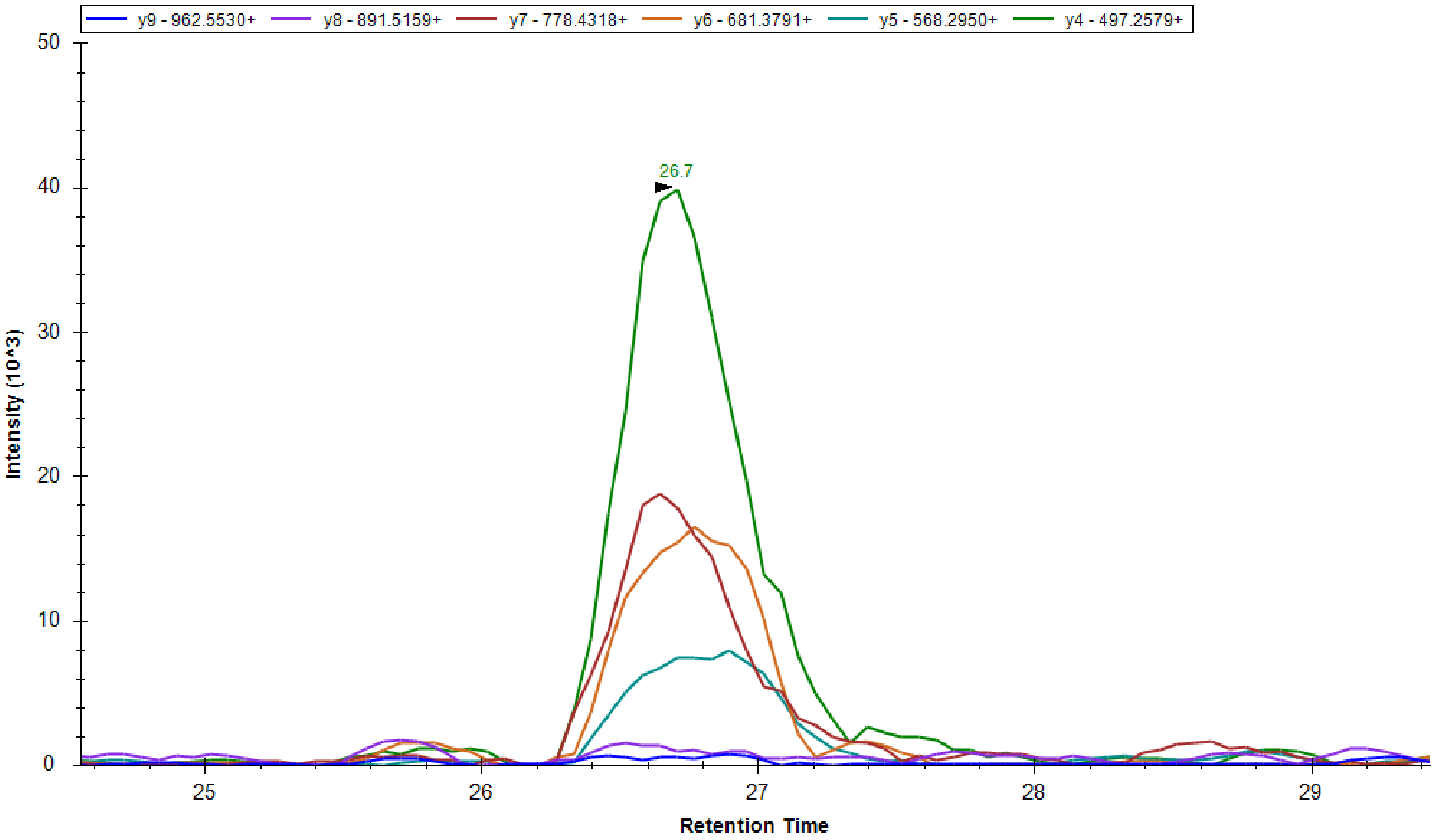

File name: 391-new#A-Round02_Negative-screen.skyd

Parent ion m/z and charges: 546.3014++

NR_038865.2.1
MGGAPDDTAR

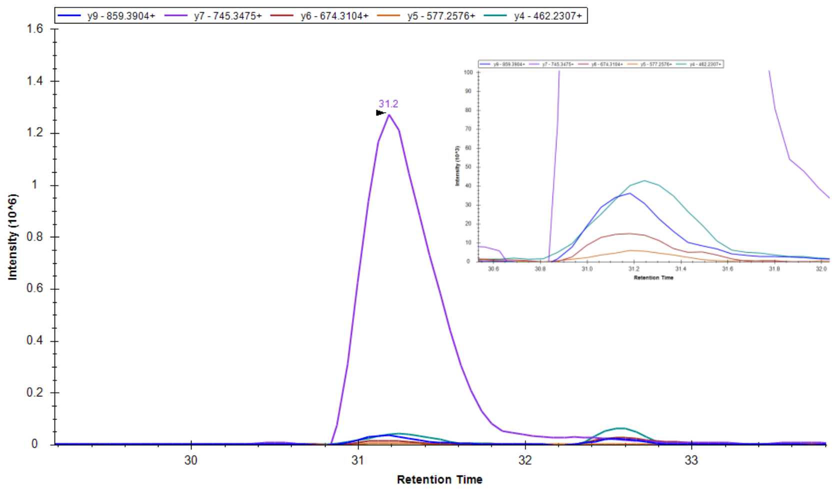

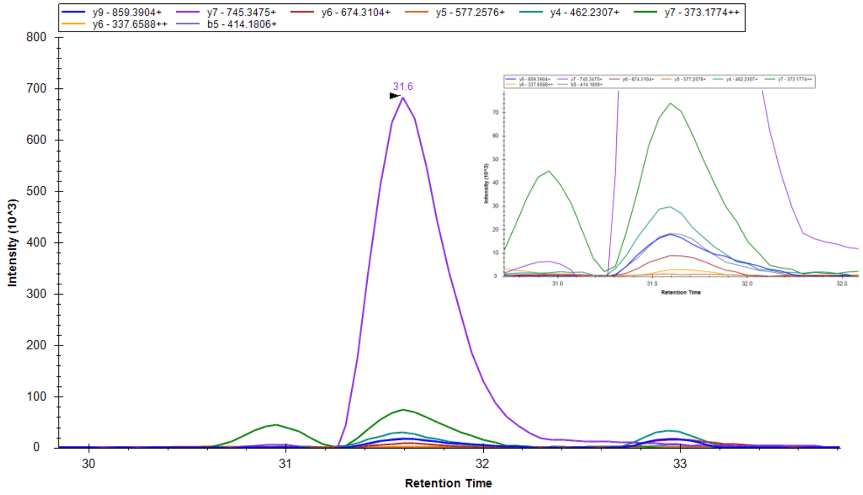

File name: 391-new#B-Round01_All-screening_Positive result.skyd

File name: 391-new#B-Round01_Positive-confirm.skyd

Parent ion m/z and charges: 495.7191++

NR_038865.2.1
MGGAPDDTAR

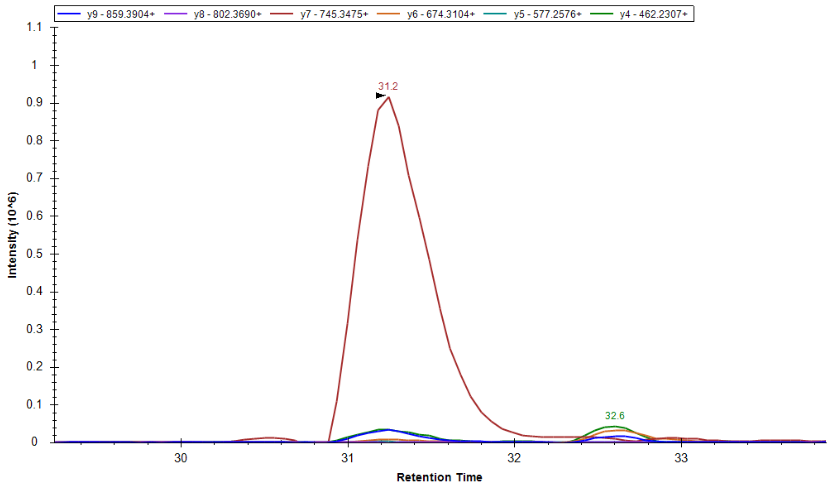

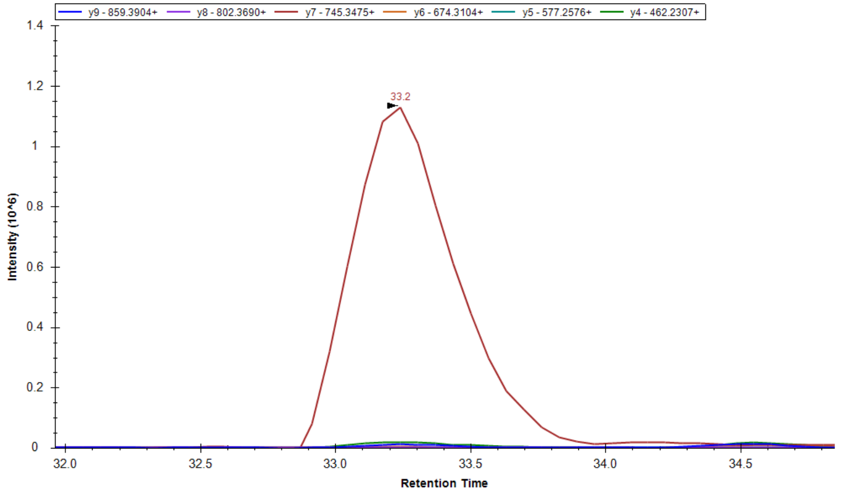

File name: 391-new#A-Round01_All-screening_Positive result.skyd

File name: 391-new#A-Round01_Positive-confirm.skyd

Parent ion m/z and charges: 495.7191++

NR_038874.2.1
WAFFPGRIRSA

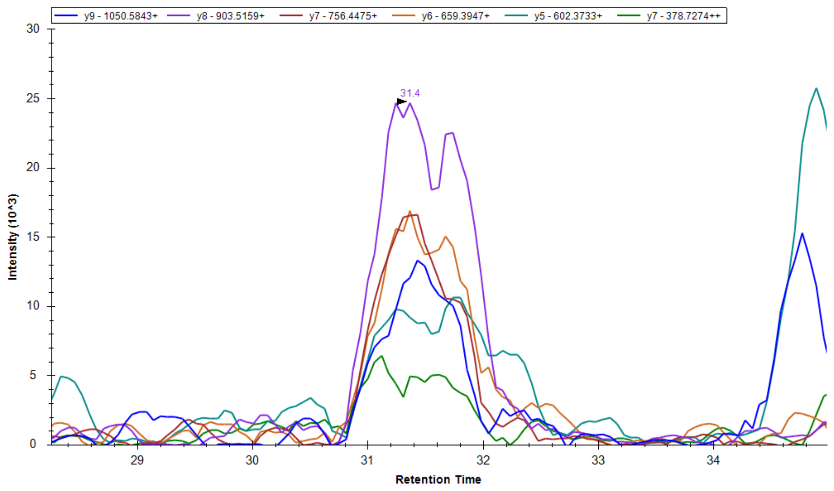

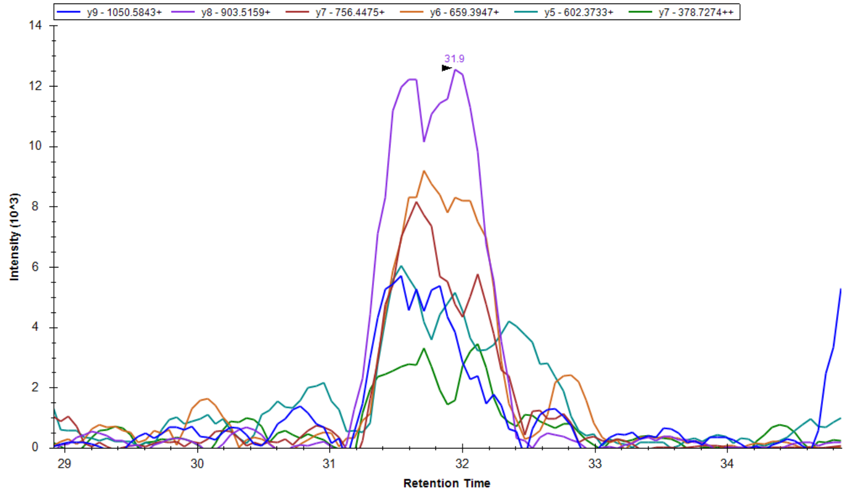

File name: 391-new#B-Round01_All-screening_Positive result.skyd

File name: 391-new#B-Round01_Positive-confirm.skyd

Parent ion m/z and charges: 654.3540++

NR_038874.2.1
WAFFPGRIRSA

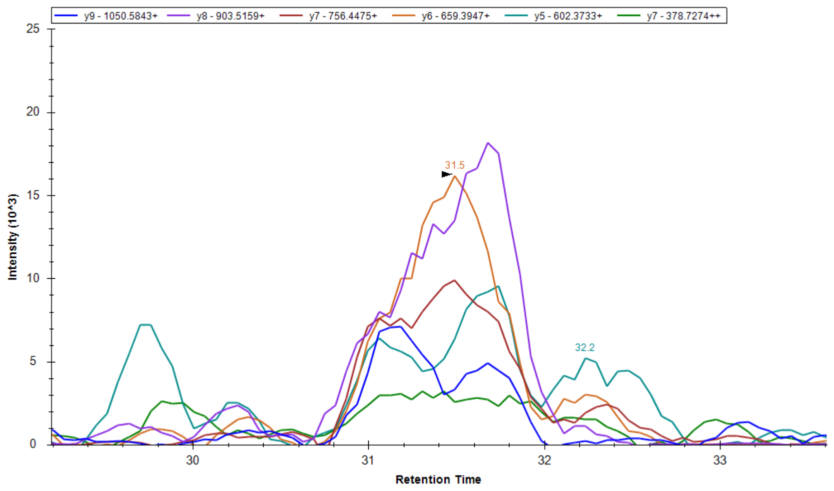

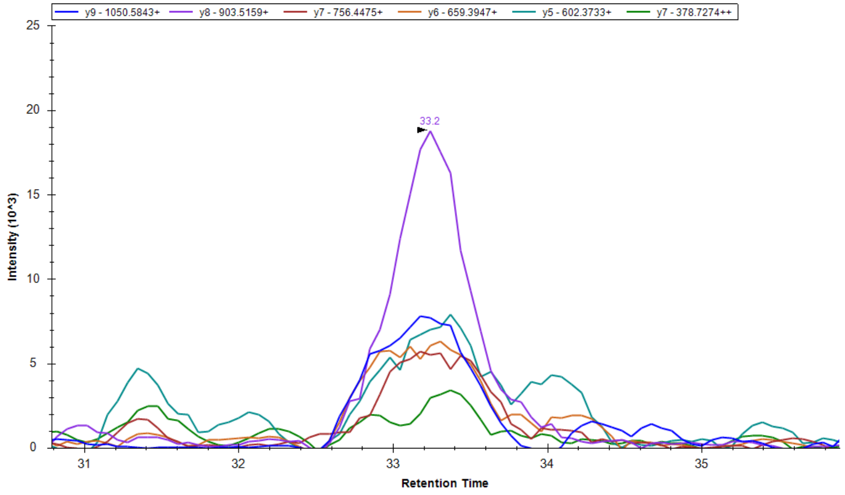

File name: 391-new#A-Round01_All-screening_Positive result.skyd

File name: 391-new#A-Round01_Positive-confirm.skyd

Parent ion m/z and charges: 654.3540++

NR_038897.3.3 EGGRAGVGVGAGACFDRSSSVVILSSPDR

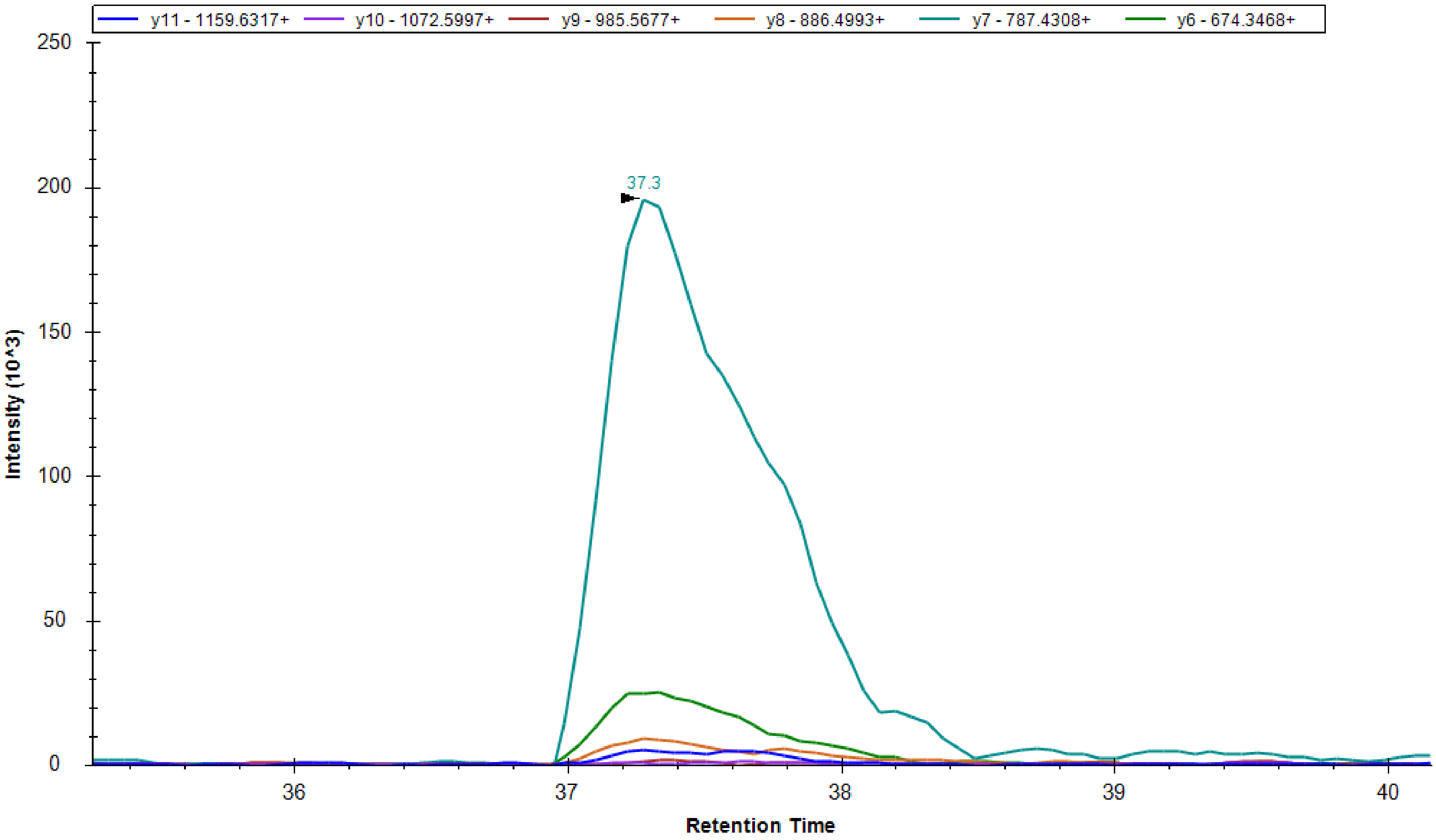

File name: 391-new#B-Round02_Negative-screen.skyd

Parent ion m/z and charges: 955.1420+++

NR_038897.3.3
EGGRAGVGVGAGACFDRSSSVVILSSPDR

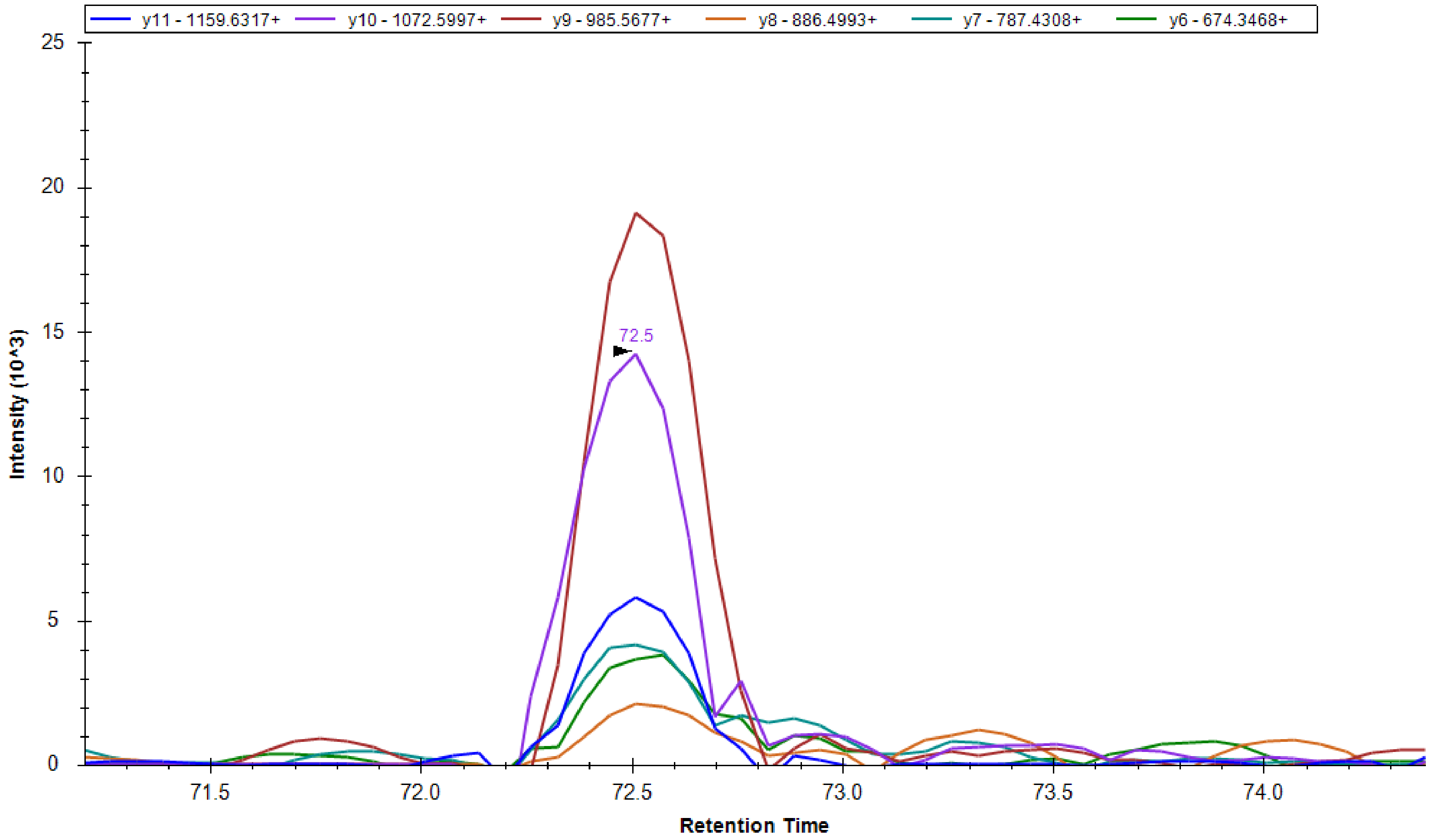

File name: 391-new#A-Round02_Negative-screen.skyd

Parent ion m/z and charges: 955.1420+++

NR_039981.2.18
MLPPFSVEPFSPLLPSKK

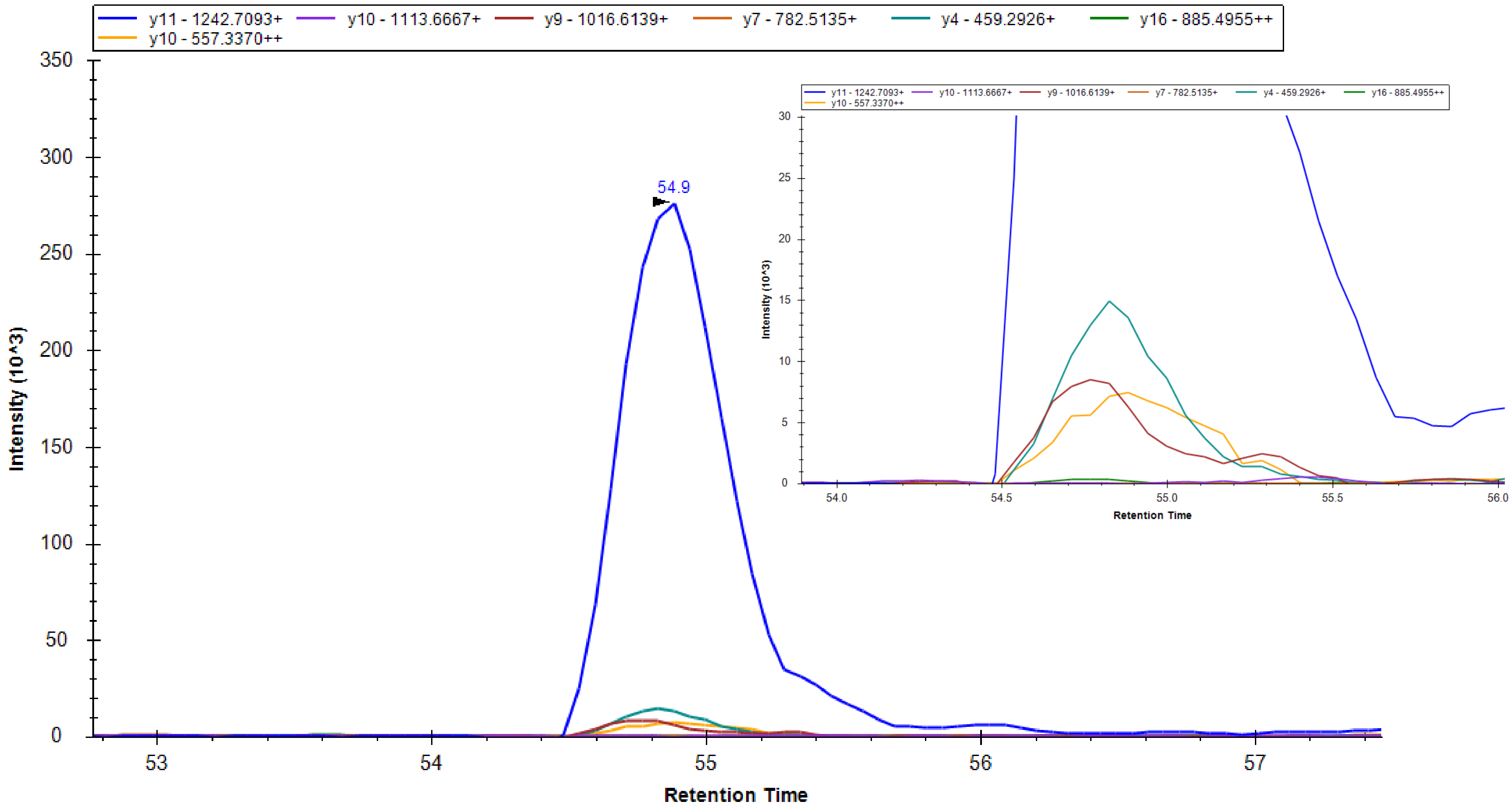

File name: 391-new#B-Round02_Negative-screen.skyd

Parent ion m/z and charges: 1007.5577++

NR_039981.2.18
MLPPFSVEPFSPLLPSKK

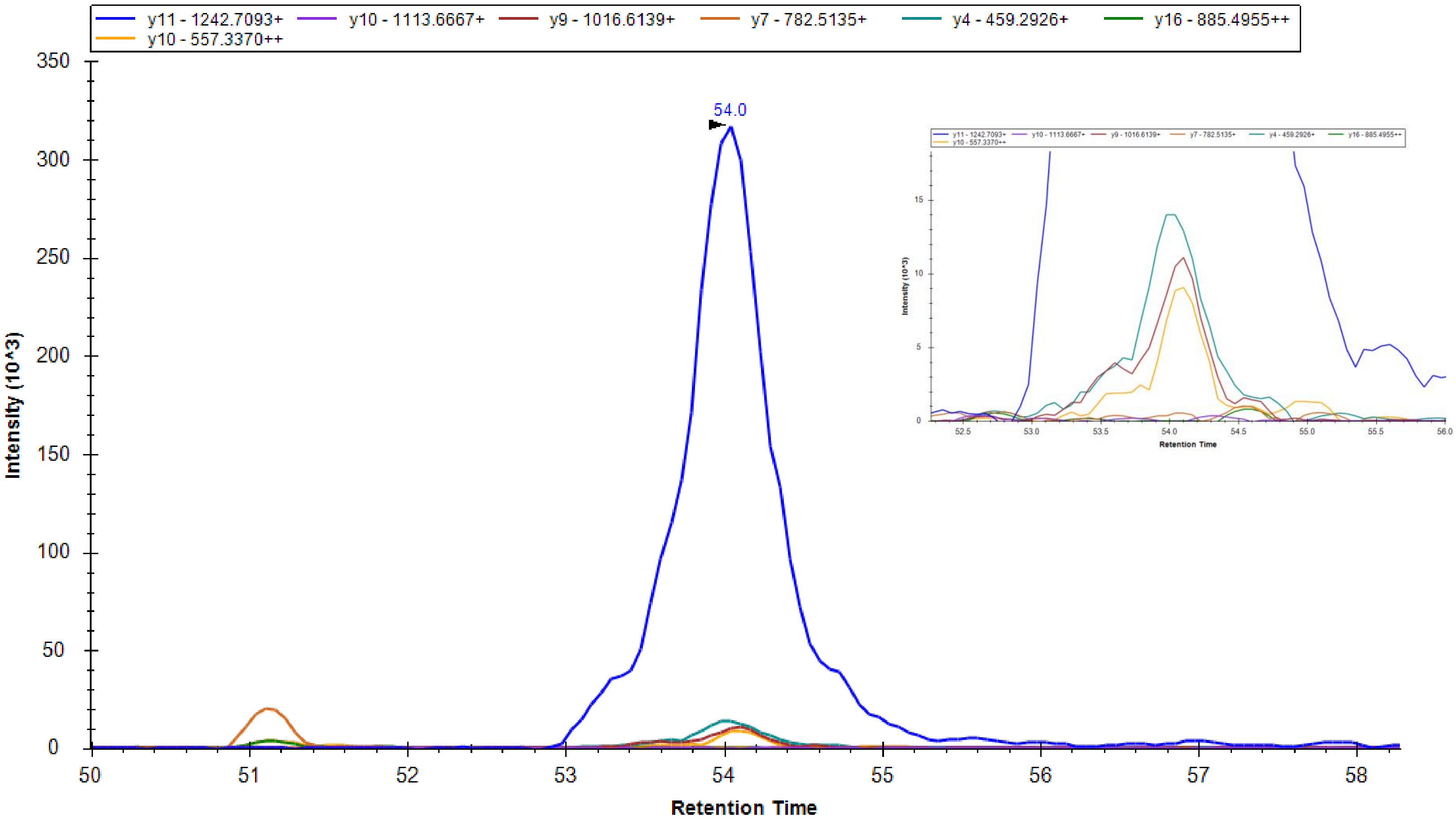

File name: 391-new#A-Round02_Negative-screen.skyd

Parent ion m/z and charges: 1007.5577++

NR_040093.3.5
VQDRTNLGFGQMK

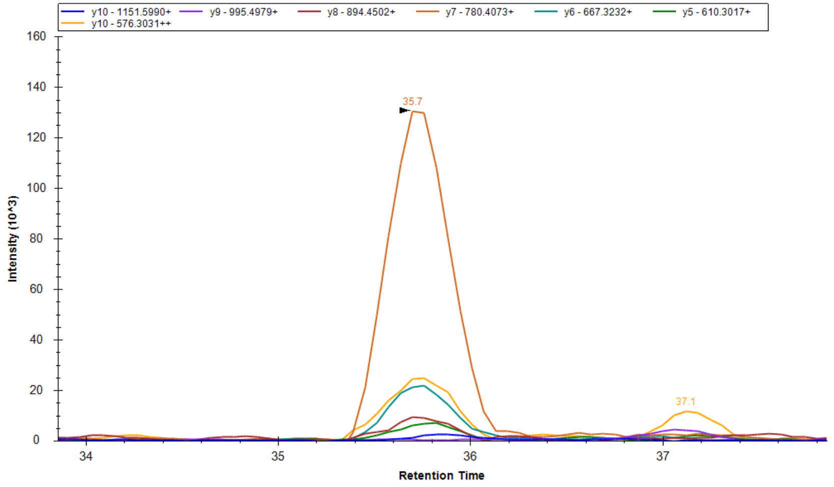

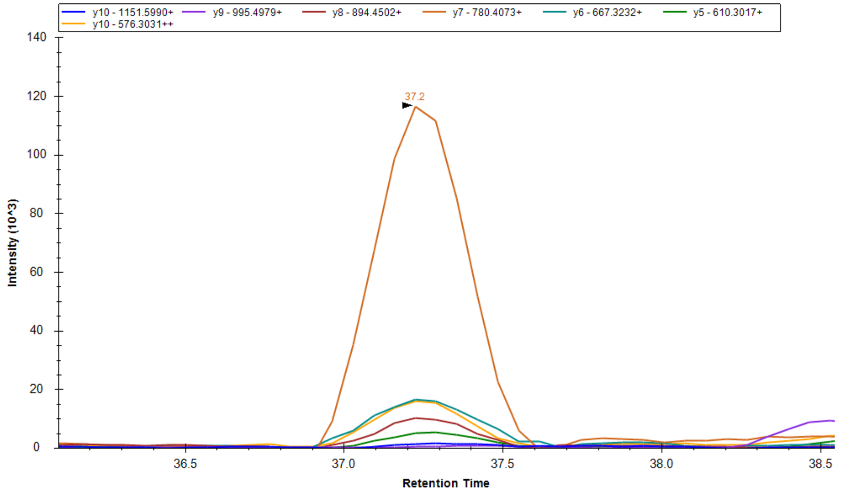

File name: 391-new#A-Round01_All-screening_Positive result.skyd

File name: 391-new#A-Round01_Positive-confirm.skyd

Parent ion m/z and charges: 747.3801++

NR_040434.2.3
MEVERNMFSFYDPLWGR

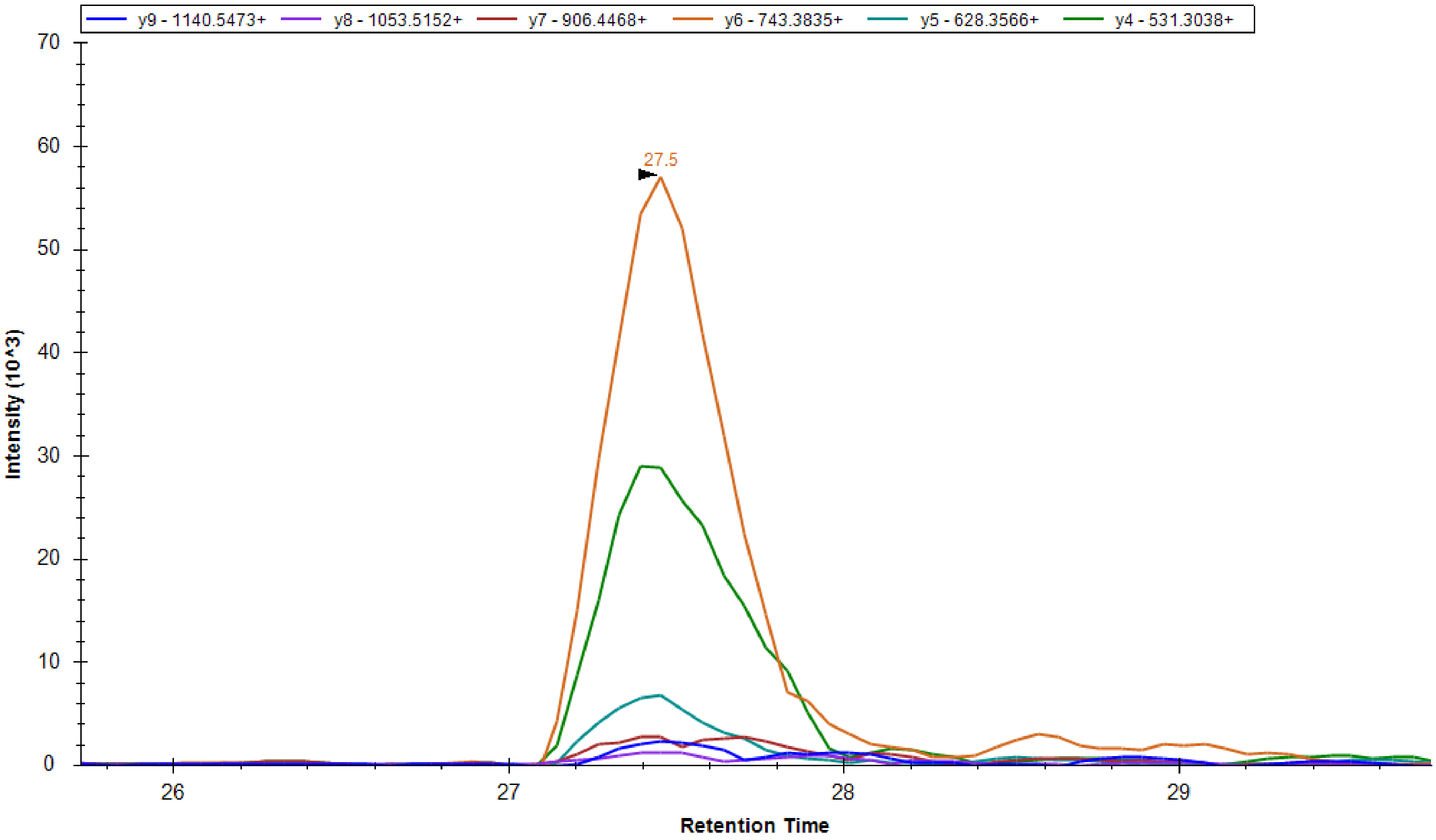

File name: 391-new#A-Round02_Negative-screen.skyd

Parent ion m/z and charges: 726.3363+++

NR_040434.2.3
MEVERNMFSFYDPLWGR

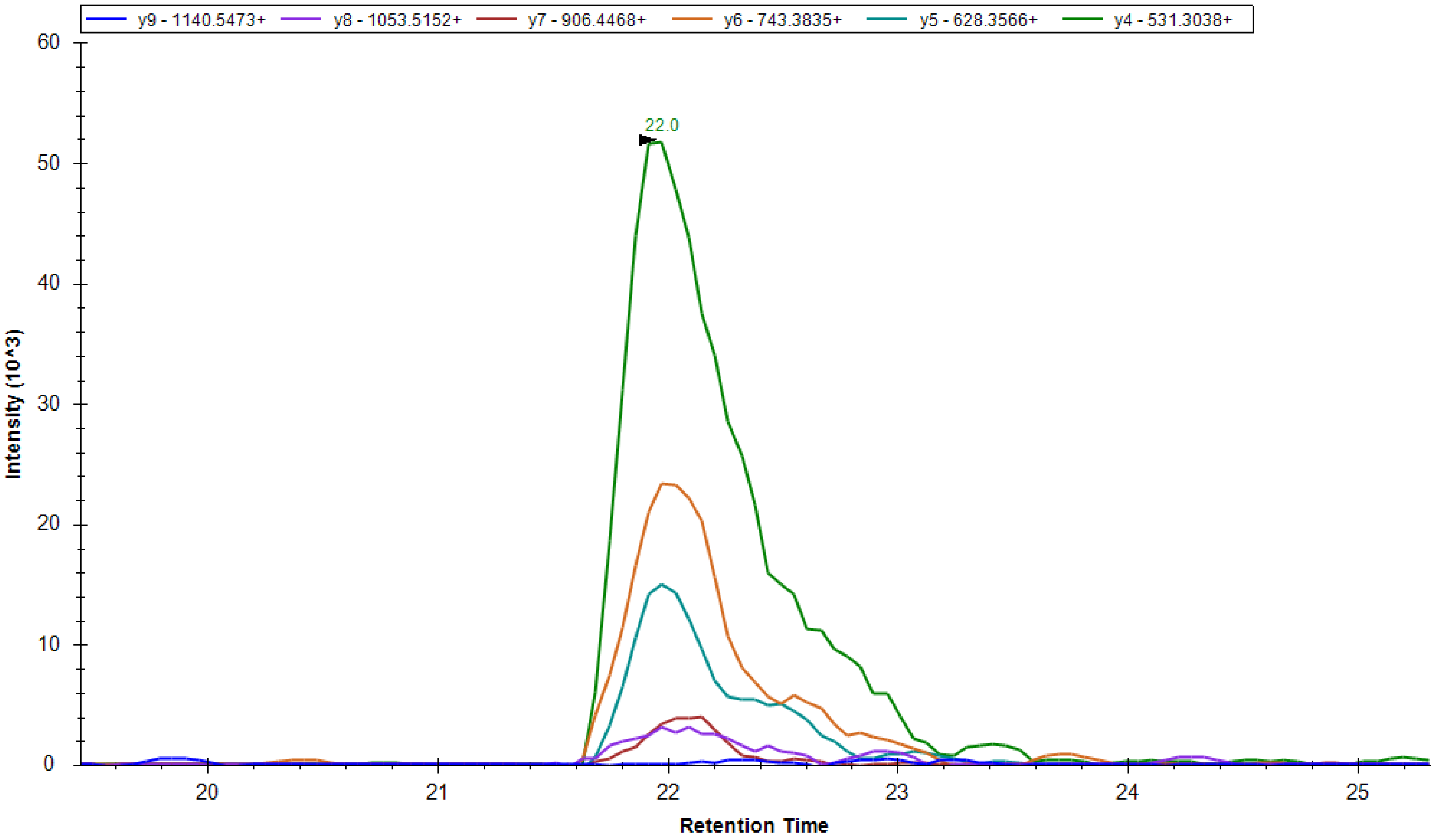

File name: 391-new#B-Round02_Negative-screen.skyd

Parent ion m/z and charges: 1089.0008++

NR_040434.2.3
MEVERNMFSFYDPLWGR

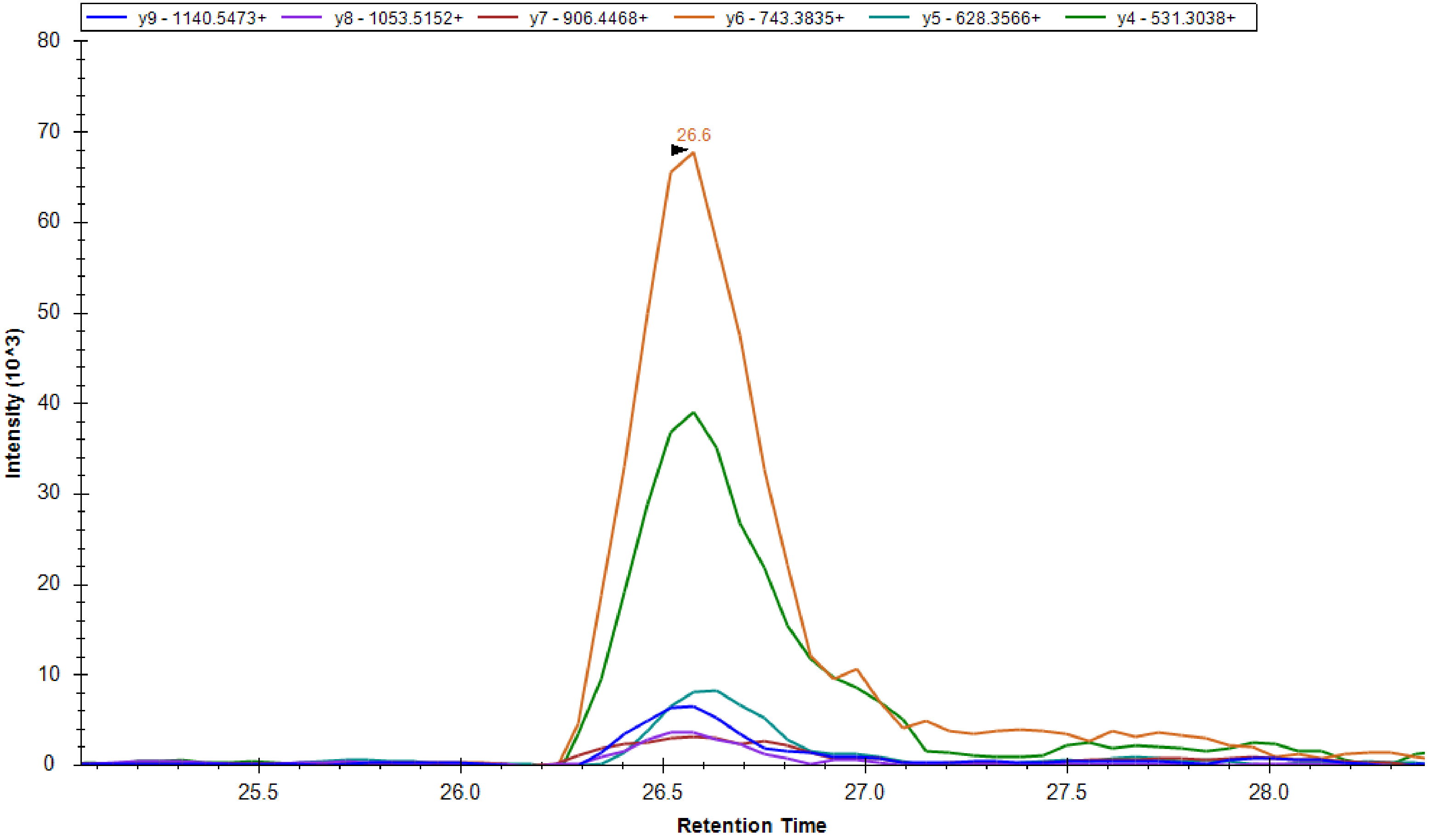

File name: 391-new#B-Round02_Negative-screen.skyd

Parent ion m/z and charges: 726.3363+++

NR_040434.2.3
MEVERNMFSFYDPLWGR

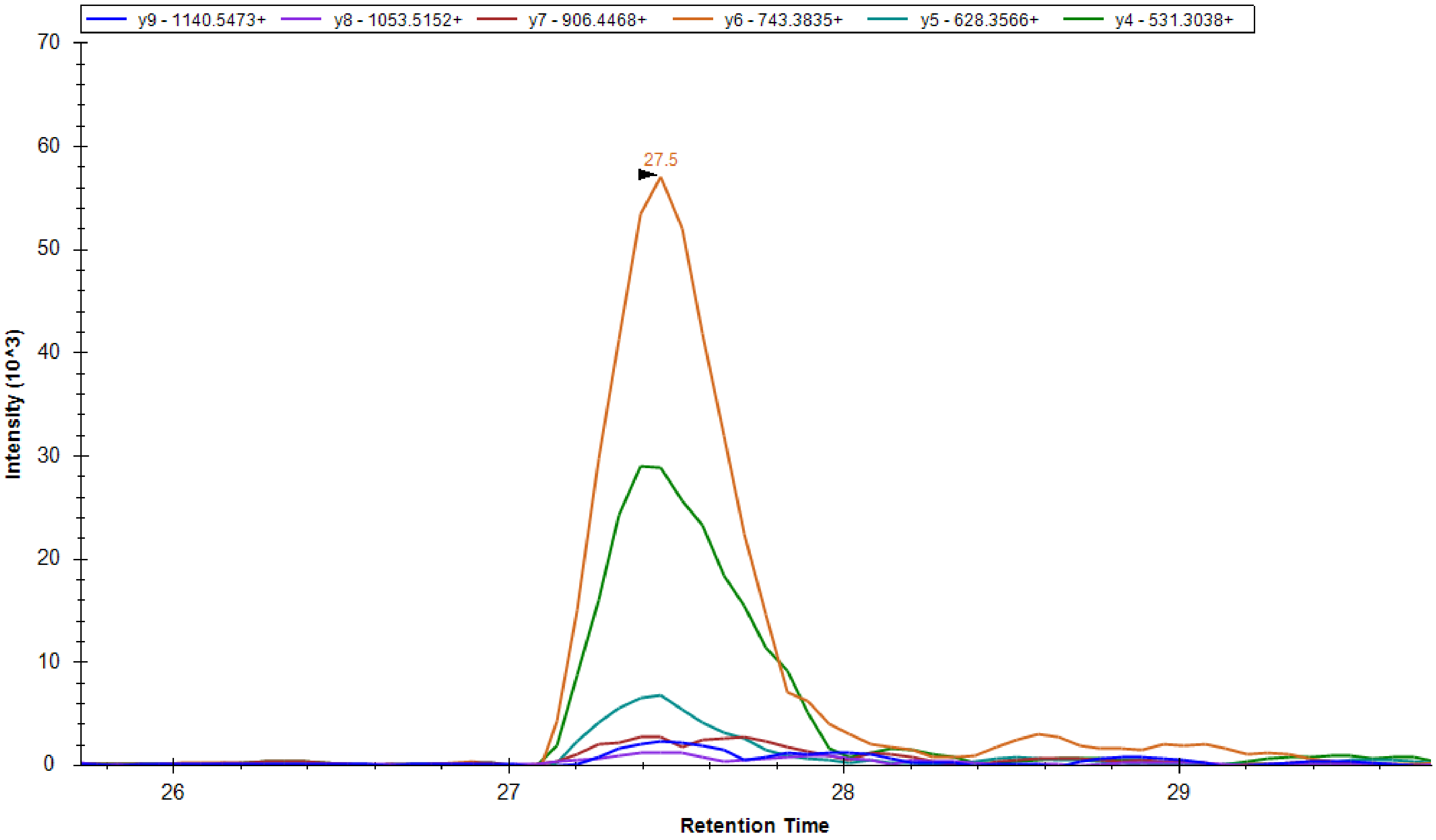

File name: 391-new#A-Round02_Negative-screen.skyd

Parent ion m/z and charges: 726.3363+++

NR_046337.3.1
SYIGSETEFR

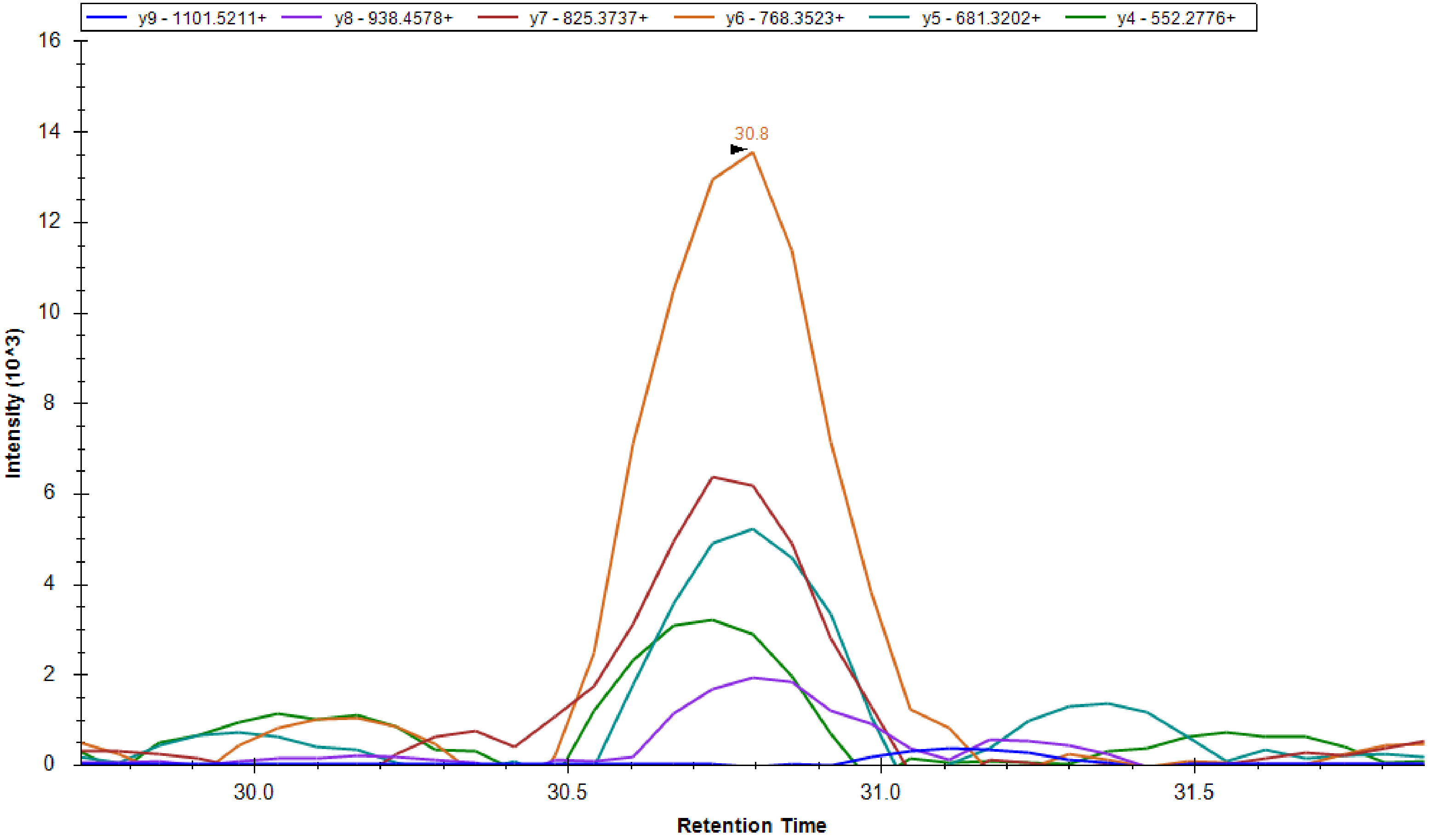

File name: 391-new#A-Round02_Negative-screen.skyd

Parent ion m/z and charges: 594.7802++

NR_046423.2.4
TQSISPPWKNVWTLGR

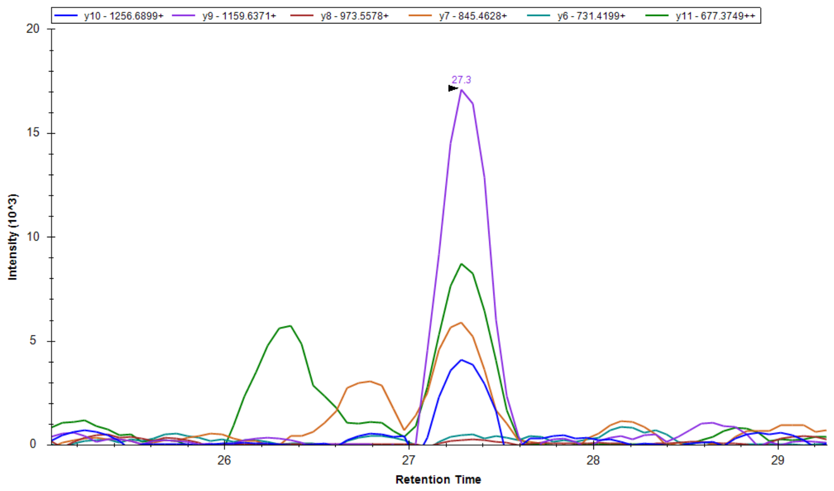

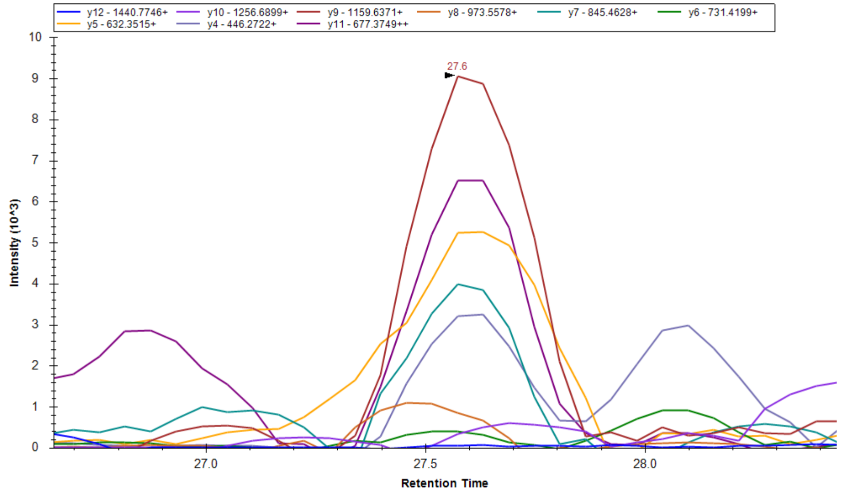

File name: 391-new#B-Round01_All-screening_Positive result.skyd

File name: 391-new#B-Round01_Positive-confirm.skyd

Parent ion m/z and charges: 935.5021++

NR_046507.1.6
ASDRSAFAFLYINFTMSR

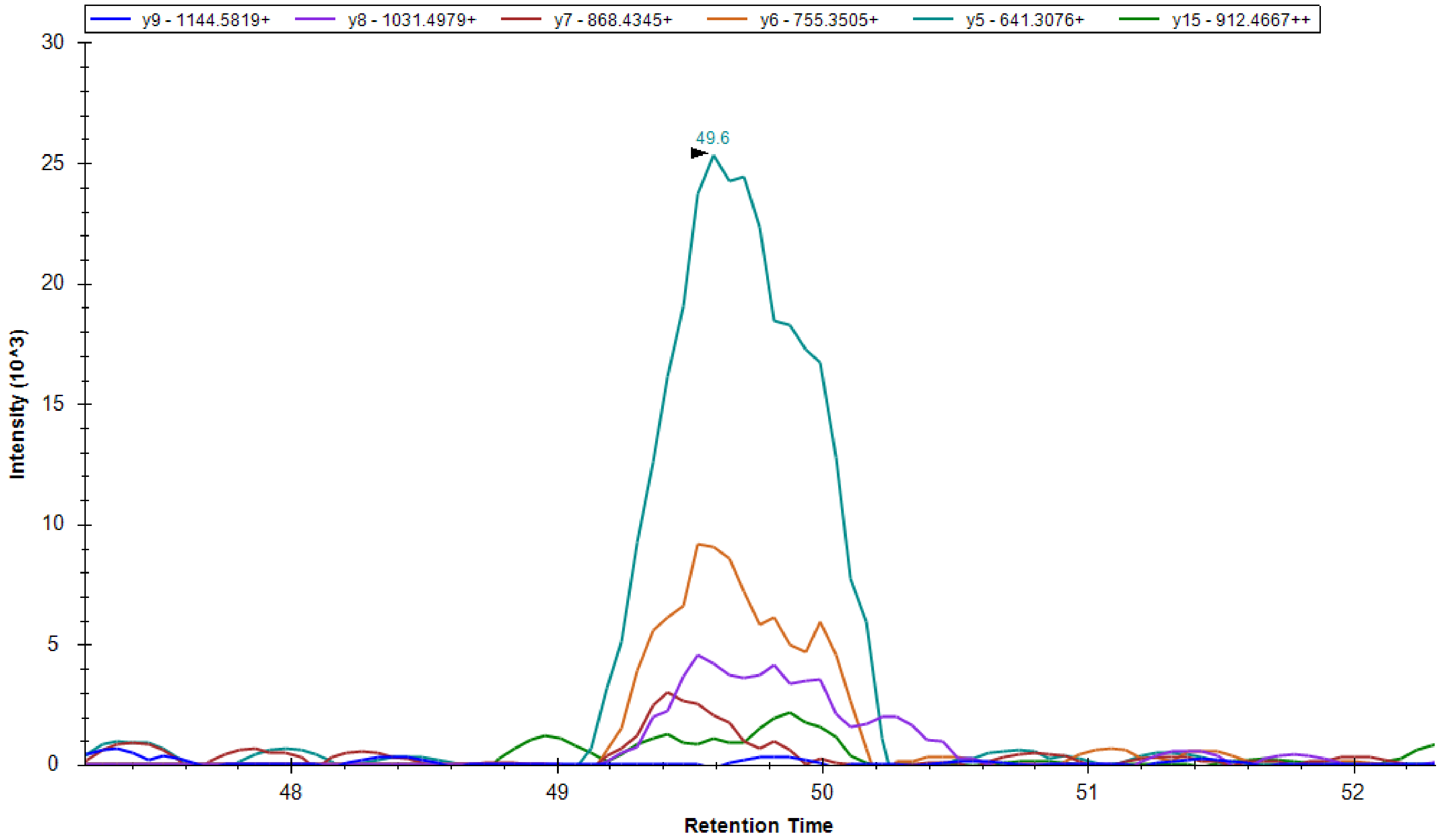

File name: 391-new#B-Round02_Negative-screen.skyd

Parent ion m/z and charges: 699.6789+++

NR_047572.3.2
SPAAPAQRGR

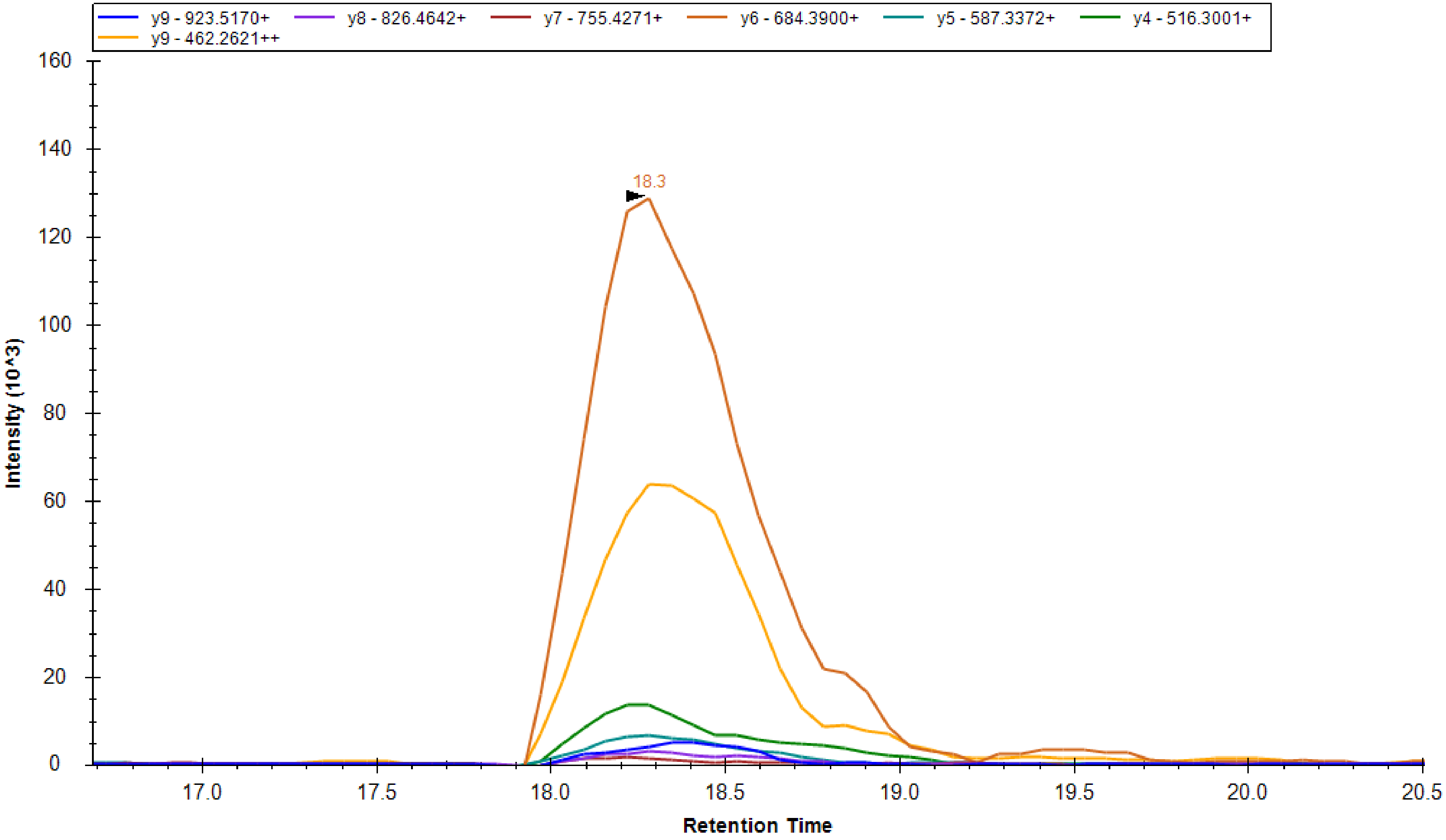

File name: 391-new#A-Round02_Negative-screen.skyd

Parent ion m/z and charges: 505.7781++

NR_049772.2.2
MKLSSSSVMCLPLFSGLR

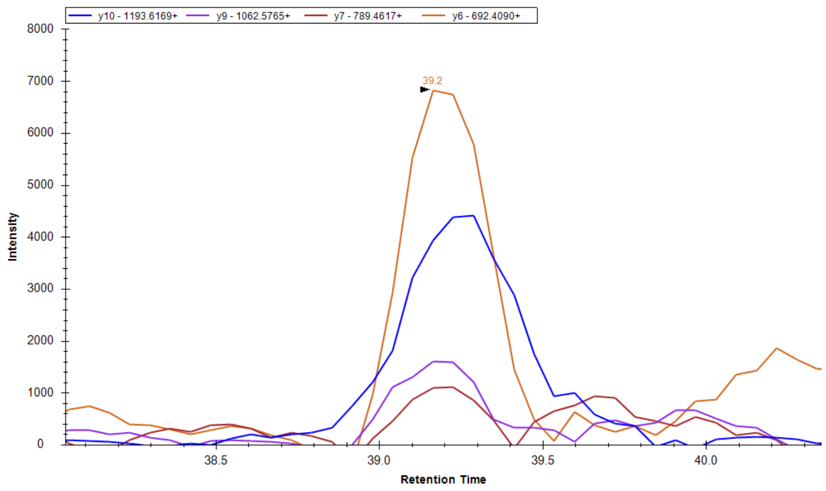

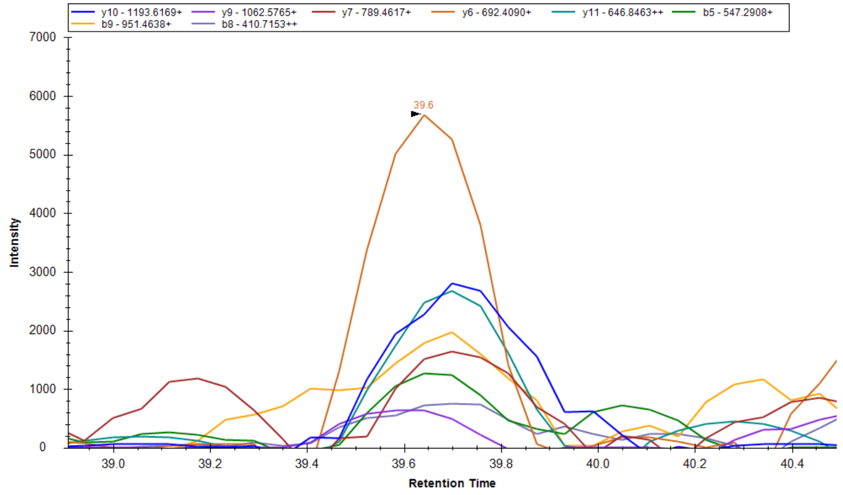

File name: 391-new#B-Round01_All-screening_Positive result.skyd

File name: 391-new#B-Round01_Positive-confirm.skyd

Parent ion m/z and charges: 1007.0201++

NR_072996.1.9
LGLLLVPVR

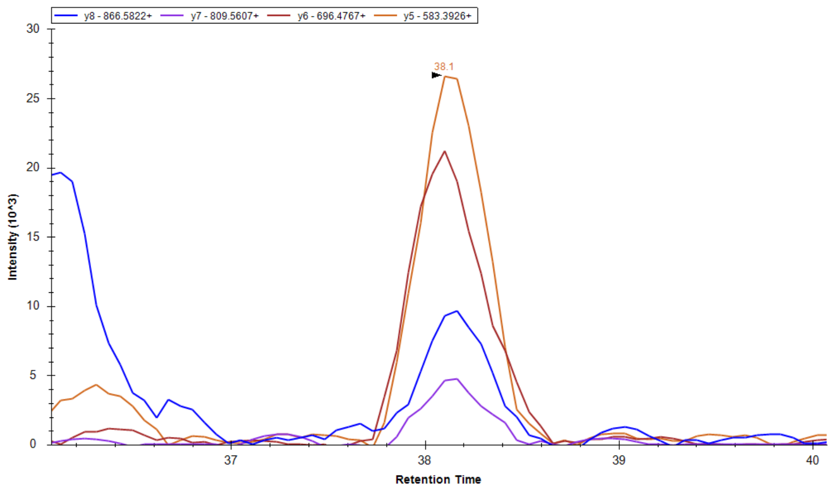

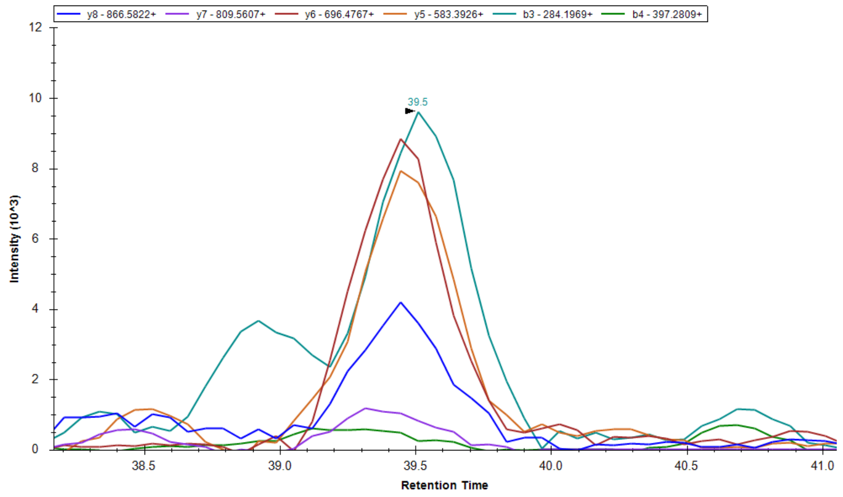

File name: 391-new#A-Round01_All-screening_Positive result.skyd

File name: 391-new#A-Round01_Positive-confirm.skyd

Parent ion m/z and charges: 490.3368++

NR_072996.1.9
LGLLLVPVR

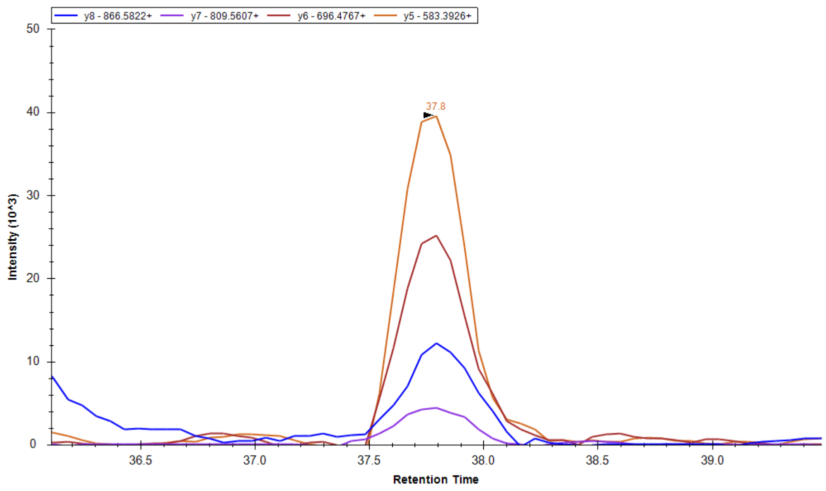

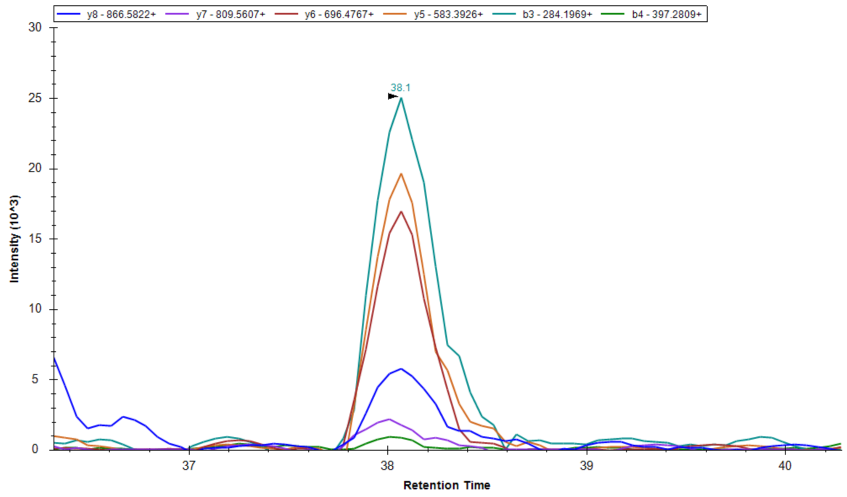

File name: 391-new#B-Round01_All-screening_Positive result.skyd

File name: 391-new#B-Round01_Positive-confirm.skyd

Parent ion m/z and charges: 490.3368++

NR_073029.3.2
CEPPCLAVTNLQGGLRGIAR

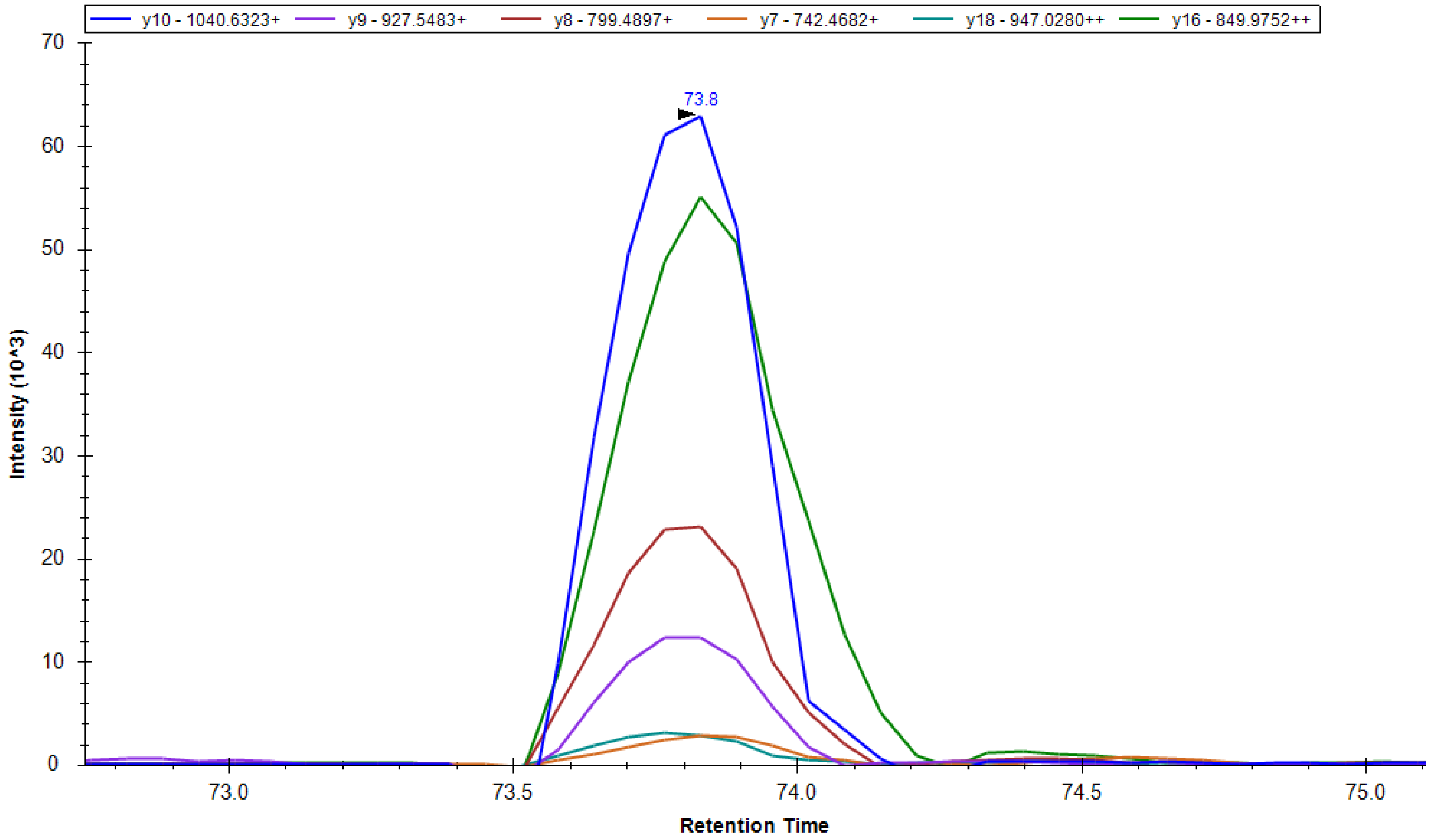

File name: 391-new#A-Round02_Negative-screen.skyd

Parent ion m/z and charges: 728.0455+++

NR_073029.3.2
CEPPCLAVTNLQGGLRGIAR

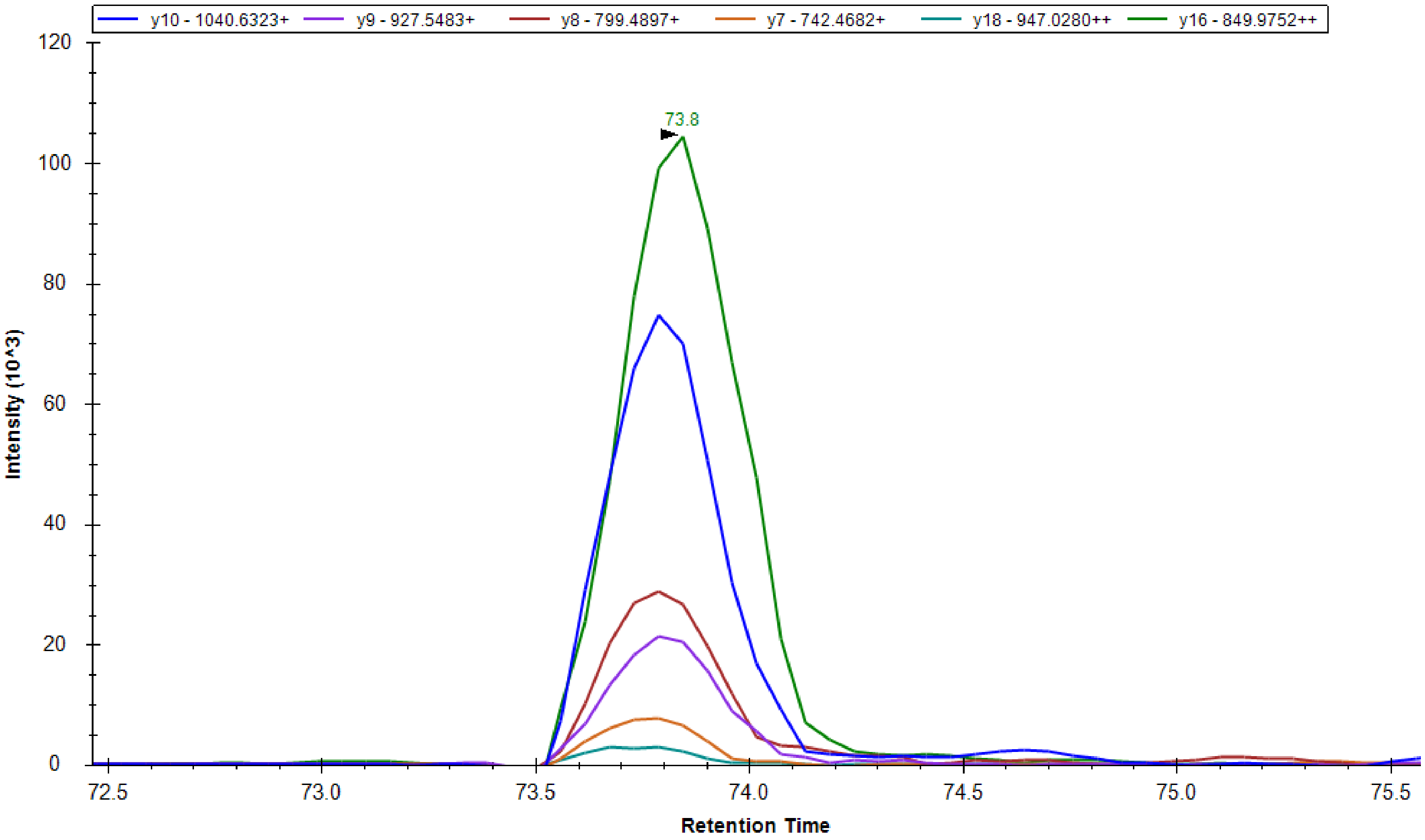

File name: 391-new#B-Round02_Negative-screen.skyd

Parent ion m/z and charges: 728.0455+++

NR_073430.1.10
EEPALVGER

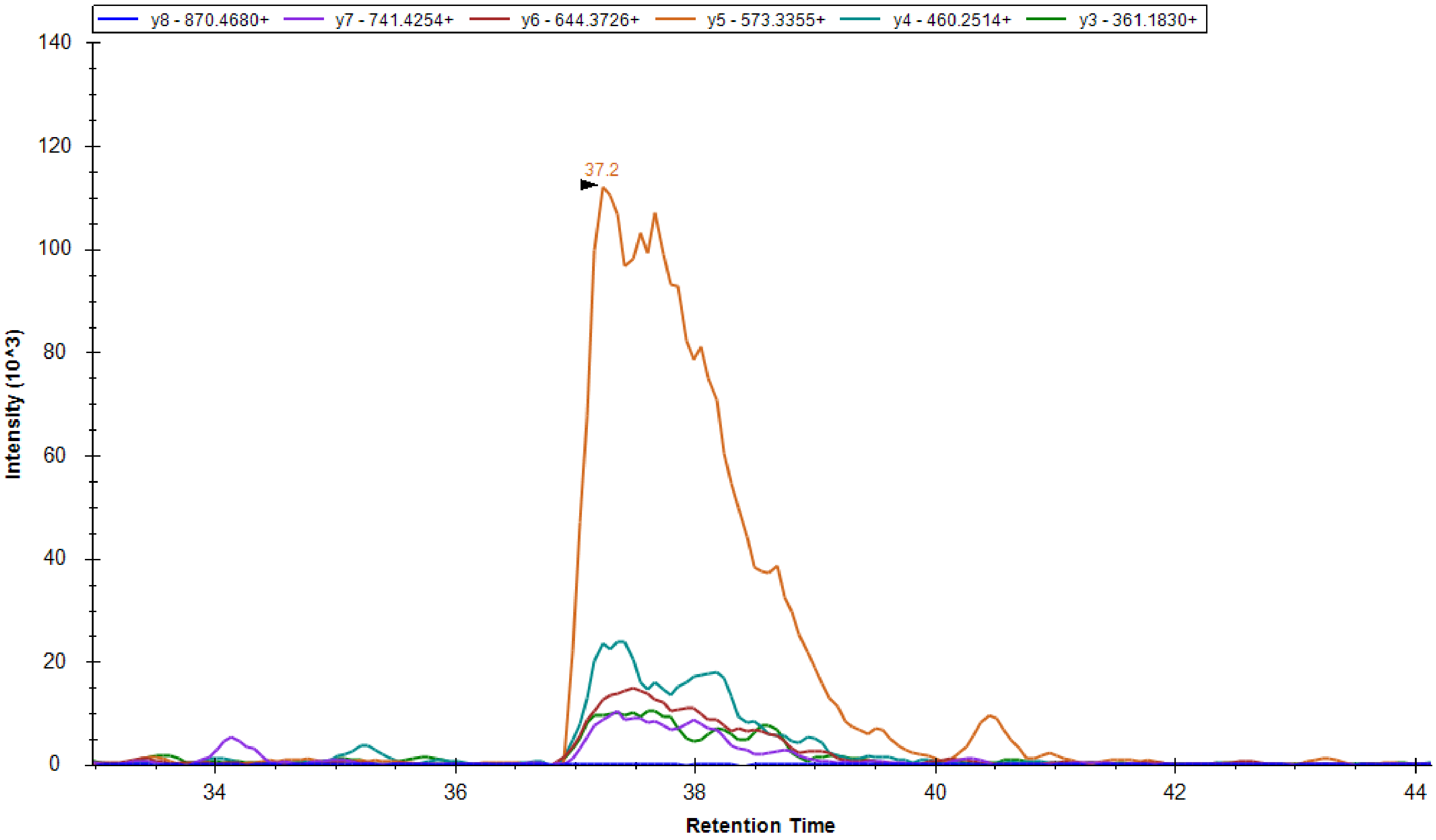

File name: 391-new#A-Round02_Negative-screen.skyd

Parent ion m/z and charges: 500.2589++

NR_073430.1.10
EEPALVGER

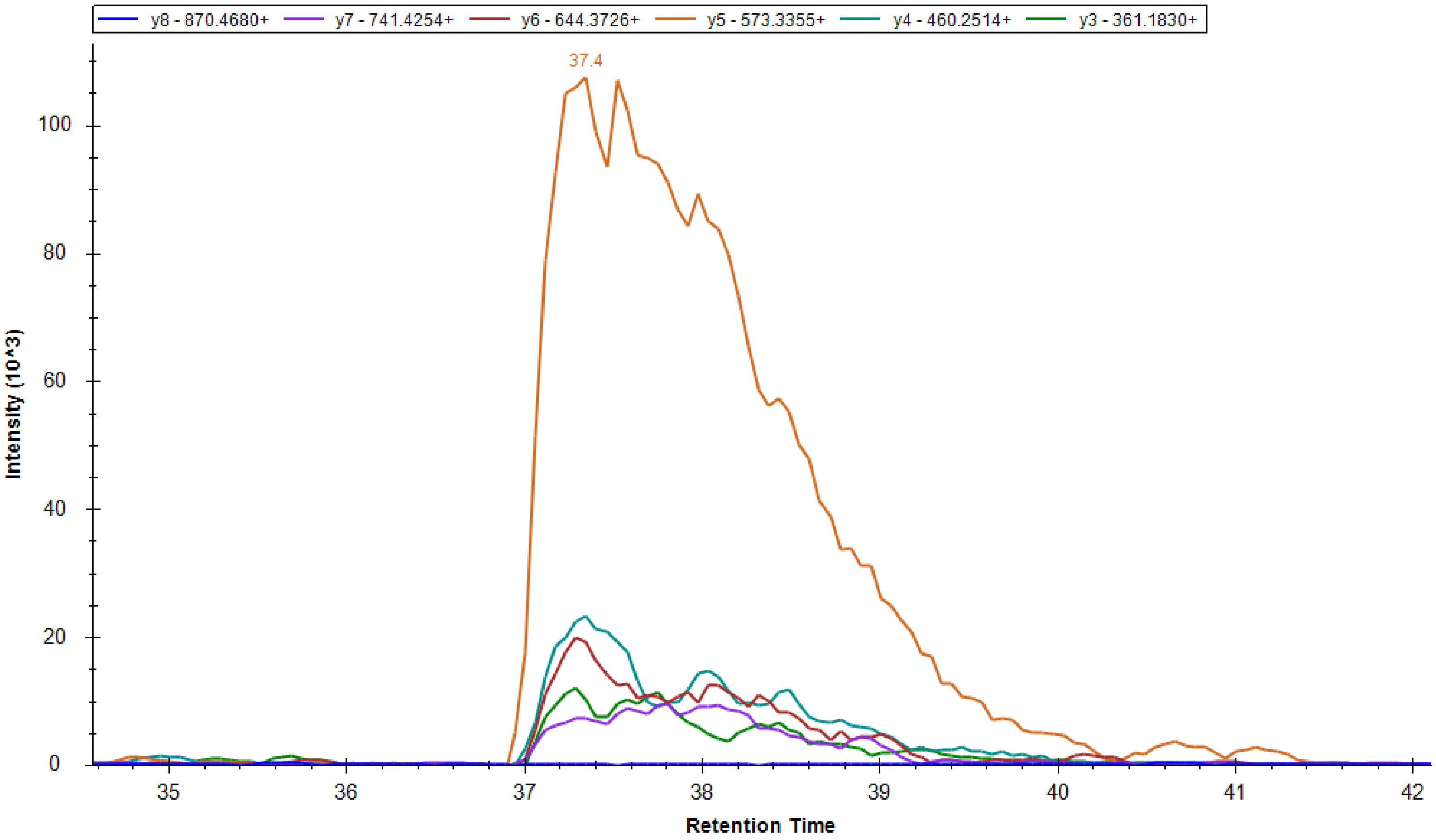

File name: 391-new#B-Round02_Negative-screen.skyd

Parent ion m/z and charges: 500.2589++

NR_075100.1.7
ISEPCIKGTWSIIWR

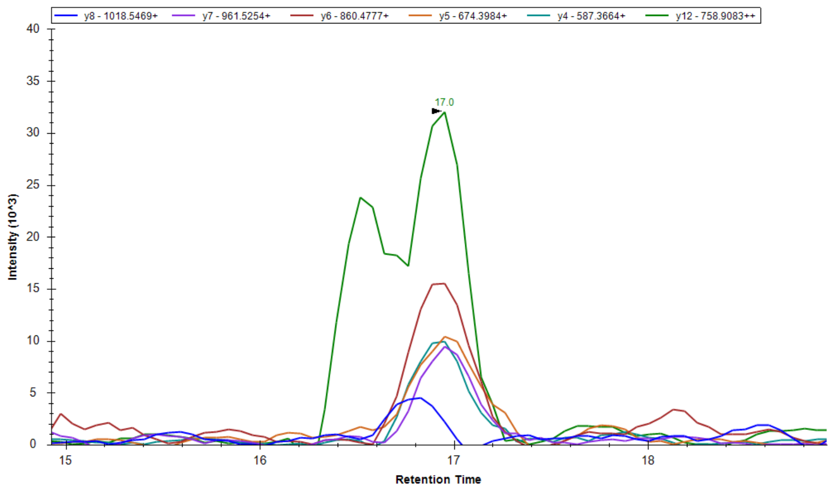

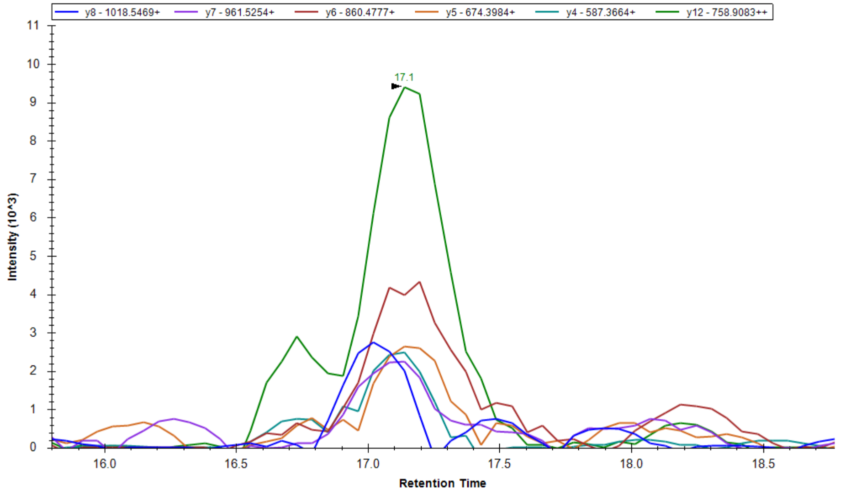

File name: 391-new#B-Round01_All-screening_Positive result.skyd

File name: 391-new#B-Round01_Positive-confirm.skyd

Parent ion m/z and charges: 615.9942+++

NR_075100.1.7
ISEPCIKGTWSIIWR

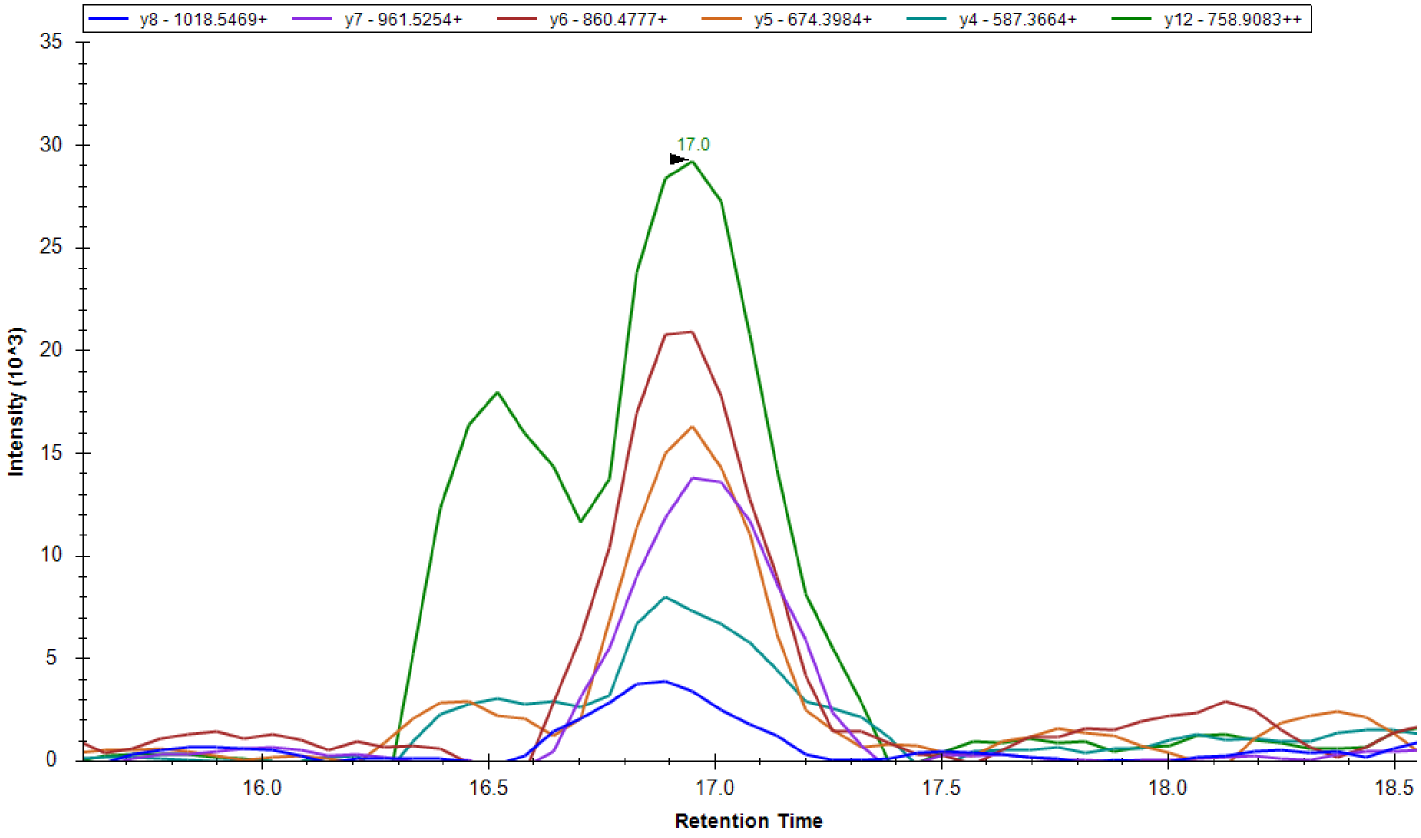

File name: 391-new#A-Round01_All-screening_Positive result.skyd

Parent ion m/z and charges: 615.9942+++

NR_075100.2.16
MYVSISHGYIPK

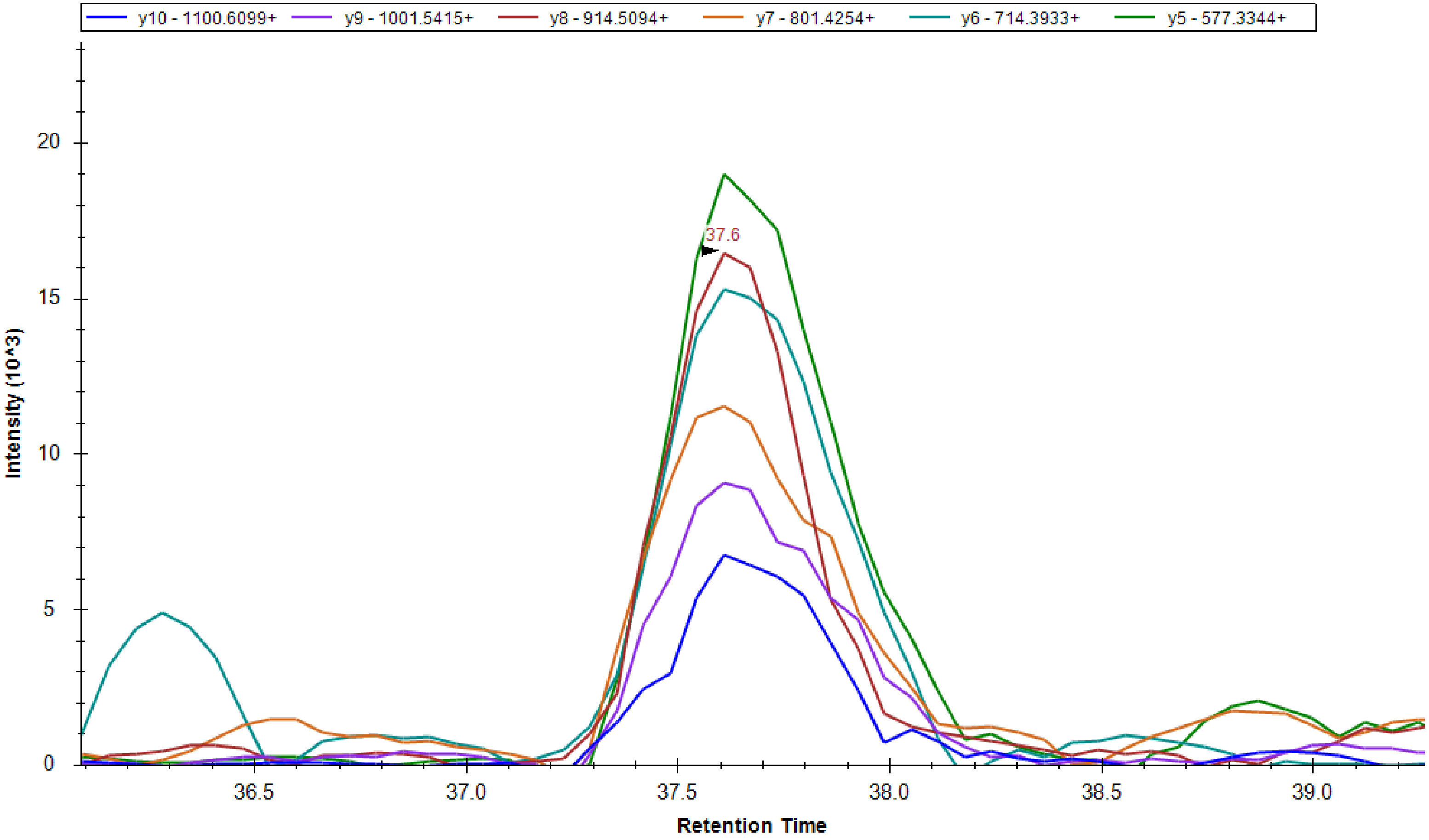

File name: 391-new#A-Round02_Negative-screen.skyd

Parent ion m/z and charges: 697.8605++

NR_075100.2.6
MLGHGHKGLTER

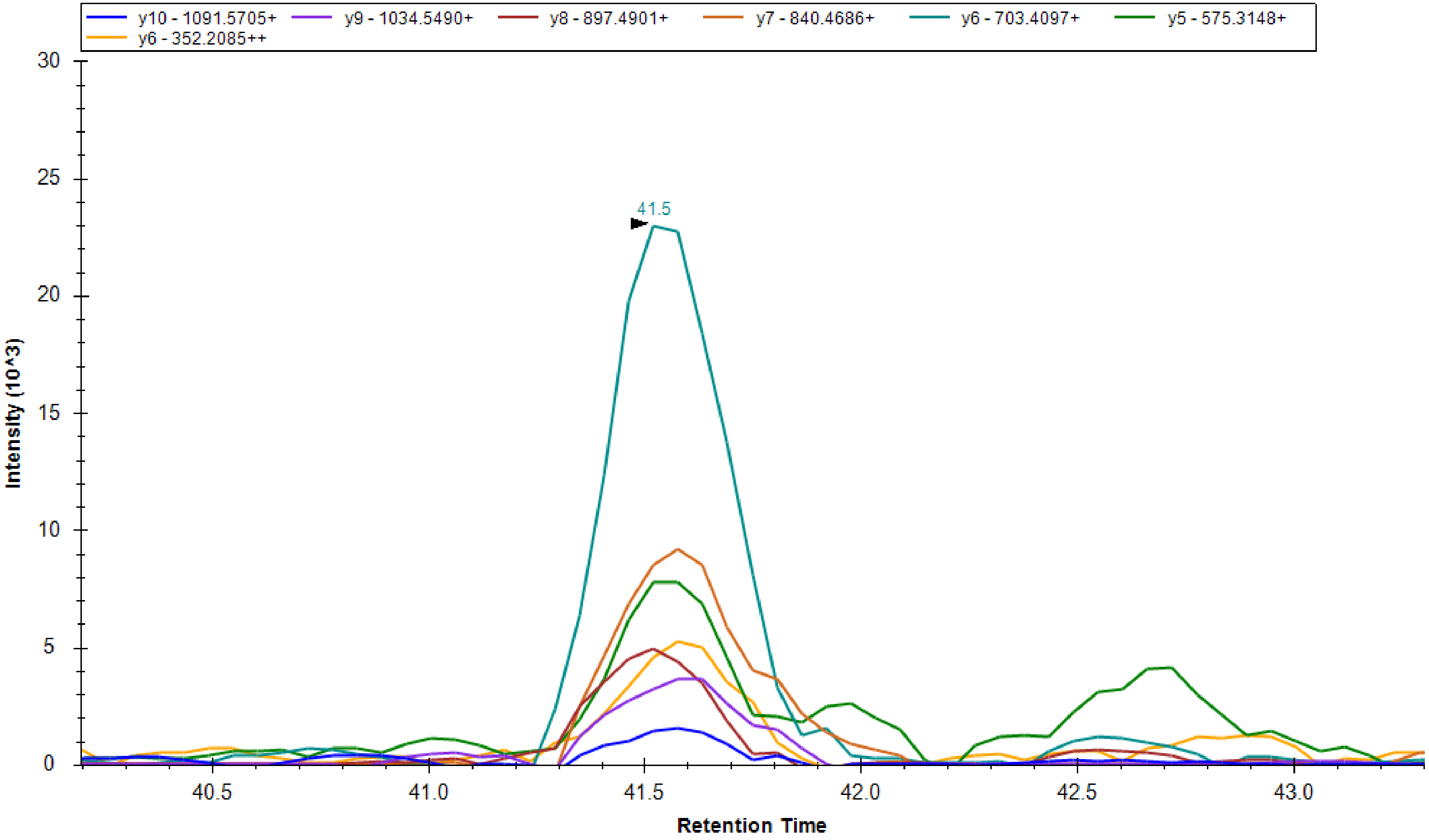

File name: 391-new#B-Round02_Negative-screen.skyd

Parent ion m/z and charges: 668.3511++

NR_077215.1.1
GPKEEHNALIGTGK

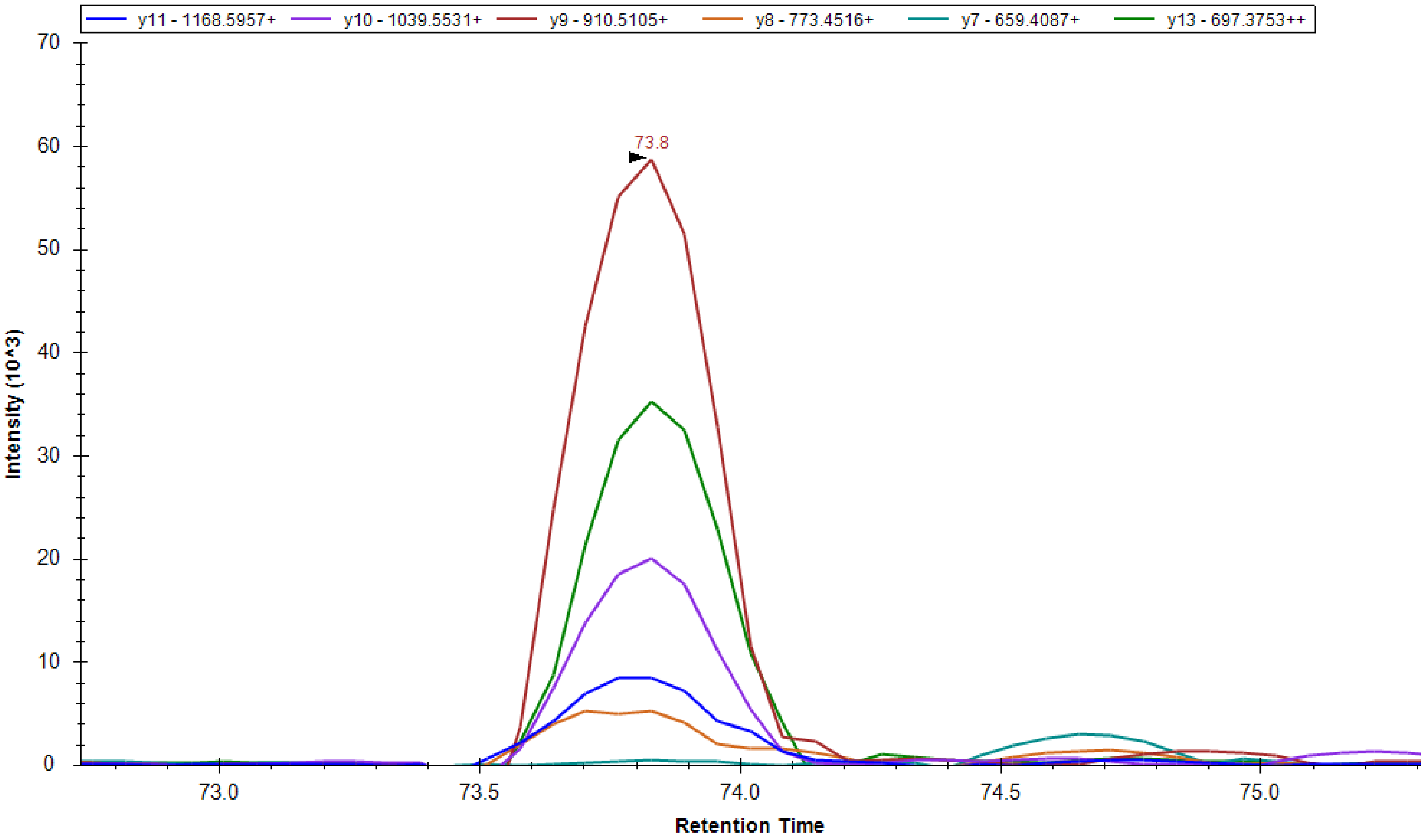

File name: 391-new#A-Round02_Negative-screen.skyd

Parent ion m/z and charges: 725.8861++

NR_077215.3.2
LMPSLLCGMVAKDSK

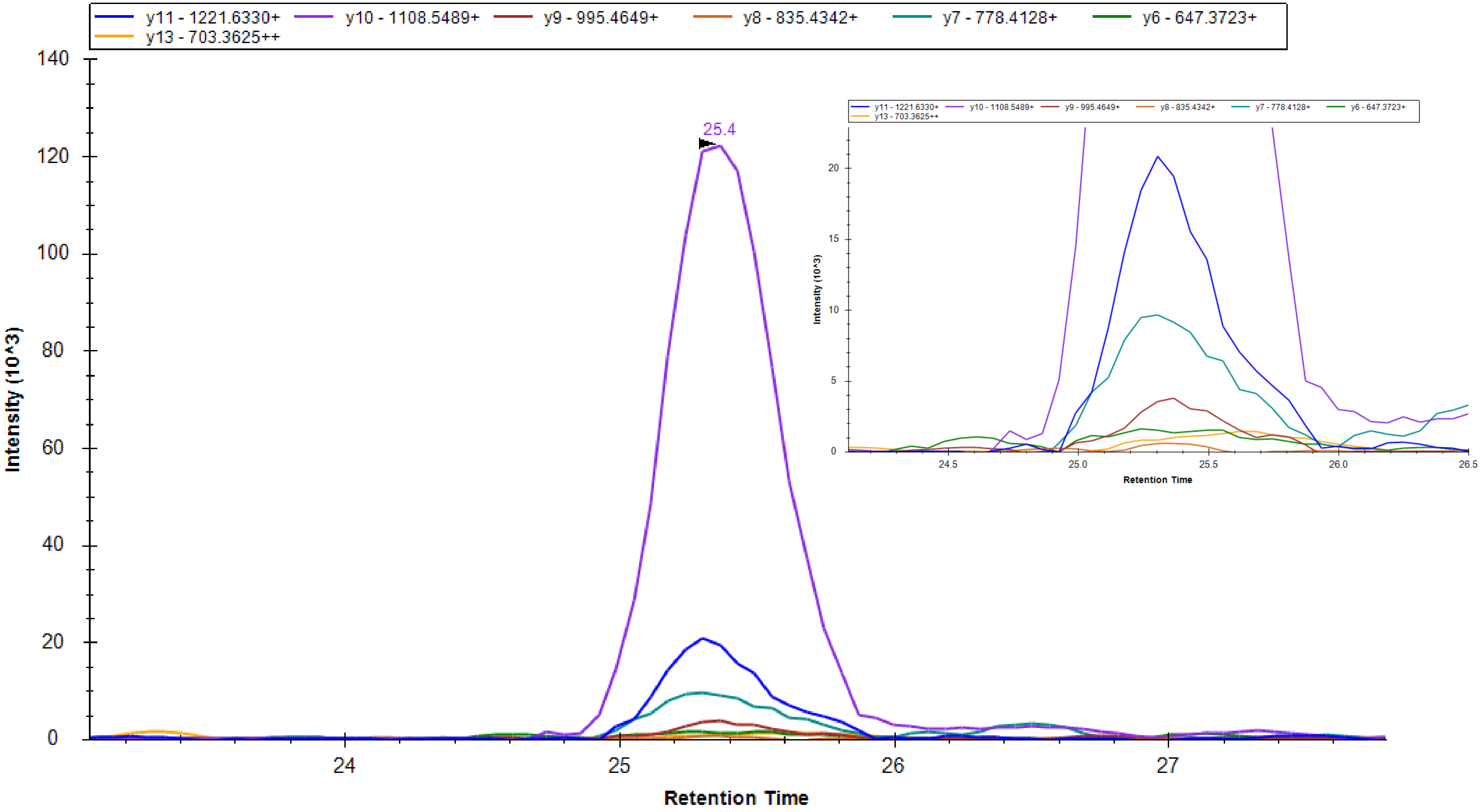

File name: 391-new#A-Round02_Negative-screen.skyd

Parent ion m/z and charges: 825.4248++

NR_102735.1.3
QDPPIPGPGAAPAEWG

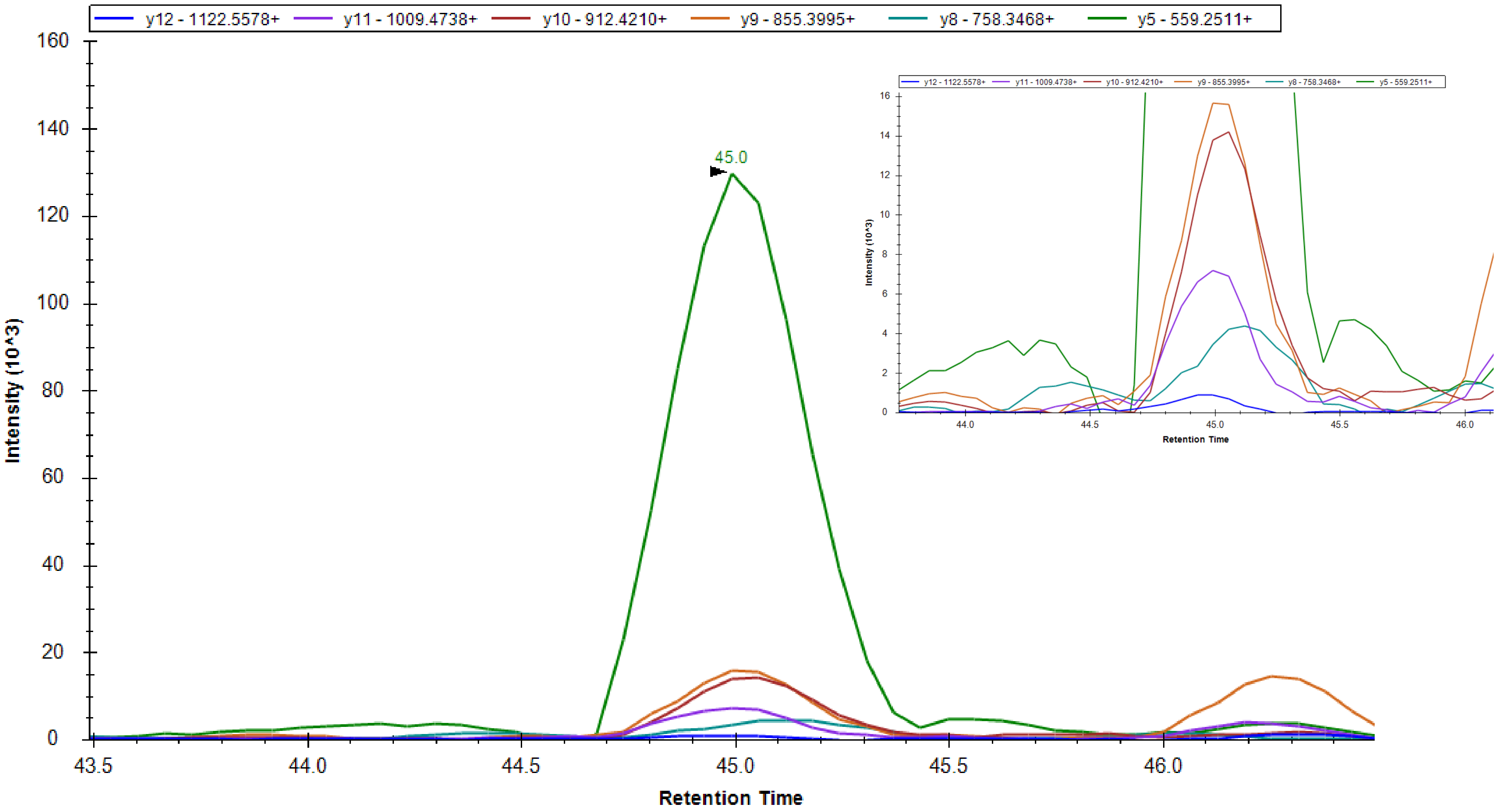

File name: 391-new#A-Round02_Negative-screen.skyd

Parent ion m/z and charges: 780.3781++

NR_102743.1.1
VVPGSVIDYVIK

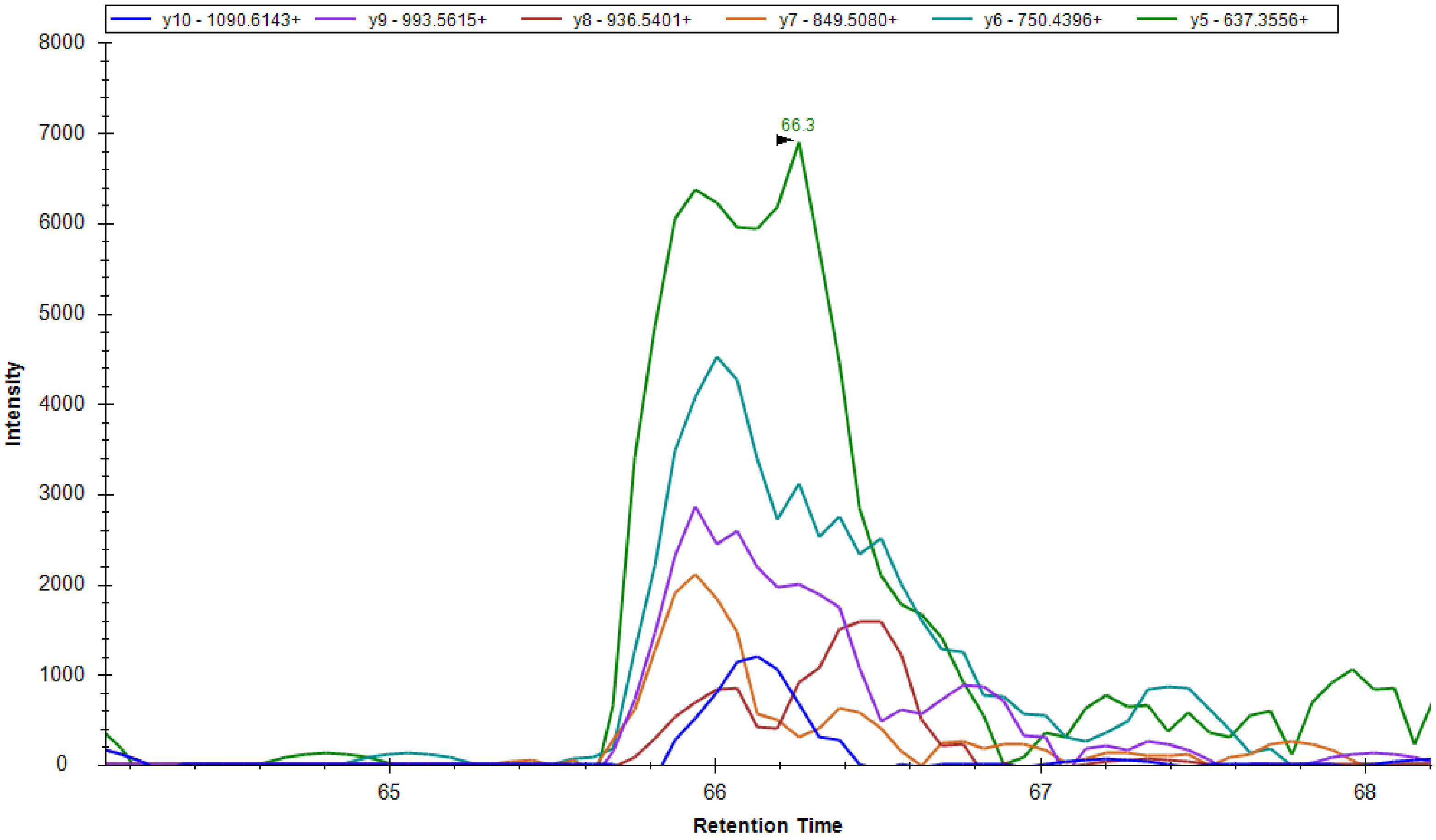

File name: 391-new#A-Round02_Negative-screen.skyd

Parent ion m/z and charges: 644.8792++

NR_103529.1.4
MAWPTVTSSGCSGK

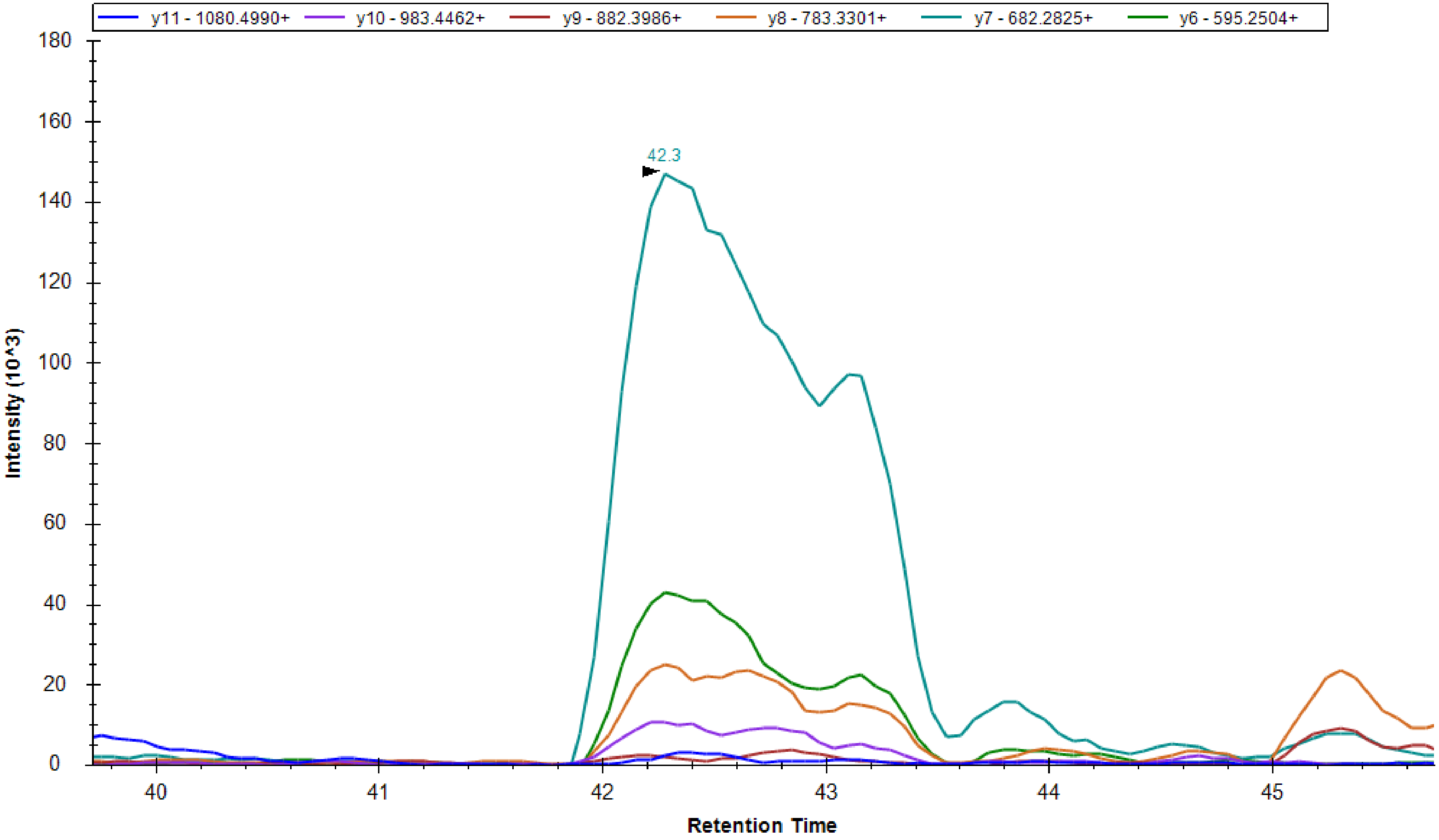

File name: 391-new#A-Round02_Negative-screen.skyd

Parent ion m/z and charges: 734.8316++

NR_103529.1.5
PAETAPPMTPISGHPR

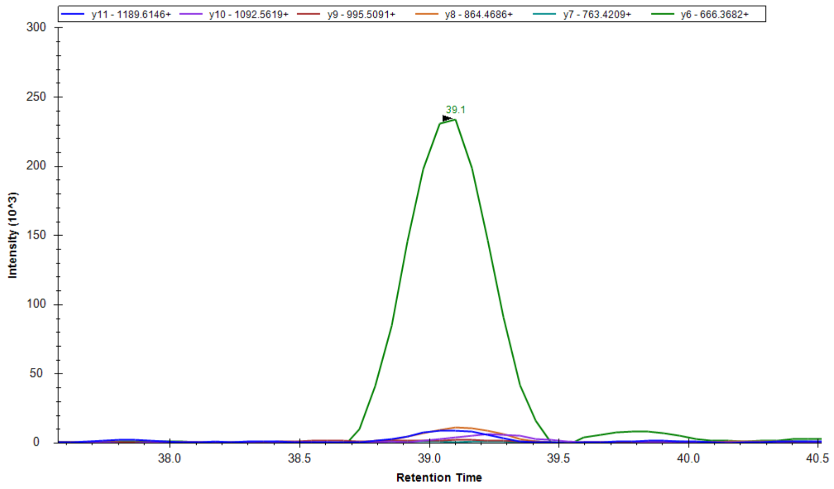

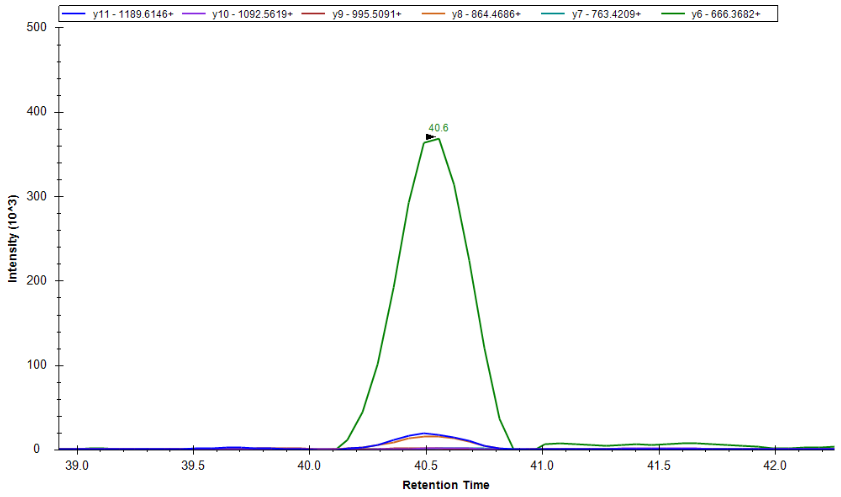

File name: 391-new#A-Round01_All-screening_Positive result.skyd

File name: 391-new#A-Round01_Positive-confirm.skyd

Parent ion m/z and charges: 829.9196++

NR_103558.3.2
TIIHAIHSHR

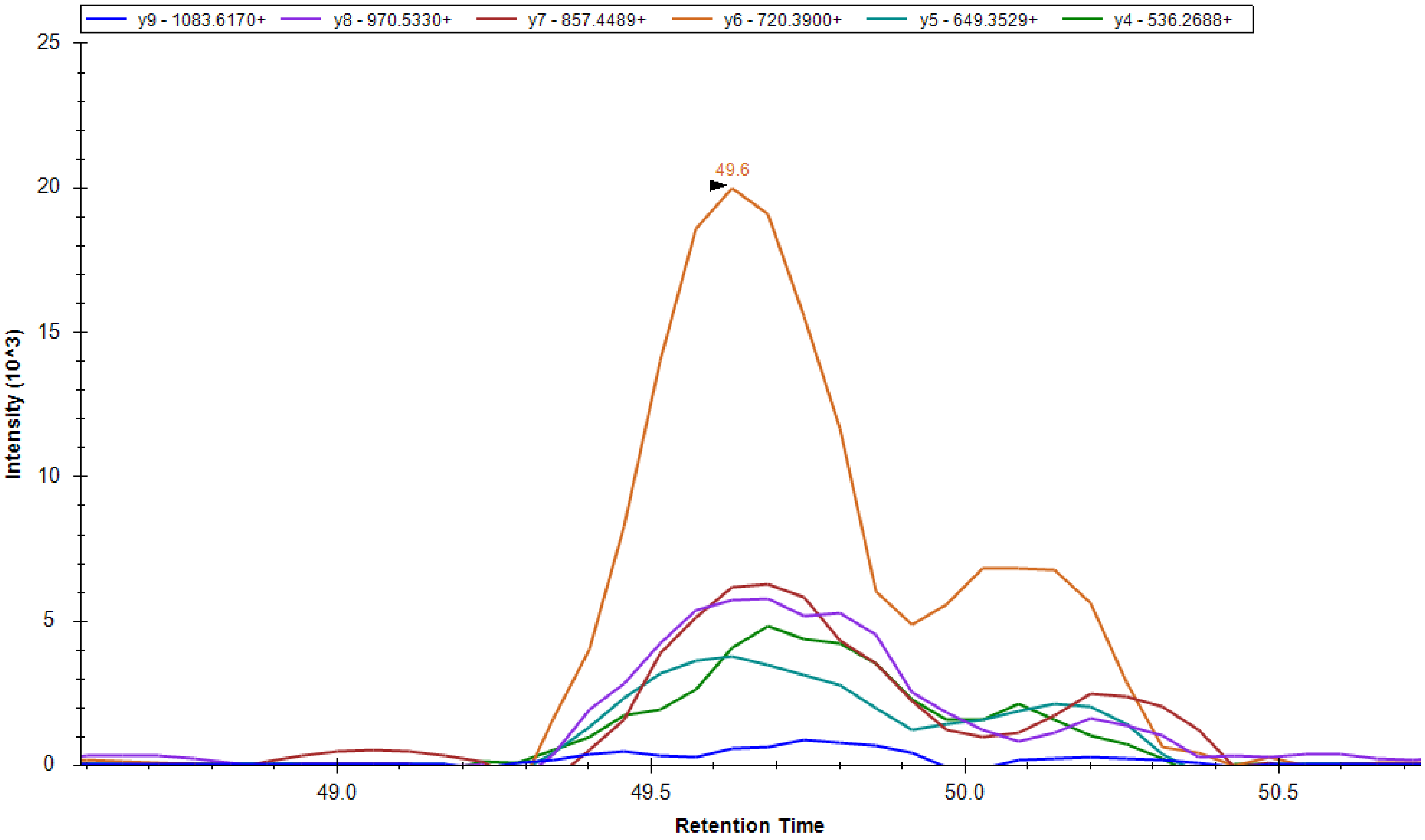

File name: 391-new#B-Round02_Negative-screen.skyd

Parent ion m/z and charges: 592.8360++

NR_103558.3.2
TIIHAIHSHR

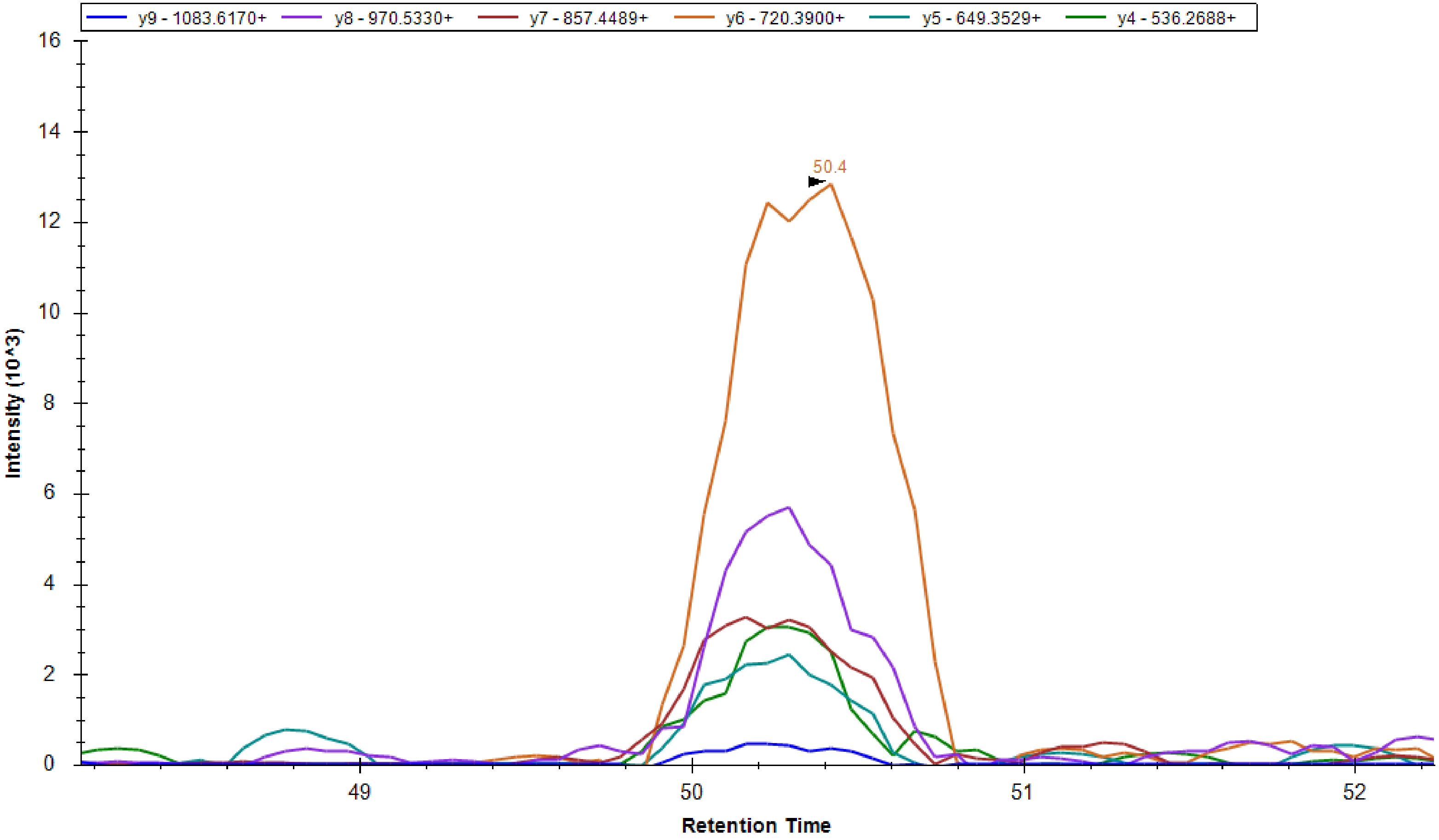

File name: 391-new#A-Round02_Negative-screen.skyd

Parent ion m/z and charges: 592.8360++

NR_103804.1.2
MARMRVASHTSK

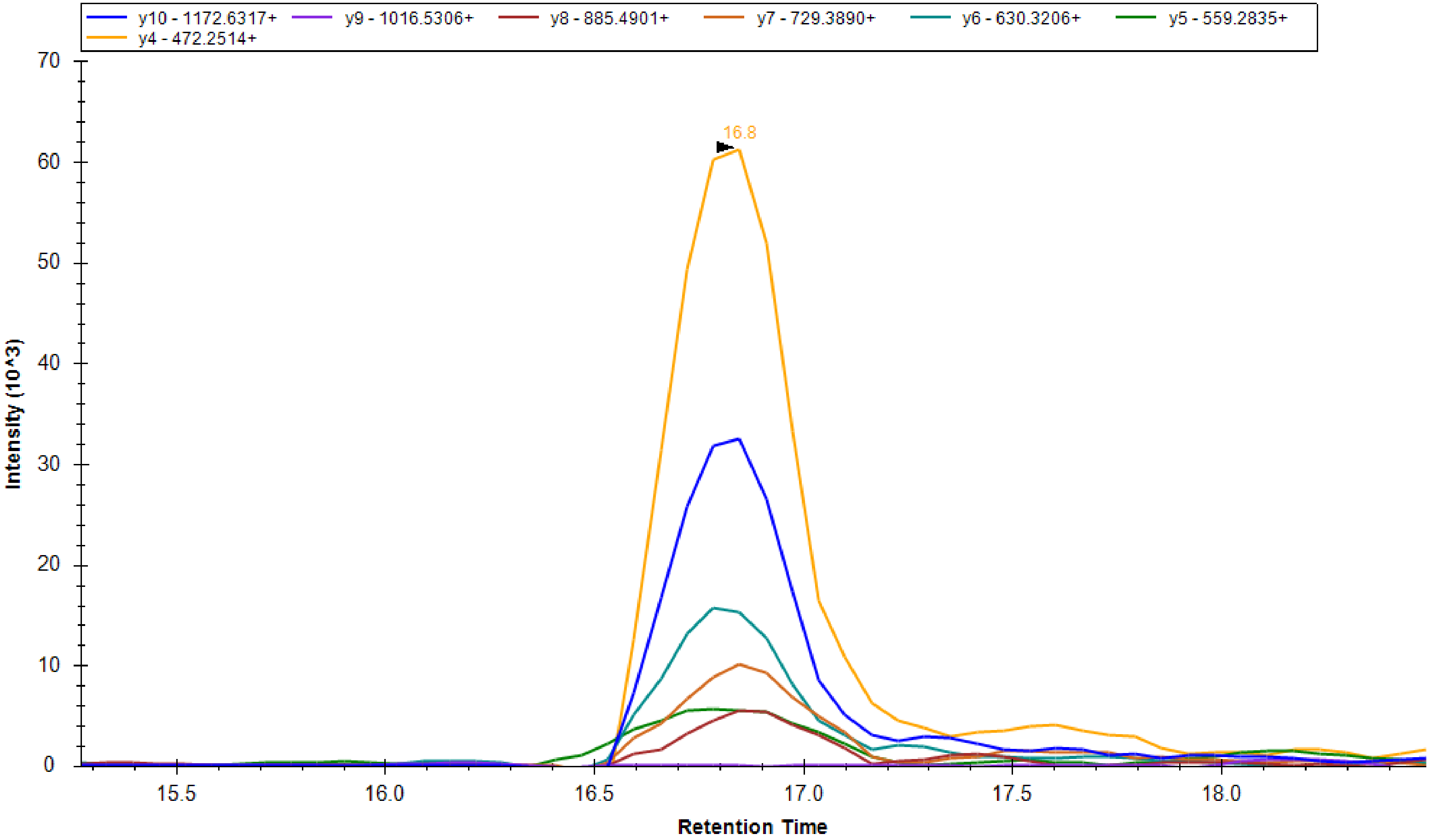

File name: 391-new#A-Round02_Negative-screen.skyd

Parent ion m/z and charges: 687.8583++

NR_103858.2.1
MLPSRPTSDLGSFHGR

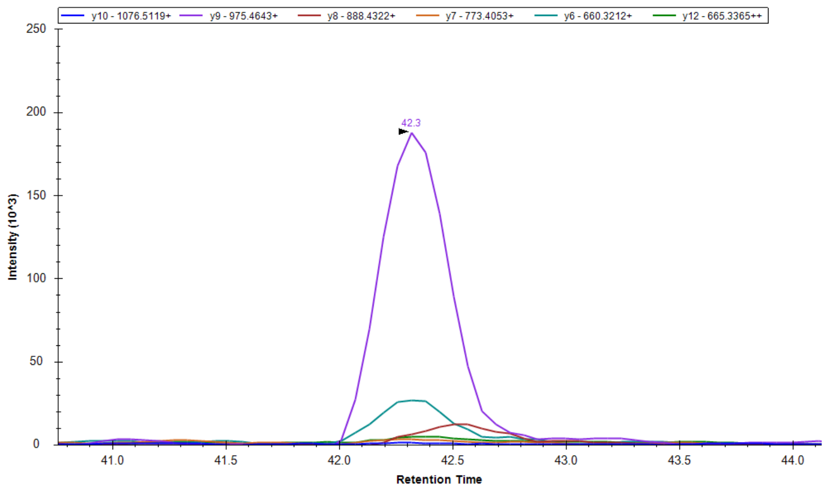

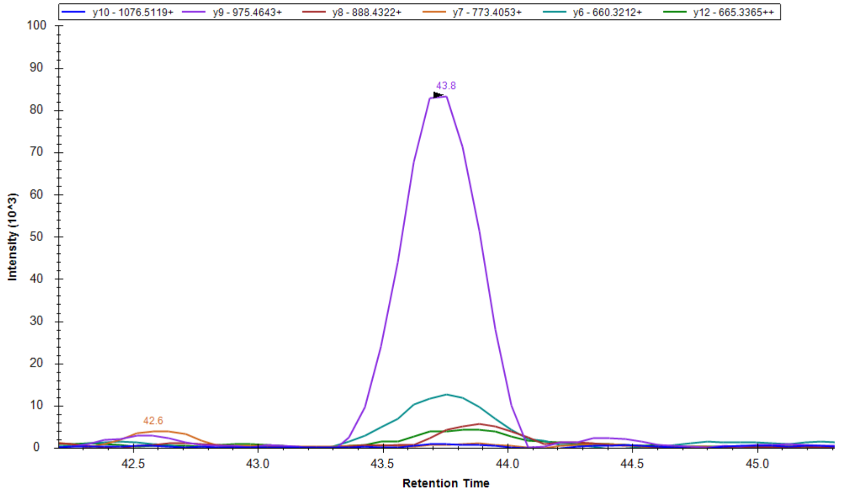

File name: 391-new#A-Round01_All-screening_Positive result.skyd

File name: 391-new#A-Round01_Positive-confirm.skyd

Parent ion m/z and charges: 879.4412++

NR_103858.2.1
MLPSRPTSDLGSFHGR

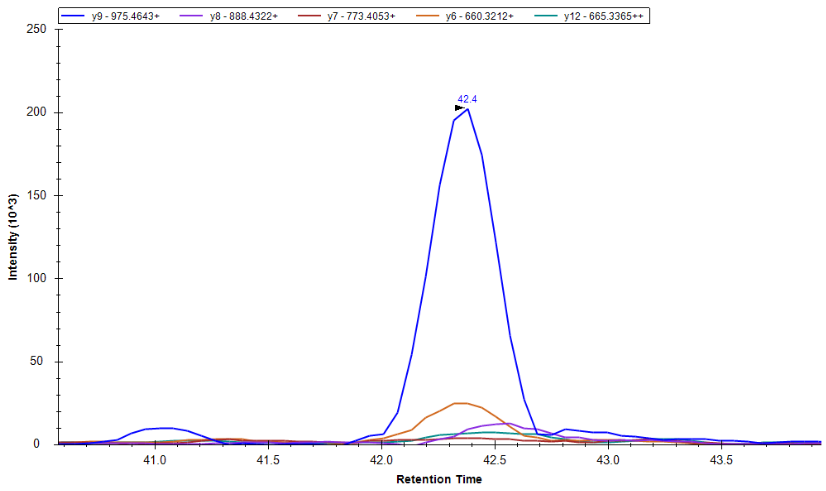

File name: 391-new#B-Round01_All-screening_Positive result.skyd

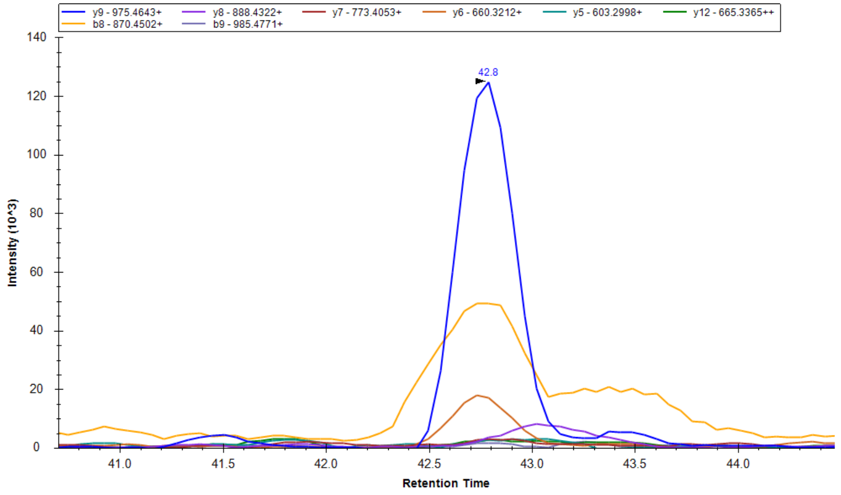

File name: 391-new#B-Round01_Positive-confirm.skyd

Parent ion m/z and charges: 879.4412++

NR_104148.2.1
MPRICHNTHIMIK

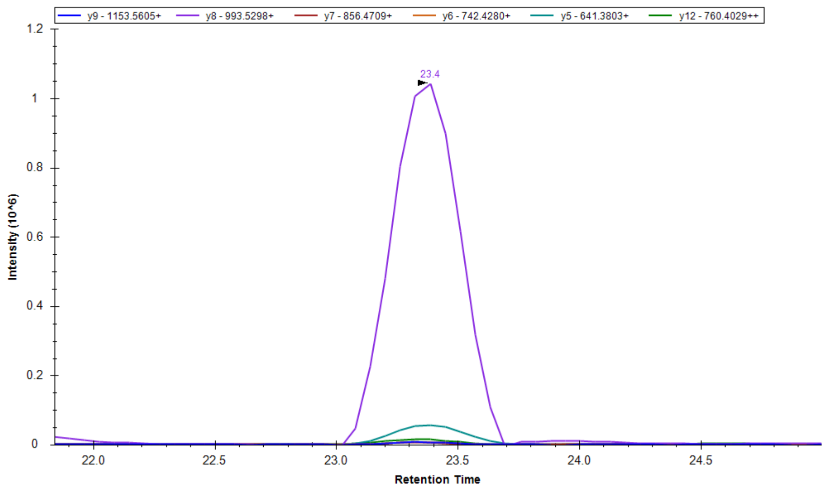

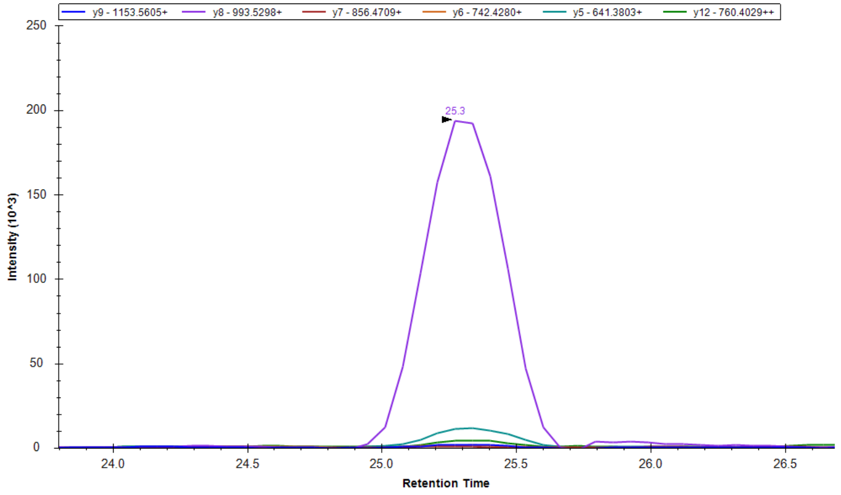

File name: 391-new#A-Round01_All-screening_Positive result.skyd

File name: 391-new#A-Round01_Positive-confirm.skyd

Parent ion m/z and charges: 825.9231++

NR_104148.2.1
MPRICHNTHIMIK

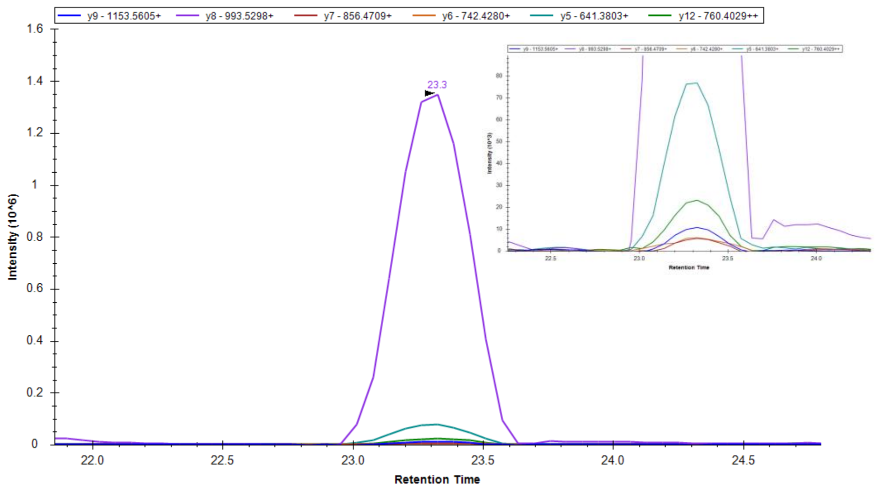

File name: 391-new#B-Round01_All-screening_Positive result.skyd

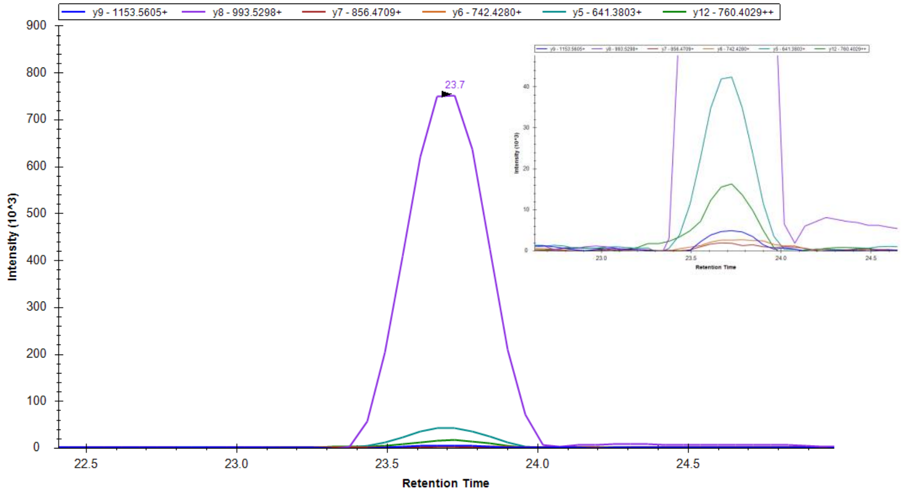

File name: 391-new#B-Round01_Positive-confirm.skyd

Parent ion m/z and charges: 825.9231++

NR_104262.1.2
AITGIDISVCTQWMK

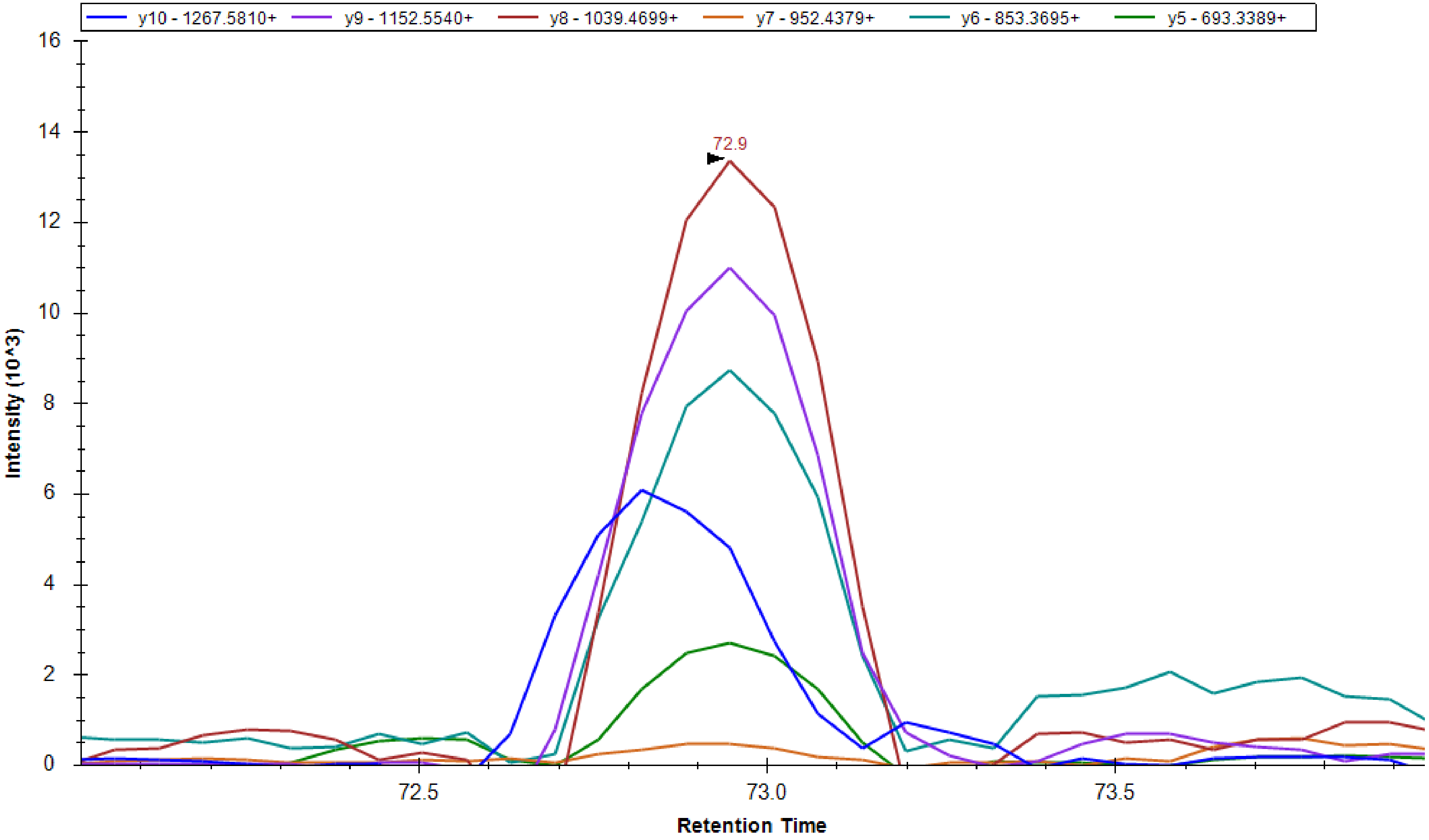

File name: 391-new#A-Round02_Negative-screen.skyd

Parent ion m/z and charges: 861.9313++

NR_104269.1.9
TNTPFSPIQEEVKHMK

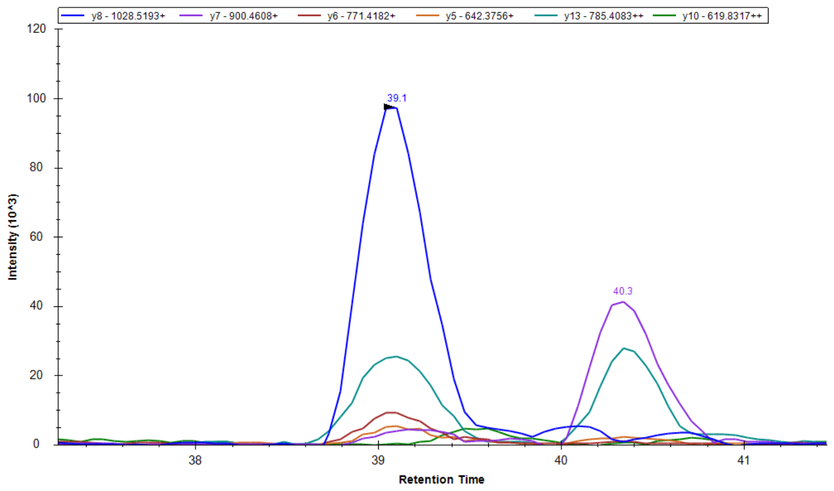

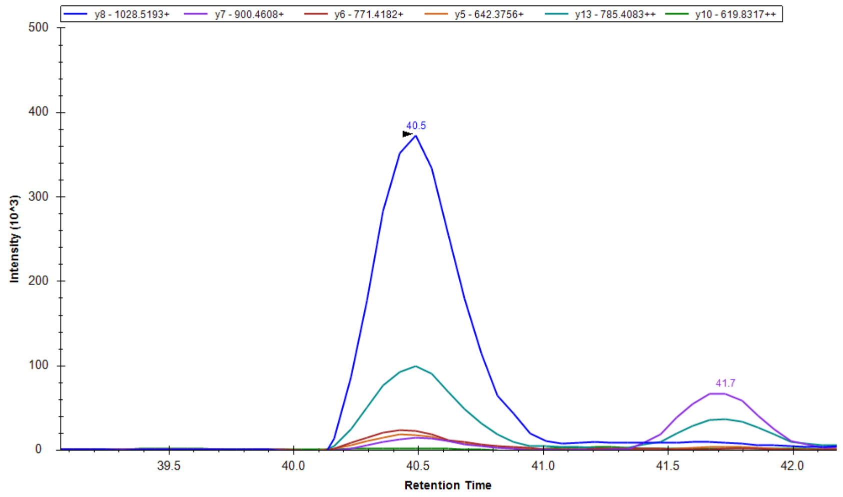

File name: 391-new#A-Round01_All-screening_Positive result.skyd

File name: 391-new#A-Round01_Positive-confirm.skyd

Parent ion m/z and charges: 629.3207+++

NR_104269.1.9
TNTPFSPIQEEVKHMK

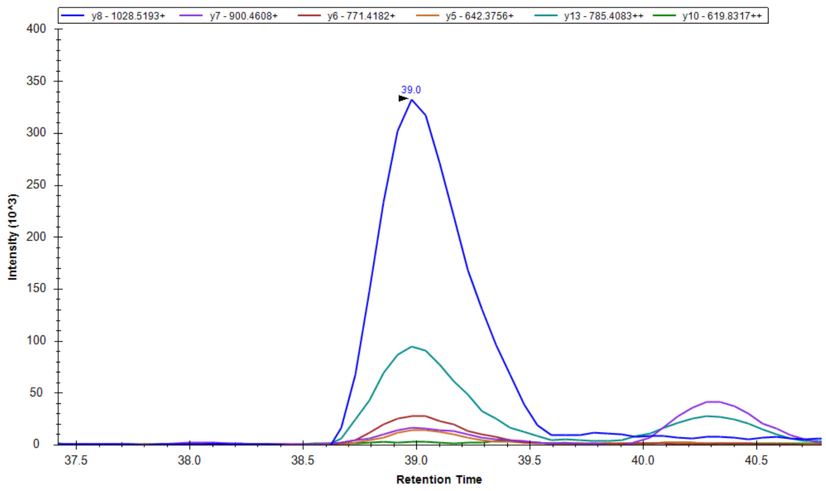

File name: 391-new#B-Round01_All-screening_Positive result.skyd

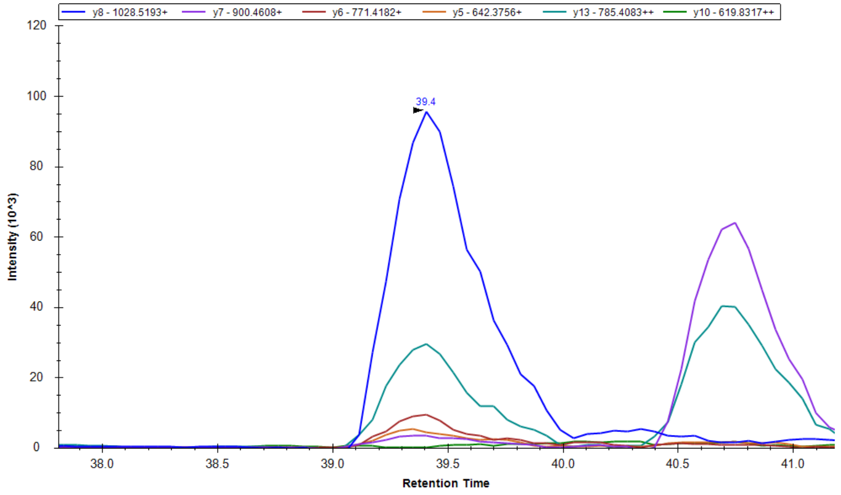

File name: 391-new#B-Round01_Positive-confirm.skyd

Parent ion m/z and charges: 629.3207+++

NR_104286.2.7
KMEPESLDIR

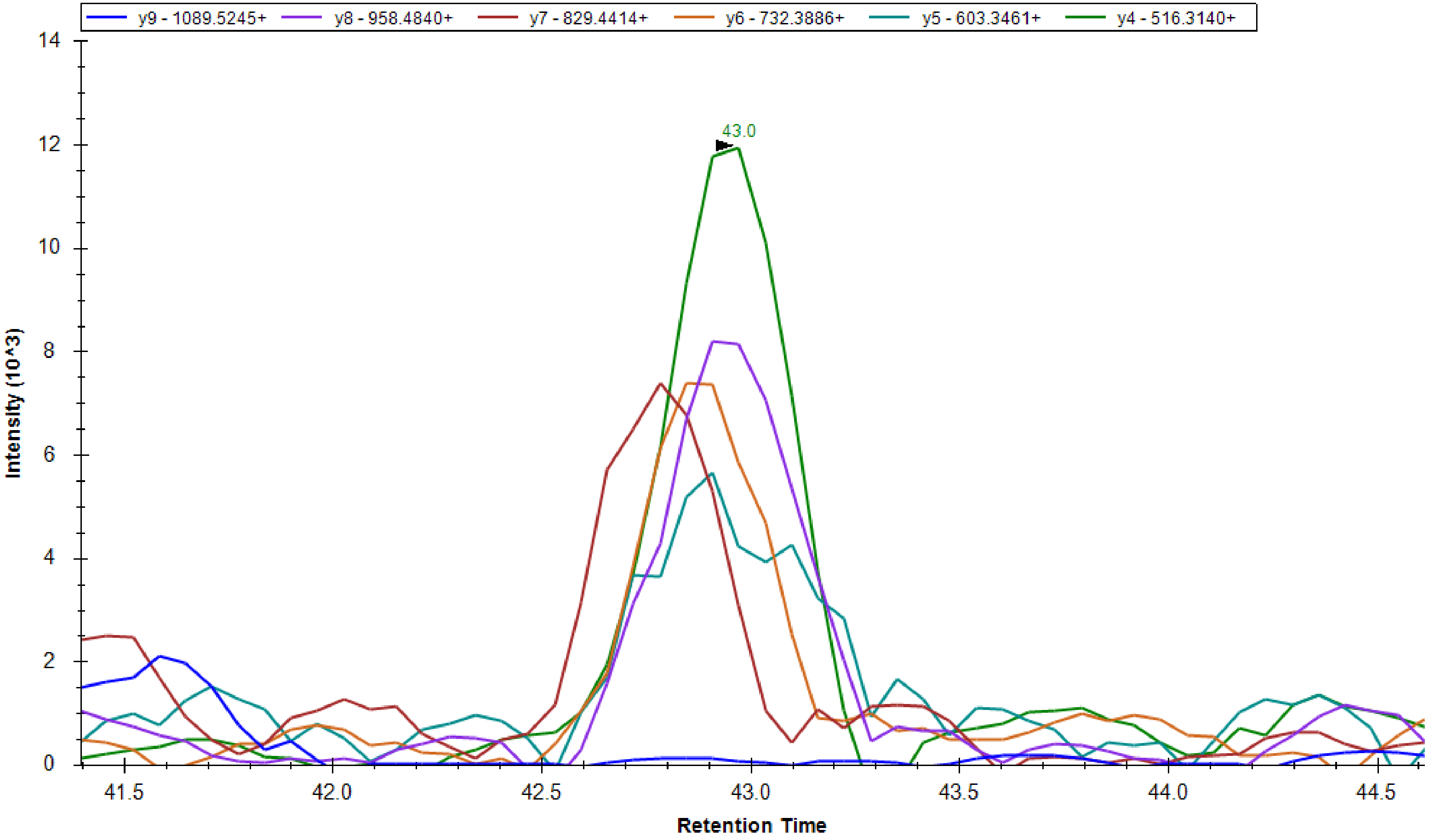

File name: 391-new#A-Round02_Negative-screen.skyd

Parent ion m/z and charges: 609.3134++

NR_104292.1.9
IHDPWNAGR

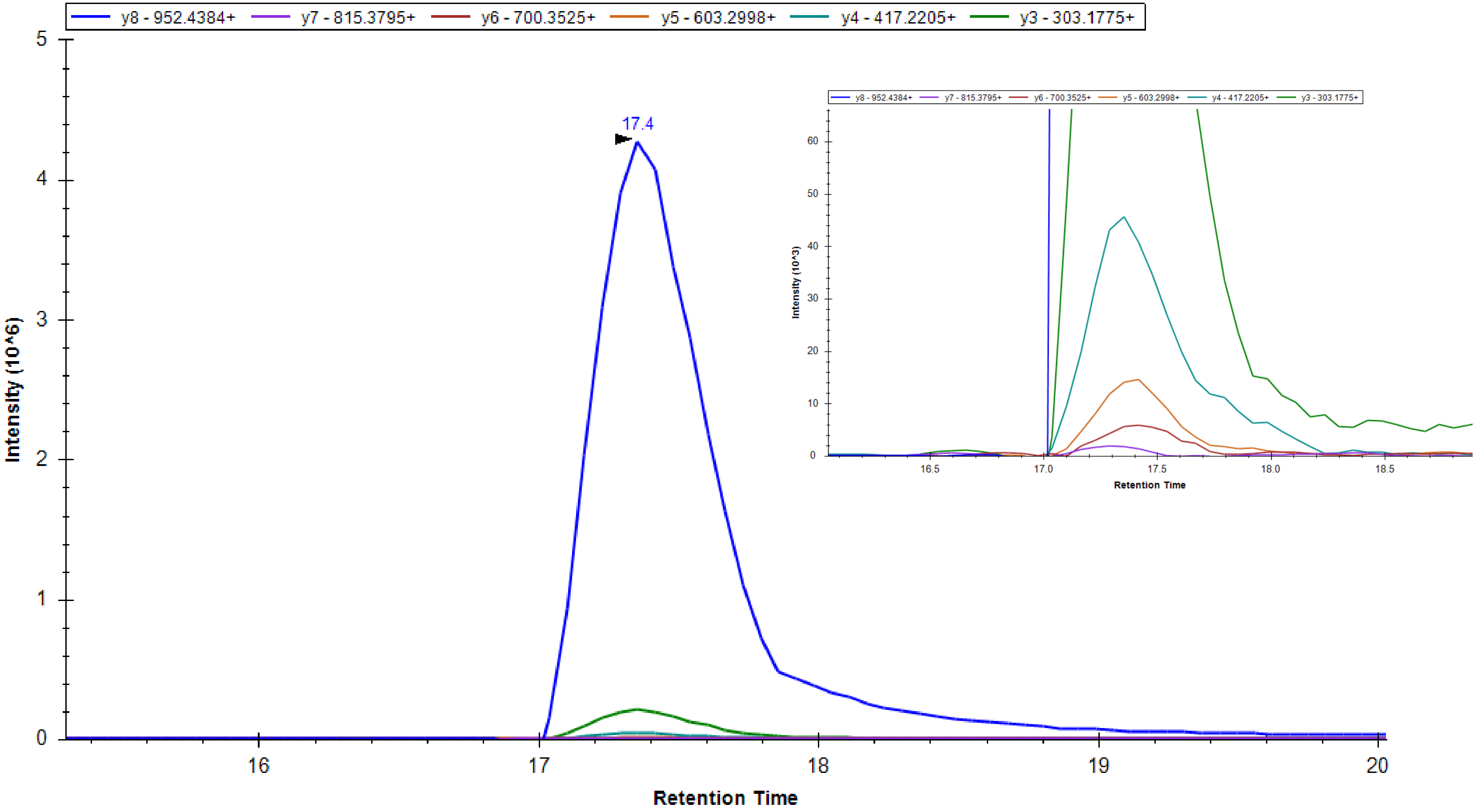

File name: 391-new#A-Round02_Negative-screen.skyd

Parent ion m/z and charges: 533.2649++

NR_105014.1.1
SVPHATSPR

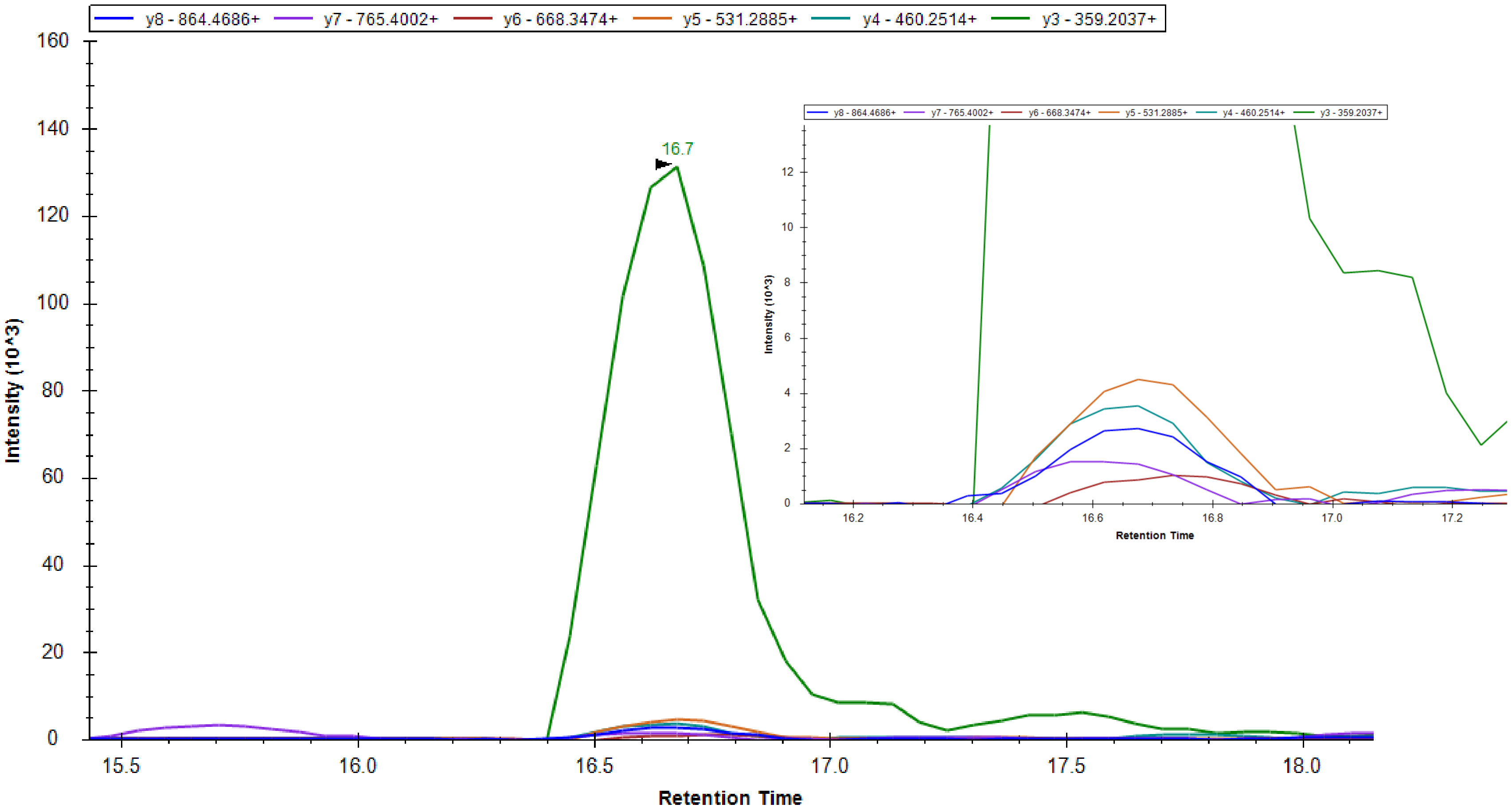

File name: 391-new#B-Round02_Negative-screen.skyd

Parent ion m/z and charges: 476.2540++

NR_105014.1.1
SVPHATSPR

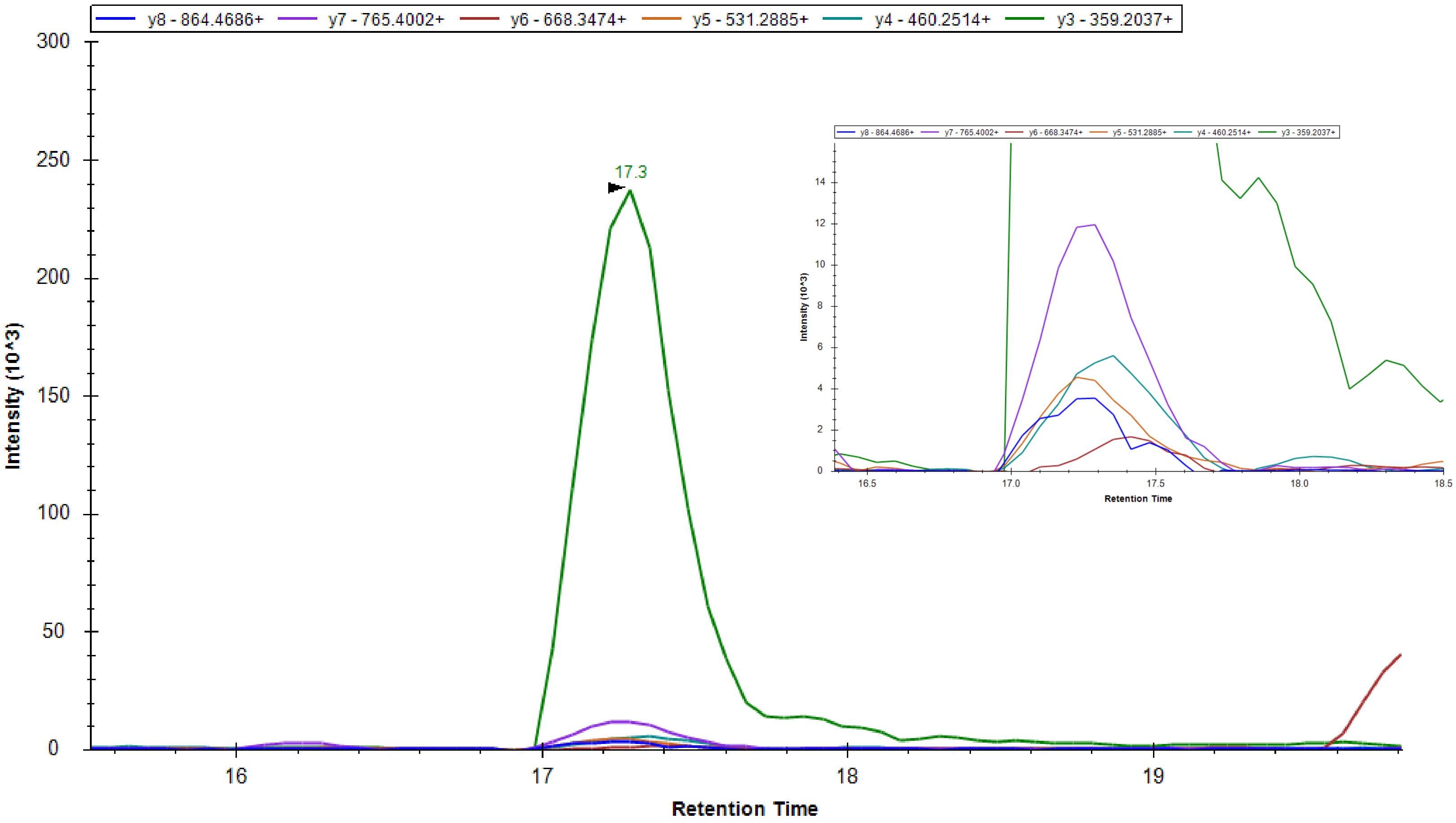

File name: 391-new#A-Round02_Negative-screen.skyd

Parent ion m/z and charges: 476.2540++

NR_105018.1.3
INSANNIDEPGQKPEPR

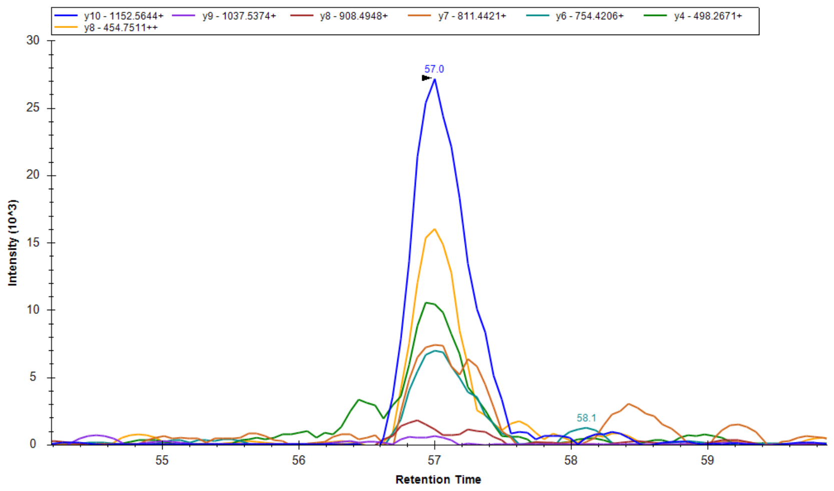

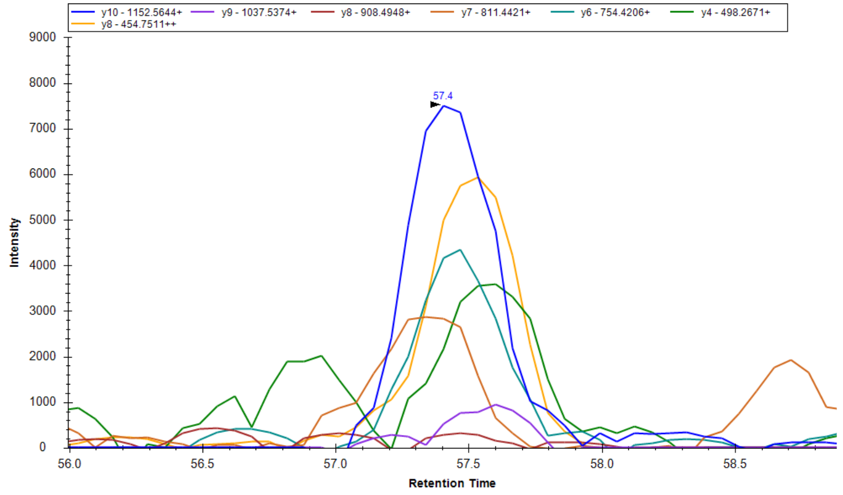

File name: 391-new#A-Round01_All-screening_Positive result.skyd

File name: 391-new#A-Round01_Positive-confirm.skyd

Parent ion m/z and charges: 626.9817+++

NR_105018.1.3
INSANNIDEPGQKPEPR

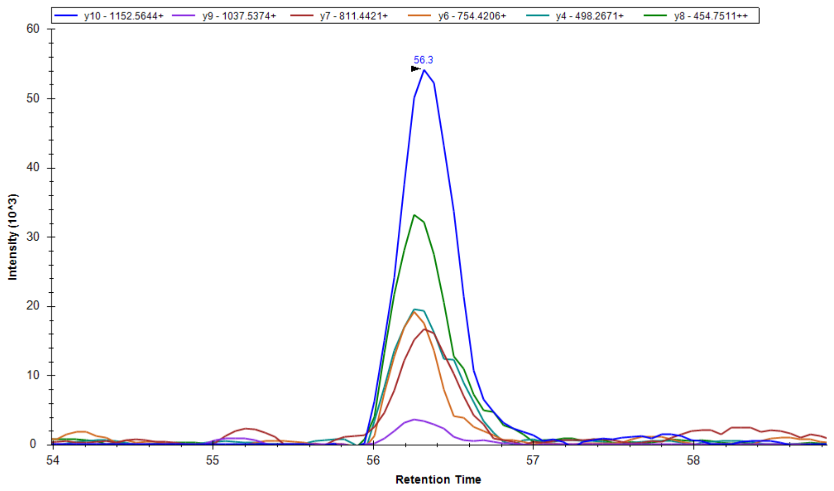

File name: 391-new#B-Round01_All-screening_Positive result.skyd

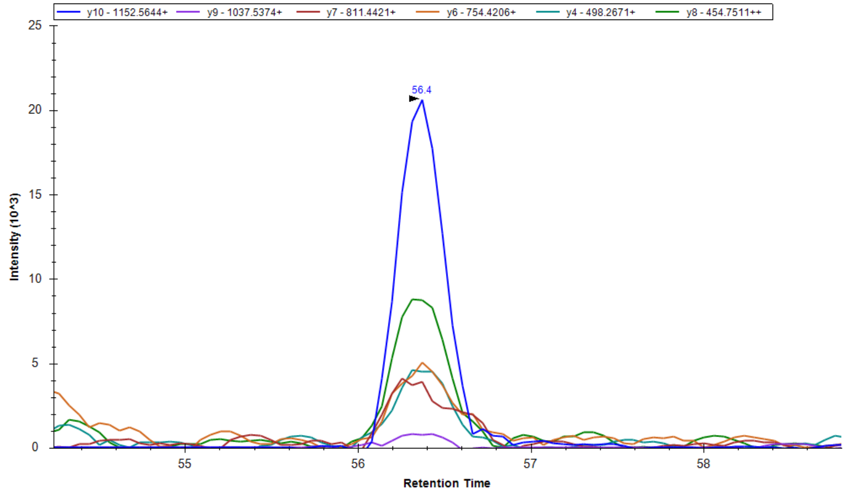

File name: 391-new#B-Round01_Positive-confirm.skyd

Parent ion m/z and charges: 626.9817+++

NR_105045.3.2
GLGGSMALAAPQFGMSIFR

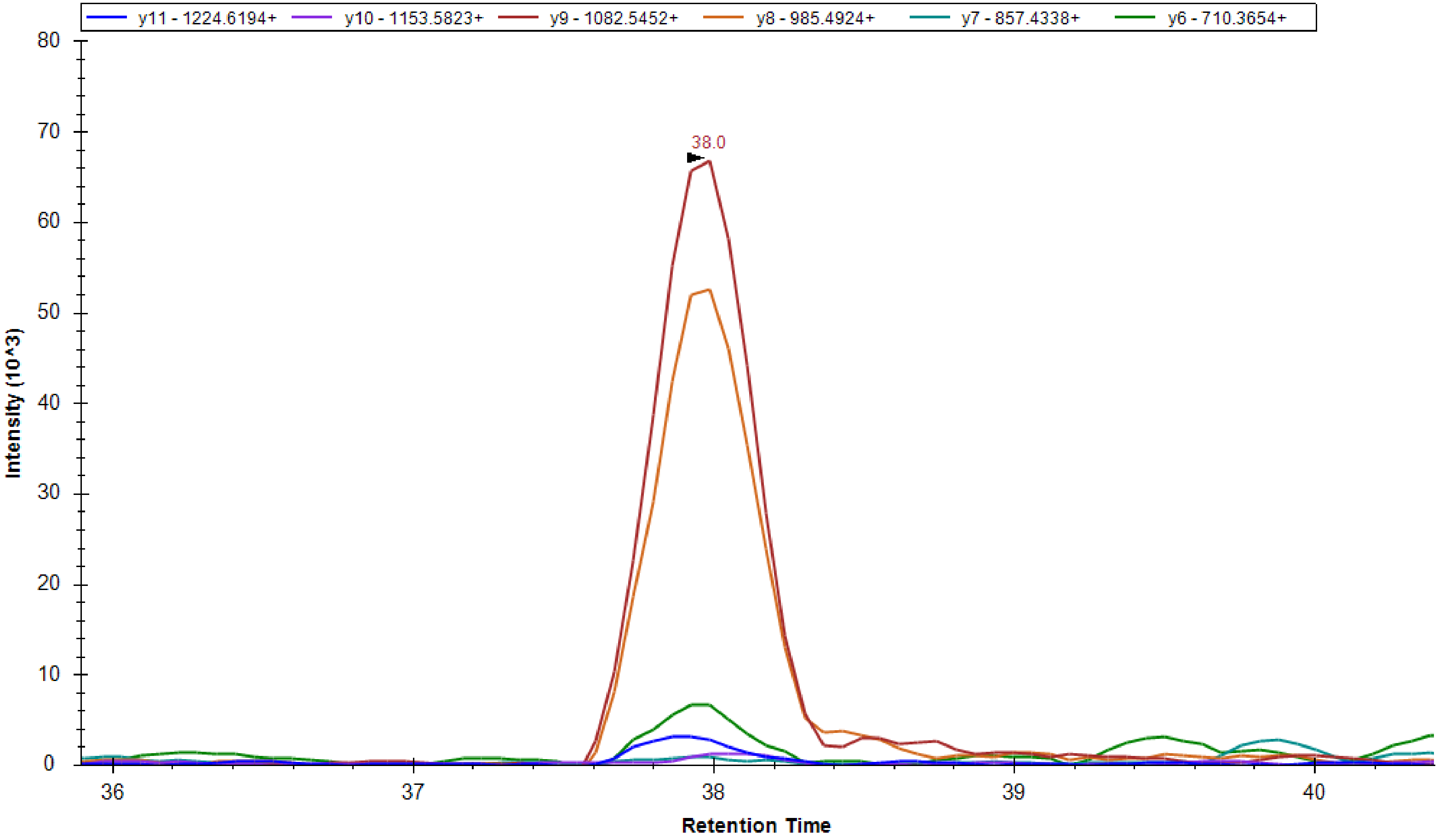

File name: 391-new#A-Round02_Negative-screen.skyd

Parent ion m/z and charges: 955.9844++

NR_108066.2.3
MNTFIIIIIR

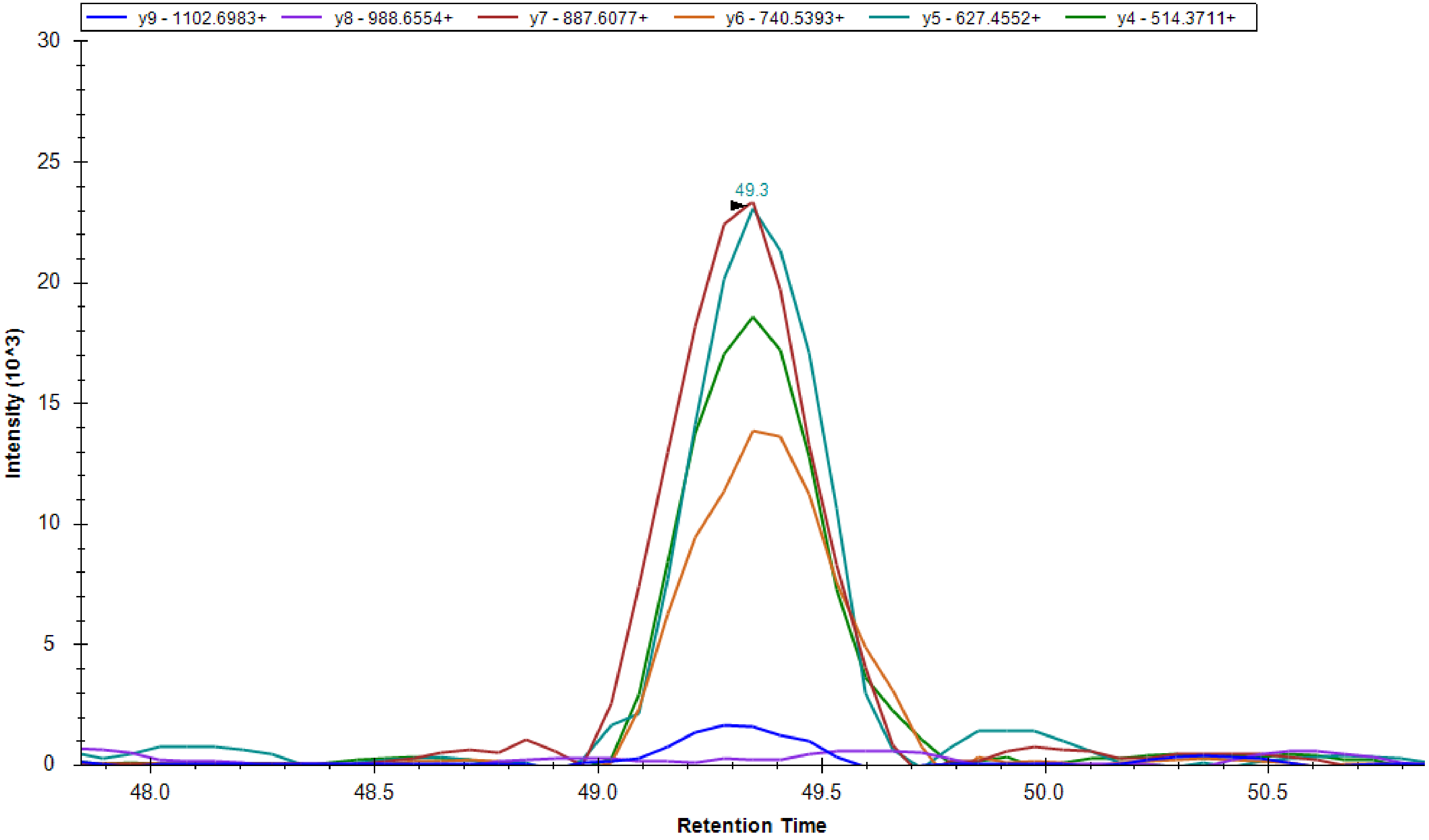

File name: 391-new#A-Round02_Negative-screen.skyd

Parent ion m/z and charges: 617.3730++

NR_109783.2.2
RDPSPGGALGQSPR

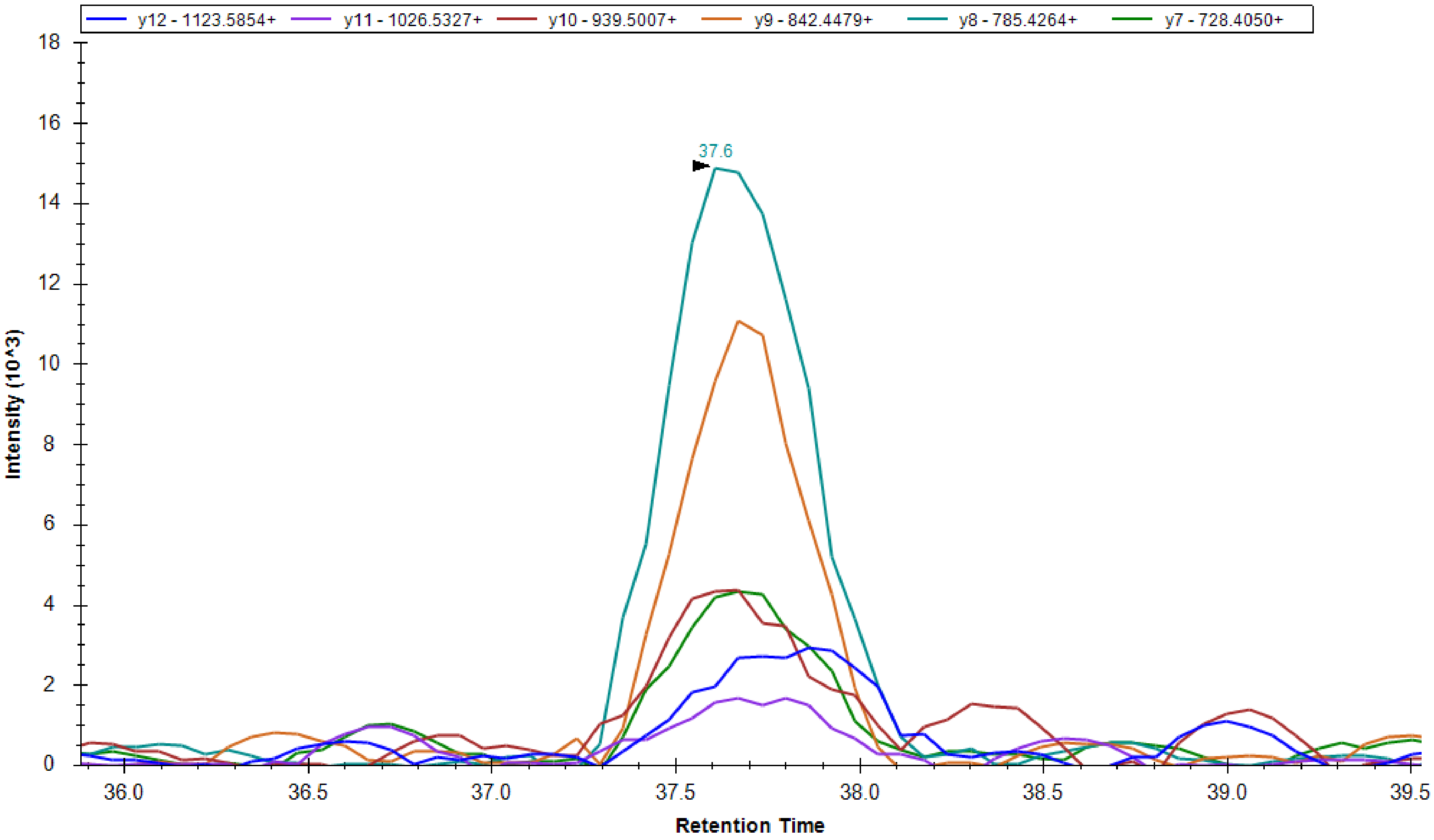

File name: 391-new#A-Round02_Negative-screen.skyd

Parent ion m/z and charges: 697.8604++

NR_110229.1.10
NIYSMPQGHMPPIK

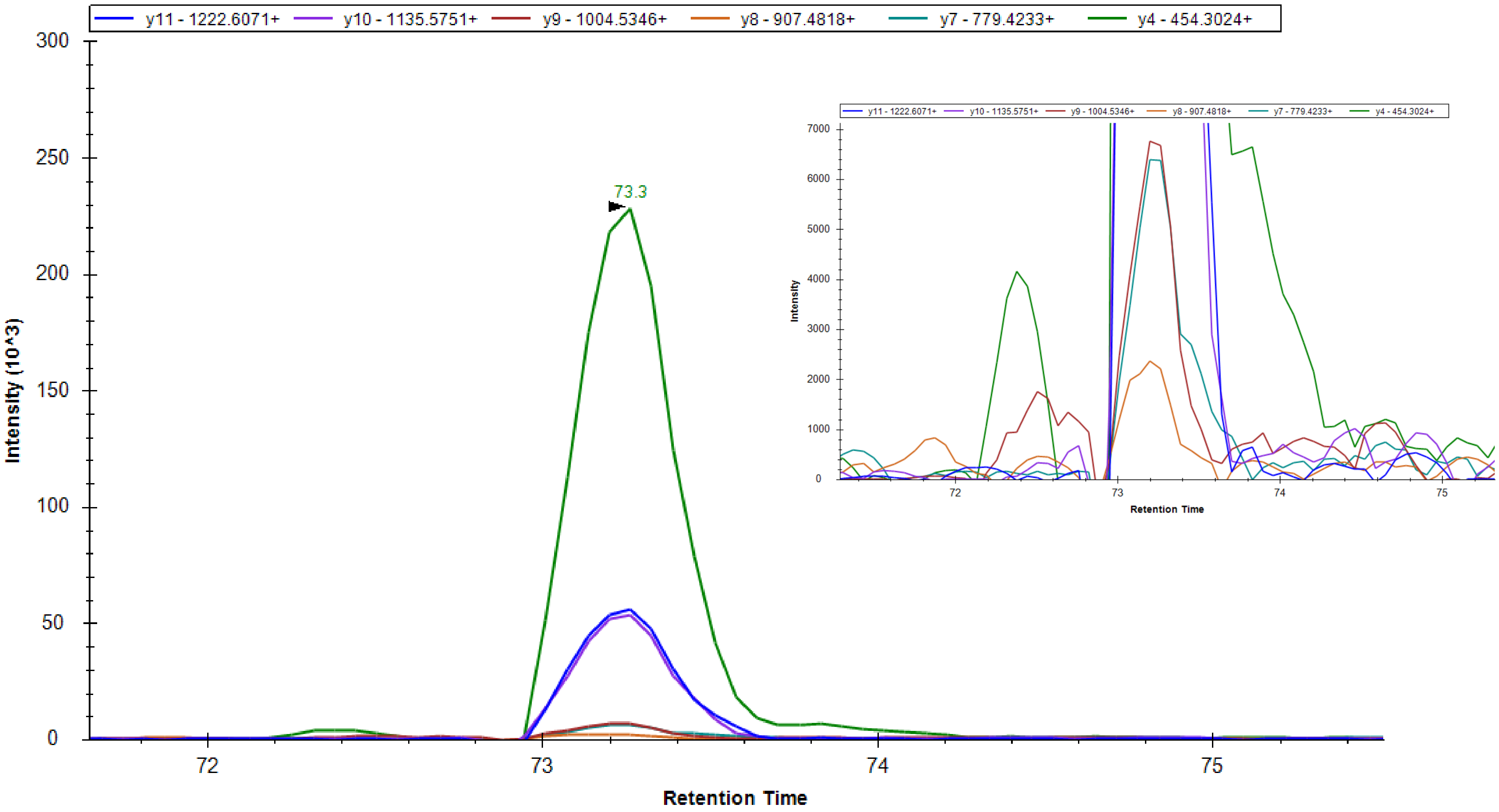

File name: 391-new#A-Round02_Negative-screen.skyd

Parent ion m/z and charges: 806.9024++

NR_110257.3.20
MGVHISLQD

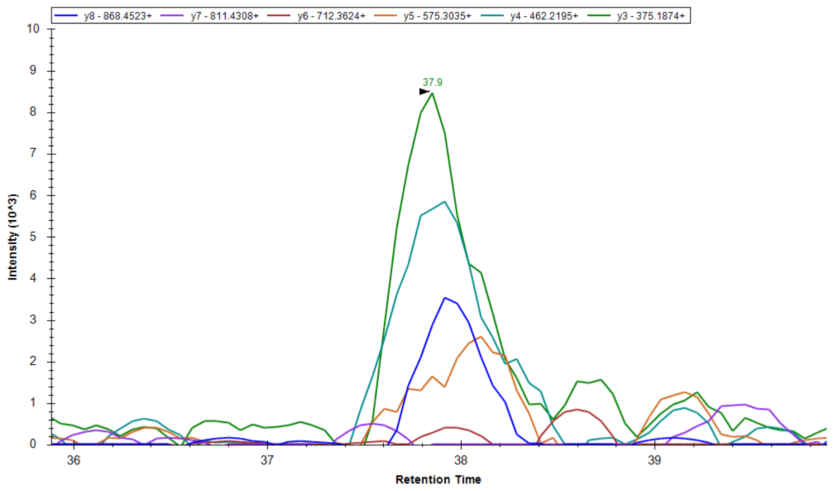

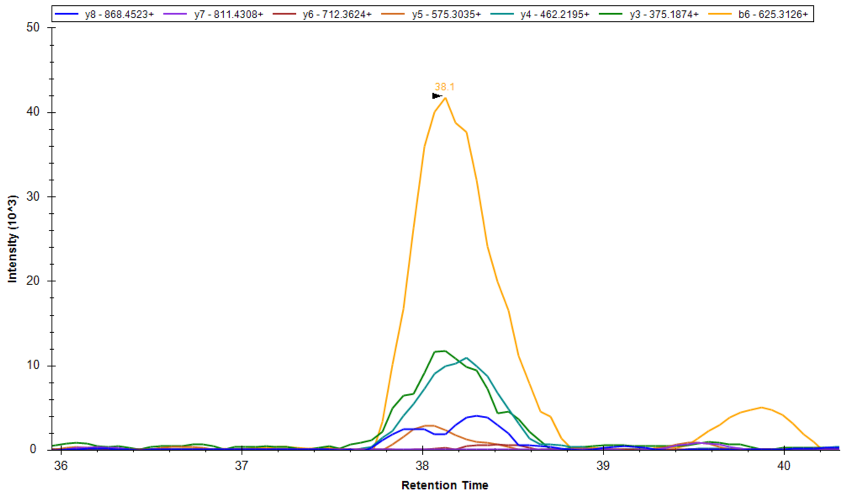

File name: 391-new#B-Round01_All-screening_Positive result.skyd

File name: 391-new#B-Round01_Positive-confirm.skyd

Parent ion m/z and charges: 500.2500++

NR_110303.3.2
HPHLPNSALPFGAPGPSNR

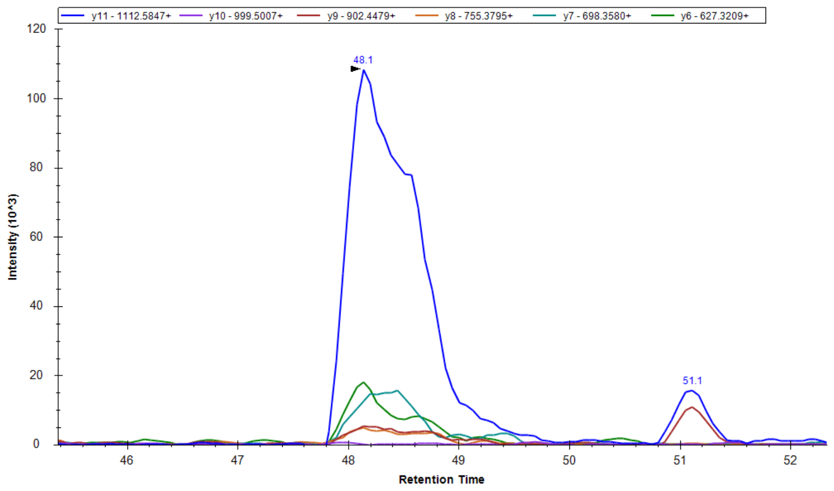

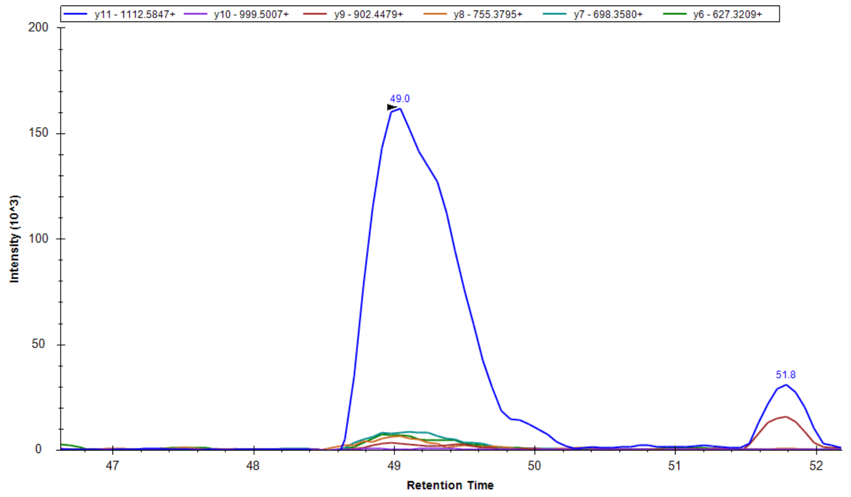

File name: 391-new#A-Round01_All-screening_Positive result.skyd

File name: 391-new#A-Round01_Positive-confirm.skyd

Parent ion m/z and charges: 656.0063+++

NR_110303.3.2
HPHLPNSALPFGAPGPSNR

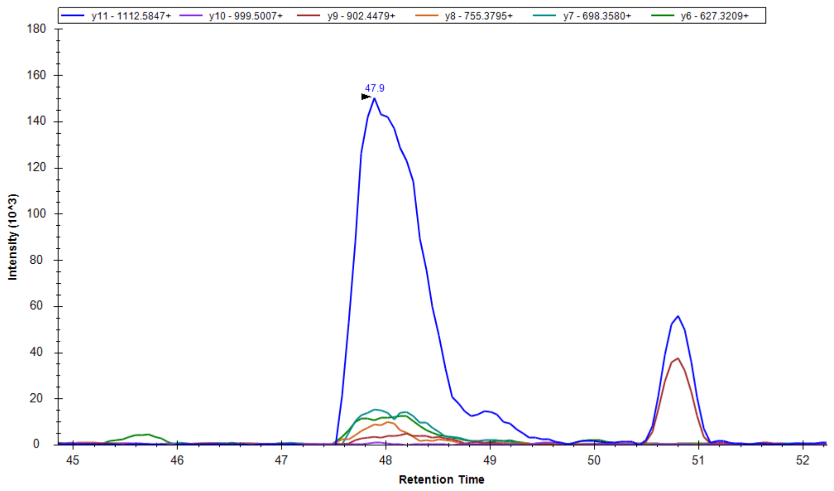

File name: 391-new#B-Round01_All-screening_Positive result.skyd

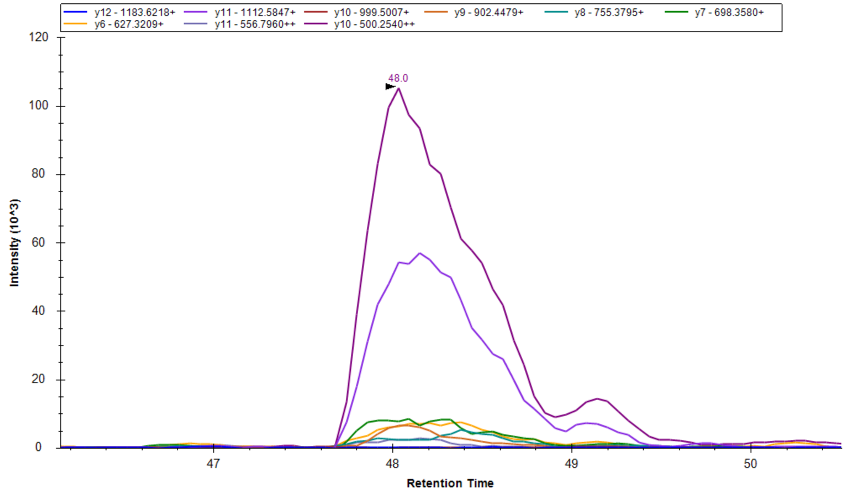

File name: 391-new#B-Round01_Positive-confirm.skyd

Parent ion m/z and charges: 656.0063+++

NR_110439.2.4
YINQELSLKILLK

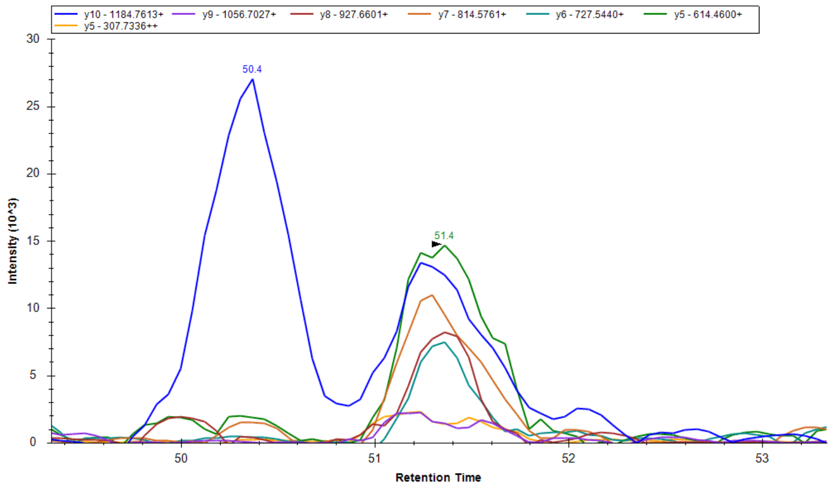

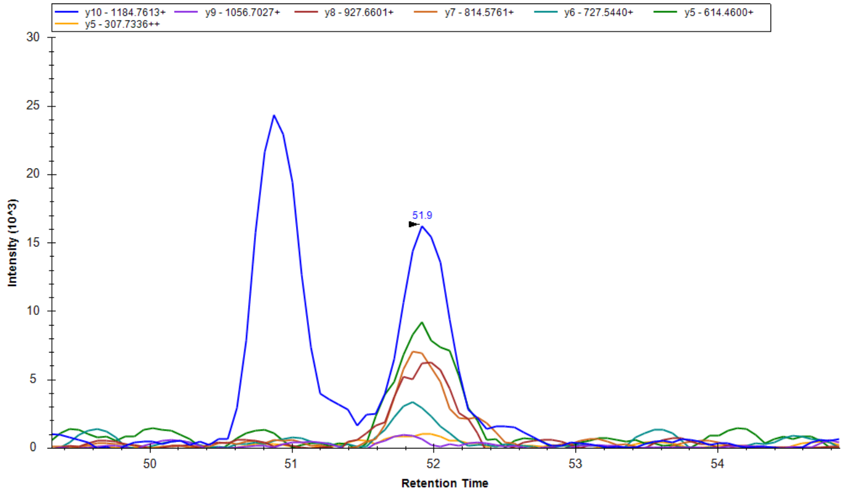

File name: 391-new#A-Round01_All-screening_Positive result.skyd

File name: 391-new#A-Round01_Positive-confirm.skyd

Parent ion m/z and charges: 787.9794++

NR_110439.2.4
YINQELSLKILLK

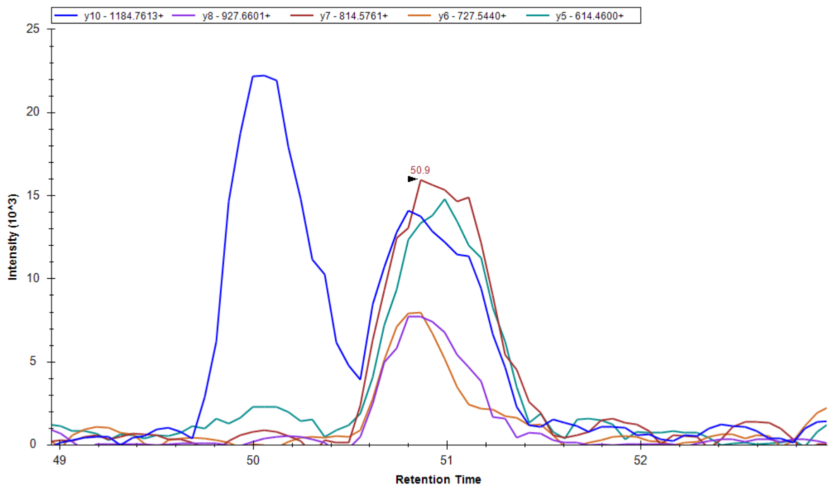

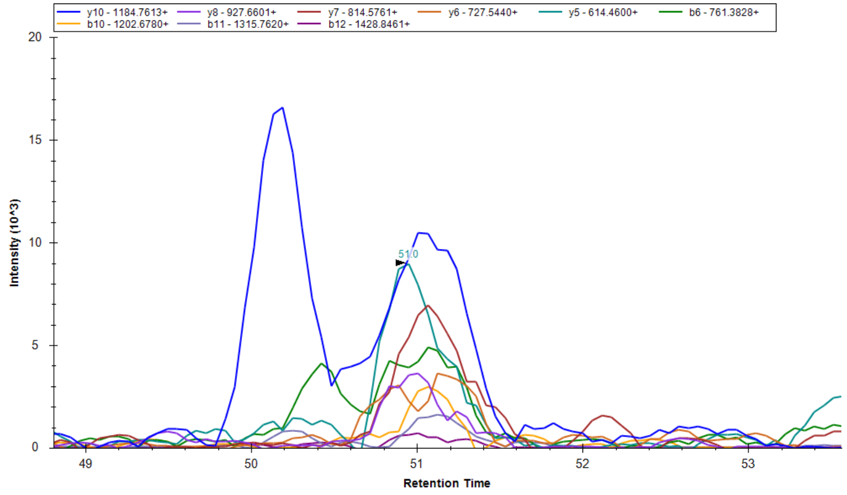

File name: 391-new#B-Round01_All-screening_Positive result.skyd

File name: 391-new#B-Round01_Positive-confirm.skyd

Parent ion m/z and charges: 787.9794++

NR_110562.3.1
DRGHSALLSPR

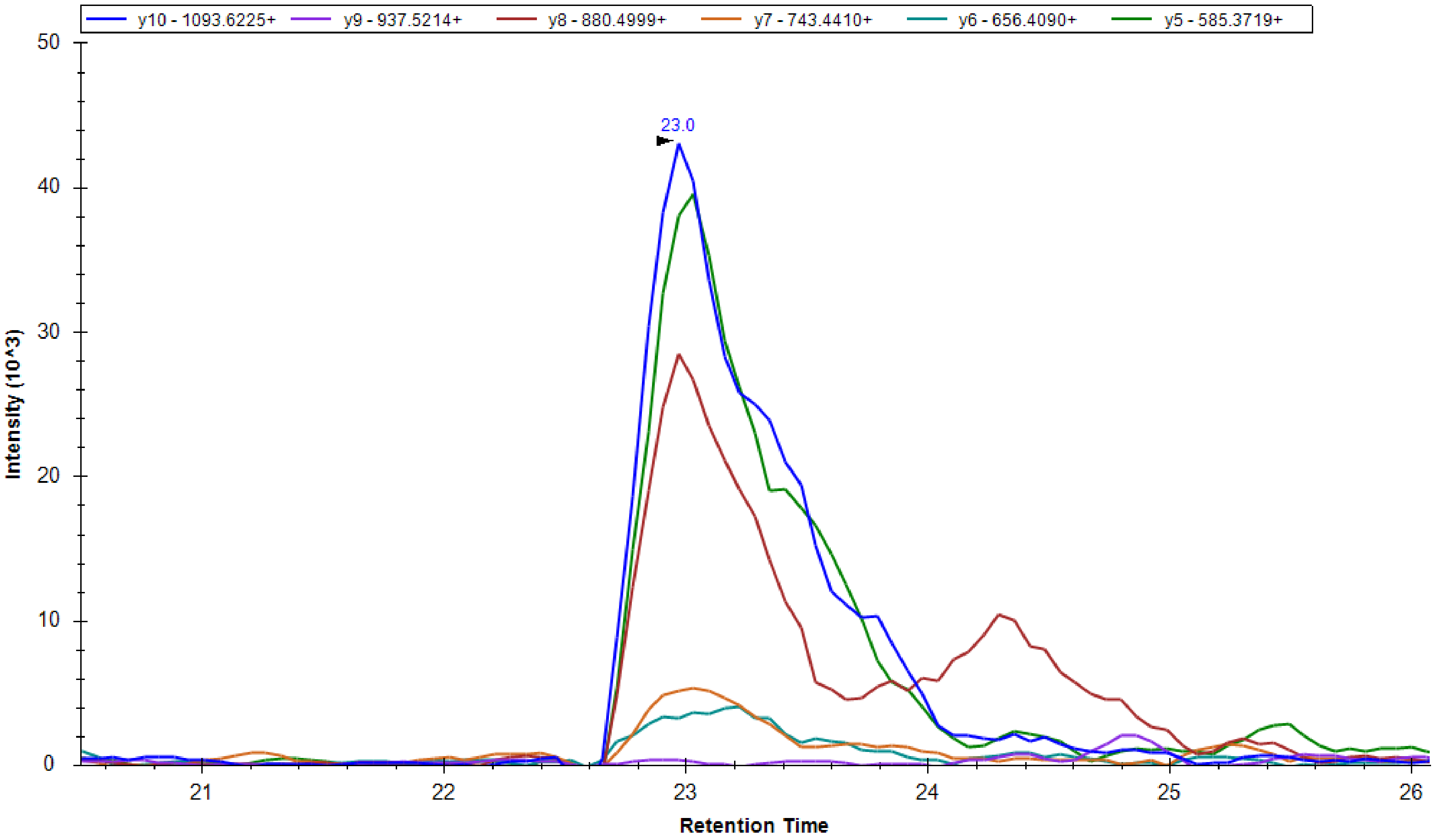

File name: 391-new#A-Round02_Negative-screen.skyd

Parent ion m/z and charges: 604.8284++

NR_110562.3.1
DRGHSALLSPR

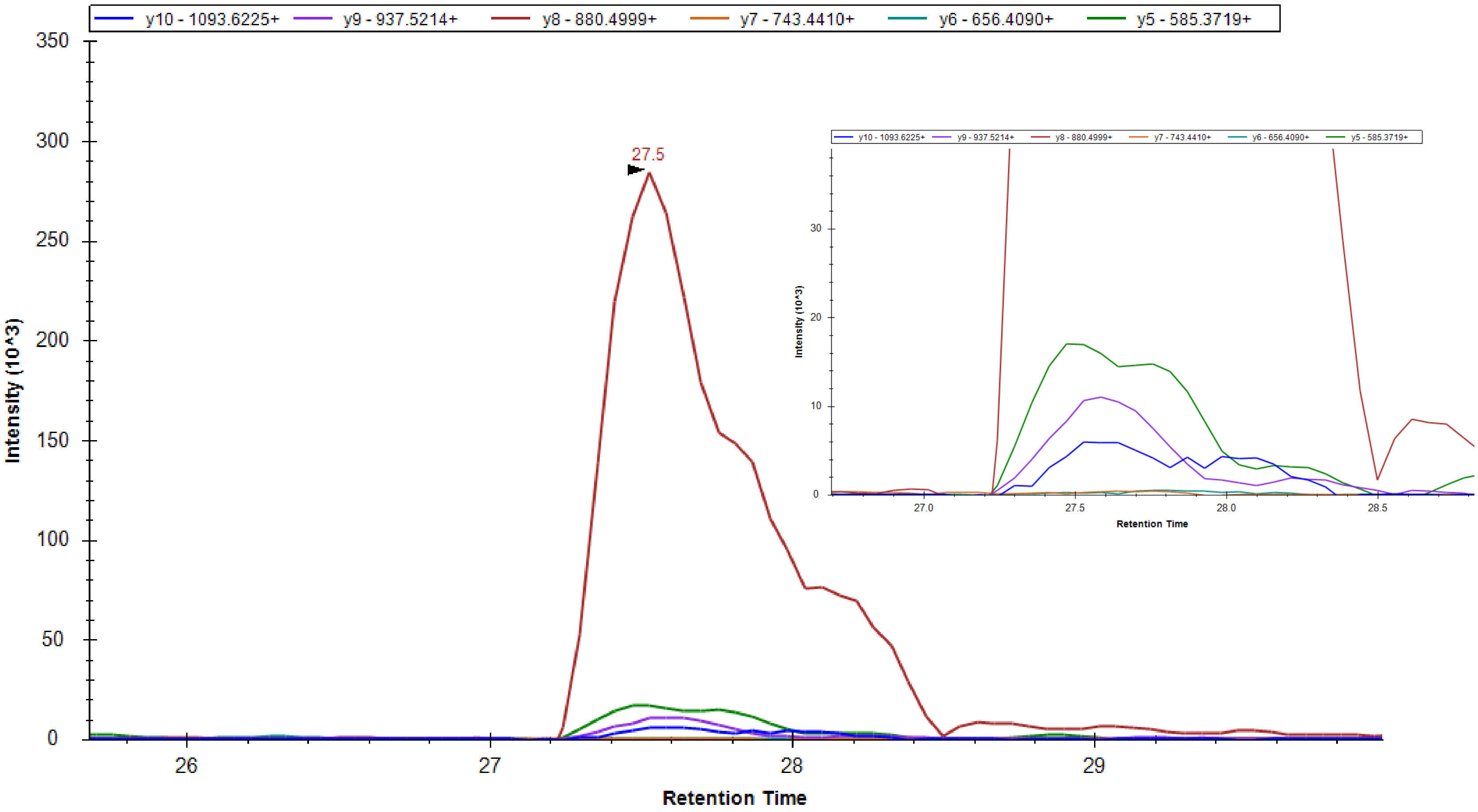

File name: 391-new#B-Round02_Negative-screen.skyd

Parent ion m/z and charges: 604.8284++

NR_110611.3.1
VMEMPIHGIR

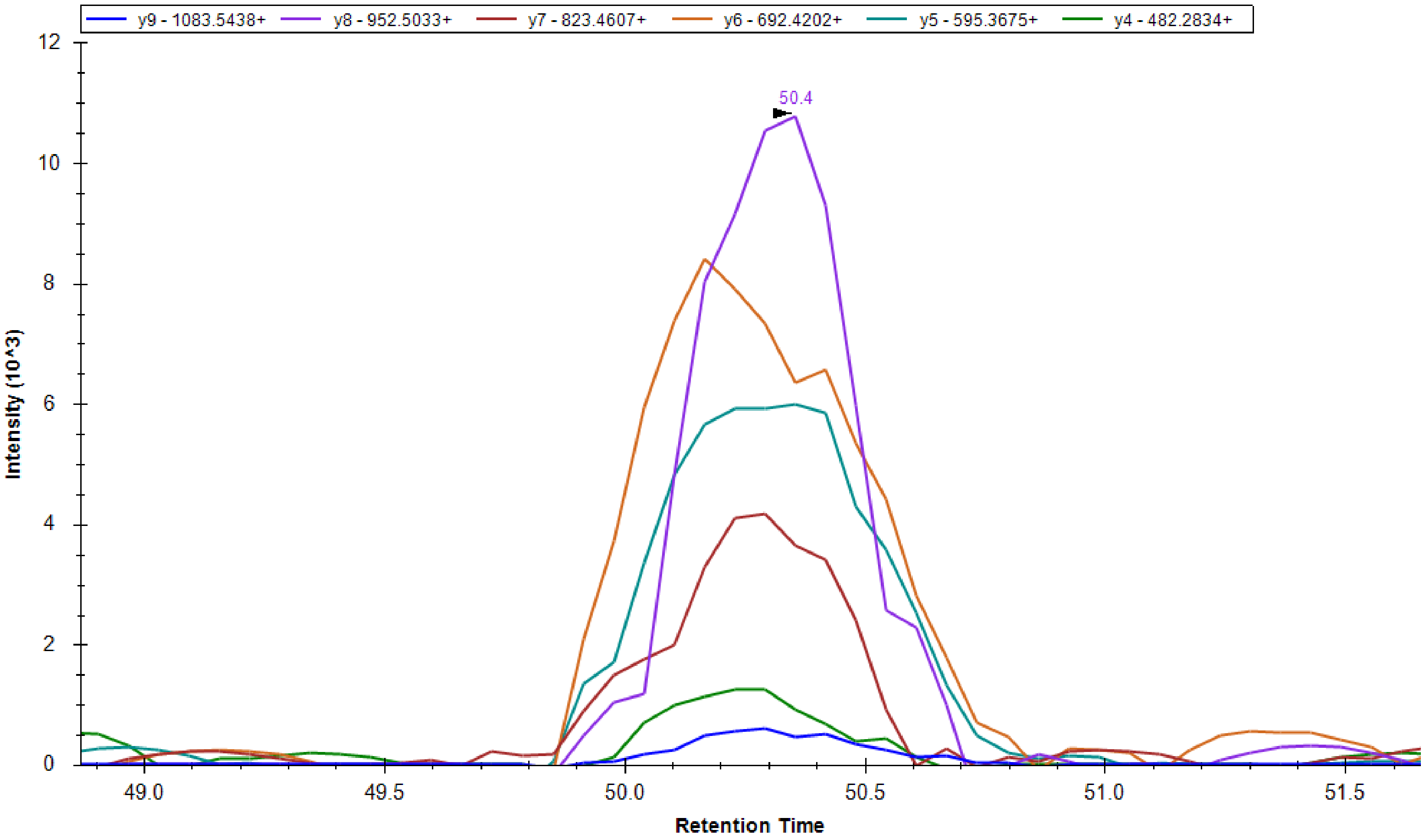

File name: 391-new#A-Round02_Negative-screen.skyd

Parent ion m/z and charges: 591.8097++

NR_110695.2.1
WGAPLPGCDPVWELR

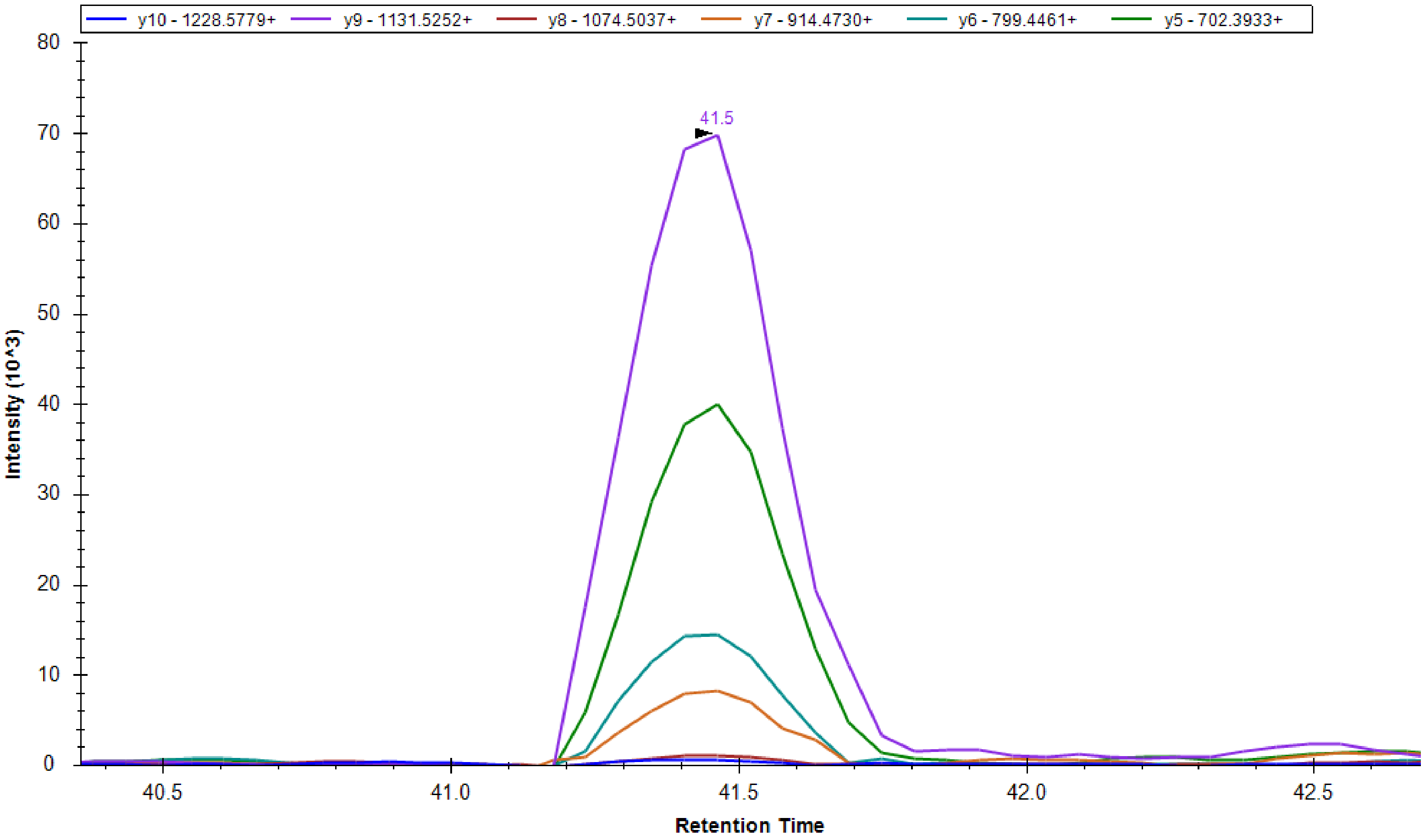

File name: 391-new#B-Round02_Negative-screen.skyd

Parent ion m/z and charges: 876.9300++

NR_110695.2.1
WGAPLPGCDPVWELR

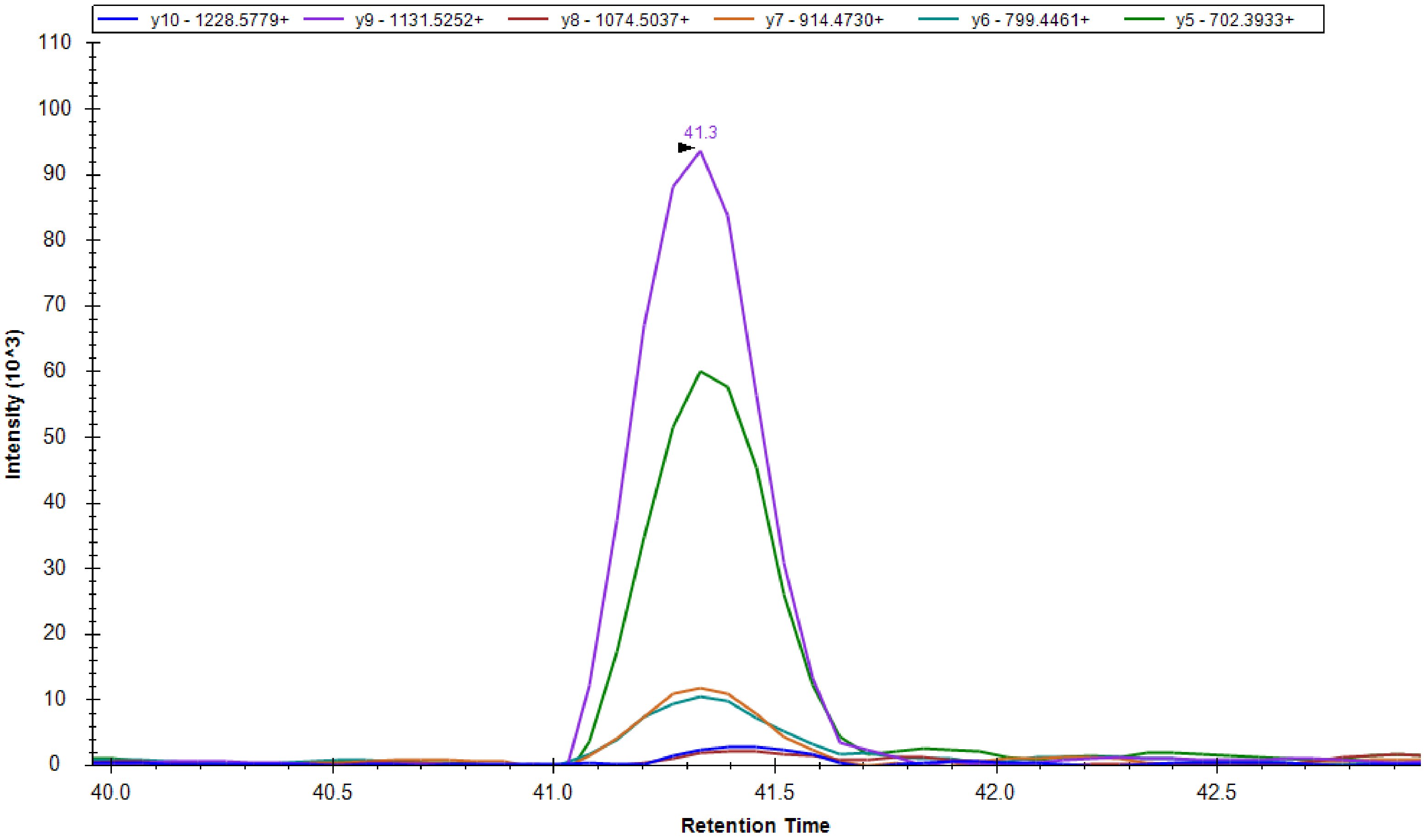

File name: 391-new#A-Round02_Negative-screen.skyd

Parent ion m/z and charges: 876.9300++

NR_110697.2.1
CAPETRLVPR

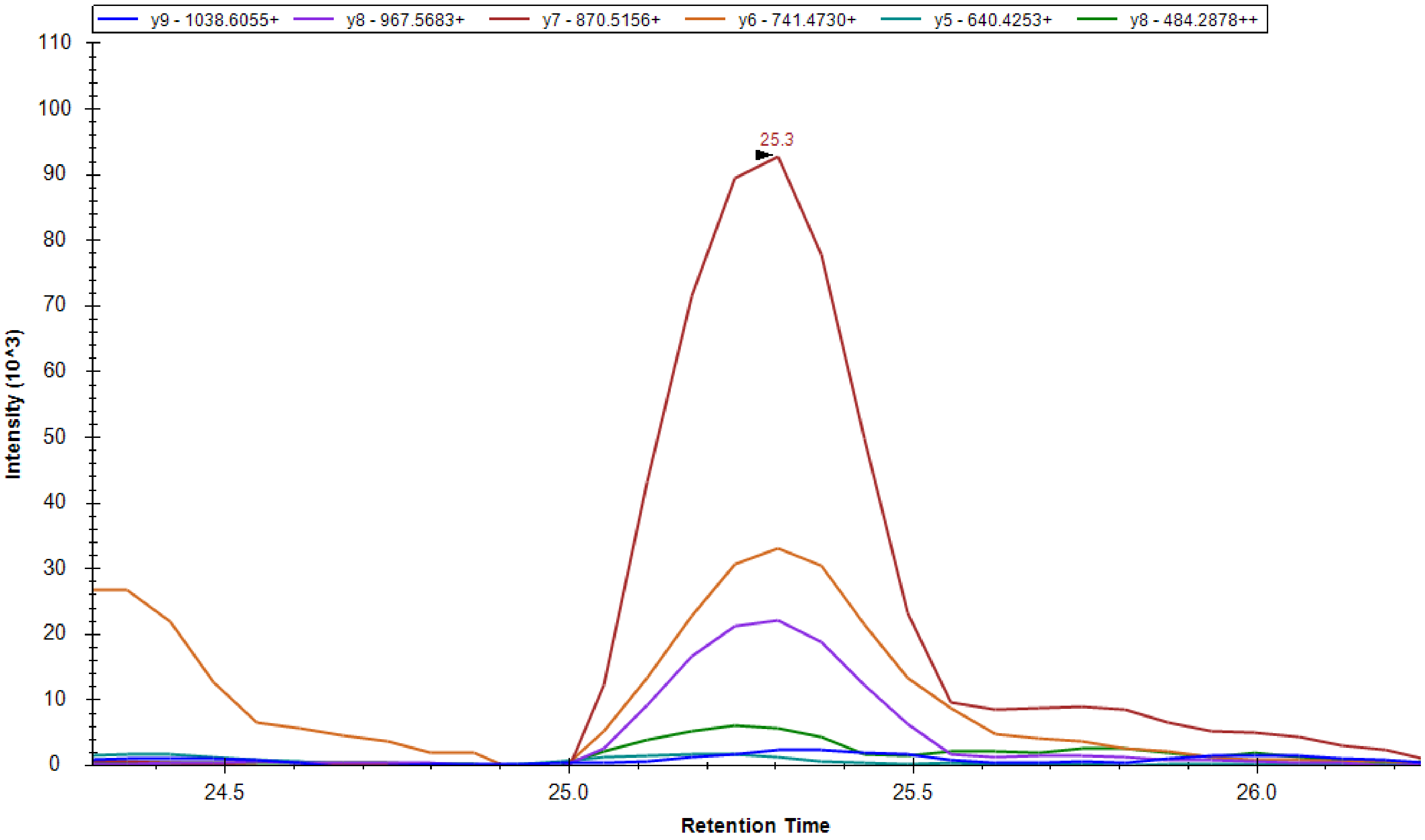

File name: 391-new#A-Round02_Negative-screen.skyd

Parent ion m/z and charges: 599.8217++

NR_110764.1.3
MLPTMGISKR

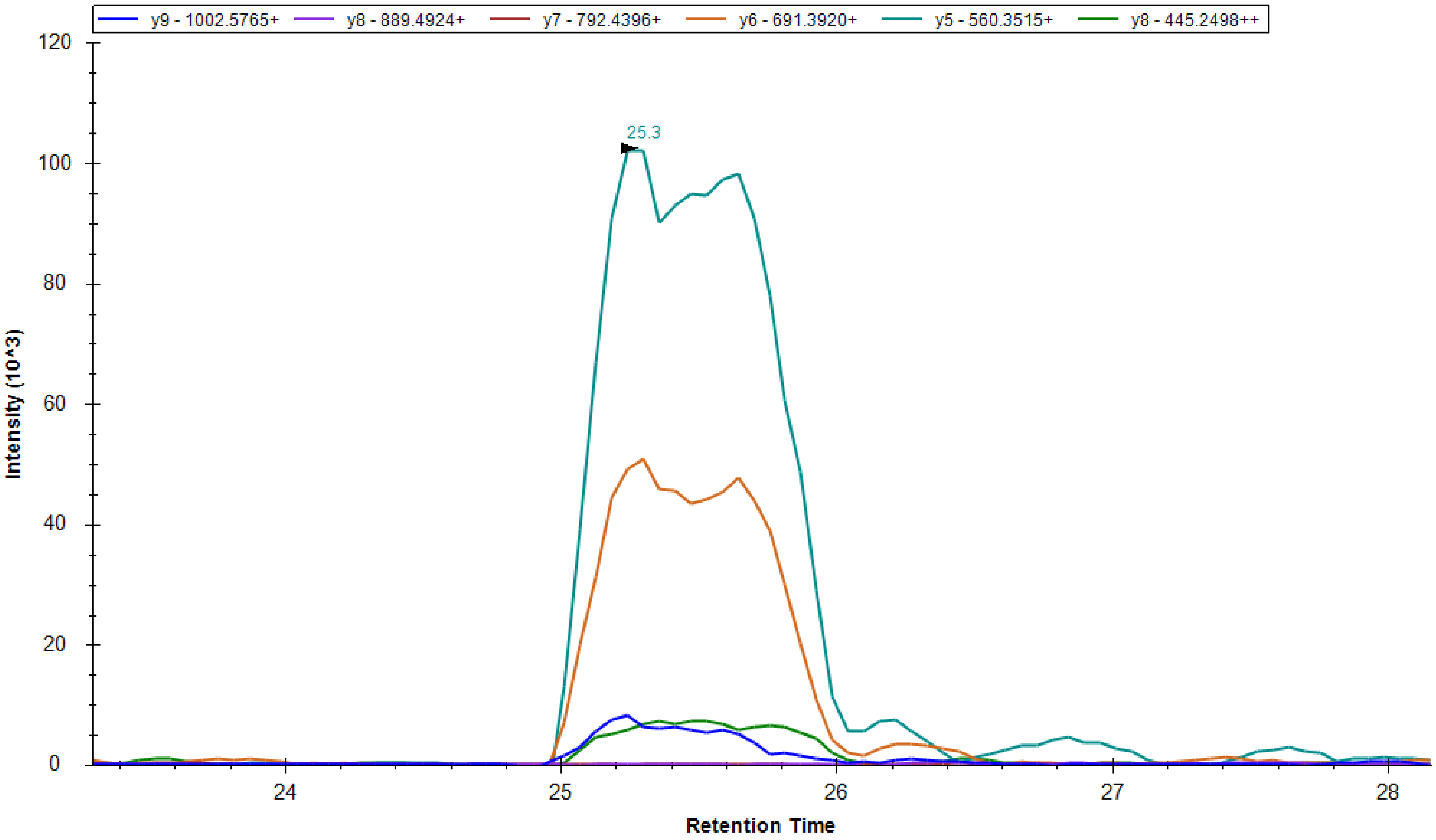

File name: 391-new#B-Round02_Negative-screen.skyd

Parent ion m/z and charges: 567.3121++

NR_110764.1.3
MLPTMGISKR

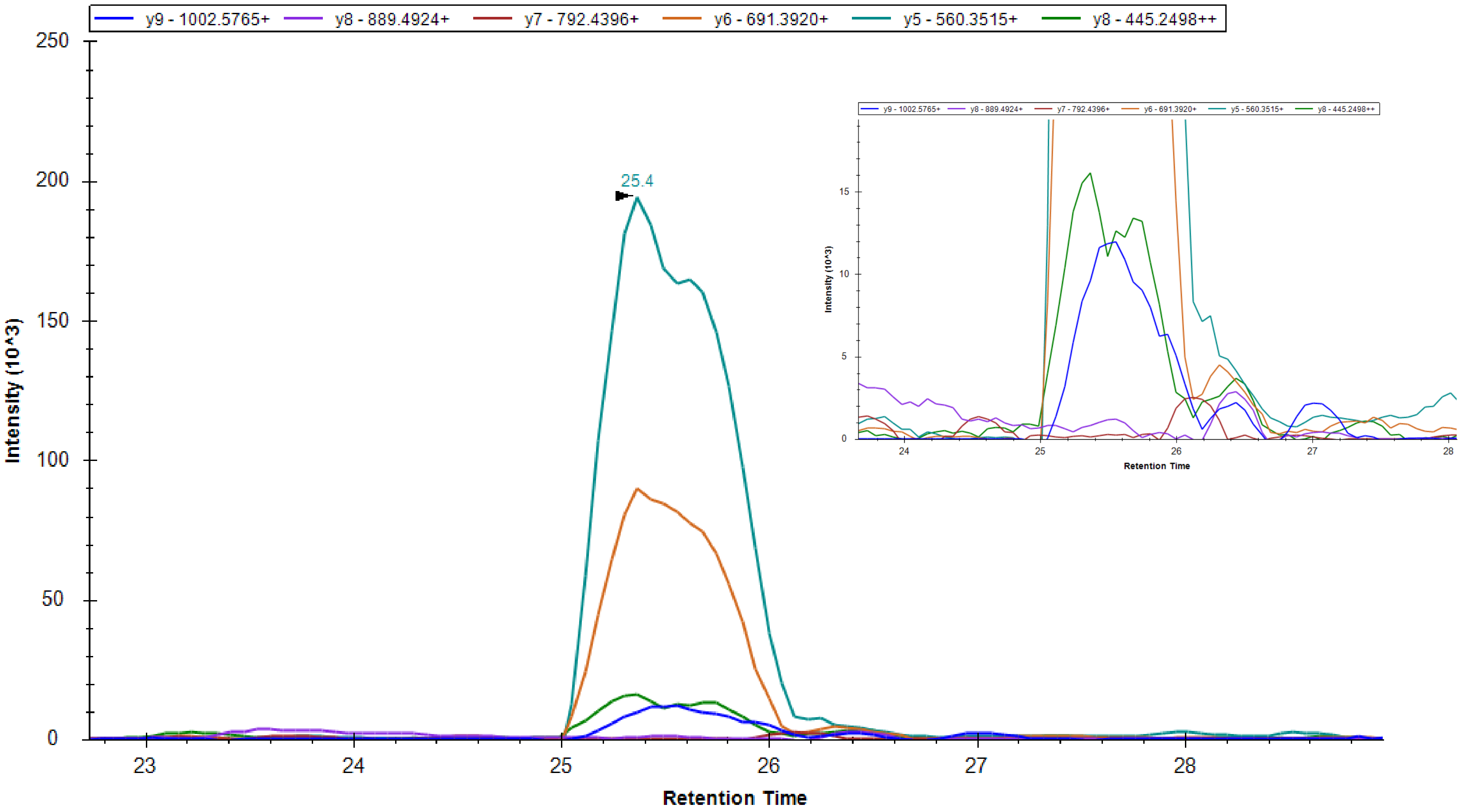

File name: 391-new#A-Round02_Negative-screen.skyd

Parent ion m/z and charges: 567.3121++

NR_110837.1.6
SHHAIPDMRK

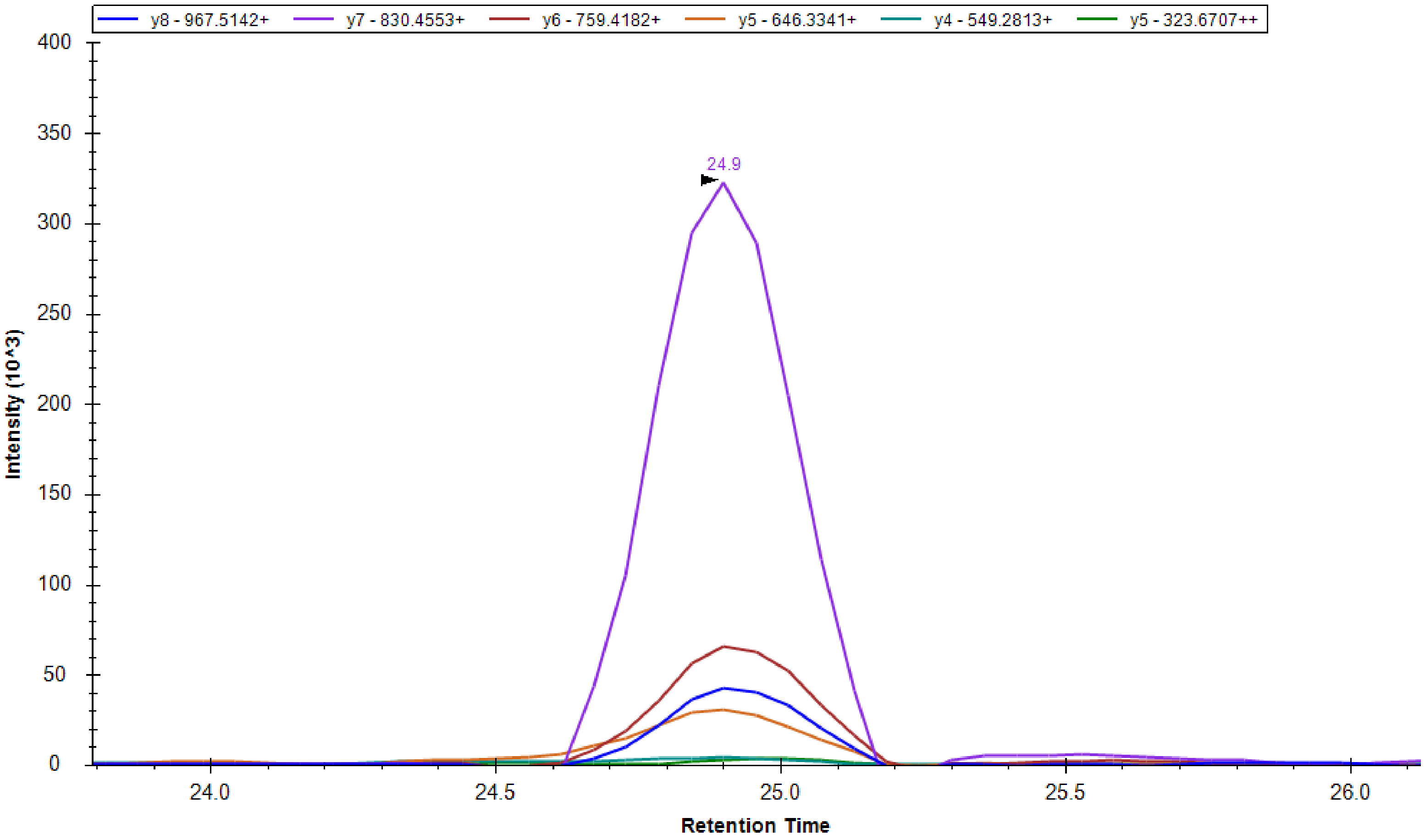

File name: 391-new#B-Round02_Negative-screen.skyd

Parent ion m/z and charges: 596.3062++

NR_110837.1.6
SHHAIPDMRK

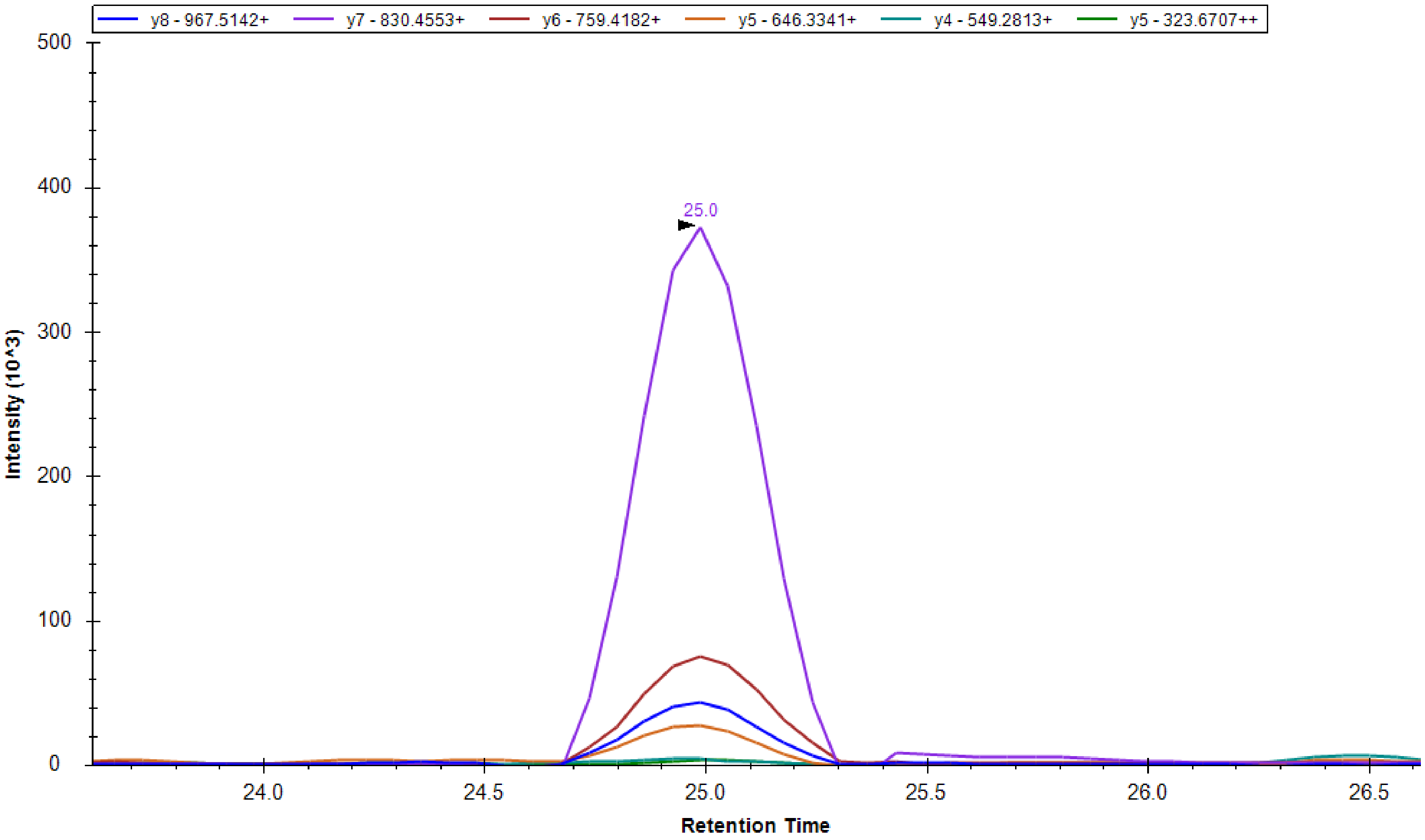

File name: 391-new#A-Round02_Negative-screen.skyd

Parent ion m/z and charges: 596.3062++

NR_110854.1.3
MSIRSDKIK

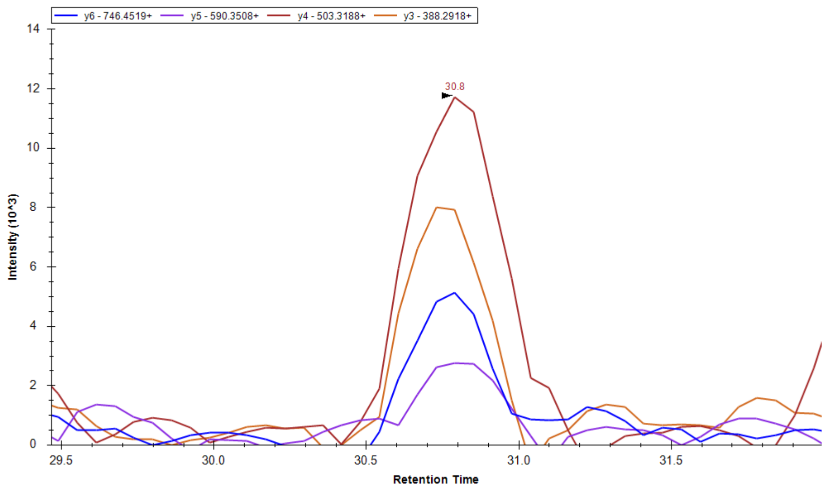

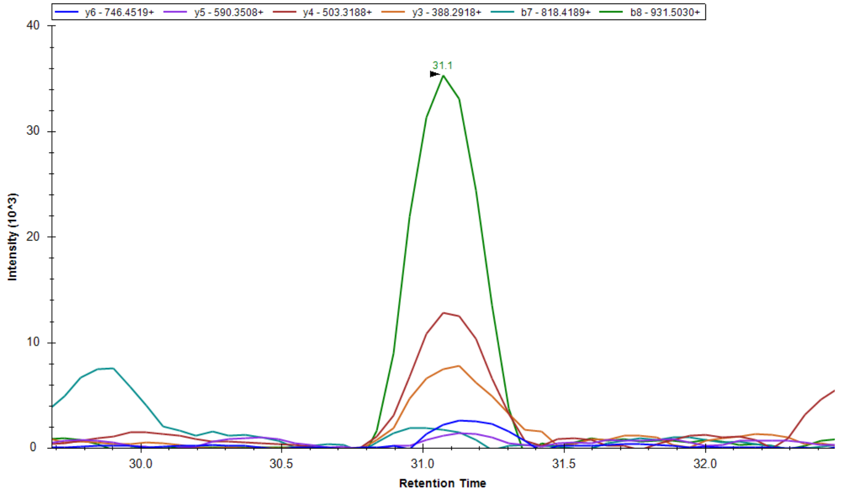

File name: 391-new#B-Round01_All-screening_Positive result.skyd

File name: 391-new#B-Round01_Positive-confirm.skyd

Parent ion m/z and charges: 539.3079++

NR_110854.1.3
MSIRSDKIK

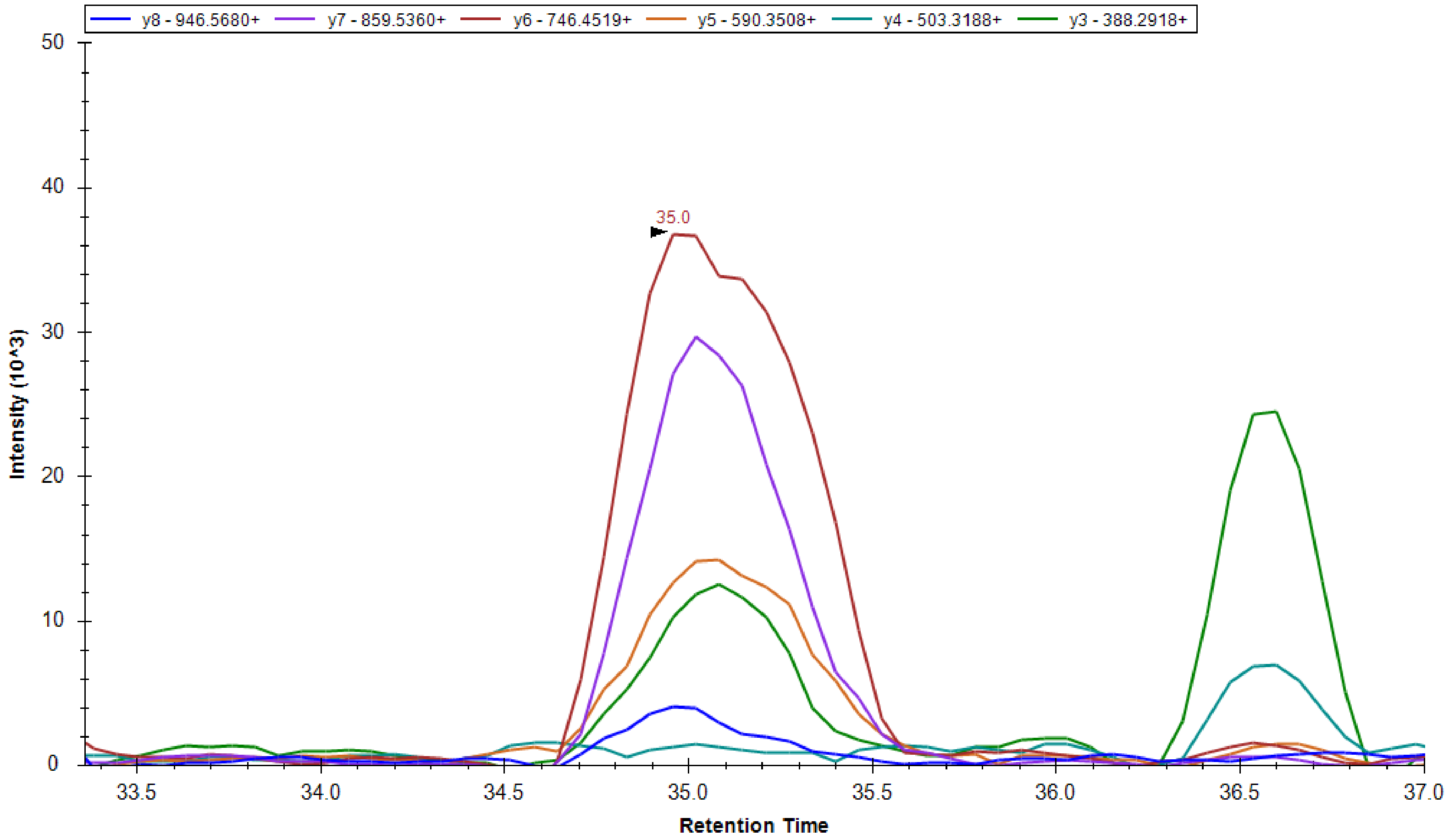

File name: 391-new#A-Round02_Negative-screen.skyd

Parent ion m/z and charges: 539.3079++

NR_111003.1.5
MQLWDELPGK

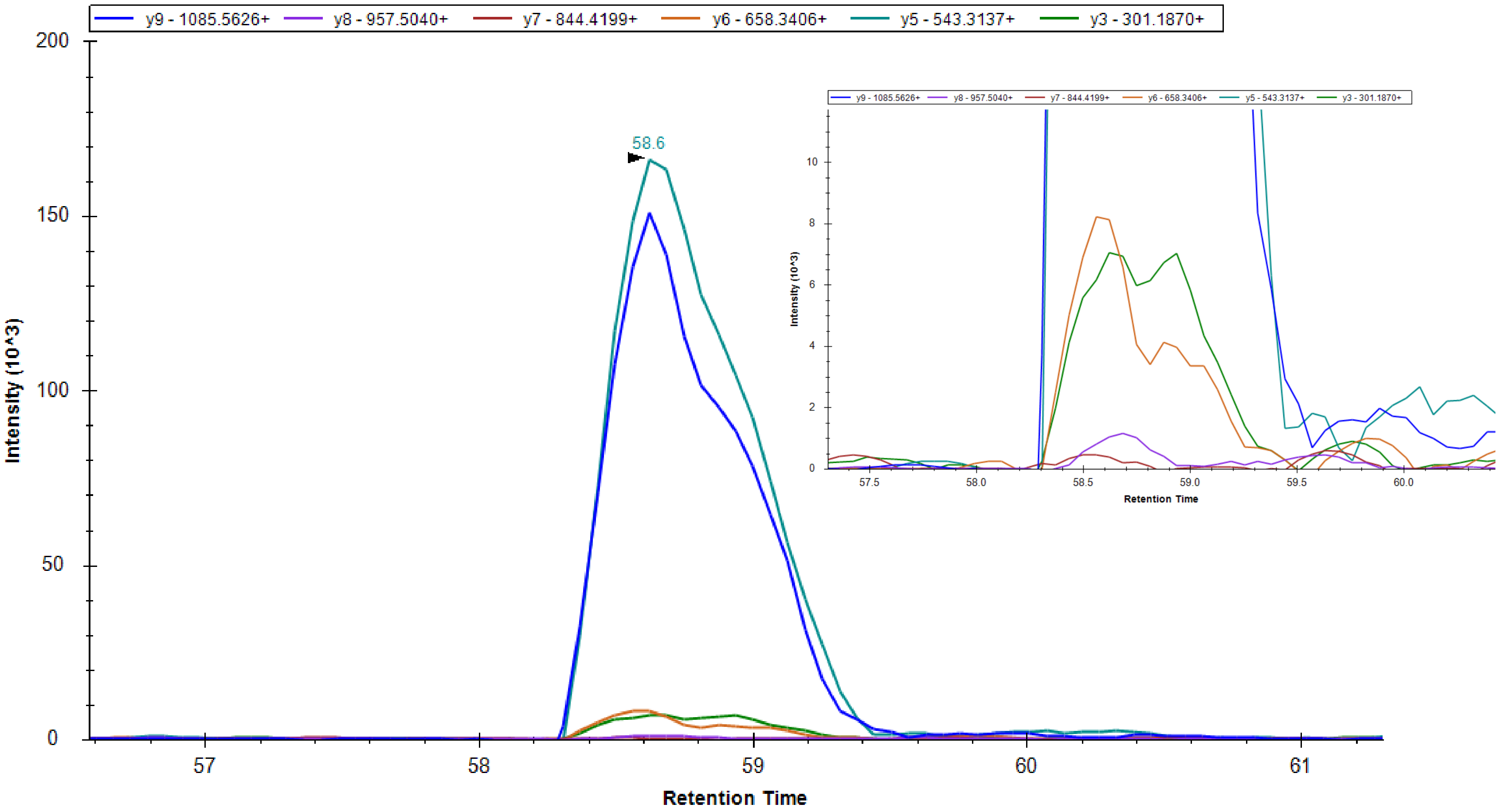

File name: 391-new#A-Round02_Negative-screen.skyd

Parent ion m/z and charges: 608.8052++

NR_111003.1.5
MQLWDELPGK

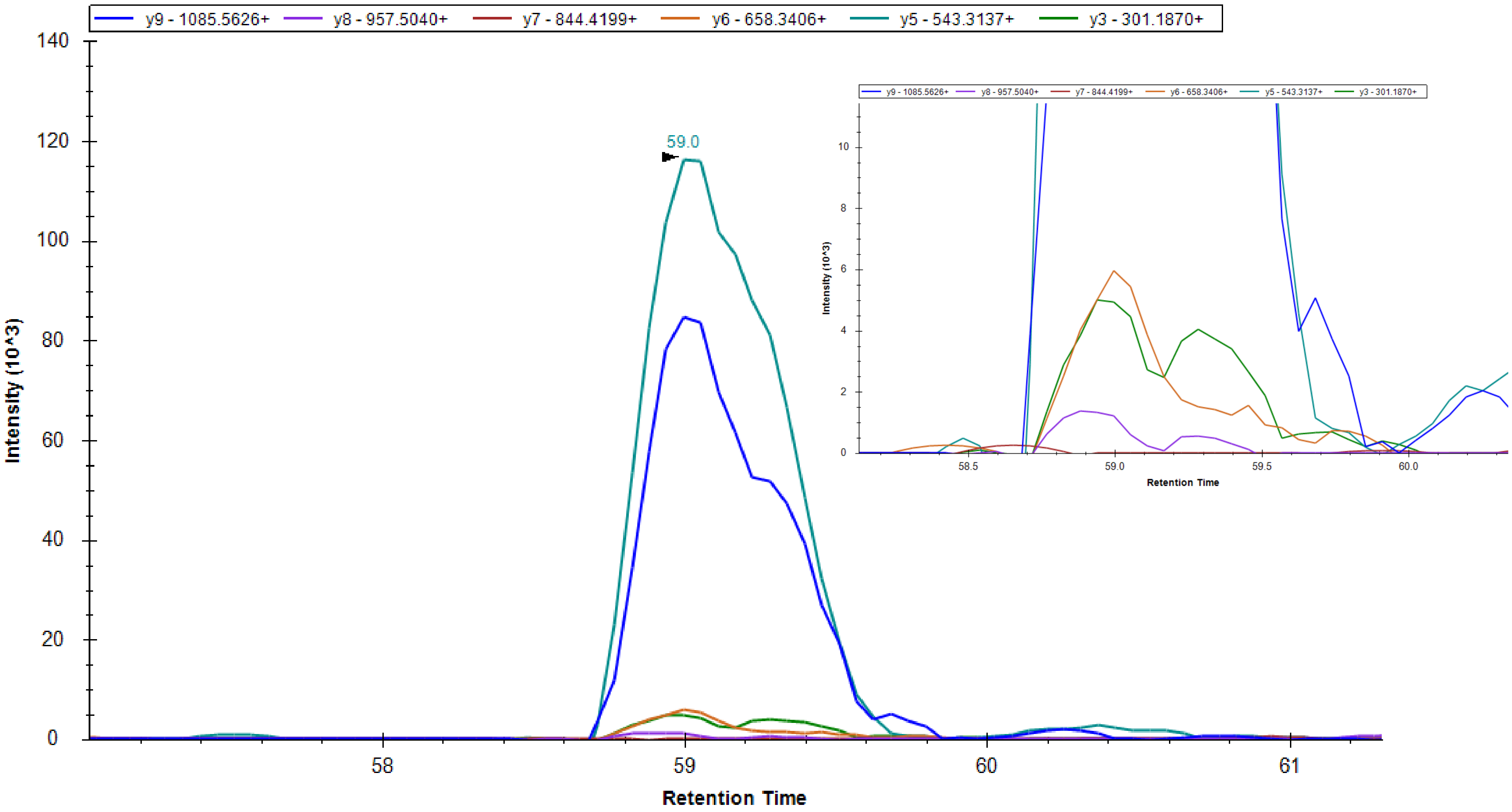

File name: 391-new#B-Round02_Negative-screen.skyd

Parent ion m/z and charges: 608.8052++

NR_111950.1.2
MNPLSPLAFSPLKR

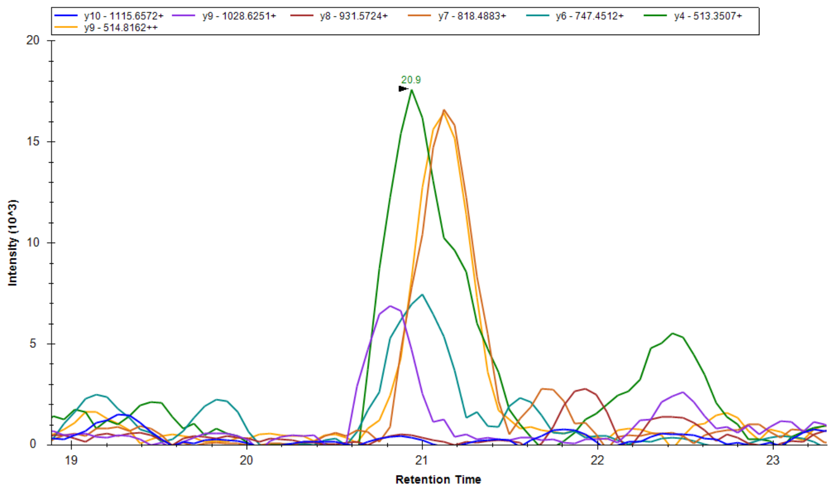

File name: 391-new#B-Round01_All-screening_Positive result.skyd

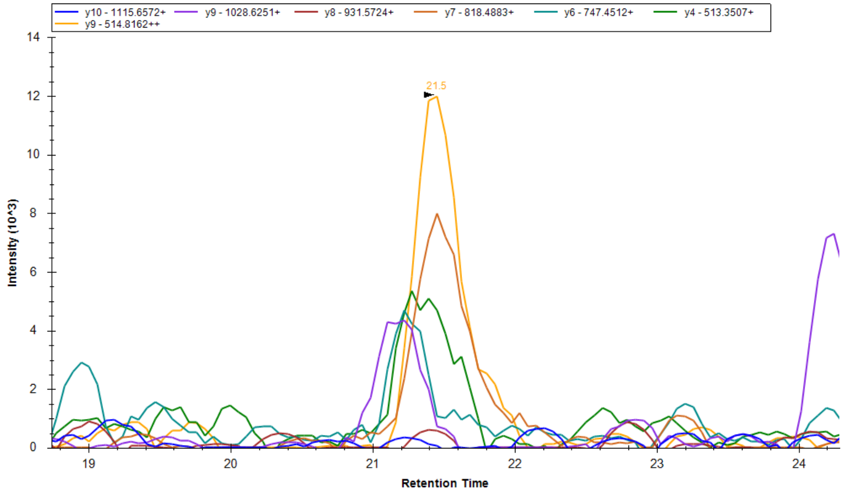

File name: 391-new#B-Round01_Positive-confirm.skyd

Parent ion m/z and charges: 785.9423++

NR_111950.1.2
MNPLSPLAFSPLKR

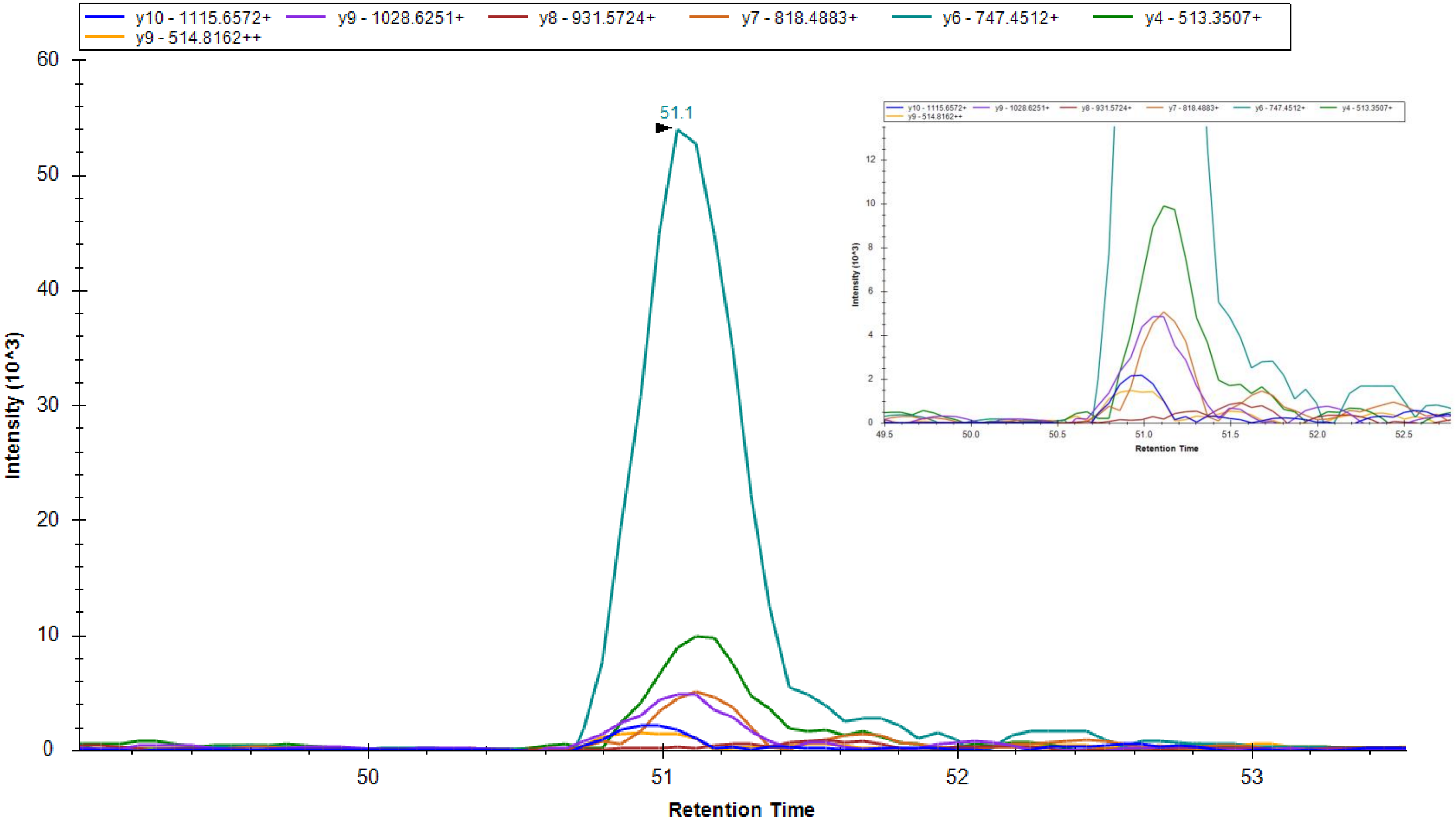

File name: 391-new#A-Round02_Negative-screen.skyd

Parent ion m/z and charges: 785.9423++

NR_111987.3.9
LEAQGAGLCRR

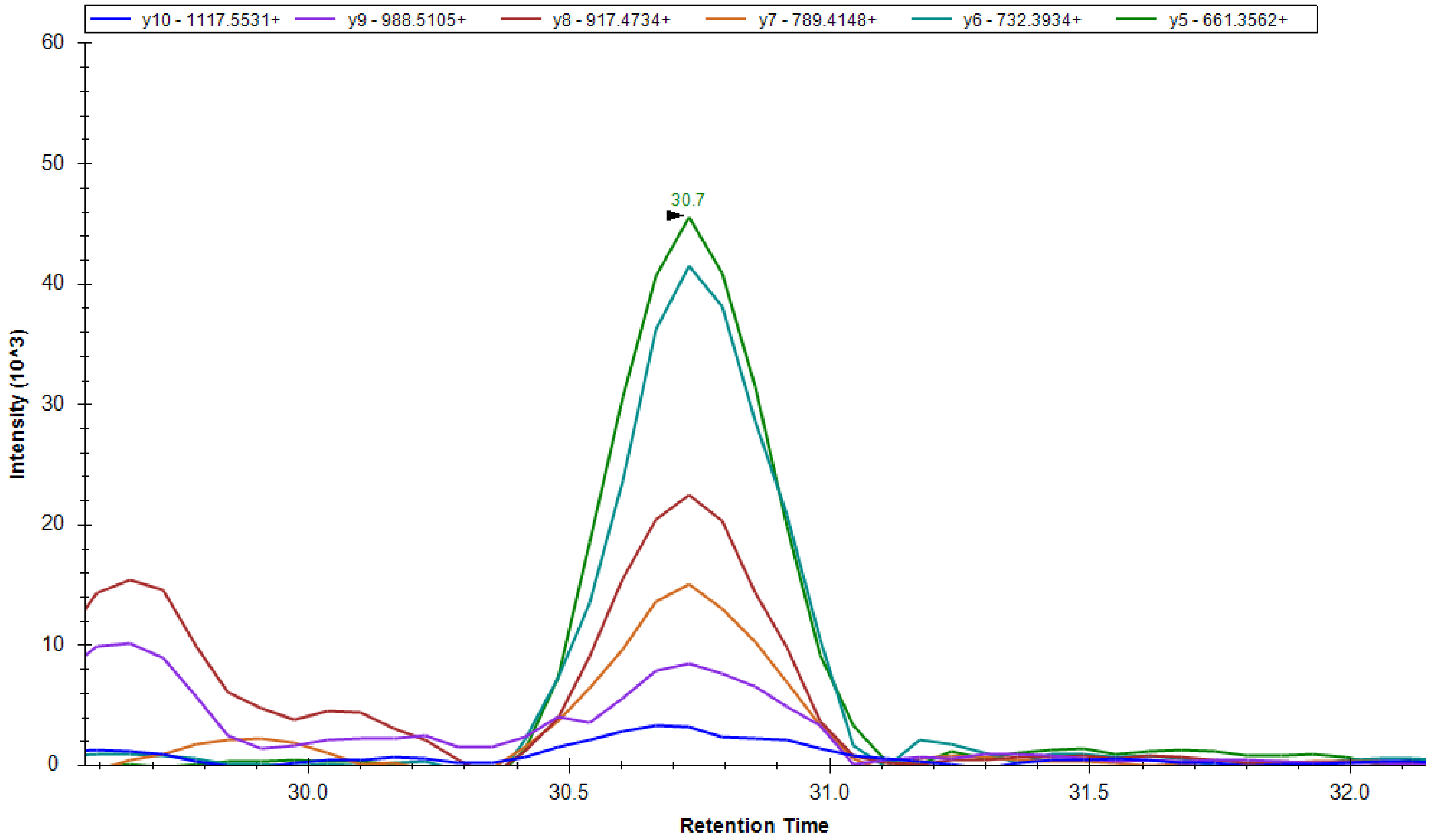

File name: 391-new#A-Round02_Negative-screen.skyd

Parent ion m/z and charges: 615.8222++

NR_111987.3.9
LEAQGAGLCRR

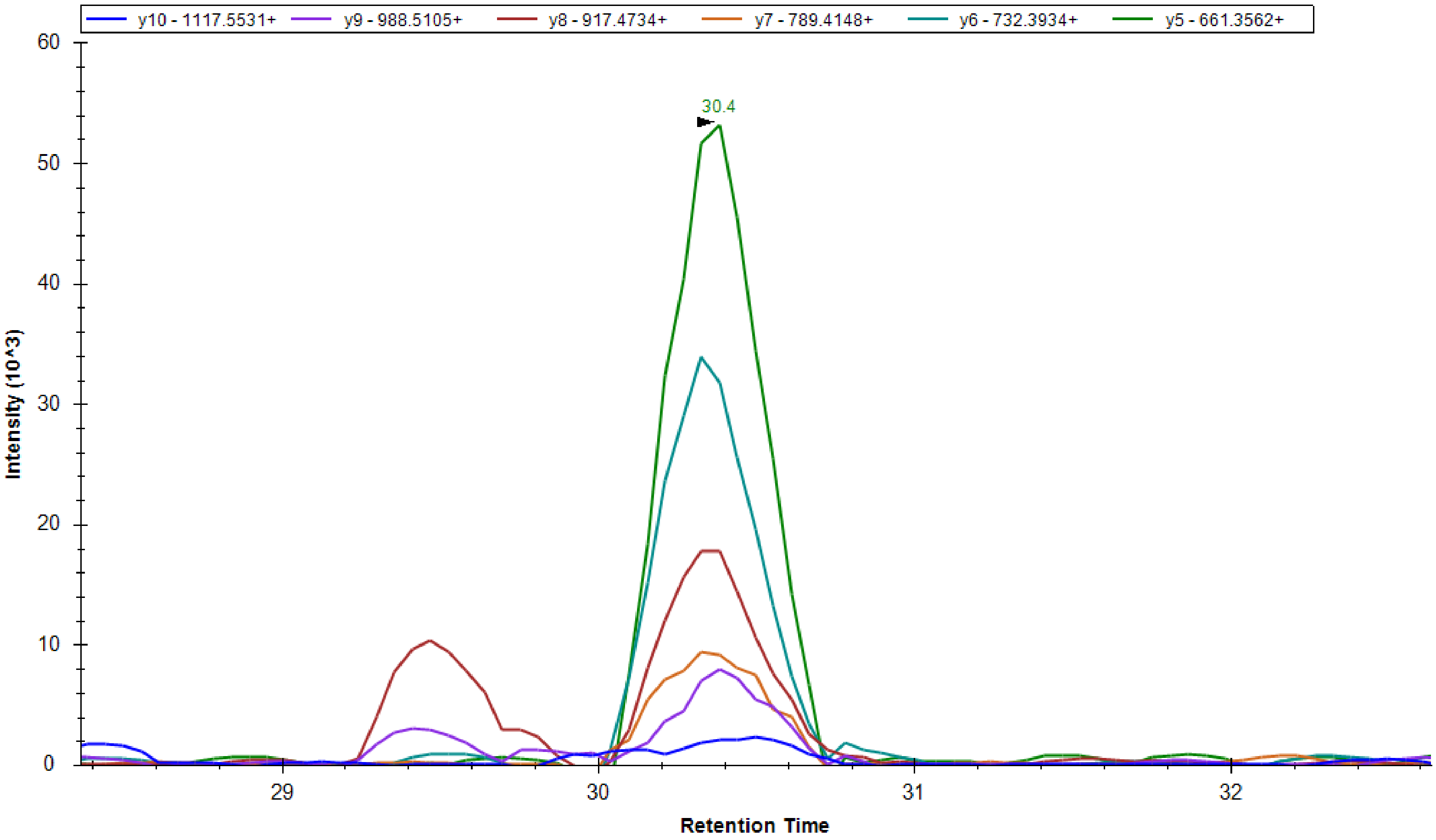

File name: 391-new#B-Round02_Negative-screen.skyd

Parent ion m/z and charges: 615.8222++

NR_119376.1.2
EGDPPTHRHCQPRHSSVVK

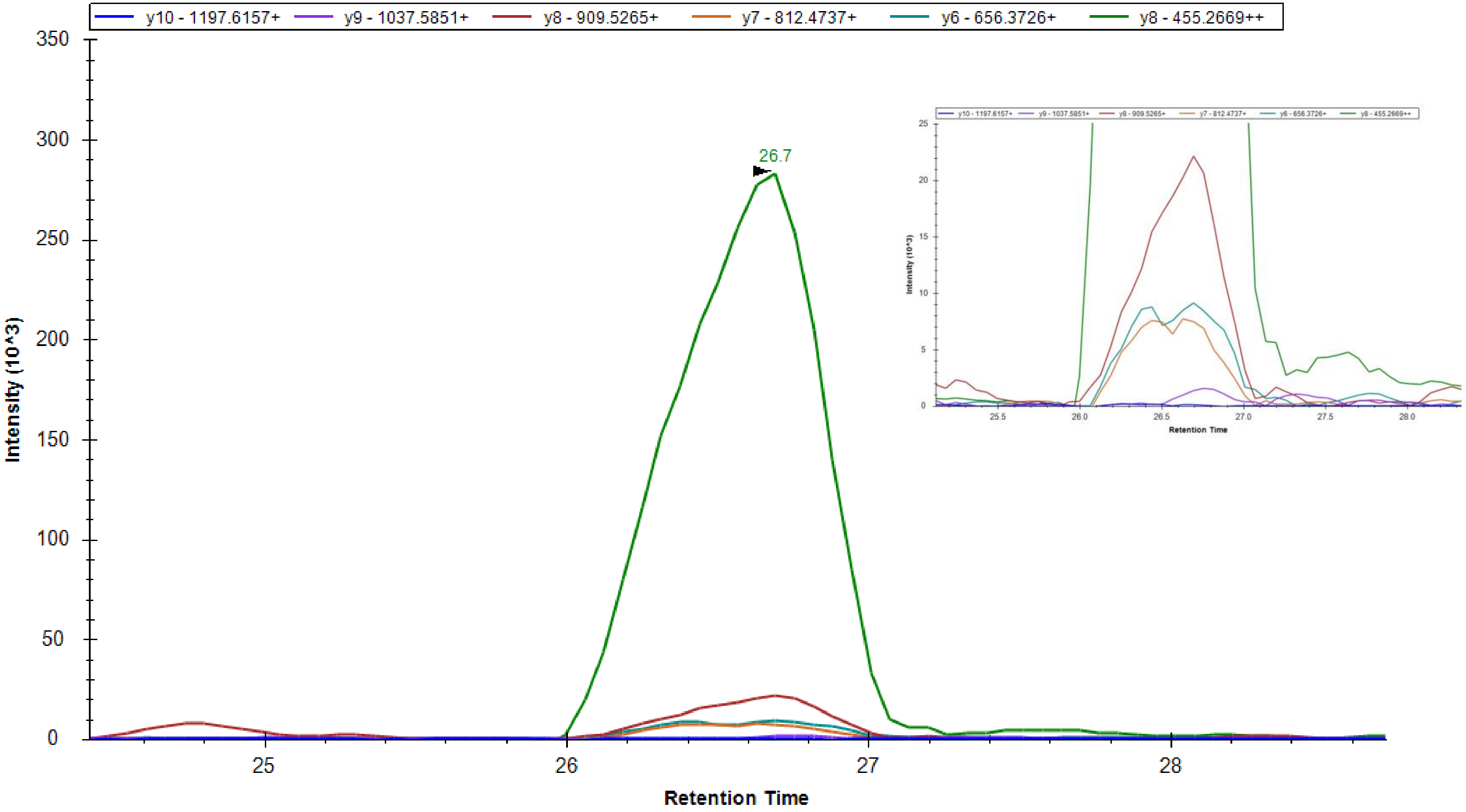

File name: 391-new#A-Round02_Negative-screen.skyd

Parent ion m/z and charges: 742.0311+++

NR_120317.2.2
EEPTPEPVYVDVDK

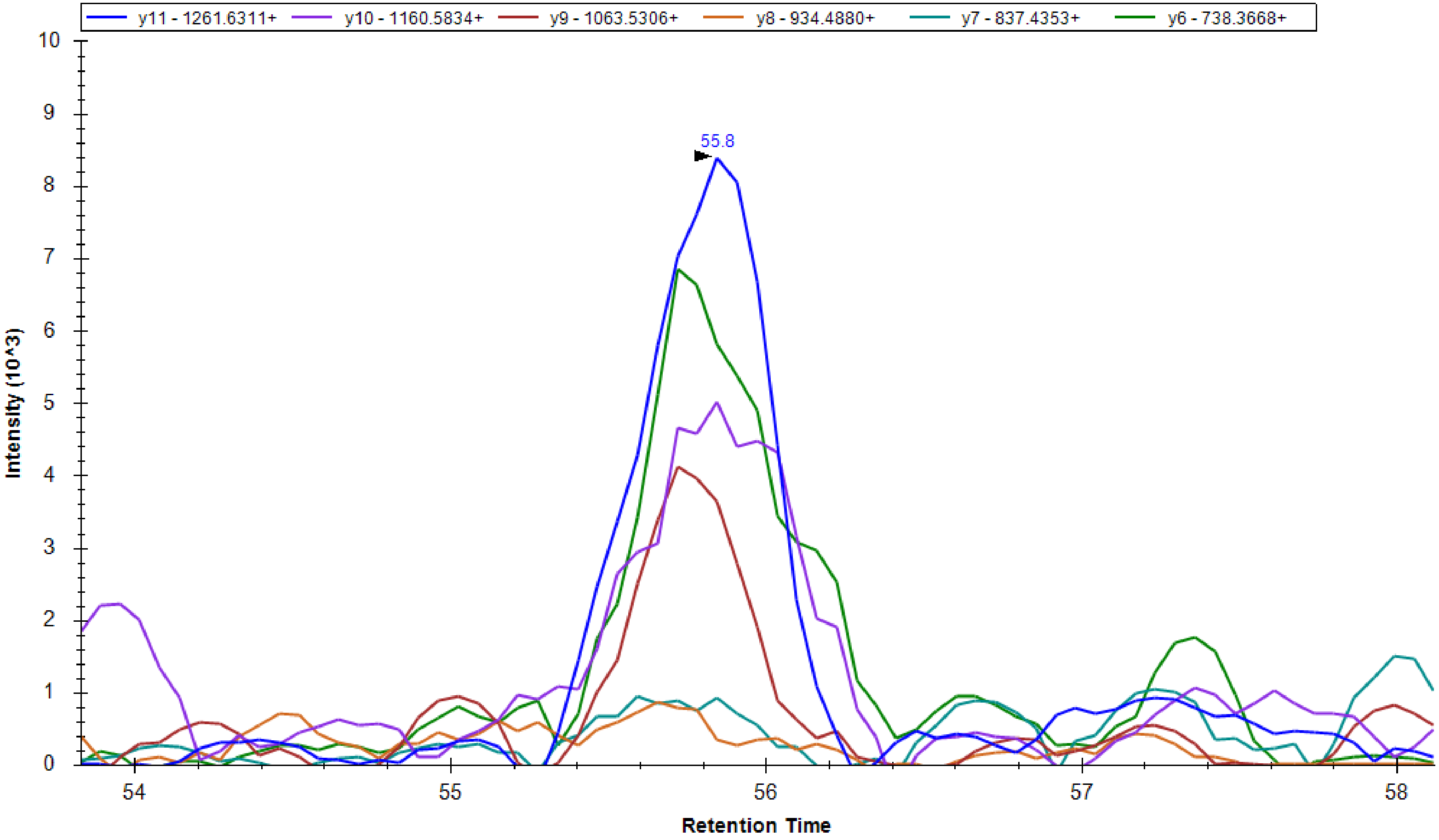

File name: 391-new#A-Round02_Negative-screen.skyd

Parent ion m/z and charges: 808.8881++

NR_026698.2.12
MFFDNCHYIIDDK

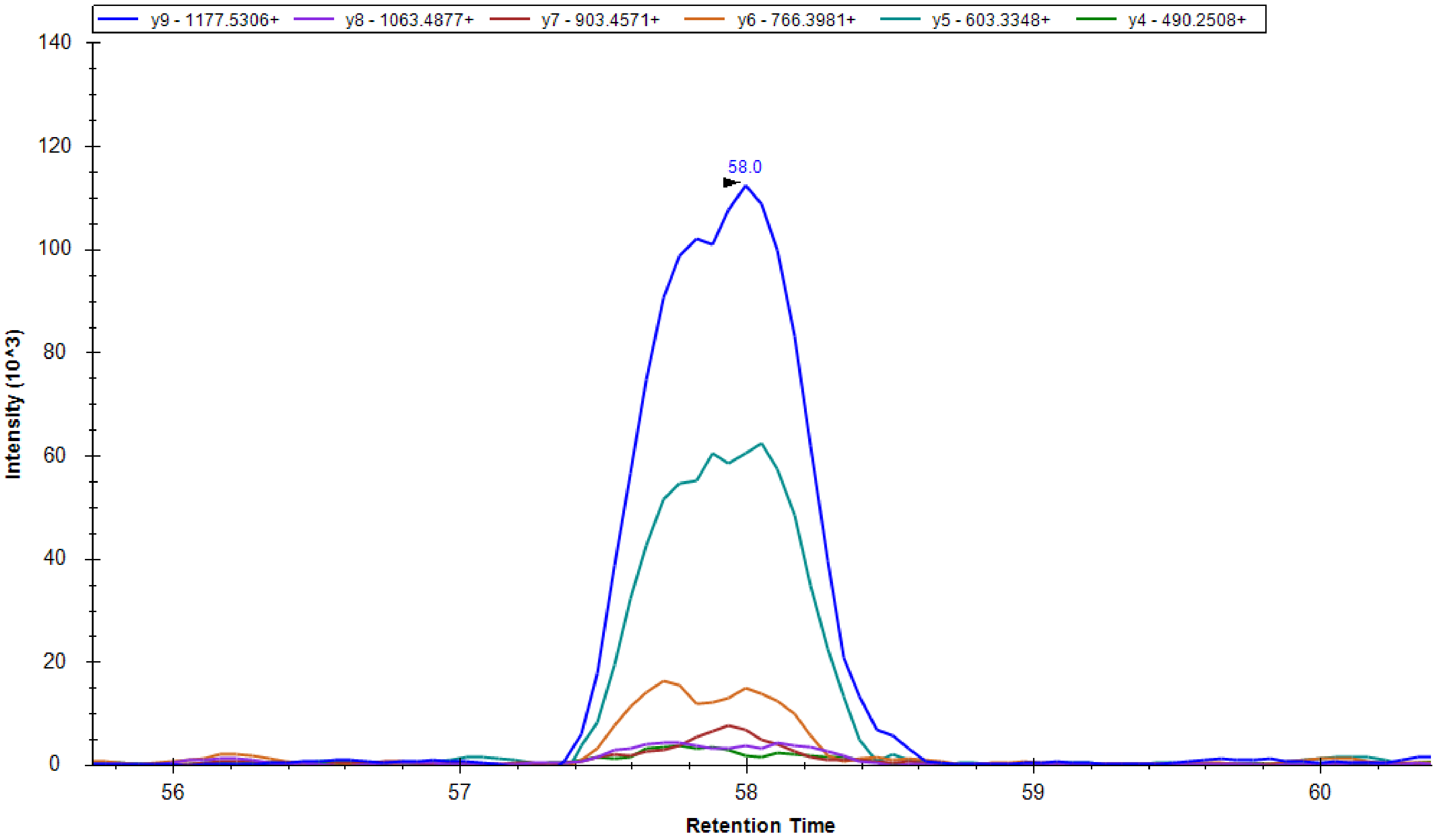

File name: 391-new#B-Round02_Negative-screen.skyd

Parent ion m/z and charges: 859.3711++

NR_002728.3.229
ITMRYHITIMR

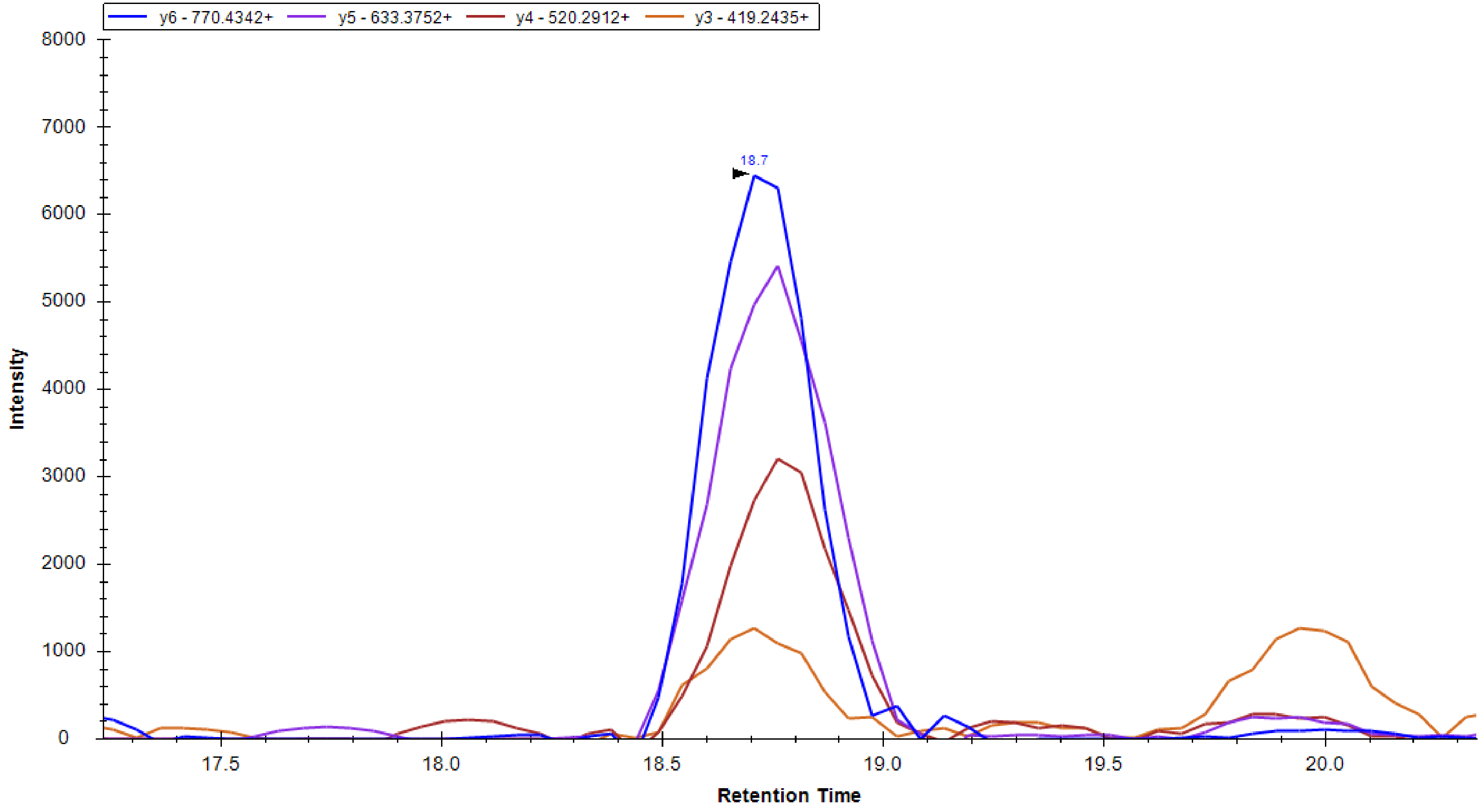

File name: 1604_JinanU(GZ)_MRM_#B_2nd_Round_Screen.skyd

Parent ion m/z and charges: 717.8890++

NR_002171.2.4
MGVPSQGGTR

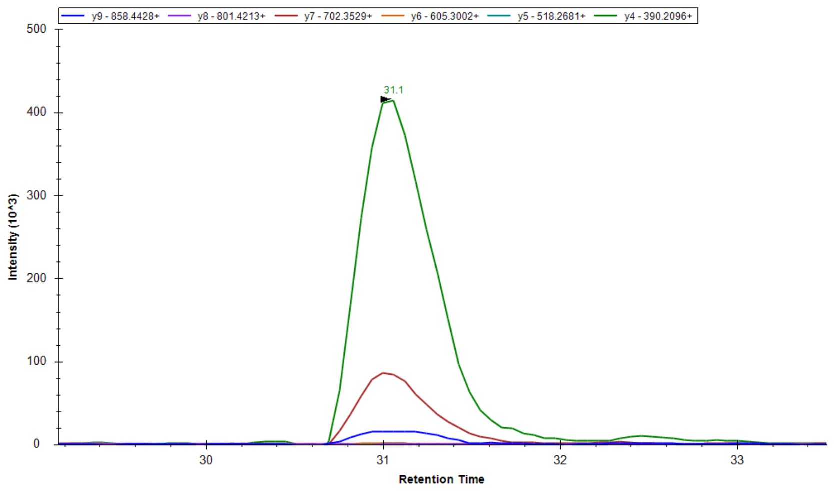

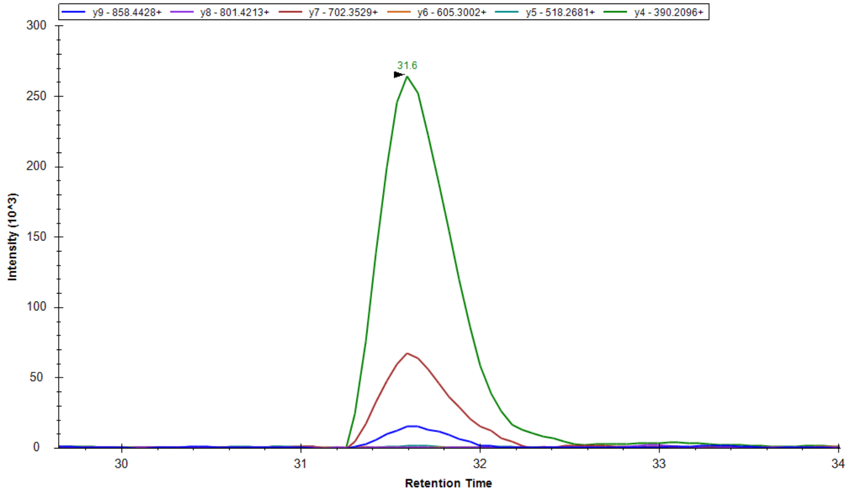

File name: 391-new#B-Round01_All-screening_Positive result.skyd

File name: 391-new#B-Round01_Positive-confirm.skyd

Parent ion m/z and charges: 495.2453++

NR_002223.2.1
QQEVLENHFKEEQYPDYDTR

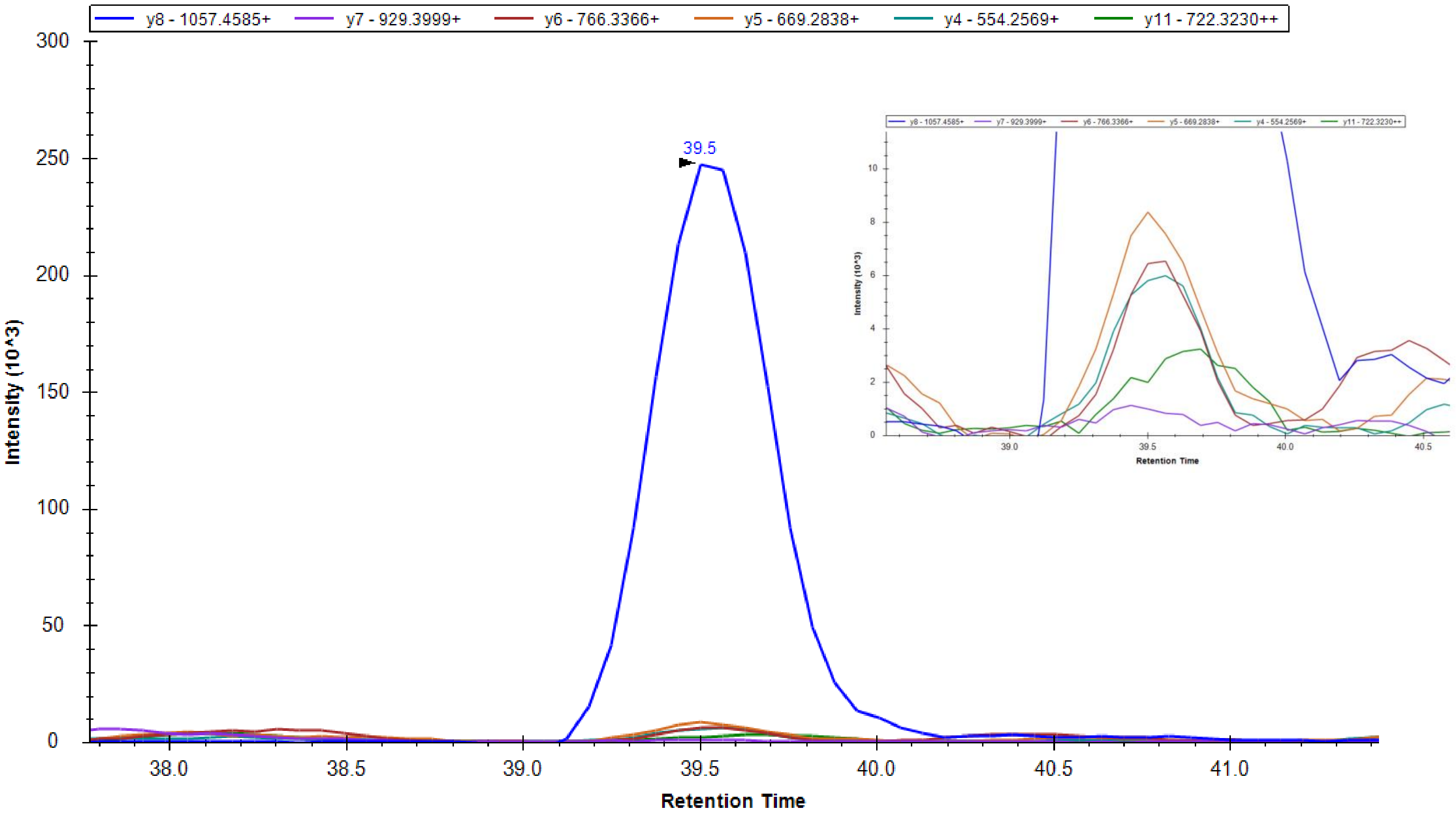

File name: 391-new#A-Round02_Negative-screen.skyd

Parent ion m/z and charges: 856.7261+++

NR_002305.1.5
NFLILAWRALLER

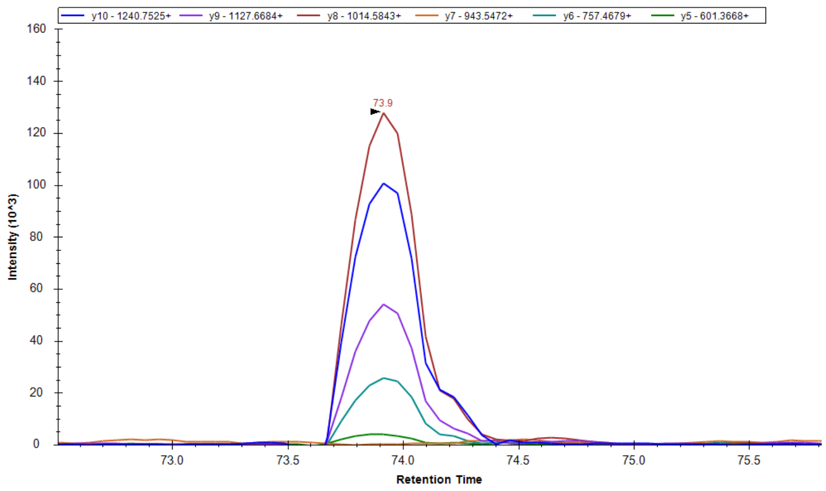

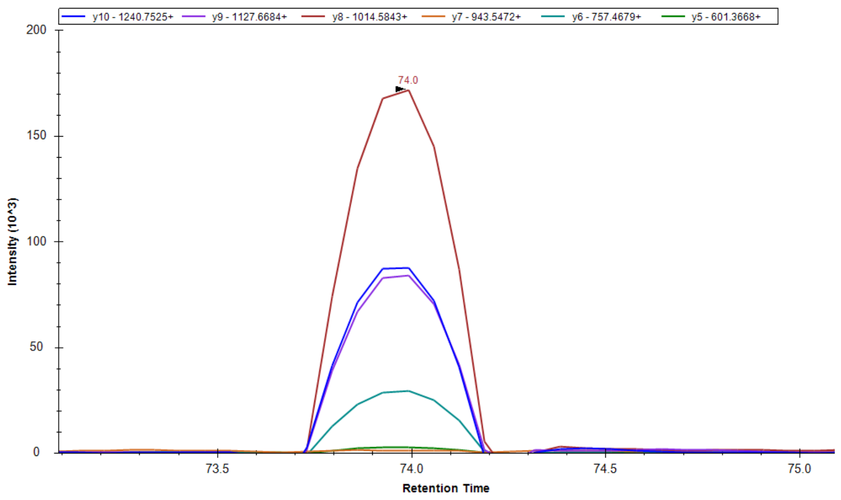

File name: 391-new#A-Round01_All-screening_Positive result.skyd

File name: 391-new#A-Round01_Positive-confirm.skyd

Parent ion m/z and charges: 807.9776++

NR_002305.1.5
NFLILAWRALLER

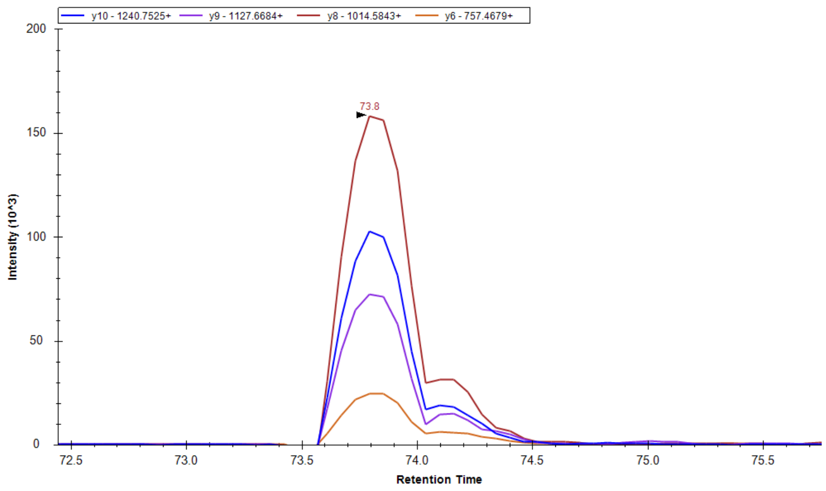

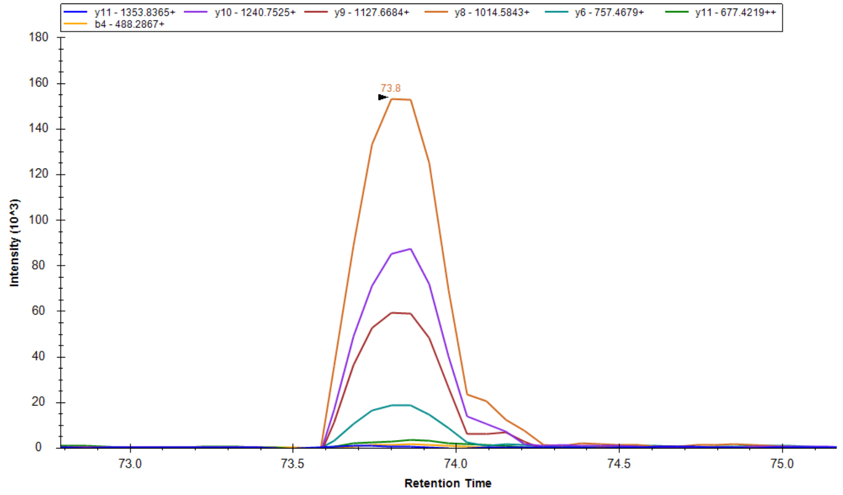

File name: 391-new#B-Round01_All-screening_Positive result.skyd

File name: 391-new#B-Round01_Positive-confirm.skyd

Parent ion m/z and charges: 807.9776++

NR_002726.1.1
MEVKPPPGCPQPDSGSRR

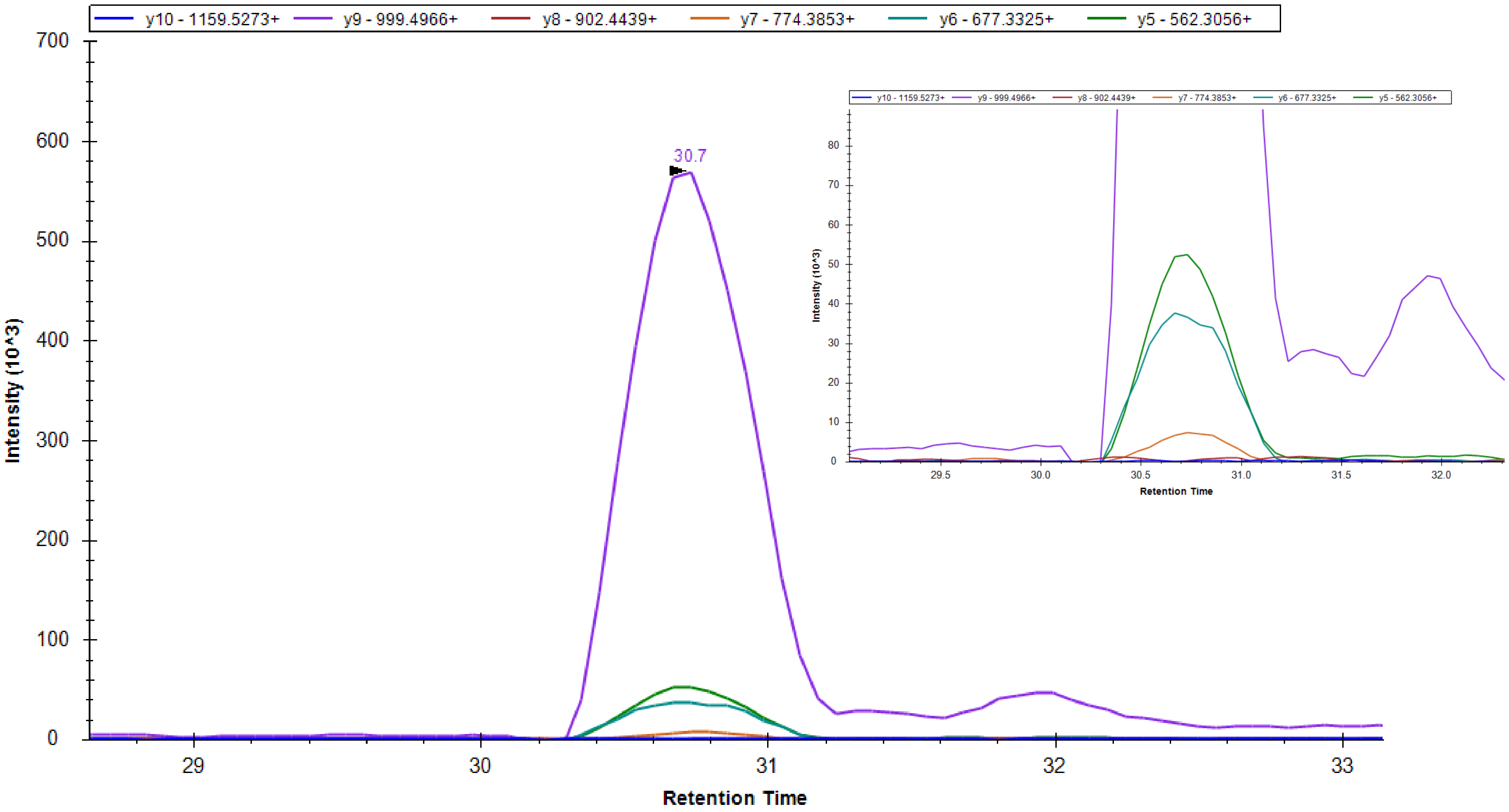

File name: 391-new#A-Round02_Negative-screen.skyd

Parent ion m/z and charges: 997.9804++

NR_002728.1.90
WVMGVSRVSPSRSR

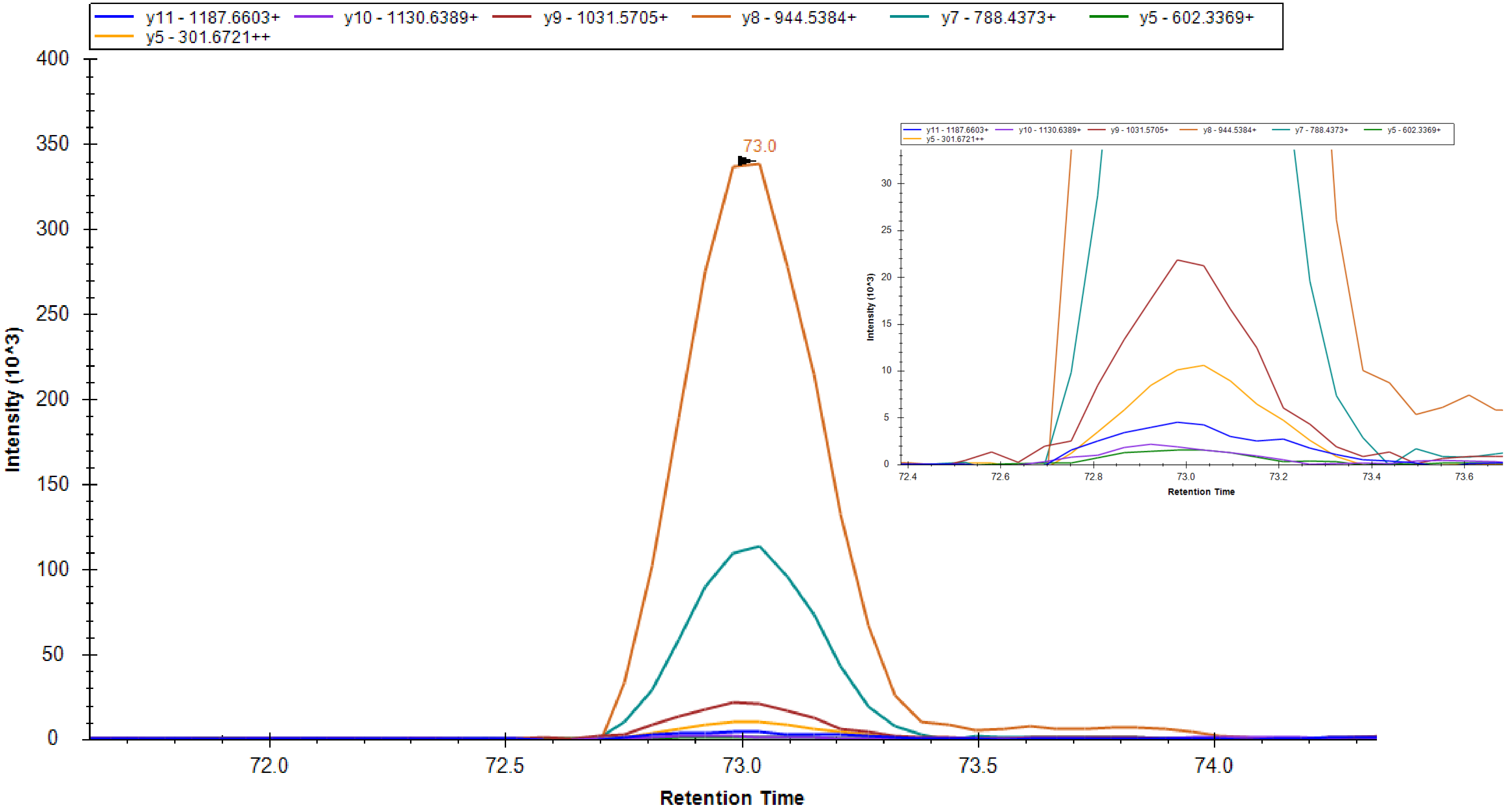

File name: 391-new#B-Round02_Negative-screen.skyd

Parent ion m/z and charges: 802.4279++

NR_002728.2.111
MHPRPAVPQGKANWK

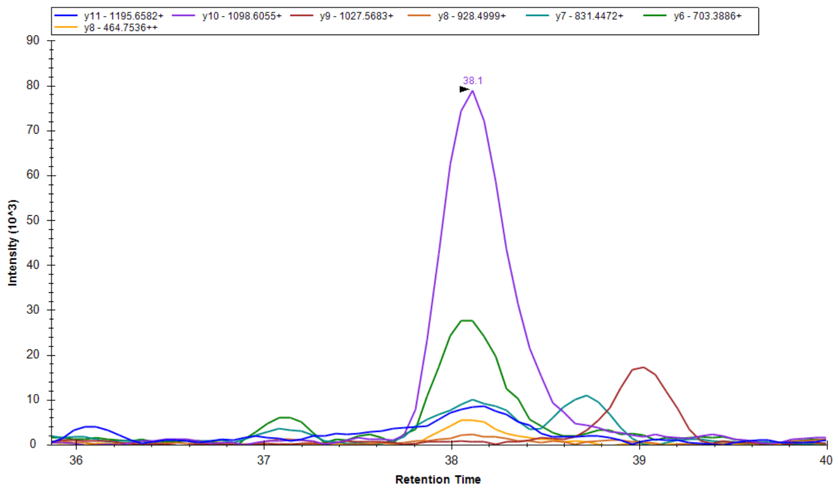

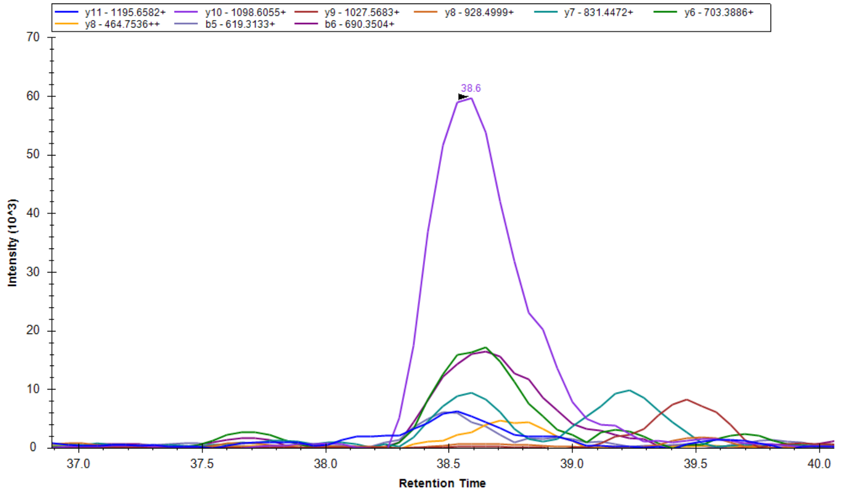

File name: 391-new#B-Round01_All-screening_Positive result.skyd

File name: 391-new#B-Round01_Positive-confirm.skyd

Parent ion m/z and charges: 858.9594++

NR_002728.2.227
MGEKIFANYSSGKGPMSR

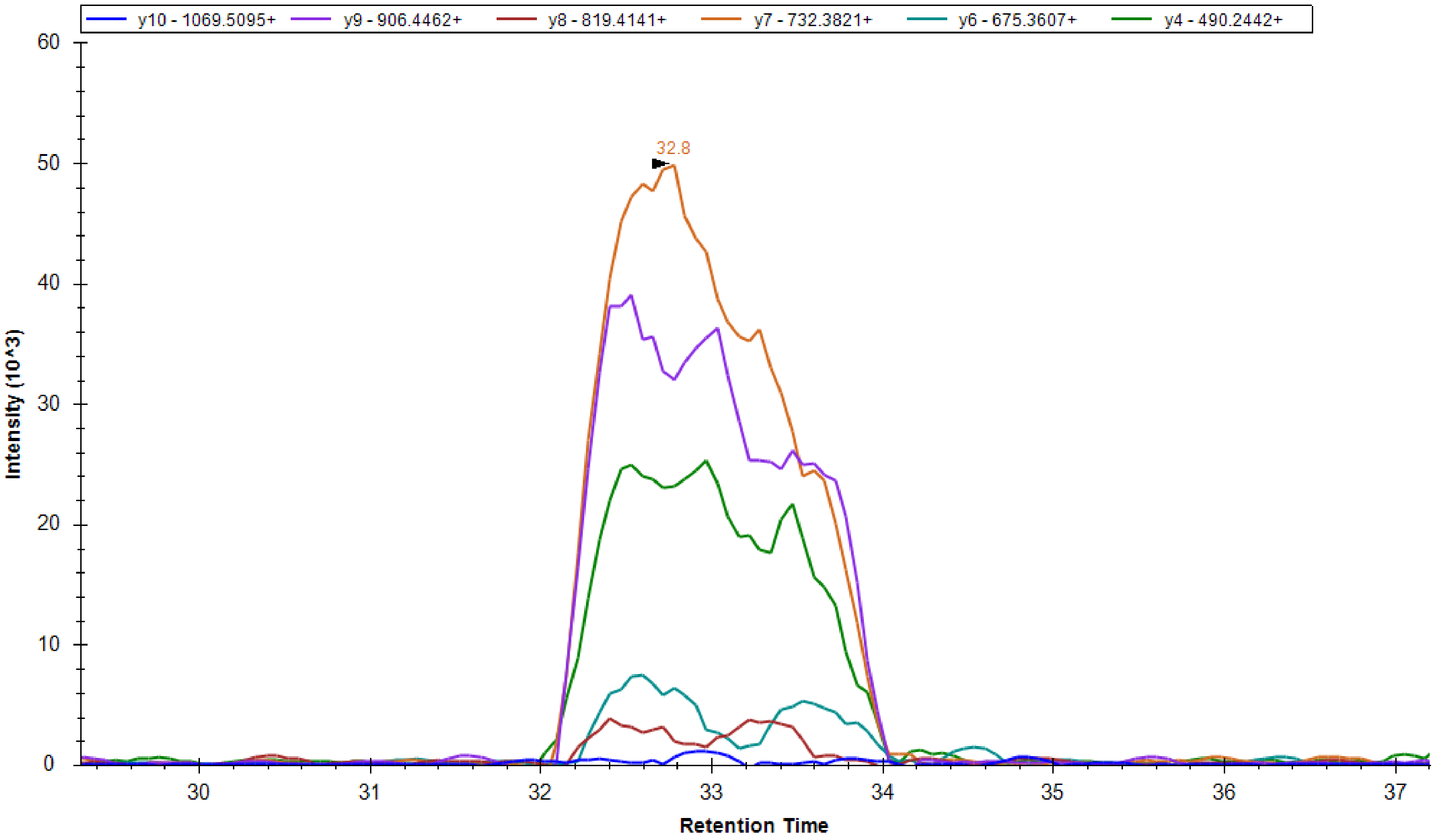

File name: 391-new#A-Round02_Negative-screen.skyd

Parent ion m/z and charges: 653.9854+++

NR_002728.2.227
MGEKIFANYSSGKGPMSR

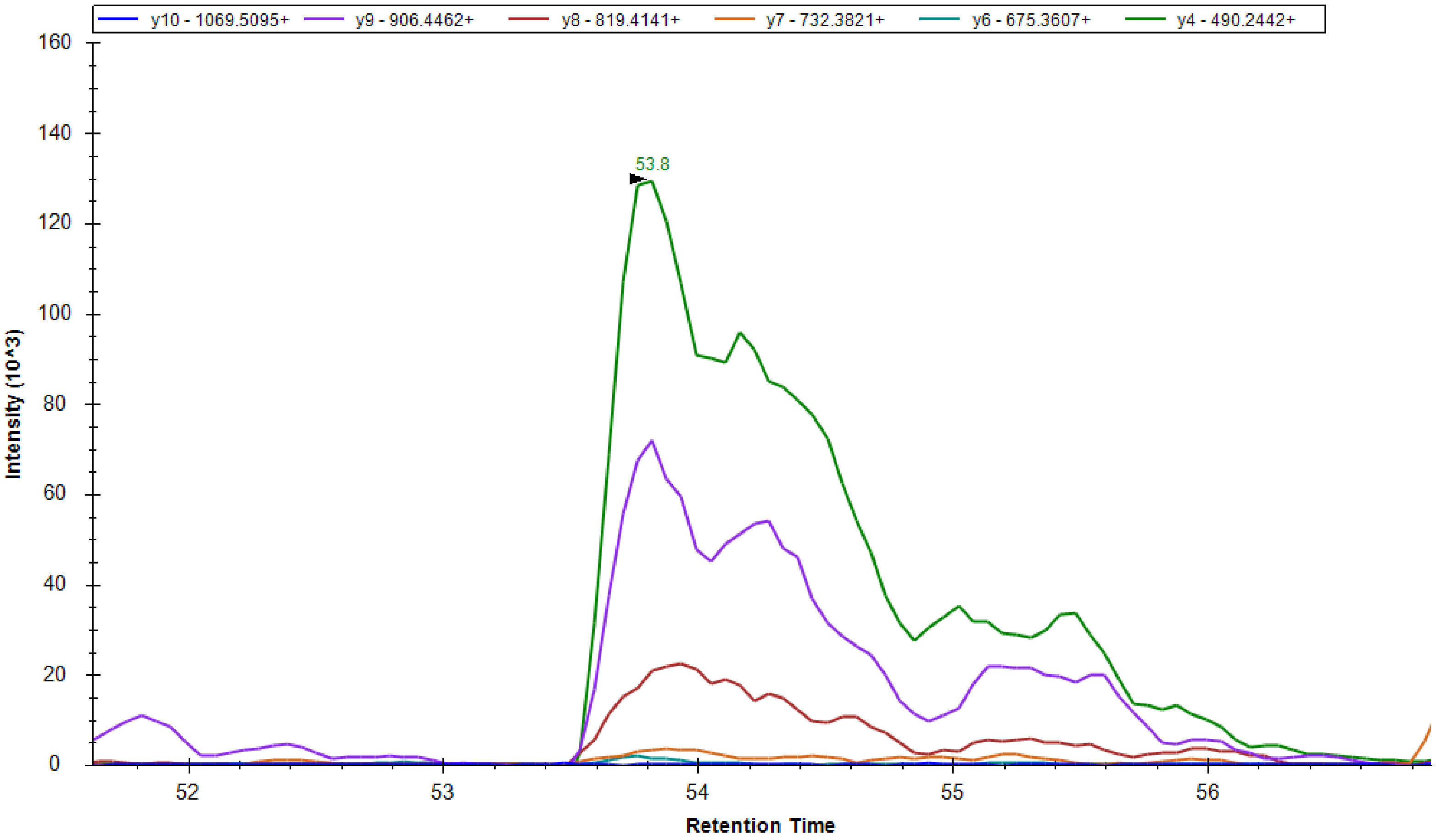

File name: 391-new#B-Round02_Negative-screen.skyd

Parent ion m/z and charges: 653.9854+++

NR_002803.2.1
MWLSQPMGQTCR

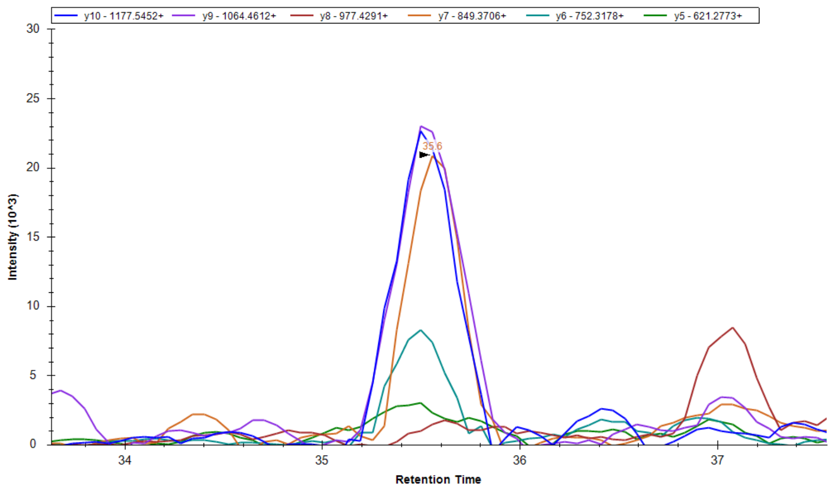

File name: 391-new#B-Round01_All-screening_Positive result.skyd

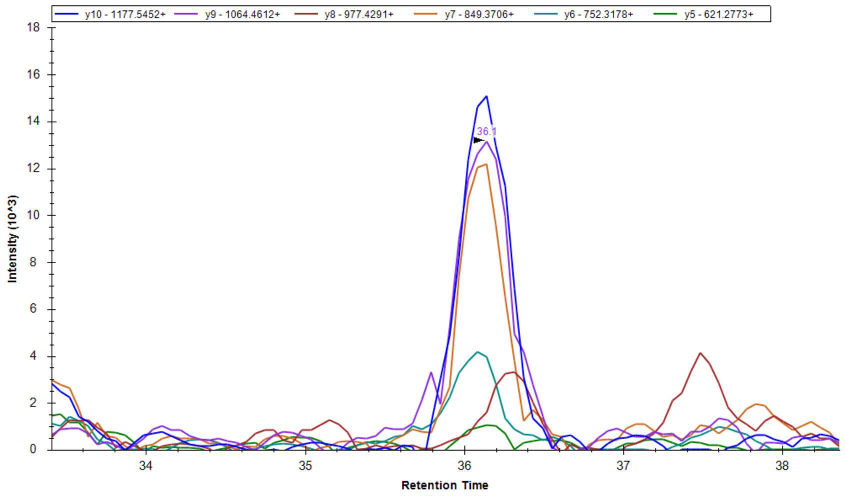

File name: 391-new#B-Round01_Positive-confirm.skyd

Parent ion m/z and charges: 747.8362++

NR_002803.2.1
MWLSQPMGQTCR

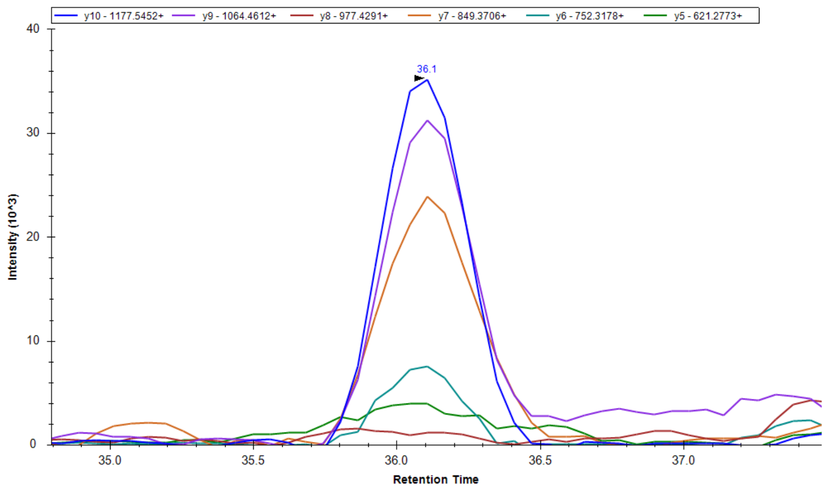

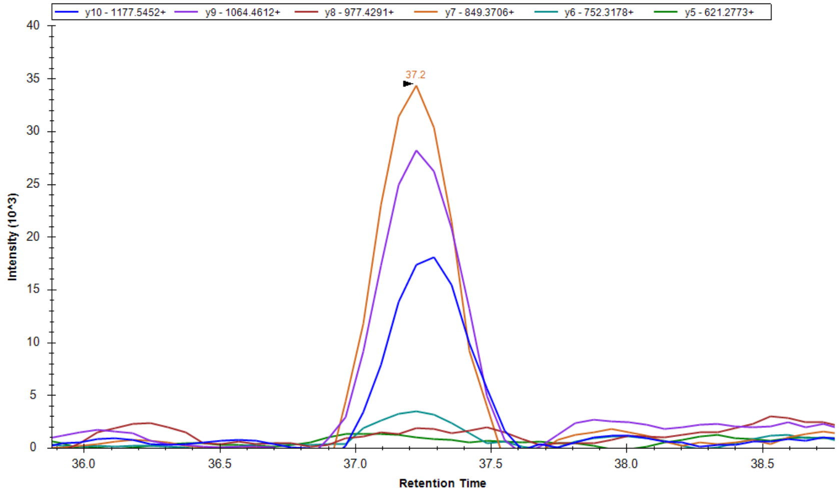

File name: 391-new#A-Round01_All-screening_Positive result.skyd

File name: 391-new#A-Round01_Positive-confirm.skyd

Parent ion m/z and charges: 747.8362++

NR_002930.1.4
PLPVSAVPGR

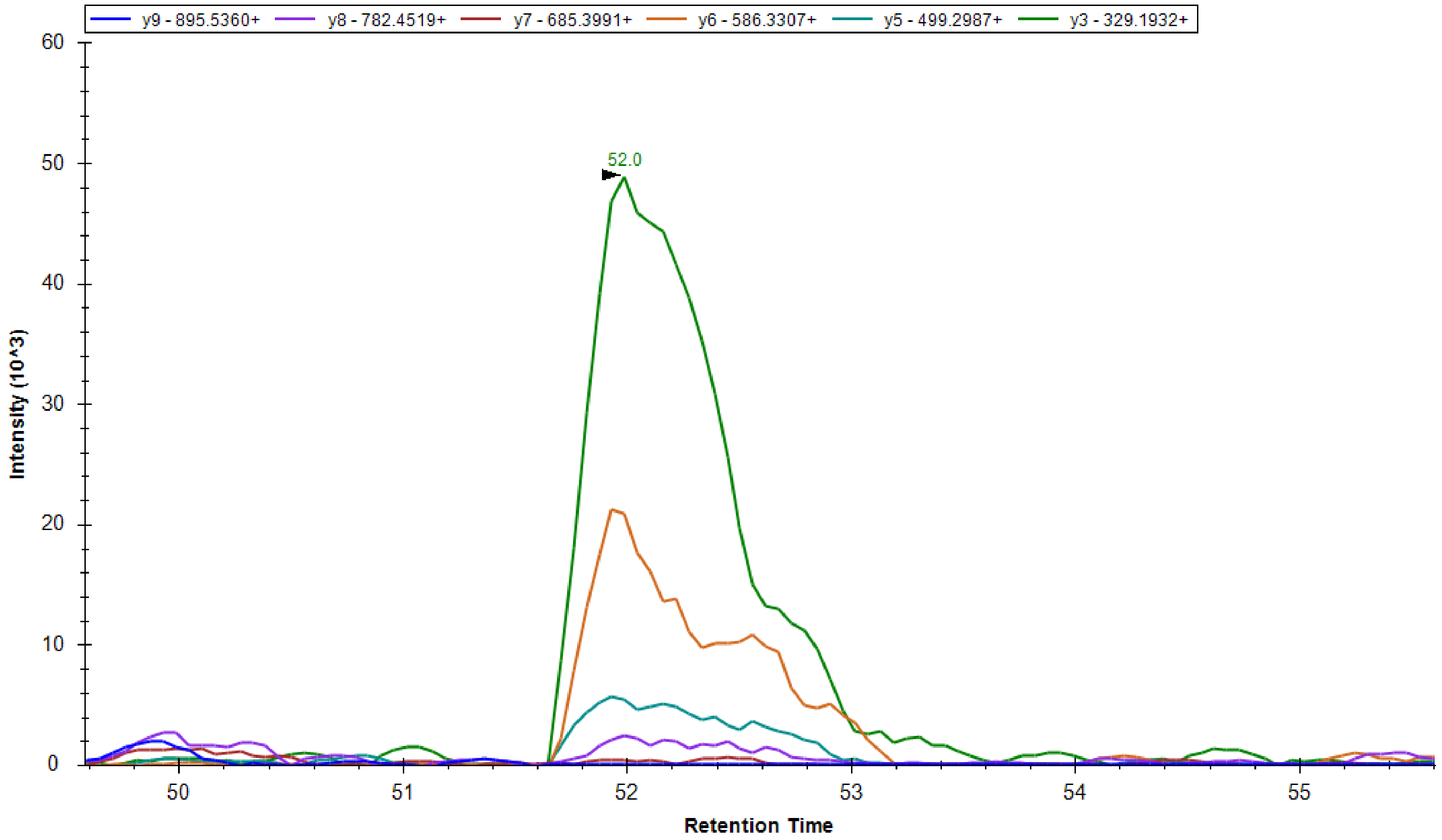

File name: 391-new#B-Round02_Negative-screen.skyd

Parent ion m/z and charges: 496.7980++

NR_002947.3.1
AETVSCEVARVFPAK

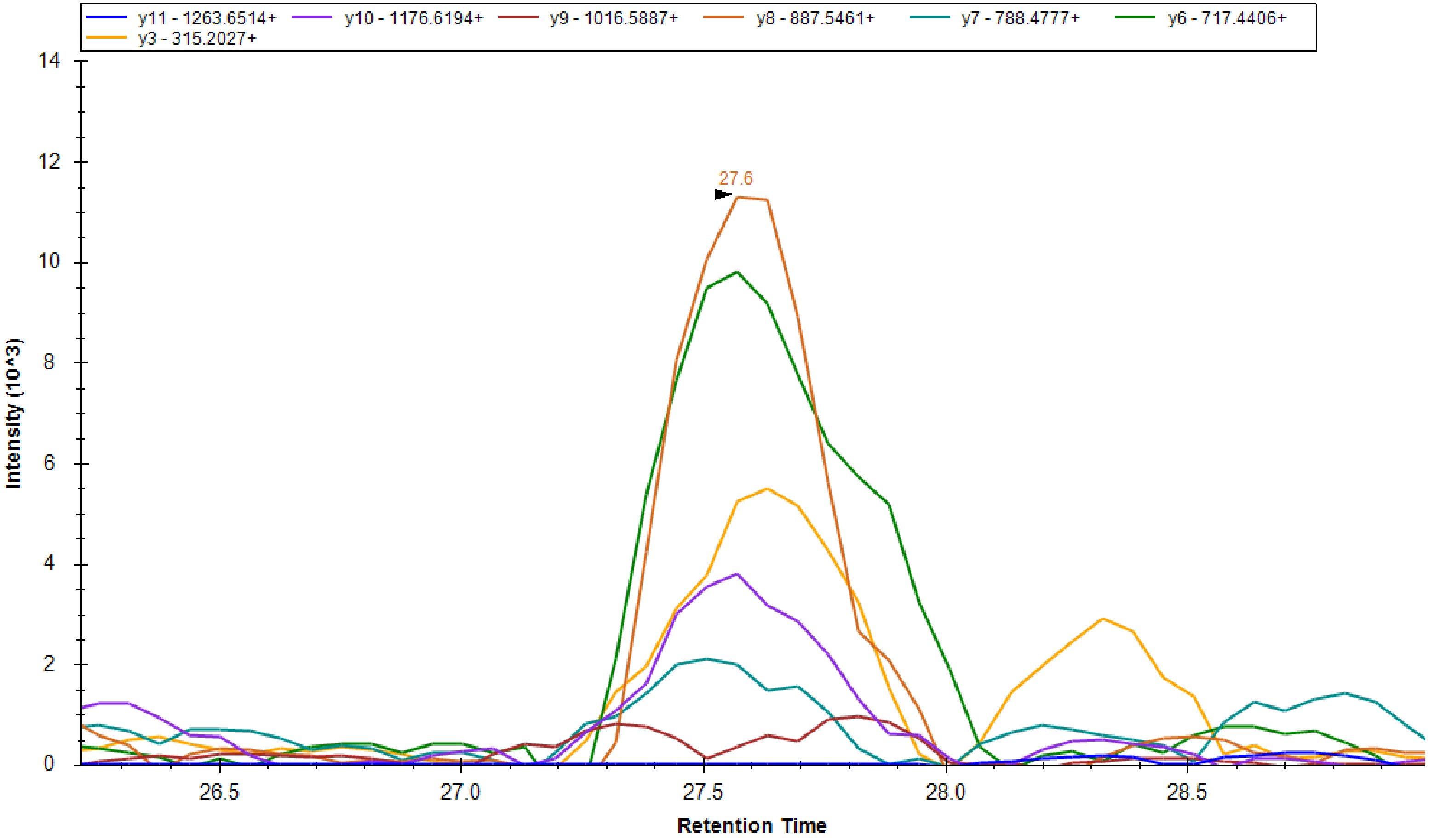

File name: 391-new#A-Round02_Negative-screen.skyd

Parent ion m/z and charges: 832.4272++

NR_003089.2.3
MYRGFSVVVNGPISPPSSCHMTPPHVLLK

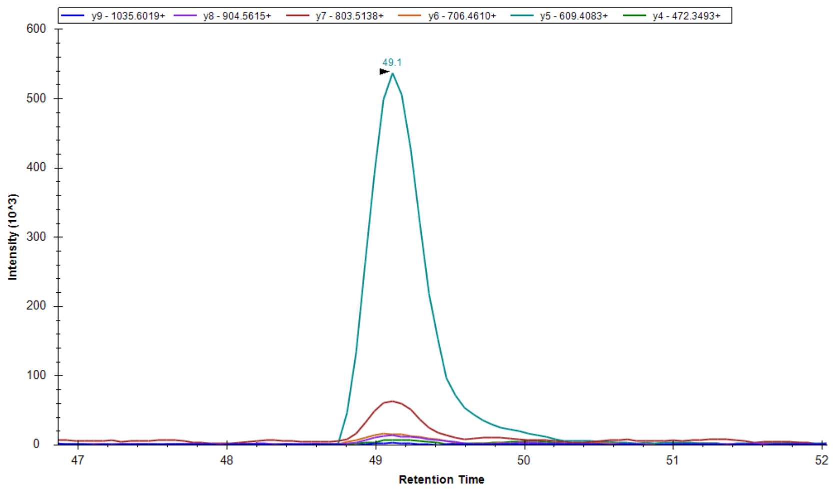

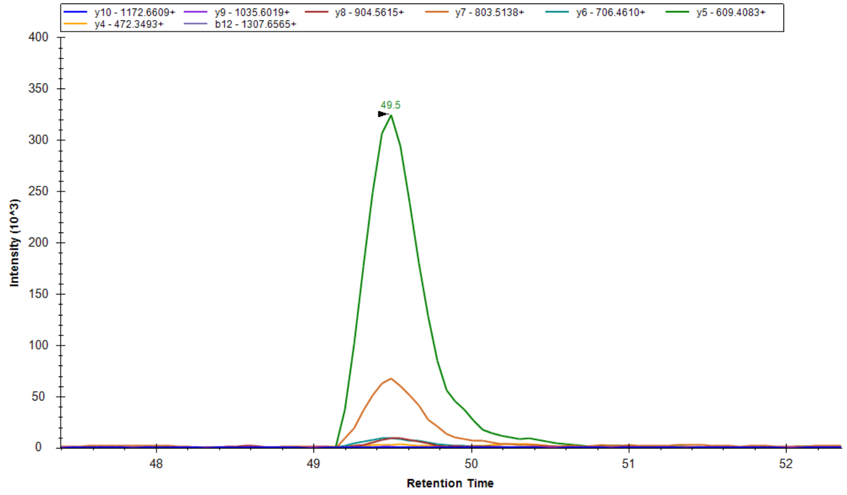

File name: 391-new#B-Round01_All-screening_Positive result.skyd

File name: 391-new#B-Round01_Positive-confirm.skyd

Parent ion m/z and charges: 802.6621++++

NR_003089.2.3
MYRGFSVVVNGPISPPSSCHMTPPHVLLK

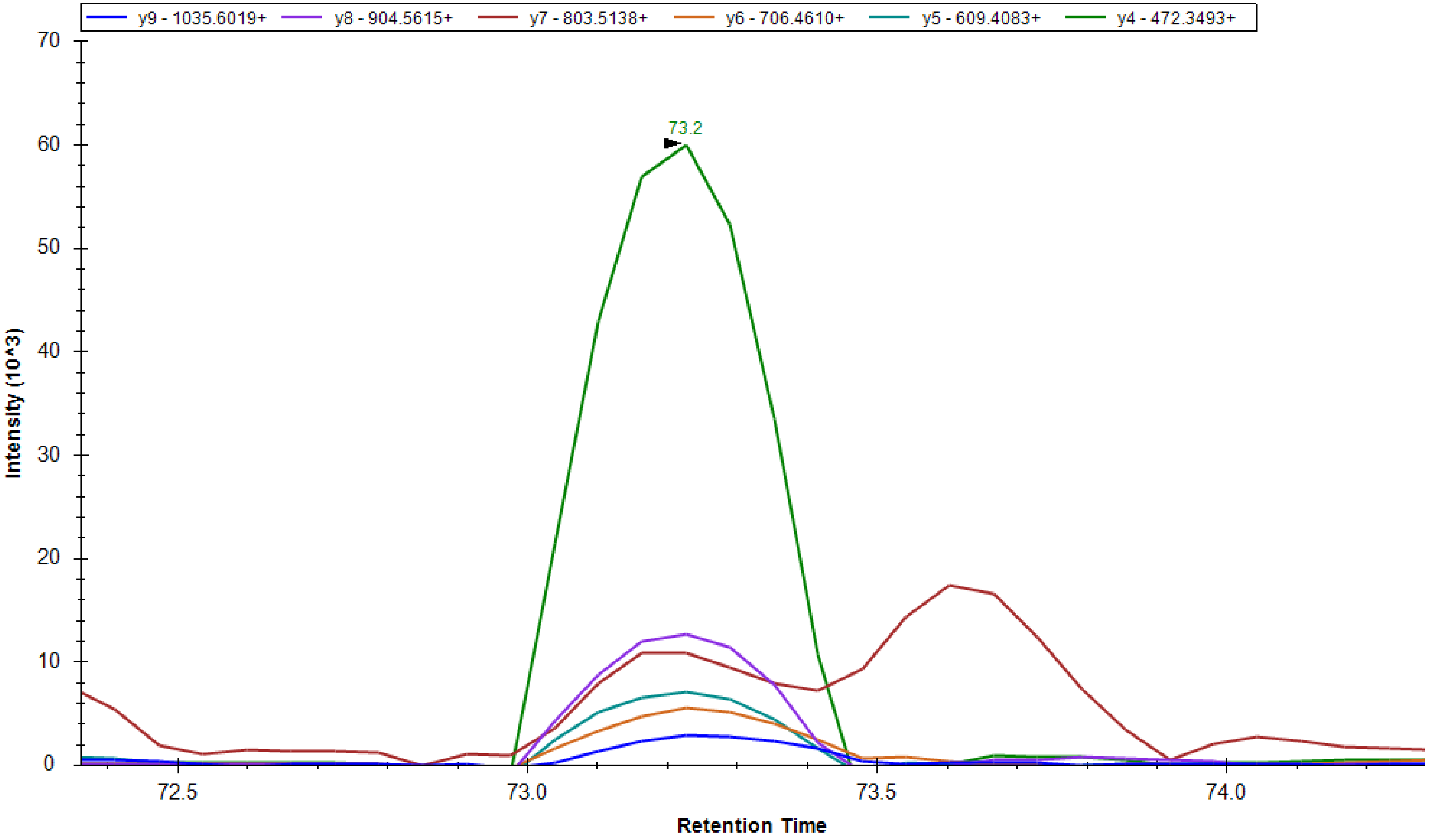

File name: 391-new#A-Round02_Negative-screen.skyd

Parent ion m/z and charges: 802.6621++++

NR_003225.2.7
MMLPSTLTHASMR

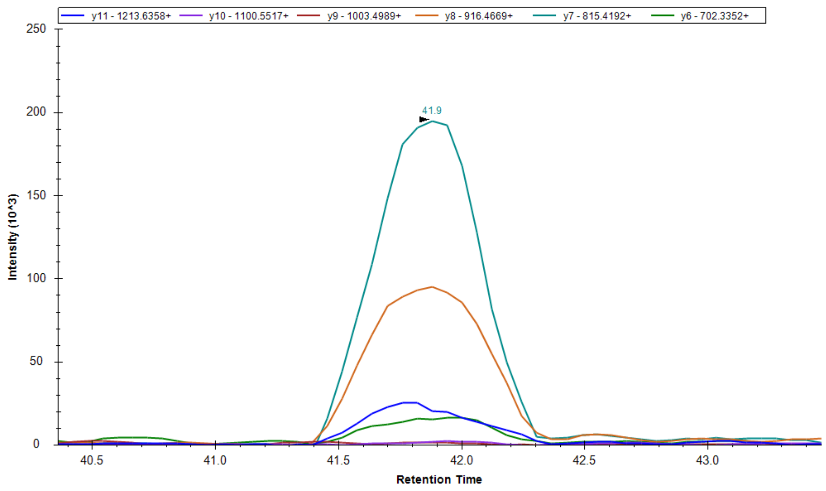

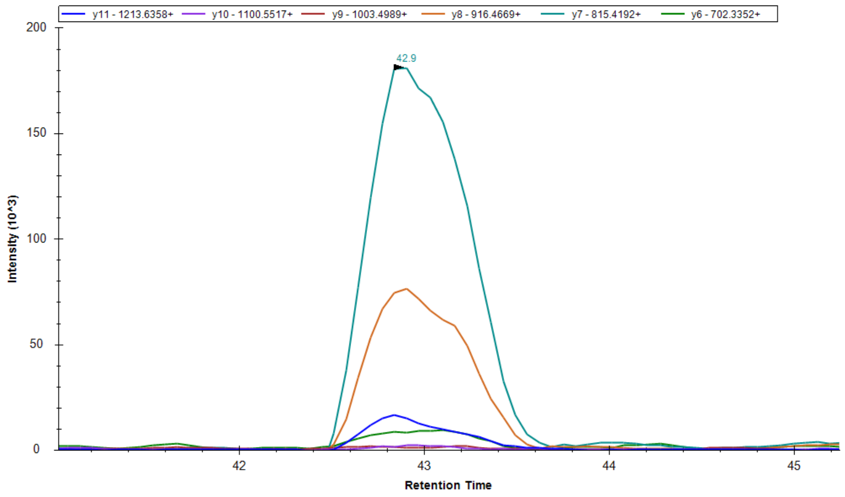

File name: 391-new#A-Round01_All-screening_Positive result.skyd

File name: 391-new#A-Round01_Positive-confirm.skyd

Parent ion m/z and charges: 738.3620++

NR_003225.2.7
MMLPSTLTHASMR

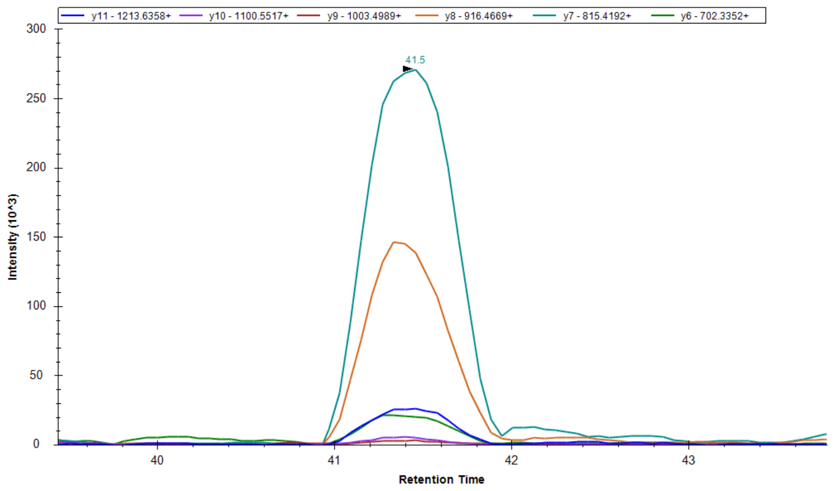

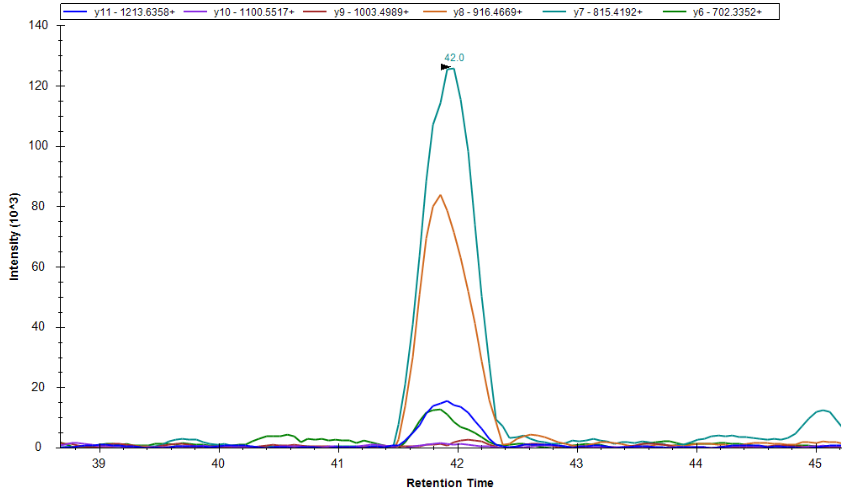

File name: 391-new#B-Round01_All-screening_Positive result.skyd

File name: 391-new#B-Round01_Positive-confirm.skyd

Parent ion m/z and charges: 738.3620++

NR_003255.1.49
EGGSIPIVKEASPEK

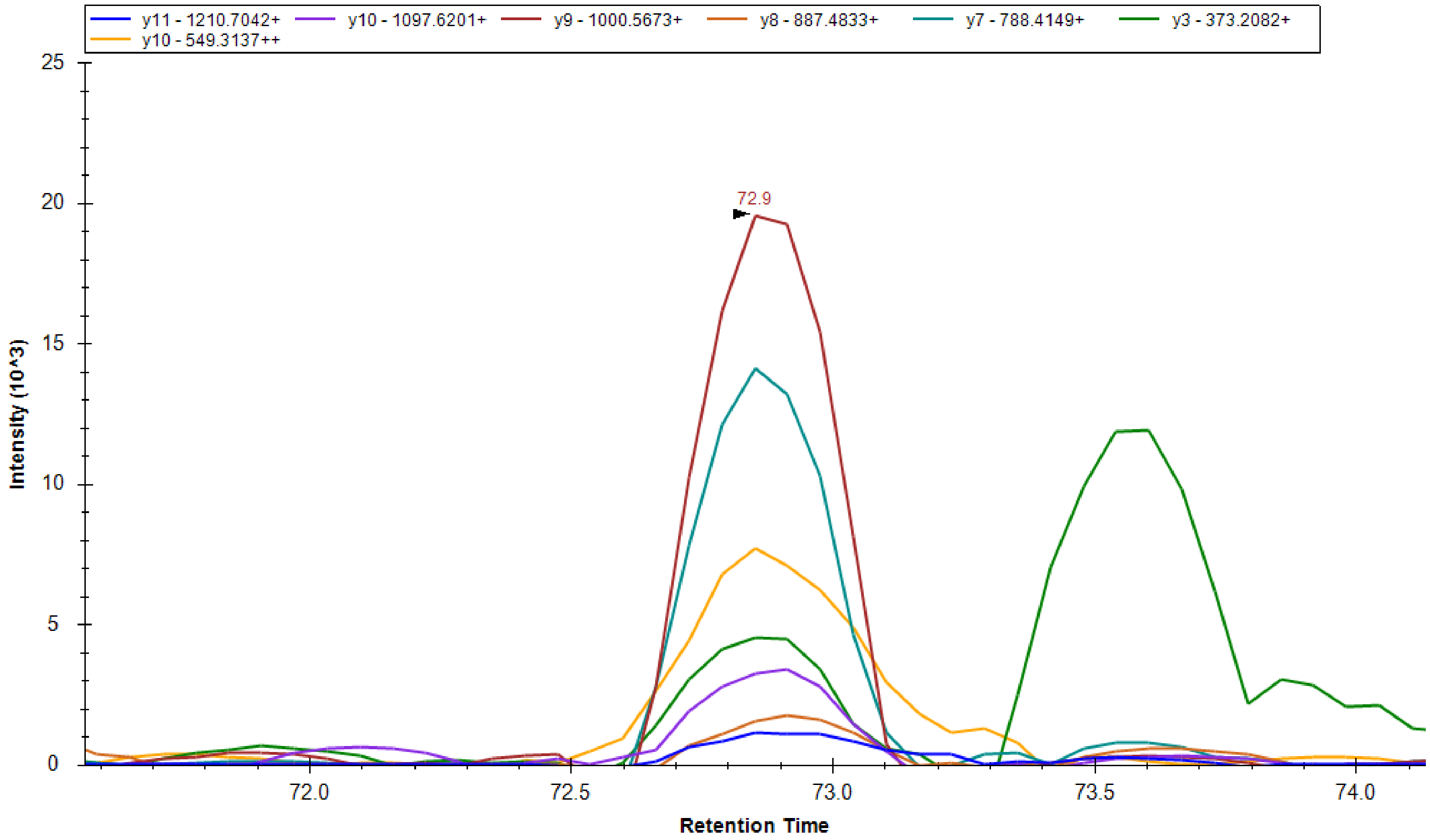

File name: 391-new#A-Round02_Negative-screen.skyd

Parent ion m/z and charges: 770.9145++

NR_003255.1.49
EGGSIPIVKEASPEK

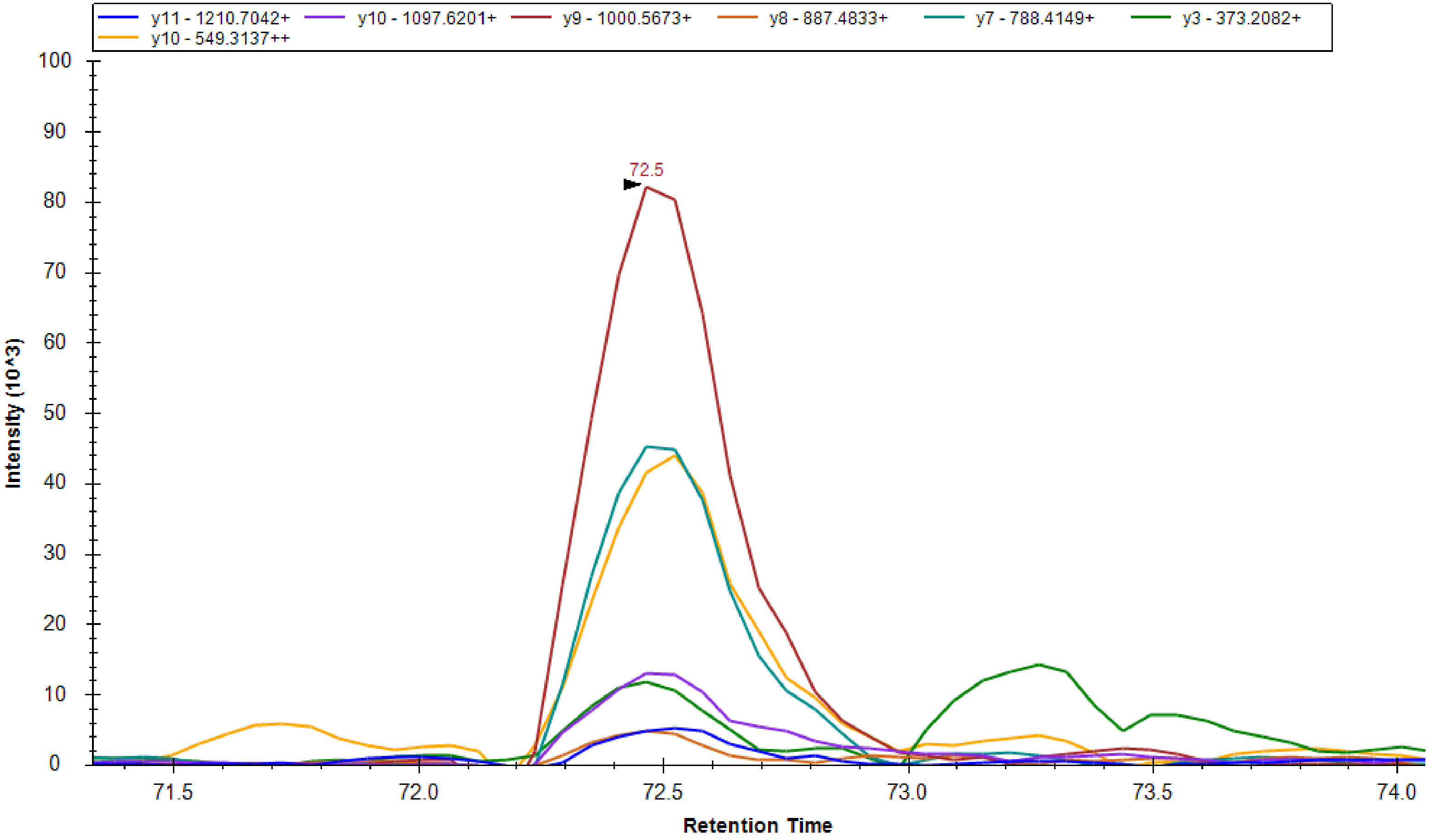

File name: 391-new#B-Round02_Negative-screen.skyd

Parent ion m/z and charges: 770.9145++

NR_027504.1.3
VWTTTIAGIITAPSGHGATIR

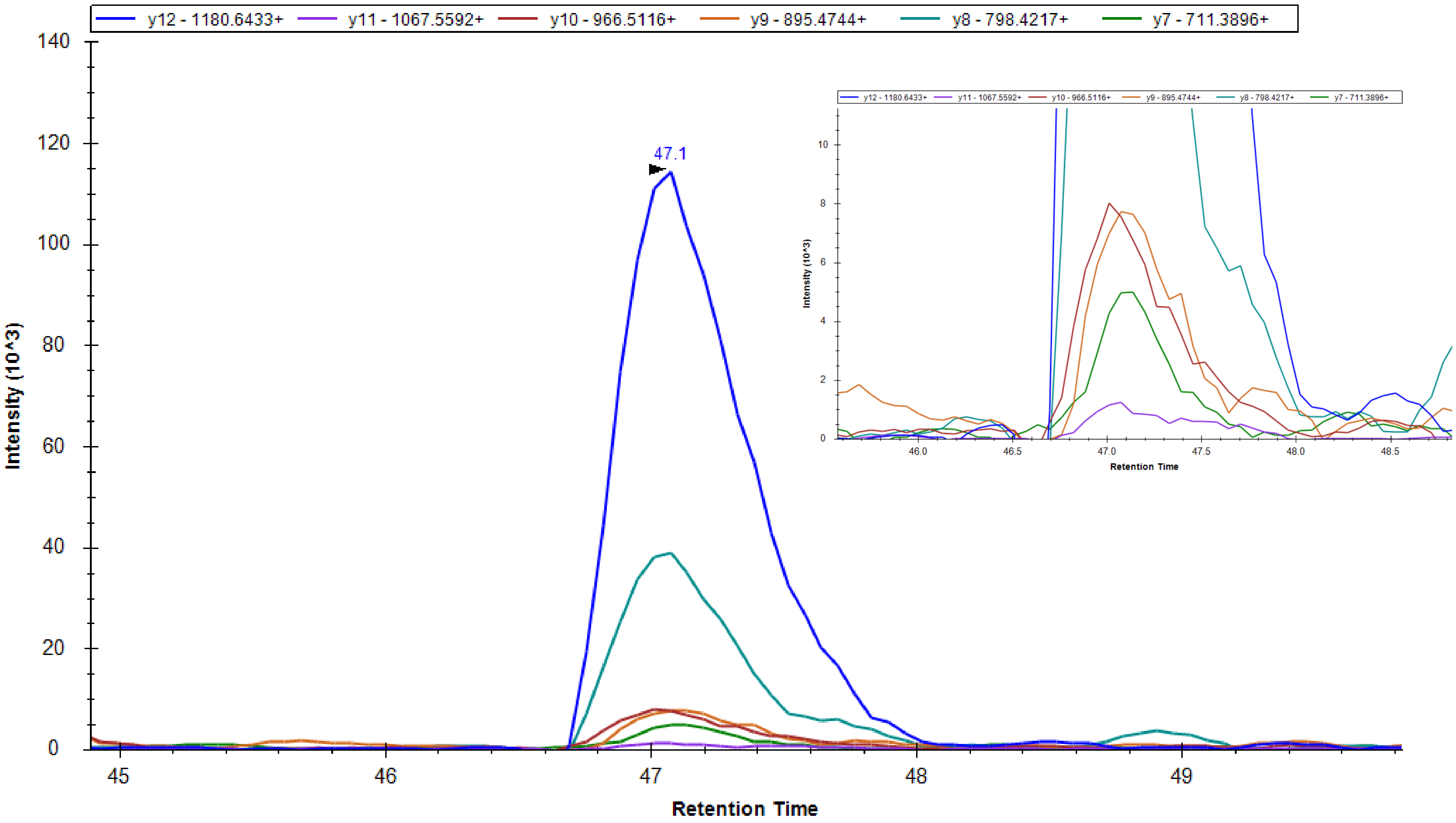

File name: 391-new#A-Round02_Negative-screen.skyd

Parent ion m/z and charges: 708.3918+++

NR_027504.1.3
VWTTTIAGIITAPSGHGATIR

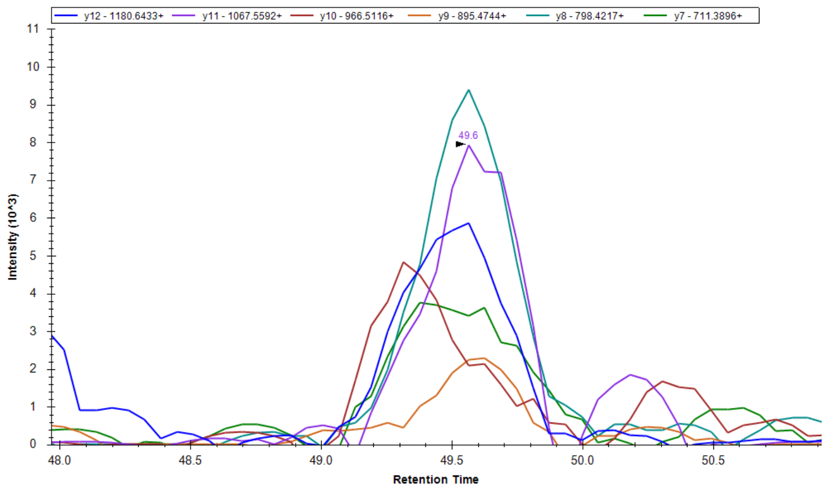

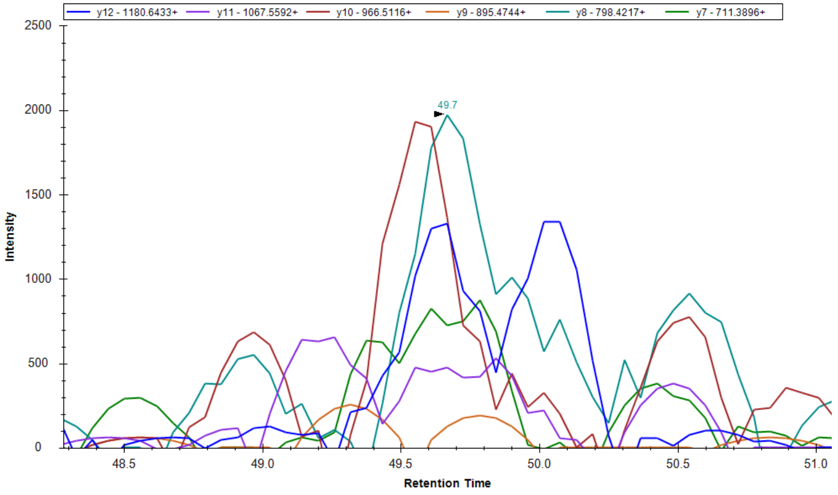

File name: 391-new#B-Round01_All-screening_Positive result.skyd

File name: 391-new#B-Round01_Positive-confirm.skyd

Parent ion m/z and charges: 1062.0840++
