# Supplementary material for: A hidden human proteome encoded by ‘non-coding’ genes
Source: Nucleic Acids Res. 2019 Jul 24;47(15):8111–25. doi: 10.1093/nar/gkz646 (PMC6735797; doi:10.1093/nar/gkz646)
Supplement: gkz646_Supplemental_Files [file gkz646_supplemental_files.zip › Supplementary Fig S3_Peptide spectra information for the PRM MS.pdf]

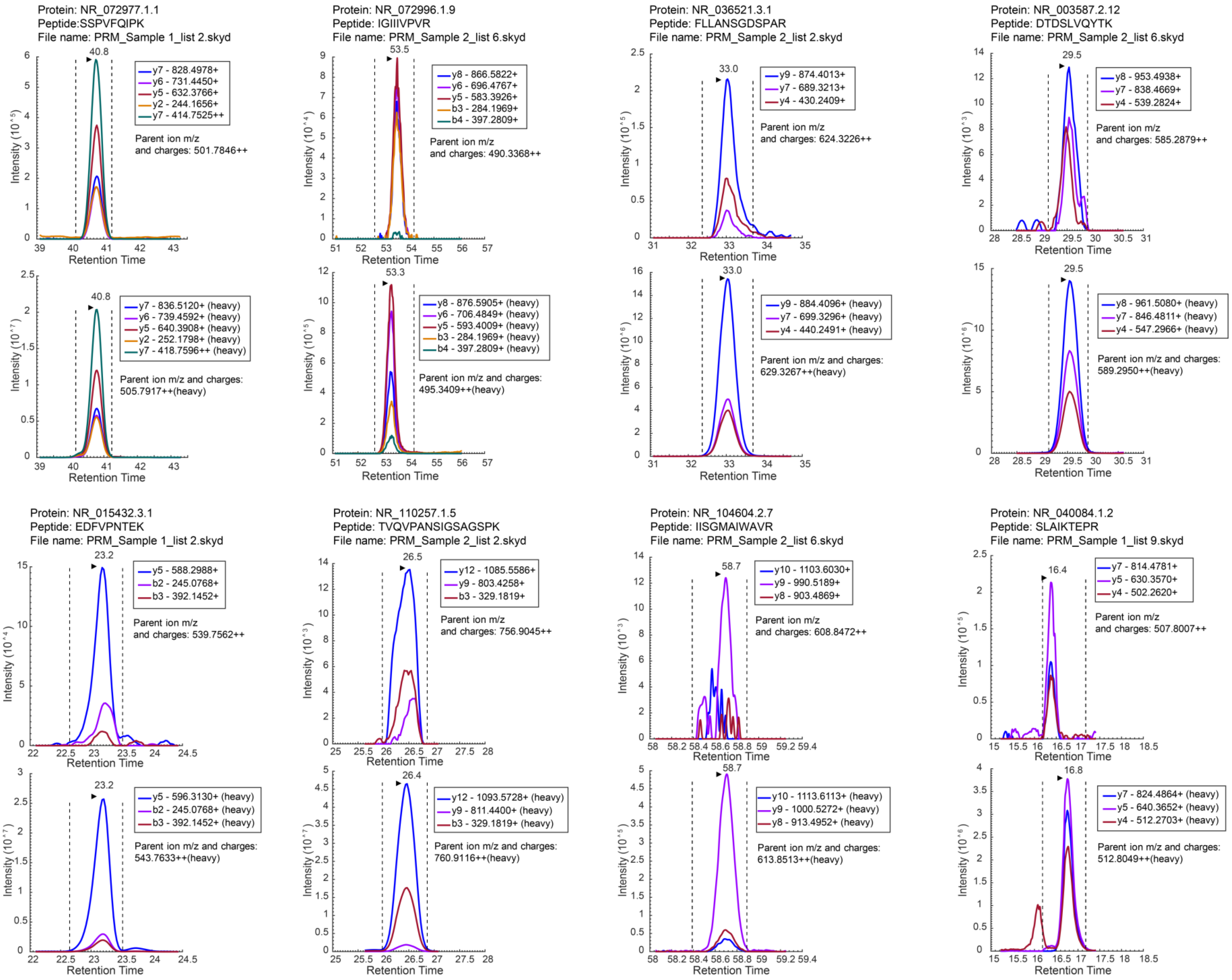
Protein: NR_072977.1.1
Peptide:SSPVFQIPK
File name: PRM_Sample 1_list 2.skyd
y7 - 828.4978+
y6 - 731.4450+
y5 - 632.3766+
y2 - 244.1656+
y7 - 414.7525++
Parent ion m/z and charges: 501.7846++
y7 - 836.5120+ (heavy)
y6 - 739.4592+ (heavy)
y5 - 640.3908+ (heavy)
y2 - 252.1798+ (heavy)
y7 - 418.7596++ (heavy)
Parent ion m/z and charges: 505.7917++(heavy)
Protein: NR_072996.1.9
Peptide: IGIIIVPVR
File name: PRM_Sample 2_list 6.skyd
y8 - 866.5822+
y6 - 696.4767+
y5 - 583.3926+
b3 - 284.1969+
b4 - 397.2809+
Parent ion m/z and charges: 490.3368++
y8 - 876.5905+ (heavy)
y6 - 706.4849+ (heavy)
y5 - 593.4009+ (heavy)
b3 - 284.1969+ (heavy)
b4 - 397.2809+ (heavy)
Parent ion m/z and charges: 495.3409++(heavy)
Protein: NR_036521.3.1
Peptide: FLLANSGDSPAR
File name: PRM_Sample 2_list 2.skyd
y9 - 874.4013+
y7 - 689.3213+
y4 - 430.2409+
Parent ion m/z and charges: 624.3226++
y9 - 884.4096+ (heavy)
y7 - 699.3296+ (heavy)
y4 - 440.2491+ (heavy)
Parent ion m/z and charges: 629.3267++(heavy)
Protein: NR_003587.2.12
Peptide: DTDSLVQYTK
File name: PRM_Sample 2_list 6.skyd
y8 - 953.4938+
y7 - 838.4669+
y4 - 539.2824+
Parent ion m/z and charges: 585.2879++
y8 - 961.5080+ (heavy)
y7 - 846.4811+ (heavy)
y4 - 547.2966+ (heavy)
Parent ion m/z and charges: 589.2950++(heavy)
Protein: NR_015432.3.1
Peptide: EDFVPNTEK
File name: PRM_Sample 1_list 2.skyd
y5 - 588.2988+
b2 - 245.0768+
b3 - 392.1452+
Parent ion m/z and charges: 539.7562++
y5 - 596.3130+ (heavy)
b2 - 245.0768+ (heavy)
b3 - 392.1452+ (heavy)
Parent ion m/z and charges: 543.7633++(heavy)
Protein: NR_110257.1.5
Peptide: TVQVPANSIGSAGSPK
File name: PRM_Sample 2_list 2.skyd
y12 - 1085.5586+
y9 - 803.4258+
b3 - 329.1819+
Parent ion m/z and charges: 756.9045++
y12 - 1093.5728+ (heavy)
y9 - 811.4400+ (heavy)
b3 - 329.1819+ (heavy)
Parent ion m/z and charges: 760.9116++(heavy)
Protein: NR_104604.2.7
Peptide: IISGMAIWAVR
File name: PRM_Sample 2_list 6.skyd
y10 - 1103.6030+
y9 - 990.5189+
y8 - 903.4869+
Parent ion m/z and charges: 608.8472++
y10 - 1113.6113+ (heavy)
y9 - 1000.5272+ (heavy)
y8 - 913.4952+ (heavy)
Parent ion m/z and charges: 613.8513++(heavy)
Protein: NR_040084.1.2
Peptide: SLAIKTEPR
File name: PRM_Sample 1_list 9.skyd
y7 - 814.4781+
y5 - 630.3570+
y4 - 502.2620+
Parent ion m/z and charges: 507.8007++
y7 - 824.4864+ (heavy)
y5 - 640.3652+ (heavy)
y4 - 512.2703+ (heavy)
Parent ion m/z and charges: 512.8049++(heavy)
Intensity
Retention Time
