# Supplementary material for: A hidden human proteome encoded by ‘non-coding’ genes
Source: Nucleic Acids Res. 2019 Jul 24;47(15):8111–25. doi: 10.1093/nar/gkz646 (PMC6735797; doi:10.1093/nar/gkz646)
Supplement: gkz646_Supplemental_Files [file gkz646_supplemental_files.zip › Supplementary Fig S4_Peptide spectra information for the Heavy-MRM MS.pdf]

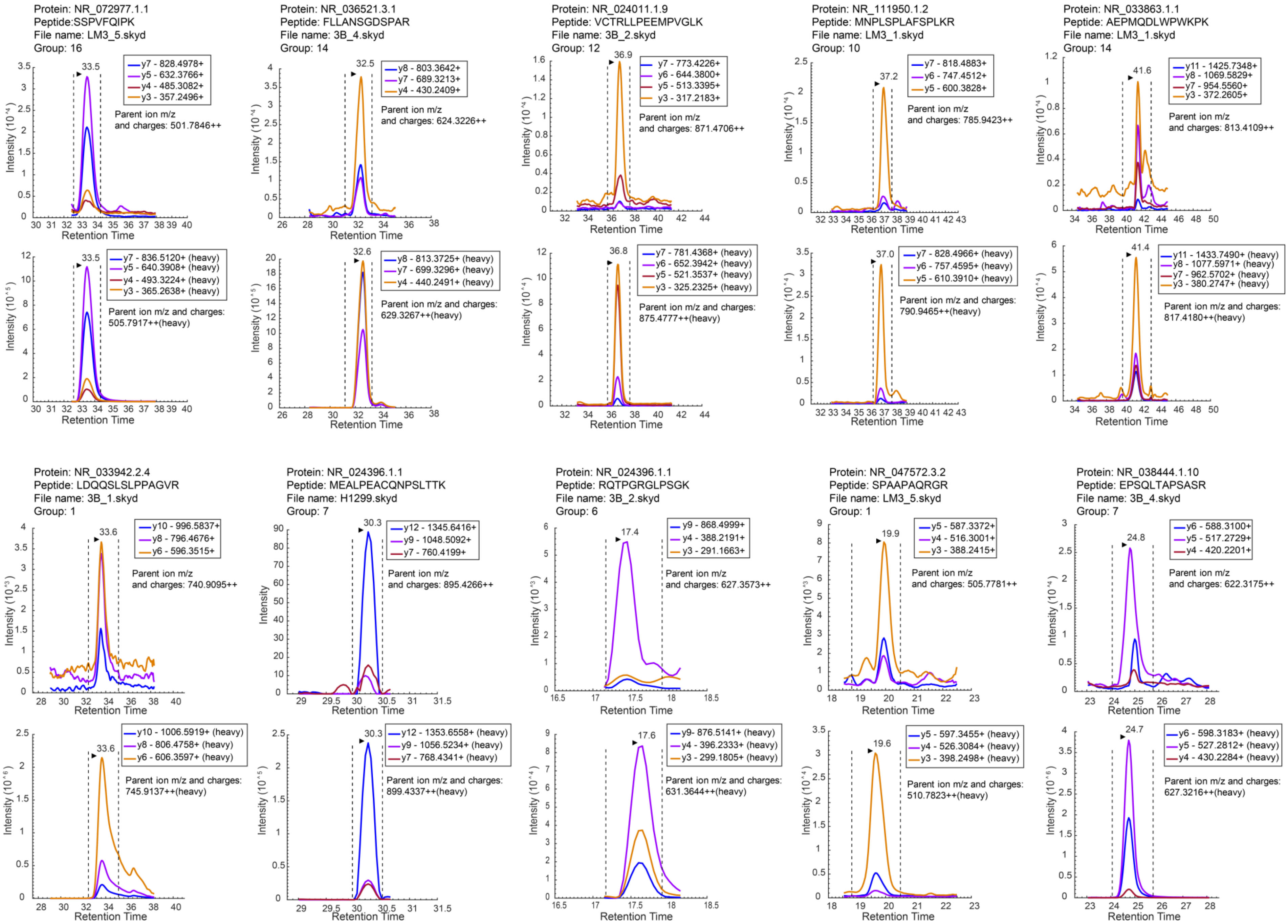
Protein: NR_072977.1.1
Peptide:SSPVFQIPK
File name: LM3_5.skyd
Group: 16
y7 - 828.4978+
y5 - 632.3766+
y4 - 485.3082+
y3 - 357.2496+
Parent ion m/z and charges: 501.7846++
y7 - 836.5120+ (heavy)
y5 - 640.3908+ (heavy)
y4 - 493.3224+ (heavy)
y3 - 365.2638+ (heavy)
Parent ion m/z and charges: 505.7917++(heavy)
Protein: NR_036521.3.1
Peptide: FLLANSGDSPAR
File name: 3B_4.skyd
Group: 14
y8 - 803.3642+
y7 - 689.3213+
y4 - 430.2409+
Parent ion m/z and charges: 624.3226++
y8 - 813.3725+ (heavy)
y7 - 699.3296+ (heavy)
y4 - 440.2491+ (heavy)
Parent ion m/z and charges: 629.3267++(heavy)
Protein: NR_024011.1.9
Peptide: VCTRLLPEEMPVGLK
File name: 3B_2.skyd
Group: 12
y7 - 773.4226+
y6 - 644.3800+
y5 - 513.3395+
y3 - 317.2183+
Parent ion m/z and charges: 871.4706++
y7 - 781.4368+ (heavy)
y6 - 652.3942+ (heavy)
y5 - 521.3537+ (heavy)
y3 - 325.2325+ (heavy)
Parent ion m/z and charges: 875.4777++(heavy)
Protein: NR_111950.1.2
Peptide: MNPLSPLAFSPLKR
File name: LM3_1.skyd
Group: 10
y7 - 818.4883+
y6 - 747.4512+
y5 - 600.3828+
Parent ion m/z and charges: 785.9423++
y7 - 828.4966+ (heavy)
y6 - 757.4595+ (heavy)
y5 - 610.3910+ (heavy)
Parent ion m/z and charges: 790.9465++(heavy)
Protein: NR_033863.1.1
Peptide: AEPMQDLWPWKPK
File name: LM3_1.skyd
Group: 14
y11 - 1425.7348+
y8 - 1069.5829+
y7 - 954.5560+
y3 - 372.2605+
Parent ion m/z and charges: 813.4109++
y11 - 1433.7490+ (heavy)
y8 - 1077.5971+ (heavy)
y7 - 962.5702+ (heavy)
y3 - 380.2747+ (heavy)
Parent ion m/z and charges: 817.4180++(heavy)
Protein: NR_033942.2.4
Peptide: LDQQSLSLPPAGVR
File name: 3B_1.skyd
Group: 1
y10 - 996.5837+
y8 - 796.4676+
y6 - 596.3515+
Parent ion m/z and charges: 740.9095++
y10 - 1006.5919+ (heavy)
y8 - 806.4758+ (heavy)
y6 - 606.3597+ (heavy)
Parent ion m/z and charges: 745.9137++(heavy)
Protein: NR_024396.1.1
Peptide: MEALPEACQNPSLTTK
File name: H1299.skyd
Group: 7
y12 - 1345.6416+
y9 - 1048.5092+
y7 - 760.4199+
Parent ion m/z and charges: 895.4266++
y12 - 1353.6558+ (heavy)
y9 - 1056.5234+ (heavy)
y7 - 768.4341+ (heavy)
Parent ion m/z and charges: 899.4337++(heavy)
Protein: NR_024396.1.1
Peptide: RQTPGRGLPSGK
File name: 3B_2.skyd
Group: 6
y9 - 868.4999+
y4 - 388.2191+
y3 - 291.1663+
Parent ion m/z and charges: 627.3573++
y9- 876.5141+ (heavy)
y4 - 396.2333+ (heavy)
y3 - 299.1805+ (heavy)
Parent ion m/z and charges: 631.3644++(heavy)
Protein: NR_047572.3.2
Peptide: SPAAPAQRGR
File name: LM3_5.skyd
Group: 1
y5 - 587.3372+
y4 - 516.3001+
y3 - 388.2415+
Parent ion m/z and charges: 505.7781++
y5 - 597.3455+ (heavy)
y4 - 526.3084+ (heavy)
y3 - 398.2498+ (heavy)
Parent ion m/z and charges: 510.7823++(heavy)
Protein: NR_038444.1.10
Peptide: EPSQLTAPSASR
File name: 3B_4.skyd
Group: 7
y6 - 588.3100+
y5 - 517.2729+
y4 - 420.2201+
Parent ion m/z and charges: 622.3175++
y6 - 598.3183+ (heavy)
y5 - 527.2812+ (heavy)
y4 - 430.2284+ (heavy)
Parent ion m/z and charges: 627.3216++(heavy)
Intensity
Retention Time

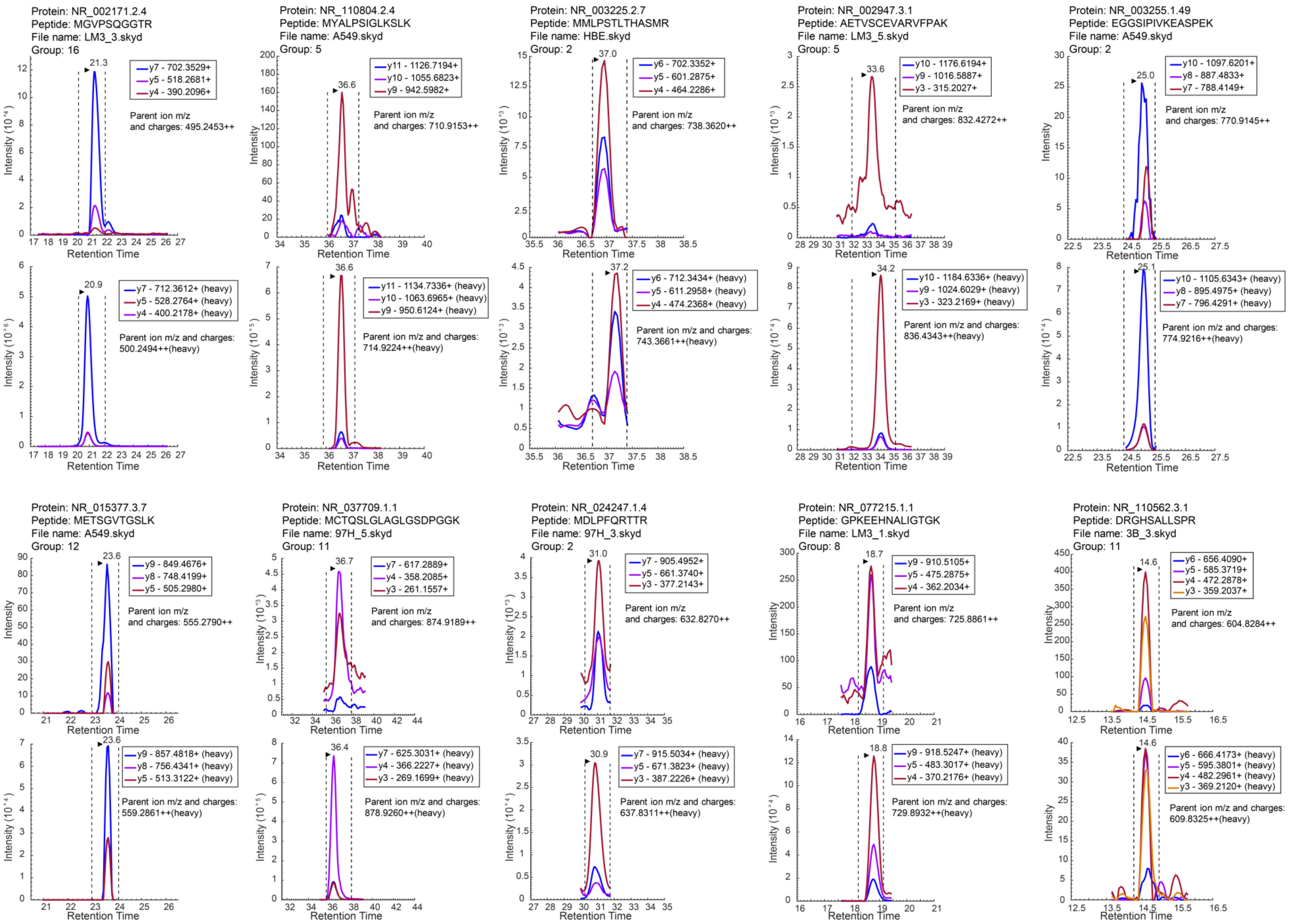
Protein: NR_002171.2.4
Peptide: MGVPSQGGTR
File name: LM3_3.skyd
Group: 16
21.3
y7 - 702.3529+
y5 - 518.2681+
y4 - 390.2096+
Parent ion m/z
and charges: 495.2453++
Intensity (10^4)
Retention Time
20.9
y7 - 712.3612+ (heavy)
y5 - 528.2764+ (heavy)
y4 - 400.2178+ (heavy)
Parent ion m/z and charges:
500.2494++(heavy)
Intensity (10^6)
Retention Time
Protein: NR_110804.2.4
Peptide: MYALPSIGLKSLK
File name: A549.skyd
Group: 5
36.6
y11 - 1126.7194+
y10 - 1055.6823+
y9 - 942.5982+
Parent ion m/z
and charges: 710.9153++
Intensity
Retention Time
36.6
y11 - 1134.7336+ (heavy)
y10 - 1063.6965+ (heavy)
y9 - 950.6124+ (heavy)
Parent ion m/z and charges:
714.9224++(heavy)
Intensity (10^5)
Retention Time
Protein: NR_003225.2.7
Peptide: MMLPSTLTHASMR
File name: HBE.skyd
Group: 2
37.0
y6 - 702.3352+
y5 - 601.2875+
y4 - 464.2286+
Parent ion m/z
and charges: 738.3620++
Intensity (10^3)
Retention Time
37.2
y6 - 712.3434+ (heavy)
y5 - 611.2958+ (heavy)
y4 - 474.2368+ (heavy)
Parent ion m/z and charges:
743.3661++(heavy)
Intensity (10^3)
Retention Time
Protein: NR_002947.3.1
Peptide: AETVSCEVARVFPAK
File name: LM3_5.skyd
Group: 5
33.6
y10 - 1176.6194+
y9 - 1016.5887+
y3 - 315.2027+
Parent ion m/z
and charges: 832.4272++
Intensity (10^3)
Retention Time
34.2
y10 - 1184.6336+ (heavy)
y9 - 1024.6029+ (heavy)
y3 - 323.2169+ (heavy)
Parent ion m/z and charges:
836.4343++(heavy)
Intensity (10^4)
Retention Time
Protein: NR_003255.1.49
Peptide: EGGSIPIVKEASPEK
File name: A549.skyd
Group: 2
25.0
y10 - 1097.6201+
y8 - 887.4833+
y7 - 788.4149+
Parent ion m/z
and charges: 770.9145++
Intensity
Retention Time
25.1
y10 - 1105.6343+ (heavy)
y8 - 895.4975+ (heavy)
y7 - 796.4291+ (heavy)
Parent ion m/z and charges:
774.9216++(heavy)
Intensity (10^4)
Retention Time
Protein: NR_015377.3.7
Peptide: METSGVTGSLK
File name: A549.skyd
Group: 12
23.6
y9 - 849.4676+
y8 - 748.4199+
y5 - 505.2980+
Parent ion m/z
and charges: 555.2790++
Intensity
Retention Time
23.6
y9 - 857.4818+ (heavy)
y8 - 756.4341+ (heavy)
y5 - 513.3122+ (heavy)
Parent ion m/z and charges:
559.2861++(heavy)
Intensity (10^4)
Retention Time
Protein: NR_037709.1.1
Peptide: MCTQSLGLAGLGSDPGGK
File name: 97H_5.skyd
Group: 11
36.7
y7 - 617.2889+
y4 - 358.2085+
y3 - 261.1557+
Parent ion m/z
and charges: 874.9189++
Intensity (10^3)
Retention Time
36.4
y7 - 625.3031+ (heavy)
y4 - 366.2227+ (heavy)
y3 - 269.1699+ (heavy)
Parent ion m/z and charges:
878.9260++(heavy)
Intensity (10^5)
Retention Time
Protein: NR_024247.1.4
Peptide: MDLPFQRTTR
File name: 97H_3.skyd
Group: 2
31.0
y7 - 905.4952+
y5 - 661.3740+
y3 - 377.2143+
Parent ion m/z
and charges: 632.8270++
Intensity (10^3)
Retention Time
30.9
y7 - 915.5034+ (heavy)
y5 - 671.3823+ (heavy)
y3 - 387.2226+ (heavy)
Parent ion m/z and charges:
637.8311++(heavy)
Intensity (10^4)
Retention Time
Protein: NR_077215.1.1
Peptide: GPKEEHNALIGTGK
File name: LM3_1.skyd
Group: 8
18.7
y9 - 910.5105+
y5 - 475.2875+
y4 - 362.2034+
Parent ion m/z
and charges: 725.8861++
Intensity
Retention Time
18.8
y9 - 918.5247+ (heavy)
y5 - 483.3017+ (heavy)
y4 - 370.2176+ (heavy)
Parent ion m/z and charges:
729.8932++(heavy)
Intensity (10^4)
Retention Time
Protein: NR_110562.3.1
Peptide: DRGHSALLSPR
File name: 3B_3.skyd
Group: 11
14.6
y6 - 656.4090+
y5 - 585.3719+
y4 - 472.2878+
y3 - 359.2037+
Parent ion m/z
and charges: 604.8284++
Intensity
Retention Time
14.6
y6 - 666.4173+ (heavy)
y5 - 595.3801+ (heavy)
y4 - 482.2961+ (heavy)
y3 - 369.2120+ (heavy)
Parent ion m/z and charges:
609.8325++(heavy)
Intensity
Retention Time

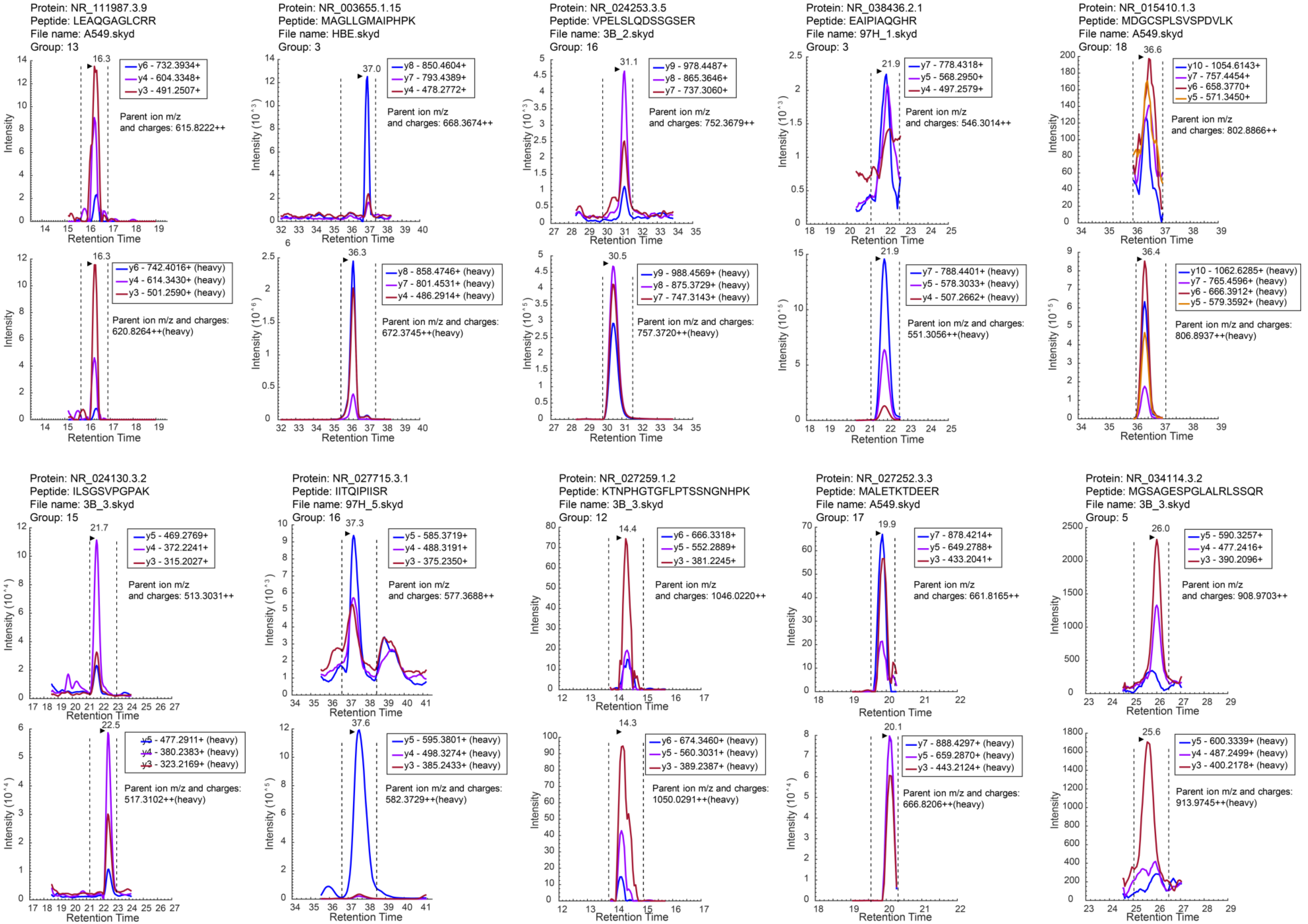

Protein: NR_111987.3.9
Peptide: LEAQGAGLCRR
File name: A549.skyd
Group: 13
y6 - 732.3934+
y4 - 604.3348+
y3 - 491.2507+
Parent ion m/z and charges: 615.8222++
y6 - 742.4016+ (heavy)
y4 - 614.3430+ (heavy)
y3 - 501.2590+ (heavy)
Parent ion m/z and charges: 620.8264++(heavy)
Protein: NR_003655.1.15
Peptide: MAGLLGMAIPHPK
File name: HBE.skyd
Group: 3
y8 - 850.4604+
y7 - 793.4389+
y4 - 478.2772+
Parent ion m/z and charges: 668.3674++
y8 - 858.4746+ (heavy)
y7 - 801.4531+ (heavy)
y4 - 486.2914+ (heavy)
Parent ion m/z and charges: 672.3745++(heavy)
Protein: NR_024253.3.5
Peptide: VPELSLQDSSGSER
File name: 3B_2.skyd
Group: 16
y9 - 978.4487+
y8 - 865.3646+
y7 - 737.3060+
Parent ion m/z and charges: 752.3679++
y9 - 988.4569+ (heavy)
y8 - 875.3729+ (heavy)
y7 - 747.3143+ (heavy)
Parent ion m/z and charges: 757.3720++(heavy)
Protein: NR_038436.2.1
Peptide: EAIPIAQGHR
File name: 97H_1.skyd
Group: 3
y7 - 778.4318+
y5 - 568.2950+
y4 - 497.2579+
Parent ion m/z and charges: 546.3014++
y7 - 788.4401+ (heavy)
y5 - 578.3033+ (heavy)
y4 - 507.2662+ (heavy)
Parent ion m/z and charges: 551.3056++(heavy)
Protein: NR_015410.1.3
Peptide: MDGCSPLSVSPDVLK
File name: A549.skyd
Group: 18
y10 - 1054.6143+
y7 - 757.4454+
y6 - 658.3770+
y5 - 571.3450+
Parent ion m/z and charges: 802.8866++
y10 - 1062.6285+ (heavy)
y7 - 765.4596+ (heavy)
y6 - 666.3912+ (heavy)
y5 - 579.3592+ (heavy)
Parent ion m/z and charges: 806.8937++(heavy)
Protein: NR_024130.3.2
Peptide: ILSGSVPGPAK
File name: 3B_3.skyd
Group: 15
y5 - 469.2769+
y4 - 372.2241+
y3 - 315.2027+
Parent ion m/z and charges: 513.3031++
y5 - 477.2911+ (heavy)
y4 - 380.2383+ (heavy)
y3 - 323.2169+ (heavy)
Parent ion m/z and charges: 517.3102++(heavy)
Protein: NR_027715.3.1
Peptide: IITQIPIISR
File name: 97H_5.skyd
Group: 16
y5 - 585.3719+
y4 - 488.3191+
y3 - 375.2350+
Parent ion m/z and charges: 577.3688++
y5 - 595.3801+ (heavy)
y4 - 498.3274+ (heavy)
y3 - 385.2433+ (heavy)
Parent ion m/z and charges: 582.3729++(heavy)
Protein: NR_027259.1.2
Peptide: KTNPHGTGFLPTSSNGNHPK
File name: 3B_3.skyd
Group: 12
y6 - 666.3318+
y5 - 552.2889+
y3 - 381.2245+
Parent ion m/z and charges: 1046.0220++
y6 - 674.3460+ (heavy)
y5 - 560.3031+ (heavy)
y3 - 389.2387+ (heavy)
Parent ion m/z and charges: 1050.0291++(heavy)
Protein: NR_027252.3.3
Peptide: MALETKTDEER
File name: A549.skyd
Group: 17
y7 - 878.4214+
y5 - 649.2788+
y3 - 433.2041+
Parent ion m/z and charges: 661.8165++
y7 - 888.4297+ (heavy)
y5 - 659.2870+ (heavy)
y3 - 443.2124+ (heavy)
Parent ion m/z and charges: 666.8206++(heavy)
Protein: NR_034114.3.2
Peptide: MGSAGESPGLALRLSSQR
File name: 3B_3.skyd
Group: 5
y5 - 590.3257+
y4 - 477.2416+
y3 - 390.2096+
Parent ion m/z and charges: 908.9703++
y5 - 600.3339+ (heavy)
y4 - 487.2499+ (heavy)
y3 - 400.2178+ (heavy)
Parent ion m/z and charges: 913.9745++(heavy)
Retention Time
Intensity

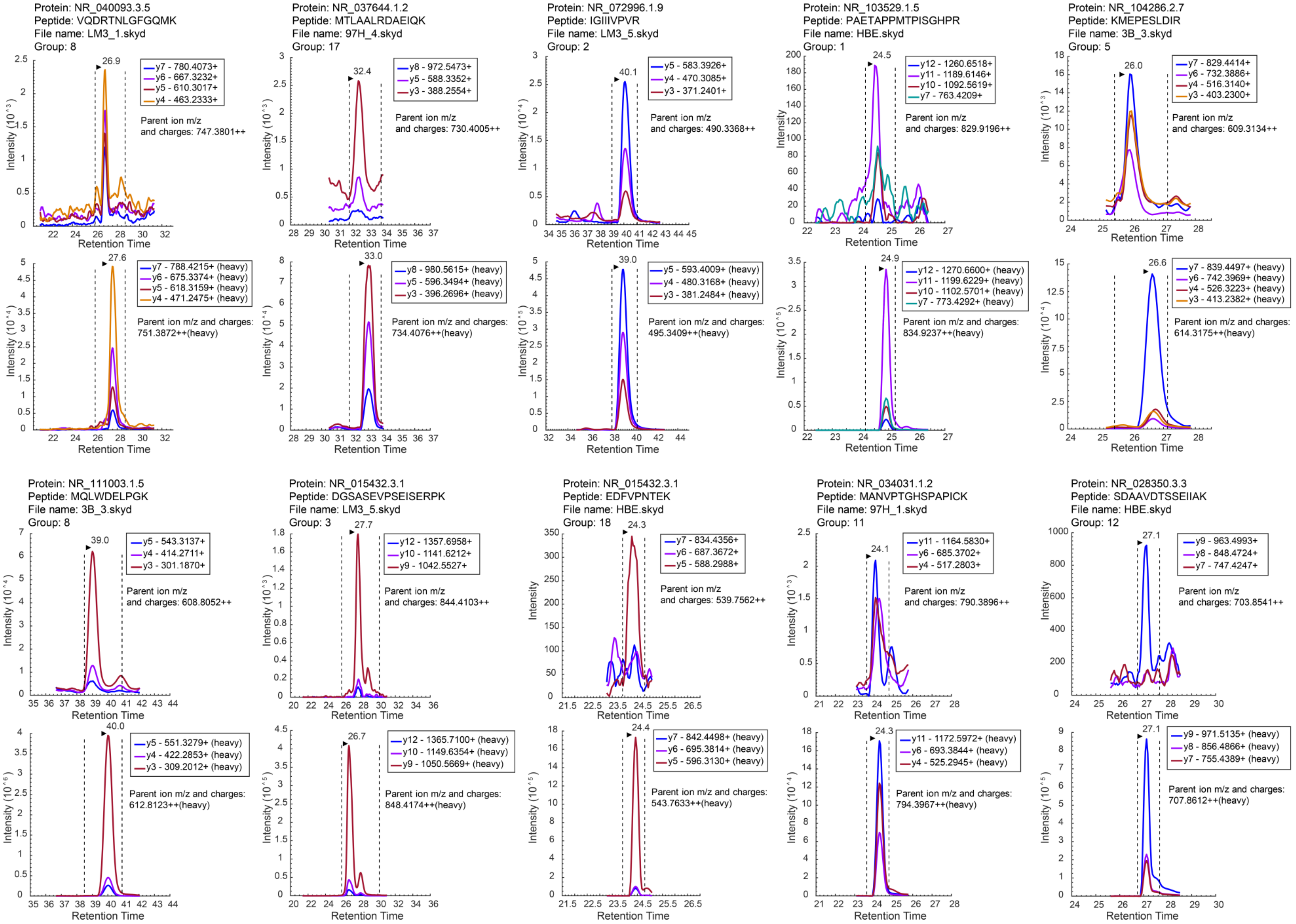
Protein: NR_040093.3.5
Peptide: VQDRTNLGFGQMK
File name: LM3_1.skyd
Group: 8
y7 - 780.4073+
y6 - 667.3232+
y5 - 610.3017+
y4 - 463.2333+
Parent ion m/z and charges: 747.3801++
y7 - 788.4215+ (heavy)
y6 - 675.3374+ (heavy)
y5 - 618.3159+ (heavy)
y4 - 471.2475+ (heavy)
Parent ion m/z and charges: 751.3872++(heavy)
Protein: NR_037644.1.2
Peptide: MTLAALRDAEIQK
File name: 97H_4.skyd
Group: 17
y8 - 972.5473+
y5 - 588.3352+
y3 - 388.2554+
Parent ion m/z and charges: 730.4005++
y8 - 980.5615+ (heavy)
y5 - 596.3494+ (heavy)
y3 - 396.2696+ (heavy)
Parent ion m/z and charges: 734.4076++(heavy)
Protein: NR_072996.1.9
Peptide: IGIIIVPVR
File name: LM3_5.skyd
Group: 2
y5 - 583.3926+
y4 - 470.3085+
y3 - 371.2401+
Parent ion m/z and charges: 490.3368++
y5 - 593.4009+ (heavy)
y4 - 480.3168+ (heavy)
y3 - 381.2484+ (heavy)
Parent ion m/z and charges: 495.3409++(heavy)
Protein: NR_103529.1.5
Peptide: PAETAPPMTPISGHPR
File name: HBE.skyd
Group: 1
y12 - 1260.6518+
y11 - 1189.6146+
y10 - 1092.5619+
y7 - 763.4209+
Parent ion m/z and charges: 829.9196++
y12 - 1270.6600+ (heavy)
y11 - 1199.6229+ (heavy)
y10 - 1102.5701+ (heavy)
y7 - 773.4292+ (heavy)
Parent ion m/z and charges: 834.9237++(heavy)
Protein: NR_104286.2.7
Peptide: KMEPESLDIR
File name: 3B_3.skyd
Group: 5
y7 - 829.4414+
y6 - 732.3886+
y4 - 516.3140+
y3 - 403.2300+
Parent ion m/z and charges: 609.3134++
y7 - 839.4497+ (heavy)
y6 - 742.3969+ (heavy)
y4 - 526.3223+ (heavy)
y3 - 413.2382+ (heavy)
Parent ion m/z and charges: 614.3175++(heavy)
Protein: NR_111003.1.5
Peptide: MQLWDELPGK
File name: 3B_3.skyd
Group: 8
y5 - 543.3137+
y4 - 414.2711+
y3 - 301.1870+
Parent ion m/z and charges: 608.8052++
y5 - 551.3279+ (heavy)
y4 - 422.2853+ (heavy)
y3 - 309.2012+ (heavy)
Parent ion m/z and charges: 612.8123++(heavy)
Protein: NR_015432.3.1
Peptide: DGSASEVPSEISERPK
File name: LM3_5.skyd
Group: 3
y12 - 1357.6958+
y10 - 1141.6212+
y9 - 1042.5527+
Parent ion m/z and charges: 844.4103++
y12 - 1365.7100+ (heavy)
y10 - 1149.6354+ (heavy)
y9 - 1050.5669+ (heavy)
Parent ion m/z and charges: 848.4174++(heavy)
Protein: NR_015432.3.1
Peptide: EDFVPNTEK
File name: HBE.skyd
Group: 18
y7 - 834.4356+
y6 - 687.3672+
y5 - 588.2988+
Parent ion m/z and charges: 539.7562++
y7 - 842.4498+ (heavy)
y6 - 695.3814+ (heavy)
y5 - 596.3130+ (heavy)
Parent ion m/z and charges: 543.7633++(heavy)
Protein: NR_034031.1.2
Peptide: MANVPTGHSPAPICK
File name: 97H_1.skyd
Group: 11
y11 - 1164.5830+
y6 - 685.3702+
y4 - 517.2803+
Parent ion m/z and charges: 790.3896++
y11 - 1172.5972+ (heavy)
y6 - 693.3844+ (heavy)
y4 - 525.2945+ (heavy)
Parent ion m/z and charges: 794.3967++(heavy)
Protein: NR_028350.3.3
Peptide: SDAAVDTSSEIIAK
File name: HBE.skyd
Group: 12
y9 - 963.4993+
y8 - 848.4724+
y7 - 747.4247+
Parent ion m/z and charges: 703.8541++
y9 - 971.5135+ (heavy)
y8 - 856.4866+ (heavy)
y7 - 755.4389+ (heavy)
Parent ion m/z and charges: 707.8612++(heavy)
Retention Time
Intensity
