# Supplementary material for: A hidden human proteome encoded by ‘non-coding’ genes
Source: Nucleic Acids Res. 2019 Jul 24;47(15):8111–25. doi: 10.1093/nar/gkz646 (PMC6735797; doi:10.1093/nar/gkz646)

Supplementary Fig. S5 Raw images of the western blotting analysis.

(All raw imagaes for Fig. 2E, 2F, 6C and 6F are provided)

Raw images for Fig.2E

NR_002305.1.5 (Positive and negative controls)

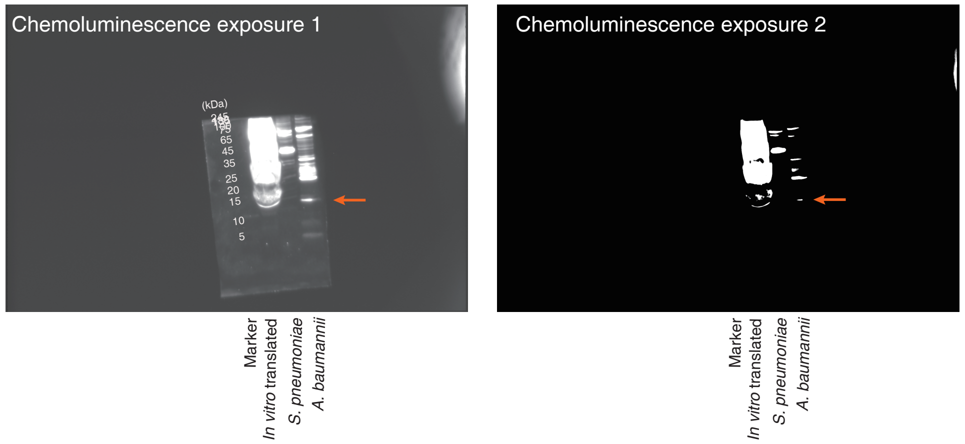

NR_002305.1.5

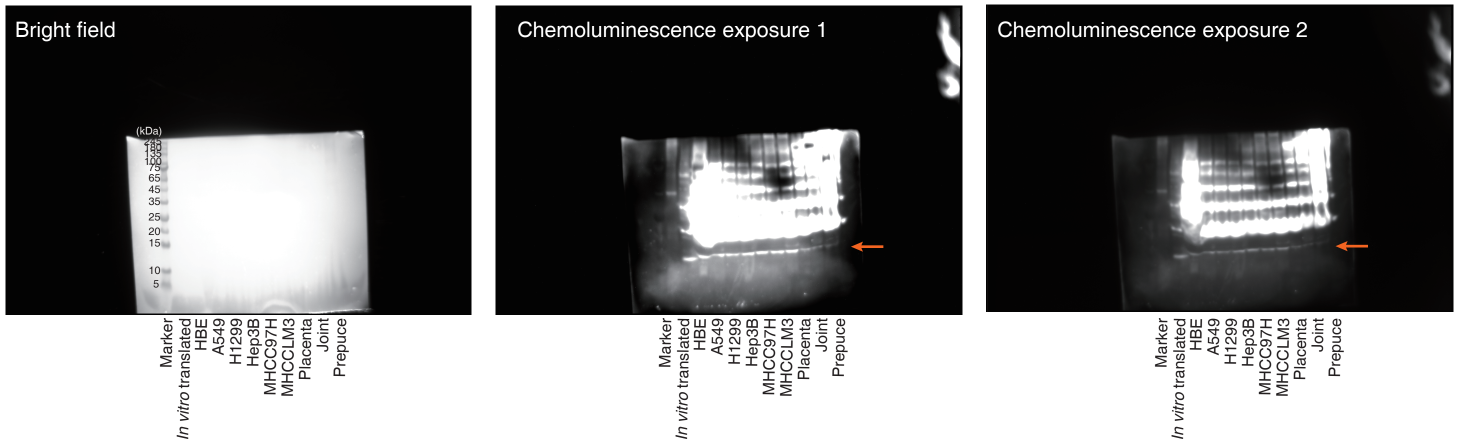

NR_015432.3.1 (Positive and negative controls)

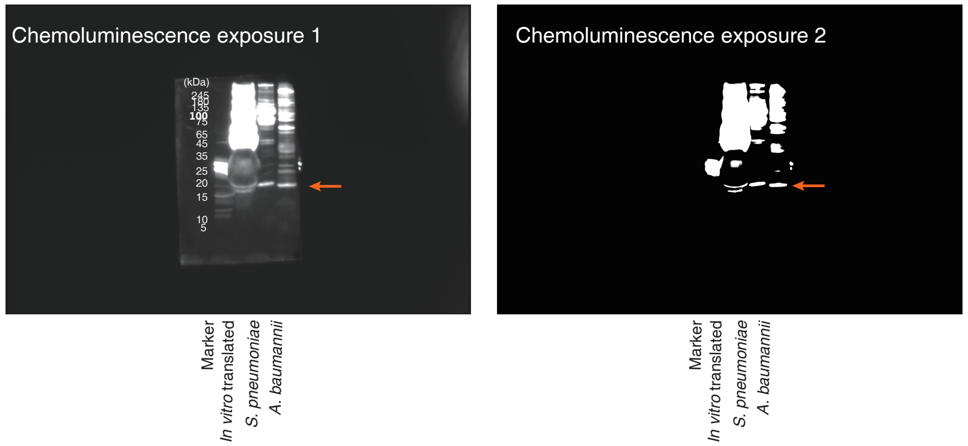

NR_015432.3.1

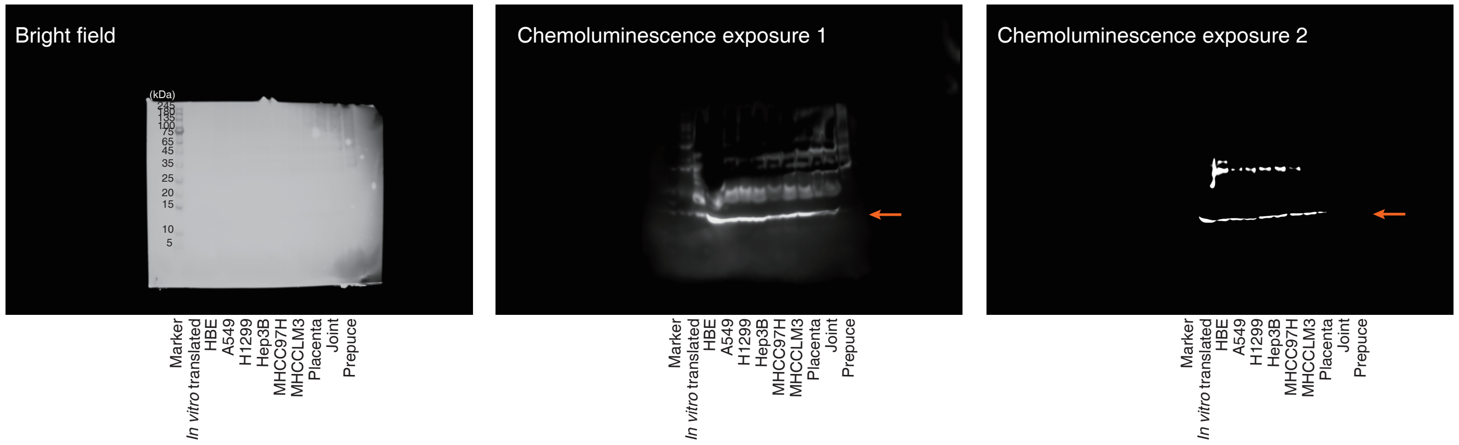

NR_072977.1.1 (Positive and negative controls)

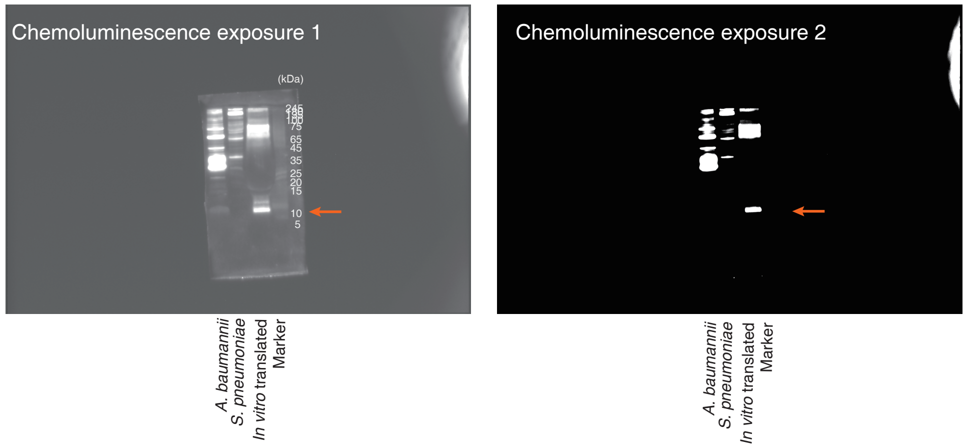

NR_072977.1.1

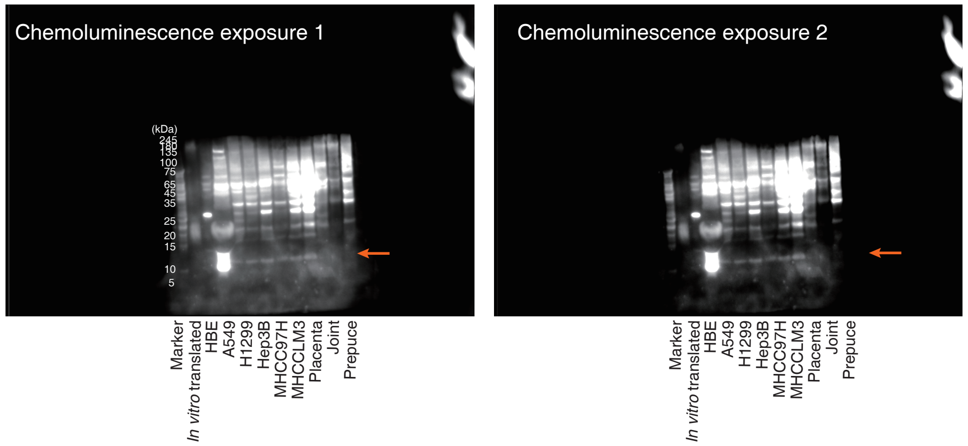

NR_104286.2.7 (Positive and negative controls)

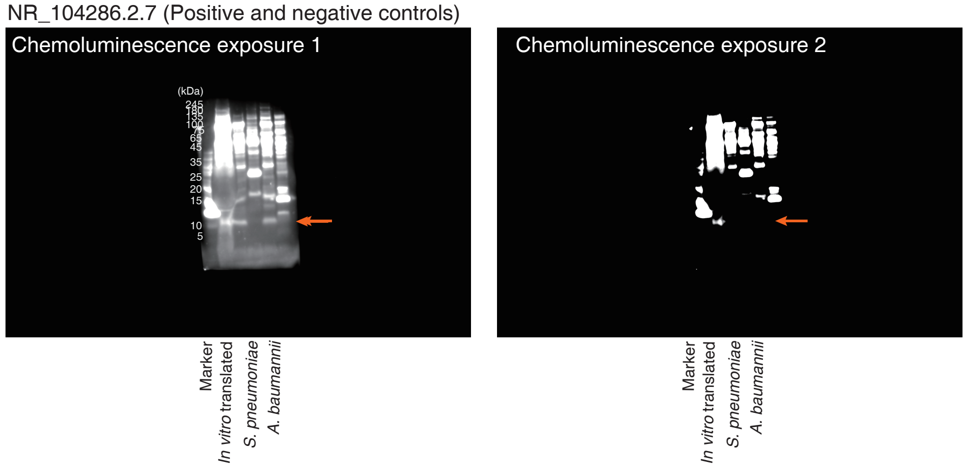

NR_104286.2.7

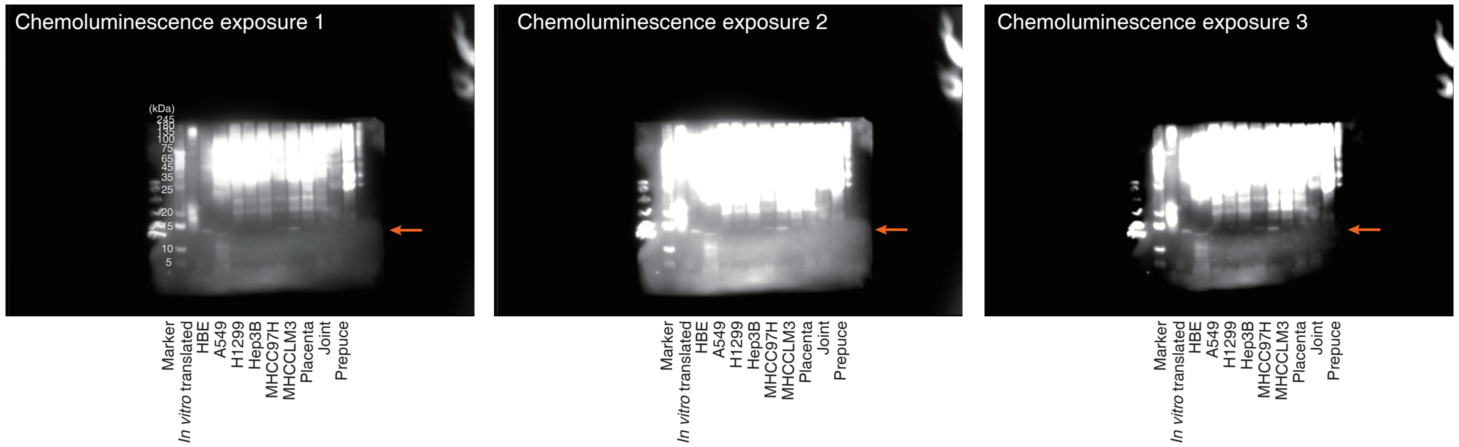

Raw images for Fig.2F

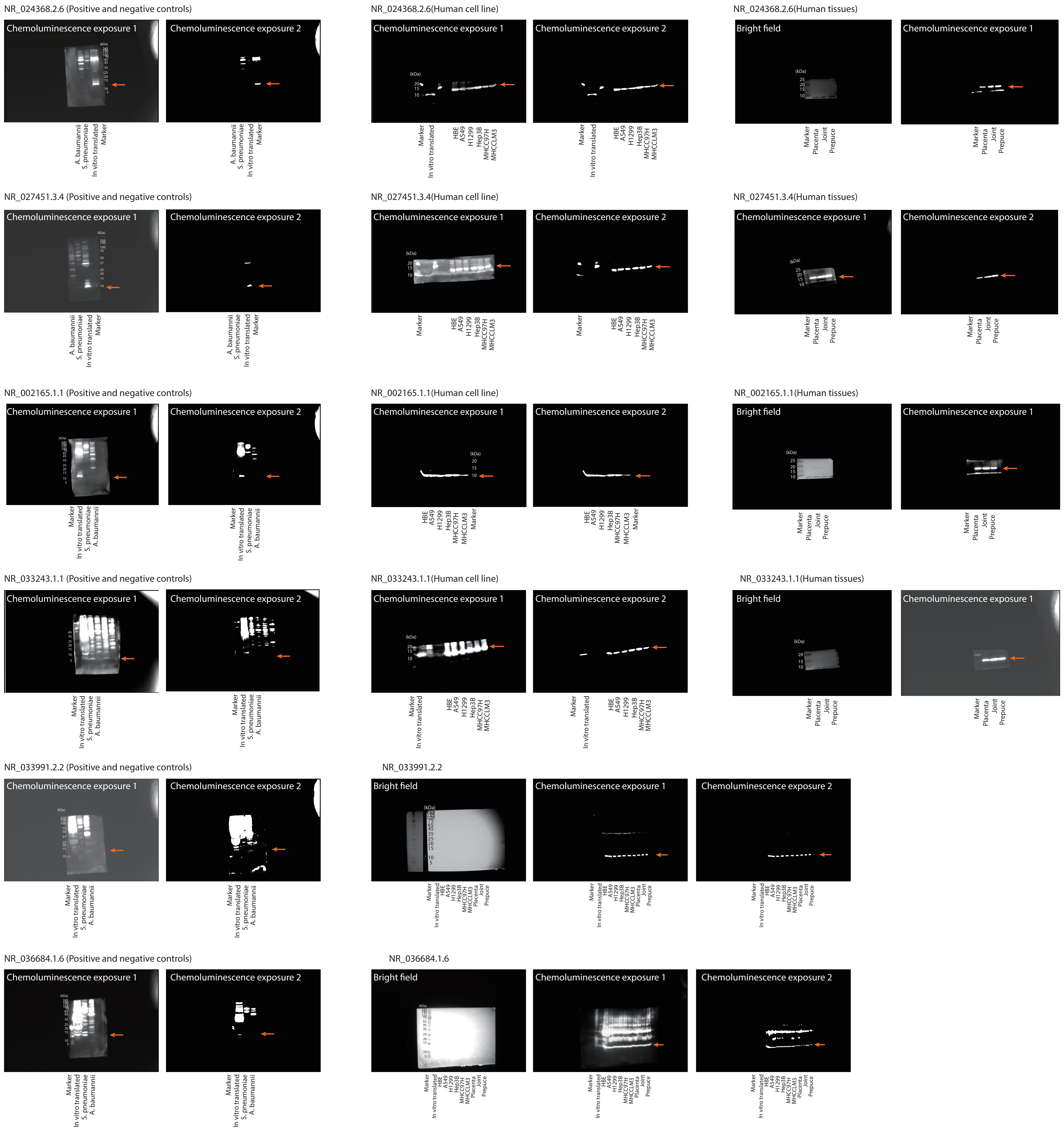

Raw images for Fig.6C

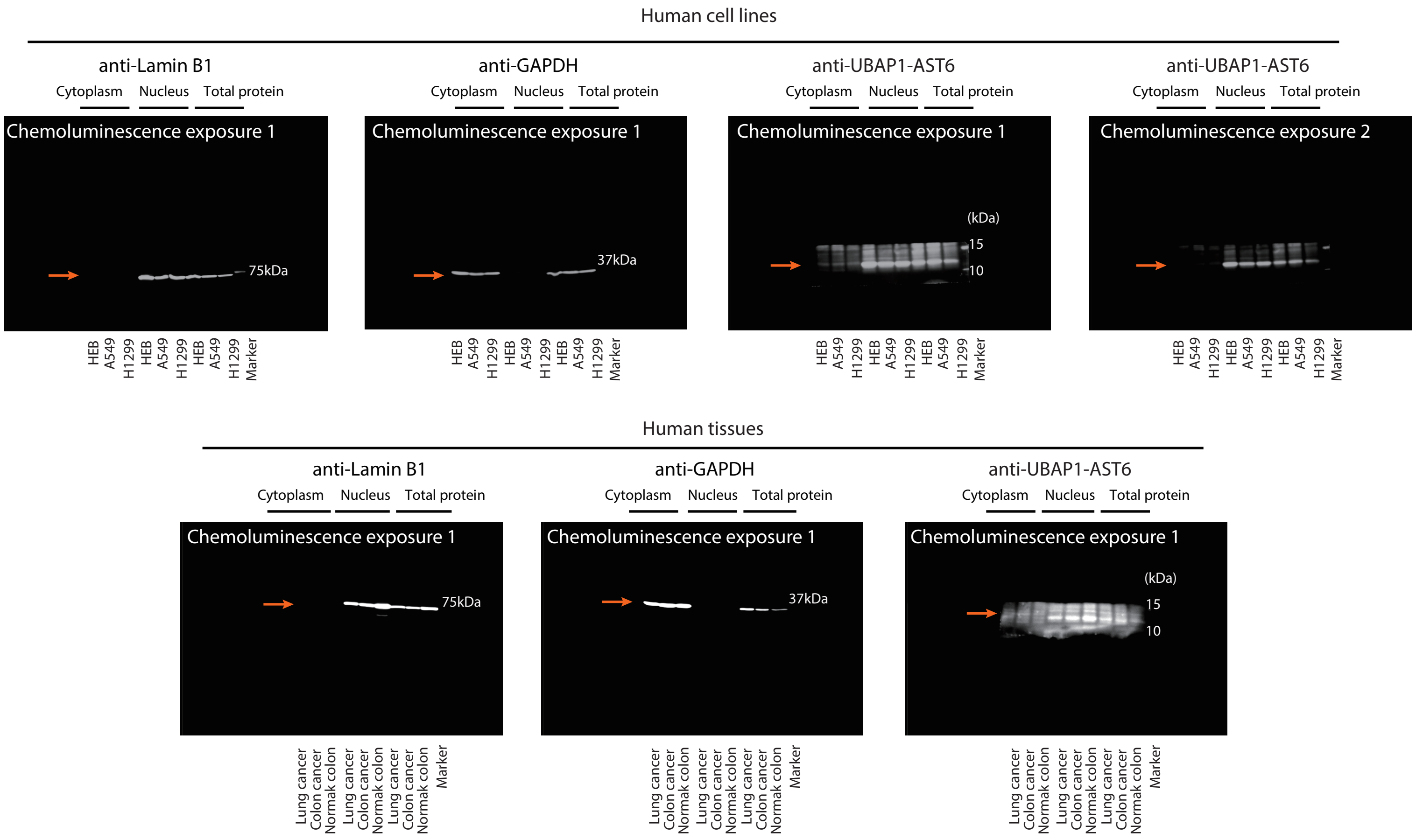

Raw images for Fig.6F

GAPDH

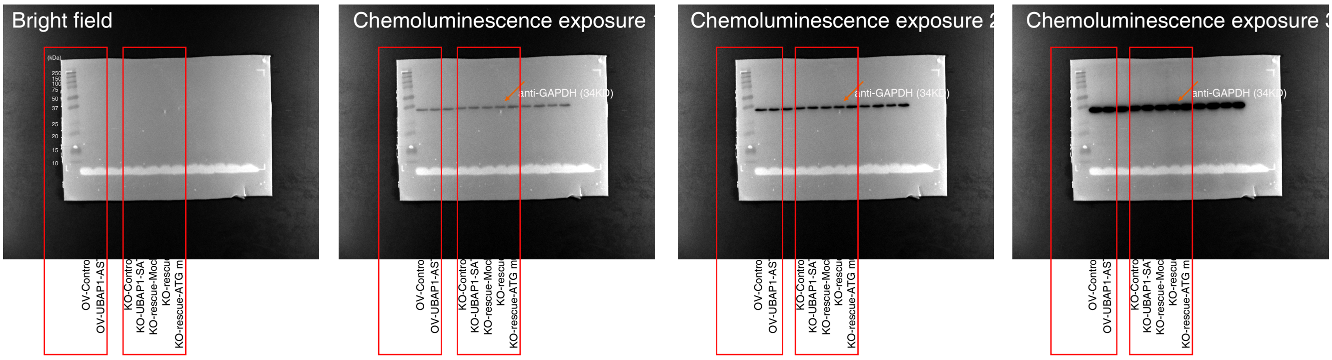

UBAP1-AST6

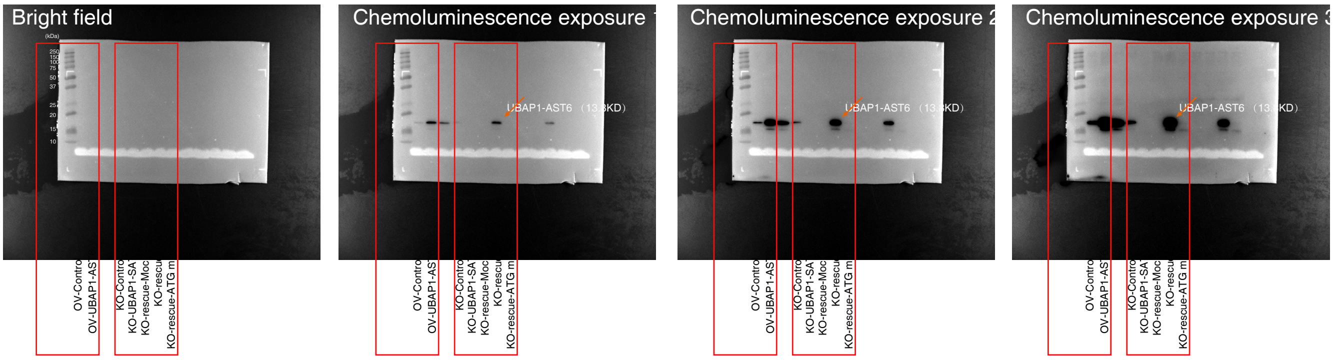

Supplement: gkz646_Supplemental_Files [file gkz646_supplemental_files.zip › Supplementary Fig S5_Raw images of the western blotting analysis.pdf]
