# Supplementary material for: A hidden human proteome encoded by ‘non-coding’ genes
Source: Nucleic Acids Res. 2019 Jul 24;47(15):8111–25. doi: 10.1093/nar/gkz646 (PMC6735797; doi:10.1093/nar/gkz646)
Supplement: gkz646_Supplemental_Files [file gkz646_supplemental_files.zip › Supplementary Information-review.pdf]

A Hidden Human Proteome Encoded by the "Non-Coding" Genes

Shaohua Lu†, Jing Zhang†, Xinlei Lian†, Li Sun, Kun Meng, Yang Chen, Zhenghua Sun, Xingfeng Yin, Yaxing Li, Jing Zhao, Tong Wang*, Gong Zhang* and Qing-Yu He*

Supplementary materials

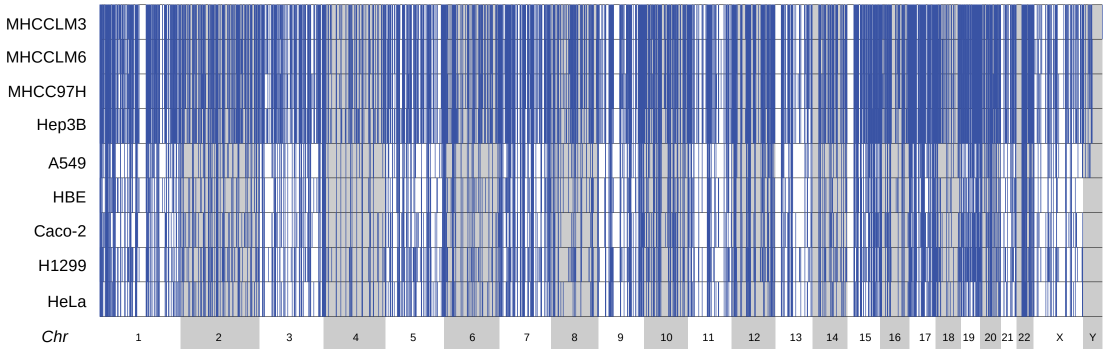

Figure S1: Chromosomal distribution of translating lncRNAs. Blue stripes represent translating lncRNAs.

Figure S2: Peptide spectra information for the MRM MS.
Separate pdf file.

Figure S3: Peptide spectra information for the PRM MS.
Separate pdf file.

Figure S4: Peptide spectra information for the Heavy-MRM MS.
Separate pdf file.

Figure S5: Raw images of the western blotting analysis.
Separate pdf file.

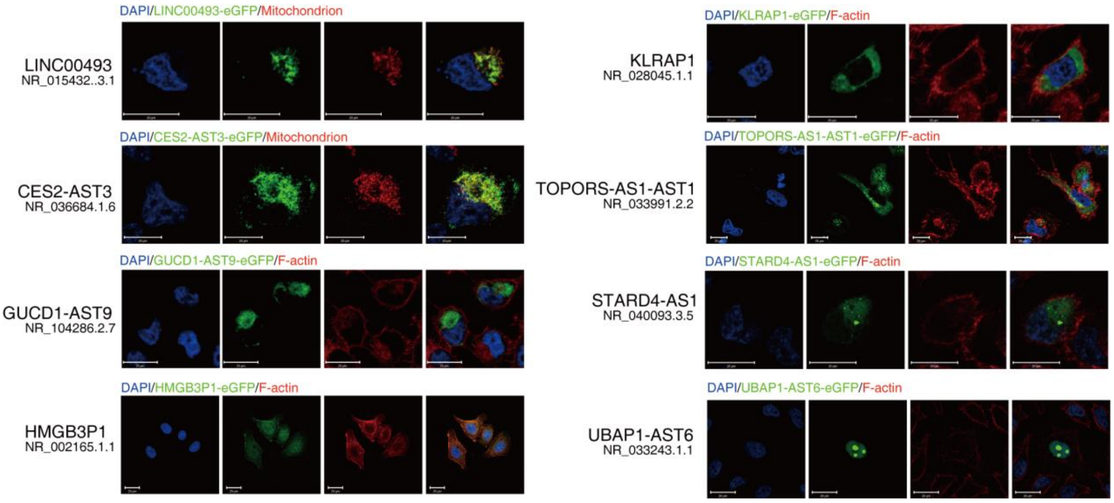

Figure S6: Confocal fluorescent microscopy observation of the subcellular localization of 8 new proteins

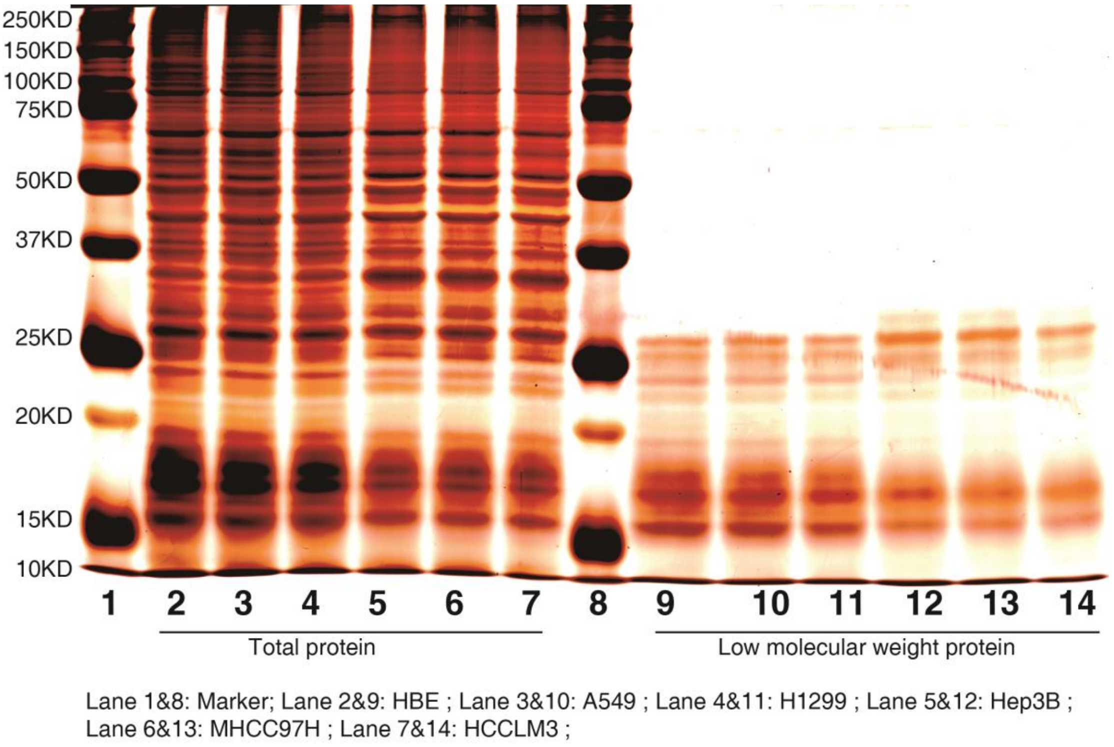

Figure S7: The silver staining gel slides showing the total proteins and protein bands between 5-25 kDa.

Supplementary Table S1. Information of translating lncRNAs.
Separate excel file.

Supplementary Table S2. Overview of new protein identifications by shotgun MS and/or

antibody verification.
Separate excel file.

Supplementary Table S3-S5 File name keys can be found at the following pages of this file.

Supplementary Table S6. Detailed results from MaxQuant, Mascot+Scaffold, and X!Tandam searches for the new proteins.
Separate excel file.

Supplementary Table S7. Detailed results of the MRM analysis on unique peptides of the new proteins.
Separate excel file.

Supplementary Table S8. Sequences of probes used for RFP isolations.

The table can be found at the following pages of this file.

Supplementary Table S9. Species and sources of genomic reference sequences.
The table can be found at the following pages of this file.

Supplementary Table S10. Detailed results of the PRM analysis on unique peptides of the new proteins.

Separate excel file.

Supplementary Table S11. Detailed results of the Heavy-MRM analysis on unique peptides of the new proteins.
Separate excel file.

Supplementary Table S12: Number of entries of the cell-specific reference databases and their file sizes. LMW = low molecular weight.
The table can be found at the following pages of this file.

Supplementary Table S13. Detailed sequences of all MS-detected new proteins.
Separate excel file.

Supplementary Table S3. Detailed information on files used in or output from the MaxQuant search.

(All file are available from iProX database: accession number: IPX00076300)

iProX	Proteome Xchange	Raw file name	Source	Instrument	Reference database file name	Search result file name
IPX00076300	PXD005299	20150709_A549_*	A549 cells; low molecular weight proteins	Triple TOF 5600	A549_LWP.fa	A549_LWP_5600_Run1_peptide.txt
						A549_LWP_5600_Run1_protein.txt
						A549_LWP_5600_Run1_psm.txt
		20150730_LWP_A549_*	A549 cells; low molecular weight proteins	Triple TOF 5600	A549_LWP.fa	A549_LWP_5600_Run2_peptide.txt
						A549_LWP_5600_Run2_protein.txt
						A549_LWP_5600_Run2_psm.txt
		20150709_H1299_*	H1299 cells; low molecular weight proteins	Triple TOF 5600	H1299_LWP.fa	H1299_LWP_5600_Run1_peptide.txt
						H1299_LWP_5600_Run1_protein.txt
						H1299_LWP_5600_Run1_psm.txt
		20150730_LWP_H1299_*	H1299 cells; low molecular weight proteins	Triple TOF 5600	H1299_LWP.fa	H1299_LWP_5600_Run2_peptide.txt
						H1299_LWP_5600_Run2_protein.txt
						H1299_LWP_5600_Run2_psm.txt
		20150709_HBE_*	HBE cells; low molecular weight proteins	Triple TOF 5600	HBE_LWP.fa	HBE_LWP_5600_Run1_peptide.txt
						HBE_LWP_5600_Run1_protein.txt
						HBE_LWP_5600_Run1_psm.txt
		20150730_LWP_HBE_*	HBE cells;	Triple TOF 5600	HBE_LWP.fa	HBE_LWP_5600_Run2_peptide.txt

			low molecular weight proteins			HBE_LWP_5600_Run2_protein.txt
						HBE_LWP_5600_Run2_psm.txt
		20150709_Hep3B_*	Hep3B cells; low molecular weight proteins	Triple TOF 5600	Hep3B_LWP.fa	Hep3B_LWP_5600_Run1_peptide.txt
						Hep3B_LWP_5600_Run1_protein.txt
						Hep3B_LWP_5600_Run1_psm.txt
		20150730_LWP_Hep3B_*	Hep3B cells; low molecular weight proteins	Triple TOF 5600	Hep3B_LWP.fa	Hep3B_LWP_5600_Run2_peptide.txt
						Hep3B_LWP_5600_Run2_protein.txt
						Hep3B_LWP_5600_Run2_psm.txt
		20150709_MHCC97H_*	MHCC97H cells; low molecular weight proteins	Triple TOF 5600	MHCC97H_LWP.fa	MHCC97H_LWP_5600_Run1_peptide.txt
						MHCC97H_LWP_5600_Run1_protein.txt
						MHCC97H_LWP_5600_Run1_psm.txt
		20150730_LWP_MHCC97H_*	MHCC97H cells; low molecular weight proteins	Triple TOF 5600	MHCC97H_LWP.fa	MHCC97H_LWP_5600_Run2_peptide.txt
						MHCC97H_LWP_5600_Run2_protein.txt
						MHCC97H_LWP_5600_Run2_psm.txt
		20150709_MHCCLM3_*	MHCCLM3 cells; low molecular weight proteins	Triple TOF 5600	MHCCLM3_LWP.fa	MHCCLM3_LWP_5600_Run1_peptide.txt
						MHCCLM3_LWP_5600_Run1_protein.txt
						MHCCLM3_LWP_5600_Run1_psm.txt
		20150730_LWP_MHCCLM3_*	MHCCLM3 cells; low molecular weight	Triple TOF 5600	MHCCLM3_LWP.fa	MHCCLM3_LWP_5600_Run2_peptide.txt
						MHCCLM3_LWP_5600_Run2_protein.txt

			proteins			MHCCLM3_LWP_5600_Run2_psm.txt
IPX00076201	PXD005299	CYZ_20120726_*.	SILAC labeled A549 and HBE cells; whole cell lysate	LTQ-Orbitrap	A549 & HBE.fa	A549_HBE_silac_LTQ_peptide.txt
						A549_HBE_silac_LTQ_protein.txt
						A549_HBE_silac_LTQ_psm.txt
IPX00076202	PXD005299	CYZ_20130101_*	SILAC labeled H1299 and HBE cells; whole cell lysate	LTQ-Orbitrap	H1299 & HBE.fa	H1299_HBE_silac_LTQ_peptide.txt
						H1299_HBE_silac_LTQ_protein.txt
						H1299_HBE_silac_LTQ_psm.txt
IPX0000020006	PXD000535	Hep3B-*-R1	Hep3B cells; whole cell lysate	Triple TOF 5600	Hep3B.fa	Hep3B_5600_Run1_peptide.txt
						Hep3B_5600_Run1_protein.txt
						Hep3B_5600_Run1_psm.txt
		Hep3B-*-R2	Hep3B cells; whole cell lysate	Triple TOF 5600	Hep3B.fa	Hep3B_5600_Run2_peptide.txt
						Hep3B_5600_Run2_protein.txt
						Hep3B_5600_Run2_psm.txt
IPX0000020004	PXD000533	3B*-1	Hep3B cells; whole cell lysate	Orbitrap Q Exactive	Hep3B.fa	Hep3B_QE_Run1_peptide.txt
						Hep3B_QE_Run1_protein.txt
						Hep3B_QE_Run1_psm.txt
		3B*-2	Hep3B cells; whole cell lysate	Orbitrap Q Exactive	Hep3B.fa	Hep3B_QE_Run2_peptide.txt
						Hep3B_QE_Run2_protein.txt
						Hep3B_QE_Run2_psm.txt

IPX0000020005	PXD000535	97H-*-R1	MHCC97H cells; whole cell lysate	Triple TOF 5600	MHCC97H.fa	MHCC97H_5600_Run1_peptide.txt
						MHCC97H_5600_Run1_protein.txt
						MHCC97H_5600_Run1_psm.txt
		97H-*-R2	MHCC97H cells; whole cell lysate	Triple TOF 5600	MHCC97H.fa	MHCC97H_5600_Run2_peptide.txt
						MHCC97H_5600_Run2_protein.txt
						MHCC97H_5600_Run2_psm.txt
IPX0000020001	PXD000529	CHPP_97H_RP*_1	MHCC97H cells; whole cell lysate	Orbitrap Q Exactive	MHCC97H.fa	MHCC97H_QE_Run1_peptide.txt
						MHCC97H_QE_Run1_protein.txt
						MHCC97H_QE_Run1_psm.txt
		CHPP_97H_RP*_2	MHCC97H cells; whole cell lysate	Orbitrap Q Exactive	MHCC97H.fa	MHCC97H_QE_Run2_peptide.txt
						MHCC97H_QE_Run2_protein.txt
						MHCC97H_QE_Run2_psm.txt
IPX0000020003	PXD000533	H*-1	MHCC97H cells; whole cell lysate	Orbitrap Q Exactive	MHCC97H.fa	MHCC97H_QE_Yang_Run1_peptide.txt
						MHCC97H_QE_Yang_Run1_protein.txt
						MHCC97H_QE_Yang_Run1_psm.txt
		H*-2	MHCC97H cells; whole cell lysate	Orbitrap Q Exactive	MHCC97H.fa	MHCC97H_QE_Yang_Run2_peptide.txt
						MHCC97H_QE_Yang_Run2_protein.txt
						MHCC97H_QE_Yang_Run2_psm.txt
IPX0000020002	PXD000529	CHPP_LM3_RP*_1	MHCCLM3 cells;	Orbitrap Q Exactive	MHCCLM3.fa	MHCCLM3_QE_Run1_peptide.txt

			whole cell lysate			MHCCLM3_QE_Run1_protein.txt
						MHCCLM3_QE_Run1_psm.txt
		CHPP_LM3_RP*_2	MHCCLM3 cells;<br>whole cell lysate	Orbitrap Q Exactive	MHCCLM3.fa	MHCCLM3_QE_Run2_peptide.txt
						MHCCLM3_QE_Run2_protein.txt
						MHCCLM3_QE_Run2_psm.txt
	PXD001305	20140529_QE7_<br>UPLC9_RJC_DEV_<br>Exp3_faster_1ug_01	Hela cells;<br>whole cell lysate	Orbitrap Q Exactive	HeLa.fa	Hela_QE_peptide.txt
						Hela_QE_protein.txt
						Hela_QE_psm.txt

Note:

* Represents the fraction number.

Supplementary Table S4. Detailed information on files used in or output from the Mascot+Scaffold searches.

(All file are available from iProX database: accession number: IPX00076300)

Mascot+ scaffold searches						
**iProX**	**Proteome-Xchange**	**Raw file name**	**Source**	**Instrument**	**Reference database file name**	**Search result file name**
IPX00076300	PXD005299	20150709_A549_*<br>20150730_LWP_A549_*	A549 cells;<br>low molecular weight proteins	Triple TOF 5600	A549_LWP.fa	A549_LWP_5600_peptide.xls
						A549_LWP_5600_protein.xls
						A549_LWP_5600_psm.xls
		20150709_H1299_*<br>20150730_LWP_H1299_*	H1299 cells;<br>low molecular weight proteins	Triple TOF 5600	H1299_LWP.fa	H1299_LWP_5600_peptide.xls
						H1299_LWP_5600_protein.xls
						H1299_LWP_5600_psm.xls
		20150709_HBE_*<br>20150730_LWP_HBE_*	HBE cells;<br>low molecular weight proteins	Triple TOF 5600	HBE_LWP.fa	HBE_LWP_5600_peptide.xls
						HBE_LWP_5600_protein.xls
						HBE_LWP_5600_psm.xls
		20150709_Hep3B_*<br>20150730_LWP_Hep3B_*	Hep3B cells;<br>low molecular weight proteins	Triple TOF 5600	Hep3B_LWP.fa	Hep3B_LWP_5600_peptide.xls
						Hep3B_LWP_5600_protein.xls
						Hep3B_LWP_5600_psm.xls
		20150709_MHCC97H_*	MHCC97H cells;	Triple TOF 5600	MHCC97H_LWP.fa	MHCC97H_LWP_5600_peptide.xls

		20150730_LWP_MHCC97H_*	low molecular weight proteins			MHCC97H_LWP_5600_protein.xls<br>MHCC97H_LWP_5600_psm.xls
		20150709_MHCCLM3_*<br>20150730_LWP_MHCCLM3_*	MHCCLM3 cells;<br>low molecular weight proteins	Triple TOF 5600	MHCCLM3_LWP.fa	MHCCLM3_LWP_5600_peptide.xls<br>MHCCLM3_LWP_5600_protein.xls<br>MHCCLM3_LWP_5600_psm.xls
IPX0000020006	PXD000535	Hep3B-*-R1<br>Hep3B-*-R2	Hep3B cells;<br>whole cell lysate	Triple TOF 5600	Hep3B.fa	Hep3B_5600_peptide.xls<br>Hep3B_5600_protein.xls<br>Hep3B_5600_psm.xls
IPX0000020004	PXD000533	3B*-1<br>3B*-2	Hep3B cells;<br>whole cell lysate	Orbitrap Q Exactive	Hep3B.fa	Hep3B_QE_peptide.xls<br>Hep3B_QE_protein.xls<br>Hep3B_QE_psm.xls
IPX0000020005	PXD000535	97H-*-R1<br>97H-*-R2	MHCC97H cells;<br>whole cell lysate	Triple TOF 5600	MHCC97H.fa	MHCC97H_5600_peptide.xls<br>MHCC97H_5600_protein.xls<br>MHCC97H_5600_psm.xls
IPX0000020001	PXD000529	CHPP_97H_RP*_1<br>CHPP_97H_RP*_2	MHCC97H cells;<br>whole cell lysate	Orbitrap Q Exactive	MHCC97H.fa	MHCC97H_QE_peptide.xls<br>MHCC97H_QE_protein.xls<br>MHCC97H_QE_psm.xls
IPX0000020003	PXD000533	H*-1<br>H*-2	MHCC97H cells;<br>whole cell lysate	Orbitrap Q Exactive	MHCC97H.fa	MHCC97H_QE_Yang_peptide.xls<br>MHCC97H_QE_Yang_protein.xls

						MHCC97H_QE_Yang_psm.xls
IPX0000020002	PXD000529	CHPP_LM3_RP*_1<br>CHPP_LM3_RP*_2	MHCCLM3 cells;<br>whole cell lysate	Orbitrap Q Exactive	MHCCLM3.fa	MHCCLM3_QE_peptide.xls
						MHCCLM3_QE_protein.xls
						MHCCLM3_QE_psm.xls

Note:

* Represents the fraction number.

Supplementary Table S5. Detailed information on files used in or output from the X!Tandem searches.
(All file are available from iProX database: accession number: IPX00076300)

iProX	Proteome Xchange	Raw file name	Source	Instrument	Reference database file name	Search result file name
IPX00076300	PXD005299	20150709_A549_*	A549 cells;<br>low molecular weight proteins	Triple TOF 5600	A549_LWP.fa	A549_LWP_5600_Run1_peptide.txt
						A549_LWP_5600_Run1_protein.txt
						A549_LWP_5600_Run1_psm.txt
		20150730_LWP_A549_*	A549 cells;<br>low molecular weight proteins	Triple TOF 5600	A549_LWP.fa	A549_LWP_5600_Run2_peptide.txt
						A549_LWP_5600_Run2_protein.txt
						A549_LWP_5600_Run2_psm.txt
		20150709_H1299_*	H1299 cells;	Triple TOF 5600	H1299_LWP.fa	H1299_LWP_5600_Run1_peptide.txt

			low molecular weight proteins			H1299_LWP_5600_Run1_protein.txt
						H1299_LWP_5600_Run1_psm.txt
		20150730_LWP_H1299_*	H1299 cells; low molecular weight proteins	Triple TOF 5600	H1299_LWP.fa	H1299_LWP_5600_Run2_peptide.txt
						H1299_LWP_5600_Run2_protein.txt
						H1299_LWP_5600_Run2_psm.txt
		20150709_HBE_*	HBE cells; low molecular weight proteins	Triple TOF 5600	HBE_LWP.fa	HBE_LWP_5600_Run1_peptide.txt
						HBE_LWP_5600_Run1_protein.txt
						HBE_LWP_5600_Run1_psm.txt
		20150730_LWP_HBE_*	HBE cells; low molecular weight proteins	Triple TOF 5600	HBE_LWP.fa	HBE_LWP_5600_Run2_peptide.txt
						HBE_LWP_5600_Run2_protein.txt
						HBE_LWP_5600_Run2_psm.txt
		20150709_Hep3B_*	Hep3B cells; low molecular weight proteins	Triple TOF 5600	Hep3B_LWP.fa	Hep3B_LWP_5600_Run1_peptide.txt
						Hep3B_LWP_5600_Run1_protein.txt
						Hep3B_LWP_5600_Run1_psm.txt
		20150730_LWP_Hep3B_*	Hep3B cells; low molecular weight proteins	Triple TOF 5600	Hep3B_LWP.fa	Hep3B_LWP_5600_Run2_peptide.txt
						Hep3B_LWP_5600_Run2_protein.txt
						Hep3B_LWP_5600_Run2_psm.txt
		20150709_MHCC97H_*	MHCC97H cells; low molecular weight	Triple TOF 5600	MHCC97H_LWP.fa	MHCC97H_LWP_5600_Run1_peptide.txt
						MHCC97H_LWP_5600_Run1_protein.txt

			proteins			MHCC97H_LWP_5600_Run1_psm.txt
		20150730_LWP_MHCC97H_*	MHCC97H cells; low molecular weight proteins	Triple TOF 5600	MHCC97H_LWP.fa	MHCC97H_LWP_5600_Run2_peptide.txt
						MHCC97H_LWP_5600_Run2_protein.txt
						MHCC97H_LWP_5600_Run2_psm.txt
		20150709_MHCCLM3_*	MHCCLM3 cells; low molecular weight proteins	Triple TOF 5600	MHCCLM3_LWP.fa	MHCCLM3_LWP_5600_Run1_peptide.txt
						MHCCLM3_LWP_5600_Run1_protein.txt
						MHCCLM3_LWP_5600_Run1_psm.txt
		20150730_LWP_MHCCLM3_*	MHCCLM3 cells; low molecular weight proteins	Triple TOF 5600	MHCCLM3_LWP.fa	MHCCLM3_LWP_5600_Run2_peptide.txt
						MHCCLM3_LWP_5600_Run2_protein.txt
						MHCCLM3_LWP_5600_Run2_psm.txt
IPX0000020006	PXD000535	Hep3B-*-R1	Hep3B cells; whole cell lysate	Triple TOF 5600	Hep3B.fa	Hep3B_5600_Run1_peptide.txt
						Hep3B_5600_Run1_protein.txt
						Hep3B_5600_Run1_psm.txt
		Hep3B-*-R2	Hep3B cells; whole cell lysate	Triple TOF 5600	Hep3B.fa	Hep3B_5600_Run2_peptide.txt
						Hep3B_5600_Run2_protein.txt
						Hep3B_5600_Run2_psm.txt
IPX0000020004	PXD000533	3B*-1	Hep3B cells; whole cell lysate	Orbitrap Q Exactive	Hep3B.fa	Hep3B_QE_Run1_peptide.txt
						Hep3B_QE_Run1_protein.txt
						Hep3B_QE_Run1_psm.txt

		3B*-2	Hep3B cells; whole cell lysate	Orbitrap Q Exactive	Hep3B.fa	Hep3B_QE_Run2_peptide.txt
						Hep3B_QE_Run2_protein.txt
						Hep3B_QE_Run2_psm.txt
IPX0000020005	PXD000535	97H-*-R1	MHCC97H cells; whole cell lysate	Triple TOF 5600	MHCC97H.fa	MHCC97H_5600_Run1_peptide.txt
						MHCC97H_5600_Run1_protein.txt
						MHCC97H_5600_Run1_psm.txt
		97H-*-R2	MHCC97H cells; whole cell lysate	Triple TOF 5600	MHCC97H.fa	MHCC97H_5600_Run2_peptide.txt
						MHCC97H_5600_Run2_protein.txt
						MHCC97H_5600_Run2_psm.txt
IPX0000020001	PXD000529	CHPP_97H_RP*_1	MHCC97H cells; whole cell lysate	Orbitrap Q Exactive	MHCC97H.fa	MHCC97H_QE_Run1_peptide.txt
						MHCC97H_QE_Run1_protein.txt
						MHCC97H_QE_Run1_psm.txt
		CHPP_97H_RP*_2	MHCC97H cells; whole cell lysate	Orbitrap Q Exactive	MHCC97H.fa	MHCC97H_QE_Run2_peptide.txt
						MHCC97H_QE_Run2_protein.txt
						MHCC97H_QE_Run2_psm.txt
IPX0000020003	PXD000533	H*-1	MHCC97H cells; whole cell lysate	Orbitrap Q Exactive	MHCC97H.fa	MHCC97H_QE_Yang_Run1_peptide.txt
						MHCC97H_QE_Yang_Run1_protein.txt
						MHCC97H_QE_Yang_Run1_psm.txt
		H*-2	MHCC97H cells;	Orbitrap Q Exactive	MHCC97H.fa	MHCC97H_QE_Yang_Run2_peptide.txt

			whole cell lysate			MHCC97H_QE_Yang_Run2_protein.txt
						MHCC97H_QE_Yang_Run2_psm.txt
IPX0000020002	PXD000529	CHPP_LM3_RP*_1	MHCCLM3 cells; whole cell lysate	Orbitrap Q Exactive	MHCCLM3.fa	MHCCLM3_QE_Run1_peptide.txt
						MHCCLM3_QE_Run1_protein.txt
						MHCCLM3_QE_Run1_psm.txt
		CHPP_LM3_RP*_2	MHCCLM3 cells; whole cell lysate	Orbitrap Q Exactive	MHCCLM3.fa	MHCCLM3_QE_Run2_peptide.txt
						MHCCLM3_QE_Run2_protein.txt
						MHCCLM3_QE_Run2_psm.txt
	PXD001305	20140529_QE7_ UPLC9_RJC_DEV_ Exp3_faster_1ug_01	Hela cells; whole cell lysate	Orbitrap Q Exactive	HeLa.fa	Hela_QE_peptide.txt
						Hela_QE_protein.txt
						Hela_QE_psm.txt

Note:

* Represents the fraction number.

Supplementary Table S8. Sequences of probes used for RFP isolations

ID	Sequence(5'→3')
RNA45S5-R	ACGCACGAGCCGAGTGATCCACCGCTAAGAGTCGTACGAG
RNA5-8S5-R	TTCTTCATCGACGCACGAGCCGAGTGATCCACCGCTAAGAGTC
RNA28S5-369	ACTCTCTCTTCAAAGTTCTTTTCAACTTTCCCTTAC
RNA28S5-1623	GTTCGATTAGTCTTTCGCCCCT
RNA28S5-2368	CAAAGTTCTCGTTTGAATAT
RNA28S5-3237	CCCGCCGCAGCTGGGGCGATCCACGGGAAGGGCCCGGCTCGCG TCCAGAGTCGC
RNA18S5-285	GCGTGCGATCGGCCCGAGGTTATCTAGAGTCACCAAA
RNA18S5-154	GGTCAGCGCCCGTCGGCATGTATTAGCTCTAGAATTACCA
RNA5S1-82	AAAGCCTACAGCACCCGGTATTCCCAGGCGGTCTCCCATC
Mir21-1-R:	GTCAACATCAGTCTGATAAGCTAC
Mir21-2-R:	CAGCCCATCGACTGGTGTTGC

Supplementary Table S9. Species and sources of genomic reference sequences

Species	Source
Bacteria	All bacterial complete genomic sequences in NCBI database, 3499 species (downloaded on June 21, 2015)
S. cerevisiae	http://www.yeastgenome.org/
C. elegans	http://legacy.wormbase.org/
Fruitfly	http://www.fruitfly.org/sequence/dlcDNA.shtml
Zebrafish	http://hgdownload.soe.ucsc.edu/downloads.html
Lizard	http://hgdownload.soe.ucsc.edu/downloads.html
Chicken	http://hgdownload.soe.ucsc.edu/downloads.html
Mouse	http://hgdownload.soe.ucsc.edu/downloads.html
Rat	http://hgdownload.soe.ucsc.edu/downloads.html

Guinea pig	http://hgdownload.soe.ucsc.edu/downloads.html
Orangutan	http://hgdownload.soe.ucsc.edu/downloads.html
Gorilla	http://hgdownload.soe.ucsc.edu/downloads.html
Human (hg19)	http://hgdownload.soe.ucsc.edu/downloads.html

Supplementary Table S12: Number of entries of the cell-specific reference databases and their file sizes. LMW = low molecular weight.

Total protein database			
Reference database	NP number	NR_ORF number	File Size (MB)
H1299&A549&HBE	18249	6334	13.6
A549 & HBE	16735	4784	10.6
H1299 & HBE	17740	6060	11.5
Hep3B	20495	9791	13.5
MHCC97H	20495	10691	13.8
MHCCLM3	20704	10563	13.7
HeLa	10563	4352	9.8
Low molecular weight (LMW) protein database			
HBE-LMW	2914	3895	0.9
A549-LMW	2912	3489	0.9
H1299-LMW	2898	3915	1
Hep3B-LMW	3797	9187	1.6
MHCC97H-LMW	3899	10027	1.7
MHCCLM3-LMW	3899	9110	1.7
